# The ClpXP protease is dispensable for degradation of unfolded proteins in *Staphylococcus aureus*

**DOI:** 10.1038/s41598-017-12122-y

**Published:** 2017-09-18

**Authors:** Steen G. Stahlhut, Abdulelah A. Alqarzaee, Camilla Jensen, Niclas S. Fisker, Ana R. Pereira, Mariana G. Pinho, Vinai Chittezham Thomas, Dorte Frees

**Affiliations:** 1 Department of Veterinary Disease Biology, University of Copenhagen, 1870 Frederiksberg C, Denmark; 2 Center for Staphylococcal Research, Department of Pathology and Microbiology, University of Nebraska Medical Center, Omaha, Nebraska 68198 USA; 3 Bacterial Cell Biology, Instituto de Tecnologia Química e Biológica António Xavier, Universidade Nova de Lisboa, Oeiras, Portugal

## Abstract

In living cells intracellular proteolysis is crucial for protein homeostasis, and ClpP proteases are conserved between eubacteria and the organelles of eukaryotic cells. In *Staphylococcus aureus*, ClpP associates to the substrate specificity factors, ClpX and ClpC forming two ClpP proteases, ClpXP and ClpCP. To address how individual ClpP proteases impact cell physiology, we constructed a *S. aureus* mutant expressing ClpX with an I_265_E substitution in the ClpP recognition tripeptide of ClpX. This mutant cannot degrade established ClpXP substrates confirming that the introduced amino acid substitution abolishes ClpXP activity. Phenotypic characterization of this mutant showed that ClpXP activity controls cell size and is required for growth at low temperature. Cells expressing the ClpX_I265E_ variant, in contrast to cells lacking ClpP, are not sensitive to heat-stress and do not accumulate protein aggregates showing that ClpXP is dispensable for degradation of unfolded proteins in *S. aureus*. Consistent with this finding, transcriptomic profiling revealed strong induction of genes responding to protein folding stress in cells devoid of ClpP, but not in cells lacking only ClpXP. In the latter cells, highly upregulated loci include the urease operon, the pyrimidine biosynthesis operon, the *betA-betB* operon, and the pathogenicity island, SaPI5, while virulence genes were dramatically down-regulated.

## Introduction

In all organisms ATP-dependent proteases are essential for maintaining protein homeostasis by disposing of damaged or unneeded proteins, as well as for the conditional degradation of functional proteins in response to external or internal signals^1^. In the cytosol and nucleus of eukaryotic cells, ATP-dependent proteolysis depends exclusively on the 26S proteasome. In contrast, bacteria and eukaryotic organelles of bacterial origin have multiple ATP-dependent proteases^1^. The ClpP proteolytic complexes are examples of ATP-dependent proteases that are highly conserved between eubacteria and the chloroplasts and mitochondria of eukaryotic cells^2^. The ClpP proteolytic complexes are compartmentalized proteases composed of separately encoded proteolytic subunits and ATPase subunits. The active sites for peptide-bond cleavage reside in the ClpP subunits; two homo-heptameric rings of ClpP subunits form a proteolytic barrel that sequesters the active sites within a protected chamber^3,4^. To prevent random degradation of larger peptides and proteins, access to the inner proteolytic chamber is restricted by narrow pores that allow passage of only very small, unfolded peptides. In order to degrade protein substrates, ClpP must associate to hexameric rings of one of several possible Clp ATPases^5^. The Clp ATPases are responsible for substrate recognition, either directly, or in a process that may be modulated by specific adaptor proteins^6^. Bound substrates are subsequently unfolded at the expense of ATP, and the denatured polypeptide is processively translocated into the inner chamber for degradation^7,8^. The Clp ATPases constitute a family of closely related proteins that are divided into subfamilies based on the basis of the presence of specific signature sequences and the number and spacing of the nucleotide binding sites^9^. All Clp ATPases function as molecular chaperones by assisting protein-folding and protein-protein interactions. However, only a subgroup of the Clp ATPases function as specificity factors of ClpP-proteases, a property that is associated with the presence of a specific ClpP recognition IGF tripeptide^10,11^. Bacterial ClpP makes use of several specificity factors: in Gram-negative bacteria ClpP typically associates to ATPases of the ClpA and ClpX families, whereas ClpP associates to ATPases of the ClpX, ClpC, or ClpE families in Gram-positive bacteria^9^. In mammalian mitochondria, ClpX appears to be the only specificity factor of the ClpP protease, and, hence, the ClpXP protease is the most universally conserved ClpP proteolytic complex^12^. The ClpXP protease is also the best characterized ATP-dependent protease at the biochemical level, and detailed insight into the mechanistic features of ATP-dependent proteolysis has been obtained from sophisticated single molecule *in vitro* studies of the ClpXP protease^4^.

Inactivation of *clpP* in bacteria has clearly demonstrated that ClpP proteases contribute to survival and growth of diverse bacteria under conditions of stress^9^. Misfolded proteins present a major problem to cells stressed by heat shock and other stressful conditions, and therefore the protein homeostasis function of the ClpP proteases is especially important in stressed cells^9^. Additionally, inactivation of *clpP* is often associated with pronounced phenotypic traits such as decreased virulence, altered resistance to antibiotics, altered motility, and defects in developmental transitions such as genetic competence and sporulation^9,13–19^. The described phenotypes of *clpP* deletion mutants are conferred by a complete loss of ClpP proteolytic activity and to our knowledge the contribution of single ClpP proteolytic complexes to bacterial cell physiology remains to be exploited. The present study was undertaken to investigate the role of the ClpXP protease in the important pathogenic bacterium *Staphylococcus aureus*. *S. aureus* encode two ClpATPases, ClpX and ClpC that can associate with ClpP to form ClpP proteolytic complexes^20^. In order to inactivate only the ClpXP protease, we constructed a *S. aureus* mutant variant of ClpX that cannot interact with ClpP, hence, the constructed mutant retains ClpCP activity. This mutant cannot degrade established ClpXP substrates confirming that the introduced amino acid substitution abolishes ClpXP activity. Phenotypic characterization of this mutant supports that ClpXP and ClpCP perform different tasks in *S. aureus*, and that ClpC is superior to ClpX in targeting stress-damaged proteins for degradation by ClpP. Hence, bacteria seem to benefit from the use of multiple ClpP specificity factors because Clp ATPases are directed to different groups of substrates thereby expanding the repertoire of substrates degraded by ClpP.

## Results

### A single amino acid substitution in the ClpP recognition sequence of ClpX prevents degradation of known ClpXP substrates

**Figure 1:**
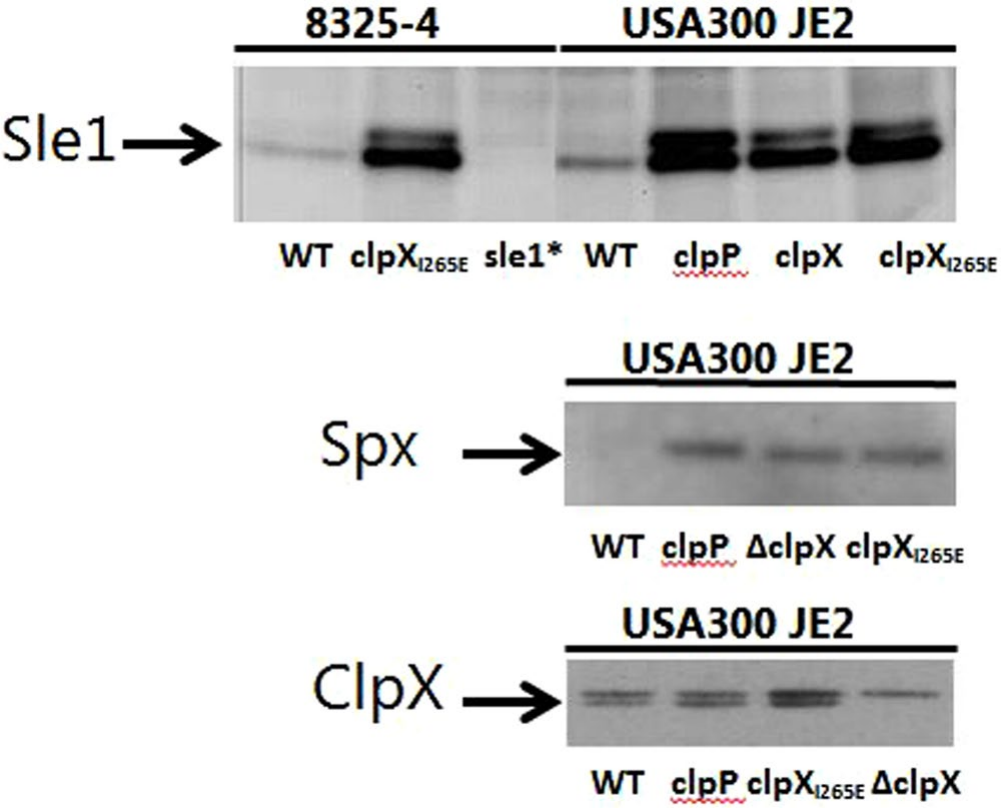
ClpXP specific substrates accumulate in *S. aureus* cells expressing the ClpX_I265E_ variant. Cells were derived from exponentially growing (OD_600_ = 0.8 +/− 0.1) cultures of the JE2 and 8325-4 wild-type strains and the mutant derivatives listed in the figure (* the *sle1* ^−^ strain expresses the ClpX_I265E_ variant). Cell extracts were separated by SDS/PAGE, electrotransferred to a PVDF membrane, and subjected to immunoblotting using Sle1 (**a**), Spx (**b**), or ClpX (**c**) specific antibodies. Each Western blot analysis was performed twice with similar results. Full-length blots are presented in Supplementary Figure 1.

Clp ATPases engaging in complexes with ClpP possess a surface exposed IGF tripeptide required for ClpP interaction^10^. In *S. aureus* ClpX, the conserved ClpP recognition tripeptide is localized at position 265–267. To create a *S. aureus* mutant that lacks ClpXP protease while retaining ClpCP protease activity, we used site-directed mutagenesis and allelic replacement to introduce an I_265_E substitution in the IGF tripeptide of ClpX, as described in the experimental section. As our parental strain, we used *S. aureus* JE2, a derivative of the multiple antibiotic resistant and community-acquired USA300 clone that is rapidly spreading worldwide^21^. To examine if *S. aureus* cells expressing the ClpX_I265E_ variant are devoid of ClpXP proteolytic activity, we assessed if two known ClpXP substrates, the transcriptional regulator Spx and the Sle1 autolysin, accumulate in the ClpX_I265E_ mutant^22,23^. Western blot analysis using Sle1 and Spx specific antibodies revealed strong accumulation of both Sle1 and Spx in the ClpX_I265E_ mutant strain (Fig. 1). The cellular levels of Spx and Sle1 expressing the ClpX_I265E_ variant are comparable to the levels in cells devoid of ClpP or ClpX, supporting that the introduced amino acid change in ClpX indeed eliminates activity of the *S. aureus* ClpXP protease. The Sle1 antibody recognized two bands of similar sizes that both disappear if the *sle1* gene is disrupted, and we speculate that the two bands represent Sle1 with and without signal sequence (top panel, Fig. 1). Next, we used ClpX specific antibodies to determine the amount of ClpX_I265E_ expressed in the mutant strain. As can be seen in Fig. 1 (lower panel), the ClpX_I265E_ variant was expressed in the same amount as ClpX in wild-type cells, ruling out that differential expression of ClpX contributes to accumulation of Spx and Sle1 in the mutant expressing the ClpX_I265E_ variant.

**Figure 2:**
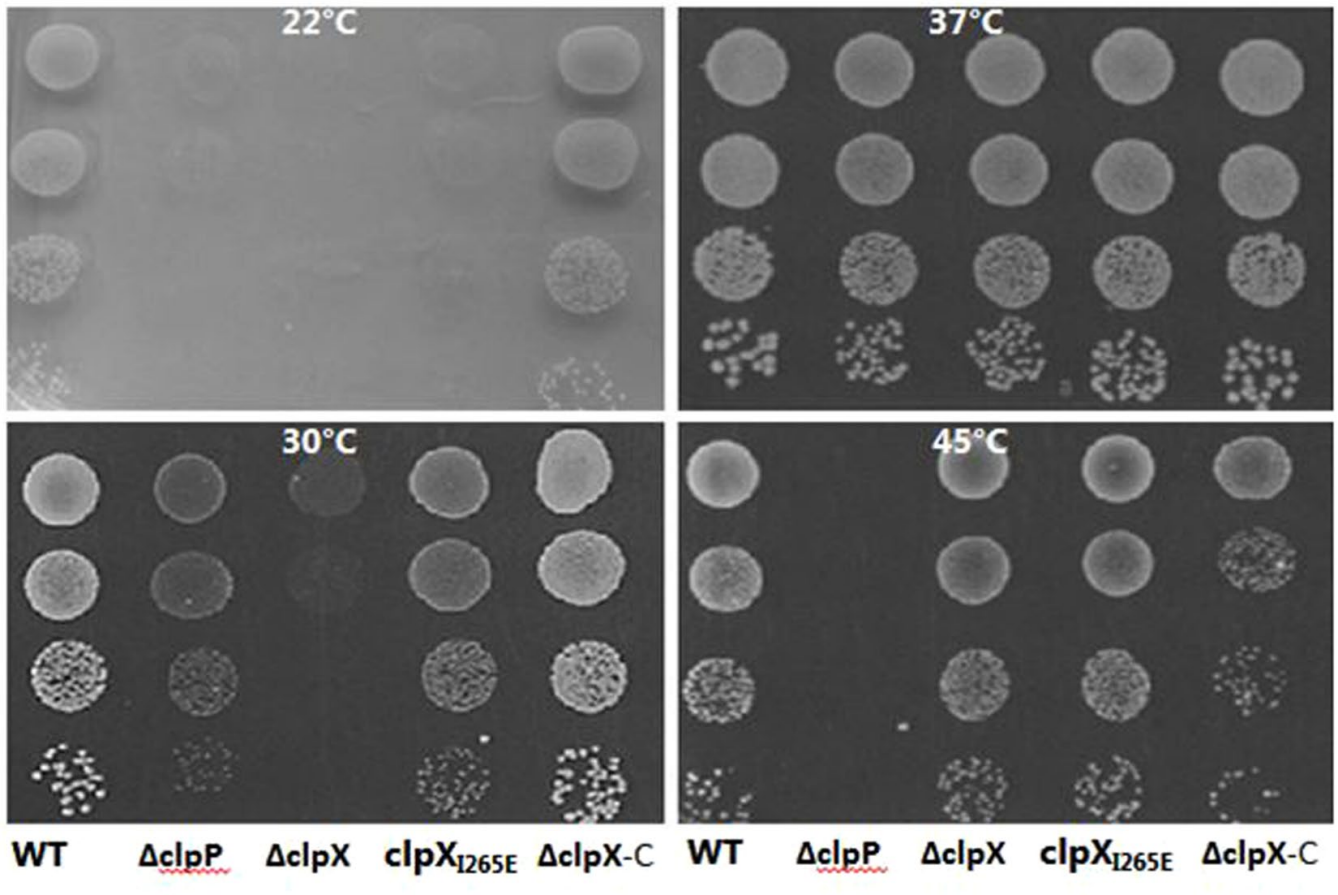
*S. aureus* expressing the ClpX_I265E_ variant are cold-sensitive but not heat-sensitive. The wild-type *S. aureus* strain JE2 and the indicated mutants were grown exponentially in TSB at 37 °C. At OD_600_ = 0.5, the cultures were diluted 10^1^, 10^2^, 10^3^ and 10^4^-fold, and 10 μL of each dilution was spotted on TSA plates that were subsequently incubated at the indicated temperatures.

In *E. coli*, the I to E substitution did not interfere with the ClpP-independent ability of ClpX to re-fold ClpX chaperone substrates such as the MU-transposase, demonstrating that this mutant variant of ClpX retains ClpP-independent chaperone activity^10^. Due to the lack of confirmed ClpX chaperone substrates in *S. aureus*, we were unable to test directly if ClpP-independent ClpX chaperone activity is maintained in the *S. aureus* ClpX_I265E_ variant. However, from previous studies we know that ClpX independently of ClpP is required for growth of *S. aureus* at 30 °C^23^. This growth defect of the *clpX* mutant is rescued by mutations in *ltaS* (LTA synthase), indicating that ClpX independently of ClpP assists processes related to cell wall biogenesis^23^. We therefore used this phenotype to indirectly assess if the ClpX_I265E_ variant retains chaperone activity. To this end, early exponential cells of the wild-type and the various mutants were spotted onto TSA plate and incubating at 30 °C for 24 hours. From this assay, it is clear that the *clpX* deletion mutant has a severe growth defect at 30 °C that is fully complemented by introducing a wild-type copy of the *clpX* gene (Fig. 2). Importantly, this severe growth defect is not shared by cells expressing ClpX_I265E_, suggesting that the ClpX_I265E_ variant retains ClpP independent chaperone activity. In conclusion, the presented results support that the I_265_E amino acid substitution in the ClpP recognition motif of *S. aureus* ClpX prevents ClpX-ClpP interaction without interfering with ClpP independent functions of ClpX.

### *S. aureus* cells expressing the ClpX_I265E_ variant are significantly reduced in size

Under normal laboratory growth conditions the ClpX_I265E_ mutant grows with a generation time that was only slightly (<10%) reduced as compared to the USA300 JE2 parental strain. However, the ClpX_I265E_ mutant culture reached a final yield (OD_600_ = 8.3 +/− 0.1) that was substantially lower than the yield reached by the JE2 wild-type culture (OD_600_ = 12.0 +/− 0.6). Despite, these differences in the final OD, we did not observe significant differences in the number of cfu/ml in ON-cultures of the wild-type and mutant strain (approximately 1 × 10^10^ cfu/ml for both strains). This prompted us to measure the size of the ClpX_I265E_ mutant cells, and indeed the mean diameter of mutant (=0.92 μm +/− 0.06) is significantly reduced (P < 0.001) compared to the mean diameter of wild-type cells (=1.16 μm +/− 0.09). The detected reduction of the cell diameter is equivalent to a two-fold decrease in cell volume for the mutant cells. Hence, the ClpXP protease contributes to processes determining *S. aureus* cell-size.

### *S. aureus* cells expressing the ClpX_I265E_ variant cannot form colonies at 22 °C but are not sensitive to heat-stress

As shown above, the *clpX*_*I265E*_ mutant grows better than mutants completely lacking *clpX* at 30 °C. However, the JE2*clpX*_*I265E*_ mutant and the JE2 *clpP* mutant also form slightly smaller colonies than the JE2 wild-type at 30 °C (Fig. 2), suggesting that ClpXP protease activity is important for growth at low temperatures. In confirmation hereof, the JE2*clpX*_*I265E*_ mutant and the JE2 *clpP* mutant in contrast to wild-type cells were unable to form colonies after 72 hours incubation at 22 °C. This finding is in agreement with previous reports showing severely reduced growth of *clpP* deletion mutants at 20 °C or 15 °C and we here attribute this phenotype to the lack of ClpXP activity^17,24^.

The cold-sensitive phenotype associated with deletion of *clpP* was suggested to be caused by accumulation of misfolded and aggregated proteins in cells lacking ClpP protease activity^24^. In order to test if ClpXP protease activity is required for growth of *S. aureus* under conditions known to generate unfolding of proteins, growth was examined under heat-stress conditions. Early exponential cells of JE2 wild-type and mutant strains were spotted on TSA plates and incubated for 24 h at 37 °C, or at 45 °C. As shown in Fig. 2, cells expressing the ClpX_I265E_ variants, similar to the *clpX* deletion mutants, grow as well as the wild-type at 45 °C, while cells lacking *clpP*, were unable to grow at this temperature (Fig. 2). Similar results were obtained, when the heat sensitivity of the ClpX_I265E_ mutant was examined in different strain-backgrounds (the clinical isolate, SA564, and the lab strains, 8325-4 and Newman) – data not shown. Taken together, these data suggest that *S. aureus* cells ClpXP activity is more important for growth of *S. aureus* at low temperatures than for growth at high temperatures.

### *S. aureus* cells expressing the ClpX_I265E_ variant do not accumulate protein aggregates at high temperatures

**Figure 3:**
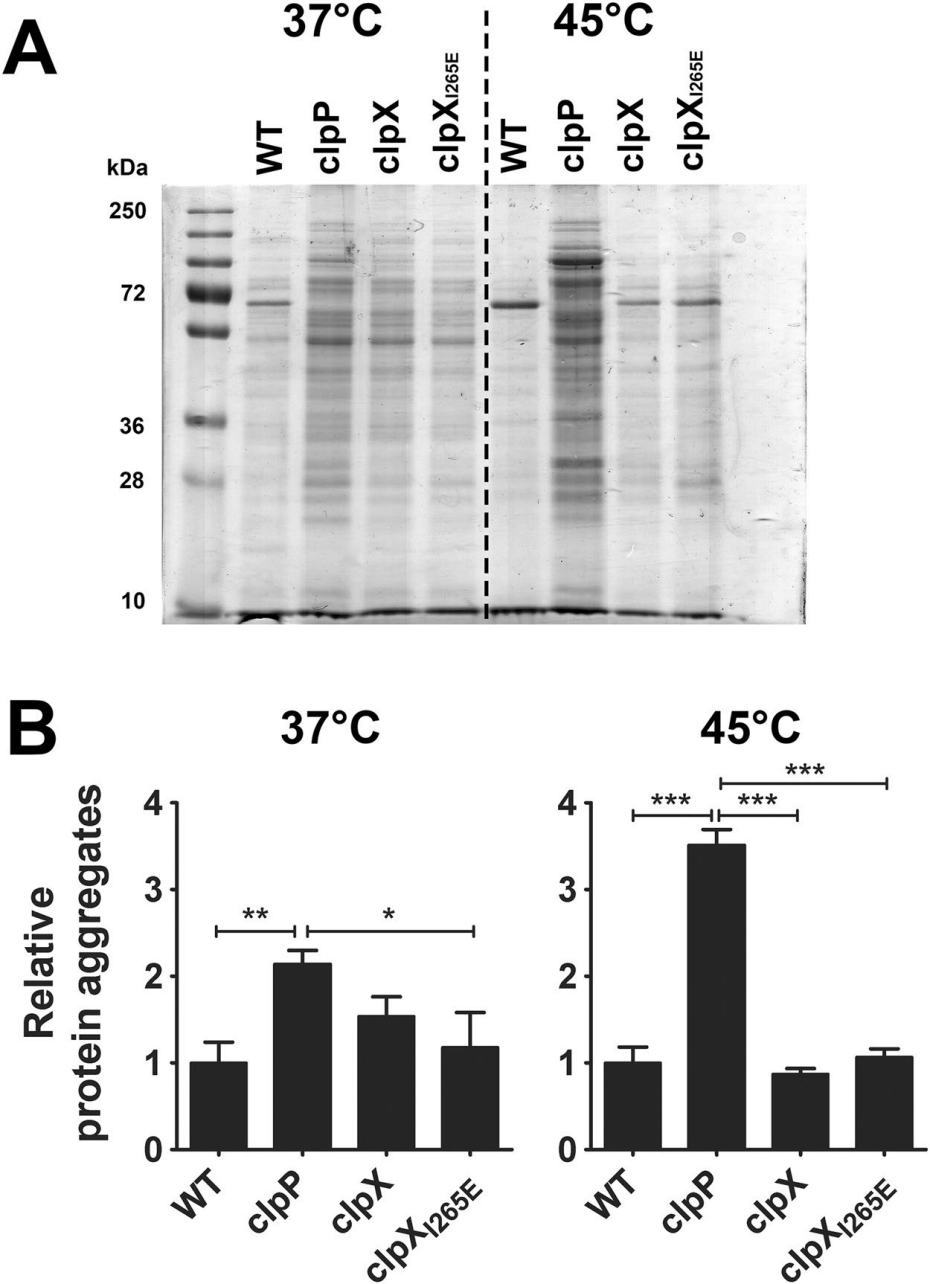
Protein aggregates accumulate in heat-shocked JE2 Δ*clp*P mutant but not in the JE2 expressing the ClpX_I265E_ variant. (**A**) Bacterial cells of the JE2 wild-type and mutant strains were grown aerobically at 37 °C in 25 ml TSB in a 250 ml flask for 3 h. At this point, the cultures in each flask were split equally with one half incubated at 37 °C and the other at 45 °C for an additional 3 h. Protein aggregates were isolated from 10 ml of culture. The solubilized protein aggregates was normalized to the total protein in the crude extract and analyzed by SDS-PAGE. (**B**) Protein aggregates were quantified densitometrically from coomassie blue G-250 stained gels using image J software (mean ± SD, n = 3, One-way ANOVA, Tukey’s post-test; *P < 0.05, **P < 0.005; ***P < 0.0005).

Due to the exposure of hydrophobic residues, normally buried in the native structure, misfolded proteins are prone to aggregate. To more directly estimate the level of misfolded protein in the different strains, aggregate proteins were isolated from JE2 wild-type and mutant strains grown at 37 °C or 45 °C for 3 hours. As depicted in Fig. 3, similar amounts of aggregate protein were isolated from JE2 wild-type cells incubated at 37 °C and 45 °C. In contrast, the amount of aggregate protein isolated from cells completely lacking ClpP activity was significantly elevated after incubation at 45 °C, demonstrating that ClpP is important for preventing protein aggregation in heat-shocked cells. Moreover, as the level of aggregate proteins in the *clpP* mutant was significantly higher than the wild-type level even at non-stress conditions (37 °C), ClpP-mediated proteolysis is also important for preventing protein aggregation in non-stressed cells. Interestingly, the level of aggregate proteins in heat-shocked JE2ΔclpX and JE2 cells expressing the ClpX_I265E_ variant did not exceed the wild-type level, showing that neither the ClpXP protease, nor the ClpX chaperone, are required to combat protein aggregation at high temperatures in *S. aureus*. Notably, while the profile of aggregate protein isolated from wild-type and the two *clpX* mutants at high temperature had similar appearance, the pattern of aggregate proteins isolated from 37 °C-cultures of the *clpX* mutants appeared different from the wild-type pattern, as the most abundant band in the wild-type profile at both temperatures was absent from the mutants at 37 °C (Fig. 3).

We conclude that *S. aureus* cells expressing the ClpX_I265E_ variant, in contrast to cells lacking ClpP, are not sensitive to heat-stress and do not accumulate protein aggregates at high temperatures, showing that ClpXP is dispensable for degradation of unfolded proteins in *S. aureus*.

### Transcriptomic analysis supports distinctive roles for the ClpXP and ClpCP proteases

In *S. aureus*, deletion of *clpP* dramatically changes global gene expression with hundreds of genes and proteins belonging to diverse pathways being affected^17,24,25^. With the ClpX_I265E_ variant in hand, we could now more specifically determine changes in gene-expression caused by inactivation of only one Clp protease, and thereby further dissect the global impact of the ClpP proteases on *S. aureus* cell physiology. To do so RNA-sequencing was performed on RNA samples obtained from mid-exponential cultures of JE2 wild-type, *clpP* and *clpX*_I265E_ mutants. Genes that change expression due to the inactivation of the ClpXP protease can be identified directly by comparing the transcriptome of the JE wild-type and the *clpX*_*I265E*_ mutant. ClpXP-regulated genes are expected to have similar expression in the *clpP* mutant and in the *clpX*_*I265E*_ mutant. Genes that are differentially expressed between the *clpP* mutant and the *clpX*_*I265E*_ mutants likely represent genes that change expression due to the absence of ClpCP.

### ClpXP affected genes

The complete list of genes expressed differentially between the JE2 wild-type and the *clpX*_*I265E*_ mutant can be found in Supplemental Table 1.

### Genes up-regulated in JE2clpX_I265E_ relative to wild-type

Genes up-regulated more than 3 fold in *clpX*_*I265E*_ mutant cells compared to wild-type cells are shown in Table 1. The genes most highly upregulated (5–10 fold) in the *clpX*_*I265E*_ mutant are located in one of four genetic loci: the urease operon (*ureABCEFGHD*), the operon encoding enzymes involved in uptake and synthesis of pyrimidines (*pyrPBC-carAB-pyrFE*), the staphylococcal phathogenicity island (=SaPI5, as discussed below), or the *betA-betB* operon and the downstream *betT* gene encoding, respectively, enzymes catalysing the formation of glycine betaine from choline, and a choline uptake system^26^. In bacteria glycine betaine and other small organic molecules function as osmoprotectants during osmotic stress^27^ and notably the *OpuCA-OpuCD* operon encoding the ABC uptake system OpuC involved in the uptake of the compatible solute carnitine^28^ is also among the most significantly up-regulated genes in the *clpX*_*I265E*_ mutant.

**Table 1:** Genes up-regulated in JE2 expressing ClpX_I265E_ relative to JE2 wild-type (3 fold cut-off).

Gene	Predicted Function	Fold change JE2X_265_/JE2 wt	Padj
ureA	urease, gamma subunit	27,0	8,31E-102
ureB	urease, beta subunit	21,0	8,21E-130
ureC	urease, alpha subunit	18,0	1,13E-117
ureE	urease accessory protein UreE	15,0	4,02E-100
ureF	urease accessory protein UreF	13,0	7,84E-72
SAUSA300_2237	putative urea transporter	12,0	2,50E-45
ureG	urease accessory protein UreG	12,0	8,02E-92
ureD	urease accessory protein UreD	11,0	4,50E-81
SAUSA300_0808	SaPI5-gene	9,0	1,27E-17
SAUSA300_0804	SaPI5-gene putative transcriptional regulator	9,0	7,93E-23
SAUSA300_0807	SaPI5-gene	7,0	2,08E-14
SAUSA300_0805	SaPI5 gene predicted to encode an excisionase	7,0	3,18E-14
**betB**	**glycine betaine aldehyde dehydrogenase**	7,0	5,48E-09
**betA**	**choline dehydrogenase**	7,0	2,08E-08
pyrP	uracil permease	6,0	1,77E-15
pyrC	dihydroorotase	6,0	4,49E-18
pyrB	aspartate carbamoyltransferase	5,0	2,27E-14
SAUSA300_0809	SaPI5-gene predicted to encode DNA primase	5,0	1,24E-15
SAUSA300_0806	SaPI5-gene	5,0	1,08E-08
carA	carbamoyl-phosphate synthase, small subunit	4,0	1,39E-18
carB	carbamoyl-phosphate synthase, large subunit	4,0	5,71E-20
pyrF	orotidine 5′-phosphate decarboxylase	4,0	1,20E-17
SAUSA300_2523	conserved hypothetical protein	4,0	3,40E-32
SAUSA300_0810	SaPI5-gene	4,0	1,01E-11
SAUSA300_2524	conserved hypothetical protein	3,0	3,72E-17
purA	adenylosuccinate synthetase	3,0	5,86E-28
**betT**	**choline/carnitine/betaine transporter**,	3,0	7,55E-12
SAUSA300_0811	SaPI5-gene	3,0	5,76E-05
fnbA	fibronectin binding protein A	3,0	1,38E-10
SAUSA300_2522	conserved hypothetical protein	3,0	1,96E-08
**opuCd**	**glycine betaine/choline transport system**	3,0	2,67E-29
gltA	citrate synthase II	3,0	8,52E-24
cysM	cysteine synthase/cystathionine beta-synthase	3,0	9,12E-27
SAUSA300_0079	putative lipoprotein	3,0	7,29E-42
metB	cystathionine gamma-synthase	3,0	4,11E-23

^*^Genes are color coded according to putative function as described in the text.

Additional loci that encode proteins with diverse or unknown functions were highly significantly induced in the *clpX*_*I265E*_ mutant; among them certain surface proteins of importance for *S. aureus* virulence such as fibronectin binding proteins, FnbA and FnbB, and the large adhesion SraP, Supplemental Table 1 ^30^. *sraP* is the last gene in an operon additionally encoding proteins required for glycosylation and export of the SraP adhesin, and the entire operon is significantly up-regulated in the *clpX*_*I265E*_ mutant^29^.

### Genes down-regulated in JE2clpX_I265E_ relative to wild-type

Genes down-regulated more than 3 fold in the *clpX*_*I265E*_ mutant compared to the JE2 wild-type are shown in Table 2. The most dramatically down-regulated locus in cells expressing the ClpX_I265E_-variant are two adjacent genes, SAUSA300_2453 and SAUSA300_2454 encoding a protein with homology to the ATP-binding protein of ABC transporters and a putative membrane spanning protein, respectively. The list encompasses additional genes encoding proteins with a predicted function in transport as well as several loci encoding lipoproteins. Strikingly, however, the list of very significantly down-regulated loci is dominated by genes with a function in virulence and virulence gene regulation. Among the most down-regulated virulence genes are the *spa* and *sbi* genes encoding the IgG binding proteins, Protein A and Sbi. Notably, the *spa* gene is the most highly expressed gene in exponentially growing JE2 wild-type cells (Supplemental Table 1). Other down-regulated virulence genes encode secreted enzymes such as *nuc* (nuclease), *tig* (lipase) and *sak* (staphylokinase). The list of genes encoding virulence regulators that are down-regulated more than 2 fold in the mutant comprises the *mgrA, sarS, sarZ, agrA*, *rnaIII* and *arlR* genes.

**Table 2:** Genes down-regulated in expressing ClpX_I265E_ relative to JE2 wild-type (0.32 fold cut-off).

Gene	Predicted Function	Fold Change JE2 wt/X_265_	Padj
SAUSA300_2453	ABC transporter, ATP-binding protein	77,3	1,3E-91
SAUSA300_2454	Conserved hypothetical protein	55,1	1,5E-97
spa	immunoglobulin G binding protein A precursor	9,6	9,3E-09
SAUSA300_0307	5′-nucleotidase, lipoprotein e(P4) family	7,8	8,8E-46
SAUSA300_1890	staphopain A	7,7	2,3E-47
chs	chemotaxis-inhibiting protein CHIPS	7,5	1,3E-09
SAUSA300_0136	cell wall surface anchor family protein	5,7	3,4E-58
tig	triacylglycerol lipase precursor	5,0	1,7E-76
nuc	thermonuclease precursor	4,5	2,8E-20
mgrA	transcriptional regulator, MarR family	4,5	6,7E-53
SAUSA300_2248	transcriptional regulator, AraC family	4,2	4,9E-19
SAUSA300_0798	ABC transporter, substrate-binding protein	4,2	7,6E-41
SAUSA300_1211	conserved hypothetical protein	4,0	1,1E-15
sbi	IgG-binding protein SBI	3,9	3,6E-16
SAUSA300_0372	putative lipoprotein	3,9	6,9E-10
SAUSA300_0359	trans-sulfuration enzyme family protein	3,7	1,2E-07
SAUSA300_0797	ABC transporter permease protein	3,7	9,0E-15
SAUSA300_0846	Na+/H+ antiporter family protein	3,7	1,5E-14
SAUSA300_0358	putative 5-methyltetrahydrofolate–homocysteine methyltransferase	3,6	6,0E-15
sak	staphylokinase precursor	3,6	7,5E-22
ear	Ear protein	3,6	4,7E-11
ilvD	dihydroxy-acid dehydratase	3,6	2,0E-09
ilvC	ketol-acid reductoisomerase	3,5	6,6E-22
ilvB	acetolactate synthase, large subunit	3,5	4,7E-20
comK	competence transcription factor	3,4	2,5E-14
SAUSA300_0796	ABC transporter, ATP-binding protein	3,4	2,41E-15
SAUSA300_1029	iron transport associated domain protein	3,4	3,82E-14
SAUSA300_2417	putative transporter	3,4	2,2E-33
sarS	staphylococcal accessory regulator	3,3	3,13E-09
asd	aspartate semialdehyde dehydrogenase	3,3	9,72E-16
brnQ	branched-chain amino acid transport system II carrier protein	3,2	4,23E-39
leuA	2-isopropylmalate synthase	3,2	7,55E-20
SAUSA300_0435	ABC transporter, ATP-binding protein	3,1	1,78E-11
dapB	dihydrodipicolinate reductase	3,1	6,14E-16
plc	1-phosphatidylinositol phosphodiesterase	3,1	1,2E-10
SAUSA300_0436	ABC transporter, permease protein	3,0	1,55E-10
SAUSA300_0010	putative membrane protein	3,0	4,31E-14
agrA	accessory gene regulator protein A	3,0	1,78E-06
leuB	3-isopropylmalate dehydrogenase	3,0	1,89E-13

*Genes with a predicted function in virulence are shaded grey.

### Genes with altered expression between the JE2 clpP mutant and JE2clpX_I265E_ (putative ClpCP affected genes)

The complete list of genes expressed differentially between the JE 2 *clpP* mutant and the *clpX*_*I265E*_ mutant are listed in Supplemental Table 2.

### Genes up-regulated in the JE2clpP^−^ mutant relative to JE2clpX_I265E_

The most highly up-regulated genes (>4 fold) in the JE2 *clpP* mutant compared to JE2 expressing the *clpX*_*I265E*_ variant are listed in Table 3. Interestingly, almost all of these genes belong to only three functional groups: the first group comprises loci responding to protein oxidation stress (the *ahpC-ahpF* operon and the downstream gene SAUSA300_0381), or protein folding stress (the *ctsR-mcsA-mcsB- clpC* operon, and the *clpB*-gene). Additionally, the *hrcA-grpE-dnaK-dnaJ-prmA* operon, and the *groEL-groES* operon are significantly upregulated – Supplemental Table 2. These genes are expressed at wild-type or slightly elevated levels in the *clpX*_*I265E*_ mutant. The second functional group of genes highly upregulated in the *clpP* mutant compared to the *clpX*_*I265E*_ mutant are genes involved in iron metabolism: Nine genes belonging to an operon encoding proteins catalyzing the biosynthesis of staphyloferrrin B (*sbnABCDEFGHI*) are among the most highly induced genes. Similarly, other genes with a confirmed role in iron homeostasis, such as genes encoding proteins with a role in staphyloferrin A transport and synthesis (*htsAB; sfaCBAD*), and the Fe-S cluster biosynthesis operon *sufCDSUB* are also among the most significantly up-regulated genes in the *clpP* mutant compared to the *clpX*_*I265E*_ mutant (Table 3 and Supplemental Table 2). Transcription of siderophore synthesis genes is controlled by the Ferric Uptake Regulator, Fur, family of transcriptional regulators, and a gene belonging to this family (SAUSA300_1448) is among the most significantly upregulated genes between the JE2 *clpP* mutant and the JE2 *clpX*_*I265E*_ mutant. In general, genes involved in iron metabolism are transcribed at wild-type levels in the *JE2 clpX*_*I265E*_ mutant, showing that loss of ClpXP activity does not alter expression of these genes.

**Table 3:** Genes up-regulated in JE2clpP::ΦNΣ relative to JE2 expressing ClpX_I265E_ (4 fold cut-off).

Gene*	Predicted Function*	Fold change P/X_265_	Padj
clpB	Chaperone clpB	27,9	2,8E-172
sbnA	Staphyloferrin B biosynthesis	12,8	3,1E-37
mcsA	Regulator of CtsR activity	12,5	5,9E-168
mcsB	ATP guanido phosphotransferase	10,9	4,7E-201
sbnB	Staphyloferrin B biosynthesis	10,8	6,3E-36
clpC	Chaperone ClpC, ClpP recognition factor	10,4	0,0E + 00
ctsR	transcriptional regulator CtsR	9,2	0,0E + 00
SAUSA300_1934	ϕSA3usa prophage, phage major tail protein	9,1	2,0E-31
SAUSA300_1923	ϕSA3usa prophage putative autolysin	8,5	3,5E-24
SAUSA300_1935	ϕSA3usa prophage	7,9	1,9E-22
SAUSA300_1958	ϕSA3usa prophage putative single-strand binding protein	7,0	2,9E-12
ahpF	alkyl hydroperoxide reductase, subunit F	7,0	1,1E-87
SAUSA300_1966	ϕSA3usa prophage putative phage anti-repressor protein	6,6	1,4E-10
sbnC	Staphyloferrin B biosynthesis	6,6	1,7E-40
SAUSA300_1930	ϕSA3usa putative phage tail tape measure protein	6,3	8,0E-37
ahpC	Alkyl hydroperoxide reductase subunit C	6,3	7,1E-94
SAUSA300_1937	ϕSA3usa prophage	6,3	2,7E-09
SAUSA300_1960	ϕSA3usa prophage similar to DNA recombination proteins	6,1	3,7E-10
SAUSA300_1929	ϕSA3usa prophage, putative phage tail component	6,1	4,2E-19
SAUSA300_1959	ϕSA3usa prophage gene	5,9	3,4E-08
SAUSA300_1939	ϕSA3usa prophage, with similarity to ClpP protease	5,6	3,2E-19
SAUSA300_1932	ϕSA3usa prophage gene	5,5	4,0E-17
SAUSA300_1957	ϕSA3usa prophage gene	5,5	1,9E-10
sbnD	Staphyloferrin B biosynthesis	5,5	4,8E-33
sbnE	Staphyloferrin B biosynthesis	5,4	2,9E-29
SAUSA300_1964	ϕSA3usa prophage gene	5,4	6,9E-08
SAUSA300_1942	ϕSA3usa prophage gene	4,9	6,7E-10
SAUSA300_1938	ϕSA3usa prophage, putative capsid protein	4,8	6,6E-11
SAUSA300_2453	ABC transporter, ATP-binding protein	4,8	2,3E-11
SAUSA300_1941	ϕSA3usa putative phage terminase, large subunit	4,8	6,3E-12
SAUSA300_1962	ϕSA3usa prophage gene	4,7	1,3E-06
SAUSA300_1943	ϕSA3usa prophage gene	4,6	3,2E-09
SAUSA300_1961	ϕSA3usa prophage gene	4,6	1,1E-07
sbnF	Staphyloferrin B biosynthesis	4,3	8,1E-22
**SAUSA300_0804**	**SaPI putative transcriptional regulator**	4,2	1,8E-12
SAUSA300_1944	ϕSA3usa prophage putative transcriptional activator	4,2	2,7E-08
SAUSA300_1963	ϕSA3usa prophage gene	4,2	7,4E-09
sbnG	HPCH/HPAI aldolase family protein	4,0	2,0E-15

*Genes are color coded according to putative function as described in the text.

The third cluster of genes that is highly up-regulated in *clpP* relative to the *clpX*_*I265E*_ mutant is comprised of ϕSA3usa prophage genes^29^. The induced prophage-genes encode proteins with a function in the lytic life-style of the phage such as the major phage tail protein (SAUSA300_1934), the tail tape measure protein (SAUSA300_1930), and a predicted phage autolysin (SAUSA300_1923). Notably, a gene (SAUSA300_1939) encoding a protein with 30% identity to *clpP* is among the induced ϕSA3usa prophage genes. To our knowledge the function of this putative protease encoded by the φSA3usa prophage has not been investigated. The SAUSA300_1939 gene similar to other lytic pro-phage genes is transcribed at very low levels in wild-type cells or in the ClpX I265E mutant ruling out that this putative protease contributes to the phenotype of cells lacking ClpXP proteolytic activity. However, we cannot rule out that induction of this putative protease may contribute to the phenotype of *clpP* deletion strains. Interestingly, genes in another mobile genetic element, the staphylococcal pathogenicity island, SaPI5, were also significantly upregulated in the *clpP* mutant relative to the *clpX*_*I265E*_ mutant and, moreover, between the *clpX*_*I265E*_ mutant and the wild-type (Table 1).

### Genes down-regulated in JE clpP::ΦNΣ relative to JE2clpX_I265E_

The most down-regulated loci in the *clpP* mutant compared to the *clpX*_*I265E*_ mutant are listed in Table 4. Strikingly, the *agr*-locus encoding the central *S. aureus* virulence regulator, the Agr quorum sensing system as well as the effector molecule, *rnaIII* are among the loci most reduced in expression between the *clpP* mutant and the *clpX*_*I265E*_ mutant. Consistent with this finding, the adjacent genes (SAUSA300_1067 and SAUSA300_1068) encoding the AgrA-controlled toxins, phenol-soluble modulin beta1 and phenol-soluble modulin beta1/2 are also among the 10 most down-regulated genes between the *clpP* deletion mutant and the *clpX*_*I265E*_ mutant. Interestingly, transcription of *rnaIII* and the *agrACDB* operon is also significantly lower in the JE2*clpX*_*I265E*_ mutant than in wild-type cells. Accordingly, this central regulatory locus depends on both the ClpCP and the ClpXP protease for full transcription in the JE2-strain. Apart from these loci, the list of genes positively regulated by ClpCP encompasses a large number of loci encoding proteins of unknown functions – Table 4 and Supplemental Table 2.

**Table 4:** Genes down-regulated JE2clpP::ΦNΣ relative to JE2 expressing ClpX_I265E_ (4 fold cut-off).

Gene*	Predicted Function*	Fold changes¤ P/X_265_	padj
SAUSA300_1180	conserved hypothetical protein	0,11	7,42E-11
agrD	accessory gene regulator protein D	0,12	5,61E-09
SAUSA300_2041	conserved hypothetical protein	0,14	2,01E-11
agrB	accessory gene regulator protein B	0,14	8,10E-09
SAUSA300_1988	rnaIII (hld)	0,16	0,00031
agrC	accessory gene regulator protein C	0,16	1,52E-14
SAUSA300_1432	ϕSA2usa gene	0,16	4,16E-10
agrA	accessory gene regulator protein A	0,18	2,85E-14
SAUSA300_0816	CsbD-like superfamily	0,18	5,21E-21
SAUSA300_0281	esXB	0,19	7,67E-10
SAUSA300_0884	conserved hypothetical protein	0,19	2,86E-16
SAUSA300_1067	phenol-soluble modulin beta1	0,20	0,001278
SAUSA300_0292	conserved hypothetical protein	0,21	4,00E-08
SAUSA300_1068	phenol soluble modulin beta 1/beta 2	0,21	0,001179
SAUSA300_0781	conserved hypothetical protein	0,22	0,000521
mscL	large conductance mechanosensitive channel protein	0,22	3,97E-21
SAUSA300_1021	hypothetical protein	0,23	1,82E-07
SAUSA300_0785	acetyltransferase, GNAT family	0,23	1,82E-10
SAUSA300_2401	addiction module toxin, Txe/YoeB family	0,23	2,07E-21
SAUSA300_0937	conserved hypothetical protein	0,24	1,34E-07
SAUSA300_0723	conserved hypothetical protein	0,25	0,004094
SAUSA300_2361	conserved hypothetical protein	0,25	1,77E-05

*Genes with a predicted function in virulence are shaded grey.

¤Genes with very low expression (normalized read count <30 in all samples were omitted from this table).

## Discussion

In bacterial cells, ClpP generally has the ability to associate to several Clp ATPases thereby forming different ClpP proteolytic complexes. Our knowledge of how individual ClpP proteolytic complexes impact cell physiology remains limited, and we here addressed this issue using *S. aureus* as our model organism. *S. aureus* possess two ClpP proteolytic complexes, ClpXP and ClpCP, and we previously suggested that the ClpCP protease is the major protease for degradation of unfolded proteins in *S. aureus* ^16,17,20^. Consistent with this hypothesis, we here show that while aggregate protein accumulate strongly in heat stressed cells devoid of ClpP, the level of aggregate protein in cells lacking only ClpXP proteolytic activity is similar to the wild-type level. Moreover, *S. aureus* cells lacking only ClpXP grow as well as the wild-type at high temperatures. Hence, ClpXP proteolytic activity is fully dispensable for degrading unfolded proteins in cells possessing a functional ClpCP protease. These, data do not rule out that ClpXP contributes to degradation on misfolded proteins in wild-type cells. However, a functional ClpXP protease is not enough to ensure growth and survival of *S. aureus* under conditions generating massive unfolding of proteins, as inactivation of *clpC*, similar to inactivation of *clpP*, confers a strong heat-sensitive phenotype to *S. aureus* ^17^. Taken together, our results support that ClpC is superior to ClpX in targeting stress-damaged proteins for degradation by ClpP. Consistent with this notion, an intriguing recent study suggested that Gram-positive bacteria use phosphorylation of exposed arginine residues as a tag to specifically mark misfolded proteins for degradation by the ClpCP proteolytic complex and, moreover that, ClpCP-mediated degradation of arginine phosphorylated proteins becomes essential for growth of *Bacillus subtilis* at elevated temperatures^30^. Even under non-stress conditions native proteins are at permanent risk of unfolding^31^. At 37 °C, the amount of aggregate protein in cells lacking ClpXP appeared slightly elevated compared to the wild-type level, however, was much lower than in cells entirely lacking ClpP proteolytic activity. Hence, ClpCP-mediated proteolytic is important for the cell’s protein quality system also under optimal growth conditions. In agreement with the detection of aggregate protein in cells lacking ClpP at 37 °C, the most highly induced loci in the *clpP* deletion mutant, compared to cells lacking only ClpXP or wild-type cells, are operons responding to protein folding stress or protein oxidation stress (the CtsR regulon and the *ahpC-ahpF* operon). Aggregation of unfolded protein results when the capacity of the cell’s protein quality-control systems is exhausted^31^. Thus, in cells lacking ClpP dependent proteolysis, the 10-fold induction of protein quality-control systems is not enough for preventing protein unfolding and subsequent aggregation of proteins underscoring the importance of ClpP mediated proteolysis for removing non-native proteins in *S. aureus* cells growing at optimal temperatures. Again, we cannot rule out that ClpXP contributes to the removal of mis-folded proteins, but our data support that ClpCP is the major protease for performing this task. Of note, Bacillus Spx is critical for the prevention of protein aggregate formation because its regulon encodes redox chaperones, such as thioredoxin, required for protection against thiol-specific oxidative stress. Hence, while we cannot rule out that the high levels of Spx contributes to the low levels of protein aggregates observed in cells lacking ClpXP activity, accumulation of Spx is clearly not enough to prevent protein aggregation if cells are devoid of ClpCP activity^32^.

Unfolded and aggregate proteins are prone to become oxidized in a process accelerated by free Fe-ions that via Fenton chemistry can react with hydrogen peroxide to form the highly reactive hydroxyl radical^33^. Similar to the protein folding stress genes, the nine-gene *sbn*-operon encoding enzymes for siderophore B synthesis is specifically upregulated in the *clpP* mutant, while being expressed at wild-type level in cells lacking only ClpXP activity. Interestingly, siderophore B seems to have a role in combating oxidative stress, as the *sbn*-operon is also among the most highly induced genes in *S. aureus* cells exposed to oxidative stress^34^. We therefore speculate that the protein-folding stress in cells lacking ClpCP activity promotes up-regulation of genes involved in iron-metabolism to prevent irreversible oxidation of misfolded proteins. The final group of genes highly upregulated in cells lacking ClpP activity are located in the ϕSA3usa prophage. In wild-type cells, and in cells only lacking ClpXP activity, transcription of genes involved in the lytic cycle of the ϕSA3usa prophage are completely repressed, as expected for a stably integrated prophages. The genome of the USA300 clonal lineage harbours one additional pro-phage, the ϕSA2usa encoding the Panton-Valentine leukocidin (LukS-PV)^35^. However, transcription of genes, with a predicted role in the lytic cycle of this phage, was not significantly affected by *clpP* inactivation.The 10-fold induction of lytic phage genes in the *clpP* deletion mutant is consistent with previous observations of elevated spontaneous release of prophages in *S. aureus clpP* mutants^17^. These prophages were not identified, but based on the presented transcriptomic analysis, we predict that they similarly to the ϕSA3usa prophage belong to the widely conserved Sa3 integrase family^36^. The induction of ϕ3 prophages is linked to the DNA damage-induced SOS response, and in agreement with this notion, transcription of *recA* and *lexA* was significantly enhanced in the *clpP* mutant (Supplemental Table 2). Taken together, our results indicate that induction of prophages in cells lacking ClpP activity is caused indirectly by DNA damage and that the ClpXP protease does not contribute to this process. In conclusion, the most highly up-regulated in the *clpP* mutant compared to the *clpX*_*I265E*_ mutant are reported to respond to cellular stresses such as protein and DNA-damage. In cells lacking only ClpXP activity, these genes are expressed at similar level as in wild-type cells, emphasizing that ClpXP is not required to combat protein folding stress in *S. aureus*.

The genes with the most significant changes in expression in the *clpX*_*I265E*_ mutant compared to the wild-type belong to various functional groups. Highly upregulated loci include the urease operon, the pyrimidine biosynthesis operon, the *betA-betB* operon, and genes belonging to the staphylococcal pathogenicity island (SaPI5). The functional diversity of these operons suggests that they belong to different regulons and, therefore that ClpXP-dependent proteolysis likely control transcription of these operons by different mechanisms. We find it intriguing that SaPI5 genes with a predicted function in SaPI excision and replication, such as SAUSA300_0805 predicted to encode the SaPi excisionase^37^ are highly upregulated in the *clpX*_*I265E*_ mutant - and even more so in the *clpP* mutant (up to 30 fold relative to wild-type cells). In the JE2 wild type cells, transcription of these genes is very low, consistent with the notion that activation of excision and mobilization of SaPIs normally require the presence of specific helper phages providing anti-repressor activity that antagonizes the transcriptional repressor blocking transcription of SaPI-genes required for excision and replication^37^. Interestingly, the presented data support that ClpXP and ClpCP activity impact the regulatory switch decisive for integration/excision of staphylococcal pathogenicity islands and thereby provides novel insight into factors controlling these interesting mobile genetic elements.

Finally, the transcriptional analysis emphasizes the importance of both ClpXP and ClpCP in controlling transcription of central virulence loci in *S. aurues*. The JE2 wild-type strain used here is a derivative of the community-acquired MRSA strain of the USA300 type that is characterized by high virulence accompanied by high expression of toxins and the *agr* virulence regulator^38^. Strikingly, the *agr* quorum sensing locus encompasses the most down-regulated genes in the JE *clpP* mutant. Inactivation, of only ClpXP decreases transcription of *agr* approximately 3 fold, while a complete inactivation of ClpP proteolytic activity reduces expression almost 20 fold, demonstrating that both ClpXP and ClpCP impact *agr* transcription. RNAIII is an extremely pleiotropic regulator that by anti-sense mechanism controls translation of mRNAs like the *rot* transcript encoding Rot, Repressor of toxins^39^. Notably, despite the differences in RNAIII levels, transcription of the RNAIII-controlled virulence genes such as *nuc*, *tig*, *hla*, and *sak* is reduced to almost the same extent by the *clpX*_*I265E*_ and the *clpP* deletion, supporting that reduction of RNAIII below a threshold level will prevent RNAIII from inhibiting translation of the *rot* mRNA^40^. Additionally, RNAIII controls expression of the virulence regulator MgrA by stabilizing the *mgrA* transcript^41^. Consistent, with the severe reduction in RNAIII levels, the level of *mgrA* transcript is reduced at least 5 fold by inactivation of *clpP*, and 4 fold by inactivation of ClpXP alone. We tentatively, conclude that both ClpXP and ClpCP contribute to virulence regulation in *S. aureus* by controlling expression of the Agr quorum sensing locus. However, virulence regulation in *S. aureus* is exceedingly complex, and the both ClpXP and ClpCP conceivably impact expression of virulence genes via a number of pathways.

In conclusion, our results support that ClpXP and ClpCP contributes to different tasks in *S. aureus*, and that ClpC is superior to ClpX in targeting stress-damaged proteins for degradation by ClpP. Hence, bacteria seem to benefit from the use of multiple ClpP specificity factors because Clp ATPases recognize different groups of substrates thereby expanding the repertoire of substrates degraded by ClpP.

## Methods

### Bacterial strains and growth conditions

The bacterial strains used in this study are listed in Table 5. The *S. aureus* strains were grown in tryptic soya broth media (TSB; Oxoid) under vigorous agitation at 200 rpm at 37 °C. In most experiments, 20 ml of medium was inoculated in 200-ml flasks to allow efficient aeration of the medium. For solid medium, 1.5% agar was added to make TSA plates. Erythromycin (7,5 µg ml^−1^) was added as required. Upon receipt of the low-passage isolate SA564, the strain was cultured once and stored frozen at −80 °C. In all of the experiments, we used SA564 and the other strains freshly streaked from the frozen stocks on TSA plates with antibiotics added as required and incubated overnight at 37 °C. The plates were used to inoculate the TSB cultures by transferring a small streak of the colonies into the liquid medium. The growth was followed by measuring the optical densities at 600 nm. The starting OD was always below 0.05.

**Table 5:** Bacterial strains used in the present study.

Strain	Description	References
8325-4	Widely used Staphylococcus aureus wild-type strain cured of all prophages	55
8325-4ΔclpX	ClpX inactivated by introduction of A 651 bp in-frame deletion in 8325-4	16
8325-4ΔclpP	ClpP inactivated by deletion of the entire clpP gene in 8325-4	16
8325-4clpX_I265E_	8325-4 expressing a ClpX_I265E_ variant from the native *clpX* locus.	This study
8325-4clpX_I265E_, sle1^−^	The *sle*1-gene was inactivated by trasnsposon insertion using NEB1688^21^ as donor and 8325-4clpX_I265E_ as recipient. Erythromycin resistant	This study
JE2	CA-MRSA strain USA300 LAC cured of plasmids	21 NARSA (http://www.narsa.net)
JE2ΔclpX	ClpX inactivated by introduction of A 651 bp in-frame deletion in JE2. Erythromycin resistant.	18
JE2 ΔclpX + clpX	JE2Δ*clpX* complemented with a wild-type copy of the *clpX* gene inserted into the SaPI integration site	This study
JE2clpP::ΦNΣ	*clpP* inactivated by transposon insertion in JE2. Erythromycin resistant.	18
JE2clpX_I265E_	JE2 expressing a ClpX_I265E_ variant from the native clpX locus. Erythromycin resistant.	This study

### Strain constructions

#### Site directed mutagenesis to construct S. aureus strains expressing the ClpX_I265E_-variant from the native clpX locus

An internal fragment of *clpX* encompassing the sequence encoding the IGF tripeptide was amplified using the primers, ClpX_Imay_kpnI_F2 AGAGAGGGTACCagcgtattcaacaattaggacca and ClpX_pBASE6_BglII_R2: AGAGAGAGATCTcagtgcaacttcagacaattct (KpnI and BglII), respectively, are underlined. The primers were constructed so that isoleucine encoding ATT-codon targeted for substitution is in the middle of the amplified sequence. The amplified fragment was cloned into a newly developed temperature-sensitive shuttle vector, pBASE6^42^, using the restriction enzymes *kpn*I and *Bgl*II. The correct sequence of the inserted fragment was verified after sequencing a PCR product amplified using the pBASE6 cloning control primers, pBASE_F: CAATCCGTTCTGCAGGCATG and pBASE_R: ACTCATCGCAGTGCAGC. Hereafter, the Q5® Site-Directed Mutagenesis Kit, New England Biolabs was used as described in the manufactures protocol (https://www.neb.com/products/e0554-q5-site-directed-mutagenesis-kit). In order to introduce an I_265_E substitution in the IGF peptide of ClpX, primers (Q5SDM_clpX_F: TGAAAAAGTTgaaGGTTTCTCAAGCAATGAAG + Q5SDM_clpX_RCCAAGACGGCGCTTAATC) were designed in order to substitute the ATT codon with GAA by site-directed mutagenesis. Introduction of the desired codon substitution was verified by sequencing of a PCR-fragment amplified with pBASE6 control primers. The obtained plasmid was introduced into *S. aureus* strain 8325-4 and SA564 as described by Monk *et al*.^43^ and allelic replacement of *clpX* gene was performed as described using 37 °C and 44 °C as the temperatures for permissive and non-permissive plasmid replication, respectively, and omitting counter-selection in selection for plasmid-loss. Correct replacement of chromosomal *clpX* with mutagenized *clpX* encoding the ClpX_I265E_ variant was confirmed by sequencing chromosomal *clpX* after amplification with the primers: clpXfull_seq_F: acgcaaagttcgttgaagga and clpXfull_seq_R: cagtgcaacttcagacaattct. The mutagenized *clpX* gene was subsequently transduced into the MRSA strains USA300 JE2 and COL by transduction with bacteriophage Φ11, by first inserting an *ermB* marker 8 kb downstream of *clpX* between the convergently transcribed genes with locus tags SAOUHSC_1768 and SAOUHSC_1769 as described in ref.^18^. Correct insertion of the mutagenized *clpX* gene into the chromosomal *clpX* locus of JE2 and COL was verified by sequencing as described above.

#### clpX complementation

Complementation of *clpX* was achieved by integrating the *clpX* allele into the chromosome of the *clpX* deletion mutant using plasmid pAQ21. For construction of pAQ21, *clpX* and its native promoter were amplified using gene-specific primers (pAQ15&16_fwd: cggccgctgcatgcctgcagTCTTCATTAAATATTAAATTACAAAAATGAG and pAQ15&16_rev: agctcggtacccggggatccTTTATATCCTCACTTTTTTATATTCTC) and assembled into pJC1111 SAPI integrative vector using NEBuilder HiFi DNA assembly cloning kit. The resulting plasmid construct, pAQ21 was integrated into the SaPI1 attachment site of *S. aureus* RN4220 containing site-specific SaPI integrase (RN9011) as previously described^44^. The chromosomal integration was thereafter moved into the *clpX* null mutant using ϕ11 mediated phage-transduction.

### Super Resolution Microscopy to determine cell size

Cell preparation: *S. aureus* strains were grown in trypic soy broth (TSB) at 37 °C with aeration until exponential phase (OD_600_ of ~0.5). 1 ml cell culture was collected and Nile Red was added to a final concentration of 5 mg/ml and incubated for 5 min at 37 °C with agitation (650 rpm.). Images were acquired using Super resolution structured illumination microscopy (SR-SIM) with an Elyra PS.1 microscope (Zeiss) using a Plan-Apochromat 63x/1.4 oil DIC M27 objective and a Pco.edge 5.5 camera. Images of cell stained with NileRed were acquired using a 561 nm laser (100 mW) with five grid rotations and a grating period of 34 mm. Laser power was set to 10% with an exposure time of 50 ms. Images was reconstructed using ZEN software (black edition, 2012, version 8.1.0.484) based on a structured illumination algorithm, using synthetic, channel specific optical transfer functions and noise filter settings ranging from 6 to 8. Size measurement was performed on reconstructed images of NileRed stained using the Fiji software (2.0.0-rc-54/1.5 h). The major axis of phase 3 cells (i.e cells with a closed septa) was determined as described in ref.^45^. Briefly, an ellipse was fitted to the border limits of the membrane and the measurements of the minor and major axis were acquired. The shape of the cells was assumed to be that of a prolate spheroid and the volume was estimated by the equation V = 4/3πab^2^, where a and b correspond to the major and minor axes, respectively. Ellipse fitting and measurements were performed using ImageJ.

### Detection and quantification of protein aggregates

Bacterial cells were grown aerobically at 37 °C and in 25 ml TSB in (250 ml flask) for 3 h. At this point, the cultures in each flask were split equally with one half incubated at 37 °C and the other at 45 °C for an additional 3 h. Protein aggregates were isolated from 10 ml of culture. The aggregates were isolated from bacterial cells as described by Maisonneuve *et al*.^46^ with minor modifications. Specifically, the cultures were centrifuged at 4000 × g for 10 min and washed twice with 1X phosphate-buffered saline (PBS). Cells were resuspended in 1.5 ml of cold PBS at pH 7, and then they were disrupted by glass beads 3 times at speed 6 for 30 sec using FastPrep FP120 (MP Biomedicals, Santa Ana, CA). A 1 ml portion of the crude extract was transferred into a 1.7 ml Eppendorf tube and centrifuged at 18,000 × g for 30 min. The pellet was resuspended in buffer A (50 mM Tris, 150 mM NaCl, pH = 8) with 1% Triton X-100 and incubated at 4 °C on a rotating platform for 3 h followed by centrifugation at 18,000 × g for 30 min. The process was repeated in buffer A containing 0.5% Triton X-100. After centrifugation, the pellet was resuspended in buffer A and centrifuged at 18,000 × g for 30 min. The resulting pellet was resolubilized in 200 µl of rehydration buffer consisting of 7 M urea, 2 M thiourea, 4% of (wt/vol) 3-[(3-cholamidopropyl)-dimethylammonio]-1- propanesulfonate (CHAPS), 100 mM dithiothreitol (DDT). The solubilized protein aggregates were loaded on 10% SDS-PAGE and was normalized to the total protein in the crude extract, which was quantified by nanodrop 1000 spectrophotometer (Thermo Fisher Scientific Inc, Wilmington, DE) at 280 nm. The gel was stained with coomassie blue G-250 as described^47^. Protein aggregates were quantified densitometrically from coomassie blue G-250 stained gels using imageJ software.

### Western blot analyses

The protein extractions and Western blotting were performed as described by Jelsbak *et al*.^40^. The membranes were pre-blocked with human IgG to avoid a signal from ProteinA. The rabbit-raised antibodies against staphylococcal Sle1^48^, Spx^49^ and ClpX^40^. The bound antibody was detected using the WesternBreeze Chemiluminescent Anti-Rabbit kit or Anti-mouse kit (Invitrogen). All of the Western blots were repeated at least three times with similar results.

### RNA extraction, library preparation and RNA sequencing

The RNA extraction was performed as described previously^40^. Briefly, cultures inoculated to a starting OD_600_ below 0.02 were grown at 37 °C with vigorous shaking, and when the cultures reached OD_600_ = 0.7 +/− 0.1 (exponential samples) samples were withdrawn for the isolation of RNA. Cells were quickly cooled on an EtOH/dry ice bath and frozen at −80 °C until extraction of RNA. RNA was isolated from three biological replicates grown on different days: cells were lyzed mechanically using the FastPrep machine (MP Biomedicals) and RNA was isolated by the RNeasy mini kit (Quiagen, Valencia, Calif) according to the manufacturer’s instructions. RNA integrity was confirmed using a TapeStation with RNA HS screen tapes (Agilent). rRNA was removed by the Ribo pure kit (Illumina, Little Chesterford, USA). High quality RNA was delivered to DNASense ApS (Denmark) for transcriptomic analysis. To remove ribosomal RNA the Ribo-Zero kit for Bacteria (Illumina, Little Chesterford, USA) was used. Based on TapeStation gels (Agilent), the majority of ribosomal RNA was removed in all 12 samples. Transcriptome libraries were prepared using the stranded TruSeq mRNAseq protocol, which enables strand specific identification of transcripts. Library preparation and subsequent Illumina HiSeq sequencing (1 × 50 bp was successful for all samples. The sequencing generated on average 11 million reads pr. Sample and on average 8 million reads mapped to non-rRNA transcripts.

### Bioinformatic processing and analysis

Raw sequence reads in fastq format were trimmed using Trimmomatic v.0.36 with the settings; LEADING:3TRAILING:3 SLIDINGWINDOW:4:15 MINLEN:50 and removing Illumina adpaters if found^50^. The trimmed transcriptome reads were mapped to features annotated as CDS, rRNA or tRNA in the annotated genome of *E. coli* APEC O2 and the two associated plasmids pAPEC-O2-CoIV andpAPEC-O2-R, using bowtie-2 using default parameters^51^. For each mapping the number of reads mapping to a specific gene was calculated using a simple command line script: grep “ˆ@”-v map.sam | cut -f3 | sort | uniq -c > result.txt. The count tables were imported to R (R Core Team (2015)) processed using the default DESeq. 2 workflow^51^ and visualized using ggplot2. PCA analysis of overall sample similarity was done using DESeq. 2 normalized counts (square root transformed), through the vegan ampvis R packages^52^.

### Analysis of gene expression

The DESeq. 2 workflow was applied to normalize the read counts and identify differential expressed genes^53^. Counts from rRNA genes were removed prior to the analysis as these would have been heavily influenced by the Ribo-Zero rRNA removal step. Functional enrichment analysis with regard to gene ontology (GO) categories was performed using the Cytoscape plugin BINGO^54^. The significantly regulated pathways were selected based on the false discovery rate (FDR) (Benjamini-Hochberg multiple testing correction)^55,56^. Only genes with were regulated with at least 2-fold were included for KEGG and functional enrichment analyses.

### Data accessibility

The complete dataset generated using RNA-sequencing is provided in Supplementary Tables 1 and 2.

## Electronic supplementary material

Supplementary files
