# Supplementary material for: The ClpXP protease is dispensable for degradation of unfolded proteins in Staphylococcus aureus
Source: Sci Rep. 2017 Sep 18;7:11739. doi: 10.1038/s41598-017-12122-y (PMC5603545; doi:10.1038/s41598-017-12122-y)
Supplement: Supplementary file 1 — Supplementary files [file 41598_2017_12122_MOESM1_ESM.pdf]

The ClpXP protease is dispensable for degradation of unfolded proteins in *Staphylococcus aureus*

Steen G. Stahlhut[1], Abdulelah A. Alqarzaee[2], Camilla Jensen[1], Niclas S. Fisker[1], Ana R. Pereira[3], Mariana G. Pinho[3], Vinai Chittezham Thomas[2], and Dorte Frees[1]*

Supplementary Figure 1

(Uncropped versions of the Western Blots shown in Figure 1)

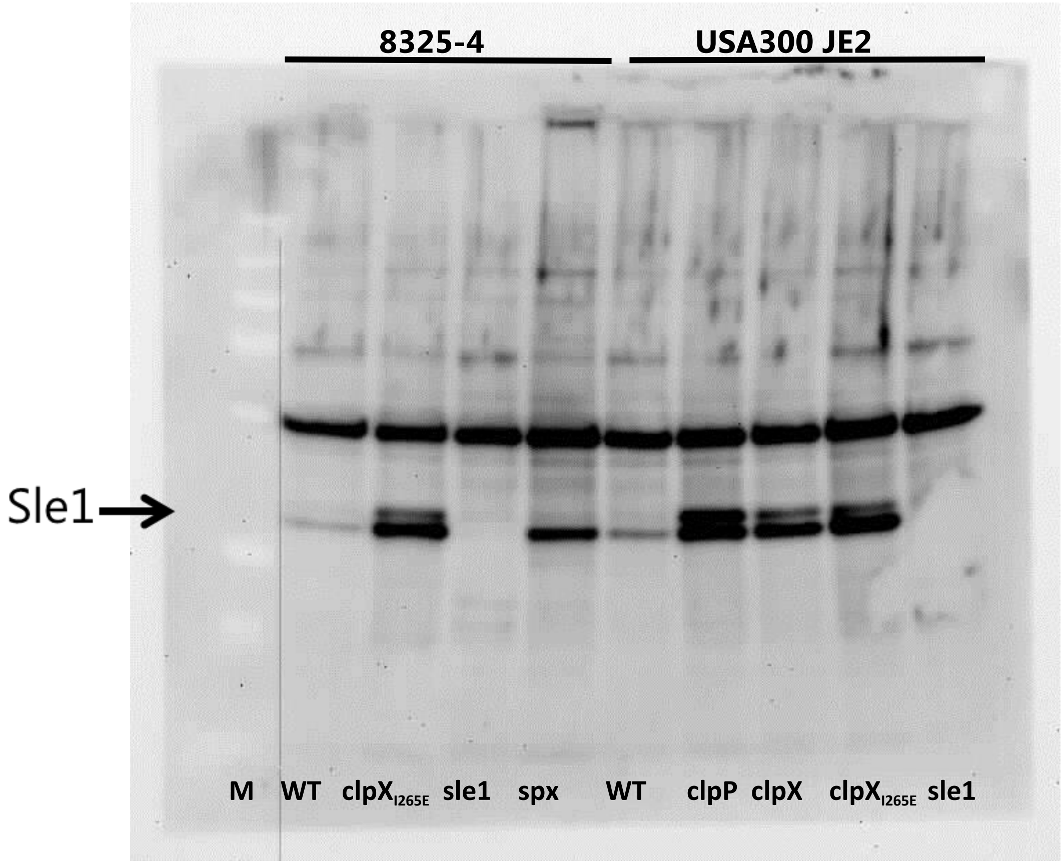
8325-4
USA300 JE2
Sle1
M
WT
clpX I265E
sle1
spx
WT
clpP
clpX
clpX I265E
sle1

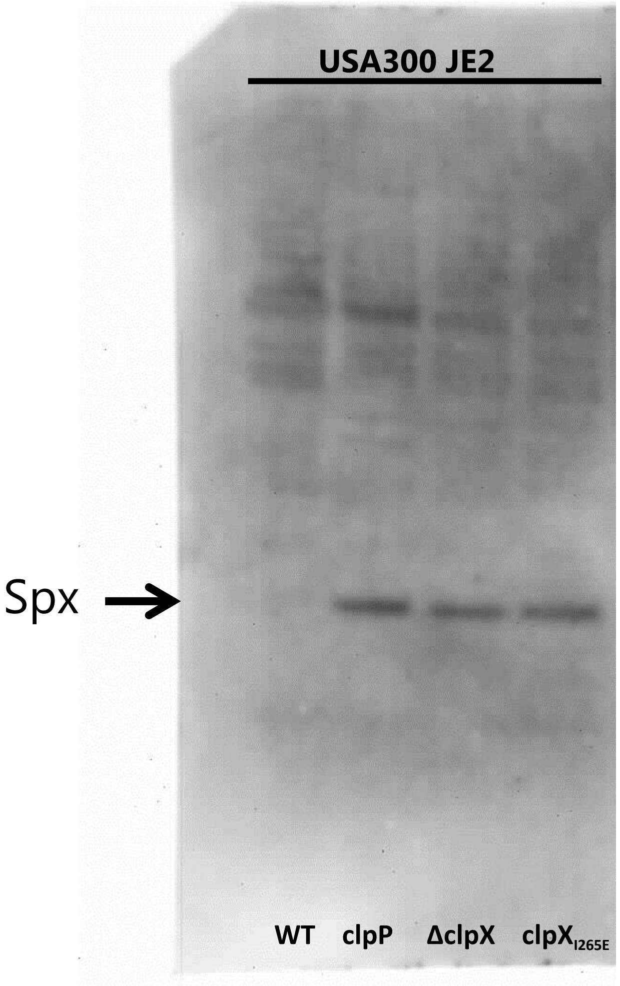
USA300 JE2
Spx
WT
clpP
ΔclpX
clpX$_{I265E}$

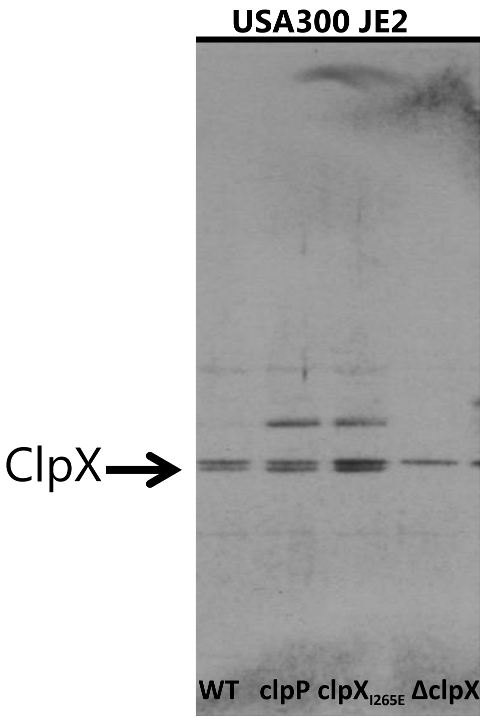
USA300 JE2
ClpX
WT
clpP
clpX$_{I265E}$
ΔclpX

Supplementary Table 1. Transcription in JE2 wild-type compared to JE2 expressing $ClpX_{I265E}$

ID	FoldChange X265/JE2	FoldChange JE2/X265	padj	Significant	JE2 WT E1	JE2 WT E2	JE2 WT E3	ΔclpP E1	ΔclpP E2	ΔclpP E3	ΔclpXP E1	ΔclpXP E2	ΔclpXP E3	Product	Gene
SAUSA300_2238	27,49	0,04	8,31E-102	Yes	47	41	50	1684	1400	1973	1402	1452	1340	urease, gamma subunit	ureA
SAUSA300_2239	21,96	0,05	8,21E-130	Yes	107	107	120	3560	3142	3641	2512	2716	2550	urease, beta subunit	ureB
SAUSA300_2240	18,28	0,05	1,13E-117	Yes	415	448	491	10623	9356	11218	8338	9013	8595	urease, alpha subunit	ureC
SAUSA300_2241	15,16	0,07	4,02E-100	Yes	191	218	219	2906	2466	2205	3201	3357	3433	urease accessory protein UreE	ureE
SAUSA300_2242	13,54	0,07	7,84E-72	Yes	239	212	239	3055	2493	2356	3187	3147	3554	urease accessory protein UreF	ureF
SAUSA300_2237	12,52	0,08	2,50E-45	Yes	102	74	96	791	760	562	1133	1154	1381	putative urea transporter	SAUSA300_2237
SAUSA300_2243	12,07	0,08	8,02E-92	Yes	305	313	355	4196	3760	3558	3873	4194	4146	urease accessory protein UreG	ureG
SAUSA300_2244	11,55	0,09	4,50E-81	Yes	293	256	323	2788	2804	2470	3190	3393	3911	urease accessory protein UreD	ureD
SAUSA300_0808	9,43	0,11	1,27E-17	Yes	19	4	4	282	284	322	84	116	108	conserved hypothetical protein	SAUSA300_0808
SAUSA300_0804	9,04	0,11	7,93E-23	Yes	55	19	32	1355	1562	1943	282	455	348	putative transcriptional regulator	SAUSA300_0804
SAUSA300_2546	7,73	0,13	5,48E-09	Yes	344	331	236	1411	1044	1386	2562	2041	4656	glycine betaine aldehyde dehydrogenase	betB
SAUSA300_0807	7,49	0,13	2,08E-14	Yes	40	16	18	690	751	1202	181	240	234	conserved hypothetical protein	SAUSA300_0807
SAUSA300_0805	7,28	0,14	3,18E-14	Yes	86	29	44	1396	1526	1748	363	583	403	pathogenicity island protein	SAUSA300_0805
SAUSA300_2545	7,16	0,14	2,08E-08	Yes	293	348	223	1272	940	1548	2231	1717	4109	choline dehydrogenase	betA
SAUSA300_1092	6,26	0,16	1,77E-15	Yes	78	86	85	266	237	336	662	403	670	uracil permease	pyrP
SAUSA300_1094	6,12	0,16	4,49E-18	Yes	79	156	113	441	329	583	852	567	911	dihydroorotase	pyrC
SAUSA300_1093	6,00	0,17	2,27E-14	Yes	57	88	64	199	147	204	491	296	612	aspartate carbamoyltransferase	pyrB
SAUSA300_0809	5,97	0,17	1,24E-15	Yes	308	159	176	3194	3320	3784	1158	1640	1397	putative DNA primase	SAUSA300_0809
SAUSA300_0806	5,77	0,17	1,08E-08	Yes	12	4	8	135	171	154	39	78	66	conserved hypothetical protein	SAUSA300_0806
SAUSA300_1095	5,00	0,20	1,39E-18	Yes	121	148	121	378	331	416	797	570	695	carbamoyl-phosphate synthase, small sub	carA
SAUSA300_1096	4,57	0,22	5,71E-20	Yes	377	642	490	1511	1377	1964	2604	2011	2571	carbamoyl-phosphate synthase, large sub	carB
SAUSA300_1097	4,30	0,23	1,20E-17	Yes	67	127	125	337	316	427	498	421	534	orotidine 5'-phosphate decarboxylase	pyrF
SAUSA300_2523	4,24	0,24	3,40E-32	Yes	141	168	159	967	760	1091	650	693	696	conserved hypothetical protein	SAUSA300_2523
SAUSA300_0431	4,16	0,24	0,000925955	Yes	2	4	0	9	9	16	11	18	11	conserved hypothetical protein	SAUSA300_0431
SAUSA300_0810	4,08	0,25	1,01E-11	Yes	81	71	68	703	658	801	259	374	323	conserved hypothetical protein	SAUSA300_0810
SAUSA300_0154	3,77	0,27	8,35E-13	Yes	36	21	22	49	52	35	102	87	116	capsular polysaccharide biosynthesis prote	cap5C
SAUSA300_2524	3,43	0,29	3,72E-17	Yes	72	57	68	285	248	254	223	225	248	conserved hypothetical protein	SAUSA300_2524
SAUSA300_0017	3,39	0,29	5,86E-28	Yes	417	645	445	1524	1569	1894	1889	1627	1680	adenylosuccinate synthetase	purA
SAUSA300_2549	3,37	0,30	7,55E-12	Yes	272	312	307	564	509	676	870	856	1382	choline/carnitine/betaine transporter, BC	bet TbccT
SAUSA300_0811	3,27	0,31	5,76E-05	Yes	33	16	14	109	124	117	62	73	95	conserved hypothetical protein	SAUSA300_0811
SAUSA300_2441	3,24	0,31	1,38E-10	Yes	1416	1274	1409	5065	6557	6617	3916	6074	3767	fibronectin binding protein A	fnbA
SAUSA300_0152	3,20	0,31	3,88E-05	Yes	17	11	8	23	18	19	37	40	49	capsular polysaccharide biosynthesis prote	cap5A
SAUSA300_2522	3,18	0,31	1,96E-08	Yes	45	30	50	160	176	211	127	134	159	conserved hypothetical protein	SAUSA300_2522
SAUSA300_0153	3,18	0,31	3,62E-07	Yes	38	18	14	52	41	52	82	67	78	capsular polysaccharide biosynthesis prote	cap5B
SAUSA300_2154	3,16	0,32	0,002714375	Yes	7	3	11	9	5	12	29	23	24	galactose-6-phosphate isomerase	lacB
SAUSA300_2390	3,15	0,32	2,67E-29	Yes	782	639	652	2363	1814	2322	2488	1824	2282	glycine betaine/carnitine/choline transpor	opuCd
SAUSA300_1641	3,12	0,32	8,52E-24	Yes	212	318	274	1307	1197	1588	861	883	820	citrate synthase II	gltA
SAUSA300_0433	3,10	0,32	9,12E-27	Yes	568	641	581	2114	2076	2532	1668	1882	2062	cysteine synthase/cystathionine beta-synt	cysM
SAUSA300_0079	3,09	0,32	7,29E-42	Yes	1168	1085	973	3891	3805	4058	3531	3078	3434	putative lipoprotein	SAUSA300_0079
SAUSA300_2155	3,09	0,32	0,00762222	Yes	5	7	5	6	16	21	17	24	23	galactose-6-phosphate isomerase	lacA
SAUSA300_0434	3,01	0,33	4,11E-23	Yes	2024	1610	1476	5264	4479	4824	4984	5168	5376	cystathionine gamma-synthase	metB
SAUSA300_2495	3,00	0,33	8,61E-22	Yes	873	602	629	1701	1936	1755	2243	2051	2084	copper chaperone copZ	SAUSA300_2495
SAUSA300_1098	2,90	0,34	1,78E-11	Yes	134	163	121	272	239	315	432	375	440	orotate phosphoribosyltransferase	pyrE
SAUSA300_1440	2,90	0,35	3,90E-13	Yes	62	68	62	113	108	129	211	169	191	conserved hypothetical protein	SAUSA300_1440
SAUSA300_0078	2,86	0,35	1,45E-41	Yes	3892	3871	3137	13147	12881	12645	11078	9741	10504	ATPase copper transport	copA
SAUSA300_2585	2,86	0,35	5,91E-15	Yes	191	244	336	955	888	1022	749	650	859	accessory secretory protein Asp3	SAUSA300_2585
SAUSA300_0231	2,84	0,35	6,88E-10	Yes	486	316	454	821	787	566	1120	1155	1380	ABC transporter, substrate-binding protei	SAUSA300_0231
SAUSA300_2152	2,82	0,35	0,000600967	Yes	16	15	12	30	18	30	47	41	40	tagatose 1,6-diphosphate aldolase	lacD
SAUSA300_1640	2,79	0,36	1,78E-64	Yes	813	854	826	3069	3110	3230	2352	2436	2211	isocitrate dehydrogenase, NADP-depende	icd
SAUSA300_0812	2,77	0,36	3,71E-07	Yes	417	283	291	2499	2720	3036	809	1043	995	conserved hypothetical protein	SAUSA300_0812
SAUSA300_2488	2,77	0,36	0,002469778	Yes	7	5	11	33	25	15	22	21	29	ferrous iron transport protein A	feoA
SAUSA300_2521	2,76	0,36	1,24E-15	Yes	83	71	71	285	248	279	200	192	231	conserved hypothetical protein	SAUSA300_2521
SAUSA300_1981	2,74	0,37	0,003317662	Yes	5	5	8	72	32	43	20	20	20	phage terminase family protein	SAUSA300_1981
SAUSA300_0156	2,73	0,37	5,56E-06	Yes	28	19	22	64	47	74	70	52	74	capsular polysaccharide biosynthesis prote	cap5E
SAUSA300_2325	2,72	0,37	1,34E-07	Yes	34	15	33	37	34	34	69	87	76	conserved hypothetical protein	SAUSA300_2325
SAUSA300_2318	2,72	0,37	1,31E-06	Yes	40	71	90	316	279	526	180	212	186	acetyltransferase, GNAT family	SAUSA300_2318
SAUSA300_2393	2,71	0,37	6,17E-14	Yes	1099	1393	1022	4325	3074	4738	3504	2719	3465	glycine betaine/carnitine/choline ABC tran	opuCa
SAUSA300_2319	2,69	0,37	4,48E-08	Yes	69	136	168	643	532	819	338	342	368	pyridine nucleotide-disulfide oxidoreducta	SAUSA300_2319

SAUSA300_0987	2,69	0,37	0,000403738	Yes	350	182	464	703	721	402	761	1045	1058	cytochrome D ubiquinol oxidase, subunit I	SAUSA300_0987
SAUSA300_2392	2,68	0,37	1,67E-17	Yes	615	678	576	2248	1603	2274	1929	1394	1748	glycine betaine/carnitine/choline ABC trar	opuCb
SAUSA300_0411	2,68	0,37	8,20E-05	Yes	16	7	19	32	34	44	42	35	41	staphylococcal tandem lipoprotein	SAUSA300_0411
SAUSA300_0157	2,66	0,38	6,97E-08	Yes	45	25	33	64	52	83	97	84	96	capsular polysaccharide biosynthesis prote	cap5F
SAUSA300_2487	2,66	0,38	2,72E-15	Yes	96	94	107	424	325	418	256	274	276	ferrous iron transport protein B	feoB
SAUSA300_0100	2,63	0,38	4,12E-07	Yes	29	27	30	49	50	47	76	84	78	staphylococcal tandem lipoprotein	SAUSA300_0100
SAUSA300_0394	2,61	0,38	2,87E-11	Yes	67	64	69	378	356	488	178	174	187	FAD/NAD(P)-binding Rossmann fold Super	SAUSA300_0394
SAUSA300_2249	2,59	0,39	4,16E-15	Yes	9313	10643	7102	30532	38977	35875	22647	23727	24519	secretory antigen precursor SsaA	ssaA
SAUSA300_2586	2,59	0,39	2,96E-24	Yes	591	754	774	2406	2065	2231	1897	1754	1887	accessory secretory protein Asp2	SAUSA300_2586
SAUSA300_0413	2,57	0,39	0,002280678	Yes	7	7	8	27	36	41	18	19	26	staphylococcal tandem lipoprotein	SAUSA300_0413
SAUSA300_0953	2,54	0,39	0,000551881	Yes	90	53	89	196	316	223	212	153	260	putative membrane protein	SAUSA300_0953
SAUSA300_2391	2,54	0,39	9,53E-13	Yes	1421	1432	1112	4795	3656	4870	3910	2698	3589	glycine betaine/carnitine/choline ABC trar	opuCc
SAUSA300_2153	2,54	0,39	0,000386429	Yes	12	15	12	20	25	28	33	35	37	tagatose-6-phosphate kinase	lacC
SAUSA300_0986	2,53	0,40	0,000142389	Yes	1113	691	1192	3023	2536	2163	2334	3111	2484	cytochrome D ubiquinol oxidase, subunit I	SAUSA300_0986
SAUSA300_0300	2,51	0,40	0,000487614	Yes	5	29	40	44	56	64	72	62	70	conserved hypothetical protein	SAUSA300_0300
SAUSA300_2159	2,49	0,40	1,34E-19	Yes	815	522	593	1879	1968	1704	1594	1654	1585	aldo/keto reductase family protein	SAUSA300_2159
SAUSA300_0270	2,48	0,40	1,28E-09	Yes	1219	1332	1175	8050	11233	12126	3118	3497	2805	peptidoglycan hydrolase	lytM
SAUSA300_1380	2,45	0,41	0,023577511	No	12	3	4	13	18	9	15	16	16	conserved hypothetical protein	SAUSA300_1380
SAUSA300_0393	2,40	0,42	0,000511025	Yes	9	12	18	44	50	41	38	28	38	conserved hypothetical protein	SAUSA300_0393
SAUSA300_2466	2,40	0,42	4,72E-07	Yes	78	37	46	73	63	61	118	115	151	putative membrane protein	SAUSA300_2466
SAUSA300_0419	2,39	0,42	4,22E-09	Yes	157	283	249	546	449	584	539	548	600	staphylococcal tandem lipoprotein	SAUSA300_0419
SAUSA300_0813	2,39	0,42	1,05E-05	Yes	88	82	107	371	372	334	209	250	229	conserved hypothetical protein	SAUSA300_0813
SAUSA300_2470	2,35	0,43	6,52E-07	Yes	69	70	88	168	207	234	175	171	199	L-serine dehydratase, iron-sulfur-depende	sdaAB
SAUSA300_1378	2,34	0,43	2,70E-05	Yes	38	22	30	69	70	78	90	62	65	conserved hypothetical protein	SAUSA300_1378
SAUSA300_2588	2,34	0,43	8,30E-12	Yes	257	233	268	401	361	260	537	581	673	preprotein translocase, SecY protein	SAUSA300_2588
SAUSA300_2448	2,30	0,43	2,64E-06	Yes	1328	708	875	1372	1170	948	2488	2049	2301	putative membrane protein	SAUSA300_2448
SAUSA300_2630	2,29	0,44	8,57E-15	Yes	1135	945	817	2220	2024	1794	2416	2166	2104	high-affinity nickel-transporter	nixA
SAUSA300_2084	2,29	0,44	8,86E-21	Yes	360	411	482	980	978	1106	934	1048	918	pantothenate kinase	coaA
SAUSA300_1192	2,27	0,44	5,87E-10	Yes	205	303	308	1000	976	1210	669	684	534	glycerol kinase	glpK
SAUSA300_1456	2,27	0,44	8,53E-06	Yes	186	205	163	328	302	319	317	656	317	alpha glucosidase	SAUSA300_1456
SAUSA300_2494	2,27	0,44	4,96E-21	Yes	2702	2870	2024	8847	8247	8844	6094	5504	5727	copper-translocating P-type ATPase	SAUSA300_2494
SAUSA300_0410	2,27	0,44	0,001531477	Yes	12	12	12	29	23	43	26	36	27	staphylococcal tandem lipoprotein	SAUSA300_0410
SAUSA300_2529	2,26	0,44	9,35E-10	Yes	2127	1261	1612	4281	4064	3737	4249	4049	3139	conserved hypothetical protein	SAUSA300_2529
SAUSA300_0101	2,24	0,45	6,22E-08	Yes	81	74	75	105	142	117	165	165	190	staphylococcal tandem lipoprotein	SAUSA300_0101
SAUSA300_0207	2,22	0,45	7,16E-08	Yes	102	110	98	470	527	510	235	250	214	conserved hypothetical protein	SAUSA300_0207
SAUSA300_0298	2,22	0,45	1,16E-05	Yes	43	40	56	70	61	86	91	92	133	conserved hypothetical protein	SAUSA300_0298
SAUSA300_0085	2,22	0,45	9,58E-06	Yes	29	30	40	105	86	83	75	81	71	conserved hypothetical protein	SAUSA300_0085
SAUSA300_1276	2,21	0,45	1,73E-07	Yes	98	83	89	115	122	83	190	161	255	oligopeptide permease, channel-forming p	opp-2B
SAUSA300_2469	2,21	0,45	2,36E-12	Yes	224	245	273	609	703	812	519	590	554	L-serine dehydratase, iron-sulfur-depende	sdaAA
SAUSA300_0558	2,21	0,45	8,75E-09	Yes	5098	2884	3287	4176	3744	3731	8044	8803	8367	putative proline/betaine transporter	SAUSA300_0558
SAUSA300_1379	2,21	0,45	0,015222227	No	12	5	8	17	29	25	24	15	21	putative lipoprotein	SAUSA300_1379
SAUSA300_0414	2,21	0,45	0,004932131	Yes	7	12	19	39	16	25	30	28	35	staphylococcal tandem lipoprotein	SAUSA300_0414
SAUSA300_2587	2,20	0,46	1,85E-31	Yes	1087	1176	1124	2942	2784	2709	2420	2410	2633	accessory secretory protein Asp1	SAUSA300_2587
SAUSA300_2440	2,19	0,46	1,18E-05	Yes	1671	1148	1895	3470	5333	4244	3158	4483	2875	fibronectin binding protein B	fnbB
SAUSA300_0409	2,18	0,46	3,04E-05	Yes	2398	1199	1043	1635	1875	1667	3638	2864	3846	conserved hypothetical protein	SAUSA300_0409
SAUSA300_0158	2,18	0,46	4,61E-06	Yes	41	66	40	96	86	142	107	106	114	capsular polysaccharide biosynthesis prote	cap5G
SAUSA300_1221	2,17	0,46	0,188639901	No	0	1	1	0	9	3	5	4	4	conserved hypothetical protein	SAUSA300_1221
SAUSA300_0628	2,16	0,46	1,24E-17	Yes	289	412	414	858	886	855	788	887	771	teichoic acid biosynthesis protein D	SAUSA300_0628
SAUSA300_0422	2,14	0,47	7,06E-12	Yes	897	769	641	1204	931	1047	1784	1500	1692	conserved hypothetical protein	SAUSA300_0422
SAUSA300_2151	2,14	0,47	0,077122145	No	5	4	7	7	14	15	12	9	19	PTS system, lactose-specific IIA componen	lacF
SAUSA300_0416	2,14	0,47	0,003403673	Yes	5	18	22	26	27	43	34	32	41	staphylococcal tandem lipoprotein	SAUSA300_0416
SAUSA300_0694	2,13	0,47	0,010257793	No	36	34	33	60	97	81	100	73	59	putative membrane protein	SAUSA300_0694
SAUSA300_1539	2,13	0,47	3,41E-07	Yes	474	657	852	3471	3322	4637	1359	1332	1605	chaperone protein DnaJ	dnaJ
SAUSA300_2492	2,12	0,47	1,21E-11	Yes	267	348	275	889	775	940	664	605	636	acetyltransferase family protein	SAUSA300_2492
SAUSA300_1414	2,11	0,47	0,23430998	No	0	0	0	0	0	3	5	1	0	phiSLT ORF 78B-like protein	SAUSA300_1414
SAUSA300_2493	2,11	0,47	9,01E-05	Yes	57	40	43	77	131	80	102	117	83	conserved hypothetical protein	SAUSA300_2493
SAUSA300_0170	2,11	0,47	3,62E-07	Yes	119	126	130	440	347	396	258	293	254	aldehyde dehydrogenase	SAUSA300_0170
SAUSA300_2317	2,11	0,47	4,90E-16	Yes	1593	1567	1528	5178	5083	5473	3425	3616	2896	putative zinc-binding dehydrogenase	SAUSA300_2317
SAUSA300_2104	2,10	0,48	8,54E-08	Yes	11381	9897	9062	19292	17206	19208	21580	22394	20475	glucosamine--fructose-6-phosphate aminc	glmS

SAUSA300_2560	2,08	0,48	8,73E-11	Yes	288	271	266	508	458	345	574	639	517	conserved hypothetical protein	SAUSA300_2560
SAUSA300_2589	2,08	0,48	4,37E-24	Yes	27981	24308	23675	58582	56215	56054	47860	54899	55728	LPXTG-motif cell wall surface anchor famil	SAUSA300_2589
SAUSA300_0127	2,08	0,48	0,009469177	Yes	34	19	40	20	27	13	70	56	77	conserved hypothetical protein	SAUSA300_0127
SAUSA300_1747	2,07	0,48	9,69E-05	Yes	17	40	62	84	83	120	86	82	95	conserved hypothetical protein	SAUSA300_1747
SAUSA300_2253	2,07	0,48	7,04E-07	Yes	3613	4111	2769	15723	18523	21301	6615	6954	8386	secretory antigen precursor SsaA	ssaA
SAUSA300_2631	2,04	0,49	0,000184291	Yes	172	162	138	485	469	526	378	304	304	putative N-acetyltransferase	SAUSA300_2631
SAUSA300_2465	2,00	0,50	0,215883477	No	2	4	6	9	11	1	12	4	15	ABC transporter, ATP-binding protein	SAUSA300_2465
SAUSA300_2160	2,00	0,50	6,28E-06	Yes	67	51	51	82	126	108	124	103	114	transcriptional regulator, MerR family	SAUSA300_2160
SAUSA300_2051	1,99	0,50	9,23E-06	Yes	150	168	125	636	726	765	276	334	284	conserved hypothetical protein	SAUSA300_2051
SAUSA300_1430	1,99	0,50	0,246980902	No	2	3	0	3	2	4	2	5	5	phiSLT ORF 87-like protein, putative DNA-l	SAUSA300_1430
SAUSA300_2598	1,98	0,50	0,17414201	No	14	8	7	4	7	1	19	16	27	capsular polysaccharide biosynthesis prote	cap1A
SAUSA300_2455	1,98	0,51	9,68E-09	Yes	270	238	208	444	367	421	421	596	410	putative fructose-1,6-bisphosphatase	SAUSA300_2455
SAUSA300_1005	1,98	0,51	1,64E-08	Yes	1288	1307	1343	1568	1377	1476	2253	2308	3297	Mn2+/Fe2+ transporter, NRAMP family	SAUSA300_1005
SAUSA300_0965	1,98	0,51	5,58E-11	Yes	2096	2944	2057	3878	3250	3552	4908	4959	4273	methylenetetrahydrofolate dehydrogenas	folD
SAUSA300_2206	1,97	0,51	0,116095468	No	7	16	14	39	68	59	36	33	19	conserved hypothetical protein	SAUSA300_2206
SAUSA300_0421	1,96	0,51	3,16E-06	Yes	532	550	461	715	584	481	1129	915	1021	conserved hypothetical protein	SAUSA300_0421
SAUSA300_2550	1,96	0,51	2,89E-05	Yes	119	127	145	278	255	229	244	248	288	anaerobic ribonucleotide reductase, small	nrdG
SAUSA300_0102	1,95	0,51	7,52E-07	Yes	84	78	86	123	142	117	156	157	178	staphylococcal tandem lipoprotein	SAUSA300_0102
SAUSA300_1538	1,95	0,51	1,05E-13	Yes	846	986	1185	4303	4573	4915	1915	1993	2019	ribosomal protein L11 methyltransferase	prmA
SAUSA300_0535	1,95	0,51	4,55E-17	Yes	940	1035	1237	1935	1774	2045	2026	2390	1890	putative pyridoxal phosphate-dependent a	SAUSA300_0535
SAUSA300_2090	1,95	0,51	7,41E-09	Yes	127	179	169	418	453	534	311	335	298	deoxyribose-phosphate aldolase	deoC
SAUSA300_2476	1,95	0,51	3,39E-07	Yes	1094	1358	1336	2011	1916	2314	2316	3074	2056	phosphotransferase system, glucose-speci	ptsG
SAUSA300_0420	1,94	0,51	3,09E-07	Yes	911	909	909	1577	1237	1319	1867	1590	1894	conserved hypothetical protein	SAUSA300_0420
SAUSA300_1417	1,94	0,52	0,295277821	No	0	0	1	3	0	4	2	2	2	phiSLT ORF 175-like protein	SAUSA300_1417
SAUSA300_2387	1,93	0,52	2,07E-14	Yes	627	728	641	2505	2635	2814	1313	1336	1220	NAD dependent epimerase/dehydratase f	SAUSA300_2387
SAUSA300_0289	1,93	0,52	2,05E-05	Yes	358	327	568	524	548	566	801	747	904	conserved hypothetical protein	SAUSA300_0289
SAUSA300_0299	1,92	0,52	0,003416756	Yes	10	42	47	62	65	62	65	68	70	conserved hypothetical protein	SAUSA300_0299
SAUSA300_2394	1,92	0,52	1,44E-14	Yes	575	501	604	985	974	884	1084	1140	1016	conserved hypothetical protein	SAUSA300_2394
SAUSA300_0638	1,92	0,52	3,81E-05	Yes	48	67	36	69	90	74	103	111	82	dihydroxyacetone kinase, phosphotransfe	SAUSA300_0638
SAUSA300_1562	1,91	0,52	2,23E-06	Yes	76	90	105	160	126	195	176	190	164	LamB/YcsF family protein	SAUSA300_1562
SAUSA300_1275	1,90	0,53	2,20E-05	Yes	81	64	76	102	83	111	142	120	163	peptide ABC transporter, permease protei	SAUSA300_1275
SAUSA300_2103	1,90	0,53	7,72E-09	Yes	117	144	140	272	304	337	257	261	255	ABC transporter, ATP-binding protein	SAUSA300_2103
SAUSA300_0365	1,90	0,53	0,012019644	No	110	48	53	82	115	43	115	183	113	conserved hypothetical protein	SAUSA300_0365
SAUSA300_0031	1,90	0,53	2,61E-06	Yes	262	216	224	331	347	247	464	433	447	conserved hypothetical protein	SAUSA300_0031
SAUSA300_0224	1,89	0,53	0,000360336	Yes	549	335	338	1459	2074	2047	701	976	673	staphylocoagulase precursor	coa
SAUSA300_0020	1,89	0,53	1,94E-22	Yes	2055	2071	2035	4521	4657	4923	3950	4063	3646	DNA-binding response regulator	SAUSA300_0020
SAUSA300_2053	1,88	0,53	6,04E-09	Yes	231	207	308	371	460	373	493	501	429	conserved hypothetical protein	SAUSA300_2053
SAUSA300_0685	1,88	0,53	1,14E-08	Yes	3297	3852	4462	6672	5858	7236	6849	8719	6441	fructose specific permease	fruA
SAUSA300_2491	1,88	0,53	1,06E-10	Yes	589	678	565	1700	1397	1655	1203	1215	1049	1-pyrroline-5-carboxylate dehydrogenase	SAUSA300_2491
SAUSA300_1099	1,88	0,53	0,000134485	Yes	90	119	121	67	68	67	219	171	245	conserved hypothetical protein	SAUSA300_1099
SAUSA300_2584	1,88	0,53	4,17E-07	Yes	722	972	1238	2021	1995	2264	1842	1691	2022	preprotein translocase, secA protein	SAUSA300_2584
SAUSA300_2262	1,87	0,53	0,012921825	No	667	424	987	463	638	383	1187	1182	1653	putative membrane protein	SAUSA300_2262
SAUSA300_2475	1,87	0,53	1,28E-05	Yes	262	389	236	1150	1161	1201	577	565	539	conserved hypothetical protein	SAUSA300_2475
SAUSA300_2100	1,87	0,53	0,011078839	No	3484	2016	3559	2025	2466	1461	5030	4925	7538	lytic regulatory protein	SAUSA300_2100
SAUSA300_1903	1,87	0,54	0,002662649	Yes	76	75	75	285	358	356	115	172	147	conserved hypothetical protein	SAUSA300_1903
SAUSA300_1595	1,87	0,54	3,79E-10	Yes	1910	2447	2862	4951	5563	6149	4474	4792	4292	queuine tRNA-ribosyltransferase	tgt
SAUSA300_2558	1,86	0,54	6,73E-16	Yes	716	637	662	1061	1046	889	1219	1290	1243	sensor histidine kinase	SAUSA300_2558
SAUSA300_2445	1,85	0,54	3,68E-24	Yes	1070	988	1036	2303	2346	2305	1835	1882	2028	transcriptional regulator, MerR family	SAUSA300_2445
SAUSA300_2446	1,85	0,54	3,57E-09	Yes	537	497	632	1113	1030	856	986	1082	1039	conserved hypothetical protein	SAUSA300_2446
SAUSA300_2530	1,85	0,54	1,52E-09	Yes	165	170	168	222	196	231	315	308	313	transcriptional regulator, TetR family	SAUSA300_2530
SAUSA300_0096	1,85	0,54	6,12E-05	Yes	59	60	72	83	119	101	118	118	124	conserved hypothetical protein	SAUSA300_0096
SAUSA300_0397	1,85	0,54	0,033137756	No	16	16	7	24	34	38	17	35	23	exotoxin	SAUSA300_0397
SAUSA300_0008	1,85	0,54	0,000160795	Yes	88	79	84	113	144	152	116	180	177	histidine ammonia-lyase	hutH
SAUSA300_2089	1,85	0,54	1,48E-13	Yes	513	580	641	1585	1557	1671	1038	1171	1019	pyrimidine nucleoside phosphorylase	pdp
SAUSA300_2083	1,84	0,54	5,91E-15	Yes	343	327	385	735	627	660	658	635	666	acetyltransferase, GNAT family	SAUSA300_2083
SAUSA300_0684	1,84	0,54	5,81E-05	Yes	603	850	1043	1289	1028	1417	1347	1871	1443	fructose 1-phosphate kinase	fruB
SAUSA300_0248	1,84	0,54	6,76E-12	Yes	1932	1517	1499	4018	4781	4053	3280	3000	2852	putative teichoic acid biosynthesis protein	SAUSA300_0248
SAUSA300_0417	1,84	0,54	0,002326302	Yes	21	37	36	50	45	55	65	58	58	staphylococcal tandem lipoprotein	SAUSA300_0417
SAUSA300_2503	1,84	0,54	0,011287431	No	1052	501	490	2072	2148	2066	1686	1075	1090	secretory antigen precursor SsaA	SAUSA300_2503

SAUSA300_0840	1,83	0,55	2,94E-06	Yes	76	90	114	199	194	226	176	189	162	conserved hypothetical protein	SAUSA300_0840
SAUSA300_2404	1,83	0,55	1,92E-08	Yes	388	576	515	1551	1506	1698	943	932	860	conserved hypothetical protein	SAUSA300_2404
SAUSA300_2348	1,83	0,55	3,04E-06	Yes	115	179	146	561	588	602	287	271	261	conserved hypothetical protein	SAUSA300_2348
SAUSA300_0159	1,82	0,55	0,004155568	Yes	36	19	26	34	41	44	52	42	54	capsular polysaccharide biosynthesis prote	cap5H
SAUSA300_2081	1,82	0,55	5,03E-29	Yes	3942	3935	3868	6033	6545	6305	7154	7278	6962	CTP synthase	pyrG
SAUSA300_2647	1,81	0,55	0,000831934	Yes	86	73	83	83	74	67	164	128	150	ribonuclease P protein component	rnpA
SAUSA300_0140	1,81	0,55	2,49E-07	Yes	799	1120	1154	2964	2982	3885	1832	2010	1753	deoxyribose-phosphate aldolase	deoC
SAUSA300_2462	1,80	0,56	3,16E-06	Yes	1653	1465	1084	4249	3205	4401	2752	2443	2422	NAD(P)H-flavin oxidoreductase	frp
SAUSA300_2473	1,79	0,56	1,10E-07	Yes	1628	2261	1721	5999	5267	6569	3566	3263	3285	conserved hypothetical protein	SAUSA300_2473
SAUSA300_0879	1,78	0,56	0,259176063	No	0	1	6	14	7	13	7	6	5	isopropylmalate synthase-related protein	SAUSA300_0879
SAUSA300_0290	1,78	0,56	0,153684624	No	7	10	16	16	20	7	15	20	30	putative lipoprotein	SAUSA300_0290
SAUSA300_0931	1,78	0,56	0,000360336	Yes	718	524	758	496	530	327	1315	1220	1068	conserved hypothetical protein	SAUSA300_0931
SAUSA300_2312	1,78	0,56	5,66E-08	Yes	644	998	909	1862	1893	2308	1464	1719	1395	malate:quinone-oxidoreductase	mqo
SAUSA300_1146	1,77	0,56	4,55E-05	Yes	591	650	917	1448	1499	1844	1119	1358	1389	ATP-dependent protease hslV	hslV
SAUSA300_2450	1,77	0,57	0,00024612	Yes	794	532	760	729	816	525	1224	1314	1188	DedA family protein	SAUSA300_2450
SAUSA300_0068	1,76	0,57	NA	NA	3	1	4	3	2	1	8	4	9	cadmium-exporting ATPase, truncation	SAUSA300_0068
SAUSA300_1124	1,76	0,57	1,35E-36	Yes	3765	4027	4252	7016	7032	7009	7140	7441	6690	3-oxoacyl-(acyl-carrier-protein) reductase	fabG
SAUSA300_2200	1,76	0,57	4,65E-05	Yes	4485	5618	5984	7549	8700	9312	9480	10280	8803	30S ribosomal protein S19	rpsS
SAUSA300_2471	1,76	0,57	0,000126237	Yes	324	285	277	510	651	543	527	489	552	perfringolysin O regulator protein	SAUSA300_2471
SAUSA300_1296	1,76	0,57	7,58E-06	Yes	298	238	242	583	557	574	496	422	454	conserved hypothetical protein	SAUSA300_1296
SAUSA300_0381	1,75	0,57	5,48E-12	Yes	1455	1417	1204	5960	5340	6640	2555	2380	2214	putative NAD(P)H-flavin oxidoreductase	SAUSA300_0381
SAUSA300_1180	1,75	0,57	0,137696366	No	491	293	751	66	128	37	977	714	1174	conserved hypothetical protein	SAUSA300_1180
SAUSA300_2132	1,74	0,57	2,52E-05	Yes	134	126	107	93	83	62	196	232	216	conserved hypothetical protein	SAUSA300_2132
SAUSA300_1658	1,73	0,58	1,12E-06	Yes	1393	1393	1260	2425	2074	2069	2551	2157	2354	conserved hypothetical protein	SAUSA300_1658
SAUSA300_1607	1,73	0,58	0,001856565	Yes	470	326	399	271	440	214	733	701	662	conserved hypothetical protein	SAUSA300_1607
SAUSA300_2452	1,73	0,58	0,20446867	No	9	4	6	6	2	0	11	9	13	transcriptional regulator, MarR family	SAUSA300_2452
SAUSA300_2526	1,73	0,58	1,17E-16	Yes	2496	2096	2293	3752	4183	4033	4015	3888	4036	dihydroorotate dehydrogenase	pyrD
SAUSA300_0355	1,73	0,58	5,65E-23	Yes	1767	1844	1761	3686	3904	4002	3185	3213	2917	acetyl-CoA acetyltransferase	SAUSA300_0355
SAUSA300_1563	1,73	0,58	3,57E-06	Yes	117	172	160	306	257	352	261	277	254	acetyl-CoA carboxylase, biotin carboxylase	accC
SAUSA300_0605	1,73	0,58	0,151051147	No	1831	809	2046	782	1095	736	2962	2274	3378	staphylococcal accessory regulator A	sarA
SAUSA300_0737	1,73	0,58	9,50E-32	Yes	11466	10981	11605	22634	24500	23148	19318	19871	19705	preprotein translocase, SecA subunit	secA
SAUSA300_2625	1,72	0,58	0,027616442	No	17	70	60	105	65	114	92	84	89	transcriptional regulator, PadR family	SAUSA300_2625
SAUSA300_0442	1,72	0,58	1,38E-05	Yes	1180	1014	1162	1538	1686	1285	1893	2023	1887	YibE/F-like protein	SAUSA300_0442
SAUSA300_1446	1,72	0,58	5,48E-09	Yes	148	136	126	228	212	229	232	220	249	conserved hypothetical protein	SAUSA300_1446
SAUSA300_0251	1,72	0,58	1,57E-18	Yes	6660	6398	6168	10269	11425	9358	10968	11236	10837	putative teichoic acid biosynthesis protein	SAUSA300_0251
SAUSA300_2603	1,71	0,58	0,00262478	Yes	257	281	207	235	187	195	411	343	537	triacylglycerol lipase precursor	lip
SAUSA300_2614	1,71	0,58	6,17E-08	Yes	684	701	658	1227	1023	1139	1025	1298	1182	putative lipoprotein	SAUSA300_2614
SAUSA300_0065	1,71	0,58	8,00E-06	Yes	158	224	159	378	331	356	332	282	319	arginine deiminase	arcA
SAUSA300_2573	1,71	0,59	2,46E-05	Yes	2308	2004	1721	3542	2973	3201	3910	3371	3097	immunodominant antigen B	isaB
SAUSA300_0775	1,71	0,59	0,05855928	No	17	10	16	34	23	33	30	28	19	conserved hypothetical protein	SAUSA300_0775
SAUSA300_0252	1,71	0,59	1,11E-29	Yes	9509	9260	9173	16014	17342	16383	15819	15976	15892	glycosyl transferase, group 2 family protei	SAUSA300_0252
SAUSA300_2646	1,70	0,59	7,14E-10	Yes	785	757	726	1570	1724	1764	1313	1265	1297	tRNA modification GTPase	trmE
SAUSA300_0400	1,70	0,59	0,040637237	No	14	15	20	21	29	25	31	28	28	exotoxin	SAUSA300_0400
SAUSA300_0817	1,70	0,59	1,47E-11	Yes	558	605	649	1529	1841	1638	1049	1063	984	putative membrane protein	SAUSA300_0817
SAUSA300_2583	1,70	0,59	3,56E-08	Yes	1061	1076	1371	1968	2110	2348	1956	1978	2065	putative glycosyl transferase	SAUSA300_2583
SAUSA300_2162	1,70	0,59	2,28E-05	Yes	298	420	425	755	669	726	652	649	663	M23/M37 peptidase domain protein	SAUSA300_2162
SAUSA300_1138	1,70	0,59	0,000551881	Yes	722	1367	1443	2284	2294	2966	1842	2106	2128	succinyl-CoA synthetase, beta subunit	sucC
SAUSA300_2148	1,70	0,59	1,03E-05	Yes	238	318	417	838	791	914	562	572	539	conserved hypothetical protein	SAUSA300_2148
SAUSA300_0443	1,70	0,59	4,62E-11	Yes	1354	1278	1307	2327	2542	2291	2224	2277	2192	YibE/F-like protein	SAUSA300_0443
SAUSA300_1147	1,69	0,59	3,62E-10	Yes	3293	4027	4417	6944	7005	7431	5935	7068	6913	heat shock protein HslVU, ATPase subunit	hslU
SAUSA300_1297	1,69	0,59	3,77E-06	Yes	264	385	356	842	935	951	590	611	516	conserved hypothetical protein	SAUSA300_1297
SAUSA300_0103	1,69	0,59	0,001557751	Yes	114	105	85	153	137	132	182	155	180	staphylococcal tandem lipoprotein	SAUSA300_0103
SAUSA300_0741	1,69	0,59	7,26E-06	Yes	1371	1659	1482	4702	4823	5003	2470	2701	2503	excinuclease ABC, B subunit	uvrB
SAUSA300_0637	1,69	0,59	0,000177439	Yes	52	67	63	133	106	123	104	115	95	dihydroxyacetone kinase, DhaL subunit	SAUSA300_0637
SAUSA300_1501	1,68	0,59	0,421803881	No	0	4	1	1	2	3	4	3	5	putative competence protein ComG	SAUSA300_1501
SAUSA300_2484	1,68	0,60	2,67E-23	Yes	5868	5403	4876	11619	11517	12000	9192	9522	8436	hydroxymethylglutaryl-CoA synthase	SAUSA300_2484
SAUSA300_2581	1,68	0,60	1,35E-10	Yes	561	728	666	1392	1163	1344	1086	1045	1173	putative surface anchored protein	SAUSA300_2581
SAUSA300_0021	1,68	0,60	6,79E-15	Yes	5126	5422	5017	10900	11488	11880	8756	9076	8347	sensory box histidine kinase	SAUSA300_0021
SAUSA300_0418	1,67	0,60	0,003616019	Yes	47	88	75	139	119	127	131	108	121	staphylococcal tandem lipoprotein	SAUSA300_0418

SAUSA300_0612	1,67	0,60	0,411192443	No	0	1	1	6	5	1	2	4	2	putative Na+/H+ antiporter, MnhC compo	SAUSA300_0612
SAUSA300_0295	1,67	0,60	0,185714376	No	7	10	11	6	16	10	22	8	19	conserved hypothetical protein	SAUSA300_0295
SAUSA300_0288	1,67	0,60	2,85E-05	Yes	923	976	1103	1717	1564	2412	1719	1572	1752	conserved hypothetical protein	SAUSA300_0288
SAUSA300_2294	1,67	0,60	3,60E-07	Yes	339	318	287	463	471	376	494	534	549	conserved hypothetical protein	SAUSA300_2294
SAUSA300_0799	1,66	0,60	1,16E-05	Yes	326	286	259	811	897	849	493	497	463	integrase	int
SAUSA300_0268	1,66	0,60	0,00010974	Yes	122	131	125	500	609	597	171	240	223	putative drug transporter	SAUSA300_0268
SAUSA300_0090	1,66	0,60	2,84E-05	Yes	86	79	99	159	131	135	145	158	143	conserved hypothetical protein	SAUSA300_0090
SAUSA300_2595	1,65	0,61	0,001829995	Yes	71	55	47	119	95	118	89	100	95	acetyltransferase, GNAT family	SAUSA300_2595
SAUSA300_1457	1,65	0,61	0,033409158	No	122	55	81	142	122	120	122	190	120	maltose operon transcriptional repressor	malR
SAUSA300_1088	1,65	0,61	8,75E-09	Yes	353	370	355	627	489	489	606	588	591	glyoxalase family protein	SAUSA300_1088
SAUSA300_2520	1,65	0,61	1,78E-05	Yes	177	133	120	299	239	254	219	235	249	transporter gate domain protein	SAUSA300_2520
SAUSA300_0489	1,65	0,61	2,78E-16	Yes	23358	24630	25398	54484	58079	61444	39770	41366	39992	putative cell division protein FtsH	SAUSA300_0489
SAUSA300_2295	1,65	0,61	1,94E-10	Yes	389	363	349	716	773	800	576	589	648	conserved hypothetical protein	SAUSA300_2295
SAUSA300_0160	1,65	0,61	0,004917578	Yes	60	36	46	36	29	34	85	67	80	capsular polysaccharide biosynthesis prote	cap5I
SAUSA300_0627	1,65	0,61	5,78E-11	Yes	761	917	968	1283	1233	1162	1392	1538	1453	teichoic acid biosynthesis protein X	tagX
SAUSA300_0287	1,65	0,61	0,005462107	Yes	78	90	82	107	97	142	147	129	140	conserved hypothetical protein	SAUSA300_0287
SAUSA300_1395	1,64	0,61	0,445290421	No	0	0	1	1	0	1	2	3	1	phiSLT ORF116b-like protein	SAUSA300_1395
SAUSA300_2078	1,64	0,61	3,63E-14	Yes	2298	2680	2514	5125	5247	5832	4226	3951	4163	UDP-N-acetylglucosamine 1-carboxyvinyltr	murA
SAUSA300_0408	1,64	0,61	0,005413848	Yes	406	208	188	485	532	393	449	478	396	putative surface protein	SAUSA300_0408
SAUSA300_0046	1,64	0,61	2,28E-05	Yes	2053	1428	1611	2281	2270	1990	3072	2873	2452	conserved hypothetical protein	SAUSA300_0046
SAUSA300_2474	1,64	0,61	0,00013688	Yes	224	309	205	964	832	963	383	432	406	conserved hypothetical protein	SAUSA300_2474
SAUSA300_0686	1,64	0,61	8,00E-13	Yes	859	1062	904	2324	2297	2543	1549	1574	1523	N-acetylglucosamine-6-phosphate deacety	nagA
SAUSA300_2125	1,64	0,61	8,19E-07	Yes	2435	3113	2423	6503	6169	7639	4615	4705	3792	ATP-binding protein, Mrp/Nbp35 family	SAUSA300_2125
SAUSA300_1139	1,64	0,61	1,87E-05	Yes	1464	2113	1979	3527	3417	4753	3001	3168	2976	succinyl-CoA synthetase, alpha subunit	sucD
SAUSA300_2648	1,63	0,61	0,000416248	Yes	9745	5753	7647	4505	5630	4405	13826	12368	11880	50S ribosomal protein L34	rpmH
SAUSA300_1145	1,63	0,61	7,29E-06	Yes	694	750	1105	1256	1240	1289	1278	1456	1461	tyrosine recombinase xerC	xerC
SAUSA300_0883	1,63	0,61	0,000918738	Yes	295	327	235	504	500	543	519	416	475	putative surface protein	SAUSA300_0883
SAUSA300_0141	1,63	0,61	4,72E-07	Yes	2074	2790	2779	6855	7010	8046	4075	4538	3915	phosphopentomutase	deoB
SAUSA300_1326	1,63	0,61	0,00162084	Yes	224	201	184	354	419	393	377	313	312	putative cell wall enzyme EbsB	SAUSA300_1326
SAUSA300_0190	1,63	0,61	1,96E-07	Yes	2079	1838	1470	2972	2545	2758	2983	2799	3021	indole-3-pyruvate decarboxylase	ipdC
SAUSA300_0423	1,63	0,61	0,34160228	No	3	4	5	1	0	7	5	8	11	conserved hypothetical protein	SAUSA300_0423
SAUSA300_1876	1,63	0,61	6,01E-08	Yes	265	250	215	557	467	621	416	397	375	DNA polymerase IV	SAUSA300_1876
SAUSA300_1022	1,63	0,61	0,052439618	No	81	68	104	37	70	28	123	134	165	conserved hypothetical protein	SAUSA300_1022
SAUSA300_2644	1,63	0,61	1,34E-10	Yes	687	768	778	1471	1575	1652	1190	1249	1207	glucose-inhibited division protein B	gidB
SAUSA300_0636	1,62	0,62	0,002762455	Yes	52	75	60	122	90	146	91	117	102	dihydroxyacetone kinase, DhaK subunit	SAUSA300_0636
SAUSA300_0023	1,62	0,62	1,40E-13	Yes	2990	2583	2644	4475	4941	4386	4463	4467	4432	YycI protein	SAUSA300_0023
SAUSA300_0229	1,62	0,62	0,005912431	Yes	55	66	50	87	83	90	93	110	80	putative acyl-CoA transferase FadX	SAUSA300_0229
SAUSA300_1148	1,62	0,62	9,76E-11	Yes	2441	2385	2692	3155	3315	3027	3795	4169	4238	GTP-sensing transcriptional pleiotropic rep	codY
SAUSA300_2599	1,62	0,62	0,031265883	No	496	333	672	338	277	237	850	822	806	intercellular adhesion operon transcriptio	tetR
SAUSA300_2551	1,62	0,62	0,004229478	Yes	524	478	496	1043	1062	1037	796	781	870	anaerobic ribonucleotide reductase, large	nrdD
SAUSA300_2102	1,61	0,62	5,75E-09	Yes	746	791	702	1800	1589	1730	1203	1280	1137	haloacid dehalogenase-like hydrolase	SAUSA300_2102
SAUSA300_2323	1,61	0,62	0,005174561	Yes	954	673	999	872	1017	821	1367	1238	1671	transporter, CorA family	cobI
SAUSA300_0626	1,61	0,62	1,62E-08	Yes	815	856	942	1296	1443	1264	1318	1485	1419	teichoic acid biosynthesis protein B	tagB
SAUSA300_2166	1,61	0,62	0,22039151	No	844	582	666	1292	1179	1088	1022	1533	1002	alpha-acetolactate synthase	alsS
SAUSA300_1129	1,61	0,62	0,040641782	No	96	114	230	99	135	80	221	209	296	conserved hypothetical protein	SAUSA300_1129
SAUSA300_2580	1,61	0,62	1,48E-07	Yes	1070	983	805	2469	2265	2481	1588	1599	1412	isochorismatase family protein	SAUSA300_2580
SAUSA300_0328	1,61	0,62	8,55E-07	Yes	165	214	200	619	604	736	315	304	320	lipoate-protein ligase A family protein	SAUSA300_0328
SAUSA300_1126	1,60	0,62	5,69E-05	Yes	224	334	408	597	600	670	495	514	563	ribonuclease III	rnc
SAUSA300_1785	1,60	0,62	0,001341021	Yes	1144	1083	1443	1804	2112	1877	1969	1850	2118	putative ABC transporter protein EcsB	SAUSA300_1785
SAUSA300_1020	1,60	0,62	6,75E-10	Yes	1531	1708	1329	3211	3365	3534	2469	2595	2275	Glycerophosphoryl diester phosphodieste	SAUSA300_1020
SAUSA300_1188	1,60	0,62	4,80E-12	Yes	1932	1855	1812	2728	2867	2743	3024	2827	3136	DNA mismatch repair protein mutS	mutS
SAUSA300_2540	1,60	0,63	5,86E-06	Yes	13066	9464	9558	29448	27263	27256	16978	18160	16393	fructose-bisphosphate aldolase class-I	SAUSA300_2540
SAUSA300_0250	1,60	0,63	9,60E-12	Yes	3806	3849	3555	7160	7808	7057	5934	6045	5983	alcohol dehydrogenase, zinc-containing	SAUSA300_0250
SAUSA300_2383	1,59	0,63	0,023911806	No	57	45	47	69	65	106	85	80	75	amino acid permease	SAUSA300_2383
SAUSA300_1861	1,59	0,63	3,52E-06	Yes	172	215	225	274	243	256	320	330	334	conserved hypothetical protein	SAUSA300_1861
SAUSA300_2046	1,59	0,63	2,73E-06	Yes	11932	8861	10436	12833	14290	11647	17470	17000	15329	membrane protein oxaA precursor	oxaA
SAUSA300_2205	1,59	0,63	0,000175529	Yes	9444	10562	11219	14046	14819	17080	16926	17985	14880	30S ribosomal protein S10	rpsJ
SAUSA300_0327	1,59	0,63	3,03E-05	Yes	210	244	225	670	717	865	341	359	384	conserved hypothetical protein	SAUSA300_0327
SAUSA300_0399	1,59	0,63	0,252273763	No	3	4	13	13	11	18	9	16	11	exotoxin	SAUSA300_0399

SAUSA300_0189	1,59	0,63	1,54E-06	Yes	539	430	369	646	597	660	764	674	680	isochorismatase	entB
SAUSA300_1786	1,58	0,63	1,65E-11	Yes	1796	1693	1632	3895	3877	3664	2850	2782	2497	ABC transporter, ATP-binding protein EcsA	SAUSA300_1786
SAUSA300_0424	1,58	0,63	0,013413688	No	67	56	55	130	104	102	108	84	92	putative cobalamin synthesis protein	SAUSA300_0424
SAUSA300_0366	1,58	0,63	0,001842773	Yes	4816	6621	7870	9353	9169	11423	10129	11403	9289	ribosomal protein S6	rpsF
SAUSA300_1564	1,58	0,63	0,006132451	Yes	26	40	40	44	41	44	54	61	59	acetyl-CoA carboxylase, biotin carboxyl car	accB
SAUSA300_2515	1,58	0,63	0,011287431	No	55	41	61	100	79	74	103	66	83	transcriptional regulator, TetR family	SAUSA300_2515
SAUSA300_0183	1,58	0,63	0,303572248	No	551	546	1260	822	733	979	826	2333	857	conserved hypothetical protein	SAUSA300_0183
SAUSA300_2643	1,58	0,63	5,57E-06	Yes	1946	2474	1779	4926	5013	5479	3375	3418	3029	putative chromosome partioning protein,	SAUSA300_2643
SAUSA300_0368	1,58	0,63	0,005222296	Yes	1996	2976	3815	4152	4161	5324	4600	5002	4390	ribosomal protein S18	rpsR
SAUSA300_2139	1,57	0,64	0,002982919	Yes	110	100	142	624	642	728	174	164	223	putative transporter	SAUSA300_2139
SAUSA300_0325	1,57	0,64	0,001282672	Yes	93	77	109	269	216	291	145	152	147	glycine cleavage H-protein	SAUSA300_0325
SAUSA300_0129	1,57	0,64	3,68E-05	Yes	555	583	530	1402	1334	1775	838	885	913	Acetoin(diacetyl) reductase	SAUSA300_0129
SAUSA300_0291	1,57	0,64	0,228949694	No	36	22	48	19	25	27	47	46	81	putative membrane protein	SAUSA300_0291
SAUSA300_2222	1,57	0,64	0,000420627	Yes	269	457	417	869	875	858	680	578	557	molybdopterin converting factor, subunit	moaE
SAUSA300_1511	1,57	0,64	0,010711213	No	4020	3783	4231	1783	1909	1668	7393	6020	5670	50S ribosomal protein L33	rpmG
SAUSA300_2519	1,57	0,64	1,99E-07	Yes	183	179	177	378	381	430	292	275	280	putative cobalamin synthesis protein	SAUSA300_2519
SAUSA300_0988	1,57	0,64	0,000860491	Yes	575	723	887	1875	1823	1749	1073	1284	1100	potassium uptake protein	trkA
SAUSA300_0407	1,57	0,64	0,129592376	No	88	22	48	93	110	81	92	92	66	exotoxin	SAUSA300_0407
SAUSA300_1710	1,56	0,64	1,95E-07	Yes	1209	1257	1355	1909	1855	1891	2031	2087	1878	putative lysophospholipase	SAUSA300_1710
SAUSA300_2645	1,56	0,64	3,25E-09	Yes	1412	1729	1580	3098	3424	3314	2453	2510	2439	glucose-inhibited division protein A	gidA
SAUSA300_2535	1,56	0,64	7,51E-10	Yes	1576	1747	1447	3265	3261	3480	2504	2616	2346	2-dehydropantoate 2-reductase	panE
SAUSA300_2163	1,56	0,64	0,001929235	Yes	140	198	272	355	336	359	302	326	343	conserved hypothetical protein	SAUSA300_2163
SAUSA300_1190	1,56	0,64	4,26E-09	Yes	398	375	419	501	530	413	635	593	640	glycerol uptake operon antiterminator reg	glpP
SAUSA300_0968	1,56	0,64	0,057890531	No	143	396	247	233	205	365	483	288	485	phosphoribosylaminoimidazole-succinocar	purC
SAUSA300_2561	1,56	0,64	0,055700448	No	28	49	35	67	92	87	63	72	45	alkaline phosphatase	phoB
SAUSA300_2527	1,56	0,64	4,58E-06	Yes	1125	986	1021	1564	1733	1593	1669	1525	1696	conserved hypothetical protein	SAUSA300_2527
SAUSA300_2260	1,56	0,64	0,00043832	Yes	146	160	237	296	313	331	261	310	290	inositol monophosphatase family protein	SAUSA300_2260
SAUSA300_0022	1,55	0,64	8,26E-11	Yes	5417	5537	4774	9394	9730	8747	8324	8254	7909	YycH protein	SAUSA300_0022
SAUSA300_0161	1,55	0,64	0,035631975	No	43	41	36	43	43	34	76	42	70	capsular polysaccharide biosynthesis prote	cap5J
SAUSA300_2597	1,55	0,64	0,000465946	Yes	117	131	109	205	187	166	173	196	186	capsular polysaccharide biosynthesis prote	cap1B
SAUSA300_0772	1,55	0,64	0,001081723	Yes	15244	12177	11888	19484	20272	20772	19410	21706	20261	clumping factor A	clfA
SAUSA300_0932	1,55	0,64	0,097538728	No	837	491	1161	806	956	661	1256	1037	1656	putative membrane protein	SAUSA300_0932
SAUSA300_0286	1,55	0,64	0,002714375	Yes	148	144	142	102	110	135	247	202	229	conserved hypothetical protein	SAUSA300_0286
SAUSA300_0439	1,55	0,65	0,005971852	Yes	413	389	420	405	435	374	769	639	502	conserved hypothetical protein	SAUSA300_0439
SAUSA300_0548	1,55	0,65	0,005254758	Yes	4521	4169	5683	4216	4693	4031	6138	8220	8064	sdrE protein	sdrE
SAUSA300_0401	1,55	0,65	0,015629997	No	59	48	57	74	106	53	88	77	91	exotoxin	SAUSA300_0401
SAUSA300_1350	1,54	0,65	4,47E-08	Yes	239	231	254	381	363	348	381	373	373	conserved hypothetical protein	SAUSA300_1350
SAUSA300_1013	1,54	0,65	4,13E-06	Yes	2454	2016	2037	3818	3958	3769	3463	3400	3210	cell division protein, FtsW/RodA/SpoVE fa	SAUSA300_1013
SAUSA300_0930	1,54	0,65	0,000307468	Yes	1090	1101	945	2185	1805	1800	1753	1575	1535	lipoate-protein ligase A family protein	SAUSA300_0930
SAUSA300_1274	1,54	0,65	0,005254758	Yes	105	148	137	165	151	172	218	159	231	peptide ABC transporter, ATP-binding prot	SAUSA300_1274
SAUSA300_2512	1,54	0,65	0,00110162	Yes	90	88	98	160	144	154	143	133	151	glyoxalase family protein	SAUSA300_2512
SAUSA300_1868	1,54	0,65	0,005441675	Yes	265	203	263	142	189	177	332	328	471	conserved hypothetical protein	SAUSA300_1868
SAUSA300_2204	1,54	0,65	0,000131127	Yes	12950	14395	15253	17646	18804	20537	22882	23888	19039	50S ribosomal protein L3	rplC
SAUSA300_0450	1,54	0,65	0,000118417	Yes	239	292	335	490	451	513	405	518	420	trehalose operon repressor	treR
SAUSA300_1123	1,53	0,65	1,08E-12	Yes	3999	4223	4920	6267	6313	5826	6455	7088	6669	malonyl CoA-acyl carrier protein transacyla	fabD
SAUSA300_0030	1,53	0,65	1,05E-07	Yes	791	918	832	1635	1530	1483	1380	1282	1250	putative glycerophosphoryl diester phospl	SAUSA300_0030
SAUSA300_2567	1,53	0,65	2,28E-05	Yes	357	360	306	577	615	694	522	540	505	carbamate kinase	arcC
SAUSA300_2437	1,53	0,65	0,490289478	No	5	5	21	11	2	13	11	12	35	staphylococcal accessory regulator T	sarT
SAUSA300_2199	1,53	0,65	0,002869861	Yes	4447	5158	5946	6066	6252	7345	7664	8360	7910	50S ribosomal protein L22	rplV
SAUSA300_2480	1,53	0,65	1,96E-05	Yes	834	564	572	1153	1039	994	1031	979	995	transcriptional regulator, LysR family	SAUSA300_2480
SAUSA300_1731	1,53	0,66	0,001504198	Yes	303	263	198	347	340	348	319	477	372	phosphoenolpyruvate carboxykinase (ATP	pckA
SAUSA300_2596	1,53	0,66	0,000707533	Yes	121	122	113	102	131	136	184	174	185	capsular polysaccharide biosynthesis prote	cap1C
SAUSA300_2213	1,52	0,66	6,15E-08	Yes	4110	4321	4618	8149	8506	8997	6734	6720	6493	AcrB/AcrD/AcrF family protein	SAUSA300_2213
SAUSA300_2381	1,52	0,66	0,000240208	Yes	1123	1012	765	1707	1648	1624	1574	1542	1315	conserved hypothetical protein	SAUSA300_2381
SAUSA300_1561	1,52	0,66	9,66E-05	Yes	162	145	134	212	167	163	223	210	236	putative membrane protein	SAUSA300_1561
SAUSA300_2434	1,52	0,66	0,003682179	Yes	96	116	83	152	178	124	154	143	154	transporter protein	SAUSA300_2434
SAUSA300_2506	1,52	0,66	0,00032929	Yes	48989	43113	35762	81423	96654	105774	68819	61169	65053	immunodominant staphylococcal antigen	isaA
SAUSA300_1237	1,52	0,66	0,000704608	Yes	3576	2542	2798	7816	7192	7197	4570	4817	4210	LexA repressor	lexA
SAUSA300_0742	1,52	0,66	0,000492877	Yes	2950	3222	3122	9151	9394	9724	4553	4967	4654	excinuclease ABC, A subunit	uvrA

SAUSA300_0037	1,52	0,66	2,61E-05	Yes	629	558	478	1578	1826	1801	889	859	781	cassette chromosome recombinase B	ccrB
SAUSA300_0415	1,52	0,66	0,186660518	No	10	15	16	23	20	25	21	21	25	staphylococcal tandem lipoprotein	lpl3
SAUSA300_0703	1,52	0,66	6,36E-05	Yes	20985	16234	18341	17751	18299	14057	27334	27472	29664	sulfatase family protein	SAUSA300_0703
SAUSA300_0730	1,51	0,66	0,002506581	Yes	484	430	548	497	512	464	706	687	836	GGDEF domain protein	SAUSA300_0730
SAUSA300_0640	1,51	0,66	0,000611074	Yes	3932	3237	3974	4133	4406	3962	5490	5815	5648	putative membrane protein	SAUSA300_0640
SAUSA300_1130	1,51	0,66	9,59E-09	Yes	3031	3529	3674	5939	6164	6709	5077	5137	5313	signal recognition particle protein	ffh
SAUSA300_1121	1,51	0,66	3,25E-06	Yes	1488	1363	1835	1840	2031	1701	2415	2337	2357	conserved hypothetical protein	SAUSA300_1121
SAUSA300_1348	1,51	0,66	1,96E-09	Yes	734	892	777	1588	1704	1581	1161	1221	1260	polyA polymerase	SAUSA300_1348
SAUSA300_1214	1,51	0,66	0,027281381	No	93	179	134	249	178	297	200	210	216	conserved hypothetical protein	SAUSA300_1214
SAUSA300_2114	1,51	0,66	0,000836675	Yes	103	155	126	223	207	188	197	217	177	arginase	rocF
SAUSA300_0080	1,51	0,66	0,441786701	No	0	0	0	0	0	0	3	1	0	conserved hypothetical protein	SAUSA300_0080
SAUSA300_0875	1,50	0,66	1,08E-06	Yes	1717	1692	1615	3194	3541	3503	2640	2641	2296	conserved hypothetical protein	SAUSA300_0875
SAUSA300_0967	1,50	0,66	0,086551877	No	577	1762	800	997	742	1282	1519	1400	1893	phosphoribosylaminoimidazole carboxylas	purK
SAUSA300_0395	1,50	0,67	0,113594599	No	67	48	68	92	38	75	96	104	82	exotoxin	SAUSA300_0395
SAUSA300_0707	1,50	0,67	3,53E-07	Yes	2954	2280	2402	3496	3388	2866	3935	3666	3894	osmoprotectant ABC transporter, permeas	SAUSA300_0707
SAUSA300_1122	1,50	0,67	1,78E-10	Yes	3491	3829	4298	6155	6460	6282	5865	6087	5541	fatty acid/phospholipid synthesis protein I	plsX
SAUSA300_1983	1,50	0,67	0,009396826	Yes	553	961	520	3648	3277	4353	1131	1041	910	10 kDa chaperonin	groES
SAUSA300_0024	1,50	0,67	1,39E-05	Yes	1359	1087	1117	2396	2364	2506	1868	1828	1663	metallo-beta-lactamase family protein	SAUSA300_0024
SAUSA300_2353	1,50	0,67	0,001444737	Yes	627	673	525	603	545	526	940	929	883	conserved hypothetical protein	SAUSA300_2353
SAUSA300_2182	1,50	0,67	2,45E-06	Yes	2701	2974	3260	2390	2342	1775	4433	4669	4344	translation initiation factor IF-1	infA
SAUSA300_1258	1,50	0,67	0,053577926	No	610	736	814	402	471	500	1414	932	939	4-oxalocrotonate tautomerase	SAUSA300_1258
SAUSA300_0285	1,50	0,67	0,018974543	No	171	136	169	239	230	313	269	207	243	conserved hypothetical protein	SAUSA300_0285
SAUSA300_2468	1,50	0,67	0,003068853	Yes	145	172	134	232	221	291	262	215	203	acetyltransferase, GNAT family	SAUSA300_2468
SAUSA300_0249	1,50	0,67	2,27E-06	Yes	2069	2003	1943	2950	3167	2527	3060	2940	3023	2-C-methyl-D-erythritol 4-phosphate cytid	SAUSA300_0249
SAUSA300_0886	1,50	0,67	7,39E-06	Yes	7156	9293	9561	12946	13438	15048	13165	13874	11998	3-oxoacyl-(acyl-carrier-protein) synthase II	fabF
SAUSA300_2185	1,49	0,67	0,000498602	Yes	5653	6835	6656	7387	7627	8917	9834	10271	8634	50S ribosomal protein L15	rplO
SAUSA300_2602	1,49	0,67	0,356646713	No	7	14	14	11	18	12	14	16	24	intercellular adhesion protein C	icaC
SAUSA300_0777	1,49	0,67	0,241781812	No	10995	5641	13242	4309	6730	4547	15275	17750	13094	cold shock protein	SAUSA300_0777
SAUSA300_0448	1,49	0,67	0,116593628	No	742	698	870	760	818	872	1006	1611	900	PTS system, trehalose-specific IIBC compo	treP
SAUSA300_0969	1,49	0,67	0,01571727	No	174	318	254	206	153	251	420	282	427	phosphoribosylformylglycinamidine synth	purS
SAUSA300_0064	1,49	0,67	0,001404584	Yes	107	111	112	228	230	240	178	150	167	arginine/oirnithine antiporter	arcD
SAUSA300_0490	1,49	0,67	0,013710118	No	844	1136	1367	3151	3248	3973	1634	1575	1832	33 kDa chaperonin (Heat shock protein 33	SAUSA300_0490
SAUSA300_2443	1,49	0,67	0,05069099	No	127	167	146	176	203	234	180	296	192	gluconate kinase	gntK
SAUSA300_0653	1,49	0,67	0,02988515	No	512	386	532	485	568	430	699	633	817	transcriptional regulator, AraC family	SAUSA300_0653
SAUSA300_0326	1,49	0,67	0,000272238	Yes	127	170	175	481	480	495	231	237	246	conserved hypothetical protein	SAUSA300_0326
SAUSA300_0992	1,49	0,67	0,001612973	Yes	2713	2081	2546	2831	2635	2085	4134	3211	3606	putative lipoprotein	SAUSA300_0992
SAUSA300_1693	1,49	0,67	2,02E-05	Yes	512	537	512	1161	992	1050	797	860	670	conserved hypothetical protein	SAUSA300_1693
SAUSA300_0660	1,48	0,67	0,067384923	No	227	151	316	231	329	207	335	300	409	conserved hypothetical protein	SAUSA300_0660
SAUSA300_2194	1,48	0,68	0,005424484	Yes	5167	6588	6754	7518	8173	8964	9357	9986	8246	50S ribosomal protein L14	rplN
SAUSA300_0624	1,48	0,68	1,48E-07	Yes	2942	2562	2787	4136	3741	4442	4080	3980	4238	teichoic acid translocation ATP-binding pro	tagH
SAUSA300_1298	1,48	0,68	0,042503733	No	406	412	646	526	487	429	678	654	863	putative XpaC protein	SAUSA300_1298
SAUSA300_2289	1,48	0,68	0,003449662	Yes	419	235	282	200	180	191	441	456	485	conserved hypothetical protein	SAUSA300_2289
SAUSA300_0302	1,48	0,68	0,012293754	No	86	57	84	72	79	114	118	105	114	conserved hypothetical protein	SAUSA300_0302
SAUSA300_2231	1,47	0,68	0,000422866	Yes	1786	1422	1176	2911	2662	2616	2482	1970	2028	formate dehydrogenase family accessory p	fdhD
SAUSA300_0074	1,47	0,68	3,12E-05	Yes	389	437	359	614	739	677	628	595	530	oligopeptide permease, channel-forming p	opp-3B
SAUSA300_2236	1,47	0,68	0,000230323	Yes	482	608	520	1310	1168	1633	734	828	823	conserved hypothetical protein	SAUSA300_2236
SAUSA300_1942	1,47	0,68	0,228442598	No	53	19	14	256	194	226	29	60	38	conserved hypothetical phage protein	SAUSA300_1942
SAUSA300_2150	1,47	0,68	0,031734701	No	65	79	67	96	56	83	118	85	111	PTS system, lactose-specific IIBC compone	lacE
SAUSA300_2263	1,47	0,68	0,121080869	No	24	25	21	17	11	13	39	33	32	putative transposase	SAUSA300_2263
SAUSA300_1850	1,47	0,68	1,64E-05	Yes	1080	908	1005	1525	1560	1514	1401	1469	1538	conserved hypothetical protein	SAUSA300_1850
SAUSA300_0826	1,47	0,68	7,58E-07	Yes	866	869	795	1561	1422	1247	1293	1196	1236	conserved hypothetical protein	SAUSA300_0826
SAUSA300_1484	1,47	0,68	0,412317465	No	5	5	8	6	18	7	12	8	10	conserved hypothetical protein	SAUSA300_1484
SAUSA300_0237	1,47	0,68	3,43E-05	Yes	472	511	424	1173	1269	1471	684	674	710	inosine-uridine preferring nucleoside hydr	SAUSA300_0237
SAUSA300_1995	1,47	0,68	0,003972137	Yes	183	186	180	206	144	136	241	305	263	sucrose operon repressor	scrR
SAUSA300_2129	1,47	0,68	0,009251174	Yes	436	423	706	699	818	685	718	779	821	putative hemolysin III	SAUSA300_2129
SAUSA300_0827	1,47	0,68	1,32E-06	Yes	463	468	464	878	847	747	709	691	653	putative membrane protein	SAUSA300_0827
SAUSA300_0301	1,47	0,68	0,117868388	No	21	42	58	33	32	34	65	48	71	conserved hypothetical protein	SAUSA300_0301
SAUSA300_1128	1,47	0,68	0,022446609	No	455	823	999	1382	1519	1770	1128	1078	1166	signal recognition particle-docking protein	ftsY
SAUSA300_0706	1,47	0,68	6,21E-09	Yes	2067	1848	1695	3166	3043	2974	2853	2670	2698	putative osmoprotectant ABC transporter,	SAUSA300_0706

SAUSA300_2361	1,46	0,68	0,256896474	No	62	37	53	14	27	9	88	69	71	conserved hypothetical protein	SAUSA300_2361
SAUSA300_1189	1,46	0,68	1,16E-06	Yes	1779	1785	1794	2175	2511	2112	2615	2485	2764	DNA mismatch repair protein mutL	mutL
SAUSA300_0128	1,46	0,68	6,26E-05	Yes	307	227	243	296	237	282	389	394	349	conserved hypothetical protein	SAUSA300_0128
SAUSA300_2201	1,46	0,68	0,004151473	Yes	16175	18466	19526	23482	25192	28806	26698	28943	23884	50S ribosomal protein L2	rplB
SAUSA300_1091	1,46	0,68	0,052922028	No	1373	1533	1037	1362	1064	1239	2079	1629	2109	PyrR bifunctional protein	pyrR
SAUSA300_2559	1,46	0,69	0,032996429	No	670	502	699	599	620	479	906	860	988	DNA-binding response regulator	SAUSA300_2559
SAUSA300_2203	1,46	0,69	0,00347981	Yes	9156	9581	10775	10016	10512	12770	13904	15306	14020	50S ribosomal protein L4	rplD
SAUSA300_1493	1,46	0,69	0,20108715	No	55	38	48	37	50	43	60	76	74	conserved hypothetical protein	SAUSA300_1493
SAUSA300_2513	1,46	0,69	0,006272731	Yes	62	133	103	200	180	210	153	146	144	conserved hypothetical protein	SAUSA300_2513
SAUSA300_0240	1,46	0,69	0,45016866	No	2	5	5	6	9	7	5	6	8	PTS system, galactitol-specific enzyme II, B	SAUSA300_0240
SAUSA300_1061	1,46	0,69	0,336842106	No	5	10	7	17	16	19	8	9	17	putative exotoxin 3	SAUSA300_1061
SAUSA300_0005	1,45	0,69	2,01E-05	Yes	4685	6373	5656	9410	8901	10307	8371	8217	7764	DNA gyrase, B subunit	gyrB
SAUSA300_1601	1,45	0,69	0,00024548	Yes	8818	9006	9992	10295	10478	11876	13882	14234	12405	50S ribosomal protein L27	rpmA
SAUSA300_1416	1,45	0,69	0,594261447	No	0	1	1	0	2	10	2	2	1	phiSLT ORF 81b-like protein	SAUSA300_1416
SAUSA300_0449	1,45	0,69	0,010756791	No	327	416	503	596	595	676	548	717	560	alpha,alpha-phosphotrehalase	treC
SAUSA300_1655	1,45	0,69	0,000161899	Yes	288	282	266	332	325	364	373	448	391	alanine dehydrogenase	ald
SAUSA300_1659	1,45	0,69	6,38E-06	Yes	5606	5187	4212	8300	7936	8463	7885	7061	6795	thiol peroxidase	tpx
SAUSA300_0453	1,45	0,69	0,024383457	No	1037	1632	1968	3205	3223	4776	2334	2319	2113	conserved hypothetical protein	SAUSA300_0453
SAUSA300_2214	1,45	0,69	4,49E-05	Yes	1896	2480	2420	3778	3509	4166	3245	3340	3279	FmhB protein	SAUSA300_2214
SAUSA300_0780	1,44	0,69	6,73E-05	Yes	248	211	198	422	464	452	319	312	313	conserved hypothetical protein	SAUSA300_0780
SAUSA300_1847	1,44	0,69	7,25E-07	Yes	1121	991	1084	2158	2382	2174	1590	1568	1460	conserved hypothetical protein	SAUSA300_1847
SAUSA300_2304	1,44	0,69	3,71E-05	Yes	598	572	460	988	1003	1019	816	768	762	putative membrane protein	SAUSA300_2304
SAUSA300_0868	1,44	0,69	2,33E-06	Yes	1405	1105	1236	1488	1499	1255	1878	1733	1792	signal peptidase IB	spsB
SAUSA300_0425	1,44	0,69	0,396820336	No	808	987	1619	1024	1032	1213	1311	2638	1215	NADH dehydrogenase I, F subunit	SAUSA300_0425
SAUSA300_0828	1,44	0,69	2,43E-09	Yes	1283	1359	1244	2061	2189	1868	1895	1893	1812	5'-nucleotidase family protein	SAUSA300_0828
SAUSA300_0643	1,44	0,69	5,22E-07	Yes	1738	1495	1408	2538	2389	2397	2279	2323	2078	acetyltransferase, GNAT family	SAUSA300_0643
SAUSA300_1431	1,44	0,69	0,604604425	No	0	0	1	1	2	1	2	1	1	phiSLT ORF71-like protein	SAUSA300_1431
SAUSA300_2300	1,44	0,69	0,054739364	No	396	337	492	314	545	244	592	584	605	transcriptional regulator, TetR family	SAUSA300_2300
SAUSA300_0367	1,44	0,70	0,027597594	No	5977	8748	10889	13188	13149	17023	12394	13540	11191	single-strand binding protein	ssb
SAUSA300_1263	1,44	0,70	0,132790623	No	22	29	29	54	63	62	37	38	42	anthranilate synthase, glutamine amidotra	trpG
SAUSA300_1492	1,43	0,70	0,158325937	No	346	283	481	185	248	127	532	451	638	putative lipoprotein	SAUSA300_1492
SAUSA300_1691	1,43	0,70	2,85E-10	Yes	1331	1185	1084	2164	2087	2044	1775	1742	1640	glutamyl-aminopeptidase	SAUSA300_1691
SAUSA300_1246	1,43	0,70	0,000105177	Yes	5108	5319	4572	8234	8360	7831	8019	7347	6181	aconitate hydratase	acnA
SAUSA300_0081	1,43	0,70	0,009222256	Yes	685	550	630	667	561	497	931	793	961	conserved hypothetical protein	SAUSA300_0081
SAUSA300_0191	1,43	0,70	0,003447791	Yes	2625	3530	3328	3486	2840	3933	4392	4819	4432	PTS system, glucose-specific IIBC compone	ptsG
SAUSA300_0385	1,43	0,70	0,001462669	Yes	639	517	585	494	417	435	842	840	816	conserved hypothetical protein	SAUSA300_0385
SAUSA300_2147	1,43	0,70	7,92E-05	Yes	2065	2192	1667	5200	4756	5755	3029	2864	2595	alcohol dehydrogenase, zinc-containing	SAUSA300_2147
SAUSA300_2184	1,43	0,70	8,18E-05	Yes	18842	22204	22200	23531	23556	22481	30717	32200	27725	preprotein translocase, SecY subunit	SAUSA300_2184
SAUSA300_0281	1,43	0,70	0,151847544	No	91	37	53	17	11	13	85	64	108	conserved hypothetical protein	SAUSA300_0281
SAUSA300_1070	1,43	0,70	0,045166352	No	1001	691	796	733	672	560	1320	1130	1131	acetyltransferase, GNAT family	SAUSA300_1070
SAUSA300_1565	1,43	0,70	0,006303542	Yes	79	110	111	153	124	145	134	161	141	putative urea amidolyase	SAUSA300_1565
SAUSA300_1667	1,43	0,70	0,013263532	No	153	240	218	341	329	300	295	288	300	putative glycerophosphoryl diester phospl	SAUSA300_1667
SAUSA300_0970	1,43	0,70	0,067942011	No	482	957	701	637	496	951	1140	782	1164	phosphoribosylformylglycinamidine synth	purQ
SAUSA300_0296	1,42	0,70	0,14386699	No	29	52	63	33	25	47	61	64	87	conserved hypothetical protein	SAUSA300_0296
SAUSA300_1497	1,42	0,70	2,71E-05	Yes	1380	1745	1618	1703	1799	1726	2093	2428	2248	glycine dehydrogenase, subunit 1 (glycine	SAUSA300_1497
SAUSA300_2192	1,42	0,70	0,005776502	Yes	6913	8360	9151	8522	8770	8626	11512	12525	10861	50S ribosomal protein L5	rplE
SAUSA300_2504	1,42	0,70	4,38E-11	Yes	7005	6583	6659	11225	11770	11246	9192	9890	9696	acyltransferase	SAUSA300_2504
SAUSA300_0903	1,42	0,70	0,001635177	Yes	1750	1395	1591	1855	1961	1550	2132	2230	2385	conserved hypothetical protein	SAUSA300_0903
SAUSA300_2193	1,42	0,70	0,010349142	No	5348	6911	6872	7510	7992	8748	9193	9614	8506	50S ribosomal protein L24	rplX
SAUSA300_1982	1,42	0,70	0,005797085	Yes	4733	6643	4105	27780	24628	32361	7985	7376	6713	60 kDa chaperonin	groL
SAUSA300_0163	1,42	0,70	0,001696281	Yes	181	190	153	239	219	228	265	225	253	capsular polysaccharide biosynthesis prote	cap5L
SAUSA300_0073	1,42	0,71	0,000427892	Yes	646	843	628	1299	1388	1335	1039	1012	962	peptide ABC transporter, peptide-binding	SAUSA300_0073
SAUSA300_2183	1,42	0,71	0,002282582	Yes	9358	11379	10727	14849	15703	17707	15360	15872	13533	adenylate kinase	adk
SAUSA300_1242	1,42	0,71	0,000277593	Yes	303	386	340	672	552	578	484	464	517	exonuclease SbcD	sbcD
SAUSA300_1177	1,42	0,71	1,78E-05	Yes	551	698	615	1021	1059	973	923	826	901	competence/damage-inducible protein cir	cinA
SAUSA300_0014	1,41	0,71	1,16E-06	Yes	10692	10982	9670	15620	16738	16892	14910	15533	13961	DHH subfamily 1 protein	SAUSA300_0014
SAUSA300_2191	1,41	0,71	0,007671444	Yes	3262	4079	3962	4348	4224	4336	5451	5654	4939	30S ribosomal protein S14	rpsN
SAUSA300_1674	1,41	0,71	0,000726846	Yes	3874	4777	3874	8424	7846	8367	5974	5850	5916	putative serine protease HtrA	SAUSA300_1674
SAUSA300_2187	1,41	0,71	0,015769035	No	8872	11309	10511	13928	14213	17290	14849	15787	12936	30S ribosomal protein S5	rpsE

SAUSA300_2165	1,41	0,71	0,225988235	No	458	305	298	503	568	429	478	603	444	alpha-acetolactate decarboxylase	budA
SAUSA300_2562	1,41	0,71	0,57314182	No	40	37	125	29	43	35	93	58	167	conserved hypothetical protein	SAUSA300_2562
SAUSA300_2259	1,41	0,71	0,002595929	Yes	587	735	671	947	976	1158	963	903	955	putative transcriptional regulator	SAUSA300_2259
SAUSA300_2485	1,41	0,71	0,019561301	No	102	157	147	380	352	464	193	190	197	methylated DNA-protein cysteine methylt	SAUSA300_2485
SAUSA300_2210	1,41	0,71	0,000187477	Yes	2416	3011	3067	6066	6018	5973	4123	4398	3477	probable glucose uptake protein	glcU
SAUSA300_2188	1,41	0,71	0,006272731	Yes	5713	7373	7409	7696	7762	8058	9705	10118	9133	50S ribosomal protein L18	rplR
SAUSA300_1351	1,41	0,71	0,000242502	Yes	19769	18778	16775	33396	34379	36849	28065	28102	21832	conserved hypothetical protein	SAUSA300_1351
SAUSA300_0223	1,40	0,71	0,020545701	No	129	100	85	165	185	152	156	147	136	conserved hypothetical protein	SAUSA300_0223
SAUSA300_1339	1,40	0,71	0,134584066	No	727	549	867	460	552	290	950	874	1219	conserved hypothetical protein	SAUSA300_1339
SAUSA300_1734	1,40	0,71	0,044909602	No	62	120	113	206	201	234	138	133	152	conserved hypothetical protein	SAUSA300_1734
SAUSA300_1340	1,40	0,71	6,60E-12	Yes	2854	2762	2738	3185	3403	3058	3864	3971	3876	recombination protein U	recU
SAUSA300_2181	1,40	0,71	0,003722827	Yes	2968	3232	3187	2233	2308	1745	4233	4146	4821	50S ribosomal protein L36	rpmJ
SAUSA300_0426	1,40	0,71	0,260133559	No	1993	2687	2855	3295	3171	4293	2964	4669	3137	conserved hypothetical protein	SAUSA300_0426
SAUSA300_0263	1,40	0,71	0,031652114	No	124	112	102	110	90	84	114	198	162	ribose permease	rbsD
SAUSA300_2582	1,40	0,71	0,000114109	Yes	1438	1155	1493	1376	1535	1313	1852	1812	2064	conserved hypothetical protein	SAUSA300_2582
SAUSA300_0881	1,40	0,71	0,535302606	No	9	5	7	10	5	4	14	5	12	putative membrane protein	SAUSA300_0881
SAUSA300_2190	1,40	0,71	0,002614243	Yes	4258	5151	5722	4508	4481	4390	7196	7379	6662	30S ribosomal protein S8	rpsH
SAUSA300_0945	1,40	0,72	0,000100374	Yes	661	857	824	1077	1174	1125	1198	1002	1090	isochorismate synthase family protein	SAUSA300_0945
SAUSA300_2091	1,40	0,72	0,001735347	Yes	6097	5333	5207	7420	7679	5860	8162	8610	6523	purine nucleoside phosphorylase	deoD
SAUSA300_1583	1,40	0,72	0,003869442	Yes	1512	1372	1423	2194	2001	2147	2172	2146	1713	conserved hypothetical protein	SAUSA300_1583
SAUSA300_0958	1,39	0,72	1,78E-05	Yes	7127	5972	6199	8822	10602	8770	8982	8943	9023	transcriptional regulator	SAUSA300_0958
SAUSA300_0398	1,39	0,72	0,391147349	No	9	7	8	6	14	7	12	12	11	exotoxin	SAUSA300_0398
SAUSA300_0013	1,39	0,72	9,59E-08	Yes	2628	2356	2294	2863	3128	2906	3408	3404	3327	putative membrane protein	SAUSA300_0013
SAUSA300_1071	1,39	0,72	0,000498602	Yes	1102	1287	1231	2412	2236	2299	1754	1577	1724	conserved hypothetical protein	SAUSA300_1071
SAUSA300_0683	1,39	0,72	0,082306659	No	484	467	860	511	460	417	705	981	859	transcriptional regulator, DeoR family	SAUSA300_0683
SAUSA300_0904	1,39	0,72	6,34E-05	Yes	1374	1318	1188	1716	1724	1421	1860	1784	1750	protozoan/cyanobacterial globin family pr	SAUSA300_0904
SAUSA300_0284	1,39	0,72	0,052900109	No	122	82	83	89	101	99	143	105	148	conserved hypothetical protein	SAUSA300_0284
SAUSA300_2322	1,39	0,72	0,007677975	Yes	863	750	704	709	712	531	1130	1025	1065	transcriptional regulator, TetR family	SAUSA300_2322
SAUSA300_0831	1,39	0,72	0,008652334	Yes	465	413	441	248	275	189	628	564	639	conserved hypothetical protein	SAUSA300_0831
SAUSA300_1369	1,38	0,72	0,001152466	Yes	651	687	670	1159	1183	1170	966	871	953	pyridine nucleotide-disulfide oxidoreducta	SAUSA300_1369
SAUSA300_2197	1,38	0,72	0,063649665	No	6343	8510	9169	10712	10961	14125	11688	12237	9576	50S ribosomal protein L16	rplP
SAUSA300_1889	1,38	0,72	0,000129902	Yes	5038	5646	4039	6095	5955	6390	7312	6451	6648	adenylosuccinate lyase	purB
SAUSA300_0324	1,38	0,72	0,009963661	Yes	158	160	170	473	442	594	221	230	230	conserved hypothetical protein	SAUSA300_0324
SAUSA300_0075	1,38	0,72	0,000327913	Yes	417	387	361	649	769	728	560	551	500	oligopeptide permease, channel-forming p	opp-3C
SAUSA300_1023	1,38	0,72	5,33E-05	Yes	508	498	510	637	604	708	687	697	716	conserved hypothetical protein	SAUSA300_1023
SAUSA300_2594	1,38	0,72	0,008661758	Yes	86	105	86	206	207	207	132	121	133	methionine-S-sulfoxide reductase	msrA
SAUSA300_0762	1,38	0,72	0,022978961	No	2153	1862	2161	1600	1968	1816	3137	2668	2761	preprotein translocase, SecG subunit	secG
SAUSA300_1875	1,38	0,72	0,037382113	No	605	461	464	630	694	473	701	644	775	exonuclease	SAUSA300_1875
SAUSA300_0613	1,38	0,72	0,314022898	No	16	14	25	26	56	56	25	30	23	putative Na+/H+ antiporter, MnhD compo	SAUSA300_0613
SAUSA300_0264	1,38	0,72	0,016416281	No	198	189	165	364	293	370	221	319	223	ribose transporter RbsU	SAUSA300_0264
SAUSA300_1044	1,38	0,73	0,01837829	No	7174	6239	6593	5270	7641	5306	10577	8949	8184	thioredoxin	trx
SAUSA300_2542	1,38	0,73	0,001438529	Yes	6049	7370	5802	11296	10551	12186	8882	9313	8361	putative AMP-binding enzyme	SAUSA300_2542
SAUSA300_0654	1,38	0,73	0,551400719	No	14	18	41	21	29	34	35	26	45	staphylococcal accessory protein X	sarX
SAUSA300_0744	1,38	0,73	0,000379374	Yes	582	798	795	1315	1199	1431	968	1053	991	prolipoprotein diacylglyceryl transferase	lgt
SAUSA300_2002	1,38	0,73	0,011924156	No	1052	1131	1224	2114	2267	2470	1619	1505	1582	glycoprotein endopeptidase	SAUSA300_2002
SAUSA300_2221	1,38	0,73	0,064503767	No	67	101	134	195	246	285	146	139	141	molybdopterin converting factor, subunit	moaD
SAUSA300_1660	1,37	0,73	0,022073095	No	150	174	224	223	309	238	224	264	273	putative membrane protein	SAUSA300_1660
SAUSA300_1872	1,37	0,73	1,68E-09	Yes	1211	1232	1278	1574	1612	1384	1692	1751	1680	conserved hypothetical protein	SAUSA300_1872
SAUSA300_2422	1,37	0,73	0,001830021	Yes	512	641	652	1792	1566	2030	852	793	850	oxidoreductase, short-chain dehydrogenas	SAUSA300_2422
SAUSA300_2198	1,37	0,73	0,022841863	No	8860	11097	11807	13032	13963	15159	14536	15763	13508	30S ribosomal protein S3	rpsC
SAUSA300_1467	1,37	0,73	6,75E-09	Yes	3953	4390	3839	4955	5170	5006	5454	5783	5494	2-oxoisovalerate dehydrogenase, E3 comp	lpdA
SAUSA300_0858	1,37	0,73	0,095844711	No	3391	2182	2756	2257	2222	2016	4048	3862	3599	conserved hypothetical protein	SAUSA300_0858
SAUSA300_1522	1,37	0,73	0,000898525	Yes	3817	3358	3576	3607	3410	3327	5170	4664	4933	DNA primase	dnaG
SAUSA300_0363	1,37	0,73	0,061935288	No	117	141	226	107	92	89	222	209	243	conserved hypothetical protein	SAUSA300_0363
SAUSA300_2130	1,37	0,73	0,000722669	Yes	1326	1366	1817	2204	2360	2165	1992	2106	2103	UTP-glucose-1-phosphate uridylyltransfera	SAUSA300_2130
SAUSA300_2180	1,37	0,73	0,00416973	Yes	6316	7867	6821	9362	9268	10073	9937	10458	8460	30S ribosomal protein S13	rpsM
SAUSA300_1009	1,37	0,73	0,004681157	Yes	9531	6987	8244	11518	13411	11101	11528	11959	10524	GTP-binding protein	typA
SAUSA300_1885	1,37	0,73	6,29E-08	Yes	3102	3474	3305	5168	5238	5343	4820	4454	4280	DNA ligase	ligA
SAUSA300_1945	1,37	0,73	0,423985916	No	7	7	11	26	32	44	12	12	11	phi77 ORF071-like protein	SAUSA300_1945

SAUSA300_0077	1,37	0,73	0,01037433	No	229	196	182	299	327	278	276	284	268	ABC transporter, ATP-binding protein	SAUSA300_0077
SAUSA300_2467	1,36	0,73	1,05E-05	Yes	2180	1826	1985	3023	2901	2614	2825	2708	2646	sortase	srtA
SAUSA300_1498	1,36	0,73	0,014562248	No	592	935	855	808	978	868	1011	1203	1056	aminomethyltransferase (glycine cleavage	gcvT
SAUSA300_2215	1,36	0,73	0,117085124	No	102	246	238	349	268	342	241	260	311	conserved hypothetical protein	SAUSA300_2215
SAUSA300_0362	1,36	0,73	0,024062796	No	198	339	317	358	462	470	398	398	382	conserved hypothetical protein	SAUSA300_0362
SAUSA300_0867	1,36	0,73	0,000449765	Yes	1340	1006	1243	1415	1551	1511	1683	1593	1616	signal peptidase IA	spsA
SAUSA300_1633	1,36	0,73	0,01571727	No	219	263	172	208	187	192	281	297	312	glyceraldehyde-3-phosphate dehydrogena	gap
SAUSA300_0444	1,36	0,73	0,037884376	No	152	162	135	199	189	167	175	224	212	LysR family regulatory protein	gltC
SAUSA300_1335	1,36	0,74	0,025276355	No	212	168	221	123	142	123	288	251	281	conserved hypothetical protein	SAUSA300_1335
SAUSA300_1336	1,36	0,74	3,37E-06	Yes	1233	1184	1174	1697	1733	1736	1673	1622	1589	conserved hypothetical protein	SAUSA300_1336
SAUSA300_1428	1,36	0,74	0,6748124	No	0	1	0	1	2	1	1	1	1	conserved hypothetical phage protein	SAUSA300_1428
SAUSA300_2481	1,36	0,74	0,154427367	No	5382	4957	5409	2276	4918	2135	8492	7316	5770	conserved hypothetical protein	SAUSA300_2481
SAUSA300_1299	1,35	0,74	0,00020567	Yes	1967	1953	2099	2499	2615	2470	2498	2709	2962	putative tellurite resistance protein	SAUSA300_1299
SAUSA300_0072	1,35	0,74	0,001918251	Yes	363	405	293	723	708	825	508	481	449	hypothetical protein	SAUSA300_0072
SAUSA300_0016	1,35	0,74	0,001960059	Yes	8619	9223	7457	14566	15453	16547	12010	12211	10102	replicative DNA helicase	dnaB
SAUSA300_0681	1,35	0,74	0,50407437	No	327	189	567	163	295	229	411	328	786	conserved hypothetical protein	SAUSA300_0681
SAUSA300_2386	1,35	0,74	0,003967896	Yes	1058	850	977	929	951	720	1251	1255	1397	beta-lactamase	SAUSA300_2386
SAUSA300_0349	1,35	0,74	0,014613638	No	1786	1340	1304	2788	3038	2396	2325	1959	1709	conserved hypothetical protein	SAUSA300_0349
SAUSA300_0885	1,35	0,74	0,006841692	Yes	2775	3584	4038	3930	4282	4384	4785	4839	4450	3-oxoacyl-(acyl-carrier-protein) synthase II	fabH
SAUSA300_0034	1,35	0,74	0,45968966	No	12	12	13	14	29	6	20	14	18	IS1272, transposase	SAUSA300_0034
SAUSA300_1264	1,35	0,74	0,290947735	No	31	27	35	49	45	31	45	35	47	anthranilate phosphoribosyltransferase	trpD
SAUSA300_0991	1,35	0,74	0,000776749	Yes	2771	2382	2223	4215	4528	4021	3519	3320	3099	peptide deformylase	def
SAUSA300_2382	1,34	0,74	0,008658297	Yes	202	185	168	418	478	479	266	236	242	conserved hypothetical protein	SAUSA300_2382
SAUSA300_2482	1,34	0,74	0,109467905	No	1280	1181	644	1588	2092	1980	1372	1241	1580	conserved hypothetical protein	SAUSA300_2482
SAUSA300_0375	1,34	0,74	0,022645215	No	468	381	406	332	298	197	565	552	571	putative phosphoglycerate mutase family	SAUSA300_0375
SAUSA300_2457	1,34	0,74	0,152813235	No	31	48	49	72	52	84	67	54	57	phospholipase/carboxylesterase family pr	SAUSA300_2457
SAUSA300_2401	1,34	0,75	0,064708386	No	484	444	471	179	140	108	718	578	590	addiction module toxin, Txe/YoeB family	SAUSA300_2401
SAUSA300_1504	1,34	0,75	0,25916784	No	24	36	35	34	38	21	41	38	50	putative competence protein ComGA	SAUSA300_1504
SAUSA300_1425	1,34	0,75	0,565546782	No	0	7	6	9	14	4	6	8	5	conserved hypothetical phage protein	SAUSA300_1425
SAUSA300_1902	1,34	0,75	8,77E-05	Yes	1798	1639	1635	1919	1857	1717	2297	2297	2200	conserved hypothetical protein	SAUSA300_1902
SAUSA300_0233	1,34	0,75	0,45968966	No	127	45	92	43	52	25	104	115	145	conserved hypothetical protein	SAUSA300_0233
SAUSA300_2332	1,34	0,75	0,351948023	No	386	200	348	143	189	86	392	394	485	heat shock protein	SAUSA300_2332
SAUSA300_0335	1,34	0,75	0,005805563	Yes	215	194	218	178	189	135	266	282	291	MATE efflux family protein	SAUSA300_0335
SAUSA300_0071	1,34	0,75	0,001602449	Yes	470	476	460	654	640	578	623	614	643	ISSep1-like transposase	SAUSA300_0071
SAUSA300_1469	1,33	0,75	0,061951738	No	422	381	545	308	343	257	559	600	651	arginine repressor	argR
SAUSA300_2202	1,33	0,75	0,010264744	No	4583	5423	5643	6212	6588	6752	7120	7431	6393	50S ribosomal protein L23	rplW
SAUSA300_1398	1,33	0,75	0,43502917	No	14	11	9	20	23	33	18	13	15	phiSLT ORF123-like protein	SAUSA300_1398
SAUSA300_0763	1,33	0,75	1,05E-05	Yes	1006	1076	1125	1282	1280	1207	1513	1441	1332	carboxylesterase	est
SAUSA300_2628	1,33	0,75	0,000524084	Yes	661	698	643	1441	1382	1349	900	932	836	RarD protein	rarD
SAUSA300_1236	1,33	0,75	0,041899475	No	753	864	782	597	721	609	1091	896	1214	conserved hypothetical protein	SAUSA300_1236
SAUSA300_1620	1,33	0,75	0,000776749	Yes	467	474	415	591	480	473	580	627	594	probable GTP-binding protein engB	SAUSA300_1620
SAUSA300_0957	1,33	0,75	0,14275662	No	2578	1890	2594	1242	1769	875	2900	2955	3596	conserved hypothetical protein	SAUSA300_0957
SAUSA300_1668	1,33	0,75	0,003710092	Yes	667	590	505	1070	985	935	816	764	760	OsmC/Ohr family protein	SAUSA300_1668
SAUSA300_1495	1,33	0,75	0,00753503	Yes	2801	2753	2618	3365	3827	3758	3943	3849	3089	conserved hypothetical protein	SAUSA300_1495
SAUSA300_2005	1,33	0,75	0,016109832	No	432	427	410	294	322	232	606	483	597	conserved hypothetical protein	SAUSA300_2005
SAUSA300_0076	1,33	0,75	0,005518452	Yes	281	289	254	481	516	432	342	374	377	ABC transporter, ATP-binding protein	SAUSA300_0076
SAUSA300_0283	1,33	0,75	0,118806205	No	3224	1993	1551	2130	1756	1964	3479	2475	3067	essC protein	SAUSA300_0283
SAUSA300_1464	1,33	0,75	2,67E-07	Yes	4716	4939	4147	7299	7156	6965	6373	6145	5776	2-oxoisovalerate dehydrogenase, E2 comp	SAUSA300_1464
SAUSA300_1283	1,32	0,75	0,50716081	No	9	10	14	26	38	13	13	10	22	phosphate ABC transporter, phosphate-bi	pstS
SAUSA300_1127	1,32	0,76	0,024658734	No	1690	2322	2470	2505	2303	2492	2816	2675	3126	chromosome segregation protein SMC	smc
SAUSA300_2421	1,32	0,76	0,024147456	No	220	205	250	315	279	251	325	244	330	conserved hypothetical protein	SAUSA300_2421
SAUSA300_0089	1,32	0,76	0,003940341	Yes	1343	1347	1175	1975	2080	2396	1688	1903	1528	Probable tRNA-dihydrouridine synthase	SAUSA300_0089
SAUSA300_2186	1,32	0,76	0,032277775	No	3260	4316	4042	4743	4877	5090	5249	5445	4720	50S ribosomal protein L30	rpmD
SAUSA300_2442	1,32	0,76	0,030138554	No	289	285	301	455	403	492	358	472	331	gluconate permease	gntP
SAUSA300_0839	1,32	0,76	0,022978961	No	2571	2480	2248	3115	3212	3416	3375	3331	2960	conserved hypothetical protein	SAUSA300_0839
SAUSA300_2640	1,32	0,76	0,062552861	No	78	97	91	74	83	70	102	135	118	putative transcriptional regulator	SAUSA300_2640
SAUSA300_1478	1,32	0,76	0,248168817	No	91	88	131	109	110	104	119	133	163	putative lipoprotein	SAUSA300_1478
SAUSA300_2402	1,32	0,76	0,067333289	No	646	597	555	308	248	198	859	703	818	conserved hypothetical protein	SAUSA300_2402
SAUSA300_0006	1,32	0,76	2,97E-05	Yes	9664	10851	9356	14632	14540	16504	13900	13361	12156	DNA gyrase, A subunit	gyrA

SAUSA300_1277	1,32	0,76	0,094472627	No	217	160	201	163	187	192	256	238	269	conserved hypothetical protein	SAUSA300_1277
SAUSA300_0364	1,32	0,76	0,023755251	No	965	1387	1631	1951	2013	2353	1818	1781	1672	GTP-binding protein YchF	ychF
SAUSA300_0279	1,31	0,76	0,135731607	No	3498	1834	1660	1531	1246	1153	3323	2617	3299	putative membrane protein	SAUSA300_0279
SAUSA300_1466	1,31	0,76	1,38E-05	Yes	3612	4007	3232	5844	5926	6220	4979	4828	4463	2-oxoisovalerate dehydrogenase, E1 comp	SAUSA300_1466
SAUSA300_0465	1,31	0,76	0,019472256	No	131	141	112	255	192	243	173	161	170	conserved hypothetical protein	SAUSA300_0465
SAUSA300_0562	1,31	0,76	0,014859971	No	2160	2446	2370	3865	3816	4410	3130	3291	2765	phosphomethylpyrimidine kinase	thiD
SAUSA300_1542	1,31	0,76	0,019780264	No	1035	1196	978	4132	3264	3777	1557	1318	1350	heat-inducible transcription repressor Hrc	hrcA
SAUSA300_1843	1,31	0,76	0,002590361	Yes	1078	1092	984	1339	1271	1111	1359	1394	1392	D-isomer specific 2-hydroxyacid dehydrog	SAUSA300_1843
SAUSA300_0731	1,31	0,76	0,018632413	No	1231	887	1142	1083	1159	957	1406	1411	1469	glycosyl transferase, group 4 family protei	SAUSA300_0731
SAUSA300_1566	1,31	0,76	0,055625706	No	76	82	86	90	97	123	98	112	114	conserved hypothetical protein	SAUSA300_1566
SAUSA300_0659	1,31	0,76	1,96E-05	Yes	1087	1021	1012	1266	1377	1146	1383	1364	1340	sugar efflux transporter	SAUSA300_0659
SAUSA300_1844	1,31	0,76	0,152735205	No	525	490	527	355	397	211	635	633	766	bacterioferritin comigratory protein	SAUSA300_1844
SAUSA300_2003	1,31	0,76	0,016058109	No	536	513	546	643	676	630	697	629	768	ribosomal-protein-alanine acetyltransfera	rimI
SAUSA300_1996	1,31	0,76	0,430265892	No	17	14	12	19	7	12	17	16	23	ammonium transporter	amt
SAUSA300_1854	1,31	0,76	0,119692901	No	257	268	323	242	255	222	360	328	430	regulatory protein RecX	SAUSA300_1854
SAUSA300_1634	1,31	0,77	0,002430925	Yes	1130	1229	1153	1912	1880	2010	1661	1492	1445	dephospho-CoA kinase	coaE
SAUSA300_0572	1,31	0,77	0,043221261	No	773	1038	872	1290	1291	1594	1282	1169	1068	mevalonate kinase	mvk
SAUSA300_0036	1,31	0,77	0,655285572	No	3	7	1	9	16	10	4	7	4	conserved hypothetical protein	SAUSA300_0036
SAUSA300_1261	1,31	0,77	6,16E-06	Yes	744	731	693	1514	1503	1445	967	970	895	putative glutamyl aminopeptidase	SAUSA300_1261
SAUSA300_1051	1,31	0,77	0,079153961	No	2389	1881	2048	1797	1828	1063	2720	2581	2978	conserved hypothetical protein	SAUSA300_1051
SAUSA300_0902	1,30	0,77	0,008689736	Yes	6876	7080	5817	10635	10070	9201	9145	8207	8472	oligoendopeptidase F	pepF
SAUSA300_1465	1,30	0,77	2,27E-05	Yes	3285	3406	2847	4067	4246	3803	4302	4127	4007	2-oxoisovalerate dehydrogenase, E1 comp	SAUSA300_1465
SAUSA300_0917	1,30	0,77	0,035101133	No	2919	2100	2284	2667	2626	1998	3192	3161	3187	putative membrane protein	SAUSA300_0917
SAUSA300_2464	1,30	0,77	0,084386719	No	107	118	85	189	176	169	133	145	125	hydrolase, haloacid dehalogenase-like fam	SAUSA300_2464
SAUSA300_1386	1,30	0,77	0,701105489	No	0	4	2	3	0	0	4	3	3	phiETA ORF59-like protein	SAUSA300_1386
SAUSA300_0526	1,30	0,77	4,76E-05	Yes	1612	1578	1622	1906	2085	1987	2090	2064	2110	Methyltransferase small domain	SAUSA300_0526
SAUSA300_1901	1,30	0,77	0,001676866	Yes	3005	2950	2395	5504	4932	4976	3755	3587	3518	aldehyde dehydrogenase	aldA2
SAUSA300_1682	1,30	0,77	0,020717536	No	2828	3837	2753	4200	3798	3919	4480	4106	3677	catabolite control protein A	ccpA
SAUSA300_0510	1,30	0,77	7,97E-05	Yes	6342	6759	5663	84486	81211	89621	8655	7840	7875	endopeptidase	clpC
SAUSA300_1621	1,30	0,77	7,17E-06	Yes	13040	12534	11464	20807	20479	19845	16508	16618	14959	ATP-dependent Clp protease, ATP-binding	clpX
SAUSA300_2179	1,30	0,77	0,015657944	No	6605	7705	6717	9267	9309	10189	9382	9741	8222	30S ribosomal protein S11	rpsK
SAUSA300_0164	1,30	0,77	0,086359522	No	105	107	82	123	92	124	140	112	128	capsular polysaccharide biosynthesis prot	cap5M
SAUSA300_1053	1,30	0,77	0,341957338	No	52	27	32	29	34	24	52	36	54	conserved hypothetical protein	SAUSA300_1053
SAUSA300_1972	1,30	0,77	0,093965238	No	122	159	92	137	135	135	175	147	163	integrase	int
SAUSA300_1680	1,30	0,77	0,29695144	No	38	57	55	43	50	34	48	87	64	acetoin utilization protein AcuA	acuA
SAUSA300_2444	1,30	0,77	0,362765435	No	45	81	70	80	81	67	69	108	83	gluconate operon transcriptional represso	gntR
SAUSA300_0241	1,30	0,77	0,320001402	No	19	38	35	42	41	50	41	45	38	PTS system, sorbitol-specific IIC componer	SAUSA300_0241
SAUSA300_0947	1,29	0,77	0,00457911	Yes	1219	1058	1316	1678	1661	1532	1499	1595	1566	hydrolase, alpha/beta hydrolase fold famil	SAUSA300_0947
SAUSA300_0238	1,29	0,77	0,270693262	No	29	34	28	42	34	34	44	44	33	transcriptional antiterminator, BglG family	SAUSA300_0238
SAUSA300_0752	1,29	0,77	0,000206773	Yes	8071	6606	6264	2062	2103	2045	9312	9047	8675	ATP-dependent Clp protease, proteolytic s	clpP
SAUSA300_2040	1,29	0,77	0,014191079	No	2813	2343	2526	3224	3577	2926	3504	3475	2951	putative membrane protein	SAUSA300_2040
SAUSA300_1374	1,29	0,78	0,049379624	No	1419	1324	1201	1690	1479	1384	1723	1617	1761	conserved hypothetical protein	SAUSA300_1374
SAUSA300_1994	1,29	0,78	0,022841863	No	1619	2342	2317	3098	2894	3506	2545	2842	2735	sucrose-6-phosphate hydrolase	scrB
SAUSA300_1025	1,29	0,78	0,002259811	Yes	505	391	391	488	485	421	571	548	532	conserved hypothetical protein	SAUSA300_1025
SAUSA300_1282	1,29	0,78	0,408266017	No	17	15	23	59	34	49	27	27	20	phosphate ABC transporter, permease pro	pstC
SAUSA300_1134	1,29	0,78	0,07934249	No	13109	11515	12060	10106	10875	11315	16382	16884	14156	50S ribosomal protein L19	rplS
SAUSA300_0943	1,29	0,78	0,011611715	No	381	413	428	738	753	699	493	573	515	acetyltransferase, GNAT family family	SAUSA300_0943
SAUSA300_1602	1,29	0,78	0,011871959	No	4817	4634	5774	4961	4970	5523	6608	7105	5905	conserved hypothetical protein	SAUSA300_1602
SAUSA300_1606	1,28	0,78	0,182698188	No	196	120	204	73	83	61	222	199	251	conserved hypothetical protein	SAUSA300_1606
SAUSA300_1119	1,28	0,78	4,65E-11	Yes	8911	9022	8483	10976	11220	10555	11610	11454	10878	conserved hypothetical protein	SAUSA300_1119
SAUSA300_1347	1,28	0,78	0,015016549	No	851	865	861	988	983	735	1073	1033	1210	BirA bifunctional protein	birA
SAUSA300_0603	1,28	0,78	0,114455806	No	350	278	354	206	261	191	410	420	434	conserved hypothetical protein	SAUSA300_0603
SAUSA300_2223	1,28	0,78	0,060008961	No	179	267	305	434	403	503	320	294	361	molybdopterin-guanine dinucleotide biosy	mobB
SAUSA300_0704	1,28	0,78	0,016346287	No	2728	2691	3054	3645	3579	3487	3929	3585	3389	ABC transporter, ATP-binding protein	SAUSA300_0704
SAUSA300_1143	1,28	0,78	0,027103437	No	8360	10372	8861	11639	13465	13196	12039	11825	11620	DNA topoisomerase I	topA
SAUSA300_0464	1,28	0,78	0,059399257	No	200	167	201	262	241	327	257	252	221	Methyltransferase	SAUSA300_0464
SAUSA300_1049	1,28	0,78	0,000803697	Yes	2597	2642	2302	5117	5288	5219	3551	3138	2969	glutamate racemase	murI
SAUSA300_0948	1,28	0,78	0,00645443	Yes	4941	5515	5254	10471	9906	12559	6658	7331	6140	naphthoate synthase	menB
SAUSA300_0093	1,28	0,78	0,257332423	No	129	94	126	74	88	68	156	114	179	transcriptional regulator, LysR family doma	SAUSA300_0093

SAUSA300_0508	1,28	0,78	0,021742327	No	804	1043	721	13951	13224	14696	1114	1119	1054	conserved hypothetical protein	SAUSA300_0508
SAUSA300_0657	1,28	0,78	0,010645023	No	701	654	812	669	717	608	937	896	943	conserved hypothetical protein	SAUSA300_0657
SAUSA300_0282	1,28	0,78	0,150252511	No	741	542	419	358	307	340	869	561	744	conserved hypothetical protein	SAUSA300_0282
SAUSA300_1243	1,28	0,78	0,000949149	Yes	782	850	877	1492	1440	1424	1089	981	1138	exonuclease SbcC	sbcC
SAUSA300_0871	1,27	0,78	0,003621069	Yes	8713	8461	8447	9976	10620	8729	10832	11643	10224	conserved hypothetical protein	SAUSA300_0871
SAUSA300_2057	1,27	0,79	0,000474908	Yes	7892	9023	7710	10582	10893	11349	10793	11007	9589	ATP synthase F1, epsilon subunit	atpC
SAUSA300_1107	1,27	0,79	0,217687987	No	1314	936	1335	480	559	441	1556	1454	1581	conserved hypothetical protein	SAUSA300_1107
SAUSA300_1873	1,27	0,79	0,000208849	Yes	1879	1797	1784	2243	2326	2317	2344	2342	2260	Mur ligase family protein	SAUSA300_1873
SAUSA300_1496	1,27	0,79	0,000621753	Yes	2609	2757	2482	2648	2784	2816	3228	3560	3197	glycine dehydrogenase, subunit 2	SAUSA300_1496
SAUSA300_1686	1,27	0,79	0,000186849	Yes	3961	3648	3911	3715	3509	3037	4763	4892	4989	UDP-N-acetylmuramate--alanine ligase	murC
SAUSA300_1118	1,27	0,79	3,31E-06	Yes	1459	1485	1497	1552	1537	1541	1993	1803	1851	conserved hypothetical protein	SAUSA300_1118
SAUSA300_0790	1,27	0,79	0,198130089	No	379	368	664	271	352	226	573	597	636	putative arsenate reductase	SAUSA300_0790
SAUSA300_2516	1,27	0,79	0,0622268	No	133	142	127	278	248	265	181	163	168	oxidoreductase, short chain dehydrogenas	SAUSA300_2516
SAUSA300_0519	1,27	0,79	0,519641713	No	171	122	390	70	137	87	231	261	393	conserved hypothetical protein	SAUSA300_0519
SAUSA300_1540	1,27	0,79	0,015428246	No	10046	12487	9723	48138	42777	54266	14277	13491	13205	chaperone protein DnaK	dnaK
SAUSA300_1541	1,27	0,79	0,026202308	No	2646	2998	2323	7633	6219	6687	3630	3150	3338	co-chaperone GrpE	grpE
SAUSA300_2458	1,27	0,79	0,445290421	No	7	30	23	27	23	40	28	27	26	glyoxylase family protein	SAUSA300_2458
SAUSA300_1733	1,27	0,79	0,053402232	No	214	267	233	528	523	642	304	311	295	conserved hypothetical protein	SAUSA300_1733
SAUSA300_2479	1,27	0,79	0,271784627	No	301	271	222	255	210	186	317	433	262	Holin-like protein cidA	cidA
SAUSA300_1717	1,27	0,79	0,414431967	No	76	57	78	36	38	18	78	87	106	arsenical resistance operon repressor	arsR
SAUSA300_0294	1,27	0,79	0,538065387	No	9	14	12	13	7	25	16	14	16	conserved hypothetical protein	SAUSA300_0294
SAUSA300_0166	1,27	0,79	0,044445305	No	369	376	309	431	424	454	494	412	429	capsular polysaccharide biosynthesis prot	cap5O
SAUSA300_0726	1,27	0,79	0,000434449	Yes	1264	1488	1265	2172	2216	2350	1684	1652	1753	glycerate kinase family protein	SAUSA300_0726
SAUSA300_0234	1,27	0,79	0,281394916	No	3212	1756	2003	3772	2716	2641	2759	3327	2794	putative flavohemoprotein	SAUSA300_0234
SAUSA300_0895	1,26	0,79	0,549099366	No	9	10	11	14	9	15	11	14	12	oligopeptide ABC transporter, permease p	oppB
SAUSA300_0743	1,26	0,79	0,001452096	Yes	1459	1614	1766	2973	2993	3306	1952	2043	2140	HPr(Ser) kinase/phosphatase	hprK
SAUSA300_0293	1,26	0,79	0,50716081	No	28	12	22	14	29	38	26	29	24	conserved hypothetical protein	SAUSA300_0293
SAUSA300_1233	1,26	0,79	0,055982181	No	2780	1934	2358	1912	1618	1603	3356	2644	2953	50S ribosomal protein L33	rpmG
SAUSA300_0527	1,26	0,79	0,003627153	Yes	54156	59298	49264	79899	80773	83059	71693	68256	65830	DNA-directed RNA polymerase, beta subur	rpoB
SAUSA300_1950	1,26	0,79	0,73369974	No	2	1	1	4	0	7	2	2	3	conserved hypothetical phage protein	SAUSA300_1950
SAUSA300_1027	1,26	0,79	0,013065168	No	6250	5344	5579	5796	5898	5250	7416	7231	7059	50S ribosomal protein L32	rpmF
SAUSA300_0512	1,26	0,79	0,082220096	No	797	765	953	654	739	583	1037	930	1217	PIN domain protein	SAUSA300_0512
SAUSA300_1946	1,26	0,79	0,643707758	No	5	5	4	26	23	27	8	6	5	phiPVL ORF057-like protein, transcriptiona	SAUSA300_1946
SAUSA300_0533	1,26	0,79	0,016671239	No	153518	161072	133150	197108	199577	227855	185546	209493	170522	translation elongation factor Tu	tuf
SAUSA300_0057	1,26	0,79	0,578058495	No	16	11	8	24	9	16	17	13	14	conserved hypothetical protein	SAUSA300_0057
SAUSA300_0663	1,26	0,79	0,129592376	No	1411	899	1101	1050	1055	849	1534	1437	1340	putative lipoprotein	SAUSA300_0663
SAUSA300_2291	1,26	0,79	0,056309221	No	413	428	534	451	566	575	565	598	579	sodium/glutamate symporter	gltS
SAUSA300_2377	1,26	0,79	0,031469714	No	634	817	718	1273	1208	1482	948	980	815	glycerate kinase	SAUSA300_2377
SAUSA300_0801	1,26	0,79	0,118806205	No	625	528	524	444	550	393	656	687	772	staphylococcal enterotoxin Q	seq
SAUSA300_2371	1,26	0,80	0,314022898	No	47	41	39	72	52	78	54	46	58	biotin synthase	bioB
SAUSA300_2063	1,26	0,80	0,008665395	Yes	3443	4038	3883	4457	4758	5659	4604	4857	4836	ATP synthase F0, C subunit	atpE
SAUSA300_1848	1,26	0,80	0,012151018	No	815	793	713	1064	1147	1258	1027	948	939	conserved hypothetical protein	SAUSA300_1848
SAUSA300_0262	1,26	0,80	0,007659831	Yes	374	365	355	553	491	584	434	503	439	ribokinase	rbsK
SAUSA300_2220	1,25	0,80	0,057890531	No	310	298	420	398	464	423	457	428	414	molybdopterin-guanine dinucleotide biosy	mobA
SAUSA300_2299	1,25	0,80	0,198632528	No	1188	790	975	925	942	770	1352	1275	1089	multidrug resistance protein A, drug resist	SAUSA300_2299
SAUSA300_2196	1,25	0,80	0,152167433	No	2999	3705	4196	4374	4429	5118	4618	4866	4235	50S ribosomal protein L29	rpmC
SAUSA300_2433	1,25	0,80	0,01184238	No	1306	1406	1147	1939	1661	1652	1627	1592	1621	phosphoglucomutase/phosphomannomut	SAUSA300_2433
SAUSA300_0266	1,25	0,80	0,712314939	No	28	5	23	9	7	18	25	17	32	conserved hypothetical protein	SAUSA300_0266
SAUSA300_0705	1,25	0,80	0,013710118	No	2739	2742	3001	2664	3016	2397	3420	3530	3685	ATP-dependent DNA helicase RecQ	recQ
SAUSA300_1078	1,25	0,80	0,000467939	Yes	4311	5139	4392	5551	5603	5680	5746	5810	5781	cell division protein	divIB
SAUSA300_0874	1,25	0,80	0,018553595	No	1469	1168	1414	1452	1553	1289	1646	1669	1757	conserved hypothetical protein	SAUSA300_0874
SAUSA300_1468	1,25	0,80	0,013202144	No	3088	3363	3294	3131	3210	2956	3790	4035	4382	DNA repair protein RecN	recN
SAUSA300_0509	1,25	0,80	0,014191079	No	1454	1864	1408	22215	21284	22166	2024	1866	2028	ATP guanido phosphotransferase	SAUSA300_0509
SAUSA300_1039	1,25	0,80	0,009589395	Yes	427	478	434	729	739	754	549	576	552	ribonuclease HIII	rnhC
SAUSA300_2261	1,25	0,80	0,075796261	No	1130	854	998	566	541	410	1163	1219	1348	conserved hypothetical protein	SAUSA300_2261
SAUSA300_0673	1,25	0,80	0,026177783	No	298	309	294	381	383	359	380	374	373	cobalamin synthesis protein/P47K family p	SAUSA300_0673
SAUSA300_1305	1,25	0,80	0,00356932	Yes	1169	1418	1183	1233	1255	1267	1537	1668	1511	2-oxoglutarate dehydrogenase, E2 compo	sucB
SAUSA300_1869	1,25	0,80	0,000418036	Yes	3138	2940	2911	4580	4055	4555	3835	3888	3505	methionine aminopeptidase	map
SAUSA300_0062	1,25	0,80	0,139320042	No	231	305	205	437	392	389	331	324	273	ornithine carbamoyltransferase	arcB

SAUSA300_0277	1,25	0,80	0,13863615	No	1411	1102	807	1320	1084	1319	1435	1386	1327	putative staphyloxanthin biosynthesis pro	SAUSA300_0277
SAUSA300_0918	1,25	0,80	0,034959637	No	4860	3773	3422	4786	4722	4124	5111	5140	4799	conserved hypothetical protein	SAUSA300_0918
SAUSA300_0280	1,25	0,80	0,306304865	No	417	229	226	156	124	80	393	312	381	conserved hypothetical protein	SAUSA300_0280
SAUSA300_0861	1,24	0,80	0,027596222	No	1436	1619	1479	2046	1706	2261	1784	2098	1775	NAD-specific glutamate dehydrogenase	gudB
SAUSA300_1603	1,24	0,81	0,021749624	No	12050	12670	13242	14619	13807	14585	16190	16321	14714	50S ribosomal protein L21	rplU
SAUSA300_1114	1,24	0,81	0,006304687	Yes	763	731	844	862	1055	884	993	943	975	ribosome small subunit-dependent GTPas	rsgA
SAUSA300_1021	1,24	0,81	0,498093189	No	105	57	127	23	32	19	118	89	157	hypothetical protein	SAUSA300_1021
SAUSA300_0652	1,24	0,81	0,257524818	No	648	520	617	464	458	356	734	677	813	putative membrane protein	SAUSA300_0652
SAUSA300_2146	1,24	0,81	0,009690977	Yes	796	872	652	1476	1404	1533	1009	969	900	alcohol dehydrogenase, zinc-containing	SAUSA300_2146
SAUSA300_1692	1,24	0,81	0,703428866	No	21	19	20	37	68	53	28	25	26	conserved hypothetical protein	SAUSA300_1692
SAUSA300_1886	1,24	0,81	0,002282582	Yes	2907	3662	3059	4613	4575	4877	4117	3968	3868	ATP-dependent DNA helicase, PcrA	pcrA
SAUSA300_2566	1,24	0,81	0,104076241	No	226	181	228	160	212	161	253	248	284	transcriptional regulator, Crp/Fnr family	arcR
SAUSA300_1349	1,24	0,81	0,001868308	Yes	846	820	895	1209	1321	1226	1105	1009	1065	glycosyl transferase, group 1 family protei	SAUSA300_1349
SAUSA300_2483	1,24	0,81	0,008542451	Yes	1526	1489	1383	2419	2168	2637	1764	1996	1691	hydroxymethylglutaryl-CoA reductase	SAUSA300_2483
SAUSA300_1421	1,24	0,81	0,765904925	No	0	0	0	0	0	0	1	0	0	phiSLT ORF122-like protein, DNA polymera	SAUSA300_1421
SAUSA300_0528	1,24	0,81	0,023824943	No	56002	61345	53942	78987	82356	79999	72424	74168	65775	DNA-directed RNA polymerase, beta' subu	rpoC
SAUSA300_1069	1,24	0,81	0,019394138	No	257	289	310	337	345	278	348	354	364	conserved hypothetical protein	SAUSA300_1069
SAUSA300_0165	1,24	0,81	0,078146956	No	195	172	155	215	207	186	230	193	220	capsular polysaccharide biosynthesis prote	cap5N
SAUSA300_1149	1,24	0,81	0,016671239	No	23932	21036	19897	20725	20815	19873	27490	28090	24694	30S ribosomal protein S2	rpsB
SAUSA300_2622	1,24	0,81	0,164960256	No	1135	1040	608	2089	2098	2137	1255	1084	1103	conserved hypothetical protein	SAUSA300_2622
SAUSA300_2178	1,24	0,81	0,052439618	No	18539	21458	17789	19572	19541	19159	24193	25650	21653	DNA-directed RNA polymerase alpha subu	rpoA
SAUSA300_1545	1,23	0,81	0,249210399	No	5720	4564	5843	1915	2340	1446	7072	6320	6607	30S ribosomal protein S20	rpsT
SAUSA300_0857	1,23	0,81	0,00886619	Yes	2677	3154	2783	3848	3597	4055	3367	3763	3502	conserved hypothetical protein	SAUSA300_0857
SAUSA300_0745	1,23	0,81	0,002747296	Yes	558	615	578	1038	994	1134	718	747	699	putative acetyltransferase	SAUSA300_0745
SAUSA300_0507	1,23	0,81	0,002662649	Yes	613	649	597	7178	7120	6974	799	750	746	transcriptional regulator CtsR	ctsR
SAUSA300_2068	1,23	0,81	0,053482825	No	1748	2532	2444	3635	3888	4303	2762	2917	2616	conserved hypothetical protein	SAUSA300_2068
SAUSA300_2235	1,23	0,81	0,236268632	No	1728	2784	2773	5095	5119	7094	3199	3461	2341	iron compound ABC transporter, iron com	SAUSA300_2235
SAUSA300_2042	1,23	0,81	0,113090094	No	189	196	160	146	113	136	249	220	202	conserved hypothetical protein	SAUSA300_2042
SAUSA300_0789	1,23	0,81	0,115915163	No	2100	1782	1351	3353	3471	3633	2453	2202	1787	putative thioredoxin	SAUSA300_0789
SAUSA300_0063	1,23	0,81	0,786814691	No	0	1	1	4	7	0	2	2	1	cyclic nucleotide-binding domain protein	SAUSA300_0063
SAUSA300_0746	1,23	0,81	0,313440012	No	625	639	910	541	613	460	862	803	1023	TPR domain protein	SAUSA300_0746
SAUSA300_0517	1,23	0,81	0,047439396	No	713	847	901	1276	1406	1529	993	1070	970	RNA methyltransferase, TrmH family, grou	SAUSA300_0517
SAUSA300_1216	1,23	0,82	0,055815316	No	1154	1033	1294	1240	1359	1164	1431	1451	1399	cardiolipin synthetase	SAUSA300_1216
SAUSA300_1178	1,23	0,82	0,042254086	No	10913	9742	8495	23354	21759	21721	11432	12736	11601	recombinase A protein	recA
SAUSA300_0687	1,23	0,82	0,004730749	Yes	4066	3612	4231	3918	4068	3364	4791	4991	4824	putative hemolysin	SAUSA300_0687
SAUSA300_0493	1,23	0,82	0,008010923	Yes	422	509	433	686	667	655	567	604	507	dihydroneopterin aldolase	folB
SAUSA300_0896	1,23	0,82	0,362844278	No	34	56	32	67	74	77	49	61	42	oligopeptide ABC transporter, permease p	oppC
SAUSA300_2395	1,22	0,82	0,176396088	No	3798	3088	3219	3902	4321	4072	4019	4304	4077	amino acid permease	SAUSA300_2395
SAUSA300_0228	1,22	0,82	0,533406997	No	34	29	18	39	59	37	35	39	26	acyl-CoA synthetase FadE	fadE
SAUSA300_2637	1,22	0,82	0,726273333	No	9	7	20	7	5	7	14	14	17	conserved hypothetical protein	SAUSA300_2637
SAUSA300_0477	1,22	0,82	0,116102284	No	2470	3864	3147	5234	4932	6468	3831	4153	3640	UDP-N-acetylglucosamine pyrophosphoryl	glmU
SAUSA300_1596	1,22	0,82	0,085109842	No	925	1306	1359	1645	1690	1751	1403	1469	1531	S-adenosylmethionine:tRNA ribosyltransfe	queA
SAUSA300_1208	1,22	0,82	0,768987751	No	9	4	34	0	5	9	12	14	36	conserved hypothetical protein	SAUSA300_1208
SAUSA300_1728	1,22	0,82	0,110516469	No	515	650	475	1340	1107	1338	737	624	649	oxidoreductase, aldo/keto reductase famil	SAUSA300_1728
SAUSA300_0825	1,22	0,82	0,148225709	No	1039	1455	1173	2420	2261	2756	1478	1494	1519	oxidoreductase, 2-nitropropane dioxygena	SAUSA300_0825
SAUSA300_2547	1,22	0,82	0,269775677	No	954	1007	736	980	802	985	1200	1037	1068	conserved hypothetical protein	SAUSA300_2547
SAUSA300_1362	1,22	0,82	0,096711888	No	86334	65394	67123	78261	95419	86145	97351	89033	81088	DNA-binding protein HU	hup
SAUSA300_1365	1,22	0,82	0,044947952	No	19618	21756	17565	28662	26670	26258	25098	23986	22871	30S ribosomal protein S1	rpsA
SAUSA300_1878	1,22	0,82	0,015182906	No	730	717	623	780	773	865	911	806	805	RNA methyltransferase, TrmA family	rumA
SAUSA300_1200	1,22	0,82	0,357713923	No	2074	2129	1681	1426	1521	1267	2763	2079	2362	glutamine synthetase repressor	glnR
SAUSA300_1420	1,22	0,82	0,72766065	No	0	0	0	1	0	0	1	1	0	conserved hypothetical phage protein	SAUSA300_1420
SAUSA300_1997	1,22	0,82	0,131058071	No	532	569	461	564	471	645	593	669	641	conserved hypothetical protein	SAUSA300_1997
SAUSA300_2075	1,22	0,82	0,001281267	Yes	8782	8153	8602	11712	11916	11488	10221	11419	9466	transcription termination factor Rho	rho
SAUSA300_1162	1,22	0,82	2,25E-05	Yes	9261	9980	9181	10231	10789	10458	11398	11942	11263	translation initiation factor IF-2	infB
SAUSA300_2149	1,22	0,82	0,282544818	No	171	171	95	175	180	201	193	149	185	6-phospho-beta-galactosidase	lacG
SAUSA300_0803	1,22	0,82	0,094472627	No	847	903	686	1527	1560	1238	1020	994	954	transcriptional regulator, Cro/CI family	SAUSA300_0803
SAUSA300_1273	1,22	0,82	0,097538728	No	245	249	231	284	252	241	303	265	313	oligopeptide permease, ATP-binding prote	opp-2F
SAUSA300_1060	1,22	0,82	0,647925781	No	9	10	13	6	11	9	14	11	14	putative exotoxin 4	SAUSA300_1060
SAUSA300_2224	1,21	0,82	0,169822712	No	517	854	744	1325	1195	1622	923	821	840	molybdopterin biosynthesis protein A	moeA

SAUSA300_0311	1,21	0,82	0,681506594	No	9	8	12	10	23	10	9	12	15	PfkB family carbohydrate kinase	SAUSA300_0311
SAUSA300_1487	1,21	0,83	0,455626777	No	40	37	30	59	74	49	42	45	42	replication initiation factor family protein	SAUSA300_1487
SAUSA300_0015	1,21	0,83	0,017980041	No	3911	4245	3487	5736	5646	5575	4795	4899	4414	50S ribosomal protein L9	rplI
SAUSA300_0029	1,21	0,83	0,457707352	No	126	89	110	89	115	65	130	125	140	conserved hypothetical protein	SAUSA300_0029
SAUSA300_2298	1,21	0,83	0,307670119	No	2961	1983	2274	2555	2191	2123	3155	3183	2428	multidrug resistance protein B, drug resist	SAUSA300_2298
SAUSA300_2568	1,21	0,83	0,376601433	No	31	74	61	77	70	102	64	76	65	arginine/ornithine antiporter	arcD
SAUSA300_0317	1,21	0,83	0,147586746	No	532	654	499	699	629	735	671	678	694	conserved hypothetical protein	SAUSA300_0317
SAUSA300_0971	1,21	0,83	0,269461481	No	2015	3612	3120	2069	1936	2956	3748	2945	3927	phosphoribosylformylglycinamidine synth	purL
SAUSA300_0180	1,21	0,83	0,104010882	No	231	282	280	316	316	336	288	314	361	integral membrane protein LmrP	SAUSA300_0180
SAUSA300_0946	1,21	0,83	0,040922729	No	1824	1730	1805	2496	2495	2650	2190	2110	2178	2-succinyl-6-hydroxy-2,4-cyclohexadiene-1	menD
SAUSA300_2219	1,21	0,83	0,035877577	No	1652	1745	1551	2539	2405	2336	2119	1999	1855	molybdenum cofactor biosynthesis protei	moaA
SAUSA300_0674	1,21	0,83	0,009700454	Yes	1411	1278	1379	1668	1720	1693	1638	1729	1541	oxidoreductase, aldo/keto reductase famil	SAUSA300_0674
SAUSA300_0639	1,21	0,83	0,352324726	No	5963	3552	4994	2596	2488	1786	5932	5775	5859	conserved hypothetical protein	SAUSA300_0639
SAUSA300_0734	1,21	0,83	0,252893121	No	72	75	60	59	47	72	89	77	83	putative comf operon protein 1	SAUSA300_0734
SAUSA300_1594	1,20	0,83	0,144940112	No	2153	1545	2191	1139	1242	938	2448	2234	2427	preprotein translocase, YajC subunit	yajC
SAUSA300_0061	1,20	0,83	0,095023475	No	372	393	308	584	591	683	472	407	413	carbamate kinase	arcC
SAUSA300_1661	1,20	0,83	0,038582625	No	541	546	639	607	703	719	668	703	715	thiamine biosynthesis protein ThiI	thiI
SAUSA300_1952	1,20	0,83	0,787474145	No	0	0	5	20	14	27	4	4	0	phiPV083 ORF027-like protein	SAUSA300_1952
SAUSA300_1387	1,20	0,83	0,754739886	No	3	1	7	9	9	13	5	5	6	phiSLT ORF129-like protein	SAUSA300_1387
SAUSA300_0912	1,20	0,83	2,80E-06	Yes	5076	5136	5298	7553	7826	7660	6248	6482	5962	trans-2-enoyl-ACP reductase	SAUSA300_0912
SAUSA300_1116	1,20	0,83	0,008587712	Yes	828	966	876	1202	1260	1254	1038	1151	1034	thiamine pyrophosphokinase	SAUSA300_1116
SAUSA300_0974	1,20	0,83	0,047471436	No	1982	2775	2547	1226	1379	1328	2963	2788	3061	phosphoribosylglycinamide formyltransfer	purN
SAUSA300_1043	1,20	0,83	0,002506581	Yes	1800	1966	1733	2655	2766	2532	2297	2170	2154	DNA mismatch repair MutS2 protein	mutS2
SAUSA300_1024	1,20	0,83	0,056381598	No	622	617	551	599	651	489	729	694	729	phosphopantetheine adenylyltransferase	coaD
SAUSA300_0347	1,20	0,83	0,164747716	No	818	724	676	1160	1296	1007	929	931	805	Sec-independent protein translocase TatC	tatC
SAUSA300_0377	1,20	0,83	0,08657507	No	1529	1158	1379	1086	1086	796	1486	1710	1688	putative lipoprotein	SAUSA300_0377
SAUSA300_1845	1,20	0,83	0,00457911	Yes	1383	1508	1394	2410	2416	2625	1668	1819	1656	glutamate-1-semialdehyde-2,1-aminomuta	hemL
SAUSA300_0353	1,20	0,83	0,151041486	No	854	955	964	636	638	560	1245	1058	1029	conserved hypothetical protein	SAUSA300_0353
SAUSA300_1346	1,20	0,83	0,019467024	No	2606	2367	2605	2452	2547	2157	2966	2989	3128	putative DnaQ family exonuclease/DinG fa	SAUSA300_1346
SAUSA300_1993	1,20	0,84	0,003379643	Yes	973	1135	1131	1681	1517	1680	1310	1342	1240	kinase, pfkB family	SAUSA300_1993
SAUSA300_0520	1,20	0,84	0,039401321	No	1190	1235	1265	1090	1001	938	1508	1434	1483	preprotein translocase, SecE subunit	SAUSA300_0520
SAUSA300_0466	1,20	0,84	0,014868789	No	365	381	347	510	572	578	443	441	424	conserved hypothetical protein	SAUSA300_0466
SAUSA300_0459	1,20	0,84	0,160355613	No	255	311	338	390	518	463	365	375	349	thymidylate kinase	tmk
SAUSA300_1943	1,20	0,84	0,6331571	No	29	16	14	105	115	139	12	29	29	phi77 ORF040-like protein	SAUSA300_1943
SAUSA300_1888	1,20	0,84	0,126187363	No	885	761	748	614	629	586	1046	881	937	conserved hypothetical protein	SAUSA300_1888
SAUSA300_2189	1,20	0,84	0,214635739	No	6231	7330	7767	7613	7995	8093	8669	9183	7708	50S ribosomal protein L6	rplF
SAUSA300_1250	1,20	0,84	0,027930918	No	3398	4230	3709	5294	5509	5566	4491	4566	4511	DNA topoisomerase IV, subunit B	parE
SAUSA300_2171	1,20	0,84	0,163347154	No	18329	17566	16247	18087	19598	21922	21033	22514	18893	30S ribosomal protein S9	rpsI
SAUSA300_1560	1,19	0,84	0,262736858	No	117	137	149	67	106	95	148	157	180	conserved hypothetical protein	SAUSA300_1560
SAUSA300_1635	1,19	0,84	0,015622243	No	930	1136	1064	1645	1479	1405	1307	1224	1219	formamidopyrimidine-DNA glycosylase	mutM
SAUSA300_1711	1,19	0,84	0,50407437	No	47	37	62	53	45	35	49	66	61	proline dehydrogenase	putA
SAUSA300_1851	1,19	0,84	0,294092451	No	320	281	295	241	268	250	368	329	374	putative membrane protein	SAUSA300_1851
SAUSA300_1884	1,19	0,84	0,012963802	No	3303	3263	3242	3390	3618	2814	3999	3873	3842	CamS sex pheromone cAM373 precursor	SAUSA300_1884
SAUSA300_2062	1,19	0,84	0,018328094	No	5908	6885	6441	7547	7614	8210	7640	7875	7459	ATP synthase F0, B subunit	atpF
SAUSA300_0007	1,19	0,84	0,334226661	No	605	560	520	845	965	868	701	733	581	conserved hypothetical protein	SAUSA300_0007
SAUSA300_0933	1,19	0,84	0,73636192	No	45	81	228	83	110	130	100	117	220	conserved hypothetical protein	SAUSA300_0933
SAUSA300_1341	1,19	0,84	0,002154856	Yes	22454	22390	21402	33253	36567	36425	25679	27412	25940	penicillin binding protein 2	pbp2
SAUSA300_0488	1,19	0,84	0,133660905	No	913	935	1139	869	987	726	1224	1117	1229	hypoxanthine phosphoribosyltransferase	hpt
SAUSA300_2532	1,19	0,84	0,05205678	No	1068	1340	1115	1338	1370	1396	1469	1339	1398	aspartate 1-decarboxylase	panD
SAUSA300_2060	1,19	0,84	0,007671444	Yes	18164	21075	19358	23061	23776	24117	23179	24537	22102	ATP synthase F1, alpha subunit	atpA
SAUSA300_0104	1,19	0,84	0,017219019	No	2267	2133	1834	2490	2362	2183	2466	2562	2389	transcriptional regulator, AraC family	SAUSA300_0104
SAUSA300_0802	1,19	0,84	0,075776003	No	1242	1332	946	2154	2067	1822	1423	1421	1341	conserved hypothetical protein	SAUSA300_0802
SAUSA300_1306	1,19	0,84	0,06533892	No	2167	2671	2136	2314	2013	2048	2597	3113	2593	2-oxoglutarate dehydrogenase, E1 compo	sucA
SAUSA300_2074	1,19	0,84	0,131799611	No	11822	10387	11880	7835	10111	7623	14604	14230	11729	50S ribosomal protein L31 type B	rpmE
SAUSA300_1165	1,19	0,84	0,016671239	No	1860	1951	1618	2685	2725	2784	2113	2169	2162	riboflavin biosynthesis protein ribF	ribF
SAUSA300_1624	1,19	0,84	0,116138424	No	629	902	895	1015	1089	1116	991	969	933	MutT/nudix family protein	SAUSA300_1624
SAUSA300_0985	1,19	0,84	0,644995061	No	133	83	139	32	68	13	135	129	162	conserved hypothetical protein	SAUSA300_0985
SAUSA300_0973	1,19	0,84	0,120835116	No	2895	4413	3716	2025	2123	2498	4380	4195	4527	phosphoribosylformylglycinamidine cyclo-	purM
SAUSA300_0515	1,19	0,84	0,096953424	No	980	1303	1338	1426	1384	1461	1324	1519	1463	cysteinyl-tRNA synthetase	cysS

SAUSA300_0590	1,18	0,84	0,049500386	No	1349	1331	1159	1660	1479	1625	1521	1626	1400	conserved hypothetical protein	SAUSA300_0590
SAUSA300_1482	1,18	0,85	0,681224054	No	12	5	19	23	25	12	18	15	12	FtsK/SpoIIIE family protein	SAUSA300_1482
SAUSA300_0690	1,18	0,85	0,357713923	No	16162	9137	8088	8621	9624	7101	13140	12699	13749	sensor histidine kinase SaeS	saeS
SAUSA300_0573	1,18	0,85	0,182375608	No	1479	1441	1266	1766	1690	2118	1728	1661	1567	diphosphomevalonate decarboxylase	mvaD
SAUSA300_0458	1,18	0,85	0,075008715	No	276	327	289	398	419	373	363	356	338	Orn/Lys/Arg decarboxylase	SAUSA300_0458
SAUSA300_0167	1,18	0,85	0,071086365	No	649	652	543	740	778	818	762	727	685	capsular polysaccharide biosynthesis prote	cap5P
SAUSA300_1794	1,18	0,85	0,722223504	No	10	11	7	6	9	1	11	11	11	conserved hypothetical protein	SAUSA300_1794
SAUSA300_1050	1,18	0,85	0,021742327	No	2726	2397	2189	4150	3904	3478	2980	2769	2870	non-canonical purine NTP pyrophosphata:	SAUSA300_1050
SAUSA300_0518	1,18	0,85	0,151051147	No	756	879	840	799	764	695	915	1018	994	conserved hypothetical protein	SAUSA300_0518
SAUSA300_1141	1,18	0,85	0,664352349	No	36	21	60	17	23	13	37	44	59	endopeptidase resistance gene	SAUSA300_1141
SAUSA300_1269	1,18	0,85	0,118806205	No	5451	4813	5120	4422	4988	3545	6274	5788	6069	methicillin resistance protein FemA	femA
SAUSA300_0042	1,18	0,85	0,047366913	No	1188	1140	1226	1434	1415	1406	1440	1339	1406	conserved hypothetical protein	SAUSA300_0042
SAUSA300_2111	1,17	0,85	0,016011755	No	6521	7249	6233	8190	7650	8163	8436	7769	7309	phosphoglucosamine mutase	glmM
SAUSA300_0232	1,17	0,85	0,533199222	No	98	52	70	122	92	108	83	95	81	conserved hypothetical protein	SAUSA300_0232
SAUSA300_2032	1,17	0,85	0,393648238	No	65	86	64	90	108	135	74	91	90	K+-transporting ATPase, C subunit	kdpC
SAUSA300_1789	1,17	0,85	0,750652568	No	43	12	82	23	29	22	58	31	76	conserved hypothetical protein	SAUSA300_1789
SAUSA300_0899	1,17	0,85	0,231209336	No	6364	4287	4925	8098	7753	6876	6812	5584	5919	putative negative regulator of genetic con	SAUSA300_0899
SAUSA300_2049	1,17	0,85	0,416960913	No	60	85	40	153	113	136	70	68	78	phosphomethylpyrimidine kinase	thiD
SAUSA300_1887	1,17	0,85	0,020000745	No	701	883	802	1004	1055	945	961	954	895	geranylgeranylglyceryl phosphate synthase	pcrB
SAUSA300_0966	1,17	0,85	0,591046248	No	203	719	299	441	291	535	503	447	496	phosphoribosylaminoimidazole carboxylas	purE
SAUSA300_2447	1,17	0,85	0,420351046	No	3343	2612	3166	2336	1841	2817	4026	3562	3147	conserved hypothetical protein	SAUSA300_2447
SAUSA300_1042	1,17	0,85	0,092633335	No	1073	964	1034	1247	1240	1041	1253	1135	1216	DNA-dependent DNA polymerase family X	SAUSA300_1042
SAUSA300_2061	1,17	0,85	0,139994637	No	4325	4498	5108	2952	3135	2367	5194	5402	5763	ATP synthase F1, delta subunit	atpH
SAUSA300_2624	1,17	0,85	0,277670717	No	134	156	183	119	97	78	184	178	197	putative membrane protein	SAUSA300_2624
SAUSA300_1666	1,17	0,85	0,088205898	No	24616	21400	21302	19587	20094	18356	26871	27915	24164	30S ribosomal protein S4	rpsD
SAUSA300_1503	1,17	0,85	0,743055786	No	14	14	16	7	14	6	21	14	17	putative competence protein ComGB	SAUSA300_1503
SAUSA300_2131	1,17	0,85	0,386520975	No	422	274	442	338	270	234	430	465	442	conserved hypothetical protein	SAUSA300_2131
SAUSA300_0658	1,17	0,85	0,008642575	Yes	1693	1688	1821	1471	1467	1258	1967	2046	2085	transcriptional regulator, LysR family	SAUSA300_0658
SAUSA300_2133	1,17	0,85	0,087220315	No	1192	1161	1336	1025	1289	1063	1430	1528	1367	transporter gate domain protein	SAUSA300_2133
SAUSA300_0226	1,17	0,85	0,527417664	No	29	53	43	59	90	71	52	54	45	3-hydroxyacyl-CoA dehydrogenase	SAUSA300_0226
SAUSA300_1115	1,17	0,85	0,064781849	No	818	869	855	935	1021	750	950	1058	973	ribulose-phosphate 3-epimerase	rpe
SAUSA300_0791	1,17	0,86	0,122209646	No	4003	4230	4173	5050	4855	4904	5137	5081	4293	glycine cleavage system H protein	gcvH
SAUSA300_1223	1,17	0,86	0,36433328	No	1262	994	1338	788	703	581	1410	1254	1545	conserved hypothetical protein	SAUSA300_1223
SAUSA300_1460	1,17	0,86	0,173500846	No	1342	1823	1437	3269	2869	3647	1838	1750	1798	peptidase, M20/M25/M40 family	SAUSA300_1460
SAUSA300_0336	1,17	0,86	0,32445564	No	165	130	151	89	131	74	161	194	165	conserved hypothetical protein	SAUSA300_0336
SAUSA300_1106	1,17	0,86	0,561410212	No	1598	661	816	1094	929	762	1271	1182	1155	putative lipoprotein	SAUSA300_1106
SAUSA300_2077	1,17	0,86	0,140339985	No	2950	2319	1968	3262	2966	3104	2846	2658	2941	conserved hypothetical protein	SAUSA300_2077
SAUSA300_0765	1,17	0,86	0,114225756	No	1462	1422	1419	1647	1697	1760	1661	1798	1568	SsrA-binding protein	smpB
SAUSA300_0334	1,17	0,86	0,343661572	No	133	126	110	126	126	170	139	141	148	transcriptional regulator, MarR family	SAUSA300_0334
SAUSA300_0454	1,17	0,86	0,198201776	No	2358	2887	3263	4713	4675	5934	3337	3466	3147	recombination protein RecR	recR
SAUSA300_0575	1,17	0,86	0,307440912	No	665	637	510	726	755	837	877	636	601	conserved hypothetical protein	SAUSA300_0575
SAUSA300_1125	1,17	0,86	0,354430276	No	7728	8728	9863	5085	7188	5122	12966	9804	7989	acyl carrier protein	acpP
SAUSA300_1882	1,17	0,86	0,02572841	No	3422	3621	3233	3321	3498	3281	4246	4114	3624	aspartyl/glutamyl-tRNA amidotransferase	gatC
SAUSA300_1646	1,17	0,86	0,028711434	No	3024	3209	2991	4109	4224	3997	3729	3620	3408	acetyl-CoA carboxylase, carboxyl transfera	accA
SAUSA300_1144	1,16	0,86	0,195972687	No	6092	6863	6189	7783	8835	8457	7291	7661	7377	glucose inhibited division protein	gid
SAUSA300_0723	1,16	0,86	0,794830602	No	16	8	28	4	5	1	21	12	28	conserved hypothetical protein	SAUSA300_0723
SAUSA300_2308	1,16	0,86	0,253781628	No	203	287	194	301	264	312	259	291	249	response regulator protein	SAUSA300_2308
SAUSA300_1687	1,16	0,86	0,018973433	No	13105	13209	11282	14753	14923	14088	14798	14546	14440	FtsK/SpoIIIE family protein	SAUSA300_1687
SAUSA300_0478	1,16	0,86	0,038315074	No	6349	6572	5502	7455	7551	8333	7359	7371	6720	ribose-phosphate pyrophosphokinase	prs
SAUSA300_0975	1,16	0,86	0,095069261	No	6330	8841	7105	4091	4206	4623	8627	8358	8947	bifunctional purine biosynthesis protein	purH
SAUSA300_2635	1,16	0,86	0,806889082	No	3	4	7	3	16	6	4	9	5	conserved hypothetical protein	SAUSA300_2635
SAUSA300_1166	1,16	0,86	0,122988407	No	16962	13416	14096	12156	14053	12490	17486	17998	16235	30S ribosomal protein S15	rpsO
SAUSA300_1241	1,16	0,86	0,459433121	No	420	292	405	371	295	269	491	378	431	conserved hypothetical protein	SAUSA300_1241
SAUSA300_0648	1,16	0,86	0,31567445	No	1157	892	1077	520	575	455	1188	1089	1356	ABC transporter, permease protein	SAUSA300_0648
SAUSA300_0641	1,16	0,86	0,190140035	No	815	772	722	941	1007	821	853	796	1029	putative lipase/esterase	SAUSA300_0641
SAUSA300_2195	1,16	0,86	0,399429744	No	2838	3203	4115	2933	3097	2932	3918	3876	4015	30S ribosomal protein S17	rpsQ
SAUSA300_0221	1,16	0,86	0,74723361	No	84	105	140	112	128	136	87	228	76	pyruvate formate-lyase activating enzyme	pflA
SAUSA300_0646	1,16	0,86	0,081978143	No	1044	920	844	985	965	929	1137	1092	1015	sensor histidine kinase	SAUSA300_0646
SAUSA300_1535	1,16	0,87	0,547419415	No	5152	5300	6517	1952	3092	1921	7049	6998	5652	30S ribosomal protein S21	rpsU

SAUSA300_1508	1,16	0,87	0,571119811	No	59	60	98	49	36	38	71	72	110	conserved hypothetical protein	SAUSA300_1508
SAUSA300_0747	1,16	0,87	0,33712533	No	2580	3502	3917	8485	7787	10478	3860	3736	3985	thioredoxin-disulfide reductase	trxB
SAUSA300_1688	1,16	0,87	0,116138424	No	810	794	764	848	998	974	934	900	901	phenylalanyl-tRNA synthetase (beta subur	SAUSA300_1688
SAUSA300_1449	1,15	0,87	0,210236451	No	327	305	317	440	428	426	375	339	382	MutT/nudix family protein	SAUSA300_1449
SAUSA300_1415	1,15	0,87	0,859282557	No	0	1	1	3	2	0	2	1	0	phiSLT ORF 77-like protein	SAUSA300_1415
SAUSA300_1344	1,15	0,87	0,277488016	No	625	598	610	470	399	382	797	636	683	putative DNA replication protein DnaD	SAUSA300_1344
SAUSA300_1627	1,15	0,87	0,093974168	No	11891	10236	9882	8593	8648	8416	13132	12920	10815	translation initiation factor IF-3	infC
SAUSA300_1867	1,15	0,87	0,043871928	No	708	691	630	826	908	788	805	770	756	conserved hypothetical protein	SAUSA300_1867
SAUSA300_1074	1,15	0,87	0,14280327	No	5725	5564	5317	4863	4605	4719	6028	7094	5990	cell division protein	ftsL
SAUSA300_2309	1,15	0,87	0,069609895	No	782	680	684	527	566	433	831	776	855	sensor histidine kinase	SAUSA300_2309
SAUSA300_2058	1,15	0,87	0,081684734	No	23334	26870	23178	32109	32211	34656	28308	30176	25949	ATP synthase F1, beta subunit	atpD
SAUSA300_0378	1,15	0,87	0,652911674	No	167	100	190	90	122	56	161	160	208	conserved hypothetical protein	SAUSA300_0378
SAUSA300_0844	1,15	0,87	0,018964746	No	22616	22164	20527	23396	23896	23484	25405	27136	22436	conserved hypothetical protein	SAUSA300_0844
SAUSA300_0833	1,15	0,87	0,05329122	No	1573	1796	1710	2016	1796	1986	1920	2022	1891	conserved hypothetical protein	SAUSA300_0833
SAUSA300_0491	1,15	0,87	0,396820336	No	9335	13683	9114	20782	18534	24498	13705	11958	11226	cysteine synthase A	cysK
SAUSA300_0322	1,15	0,87	0,347250638	No	518	664	512	709	780	893	607	707	630	NADH-dependent flavin oxidoreductase, C	SAUSA300_0322
SAUSA300_0139	1,15	0,87	0,416960913	No	110	136	133	115	140	121	148	137	152	putative tetracycline resistance protein	SAUSA300_0139
SAUSA300_0485	1,15	0,87	0,216652786	No	1016	1244	1000	1627	1690	1591	1312	1295	1132	cell-division initiation protein	SAUSA300_0485
SAUSA300_0851	1,15	0,87	0,492579862	No	1040	887	1356	713	841	540	1186	1203	1382	Na(+)/H(+) antiporter subunit E	mnhE
SAUSA300_0487	1,15	0,87	0,043871928	No	1154	1205	1159	1538	1510	1607	1439	1328	1265	MesJ/Ycf62 family protein	SAUSA300_0487
SAUSA300_1605	1,14	0,87	0,21245531	No	949	1118	1080	1091	1107	1066	1231	1182	1194	rod shape-determining protein MreC	mreC
SAUSA300_2059	1,14	0,87	0,06741003	No	11807	13765	12640	13851	13388	13047	14323	14736	14643	ATP synthase F1, gamma subunit	atpG
SAUSA300_1105	1,14	0,88	0,060008961	No	2509	3048	2568	4544	4517	4691	3157	3196	2942	primosomal protein N`	priA
SAUSA300_0724	1,14	0,88	0,42597381	No	591	480	597	407	451	299	648	567	694	putative lipoprotein	SAUSA300_0724
SAUSA300_1703	1,14	0,88	0,437737076	No	257	301	370	284	257	359	375	322	367	rhodanese-like domain protein	SAUSA300_1703
SAUSA300_0209	1,14	0,88	0,616750609	No	45	77	71	66	45	59	64	90	69	putative maltose ABC transporter, maltose	SAUSA300_0209
SAUSA300_1977	1,14	0,88	0,722223504	No	31	31	57	17	29	18	47	39	52	conserved hypothetical protein	SAUSA300_1977
SAUSA300_1625	1,14	0,88	0,215690833	No	12380	10299	10592	9198	9225	8976	13122	13482	11384	50S ribosomal protein L20	rplT
SAUSA300_1918	1,14	0,88	0,756660225	No	269	86	61	56	54	33	231	109	136	truncated beta-hemolysin	SAUSA300_1918
SAUSA300_1865	1,14	0,88	0,078543646	No	2079	2060	1779	3271	3205	3141	2347	2272	2123	DNA-binding response regulator	vraR
SAUSA300_1131	1,14	0,88	0,411169369	No	4289	3727	3541	2804	3302	2819	4666	4347	4173	30S ribosomal protein S16	rpsP
SAUSA300_1463	1,14	0,88	0,210236451	No	999	869	832	1507	1375	1752	1110	995	966	conserved hypothetical protein	SAUSA300_1463
SAUSA300_1389	1,14	0,88	0,565538181	No	64	111	55	116	142	191	91	81	90	phiSLT ORF636-like protein	SAUSA300_1389
SAUSA300_1159	1,14	0,88	0,008852523	Yes	3613	3721	3414	4003	4282	4161	4150	4105	3972	transcription termination factor NusA	nusA
SAUSA300_0972	1,14	0,88	0,330472433	No	3121	4777	4038	2393	2495	3182	4603	4154	4846	amidophosphoribosyltransferase	purF
SAUSA300_1224	1,14	0,88	0,668742089	No	231	136	226	64	108	59	203	211	262	conserved hypothetical protein	SAUSA300_1224
SAUSA300_1158	1,14	0,88	0,038582625	No	1786	1844	1574	1764	1677	1604	2032	1986	1893	conserved hypothetical protein	SAUSA300_1158
SAUSA300_2378	1,14	0,88	0,210681606	No	754	860	890	852	784	893	907	958	987	conserved hypothetical protein	SAUSA300_2378
SAUSA300_0633	1,14	0,88	0,423938588	No	1247	1461	1367	1930	1521	2447	1519	1671	1448	ferrichrome transport ATP-binding proteir	fhuA
SAUSA300_2266	1,13	0,88	0,437697553	No	723	491	646	663	665	566	767	718	628	conserved hypothetical protein	SAUSA300_2266
SAUSA300_2211	1,13	0,88	0,261357041	No	398	371	345	427	440	398	436	414	412	putative membrane protein	SAUSA300_2211
SAUSA300_1654	1,13	0,88	0,293316038	No	1924	2029	1616	2699	2436	2335	2218	2174	1924	proline dipeptidase	SAUSA300_1654
SAUSA300_1543	1,13	0,88	0,211886682	No	338	381	359	448	469	426	423	385	415	oxygen-independent coproporphyrinogen	SAUSA300_1543
SAUSA300_1662	1,13	0,88	0,264997841	No	410	435	460	382	430	379	474	514	494	aminotransferase, class V	SAUSA300_1662
SAUSA300_1239	1,13	0,88	0,107711057	No	21416	21600	17633	31088	31685	32070	23894	24057	20739	transketolase	tkt
SAUSA300_2004	1,13	0,89	0,305177913	No	961	957	853	1209	1080	1168	1107	976	1048	conserved hypothetical protein	SAUSA300_2004
SAUSA300_1526	1,13	0,89	0,149449289	No	763	873	891	1136	1159	1158	926	955	982	DNA repair protein RecO	recO
SAUSA300_1636	1,13	0,89	0,17315843	No	2980	3343	3023	3815	3460	3508	3691	3445	3429	DNA polymerase I superfamily	polA
SAUSA300_1622	1,13	0,89	0,17359965	No	21460	22103	19251	28391	26474	26767	24869	24708	21413	trigger factor	tig
SAUSA300_1270	1,13	0,89	0,200655566	No	7508	6321	6718	6230	6728	5522	7529	7636	8038	methicillin resistance protein FemB	femB
SAUSA300_0834	1,13	0,89	0,031161074	No	2013	2179	2067	2565	2353	2447	2307	2458	2296	D-isomer specific 2-hydroxyacid dehydrog	SAUSA300_0834
SAUSA300_1783	1,13	0,89	0,291148148	No	1047	1269	1110	2127	1864	2044	1289	1276	1300	uroporphyrinogen decarboxylase	hemE
SAUSA300_1163	1,13	0,89	0,420351046	No	1076	868	1260	1012	967	843	1201	1131	1280	ribosome-binding factor A	rbfA
SAUSA300_0348	1,12	0,89	0,442847443	No	706	523	552	904	956	772	706	671	623	twin-arginine translocation protein, TatA/I	SAUSA300_0348
SAUSA300_2419	1,12	0,89	0,396767977	No	289	204	186	213	255	198	262	225	268	conserved hypothetical protein	SAUSA300_2419
SAUSA300_0427	1,12	0,89	0,185883952	No	846	834	1066	856	922	790	992	1056	1041	conserved hypothetical protein	SAUSA300_0427
SAUSA300_0910	1,12	0,89	0,182655427	No	1619	1960	1639	2214	2328	2146	2059	1957	1839	magnesium transporter	mgtE
SAUSA300_2136	1,12	0,89	0,57785398	No	1147	1585	1342	2735	2387	2850	1486	1532	1560	iron compound ABC transporter, iron com	SAUSA300_2136
SAUSA300_2172	1,12	0,89	0,348835129	No	15911	15735	14301	14577	15315	15154	17453	18277	15796	50S ribosomal protein L13	rplM

SAUSA300_1699	1,12	0,89	0,16751111	No	2275	2337	2330	1887	2263	1838	2563	2575	2639	pseudouridine synthase, family 1	SAUSA300_1699
SAUSA300_1248	1,12	0,89	0,371163792	No	574	563	676	1011	924	1020	719	684	632	conserved hypothetical protein	SAUSA300_1248
SAUSA300_0428	1,12	0,89	0,874503534	No	2	4	2	0	0	1	3	5	2	conserved hypothetical protein	SAUSA300_0428
SAUSA300_0959	1,12	0,89	0,591090386	No	1137	817	1222	485	491	342	1110	1069	1383	fmt protein	fmt
SAUSA300_0884	1,12	0,89	0,669471239	No	1082	955	1215	211	282	180	1384	1032	1237	conserved hypothetical protein	SAUSA300_0884
SAUSA300_1045	1,12	0,89	0,334371788	No	696	834	644	884	764	902	852	772	812	excinuclease ABC, C subunit	uvrC
SAUSA300_0853	1,12	0,89	0,304950198	No	1254	1187	1238	992	1010	722	1353	1389	1375	Na(+)/H(+) antiporter subunit C	mnhC
SAUSA300_1140	1,12	0,89	0,678029679	No	74	79	92	66	79	70	65	75	135	cell wall hydrolase	lytN
SAUSA300_0546	1,12	0,89	0,521343159	No	920	938	1086	1120	974	1236	912	1213	1172	sdrC protein	sdrC
SAUSA300_1859	1,12	0,90	0,210992541	No	2304	2490	2150	3184	3246	3568	2521	2634	2604	conserved hypothetical protein	SAUSA300_1859
SAUSA300_1259	1,12	0,90	0,549685488	No	310	222	190	980	935	875	266	278	259	ImpB/MucB/SamB family protein	SAUSA300_1259
SAUSA300_0611	1,12	0,90	0,884461561	No	0	0	0	4	5	3	1	1	0	putative Na+/H+ antiporter, MnhB compo	SAUSA300_0611
SAUSA300_1100	1,12	0,90	0,273291667	No	1018	793	797	1230	1118	1051	1009	939	956	conserved hypothetical protein	SAUSA300_1100
SAUSA300_0088	1,12	0,90	0,696819811	No	40	42	36	29	23	21	53	36	42	hypothetical protein	SAUSA300_0088
SAUSA300_1470	1,12	0,90	0,048752636	No	3686	4145	3694	4258	4222	4284	4392	4238	4228	geranyltranstransferase	SAUSA300_1470
SAUSA300_0242	1,12	0,90	0,622672089	No	34	59	56	79	59	80	62	60	49	sorbitol dehydrogenase	gutB
SAUSA300_0709	1,11	0,90	0,333860577	No	999	793	882	918	978	762	1019	1022	938	5'(3')-deoxyribonucleotidase	SAUSA300_0709
SAUSA300_0830	1,11	0,90	0,452134349	No	420	411	419	258	250	254	514	380	500	conserved hypothetical protein	SAUSA300_0830
SAUSA300_0530	1,11	0,90	0,328127695	No	26731	27467	25289	22081	23977	23583	28974	31834	27802	ribosomal protein S12	rpsL
SAUSA300_0452	1,11	0,90	0,380244444	No	1762	2141	2267	3027	2673	3556	2261	2359	2261	DNA polymerase III, gamma and tau subur	dnaX
SAUSA300_1251	1,11	0,90	0,243438622	No	4154	4345	4556	4517	4799	3895	4583	4859	5099	DNA topoisomerase IV, subunit A	parC
SAUSA300_0656	1,11	0,90	0,085335887	No	1297	1347	1353	1475	1596	1449	1471	1620	1366	conserved hypothetical protein	SAUSA300_0656
SAUSA300_0911	1,11	0,90	0,082850496	No	1674	1666	1754	2029	1977	1971	1986	1837	1850	transporter, monovalent cation:proton an	SAUSA300_0911
SAUSA300_1201	1,11	0,90	0,550169005	No	13860	16753	11483	13124	12973	13801	17325	14642	14946	glutamine synthetase, type I	glnA
SAUSA300_2459	1,11	0,90	0,50894717	No	189	218	170	179	174	235	219	233	191	transcriptional regulator, MarR family	SAUSA300_2459
SAUSA300_2578	1,11	0,90	0,181648301	No	7079	6706	5841	8300	8423	8689	7070	7409	7338	putative phage infection protein	SAUSA300_2578
SAUSA300_2067	1,11	0,90	0,336335996	No	5437	7486	7146	10102	10372	12151	7472	7919	6943	serine hydroxymethyltransferase	glyA
SAUSA300_0678	1,11	0,90	0,791700903	No	134	90	155	137	214	140	134	112	179	putative membrane protein	SAUSA300_0678
SAUSA300_2369	1,11	0,90	0,674206035	No	50	79	68	92	68	56	65	76	80	6-carboxyhexanoate--CoA ligase	SAUSA300_2369
SAUSA300_2064	1,11	0,90	0,357294129	No	7478	8830	8057	8173	8107	7490	9283	9482	8308	ATP synthase F0, A subunit	atpB
SAUSA300_0004	1,11	0,90	0,142261226	No	3326	3655	3462	3953	3629	3667	3835	3987	3777	DNA replication and repair protein recF	recF
SAUSA300_1090	1,11	0,90	0,20610342	No	1442	1332	1437	1726	1990	1785	1519	1585	1571	conserved hypothetical protein	SAUSA300_1090
SAUSA300_0192	1,11	0,90	0,74723361	No	22	38	29	20	25	25	27	29	45	conserved hypothetical protein	SAUSA300_0192
SAUSA300_1781	1,11	0,90	0,209210345	No	1479	1774	1667	3016	2772	2949	1858	1745	1862	protoporphyrinogen oxidase	hemG
SAUSA300_0035	1,11	0,90	0,28963905	No	5577	4972	4604	5435	5955	6064	5482	6380	4913	truncated hypothetical protein	SAUSA300_0035
SAUSA300_0597	1,11	0,90	0,735037065	No	28	42	30	19	16	19	36	41	35	putative endonuclease III	SAUSA300_0597
SAUSA300_1370	1,11	0,90	0,334371788	No	17373	19213	14794	23077	21879	25037	19430	18719	18672	cell surface elastin binding protein	ebpS
SAUSA300_2350	1,10	0,91	0,497358411	No	419	338	357	547	498	489	457	424	349	conserved hypothetical protein	SAUSA300_2350
SAUSA300_1197	1,10	0,91	0,496096048	No	1452	1573	1231	1618	1476	1661	1685	1537	1483	glutathione peroxidase	SAUSA300_1197
SAUSA300_2088	1,10	0,91	0,441786701	No	2384	2181	1871	3255	2955	3447	2564	2450	2097	S-ribosylhomocysteinase	luxS
SAUSA300_0220	1,10	0,91	0,876398821	No	167	178	334	182	169	191	116	540	114	formate acetyltransferase	pflB
SAUSA300_0569	1,10	0,91	0,396767977	No	3989	3488	3220	4687	4057	3882	4143	3899	3772	conserved hypothetical protein	SAUSA300_0569
SAUSA300_0649	1,10	0,91	0,691333737	No	878	704	1132	378	458	293	913	888	1203	conserved hypothetical protein	SAUSA300_0649
SAUSA300_0227	1,10	0,91	0,876473534	No	2	3	6	9	7	10	5	4	4	acyl-CoA dehydrogenase FadD	fadD
SAUSA300_1720	1,10	0,91	0,389584433	No	8808	7044	6448	9521	9527	9625	8582	7553	8473	conserved hypothetical protein	SAUSA300_1720
SAUSA300_0178	1,10	0,91	0,56776601	No	2954	2940	2484	2482	1999	2242	3293	2754	3204	conserved hypothetical protein	SAUSA300_0178
SAUSA300_0511	1,10	0,91	0,31340029	No	2179	2936	2713	3456	3527	3891	2897	2871	2876	DNA repair protein RadA	radA
SAUSA300_0760	1,10	0,91	0,344796549	No	43212	45552	38251	64809	68476	68200	48112	50678	41182	phosphopyruvate hydratase	eno
SAUSA300_0555	1,10	0,91	0,417982125	No	4420	4432	3765	6227	6126	5930	4923	4869	4101	putative hexulose-6-phosphate synthase	SAUSA300_0555
SAUSA300_1155	1,10	0,91	0,301606085	No	2389	2625	2592	2778	3034	2566	2814	2872	2693	putative membrane-associated zinc metall	SAUSA300_1155
SAUSA300_1272	1,10	0,91	0,512492064	No	522	441	439	388	343	303	529	487	525	conserved hypothetical protein	SAUSA300_1272
SAUSA300_1645	1,10	0,91	0,533428438	No	5933	9431	8034	9993	9548	12109	8308	9224	8244	6-phosphofructokinase	pfkA
SAUSA300_1073	1,10	0,91	0,38002145	No	14506	13912	12496	13235	12552	13352	14578	16942	13495	S-adenosyl-methyltransferase MraW	mraW
SAUSA300_1475	1,10	0,91	0,199935958	No	6149	7278	6306	7597	7873	8179	7315	7520	6870	acetyl-CoA carboxylase, biotin carboxylase	accC
SAUSA300_0134	1,10	0,91	0,661409349	No	248	145	249	90	115	95	208	232	265	polysaccharide extrusion protein	SAUSA300_0134
SAUSA300_2574	1,10	0,91	0,495290664	No	691	741	560	997	1086	1130	699	789	700	conserved hypothetical protein	SAUSA300_2574
SAUSA300_0131	1,10	0,91	0,691786867	No	55	73	71	105	95	93	48	87	85	putative Bacterial sugar transferase	SAUSA300_0131
SAUSA300_2505	1,10	0,91	0,814014536	No	129	82	172	93	230	98	122	112	189	acetyltransferase, GNAT family	SAUSA300_2505
SAUSA300_0574	1,10	0,91	0,414431967	No	1905	1752	1538	1714	1760	1911	2040	1844	1816	phosphomevalonate kinase	SAUSA300_0574

SAUSA300_1623	1,10	0,91	0,515543814	No	1066	1096	1282	743	825	589	1185	1229	1372	conserved hypothetical protein	SAUSA300_1623
SAUSA300_2500	1,10	0,91	0,692417351	No	320	387	330	703	696	868	365	426	352	glycosyl transferase	SAUSA300_2500
SAUSA300_1120	1,10	0,91	0,239678384	No	1507	1522	1349	2121	2132	1850	1626	1624	1550	ATP-dependent DNA helicase RecG	recG
SAUSA300_1908	1,10	0,91	0,260133559	No	582	560	656	463	543	410	664	636	677	conserved hypothetical protein	SAUSA300_1908
SAUSA300_1939	1,10	0,91	0,744219547	No	98	49	48	378	367	492	54	91	67	phi77 ORF015-like protein, putative protea	SAUSA300_1939
SAUSA300_2352	1,10	0,91	0,624964413	No	861	768	646	682	591	620	887	836	772	addiction module toxin, Txe/YoeB family	SAUSA300_2352
SAUSA300_1399	1,10	0,91	0,904355351	No	0	1	5	1	2	0	4	1	2	phiSLT ORF110-like protein	SAUSA300_1399
SAUSA300_1257	1,10	0,91	0,198201776	No	2263	2092	2077	2213	2272	2112	2301	2297	2445	peptide methionine sulfoxide reductase re	msrR
SAUSA300_1547	1,09	0,91	0,689739896	No	107	77	119	123	115	89	98	109	125	DNA internalization-related competence p	SAUSA300_1547
SAUSA300_2346	1,09	0,91	0,856642532	No	60	57	162	125	149	201	84	142	87	nitrite reductase [NAD(P)H	nirB
SAUSA300_1580	1,09	0,91	0,527782126	No	1321	984	827	2797	2274	2671	1239	1109	1078	bacterial luciferase family protein	SAUSA300_1580
SAUSA300_2430	1,09	0,91	0,567075889	No	245	357	281	352	340	413	352	274	343	staphylococcal tandem lipoprotein	SAUSA300_2430
SAUSA300_0919	1,09	0,91	0,470299158	No	1869	2334	2394	2522	2585	2810	2357	2454	2417	UDP-N-acetylmuramoylalanyl-D-glutamate	murE
SAUSA300_0632	1,09	0,92	0,460525153	No	699	897	712	776	649	731	935	824	767	conserved hypothetical protein	SAUSA300_0632
SAUSA300_1559	1,09	0,92	0,832391097	No	10	11	14	23	23	19	8	12	19	putative enterotoxin type A	SAUSA300_1559
SAUSA300_2370	1,09	0,92	0,778789189	No	40	25	49	24	32	37	31	45	48	putative 8-amino-7-oxononanoate synthas	SAUSA300_2370
SAUSA300_1388	1,09	0,92	0,81173877	No	26	15	15	17	20	19	25	16	19	phiSLT ORF488-like protein	SAUSA300_1388
SAUSA300_2563	1,09	0,92	0,362106716	No	744	672	621	729	793	775	793	681	744	putative transcriptional regulator	SAUSA300_2563
SAUSA300_1160	1,09	0,92	0,305617679	No	959	1014	925	961	962	843	1010	1065	1087	conserved hypothetical protein	SAUSA300_1160
SAUSA300_1528	1,09	0,92	0,264486014	No	605	697	713	778	751	725	726	731	748	cytidine deaminase	cdd
SAUSA300_1486	1,09	0,92	0,871840478	No	7	5	7	11	16	6	6	6	9	conserved hypothetical protein	SAUSA300_1486
SAUSA300_0396	1,09	0,92	0,856903831	No	12	10	8	23	9	30	15	5	13	exotoxin 7	set7
SAUSA300_1133	1,09	0,92	0,515543814	No	937	1007	852	1005	1023	1193	1037	1041	969	tRNA (guanine-N1)-methyltransferase	trmD
SAUSA300_1985	1,09	0,92	0,691971212	No	1624	2443	1488	2327	2518	2789	2109	2239	1715	serine-aspartate repeat family protein, Sdr	sdrH
SAUSA300_0050	1,09	0,92	0,900519882	No	10	5	32	4	27	6	14	13	26	conserved hypothetical protein	SAUSA300_0050
SAUSA300_0292	1,09	0,92	0,806061136	No	110	52	113	23	18	13	121	86	94	conserved hypothetical protein	SAUSA300_0292
SAUSA300_1026	1,09	0,92	0,363895255	No	14202	11234	11873	14183	14560	13887	14017	13833	12759	conserved hypothetical protein	SAUSA300_1026
SAUSA300_0669	1,09	0,92	0,337590324	No	4886	5503	4392	5787	5734	5520	5415	5748	4922	undecaprenol kinase	SAUSA300_0669
SAUSA300_2177	1,09	0,92	0,465767032	No	5737	6275	5506	5396	5587	5288	6593	6721	5752	50S ribosomal protein L17	rplQ
SAUSA300_0484	1,09	0,92	0,396820336	No	673	801	769	1273	1217	1341	834	804	808	conserved hypothetical protein	SAUSA300_0484
SAUSA300_1451	1,09	0,92	0,315877134	No	432	534	460	696	683	683	503	498	551	oxidoreductase, short-chain dehydrogenas	SAUSA300_1451
SAUSA300_1754	1,09	0,92	0,871840478	No	3	11	13	13	5	10	9	10	12	serine protease SplE	splE
SAUSA300_1597	1,09	0,92	0,531321579	No	1288	1774	1750	2203	2416	2684	1635	1843	1759	holliday junction DNA helicase RuvB	ruvB
SAUSA300_1604	1,08	0,92	0,764702501	No	155	142	211	160	176	172	183	136	235	rod shape-determining protein MreD	mreD
SAUSA300_0516	1,08	0,92	0,589510065	No	350	445	476	564	505	521	443	479	462	conserved hypothetical protein	SAUSA300_0516
SAUSA300_1951	1,08	0,92	0,909830591	No	5	0	1	7	11	12	3	2	2	phiPVL ORF052-like protein	SAUSA300_1951
SAUSA300_1514	1,08	0,92	0,807747644	No	112	93	189	66	95	59	135	121	173	ferric uptake regulation protein	fur
SAUSA300_1392	1,08	0,92	0,882217954	No	16	10	6	1	7	1	11	11	11	phiSLT ORF191-like protein	SAUSA300_1392
SAUSA300_1546	1,08	0,92	0,744219547	No	276	164	210	106	122	92	257	203	241	DNA polymerase III, delta subunit	holA
SAUSA300_0225	1,08	0,93	0,806061136	No	33	70	35	56	72	106	57	49	45	putative acyl-CoA acetyltransferase FadA	SAUSA300_0225
SAUSA300_0824	1,08	0,93	0,581807703	No	737	1002	789	1130	1066	992	934	895	908	conserved hypothetical protein	SAUSA300_0824
SAUSA300_0480	1,08	0,93	0,538065387	No	623	652	721	849	805	908	709	773	679	peptidyl-tRNA hydrolase	pth
SAUSA300_2127	1,08	0,93	0,845773634	No	72	52	72	168	212	112	63	71	80	conserved hypothetical protein	SAUSA300_2127
SAUSA300_0625	1,08	0,93	0,735798846	No	2670	1703	2541	1133	1201	741	2350	2272	2855	teichoic acid ABC transporter protein	tagG
SAUSA300_0479	1,08	0,93	0,558554642	No	38088	35872	30407	38424	39040	43148	39750	40767	32161	ribosomal protein L25, Ctc-form	SAUSA300_0479
SAUSA300_1161	1,08	0,93	0,459260664	No	885	908	917	799	834	725	1011	920	994	ribosomal protein L7Ae	SAUSA300_1161
SAUSA300_0860	1,08	0,93	0,531321579	No	596	840	760	1034	956	1041	790	819	767	Ornithine aminotransferase	rocD
SAUSA300_0086	1,08	0,93	0,709993652	No	152	145	133	212	185	201	160	171	132	conserved hypothetical protein	SAUSA300_0086
SAUSA300_1512	1,08	0,93	0,389212535	No	6044	6090	4779	7279	7215	6802	6106	6223	5893	penicillin-binding protein 3	pbp3
SAUSA300_1343	1,08	0,93	0,597414815	No	1064	1055	754	1445	1282	1346	1129	949	1015	endonuclease III	nth
SAUSA300_0909	1,08	0,93	0,498376629	No	1288	1306	1080	871	818	717	1376	1242	1336	pseudouridine synthases, RluA subfamily	SAUSA300_0909
SAUSA300_0483	1,08	0,93	0,360980133	No	2615	3276	2763	4270	4210	4367	3180	3149	2994	tetrapyrrole methylase family protein	SAUSA300_0483
SAUSA300_0650	1,08	0,93	0,35583495	No	5408	5315	4620	5398	5529	5922	5438	5814	5265	phosphate transporter family protein	SAUSA300_0650
SAUSA300_2639	1,08	0,93	0,829512625	No	52585	20831	31930	15030	20373	13062	46996	37194	29649	cold shock protein	SAUSA300_2639
SAUSA300_1010	1,08	0,93	0,700776749	No	394	308	335	265	273	238	367	421	329	conserved hypothetical protein	SAUSA300_1010
SAUSA300_1150	1,08	0,93	0,376601433	No	14053	16226	14736	18112	17655	19657	16145	16959	15361	translation elongation factor Ts	tsf
SAUSA300_0095	1,08	0,93	0,795352309	No	43	40	60	16	27	25	55	45	55	transcriptional regulator, LysR family doma	SAUSA300_0095
SAUSA300_1587	1,08	0,93	0,421729709	No	1738	2000	1792	1594	1661	1428	1913	2062	1975	histidyl-tRNA synthetase	hisS
SAUSA300_1780	1,08	0,93	0,419701169	No	589	627	714	833	895	893	709	659	715	conserved hypothetical protein	SAUSA300_1780

SAUSA300_0521	1,08	0,93	0,418131057	No	3569	3929	3713	4956	5123	5199	4073	4161	3825	transcription termination/antitermination	nusG
SAUSA300_0514	1,07	0,93	0,593269366	No	420	595	602	839	791	849	569	642	537	serine acetyltransferase	cysE
SAUSA300_1234	1,07	0,93	0,606663757	No	184	152	133	163	131	142	173	161	166	30S ribosomal protein S14-2	rpmN
SAUSA300_0976	1,07	0,93	0,422972199	No	8627	11283	8925	5778	5883	6677	10342	10485	10145	phosphoribosylamine--glycine ligase	purD
SAUSA300_0531	1,07	0,93	0,555200333	No	25076	26594	23828	23380	24126	24388	26379	29894	24818	30S ribosomal protein S7	SAUSA300_0531
SAUSA300_0894	1,07	0,93	0,876398821	No	19	16	8	23	25	13	14	14	18	oligopeptide ABC transporter, ATP-binding	oppD
SAUSA300_0215	1,07	0,93	0,85571025	No	167	105	218	46	56	28	160	129	237	conserved hypothetical protein	SAUSA300_0215
SAUSA300_0732	1,07	0,93	0,453966968	No	689	645	637	902	962	895	772	677	665	conserved hypothetical protein	SAUSA300_0732
SAUSA300_0269	1,07	0,93	0,672011838	No	188	219	172	390	363	501	203	198	219	choloylglycine hydrolase family protein	SAUSA300_0269
SAUSA300_2365	1,07	0,93	0,833554309	No	40	29	19	43	59	49	31	35	26	gamma-hemolysin component A	hlgA
SAUSA300_1725	1,07	0,93	0,670968726	No	2844	2567	1964	4086	3798	3959	3014	2413	2484	transaldolase	SAUSA300_1725
SAUSA300_0870	1,07	0,93	0,320001402	No	4079	4509	3833	5032	5261	5162	4514	4405	4395	exonuclease RexA	rexA
SAUSA300_1894	1,07	0,93	0,760706082	No	357	491	686	901	949	1239	499	525	627	nicotinate phosphoribosyltransferase	SAUSA300_1894
SAUSA300_1700	1,07	0,93	0,421639479	No	2186	2149	2303	2174	2459	1948	2355	2415	2339	polysaccharide biosynthesis protein	SAUSA300_1700
SAUSA300_1240	1,07	0,93	0,724878803	No	2740	2397	2948	1222	1427	972	2986	2787	2892	conserved hypothetical protein	SAUSA300_1240
SAUSA300_0748	1,07	0,93	0,457713432	No	773	778	881	939	780	760	832	870	904	conserved hypothetical protein	SAUSA300_0748
SAUSA300_1871	1,07	0,94	0,926542652	No	7	4	36	9	11	6	13	6	32	conserved hypothetical protein	SAUSA300_1871
SAUSA300_0900	1,07	0,94	0,865506873	No	24	16	28	52	47	28	29	17	28	putative competence protein	SAUSA300_0900
SAUSA300_0869	1,07	0,94	0,402629452	No	1767	2012	1918	2102	2236	2047	2014	1956	2123	exonuclease RexB	rexB
SAUSA300_1860	1,07	0,94	0,470239045	No	3300	3658	3415	4080	4447	4062	3665	3936	3486	aminopeptidase PepS	pepS
SAUSA300_1135	1,07	0,94	0,691837886	No	450	385	501	427	426	402	433	453	541	putative membrane protein	SAUSA300_1135
SAUSA300_1598	1,07	0,94	0,597128749	No	729	876	992	682	764	568	865	954	961	holliday junction DNA helicase RuvA	ruvA
SAUSA300_0055	1,07	0,94	0,718875865	No	93	145	114	235	237	241	121	136	123	alcohol dehydrogenase, zinc-containing	SAUSA300_0055
SAUSA300_0621	1,07	0,94	0,696819811	No	1977	1251	1568	1179	1021	977	1653	1704	1760	iron-dependent repressor	SAUSA300_0621
SAUSA300_1089	1,07	0,94	0,809352968	No	208	126	229	89	65	62	183	165	253	lipoprotein signal peptidase	lspA
SAUSA300_1921	1,07	0,94	0,602012216	No	1740	1493	1433	2427	2421	2366	1655	1804	1514	truncated amidase	SAUSA300_1921
SAUSA300_1998	1,07	0,94	0,709993652	No	656	888	740	905	751	1096	777	866	795	putative membrane protein	SAUSA300_1998
SAUSA300_0429	1,07	0,94	0,33712533	No	1245	1250	1160	1289	1310	1150	1339	1232	1319	PAP2 family protein	SAUSA300_0429
SAUSA300_2135	1,07	0,94	0,697112937	No	563	745	555	1371	1499	1658	632	710	644	iron compound ABC transporter, permeas	SAUSA300_2135
SAUSA300_1019	1,07	0,94	0,534151368	No	1071	880	805	1149	1091	1180	1032	937	958	conserved hypothetical protein	SAUSA300_1019
SAUSA300_2368	1,06	0,94	0,899768307	No	5	12	13	6	9	7	11	12	10	putative membrane protein	SAUSA300_2368
SAUSA300_0708	1,06	0,94	0,641649464	No	582	571	459	766	721	627	625	574	515	histidinol-phosphate aminotransferase	hisC
SAUSA300_1342	1,06	0,94	0,813982976	No	264	190	217	235	223	167	256	182	275	conserved hypothetical protein	SAUSA300_1342
SAUSA300_0764	1,06	0,94	0,363507267	No	5172	5573	5722	5959	6237	5949	5718	6275	5536	ribonuclease R	rnr
SAUSA300_1675	1,06	0,94	0,465767032	No	2081	1999	2155	1968	2044	1906	2013	2481	2134	tyrosyl-tRNA synthetase	tyrS
SAUSA300_0304	1,06	0,94	0,808376976	No	944	463	648	640	489	565	751	610	821	conserved hypothetical protein	SAUSA300_0304
SAUSA300_0124	1,06	0,94	0,815488324	No	78	83	99	298	361	495	90	96	92	HPCH/HPAI aldolase family protein	SAUSA300_0124
SAUSA300_0688	1,06	0,94	0,688000159	No	1414	1376	1069	1926	1772	1715	1427	1312	1351	oxidoreductase, aldo/keto reductase famil	SAUSA300_0688
SAUSA300_0532	1,06	0,94	0,643707758	No	133142	144012	127175	124954	130005	137370	138594	158620	131668	translation elongation factor G	fusA
SAUSA300_0800	1,06	0,94	0,795352309	No	632	519	617	354	412	263	589	548	739	staphylococcal enterotoxin K	sek
SAUSA300_0060	1,06	0,94	0,845773634	No	50	23	35	40	43	37	42	31	39	putative transposase	SAUSA300_0060
SAUSA300_0634	1,06	0,94	0,703428866	No	481	580	620	722	775	969	575	595	614	ferrichrome transport permease protein fl	fhuB
SAUSA300_0920	1,06	0,94	0,656910142	No	363	461	427	327	361	345	430	454	445	conserved hypothetical protein	SAUSA300_0920
SAUSA300_1168	1,06	0,95	0,38413013	No	6552	6401	6932	7218	6778	6832	6635	7512	6894	RNA-metabolising metallo-beta-lactamase	SAUSA300_1168
SAUSA300_1191	1,06	0,95	0,813823426	No	295	257	303	236	250	248	308	353	246	glycerol uptake facilitator	glpF
SAUSA300_2510	1,06	0,95	0,753372515	No	141	127	127	198	167	191	140	134	143	conserved hypothetical protein	SAUSA300_2510
SAUSA300_1523	1,06	0,95	0,549681341	No	996	1154	1003	1057	935	954	1087	1121	1127	conserved hypothetical protein	SAUSA300_1523
SAUSA300_2533	1,06	0,95	0,760706082	No	1731	2931	2157	3245	3176	3861	2459	2353	2405	pantoate--beta-alanine ligase	panC
SAUSA300_2555	1,06	0,95	0,762252151	No	105	104	128	103	110	109	113	126	120	glutathione peroxidase	SAUSA300_2555
SAUSA300_1586	1,06	0,95	0,377682784	No	5508	5634	5071	5627	5795	5473	5452	6043	5632	aspartyl-tRNA synthetase	aspS
SAUSA300_0877	1,06	0,95	0,748776661	No	1121	1228	1055	35786	29274	39656	1215	1258	1128	Chaperone clpB	clpB
SAUSA300_2463	1,06	0,95	0,935426095	No	2790	2757	9543	2541	2459	2301	2290	11336	2565	D-lactate dehydrogenase	ddh
SAUSA300_1450	1,06	0,95	0,659337651	No	2084	1936	1828	1965	1751	1752	2105	2076	1998	oxidoreductase, aldo/keto reductase famil	SAUSA300_1450
SAUSA300_0239	1,06	0,95	0,925437657	No	5	10	5	7	14	3	7	8	5	PTS system, fructose-specific enzyme II, B	SAUSA300_0239
SAUSA300_0635	1,06	0,95	0,630223664	No	1228	1440	1467	1608	1535	1912	1428	1517	1426	ferrichrome transport permease protein fl	fhuG
SAUSA300_1443	1,06	0,95	0,423938588	No	1009	1040	1048	951	1046	1000	1107	1030	1133	ribosomal large subunit pseudouridine syn	rluB
SAUSA300_1474	1,05	0,95	0,533428438	No	1142	1278	1050	1130	1219	1091	1266	1216	1177	conserved hypothetical protein	SAUSA300_1474
SAUSA300_1376	1,05	0,95	0,727509429	No	96	97	105	113	95	121	108	106	102	putative lipoprotein	SAUSA300_1376
SAUSA300_1385	1,05	0,95	0,940577881	No	2	7	1	6	5	10	4	1	5	phiSLT ORF 99-like protein	SAUSA300_1385

SAUSA300_1642	1,05	0,95	0,645305706	No	14902	14113	13507	14587	14479	13100	14923	16217	13669	D-serine/D-alanine/glycine transporter	SAUSA300_1642
SAUSA300_1358	1,05	0,95	0,578187282	No	1042	1040	1040	766	782	716	1084	1093	1112	nucleoside diphosphate kinase superfamil	ndk
SAUSA300_1810	1,05	0,95	0,764376444	No	234	281	253	183	201	143	243	281	286	IS1181, transposase	SAUSA300_1810
SAUSA300_1656	1,05	0,95	0,707244446	No	2508	2576	2217	2679	2766	3375	2590	2769	2329	universal stress protein family	SAUSA300_1656
SAUSA300_2334	1,05	0,95	0,873020447	No	21	36	32	16	27	31	34	29	31	conserved hypothetical protein	SAUSA300_2334
SAUSA300_0125	1,05	0,95	0,72766065	No	133	159	140	518	593	665	151	150	154	pyridoxal-dependent decarboxylase	SAUSA300_0125
SAUSA300_1866	1,05	0,95	0,612415194	No	2323	2294	2100	2525	2626	2215	2375	2285	2400	two-component sensor histidine kinase	vraS
SAUSA300_0855	1,05	0,95	0,538065387	No	6493	6457	6878	6148	6428	5974	6614	7144	7085	Na(+)/H(+) antiporter subunit A	mnhA
SAUSA300_1303	1,05	0,95	0,80192089	No	496	464	602	319	286	217	523	526	596	conserved hypothetical protein	SAUSA300_1303
SAUSA300_1760	1,05	0,95	0,90091204	No	16	14	33	9	20	12	22	21	24	lantibiotic epidermin immunity protein F	epiG
SAUSA300_1104	1,05	0,95	0,602192625	No	916	1094	971	1385	1330	1430	1097	1047	993	phosphopantothenoylcysteine decarboxyl	coaBC
SAUSA300_1973	1,05	0,95	0,7769807	No	131	174	112	243	203	237	150	148	140	truncated beta-hemolysin	SAUSA300_1973
SAUSA300_1334	1,05	0,95	0,883949	No	574	292	419	222	176	183	394	510	445	putative membrane protein	SAUSA300_1334
SAUSA300_1079	1,05	0,95	0,522194316	No	14140	16497	16122	14437	14441	12949	16693	16498	15922	cell division protein ftsA	ftsA
SAUSA300_0615	1,05	0,95	0,904476471	No	7	19	16	26	14	30	16	16	14	putative Na+/H+ antiporter, MnhF compo	SAUSA300_0615
SAUSA300_1527	1,05	0,95	0,61602727	No	1268	1560	1582	1608	1733	1686	1483	1514	1642	GTP-binding protein Era	era
SAUSA300_2514	1,05	0,95	0,870540736	No	29	42	26	66	65	58	27	33	42	conserved hypothetical protein	SAUSA300_2514
SAUSA300_2345	1,05	0,95	0,9212043	No	17	30	30	49	52	65	25	32	26	nitrite reductase [NAD(P)H	nirD
SAUSA300_1719	1,05	0,96	0,739597301	No	164	162	155	170	214	210	171	163	168	arsenate reductase	arsC
SAUSA300_1795	1,05	0,96	0,806035754	No	15673	11004	11014	9900	9324	7756	14552	12578	12345	conserved hypothetical protein	SAUSA300_1795
SAUSA300_1338	1,05	0,96	0,806061136	No	3171	3092	3142	3066	2815	2600	3251	3094	3505	conserved hypothetical protein	SAUSA300_1338
SAUSA300_1715	1,05	0,96	0,934377289	No	281	572	638	1171	1161	1668	511	474	588	riboflavin biosynthesis protein	ribD
SAUSA300_1689	1,05	0,96	0,768987751	No	1085	842	911	809	746	605	1029	928	1011	conserved hypothetical protein	SAUSA300_1689
SAUSA300_1462	1,05	0,96	0,643188341	No	1300	1155	995	1542	1332	1411	1242	1189	1170	conserved hypothetical protein	SAUSA300_1462
SAUSA300_0614	1,05	0,96	0,95465133	No	2	1	4	4	7	3	6	1	1	putative Na+/H+ antiporter, MnhE compo	SAUSA300_0614
SAUSA300_1735	1,05	0,96	0,777692309	No	305	465	464	730	699	874	414	432	452	O-succinylbenzoic acid synthetase	menC
SAUSA300_2400	1,05	0,96	0,712314939	No	1061	1248	1100	2336	2207	2647	1259	1207	1103	glutamyl-aminopeptidase	SAUSA300_2400
SAUSA300_1157	1,05	0,96	0,636230171	No	8660	9002	7809	9508	9683	9837	8988	8937	8697	DNA polymerase III, alpha subunit	polC
SAUSA300_0859	1,04	0,96	0,832391097	No	2373	2528	1498	5521	4091	6066	2593	1974	2120	NADH-dependent flavin oxidoreductase	SAUSA300_0859
SAUSA300_1551	1,04	0,96	0,533846746	No	1164	1235	1310	1283	1355	1355	1299	1318	1264	conserved hypothetical protein	SAUSA300_1551
SAUSA300_2301	1,04	0,96	0,806061136	No	291	379	511	455	440	418	428	397	416	teicoplanin resistance associated membra	tcaB
SAUSA300_1853	1,04	0,96	0,838806422	No	264	379	341	243	295	250	368	337	326	conserved hypothetical protein	SAUSA300_1853
SAUSA300_1529	1,04	0,96	0,86014342	No	138	133	177	117	131	84	138	149	181	diacylglycerol kinase	dgkA
SAUSA300_1782	1,04	0,96	0,720962976	No	851	1136	973	1776	1697	1635	994	1030	1066	ferrochelatase	hemH
SAUSA300_1611	1,04	0,96	0,711361733	No	5629	6925	7415	5929	6626	6549	6521	7219	7071	valyl-tRNA synthetase	valS
SAUSA300_0829	1,04	0,96	0,703932274	No	4976	6097	5108	6666	6421	7159	5986	5830	5043	lipoic acid synthetase	lipA
SAUSA300_0842	1,04	0,96	0,764487275	No	735	793	658	1525	1321	1622	834	717	727	conserved hypothetical protein	SAUSA300_0842
SAUSA300_1530	1,04	0,96	0,688106239	No	797	875	793	748	748	683	829	880	858	conserved hypothetical protein	SAUSA300_1530
SAUSA300_0049	1,04	0,96	0,911424152	No	50	23	46	23	32	12	38	37	48	hypothetical protein	SAUSA300_0049
SAUSA300_1406	1,04	0,96	0,956774959	No	0	0	1	3	0	0	1	1	0	phiSLT ORF 104b-like protein	SAUSA300_1406
SAUSA300_1448	1,04	0,96	0,672011838	No	1280	1328	1364	2960	2946	3234	1486	1350	1299	transcriptional regulator, Fur family	SAUSA300_1448
SAUSA300_0925	1,04	0,96	0,705221941	No	932	1087	993	1053	1003	983	1063	1066	1007	5' nucleotidase family protein	SAUSA300_0925
SAUSA300_0556	1,04	0,96	0,789818343	No	4494	4610	3716	8179	7835	9211	4670	4517	4125	SIS domain protein	SAUSA300_0556
SAUSA300_0922	1,04	0,96	0,836356825	No	2888	2560	1894	2665	2319	2703	2796	2619	2203	membrane protein, TerC family	SAUSA300_0922
SAUSA300_0845	1,04	0,96	0,63492123	No	2623	2237	2193	3184	3135	2972	2498	2424	2388	cytosol aminopeptidase	ampA
SAUSA300_2579	1,04	0,97	0,776476158	No	8989	9397	7458	11467	12331	9742	8619	8926	9234	N-acetylmuramoyl-L-alanine amidase dom	SAUSA300_2579
SAUSA300_2314	1,03	0,97	0,712884825	No	1528	1545	1331	1113	1120	948	1447	1544	1562	conserved hypothetical protein	SAUSA300_2314
SAUSA300_2290	1,03	0,97	0,887447921	No	126	116	100	145	198	213	121	124	109	putative 3-methyladenine DNA glycosylase	SAUSA300_2290
SAUSA300_1472	1,03	0,97	0,735798846	No	3390	3437	3535	2814	2835	2784	3559	3333	3820	exodeoxyribonuclease VII, large subunit	xseA
SAUSA300_0402	1,03	0,97	0,947481546	No	9	11	13	7	0	3	14	9	11	exotoxin	SAUSA300_0402
SAUSA300_1254	1,03	0,97	0,813823426	No	4755	4350	4037	4266	3868	3558	4518	4332	4733	putative membrane protein	SAUSA300_1254
SAUSA300_1846	1,03	0,97	0,792648551	No	1281	1236	1236	894	992	736	1176	1274	1427	conserved hypothetical protein	SAUSA300_1846
SAUSA300_1572	1,03	0,97	0,79271205	No	2050	1885	2137	1770	2056	1823	2213	2067	1999	conserved hypothetical protein	SAUSA300_1572
SAUSA300_2065	1,03	0,97	0,760706082	No	5811	6503	6392	7686	7826	8475	6493	6561	6279	UDP-N-acetylglucosamine 2-epimerase	SAUSA300_2065
SAUSA300_0211	1,03	0,97	0,913611198	No	34	45	42	49	32	19	38	44	44	maltose ABC transporter, permease protei	SAUSA300_0211
SAUSA300_0276	1,03	0,97	0,915573073	No	169	83	149	69	81	64	122	133	158	putative membrane protein	SAUSA300_0276
SAUSA300_1018	1,03	0,97	0,832391097	No	1765	1266	1499	1124	1100	849	1471	1481	1723	conserved hypothetical protein	SAUSA300_1018
SAUSA300_1579	1,03	0,97	0,793518044	No	3743	4070	3157	4611	4621	4705	3986	3639	3694	aminotransferase, class V	SAUSA300_1579
SAUSA300_0729	1,03	0,97	0,790405804	No	365	442	330	500	471	489	374	389	408	integral membrane protein	SAUSA300_0729

SAUSA300_2226	1,03	0,97	0,845773634	No	339	434	351	666	539	707	404	365	392	molybdenum cofactor biosynthesis protei	moaB
SAUSA300_0032	1,03	0,97	0,770274833	No	130433	114846	118479	99940	107248	85166	125427	131781	117747	penicillin-binding protein 2'	mecA
SAUSA300_2216	1,03	0,97	0,869477284	No	114	120	106	86	77	74	123	106	121	transcriptional regulator, MarR family	SAUSA300_2216
SAUSA300_0210	1,03	0,97	0,915738839	No	78	99	85	70	47	87	85	109	76	maltose ABC transporter, permease protei	SAUSA300_0210
SAUSA300_0430	1,03	0,97	0,732790093	No	1431	1510	1323	1989	1887	1936	1496	1547	1349	conserved hypothetical protein	SAUSA300_0430
SAUSA300_0205	1,03	0,97	0,867524812	No	239	229	201	99	106	67	238	218	231	staphylococcal tandem lipoprotein	SAUSA300_0205
SAUSA300_0330	1,03	0,97	0,881447558	No	96	162	111	152	122	135	138	128	117	putative transport protein SgaT	SAUSA300_0330
SAUSA300_0655	1,03	0,97	0,715317463	No	2449	2606	2608	3394	3415	3750	2613	2767	2512	conserved hypothetical protein	SAUSA300_0655
SAUSA300_1515	1,03	0,97	0,896228522	No	358	320	352	236	252	132	327	360	373	ABC transporter, permease protein	SAUSA300_1515
SAUSA300_0691	1,03	0,97	0,900894941	No	16719	10309	8018	10272	10136	7760	12147	11378	12537	DNA-binding response regulator SaeR	saeR
SAUSA300_1101	1,03	0,97	0,836012416	No	2937	2331	2064	2230	2171	1856	2653	2414	2467	putative fibronectin/fibrinogen binding pr	SAUSA300_1101
SAUSA300_0727	1,03	0,97	0,709993652	No	2894	3003	2574	3605	3563	3794	2975	2999	2727	peptidase T	pepT
SAUSA300_1427	1,03	0,97	0,976603125	No	0	1	2	1	0	1	1	2	2	phiSLT ORF86-like protein	SAUSA300_1427
SAUSA300_0728	1,03	0,97	0,83244665	No	372	423	326	579	631	519	390	391	370	conserved hypothetical protein	SAUSA300_0728
SAUSA300_0837	1,03	0,97	0,894941203	No	1061	891	1180	530	518	417	1100	996	1122	D-alanine-activating enzyme/D-alanine-D-a	dltC
SAUSA300_1235	1,03	0,97	0,914461587	No	355	631	292	311	273	350	488	371	453	guanosine monophosphate reductase	guaC
SAUSA300_1075	1,03	0,97	0,697112937	No	10167	9925	9504	9833	9946	9344	9922	10827	9617	penicillin-binding protein 1	pbpA
SAUSA300_1332	1,03	0,97	0,743055786	No	1883	1829	1719	1813	1889	1751	1860	1949	1758	putative 5'-3' exonuclease	SAUSA300_1332
SAUSA300_1718	1,03	0,98	0,841814831	No	276	300	243	208	228	185	297	264	276	aresenical pump membrane protein	arsB
SAUSA300_2052	1,02	0,98	0,886483911	No	251	224	187	155	178	154	223	248	206	single-stranded DNA- binding protein fami	SAUSA300_2052
SAUSA300_2509	1,02	0,98	0,872417043	No	257	226	247	223	174	206	221	243	280	transcriptional regulator, TetR family	SAUSA300_2509
SAUSA300_0832	1,02	0,98	0,892084864	No	536	526	632	483	433	350	558	562	616	conserved hypothetical protein	SAUSA300_0832
SAUSA300_2557	1,02	0,98	0,899515521	No	141	118	173	186	167	216	133	152	159	ABC transporter protein	SAUSA300_2557
SAUSA300_0492	1,02	0,98	0,814386766	No	599	697	603	988	947	1004	667	674	606	dihydropteroate synthase	folP
SAUSA300_1600	1,02	0,98	0,870540736	No	1989	2438	2617	2529	2698	2779	2401	2489	2318	GTP-binding protein Obg/CgtA	SAUSA300_1600
SAUSA300_2093	1,02	0,98	0,898781665	No	374	346	342	377	329	311	339	403	342	conserved hypothetical protein	SAUSA300_2093
SAUSA300_0171	1,02	0,98	0,911424152	No	207	151	218	140	167	120	185	200	202	cation efflux family protein	SAUSA300_0171
SAUSA300_2278	1,02	0,98	0,949360589	No	74	64	37	112	65	92	54	59	64	urocanate hydratase	hutU
SAUSA300_2246	1,02	0,98	0,911424152	No	636	517	508	470	428	447	641	536	515	conserved hypothetical protein	SAUSA300_2246
SAUSA300_1713	1,02	0,98	0,977665409	No	370	542	505	884	951	1109	489	428	535	riboflavin biosynthesis protein	ribBA
SAUSA300_0235	1,02	0,98	NA	NA	730	387	26175	261	266	281	258	27193	874	L-lactate dehydrogenase	SAUSA300_0235
SAUSA300_0133	1,02	0,98	0,946003461	No	62	77	112	62	79	50	84	79	95	putative membrane protein	SAUSA300_0133
SAUSA300_1314	1,02	0,98	0,912911647	No	1528	1659	1241	1956	1675	1718	1652	1484	1378	conserved hypothetical protein	SAUSA300_1314
SAUSA300_1080	1,02	0,98	0,836012416	No	27533	30446	29512	40086	41147	45229	29108	30766	29309	cell division protein ftsZ	ftsZ
SAUSA300_0693	1,02	0,98	0,942622669	No	5246	3710	2332	3026	2757	2237	3735	3426	4345	putative lipoprotein	SAUSA300_0693
SAUSA300_2623	1,02	0,98	0,914461587	No	372	434	371	527	575	540	379	453	368	pyrrolidone-carboxylate peptidase	pcp
SAUSA300_0854	1,02	0,98	0,911424152	No	773	771	982	624	651	470	827	866	884	Na(+)/H(+) antiporter subunit B	mnhB
SAUSA300_2329	1,02	0,98	0,864624415	No	6461	5113	6460	3897	3762	3369	6124	6154	6087	proton/sodium-glutamate symport protei	gltT
SAUSA300_1337	1,02	0,98	0,928978106	No	4213	3220	4439	2247	2191	1735	3844	3828	4418	conserved hypothetical protein	SAUSA300_1337
SAUSA300_0908	1,02	0,98	0,876398821	No	2788	3243	2388	3165	2926	3196	2893	2937	2738	NAD(+)/NADH kinase	SAUSA300_0908
SAUSA300_0297	1,02	0,98	0,96895003	No	16	16	27	4	9	7	16	23	22	putative lipoprotein	SAUSA300_0297
SAUSA300_2140	1,02	0,98	0,883199311	No	822	972	854	1257	1388	1380	842	877	970	conserved hypothetical protein	SAUSA300_2140
SAUSA300_2425	1,02	0,98	0,952979339	No	72	131	170	221	176	197	147	111	127	conserved hypothetical protein	SAUSA300_2425
SAUSA300_1649	1,01	0,99	0,85571025	No	3436	3221	3266	3102	3433	2906	3438	3238	3391	DNA polymerase III, alpha subunit	dnaE
SAUSA300_0460	1,01	0,99	0,939377293	No	1822	2443	2819	2263	2328	2617	2305	2524	2358	conserved hypothetical protein	SAUSA300_0460
SAUSA300_2347	1,01	0,99	0,987081154	No	2	7	25	17	27	21	11	16	8	nitrite reductase transcriptional regulator	nirR
SAUSA300_1716	1,01	0,99	0,946003461	No	122	175	183	233	214	268	163	160	169	conserved hypothetical protein	SAUSA300_1716
SAUSA300_1447	1,01	0,99	0,93559226	No	1378	1437	1208	2195	1902	1973	1381	1253	1434	tyrosine recombinase XerD	xerD
SAUSA300_2429	1,01	0,99	0,946147513	No	145	192	174	198	178	197	187	155	176	staphylococcal tandem lipoprotein	SAUSA300_2429
SAUSA300_2531	1,01	0,99	0,951647114	No	389	426	289	422	482	463	392	393	330	hydrolase, CocE/NonD family	SAUSA300_2531
SAUSA300_0849	1,01	0,99	0,940577881	No	1252	1118	1122	1033	1129	940	1171	1184	1175	Na(+)/H(+) antiporter subunit G	mnhG
SAUSA300_0629	1,01	0,99	0,940593258	No	2075	2116	2331	3169	3394	3366	2236	2160	2197	penicillin-binding protein 4	pbp4
SAUSA300_0682	1,01	0,99	0,958567616	No	107	130	139	183	149	186	122	136	125	ybaK/ebsC protein	ybaK
SAUSA300_2565	1,01	0,99	0,967412604	No	28277	21907	31159	15816	19769	16152	24249	31848	26050	clumping factor B	clfB
SAUSA300_2037	1,01	0,99	0,961364915	No	32489	19399	25932	21275	24820	21235	28111	26712	23752	ATP-dependent RNA helicase	SAUSA300_2037
SAUSA300_1247	1,01	0,99	0,939225314	No	944	758	775	891	875	762	870	821	804	conserved hypothetical protein	SAUSA300_1247
SAUSA300_1532	1,01	0,99	0,958567616	No	10511	11304	10359	13844	14853	15576	10759	10982	10731	conserved hypothetical protein	SAUSA300_1532
SAUSA300_1880	1,01	0,99	0,914461587	No	17201	18050	16108	18259	19219	18007	17556	17824	16440	Aspartyl/glutamyl-tRNA amidotransferase	gatB
SAUSA300_0482	1,01	0,99	0,949360589	No	1431	1577	1476	1376	1512	1281	1569	1459	1497	polysaccharide biosynthesis protein	SAUSA300_0482

SAUSA300_2038	1,01	0,99	0,938496835	No	4203	4922	4297	4690	4983	5019	4522	4880	4139	UDP-N-acetylmuramoyl-tripeptide--D-alan	murF
SAUSA300_2499	1,01	0,99	0,976603125	No	179	164	162	169	169	229	159	155	196	squalene desaturase	crtM
SAUSA300_1077	1,01	0,99	0,897090279	No	3278	3488	3416	2860	2874	2978	3268	3423	3574	UDP-N-acetylmuramoylalanine--D-glutama	murD
SAUSA300_1736	1,01	0,99	0,953468537	No	727	568	555	513	618	538	659	640	561	conserved hypothetical protein	SAUSA300_1736
SAUSA300_2227	1,01	0,99	0,950177061	No	716	861	746	1002	947	1053	799	737	807	molybdopterin biosynthesis protein B	moeB
SAUSA300_1771	1,01	0,99	0,9878257	No	127	73	177	62	106	58	109	108	163	conserved hypothetical protein	SAUSA300_1771
SAUSA300_0758	1,01	0,99	0,977488218	No	7472	10867	10254	10423	11037	12664	8879	10978	8957	triosephosphate isomerase	tpiA
SAUSA300_0835	1,01	0,99	0,950177061	No	11107	11665	9370	10385	10075	10036	11807	10348	10214	D-alanine-activating enzyme/D-alanine-D-a	dltA
SAUSA300_2498	1,01	0,99	0,979798917	No	276	337	347	624	516	801	307	308	353	squalene synthase	crtN
SAUSA300_0306	1,01	0,99	0,977488218	No	3686	2762	3269	3330	3751	3101	3172	3401	3200	branched-chain amino acid transport syste	brnQ
SAUSA300_0432	1,01	0,99	0,979650254	No	481	519	298	463	575	460	469	372	458	sodium dependent transporter	SAUSA300_0432
SAUSA300_2405	1,01	0,99	0,990044321	No	229	168	352	109	178	92	236	222	297	putative membrane protein	SAUSA300_2405
SAUSA300_1984	1,01	0,99	0,984053314	No	496	428	491	318	385	235	479	465	478	putative membrane protein	SAUSA300_1984
SAUSA300_2342	1,01	0,99	0,990044321	No	29	40	40	63	36	61	39	45	27	respiratory nitrate reductase, beta subunit	narH
SAUSA300_2134	1,01	0,99	0,961364915	No	520	620	524	878	886	871	546	565	562	iron compound ABC transporter, permease	SAUSA300_2134
SAUSA300_1737	1,01	1,00	0,977249124	No	512	734	643	980	829	994	629	647	627	O-succinylbenzoate-CoA ligase	menE
SAUSA300_2105	1,00	1,00	0,977488218	No	425	301	315	375	347	334	368	361	312	PTS system, mannitol specific IIBC compor	mtlF
SAUSA300_2082	1,00	1,00	0,96895003	No	3303	3339	3559	2913	2883	2572	3660	3383	3209	DNA-directed RNA polymerase, delta subu	rpoE
SAUSA300_1476	1,00	1,00	0,967185005	No	2840	3110	2736	3116	3022	3410	2994	2948	2785	acetyl-CoA carboxylase, biotin carboxyl car	accB
SAUSA300_1220	1,00	1,00	0,981936026	No	332	313	288	241	183	180	322	290	325	DNA-binding response regulator, LuxR fam	SAUSA300_1220
SAUSA300_0645	1,00	1,00	0,984541484	No	570	609	510	430	381	321	597	526	571	DNA-binding response regulator	SAUSA300_0645
SAUSA300_2511	1,00	1,00	0,989209244	No	184	216	147	284	275	287	207	165	175	conserved hypothetical protein	SAUSA300_2511
SAUSA300_0921	1,00	1,00	0,979316119	No	3937	4222	4292	3918	3852	3967	4049	4271	4171	peptide chain release factor 3	prfC
SAUSA300_0303	1,00	1,00	0,996378838	No	455	162	355	96	86	53	319	231	421	putative lipoprotein	SAUSA300_0303
SAUSA300_0179	1,00	1,00	0,994420996	No	186	193	142	238	151	195	163	161	197	putative D-isomer specific 2-hydroxyacid c	SAUSA300_0179
SAUSA300_1381	1,00	1,00	0,99139356	No	184	205	209	202	174	152	197	185	219	Panton-Valentine leukocidin, LukF-PV	lukF-PV
SAUSA300_1426	1,00	1,00	0,999461939	No	0	1	0	0	2	1	1	1	0	conserved hypothetical phage protein	SAUSA300_1426
SAUSA300_2638	1,00	1,00	0,996686812	No	26	22	28	26	20	10	24	30	22	conserved hypothetical protein	SAUSA300_2638
SAUSA300_0901	1,00	1,00	0,998800113	No	17	7	15	13	16	12	14	8	16	putative competence protein	SAUSA300_0901
SAUSA300_0503	1,00	1,00	0,996378838	No	109	146	93	103	65	78	129	91	128	transcriptional regulator, gntR family prote	SAUSA300_0503
SAUSA300_0321	1,00	1,00	0,996378838	No	847	820	755	692	766	612	804	849	771	conserved hypothetical protein	SAUSA300_0321
SAUSA300_2039	1,00	1,00	0,991230365	No	3980	4294	4075	4351	4287	4265	4071	4363	3934	D-alanine--D-alanine ligase	ddl
SAUSA300_1083	1,00	1,00	0,995174438	No	7468	6491	6621	6870	7188	7058	6988	7014	6591	conserved hypothetical protein	SAUSA300_1083
SAUSA300_2426	1,00	1,00	0,998754123	No	95	110	128	82	88	74	113	105	117	conserved hypothetical protein	SAUSA300_2426
SAUSA300_0041	1,00	1,00	1	No	40	25	53	63	72	55	40	28	50	conserved hypothetical protein	SAUSA300_0041
SAUSA300_1746	1,00	1,00	1	No	2	3	8	0	0	0	2	4	7	conserved hypothetical protein	SAUSA300_1746
SAUSA300_0052	1,00	1,00	1	No	0	0	0	1	0	0	0	0	0	hypothetical protein	SAUSA300_0052
SAUSA300_0578	1,00	1,00	1	No	0	0	0	0	0	0	0	0	0	conserved hypothetical protein	SAUSA300_0578
SAUSA300_0609	1,00	1,00	1	No	0	0	0	3	5	7	0	0	0	phage integrase family protein	SAUSA300_0609
SAUSA300_0610	1,00	1,00	1	No	0	0	0	10	2	16	0	0	0	putative Na+/H+ antiporter, MnhA compo	SAUSA300_0610
SAUSA300_1422	1,00	1,00	1	No	0	0	0	0	0	0	0	0	0	phiSLT ORF65-like protein	SAUSA300_1422
SAUSA300_1663	1,00	1,00	1	No	0	0	0	0	0	0	0	0	0	conserved hypothetical protein	SAUSA300_1663
SAUSA300_1676	1,00	1,00	0,99561498	No	351	300	323	238	250	158	315	334	322	probable transglycosylase	sgtA
SAUSA300_0759	1,00	1,00	0,996378838	No	16786	22898	20543	20777	22350	26025	19737	22133	18268	2,3-bisphosphoglycerate-independent pho	gpmI
SAUSA300_0534	1,00	1,00	0,991652897	No	1803	1719	1358	2843	2851	3252	1600	1646	1619	amidohydrolase	SAUSA300_0534
SAUSA300_2451	1,00	1,00	0,993826758	No	109	125	120	87	95	74	113	117	124	drug transporter	SAUSA300_2451
SAUSA300_0662	1,00	1,00	0,993826758	No	41	56	46	39	23	30	55	42	47	acetyltransferase, GNAT family	SAUSA300_0662
SAUSA300_0538	1,00	1,00	0,984053314	No	1400	1399	1262	1141	1050	1177	1208	1691	1148	NAD dependent epimerase/dehydratase fa	SAUSA300_0538
SAUSA300_0856	1,00	1,00	0,984053314	No	229	249	215	243	248	265	244	244	203	conserved hypothetical protein	SAUSA300_0856
SAUSA300_0750	1,00	1,00	0,967412604	No	2585	2497	2328	2824	2687	2610	2589	2525	2264	conserved hypothetical protein	SAUSA300_0750
SAUSA300_1012	1,00	1,00	0,990005824	No	226	285	453	202	196	167	263	302	398	conserved hypothetical protein	SAUSA300_1012
SAUSA300_1156	1,00	1,00	0,966742881	No	5338	5907	5822	7656	8114	7644	5592	5978	5425	prolyl-tRNA synthetase	proS
SAUSA300_0778	1,00	1,01	0,979798917	No	301	214	250	130	122	112	224	266	267	conserved hypothetical protein	SAUSA300_0778
SAUSA300_0852	0,99	1,01	0,960405386	No	5384	5338	5044	5120	5540	4958	5173	5458	5043	Na(+)/H(+) antiporter subunit D	mnhD
SAUSA300_0589	0,99	1,01	0,959101411	No	808	923	856	1222	1210	1230	875	860	840	aldo/keto reductase family protein	SAUSA300_0589
SAUSA300_0848	0,99	1,01	0,984541484	No	69	56	46	64	56	64	44	78	46	conserved hypothetical protein	SAUSA300_0848
SAUSA300_0003	0,99	1,01	0,952674804	No	927	875	916	985	962	976	929	973	799	conserved hypothetical protein	SAUSA300_0003
SAUSA300_1531	0,99	1,01	0,929737601	No	1474	1484	1362	1605	1589	1557	1414	1496	1374	phosphate starvation-induced protein, Pho	phoH
SAUSA300_1790	0,99	1,01	0,962318075	No	3264	2977	3356	2520	2455	2047	3188	3100	3225	foldase protein PrsA precursor	prsA

SAUSA300_0757	0,99	1,01	0,977488218	No	7663	10736	12430	8477	8684	9143	9196	12217	9144	phosphoglycerate kinase	pgk
SAUSA300_0989	0,99	1,01	0,928605306	No	17568	15788	15096	14889	14707	13078	15726	17075	15192	conserved hypothetical protein	SAUSA300_0989
SAUSA300_0559	0,99	1,01	0,954043379	No	238	188	212	103	169	96	211	209	208	putative substrate--CoA ligase	SAUSA300_0559
SAUSA300_1647	0,99	1,01	0,894969918	No	3161	3204	2921	3646	3915	3697	3173	3045	2968	acetyl-CoA carboxylase, carboxyl transfera	accD
SAUSA300_0258	0,99	1,01	0,941425445	No	1609	1248	1324	970	1046	846	1373	1417	1340	transcriptional regulator, GntR family	SAUSA300_0258
SAUSA300_1738	0,99	1,01	0,967185005	No	62	51	56	43	52	27	57	58	52	putative lipoprotein	SAUSA300_1738
SAUSA300_0028	0,99	1,01	0,941425445	No	403	315	305	266	279	257	330	345	329	putative transposase	SAUSA300_0028
SAUSA300_0692	0,99	1,01	0,954705062	No	5320	3055	2422	3901	3868	3385	3590	3308	3748	conserved hypothetical protein	SAUSA300_0692
SAUSA300_1533	0,99	1,01	0,900519882	No	9645	10511	9240	13739	14468	15422	9519	10248	9212	conserved hypothetical protein	SAUSA300_1533
SAUSA300_1454	0,99	1,01	0,890570662	No	8970	8837	7426	9109	8756	8229	8452	7746	8665	glucose-6-phosphate 1-dehydrogenase	zwf
SAUSA300_0872	0,99	1,01	0,930937518	No	336	335	380	327	473	426	352	318	367	conserved hypothetical protein	SAUSA300_0872
SAUSA300_1262	0,99	1,01	0,960405386	No	69	79	53	60	56	49	47	70	78	anthranilate synthase component I	trpE
SAUSA300_0984	0,99	1,01	0,875958358	No	45888	44884	37544	42486	38452	38072	43130	44971	38326	phosphoenolpyruvate-protein phosphotra	ptsI
SAUSA300_1912	0,99	1,01	0,97593606	No	26	31	39	13	36	24	37	25	33	putative membrane protein	SAUSA300_1912
SAUSA300_0523	0,99	1,02	0,904355351	No	22266	20411	21527	22283	24392	24379	20759	22799	19690	ribosomal protein L1	rplA
SAUSA300_1933	0,98	1,02	0,981368559	No	12	3	4	27	25	34	8	5	4	hypothetical phage protein	SAUSA300_1933
SAUSA300_1575	0,98	1,02	0,882217954	No	4073	4968	4765	4478	4571	4957	4364	4861	4370	alanyl-tRNA synthetase	alaS
SAUSA300_1770	0,98	1,02	0,967412604	No	12	27	28	16	7	16	19	25	23	conserved hypothetical protein	SAUSA300_1770
SAUSA300_1444	0,98	1,02	0,900317435	No	512	419	525	344	334	315	459	478	494	segregation and condensation protein B	scpB
SAUSA300_2208	0,98	1,02	0,882233965	No	2682	2202	2262	2187	2391	2055	2297	2511	2214	DNA topoisomerase III	topB
SAUSA300_1323	0,98	1,02	0,928605306	No	475	701	534	647	660	512	607	577	500	conserved hypothetical protein	SAUSA300_1323
SAUSA300_0644	0,98	1,02	0,898781665	No	1018	1054	950	822	879	688	902	943	1122	conserved hypothetical protein	SAUSA300_0644
SAUSA300_2341	0,98	1,02	0,979650254	No	3	8	7	14	5	7	8	6	5	respiratory nitrate reductase, delta subuni	narJ
SAUSA300_0710	0,98	1,02	0,945638863	No	81	73	51	113	160	114	72	67	60	hypothetical protein	SAUSA300_0710
SAUSA300_0040	0,98	1,02	0,967185005	No	24	18	15	24	41	22	21	17	18	conserved hypothetical protein	SAUSA300_0040
SAUSA300_1302	0,98	1,02	0,889705046	No	1107	1295	1043	1217	1244	1072	1151	1090	1137	ATPase family protein	SAUSA300_1302
SAUSA300_1702	0,98	1,02	0,882217954	No	663	730	575	871	976	920	590	694	643	cell wall surface anchor family protein	SAUSA300_1702
SAUSA300_2267	0,98	1,02	0,84456403	No	797	980	886	1207	1181	1176	802	907	905	hydrolase, haloacid dehalogenase-like fam	SAUSA300_2267
SAUSA300_0212	0,98	1,02	0,934377289	No	67	112	123	119	131	174	97	98	104	oxidoreductase, Gfo/Idh/MocA family	SAUSA300_0212
SAUSA300_1199	0,98	1,02	0,829374655	No	1102	1112	1079	1292	1208	1187	1118	1036	1071	putative aluminium resistance protein	SAUSA300_1199
SAUSA300_1359	0,98	1,02	0,748241121	No	3030	3356	3176	3151	3192	3107	3047	3224	3103	polyprenyl synthetase	SAUSA300_1359
SAUSA300_1797	0,98	1,02	0,871840478	No	3567	2755	2937	1550	1571	1227	2984	3035	3044	conserved hypothetical protein	SAUSA300_1797
SAUSA300_1167	0,98	1,02	0,83763366	No	9626	10002	11159	11762	12759	12442	9821	10780	9567	polyribopolyribonucleotide nucleotidyltra	pnpA
SAUSA300_1442	0,98	1,02	0,935207787	No	1178	1155	1945	1171	1163	1253	1261	1649	1283	staphylococcal respiratory response prote	srrA
SAUSA300_2279	0,98	1,02	0,937751231	No	100	116	77	87	81	58	86	102	97	LysR family regulatory protein	SAUSA300_2279
SAUSA300_1578	0,98	1,02	0,85571025	No	4535	4764	3960	4899	5125	4779	4468	4239	4256	tRNA (5-methylaminomethyl-2-thiouridyla	trmU
SAUSA300_2209	0,98	1,02	0,884461561	No	1242	1252	1064	885	863	621	1158	1142	1177	conserved hypothetical protein	SAUSA300_2209
SAUSA300_2641	0,98	1,02	0,941852027	No	31	25	48	42	56	52	32	35	36	conserved hypothetical protein	SAUSA300_2641
SAUSA300_0550	0,98	1,02	0,911424152	No	1371	986	1282	617	591	408	1103	1238	1212	glycosyl transferase, group 1 family protein	SAUSA300_0550
SAUSA300_0916	0,98	1,02	0,839991188	No	1893	1725	1506	1309	1228	1254	1640	1771	1588	conserved hypothetical protein	SAUSA300_0916
SAUSA300_1396	0,98	1,02	0,930937518	No	45	68	53	70	88	109	60	49	53	phiSLT ORF151-like protein, major tail prot	SAUSA300_1396
SAUSA300_0779	0,98	1,02	0,900894941	No	229	162	177	84	88	71	184	175	192	conserved hypothetical protein	SAUSA300_0779
SAUSA300_0865	0,98	1,02	0,836012416	No	14295	14099	11719	14534	13046	12741	14132	12954	12061	glucose-6-phosphate isomerase	pgi
SAUSA300_1488	0,98	1,02	0,954897743	No	22	8	18	13	25	12	16	18	13	conserved hypothetical protein	SAUSA300_1488
SAUSA300_1852	0,98	1,03	0,832391097	No	1340	1452	1360	1266	1251	1053	1317	1372	1363	putative ABC transporter, ATP-binding pro	SAUSA300_1852
SAUSA300_0725	0,98	1,03	0,907511802	No	1678	1280	1593	735	708	592	1625	1406	1404	conserved hypothetical protein	SAUSA300_0725
SAUSA300_2066	0,98	1,03	0,856494173	No	2304	2896	2892	3383	3516	3885	2749	2605	2544	uracil phosphoribosyltransferase	upp
SAUSA300_1132	0,97	1,03	0,866470112	No	391	364	315	186	185	209	377	329	333	16S rRNA processing protein RimM	rimM
SAUSA300_1473	0,97	1,03	0,899515521	No	1502	1235	1710	541	789	512	1367	1311	1653	transcription antitermination factor NusB	nusB
SAUSA300_1650	0,97	1,03	0,745973295	No	1311	1362	1168	1448	1521	1498	1193	1345	1199	conserved hypothetical protein	SAUSA300_1650
SAUSA300_2031	0,97	1,03	0,896229982	No	103	151	155	84	61	65	118	128	155	conserved hypothetical protein	SAUSA300_2031
SAUSA300_0836	0,97	1,03	0,866243433	No	3419	2836	3539	2148	2189	1989	3198	2861	3476	dltB protein	dltB
SAUSA300_2029	0,97	1,03	0,806061136	No	463	527	461	557	559	500	483	451	478	conserved hypothetical protein	SAUSA300_2029
SAUSA300_0735	0,97	1,03	0,940593258	No	36	25	29	9	11	15	30	30	27	competence protein F	SAUSA300_0735
SAUSA300_1525	0,97	1,03	0,754776506	No	14483	13716	12493	13284	12741	12009	12824	14314	12444	glycyl-tRNA synthetase	glyS
SAUSA300_0788	0,97	1,03	0,872417043	No	1095	1181	953	1552	1251	1698	992	974	1173	nitroreductase family protein	SAUSA300_0788
SAUSA300_2310	0,97	1,03	0,967214989	No	22	12	51	6	16	12	17	17	49	conserved hypothetical protein	SAUSA300_2310
SAUSA300_0278	0,97	1,03	0,92328153	No	23574	8865	7963	8053	7891	8143	13395	12595	13241	conserved hypothetical protein	SAUSA300_0278
SAUSA300_0938	0,97	1,03	0,936373778	No	71	36	93	21	25	41	54	56	83	conserved hypothetical protein	SAUSA300_0938

SAUSA300_2548	0,97	1,03	0,833554309	No	295	249	252	243	214	234	257	243	269	conserved hypothetical protein	SAUSA300_2548
SAUSA300_2234	0,97	1,03	0,876473534	No	165	259	224	259	257	313	211	215	206	Inosine-uridine preferring nucleoside hydr	SAUSA300_2234
SAUSA300_0172	0,97	1,03	0,936373778	No	115	68	69	24	34	35	95	89	60	conserved hypothetical protein	SAUSA300_0172
SAUSA300_1906	0,97	1,03	0,869303845	No	1326	1102	1497	779	744	544	1174	1193	1438	conserved hypothetical protein	SAUSA300_1906
SAUSA300_0714	0,97	1,03	0,832391097	No	701	574	660	359	379	272	635	601	638	Integral membrane protein	SAUSA300_0714
SAUSA300_1176	0,97	1,03	0,801932106	No	1397	1270	1399	995	1149	844	1311	1305	1323	CDP-diacylglycerol--glycerol-3-phosphate 3	pgsA
SAUSA300_1244	0,97	1,03	0,891532386	No	7131	5386	8487	1454	1805	1170	6733	6409	7194	large conductance mechanosensitive chan	mscL
SAUSA300_2372	0,97	1,03	0,928744692	No	22	31	46	32	50	33	34	41	23	adenosylmethionine-8-amino-7-oxononan	bioA
SAUSA300_1505	0,97	1,03	0,698043406	No	1605	1656	1687	1432	1594	1510	1660	1510	1626	conserved hypothetical protein	SAUSA300_1505
SAUSA300_1382	0,97	1,03	0,89462938	No	179	85	119	170	151	163	118	111	138	Panton-Valentine leukocidin, LukS-PV	lukS-PV
SAUSA300_0109	0,97	1,03	0,961328734	No	5	1	7	1	2	1	4	4	5	integral membrane domain protein	SAUSA300_0109
SAUSA300_0944	0,97	1,03	0,689230567	No	1948	1926	1962	1233	1285	1171	1735	1831	2075	1,4-dihydroxy-2-naphthoate octaprenyltra	menA
SAUSA300_2591	0,97	1,03	0,90091204	No	65	60	47	32	36	25	54	59	52	conserved hypothetical protein	SAUSA300_2591
SAUSA300_0033	0,97	1,03	0,911424152	No	4692	3136	5965	1448	1878	1056	3806	4185	5323	methicillin-resistance MecR1 regulatory pr	SAUSA300_0033
SAUSA300_0876	0,97	1,03	0,865506873	No	160	134	160	175	171	152	162	124	152	putative membrane protein	SAUSA300_0876
SAUSA300_1361	0,97	1,04	0,808615287	No	484	498	623	427	482	402	557	496	502	conserved hypothetical protein	SAUSA300_1361
SAUSA300_0376	0,97	1,04	0,881174427	No	326	193	310	116	97	53	241	265	291	conserved hypothetical protein	SAUSA300_0376
SAUSA300_0002	0,96	1,04	0,662113911	No	6371	6175	5579	5148	5362	4732	6008	5734	5728	DNA polymerase III, beta subunit	dnaN
SAUSA300_0379	0,96	1,04	0,797591968	No	27316	26164	19988	161751	165759	173600	23046	23661	24099	alkyl hydroperoxide reductase, subunit F	ahpF
SAUSA300_1516	0,96	1,04	0,88832908	No	277	186	316	142	185	111	257	236	257	ABC transporter, ATP-binding protein	SAUSA300_1516
SAUSA300_1941	0,96	1,04	0,912911647	No	157	64	75	547	406	478	69	122	91	phi77 ORF003-like protein, phage termina	SAUSA300_1941
SAUSA300_2157	0,96	1,04	0,788693858	No	518	549	450	581	548	596	514	504	444	NAD-dependent deacetylase	SAUSA300_2157
SAUSA300_0983	0,96	1,04	0,713186401	No	7652	7396	6222	5809	5698	5139	7349	7284	5844	phosphocarrier protein HPr	ptsH
SAUSA300_1085	0,96	1,04	0,821888671	No	1860	1677	1708	859	978	587	1584	1614	1847	conserved hypothetical protein	SAUSA300_1085
SAUSA300_1767	0,96	1,04	0,934377289	No	21	8	28	10	7	7	24	14	19	lantibiotic epidermin biosynthesis protein	epiA
SAUSA300_0361	0,96	1,04	0,87385365	No	227	407	350	392	347	467	304	330	315	ParB-like partition protein	SAUSA300_0361
SAUSA300_1881	0,96	1,04	0,560792364	No	11381	11449	10973	9488	10028	8954	11039	10983	10489	Aspartyl/glutamyl-tRNA amidotransferase	gatA
SAUSA300_0182	0,96	1,04	0,775741842	No	971	754	753	580	548	507	786	739	852	4'-phosphopantetheinyl transferase super	SAUSA300_0182
SAUSA300_0305	0,96	1,04	NA	NA	62	68	1663	46	43	58	89	1444	143	formate/nitrite transporter family protein	SAUSA300_0305
SAUSA300_1278	0,96	1,04	0,67132604	No	5749	5315	4656	5677	5186	4773	5157	4785	5134	oligoendopeptidase F	pepF
SAUSA300_1164	0,96	1,04	0,755179118	No	632	725	658	730	622	627	614	666	654	tRNA pseudouridine synthase B	truB
SAUSA300_0039	0,96	1,04	0,764487275	No	146	152	128	182	165	180	147	132	129	conserved hypothetical protein	SAUSA300_0039
SAUSA300_1360	0,96	1,04	0,714956642	No	1299	1508	1644	1509	1470	1667	1417	1408	1447	menaquinone biosynthesis methyltransfer	ubiE
SAUSA300_1721	0,96	1,04	0,814386766	No	436	411	412	242	239	152	386	359	459	conserved hypothetical protein	SAUSA300_1721
SAUSA300_0617	0,96	1,04	0,758309074	No	2863	2501	2016	3375	3104	2709	2637	2192	2237	Na+/H+ antiporter	SAUSA300_0617
SAUSA300_1245	0,96	1,04	0,745629675	No	10041	11968	12421	12565	12678	13396	11042	10742	11204	glycine betaine transporter opuD	opuD
SAUSA300_1705	0,96	1,04	0,741353032	No	622	530	557	760	721	685	571	557	505	putative drug transporter	SAUSA300_1705
SAUSA300_2138	0,96	1,04	0,734668701	No	1033	971	830	2373	2926	2949	879	893	935	conserved hypothetical protein	SAUSA300_2138
SAUSA300_0990	0,96	1,04	0,757153613	No	1402	1103	1274	528	552	464	1151	1158	1306	conserved hypothetical protein	SAUSA300_0990
SAUSA300_0593	0,96	1,04	0,608745322	No	1509	1455	1385	1720	1508	1767	1435	1287	1437	conserved hypothetical protein	SAUSA300_0593
SAUSA300_1304	0,96	1,04	0,691333737	No	2091	2259	1929	2831	2558	2777	2074	2033	1902	conserved hypothetical protein	SAUSA300_1304
SAUSA300_0208	0,96	1,05	0,891648815	No	34	68	49	37	25	40	47	55	44	putative maltose ABC transporter, ATP-bin	SAUSA300_0208
SAUSA300_1626	0,96	1,05	0,697112937	No	8131	6674	6727	6599	6095	6385	7098	7333	6143	50S ribosomal protein L35	rpmI
SAUSA300_0260	0,96	1,05	0,816400691	No	214	352	273	342	327	442	259	274	270	6-phospho-beta-glucosidase	bglA
SAUSA300_1714	0,95	1,05	0,940577881	No	191	267	270	455	419	529	229	216	244	riboflavin synthase, alpha subunit	ribE
SAUSA300_1570	0,95	1,05	0,567075889	No	2151	2250	2053	2114	2074	1970	2135	1986	2038	peptidase, U32 family	SAUSA300_1570
SAUSA300_1506	0,95	1,05	0,712314939	No	424	515	592	443	559	504	456	481	529	conserved hypothetical protein	SAUSA300_1506
SAUSA300_1730	0,95	1,05	0,736372792	No	7647	7934	6989	8973	8299	9009	7278	7373	6870	S-adenosylmethionine synthetase	metK
SAUSA300_1397	0,95	1,05	0,888864407	No	31	25	18	60	41	40	27	22	20	phiSLT ORF213-like protein, major tail prot	SAUSA300_1397
SAUSA300_1082	0,95	1,05	0,451866501	No	4440	4038	4283	3274	3338	2994	4121	4088	3948	conserved hypothetical protein	SAUSA300_1082
SAUSA300_2050	0,95	1,05	0,871840478	No	41	62	39	77	86	62	40	38	56	TENA/THI-4 family protein	SAUSA300_2050
SAUSA300_0380	0,95	1,05	0,697112937	No	14468	13311	10527	74606	75623	80851	11714	12085	12654	Alkyl hydroperoxide reductase subunit C	ahpC
SAUSA300_1679	0,95	1,05	0,809697908	No	136	119	124	183	171	149	108	139	113	acetyl-coenzyme A synthetase	acsA
SAUSA300_0787	0,95	1,05	0,709993652	No	239	315	254	262	257	247	266	233	271	3-dehydroquinate dehydratase, type I	aroD
SAUSA300_2284	0,95	1,05	0,599781995	No	684	634	599	779	746	762	648	613	558	conserved hypothetical protein	SAUSA300_2284
SAUSA300_0056	0,95	1,05	0,947714428	No	7	3	0	4	5	9	2	4	3	conserved hypothetical protein	SAUSA300_0056
SAUSA300_2621	0,95	1,05	0,735037065	No	198	229	175	241	232	237	211	202	159	conserved hypothetical protein	SAUSA300_2621
SAUSA300_1383	0,95	1,05	0,904355351	No	14	15	11	23	20	46	13	11	13	phiSLT ORF484-like protein, lysin	SAUSA300_1383
SAUSA300_0130	0,95	1,05	0,836356825	No	45	51	71	36	41	22	47	51	61	NAD-dependent epimerase/dehydratase f	SAUSA300_0130

SAUSA300_0539	0,95	1,05	0,551400719	No	4585	4970	3900	6399	6313	6542	4412	4320	4033	branched-chain amino acid aminotransfer	ilvE
SAUSA300_0181	0,95	1,05	0,679363974	No	5763	5753	5320	6784	6931	6758	5529	4994	5447	non-ribosomal peptide synthetase	SAUSA300_0181
SAUSA300_0622	0,95	1,05	0,759327648	No	186	137	131	149	171	186	157	128	141	putative membrane protein	SAUSA300_0622
SAUSA300_1364	0,95	1,05	0,450487235	No	5997	5833	6009	5495	5966	5508	5599	5794	5531	GTP-binding protein EngA	engA
SAUSA300_2330	0,95	1,05	0,832391097	No	351	238	421	113	126	81	331	298	328	conserved hypothetical protein	SAUSA300_2330
SAUSA300_0756	0,95	1,05	0,865506873	No	38060	43886	51962	44688	46667	52473	36563	54075	36106	glyceraldehyde-3-phosphate dehydrogena	gap
SAUSA300_0623	0,95	1,05	0,57785398	No	2137	1878	1716	1700	1612	1837	1807	1872	1748	teichoic acid biosynthesis protein	tagA
SAUSA300_0312	0,95	1,05	0,882217954	No	21	27	22	36	41	59	21	29	18	indigoidine synthase family protein	SAUSA300_0312
SAUSA300_1413	0,95	1,06	0,928605306	No	9	8	6	4	9	0	6	6	9	conserved hypothetical phage protein	SAUSA300_1413
SAUSA300_1568	0,95	1,06	0,46287105	No	3675	3374	3448	3645	3762	3417	3234	3407	3294	uridine kinase	udk
SAUSA300_0496	0,95	1,06	0,45968966	No	13095	12852	12638	14179	15419	13508	11824	12867	11809	lysyl-tRNA synthetase	lysS
SAUSA300_0722	0,95	1,06	0,459129504	No	1266	1376	1261	1475	1555	1653	1196	1275	1222	UDP-N-acetylenolpyruvoylglucosamine re	murB
SAUSA300_2128	0,95	1,06	0,501103478	No	501	467	452	623	665	584	453	445	441	putative drug transporter	SAUSA300_2128
SAUSA300_2420	0,95	1,06	0,760706082	No	119	99	114	122	110	126	115	88	110	conserved hypothetical protein	SAUSA300_2420
SAUSA300_2079	0,95	1,06	0,672236544	No	53555	45629	42197	45767	42675	44766	47140	50454	35980	fructose bisphosphate aldolase	fba
SAUSA300_0850	0,95	1,06	0,670650032	No	587	569	554	530	579	540	560	557	498	Na(+)/H(+) antiporter subunit F	mnhF
SAUSA300_0907	0,94	1,06	0,696819811	No	1521	1989	1488	1919	1736	1983	1504	1642	1574	GTP pyrophosphokinase	SAUSA300_0907
SAUSA300_1537	0,94	1,06	0,555966777	No	780	887	875	851	967	933	812	832	762	conserved hypothetical protein	SAUSA300_1537
SAUSA300_1445	0,94	1,06	0,727509429	No	801	699	777	397	449	313	764	667	718	segregation and condensation protein A	scpA
SAUSA300_0132	0,94	1,06	0,703428866	No	176	186	190	149	144	139	168	170	183	glycosyl transferase, group 1 family protei	SAUSA300_0132
SAUSA300_1548	0,94	1,06	0,639197659	No	813	850	723	1260	1041	1149	747	760	744	ComE operon protein 2	SAUSA300_1548
SAUSA300_2344	0,94	1,06	0,853918441	No	86	134	139	137	126	167	105	126	110	uroporphyrin-III C-methyl transferase	SAUSA300_2344
SAUSA300_1169	0,94	1,06	0,243797067	No	5716	5694	5300	6470	6491	6496	5281	5195	5286	DNA translocase FtsK	ftsK
SAUSA300_1940	0,94	1,06	0,822979202	No	162	83	85	337	302	302	80	109	117	phage portal protein	SAUSA300_1940
SAUSA300_1222	0,94	1,06	0,6937318	No	181	167	191	129	158	161	179	155	174	thermonuclease	nuc
SAUSA300_1938	0,94	1,06	0,870540736	No	181	64	88	533	480	608	65	132	112	phi77 ORF006-like protein, putative capsic	SAUSA300_1938
SAUSA300_0755	0,94	1,06	0,902920405	No	19786	21863	33063	18136	17549	22091	17753	34544	17651	glycolytic operon regulator	SAUSA300_0755
SAUSA300_2047	0,94	1,06	0,784886273	No	72	85	71	146	110	164	72	76	68	thiamine-phosphate pyrophosphorylase	thiE
SAUSA300_1459	0,94	1,06	0,173334171	No	12268	12844	12784	12917	12345	12800	11983	11943	11757	6-phosphogluconate dehydrogenase, deca	gnd
SAUSA300_2218	0,94	1,06	0,6937318	No	704	523	540	388	370	288	603	536	519	staphylococcal accessory regulator	SAUSA300_2218
SAUSA300_0689	0,94	1,06	0,582576433	No	1671	1597	1486	1800	1609	1525	1525	1424	1519	glycosyl transferase, group 2 family protei	SAUSA300_0689
SAUSA300_1072	0,94	1,06	0,496303838	No	7933	6583	7130	5436	5150	4884	7034	7141	6177	protein mraZ	mraZ
SAUSA300_0316	0,94	1,06	0,871778377	No	31	67	49	70	52	56	39	49	51	ROK family protein	SAUSA300_0316
SAUSA300_2110	0,94	1,06	0,745629675	No	744	1075	553	841	820	969	702	706	818	truncated FmtB protein	fmtB
SAUSA300_0838	0,94	1,06	0,475206552	No	13073	11229	11710	8558	8865	7113	11361	11181	11314	D-alanine-activating enzyme/D-alanine-D-a	dltD
SAUSA300_0939	0,94	1,06	0,748776661	No	1347	1274	1728	607	676	500	1267	1342	1479	glycosyl transferase, group 1 family protei	SAUSA300_0939
SAUSA300_2109	0,94	1,06	0,658866423	No	1323	1421	876	1442	1456	1587	1171	1104	1120	truncated FmtB protein	fmtB
SAUSA300_0162	0,94	1,06	0,884461561	No	22	22	21	16	16	9	24	15	21	capsular polysaccharide biosynthesis prot	cap5K
SAUSA300_2280	0,94	1,06	0,850657396	No	28	25	25	14	23	12	22	24	25	metallothiol transferase fosB	fosB
SAUSA300_2113	0,94	1,06	0,57785398	No	2416	2721	2639	2124	1880	1693	2441	2388	2473	conserved hypothetical protein	SAUSA300_2113
SAUSA300_0599	0,94	1,07	0,672011838	No	439	494	515	364	449	396	412	506	442	iron compound ABC transporter, permeas	SAUSA300_0599
SAUSA300_0193	0,94	1,07	0,865506873	No	12	15	22	20	23	19	12	13	22	conserved hypothetical protein	SAUSA300_0193
SAUSA300_1313	0,94	1,07	0,414655691	No	3004	2914	2760	3330	3629	3253	2834	2531	2766	carboxyl-terminal protease	ctpA
SAUSA300_1076	0,94	1,07	0,411080701	No	1257	1278	1444	902	929	800	1177	1304	1253	phospho-N-acetylmuramoyl-pentapeptide	mraY
SAUSA300_1507	0,94	1,07	0,691916449	No	1166	1799	1766	1802	1963	2304	1392	1447	1593	glucokinase	glk
SAUSA300_0350	0,94	1,07	0,828980203	No	31	52	47	82	56	65	48	34	41	transcriptional regulator, Cro/CI family-rel	SAUSA300_0350
SAUSA300_2360	0,94	1,07	0,672402433	No	892	879	1253	902	1066	1254	911	1013	909	multidrug resistance protein	SAUSA300_2360
SAUSA300_2388	0,94	1,07	0,620177997	No	1433	1710	1568	1724	1911	1968	1360	1537	1506	2-dehydropantoate 2-reductase	panE
SAUSA300_0592	0,94	1,07	0,363587114	No	2453	2829	2703	2661	2657	2579	2582	2442	2447	conserved hypothetical protein	SAUSA300_0592
SAUSA300_0579	0,93	1,07	0,70833739	No	522	676	631	511	489	540	552	535	623	conserved hypothetical protein	SAUSA300_0579
SAUSA300_0675	0,93	1,07	0,814014536	No	95	51	93	74	50	75	80	85	57	conserved hypothetical protein	SAUSA300_0675
SAUSA300_1230	0,93	1,07	0,80192089	No	503	387	508	99	156	77	442	378	483	conserved hypothetical protein	SAUSA300_1230
SAUSA300_1648	0,93	1,07	0,470239045	No	5086	5594	4658	5356	5637	5927	4816	4929	4576	putative NADP-dependent malic enzyme	SAUSA300_1648
SAUSA300_2076	0,93	1,07	0,704212651	No	3827	4420	3522	5926	5635	7426	3907	3775	3301	aldehyde dehydrogenase family protein	SAUSA300_2076
SAUSA300_1534	0,93	1,07	0,395020811	No	5131	5140	5202	5024	5337	5031	4658	4923	4863	conserved hypothetical protein	SAUSA300_1534
SAUSA300_0351	0,93	1,07	0,770444463	No	136	303	293	209	185	216	242	208	235	putative membrane protein	SAUSA300_0351
SAUSA300_1008	0,93	1,07	0,888364656	No	29	49	90	23	27	37	54	38	65	conserved hypothetical protein	SAUSA300_1008
SAUSA300_0561	0,93	1,07	0,796932216	No	48	55	90	40	41	35	57	60	64	hypothetical protein	SAUSA300_0561
SAUSA300_1614	0,93	1,07	0,549099366	No	6352	6254	4609	6824	6117	6242	5752	5303	4992	glutamate-1-semialdehyde-2,1-aminomut	hemL

SAUSA300_1712	0,93	1,07	0,911424152	No	205	222	222	301	282	319	211	189	194	riboflavin synthase, beta subunit	ribH
SAUSA300_0606	0,93	1,07	0,621609684	No	195	242	184	160	183	145	206	186	187	putative membrane protein	SAUSA300_0606
SAUSA300_2087	0,93	1,07	0,794608367	No	468	999	1155	1001	1098	1485	774	869	801	putative peptidase	SAUSA300_2087
SAUSA300_1927	0,93	1,07	0,884461561	No	14	7	12	17	18	15	8	12	10	phi77 ORF109-like protein	SAUSA300_1927
SAUSA300_2556	0,93	1,07	0,57785398	No	513	480	446	337	293	266	452	413	472	ABC transporter protein	SAUSA300_2556
SAUSA300_1879	0,93	1,07	0,228442598	No	1421	1589	1418	1318	1242	1343	1362	1391	1372	conserved hypothetical protein	SAUSA300_1879
SAUSA300_2126	0,93	1,07	0,45968966	No	940	873	776	1123	1100	1032	860	818	729	drug resistance transporter, EmrB/QacA su	SAUSA300_2126
SAUSA300_0905	0,93	1,07	0,720962976	No	313	361	350	325	316	216	300	333	322	putative adenylate cyclase	SAUSA300_0905
SAUSA300_0383	0,93	1,07	0,50716081	No	5909	6457	4995	6655	6599	7160	5408	5688	5061	conserved hypothetical protein	SAUSA300_0383
SAUSA300_0342	0,93	1,07	0,793918269	No	38	41	41	57	56	55	49	35	28	conserved hypothetical protein	SAUSA300_0342
SAUSA300_0924	0,93	1,07	0,349477464	No	2358	2190	2204	1833	1947	1674	2087	2064	2124	sodium transport family protein	SAUSA300_0924
SAUSA300_2027	0,93	1,07	0,452520966	No	2244	2360	2125	3176	3311	3468	2244	2060	1954	alanine racemase	alr
SAUSA300_2607	0,93	1,08	0,827283739	No	29	33	60	64	95	129	39	38	38	phosphoribosylformimino-5-aminoimidazo	hisA
SAUSA300_1573	0,93	1,08	0,308659139	No	3374	3110	3050	3234	3002	2952	3079	2944	2835	conserved hypothetical protein TIGR0025(	SAUSA300_1573
SAUSA300_1726	0,93	1,08	0,760600425	No	198	108	174	70	79	52	140	170	134	crcB family protein	SAUSA300_1726
SAUSA300_2217	0,93	1,08	0,542932258	No	315	335	264	274	248	209	297	276	273	putative drug transporter	SAUSA300_2217
SAUSA300_0713	0,93	1,08	0,549099366	No	675	623	562	447	554	492	594	570	559	GTP cyclohydrolase I	folE
SAUSA300_1170	0,93	1,08	0,337925258	No	1678	1566	1517	1366	1605	1374	1473	1392	1548	transcriptional regulator, GntR family	SAUSA300_1170
SAUSA300_1798	0,93	1,08	0,474100941	No	529	568	496	318	397	274	478	497	501	DNA-binding response regulator	SAUSA300_1798
SAUSA300_0352	0,93	1,08	0,605423349	No	394	558	489	369	365	364	458	421	461	ABC transporter, ATP-binding protein	SAUSA300_0352
SAUSA300_0841	0,93	1,08	0,487413164	No	2833	3236	2805	3151	3273	3462	2708	3012	2506	conserved hypothetical protein	SAUSA300_0841
SAUSA300_0471	0,93	1,08	0,73636192	No	6026	3888	6294	1600	1659	1238	5256	4723	5002	veg protein	SAUSA300_0471
SAUSA300_0733	0,93	1,08	0,498376629	No	1151	1355	1715	1249	1206	1275	1310	1335	1268	degV family protein	SAUSA300_0733
SAUSA300_1006	0,93	1,08	0,503688668	No	2950	2264	2079	2897	2524	2569	2451	2056	2231	conserved hypothetical protein	SAUSA300_1006
SAUSA300_1384	0,92	1,08	0,916739204	No	0	0	0	3	5	1	0	0	0	phiSLT ORF100b-like protein, holin	SAUSA300_1384
SAUSA300_0793	0,92	1,08	0,650070616	No	351	419	335	408	385	361	365	338	318	conserved hypothetical protein	SAUSA300_0793
SAUSA300_0525	0,92	1,08	0,53373692	No	11226	10247	11896	7341	7118	7709	10249	11135	9451	ribosomal protein L7/L12	rplL
SAUSA300_1678	0,92	1,08	0,613615016	No	2084	3293	2150	2836	2786	3417	2384	2525	2045	formate-tetrahydrofolate ligase	fhs
SAUSA300_0679	0,92	1,08	0,487665656	No	761	630	611	559	460	410	661	603	581	conserved hypothetical protein	SAUSA300_0679
SAUSA300_1585	0,92	1,08	0,461206479	No	1662	1369	1279	1249	1165	1090	1321	1291	1358	conserved hypothetical protein	SAUSA300_1585
SAUSA300_1007	0,92	1,08	0,35583495	No	2537	2242	2015	2674	2578	2641	2138	2206	1920	inositol monophosphatase family protein	SAUSA300_1007
SAUSA300_1393	0,92	1,08	0,71308064	No	71	115	105	156	142	197	87	80	102	phiSLT ORF2067-like protein, phage tail ta	SAUSA300_1393
SAUSA300_1401	0,92	1,08	0,765352133	No	50	36	42	59	77	46	31	49	37	phiSLT ORF387-like protein, putative phag	SAUSA300_1401
SAUSA300_2274	0,92	1,08	0,829357005	No	43	40	99	37	36	13	50	47	71	putative membrane protein	SAUSA300_2274
SAUSA300_0661	0,92	1,08	0,589400204	No	341	234	285	139	158	111	241	255	291	conserved hypothetical protein	SAUSA300_0661
SAUSA300_1041	0,92	1,09	0,871840478	No	150	64	232	40	88	55	121	84	203	conserved hypothetical protein	SAUSA300_1041
SAUSA300_2634	0,92	1,09	0,73745626	No	67	38	69	49	65	28	55	50	55	ABC transporter, permease protein	SAUSA300_2634
SAUSA300_0505	0,92	1,09	0,611170138	No	1728	2541	2081	2001	2026	2081	2414	1868	1564	conserved hypothetical protein	SAUSA300_0505
SAUSA300_0554	0,92	1,09	0,550297947	No	415	474	400	441	397	379	428	395	363	glucosamine-6-phosphate isomerase	SAUSA300_0554
SAUSA300_1003	0,92	1,09	0,333860577	No	14755	12764	12199	14289	13742	13616	12120	12307	12117	conserved hypothetical protein	SAUSA300_1003
SAUSA300_1471	0,92	1,09	0,57785398	No	1102	1033	1072	560	611	469	955	934	1054	exodeoxyribonuclease VII, small subunit	xseB
SAUSA300_2137	0,92	1,09	0,500027291	No	529	500	434	958	1208	939	467	420	452	conserved hypothetical protein	SAUSA300_2137
SAUSA300_0524	0,92	1,09	0,61602727	No	29201	27270	27286	24272	23506	23979	24775	28412	23633	ribosomal protein L10	rplJ
SAUSA300_1004	0,92	1,09	0,494517712	No	1159	947	1031	1083	881	801	1077	894	905	conserved hypothetical protein	SAUSA300_1004
SAUSA300_1418	0,92	1,09	0,908547255	No	0	0	1	1	2	0	0	1	0	phiSLT ORF 82-like protein	SAUSA300_1418
SAUSA300_1791	0,92	1,09	0,378945829	No	7062	6566	5519	5736	5687	5554	6229	5943	5377	cmp-binding-factor 1	cbf1
SAUSA300_2375	0,92	1,09	0,782561825	No	43	86	69	57	43	83	57	65	60	ABC transporter, ATP-binding/permease p	SAUSA300_2375
SAUSA300_2501	0,92	1,09	0,683886816	No	544	535	545	955	872	1105	488	577	423	phytoene dehydrogenase	SAUSA300_2501
SAUSA300_2336	0,92	1,09	0,729332264	No	45	62	53	30	29	15	39	54	52	transcriptional regulator, MerR family	SAUSA300_2336
SAUSA300_1610	0,92	1,09	0,223871744	No	3908	4204	4371	3331	3636	3187	3573	3949	3918	folylpolyglutamate synthase	folC
SAUSA300_1172	0,92	1,09	0,156215203	No	2311	2313	2323	1767	1857	1644	2161	1979	2220	peptidase, M16 family	SAUSA300_1172
SAUSA300_0873	0,92	1,09	0,150252511	No	6461	6119	5692	7063	7499	7105	5422	5848	5451	coenzyme A disulfide reductase	cdr
SAUSA300_1016	0,92	1,09	0,688889013	No	1932	2064	3374	2148	2047	2226	1959	2342	2435	protoheme IX farnesyltransferase	cyoE
SAUSA300_0537	0,92	1,09	0,56330308	No	386	434	378	375	437	356	368	361	366	L-ribulokinase	SAUSA300_0537
SAUSA300_1925	0,91	1,09	0,829512625	No	22	22	28	53	41	87	24	24	19	phiPVL ORF17-like protein	SAUSA300_1925
SAUSA300_0522	0,91	1,09	0,538065387	No	23875	23715	22330	24184	26166	27528	21577	23410	18857	ribosomal protein L11	rplK
SAUSA300_1215	0,91	1,09	0,902920405	No	0	7	6	0	0	1	5	5	1	conserved hypothetical protein	SAUSA300_1215
SAUSA300_1084	0,91	1,09	0,734668701	No	520	407	510	311	304	291	430	425	452	conserved hypothetical protein	SAUSA300_1084
SAUSA300_0739	0,91	1,10	0,463513313	No	227	245	194	258	291	284	202	188	214	LysM domain protein	SAUSA300_0739

SAUSA300_1403	0,91	1,10	0,655285572	No	74	85	64	66	63	56	75	61	67	phiSLT ORF412-like protein, portal protein	SAUSA300_1403
SAUSA300_2321	0,91	1,10	0,505063509	No	214	196	240	213	147	209	196	199	197	putative membrane protein	SAUSA300_2321
SAUSA300_0923	0,91	1,10	0,333860577	No	2089	2323	2105	1842	1920	1661	1883	2007	2048	serine protease	htrA
SAUSA300_0253	0,91	1,10	0,531384026	No	241	189	204	206	216	209	180	191	203	ScdA protein	scdA
SAUSA300_0259	0,91	1,10	0,707244446	No	48	140	98	112	104	98	83	98	82	PTS system, IIA component	SAUSA300_0259
SAUSA300_2033	0,91	1,10	0,614917415	No	93	129	133	168	232	185	113	102	110	K+-transporting ATPase, B subunit	kdpB
SAUSA300_1638	0,91	1,10	0,56330308	No	1571	1495	1504	796	825	627	1338	1338	1478	sensory box histidine kinase PhoR	phoR
SAUSA300_0214	0,91	1,10	0,567075889	No	148	198	167	218	248	243	161	165	143	conserved hypothetical protein	SAUSA300_0214
SAUSA300_2316	0,91	1,10	0,74723361	No	86	168	205	374	417	418	121	130	168	acetyltransferase, GNAT family	SAUSA300_2316
SAUSA300_1576	0,91	1,10	0,241638921	No	5482	4931	4379	4586	4853	4143	4626	4340	4480	helicase, RecD/TraA family	SAUSA300_1576
SAUSA300_2085	0,91	1,10	0,271784627	No	2823	3254	2592	2281	2195	2094	2732	2692	2457	conserved hypothetical protein	SAUSA300_2085
SAUSA300_1087	0,91	1,10	0,191902551	No	10677	11420	10120	10862	11159	11085	9406	10387	9493	isoleucyl-tRNA synthetase	ileS
SAUSA300_1651	0,91	1,10	0,145154124	No	1300	1285	1272	1243	1215	1193	1189	1273	1047	CBS domain protein	SAUSA300_1651
SAUSA300_0044	0,91	1,10	0,723981706	No	22	42	42	30	41	28	37	34	28	metallo-beta-lactamase family protein	SAUSA300_0044
SAUSA300_2324	0,91	1,10	0,673152156	No	320	454	380	226	187	232	281	461	306	PTS system, sucrose-specific IIBC compone	SAUSA300_2324
SAUSA300_1408	0,91	1,10	0,683886816	No	59	77	79	103	50	93	70	65	62	phage helicase	SAUSA300_1408
SAUSA300_0126	0,91	1,10	0,653484961	No	153	182	165	387	563	685	135	169	149	conserved hypothetical protein	SAUSA300_0126
SAUSA300_0647	0,91	1,10	0,338580319	No	756	679	648	733	676	702	723	554	608	ABC transporter, ATP-binding protein	SAUSA300_0647
SAUSA300_1363	0,91	1,10	0,124616826	No	3839	4024	4015	3748	4420	4115	3520	3758	3485	glycerol-3-phosphate dehydrogenase, NAI	gpsA
SAUSA300_1253	0,91	1,10	0,691916449	No	253	249	344	110	131	80	220	225	320	transcription antiterminator	glcT
SAUSA300_2286	0,91	1,10	0,67132604	No	171	311	352	369	327	379	239	290	228	conserved hypothetical protein	SAUSA300_2286
SAUSA300_1913	0,91	1,10	0,473002025	No	1111	1070	1156	600	618	500	984	912	1123	ABC transporter, ATP-binding protein	SAUSA300_1913
SAUSA300_0792	0,91	1,10	0,874121473	No	7	5	6	4	0	0	7	5	4	conserved hypothetical protein	SAUSA300_0792
SAUSA300_1390	0,91	1,10	0,796882563	No	12	12	21	21	18	16	17	13	12	phiSLT ORF96-like protein	SAUSA300_1390
SAUSA300_1557	0,90	1,11	0,50601352	No	622	939	863	563	600	553	704	690	799	hydrolase, HAD-superfamily, subfamily IIIA	SAUSA300_1557
SAUSA300_0882	0,90	1,11	0,47162396	No	150	203	155	146	131	142	155	147	157	conserved hypothetical protein	SAUSA300_0882
SAUSA300_1553	0,90	1,11	0,234450816	No	1738	2053	1954	1481	1537	1461	1691	1769	1735	nicotinate (nicotinamide) nucleotide aden	nadD
SAUSA300_0318	0,90	1,11	0,650846121	No	281	275	252	295	307	358	229	255	243	N-acetylmannosamine-6-phosphate 2-epir	SAUSA300_0318
SAUSA300_2606	0,90	1,11	0,662964175	No	45	66	67	117	122	142	48	50	62	imidazole glycerol phosphate synthase sul	hisF
SAUSA300_0749	0,90	1,11	0,406143756	No	2019	2324	2113	2359	2207	2569	1923	2100	1798	conserved hypothetical protein	SAUSA300_0749
SAUSA300_1915	0,90	1,11	0,876473534	No	2	4	7	7	25	12	4	5	3	conserved hypothetical protein	SAUSA300_1915
SAUSA300_2406	0,90	1,11	0,494438111	No	491	546	442	692	694	711	441	434	454	putative transporter	SAUSA300_2406
SAUSA300_1978	0,90	1,11	0,729332264	No	38	62	56	37	56	43	41	38	61	ferric hydroxamate receptor	SAUSA300_1978
SAUSA300_1193	0,90	1,11	0,526080587	No	243	389	350	438	347	452	337	272	277	glycerol-3-phosphate dehydrogenase	glpD
SAUSA300_1047	0,90	1,11	0,541388684	No	1510	1904	2003	1159	1073	1180	1426	2089	1350	succinate dehydrogenase, flavoprotein sul	sdhA
SAUSA300_1458	0,90	1,11	0,45968966	No	2472	1764	1966	2350	2603	2489	1994	1750	1820	glyoxalase family protein	SAUSA300_1458
SAUSA300_0267	0,90	1,11	0,575818248	No	103	99	99	76	86	47	82	100	87	transposase	SAUSA300_0267
SAUSA300_1109	0,90	1,11	0,320498809	No	1998	2357	2034	1691	1830	1931	1968	1894	1874	methionyl-tRNA formyltransferase	fmt
SAUSA300_1153	0,90	1,11	0,448341492	No	1033	936	1416	607	627	492	946	969	1123	undecaprenyl diphosphate synthase	uppS
SAUSA300_0476	0,90	1,11	0,856494173	No	0	1	0	0	2	0	1	0	0	hypothetical protein	SAUSA300_0476
SAUSA300_0244	0,90	1,11	0,531384026	No	157	177	128	166	180	240	150	137	125	oxidoreductase, zinc-binding dehydrogena	SAUSA300_0244
SAUSA300_0001	0,90	1,11	0,046113616	No	7623	6914	6674	5028	4902	4702	6229	6540	6246	chromosomal replication initiator protein	dnaA
SAUSA300_0053	0,90	1,11	0,814386766	No	107	62	152	54	77	58	103	71	110	Spermidine N(1)-acetyltransferase	speG
SAUSA300_1433	0,90	1,11	0,886584917	No	0	1	0	3	0	0	1	1	0	putative phage regulatory protein	SAUSA300_1433
SAUSA300_2380	0,90	1,12	0,571148031	No	148	205	167	137	119	155	178	149	140	conserved hypothetical protein	SAUSA300_2380
SAUSA300_2534	0,90	1,12	0,614848406	No	1159	2371	1588	2446	2416	3070	1527	1547	1503	3-methyl-2-oxobutanoate hydroxymethylt	panB
SAUSA300_2030	0,90	1,12	0,301606085	No	996	1206	1220	1150	1161	1165	1008	990	1068	putative membrane protein	SAUSA300_2030
SAUSA300_1697	0,90	1,12	0,273472124	No	4170	5508	4817	4856	4794	5377	4293	4495	4185	Peptidase family M20/M25/M40	SAUSA300_1697
SAUSA300_2389	0,89	1,12	0,33107344	No	6004	6141	6369	4984	5700	4723	5355	5724	5465	putative drug transporter	SAUSA300_2389
SAUSA300_0547	0,89	1,12	0,670678359	No	5887	9912	11563	9251	8732	9307	6503	9323	8554	sdrD protein	sdrD
SAUSA300_1353	0,89	1,12	0,116662994	No	699	693	716	444	523	435	627	602	655	conserved hypothetical protein	SAUSA300_1353
SAUSA300_0199	0,89	1,12	0,47491302	No	210	242	303	125	142	160	193	234	251	conserved hypothetical protein	SAUSA300_0199
SAUSA300_1411	0,89	1,12	0,871840478	No	0	0	2	0	0	0	0	1	0	phiSLT ORF66-like protein	SAUSA300_1411
SAUSA300_0331	0,89	1,12	0,725011238	No	19	38	29	40	25	33	26	28	23	conserved hypothetical protein	SAUSA300_0331
SAUSA300_0671	0,89	1,12	0,540338205	No	982	875	1026	780	868	671	857	909	802	ABC transporter, ATP-binding protein, Msl	SAUSA300_0671
SAUSA300_0462	0,89	1,12	0,076498389	No	1586	1808	1651	1376	1463	1545	1572	1499	1434	conserved hypothetical protein	SAUSA300_0462
SAUSA300_1479	0,89	1,12	0,775741842	No	19	16	12	19	9	18	12	15	14	conserved hypothetical protein	SAUSA300_1479
SAUSA300_1639	0,89	1,12	0,347592873	No	1025	1280	981	1128	960	1063	958	1038	933	alkaline phosphatase synthesis transcriptio	phoP
SAUSA300_1696	0,89	1,12	0,045826205	No	2778	3025	2954	2442	2581	2496	2570	2654	2590	D-alanine aminotransferase	dat

SAUSA300_0323	0,89	1,12	0,853676076	No	2	0	1	0	0	0	1	0	1	conserved hypothetical protein	SAUSA300_0323
SAUSA300_0773	0,89	1,12	0,673152156	No	48	40	33	44	52	65	34	36	35	putative staphylocoagulase	SAUSA300_0773
SAUSA300_0942	0,89	1,12	0,865506873	No	3	5	2	4	14	3	4	3	3	conserved hypothetical protein	SAUSA300_0942
SAUSA300_1317	0,89	1,12	0,337931761	No	3577	3782	2900	3711	3793	3892	3270	3028	2822	methionine-S-sulfoxide reductase	msrA
SAUSA300_2048	0,89	1,12	0,487315951	No	88	103	67	153	153	152	74	72	80	hydroxyethylthiazole kinase	thiM
SAUSA300_0438	0,89	1,12	0,421803881	No	6774	9174	7514	10156	11195	13689	6844	7071	6921	CHAP domain family	SAUSA300_0438
SAUSA300_0700	0,89	1,12	0,334371788	No	310	371	295	231	232	179	294	272	300	conserved hypothetical protein	SAUSA300_0700
SAUSA300_0121	0,89	1,13	0,571119811	No	57	83	56	253	361	367	57	56	60	putative drug transporter	SAUSA300_0121
SAUSA300_2601	0,89	1,13	0,703977493	No	24	40	37	26	23	31	26	34	31	intercellular adhesion protein B	icaB
SAUSA300_1373	0,89	1,13	0,41194452	No	915	753	711	856	753	918	772	649	684	ferredoxin	SAUSA300_1373
SAUSA300_0451	0,89	1,13	0,412063383	No	112	155	167	126	128	161	126	125	136	acetyltransferase, GNAT family	SAUSA300_0451
SAUSA300_1893	0,89	1,13	0,534589033	No	482	699	782	1250	1368	1573	591	557	594	NH(3)-dependent NAD+ synthetase	nadE
SAUSA300_1509	0,89	1,13	0,490638981	No	505	638	883	467	485	479	505	556	736	peptidase, rhomboid family	SAUSA300_1509
SAUSA300_2098	0,89	1,13	0,523892959	No	1104	1242	680	898	805	688	910	809	956	transcriptional repressor, ArsR family	arsR
SAUSA300_0123	0,89	1,13	0,552708397	No	146	203	165	497	717	799	160	157	139	siderophore biosynthesis protein, IucC fan	SAUSA300_0123
SAUSA300_0795	0,89	1,13	0,74723361	No	43	36	64	17	25	15	37	33	56	putative thioredoxin	SAUSA300_0795
SAUSA300_2092	0,89	1,13	0,479945187	No	2887	2242	1772	5250	4068	4170	1903	1953	2246	general stress protein 20U	dps
SAUSA300_2272	0,89	1,13	0,371610296	No	1218	1578	1306	1560	1436	1825	1332	1176	1126	conserved hypothetical protein	SAUSA300_2272
SAUSA300_1152	0,89	1,13	0,129592376	No	3901	4690	4425	4497	4503	4924	3806	3818	3911	ribosome recycling factor	rrf
SAUSA300_1173	0,88	1,13	0,200873443	No	1364	1385	1440	1355	1501	1609	1249	1157	1297	putative acetoacetyl-CoA reductase	SAUSA300_1173
SAUSA300_0587	0,88	1,13	0,849169777	No	5	8	11	4	11	6	11	6	4	conserved hypothetical protein	SAUSA300_0587
SAUSA300_0551	0,88	1,13	0,21803518	No	4041	4020	3166	4696	4341	4717	3600	3198	3110	conserved hypothetical protein	SAUSA300_0551
SAUSA300_2112	0,88	1,13	0,347252784	No	2120	2181	2219	1313	1280	998	1937	1810	2007	conserved hypothetical protein	SAUSA300_2112
SAUSA300_0339	0,88	1,13	0,33712533	No	236	287	236	308	239	333	231	217	223	conserved hypothetical protein	SAUSA300_0339
SAUSA300_0843	0,88	1,13	0,122534792	No	1168	1073	991	1548	1465	1501	1011	953	885	conserved hypothetical protein	SAUSA300_0843
SAUSA300_1402	0,88	1,13	0,72766065	No	21	25	13	27	34	30	16	19	15	phiSLT ORF257-like protein, putative prop	SAUSA300_1402
SAUSA300_1909	0,88	1,13	0,57785398	No	3906	5126	2658	4724	3593	4401	3818	3298	3164	conserved hypothetical protein	SAUSA300_1909
SAUSA300_0699	0,88	1,13	0,291014139	No	446	515	396	337	322	312	390	411	394	chorismate binding enzyme domain prote	SAUSA300_0699
SAUSA300_2043	0,88	1,13	0,508605428	No	150	111	116	97	90	109	128	98	102	conserved hypothetical protein	SAUSA300_2043
SAUSA300_1232	0,88	1,13	0,272645874	No	12084	12038	9119	18975	14443	17565	9660	9554	10062	catalase	SAUSA300_1232
SAUSA300_0091	0,88	1,13	0,6937318	No	40	27	36	17	45	15	29	31	30	putative permease	SAUSA300_0091
SAUSA300_0600	0,88	1,13	0,209210345	No	467	598	553	553	570	526	443	471	514	hydrolase, haloacid dehalogenase-like fam	SAUSA300_0600
SAUSA300_0936	0,88	1,14	0,650137921	No	41	38	44	42	50	35	39	39	31	ABC transporter, ATP-binding protein	SAUSA300_0936
SAUSA300_1569	0,88	1,14	0,096159256	No	4142	4438	3967	4444	4828	4377	3722	3659	3664	peptidase, U32 family	SAUSA300_1569
SAUSA300_1524	0,88	1,14	0,341181162	No	415	532	551	497	446	408	404	475	443	CBS domain pair protein	SAUSA300_1524
SAUSA300_1520	0,88	1,14	0,25333519	No	704	757	749	604	631	603	648	640	657	conserved hypothetical protein	SAUSA300_1520
SAUSA300_0309	0,88	1,14	0,313740641	No	1388	1242	1166	882	771	1020	1087	1208	1041	ABC transporter ATP-binding protein	SAUSA300_0309
SAUSA300_2302	0,88	1,14	0,182655427	No	2513	2031	1883	1373	1260	1344	2011	1763	1872	teicoplanin resistance associated membrai	tcaA
SAUSA300_1544	0,88	1,14	0,017249846	No	4991	4999	4754	4156	4526	4225	4448	4265	4251	GTP-binding protein LepA	lepA
SAUSA300_1405	0,88	1,14	0,872417043	No	3	4	0	3	0	1	1	2	2	phiSLT ORF 101-like protein, terminase, sn	SAUSA300_1405
SAUSA300_1552	0,88	1,14	0,190324285	No	1650	1914	1801	1643	1684	1622	1548	1615	1552	conserved hypothetical protein	SAUSA300_1552
SAUSA300_1171	0,88	1,14	0,425595337	No	1888	1576	1863	1060	1071	716	1558	1344	1764	conserved hypothetical protein	SAUSA300_1171
SAUSA300_1784	0,88	1,14	0,234440334	No	3446	3141	3222	2012	1848	1434	3014	2816	2773	signal transduction protein TRAP	SAUSA300_1784
SAUSA300_2351	0,88	1,14	0,451604242	No	1044	1168	863	818	721	847	1008	963	720	Zn-binding lipoprotein adcA-like protein	SAUSA300_2351
SAUSA300_1904	0,88	1,14	0,691916449	No	60	60	85	62	126	70	60	48	71	conserved hypothetical protein	SAUSA300_1904
SAUSA300_1558	0,88	1,14	0,200428258	No	625	795	769	629	568	522	638	613	670	5'-methylthioadenosine/S-adenosylhomoc	mtnN
SAUSA300_2073	0,88	1,14	0,214635739	No	2530	2192	2041	1545	1503	1579	2100	2014	1801	thymidine kinase	tdk
SAUSA300_1842	0,88	1,14	0,518132707	No	3917	2328	2765	1458	1510	1196	2710	2231	2925	transcriptional regulator, Fur family	SAUSA300_1842
SAUSA300_1936	0,88	1,14	0,761141857	No	14	5	12	62	52	72	6	11	10	conserved hypothetical phage protein	SAUSA300_1936
SAUSA300_2028	0,88	1,14	0,219668374	No	770	753	652	855	922	915	660	647	593	holo-(acyl-carrier-protein) synthase	acpS
SAUSA300_1017	0,88	1,14	0,616750609	No	1080	799	1644	329	372	259	846	1009	1215	conserved hypothetical protein	SAUSA300_1017
SAUSA300_0999	0,88	1,14	0,480837613	No	379	508	452	345	327	364	357	344	468	spermidine/putrescine ABC transporter, A	potA
SAUSA300_1327	0,87	1,14	0,332222993	No	1666	2190	1958	2044	2040	1961	1405	1917	1758	cell surface protein	SAUSA300_1327
SAUSA300_0642	0,87	1,14	0,572156734	No	646	423	715	231	293	257	486	421	646	conserved hypothetical protein	SAUSA300_0642
SAUSA300_2056	0,87	1,14	NA	NA	9	12	34	11	11	9	11	6	30	conserved hypothetical protein	SAUSA300_2056
SAUSA300_1665	0,87	1,14	0,219181154	No	734	749	616	901	992	889	646	594	590	conserved hypothetical protein	SAUSA300_1665
SAUSA300_1452	0,87	1,14	0,323591389	No	1328	1693	1345	1243	1258	1381	1231	1281	1300	pyrroline-5-carboxylate reductase	proC
SAUSA300_2407	0,87	1,14	0,362106716	No	153	205	153	241	325	274	146	146	154	oligopeptide ABC transporter, ATP-binding	SAUSA300_2407
SAUSA300_0715	0,87	1,14	0,334860525	No	2313	3020	2923	3171	3631	3630	2453	2558	2195	nrdI protein	nrdI

SAUSA300_0564	0,87	1,15	0,237953172	No	661	720	756	656	699	800	605	621	640	conserved hypothetical protein	SAUSA300_0564
SAUSA300_1644	0,87	1,15	0,018356561	No	33792	31459	29585	28397	27928	27722	28172	28788	25805	pyruvate kinase	pyk
SAUSA300_0616	0,87	1,15	0,751113426	No	16	19	20	14	29	24	18	15	15	putative Na+/H+ antiporter, MnhG compo	SAUSA300_0616
SAUSA300_2363	0,87	1,15	0,27090519	No	716	567	670	463	449	393	610	498	591	cation efflux family protein	SAUSA300_2363
SAUSA300_1628	0,87	1,15	0,498243339	No	532	920	1114	922	884	1136	729	758	748	lysine-specific permease	lysP
SAUSA300_1577	0,87	1,15	0,241638921	No	1145	999	1017	624	633	455	996	834	919	TPR domain protein	SAUSA300_1577
SAUSA300_1914	0,87	1,15	0,387913259	No	512	665	565	481	433	430	497	474	544	GntR family regulatory protein	SAUSA300_1914
SAUSA300_0580	0,87	1,15	0,670715926	No	17	36	35	20	18	22	28	24	25	conserved hypothetical protein	SAUSA300_0580
SAUSA300_1441	0,87	1,15	0,540338205	No	1381	1893	2451	1445	1323	1275	1489	2016	1466	staphylococcal respiratory response prote	srrB
SAUSA300_1555	0,87	1,15	0,151401011	No	878	1075	1155	670	766	670	902	861	946	shikimate 5-dehydrogenase	aroE
SAUSA300_1186	0,87	1,15	0,247937018	No	1827	1957	1575	1220	1186	1053	1537	1476	1641	conserved hypothetical protein	SAUSA300_1186
SAUSA300_2086	0,87	1,15	0,308058509	No	1383	1855	1765	1484	1562	1831	1478	1517	1351	conserved hypothetical protein	SAUSA300_2086
SAUSA300_0866	0,87	1,15	0,396820336	No	648	587	597	660	663	497	575	497	516	conserved hypothetical protein	SAUSA300_0866
SAUSA300_0504	0,87	1,15	0,411192443	No	1686	2743	2477	2502	2443	3012	2355	2006	1631	pyridoxine biosynthesis protein	SAUSA300_0504
SAUSA300_1142	0,87	1,15	0,451223716	No	60	57	64	54	52	58	55	51	52	DNA protecting protein DprA	dprA
SAUSA300_1877	0,87	1,15	0,363587114	No	179	222	235	160	162	197	180	166	207	conserved hypothetical protein	SAUSA300_1877
SAUSA300_0045	0,87	1,15	0,709993652	No	41	19	28	13	18	12	26	21	27	HNH endonuclease family protein	SAUSA300_0045
SAUSA300_1352	0,87	1,15	0,549977721	No	338	246	350	152	223	127	256	254	296	putative membrane protein	SAUSA300_1352
SAUSA300_2311	0,87	1,15	0,404073032	No	107	100	118	57	65	40	82	94	104	conserved hypothetical protein	SAUSA300_2311
SAUSA300_0565	0,87	1,15	0,144059057	No	341	409	428	311	302	297	358	331	338	conserved hypothetical protein	SAUSA300_0565
SAUSA300_2553	0,87	1,15	0,377711205	No	844	921	573	1077	911	1226	703	645	672	putative siroheme synthase	SAUSA300_2553
SAUSA300_1489	0,87	1,15	0,67132604	No	22	22	18	30	11	24	21	18	15	conserved hypothetical protein	SAUSA300_1489
SAUSA300_0236	0,87	1,15	0,542616672	No	458	433	268	633	611	657	367	351	280	PTS system, IIBC components	SAUSA300_0236
SAUSA300_1987	0,87	1,15	0,110046791	No	1039	1061	900	1157	980	1019	872	809	912	hydrolase, carbon-nitrogen family	SAUSA300_1987
SAUSA300_2164	0,87	1,15	0,666349222	No	119	96	82	24	27	47	100	65	90	conserved hypothetical protein	SAUSA300_2164
SAUSA300_1554	0,87	1,16	0,503021348	No	375	345	551	140	210	114	342	314	441	conserved hypothetical protein	SAUSA300_1554
SAUSA300_1194	0,87	1,16	0,322436766	No	226	298	256	239	293	278	207	206	261	hydrolase, alpha/beta hydrolase fold famil	SAUSA300_1194
SAUSA300_1333	0,87	1,16	0,149647093	No	4178	3945	3935	2536	2790	2100	3317	3452	3655	conserved hypothetical protein	SAUSA300_1333
SAUSA300_2497	0,87	1,16	0,359445756	No	615	968	669	861	798	972	598	758	590	aminotransferase, class I	SAUSA300_2497
SAUSA300_1295	0,86	1,16	0,464011331	No	22868	17617	25509	5886	7954	5625	21141	19423	16261	cold shock protein, CSD family	SAUSA300_1295
SAUSA300_0549	0,86	1,16	0,138349728	No	739	753	791	339	352	312	642	677	652	glycosyl transferase, group 1 family protei	SAUSA300_0549
SAUSA300_0243	0,86	1,16	0,801932106	No	2	8	7	7	5	1	2	6	6	conserved hypothetical protein	SAUSA300_0243
SAUSA300_0333	0,86	1,16	0,48346499	No	105	90	70	89	68	70	72	76	78	transcriptional antiterminator, BglG family	SAUSA300_0333
SAUSA300_1174	0,86	1,16	0,023169923	No	2639	2861	2704	2774	2675	2551	2406	2346	2329	conserved hypothetical protein	SAUSA300_1174
SAUSA300_0781	0,86	1,16	0,788708733	No	81	22	90	6	5	12	55	40	66	conserved hypothetical protein	SAUSA300_0781
SAUSA300_0997	0,86	1,16	0,24168699	No	320	290	370	285	277	265	278	303	265	conserved hypothetical protein	SAUSA300_0997
SAUSA300_1400	0,86	1,16	0,74723361	No	12	3	14	16	20	18	9	9	7	phiSLT ORF92-like protein, uncharacterize	SAUSA300_1400
SAUSA300_0213	0,86	1,16	0,379990851	No	90	142	103	140	144	158	105	94	91	oxidoreductase, Gfo/Idh/MocA family	SAUSA300_0213
SAUSA300_0893	0,86	1,16	0,694550386	No	21	21	14	11	14	19	19	14	14	oligopeptide ABC transporter, ATP-binding	oppF
SAUSA300_0009	0,86	1,16	0,084365363	No	4795	4658	4177	4418	4323	4160	3900	4358	3473	seryl-tRNA synthetase	serS
SAUSA300_2156	0,86	1,16	0,133853559	No	680	827	704	740	739	676	628	670	607	lactose phosphotransferase system repres	SAUSA300_2156
SAUSA300_2604	0,86	1,16	0,73636192	No	26	12	12	7	14	12	16	15	11	conserved hypothetical protein	SAUSA300_2604
SAUSA300_2254	0,86	1,16	0,121992673	No	2432	2430	2003	2344	2189	2668	1981	2090	1825	glycerate dehydrogenase-like protein	SAUSA300_2254
SAUSA300_1238	0,86	1,16	0,221817307	No	813	762	711	468	512	367	688	611	662	conserved hypothetical protein	SAUSA300_1238
SAUSA300_2230	0,86	1,16	0,077545744	No	837	812	964	649	624	552	722	737	788	molybdenum ABC transporter, molybdenu	modA
SAUSA300_1198	0,86	1,16	0,190069426	No	530	531	567	455	595	445	495	424	480	putative GTP-binding protein	SAUSA300_1198
SAUSA300_0108	0,86	1,16	0,481832887	No	346	237	193	226	162	210	237	223	201	antigen, 67 kDa	SAUSA300_0108
SAUSA300_2228	0,86	1,16	0,010270951	No	632	682	681	594	568	611	564	598	556	molybdenum ABC transporter, ATP-bindin	modC
SAUSA300_1608	0,86	1,16	0,703932274	No	14	18	11	24	14	33	8	14	14	DNA repair protein RadC	SAUSA300_1608
SAUSA300_2271	0,86	1,17	0,31567445	No	391	320	352	168	178	136	296	270	343	phosphosugar-binding transcriptional regu	SAUSA300_2271
SAUSA300_1615	0,86	1,17	0,122751296	No	2229	2265	1665	2447	2141	2354	1811	1731	1734	delta-aminolevulinic acid dehydratase	hemB
SAUSA300_0059	0,86	1,17	0,794284504	No	7	10	5	3	7	0	4	4	6	conserved hypothetical protein	SAUSA300_0059
SAUSA300_0217	0,86	1,17	0,15056867	No	417	424	354	301	309	275	329	381	311	DNA-binding response regulator, AraC fam	SAUSA300_0217
SAUSA300_0604	0,86	1,17	0,298658104	No	920	760	917	316	264	348	743	758	717	hydrolase, alpha/beta hydrolase fold famil	SAUSA300_0604
SAUSA300_1567	0,86	1,17	0,042431526	No	4893	4397	4378	4081	4136	3394	3941	4038	3707	transcription elongation factor GreA	greA
SAUSA300_0536	0,86	1,17	0,117868388	No	28179	24016	21804	27712	27317	25954	22035	21775	19453	DJ-1/PfpI family protein	SAUSA300_0536
SAUSA300_1294	0,86	1,17	0,832352971	No	14	1	0	4	5	13	7	1	3	conserved hypothetical protein	SAUSA300_1294
SAUSA300_2460	0,86	1,17	0,451223716	No	279	204	267	126	153	98	238	213	188	acetyltransferase family protein	SAUSA300_2460
SAUSA300_2432	0,86	1,17	0,252467192	No	177	242	232	169	187	194	169	200	190	hydrolase, MutT/nudix family	SAUSA300_2432

SAUSA300_2099	0,86	1,17	0,271912824	No	2456	2275	1631	1580	1244	1241	1931	1511	1983	cation efflux family protein	SAUSA300_2099
SAUSA300_1681	0,86	1,17	0,541973596	No	60	79	62	49	72	50	47	66	58	acetoin utilization protein AcuC	acuC
SAUSA300_2496	0,85	1,17	0,190069426	No	734	977	700	891	843	983	683	753	623	D-isomer specific 2-hydroxyacid dehydrog	SAUSA300_2496
SAUSA300_1266	0,85	1,17	0,712314939	No	10	16	9	9	11	10	11	9	11	N-(5'phosphoribosyl)anthranilate isomera	trpF
SAUSA300_0137	0,85	1,17	0,137696366	No	596	686	529	518	392	484	484	538	521	transcriptional regulator, GntR family	SAUSA300_0137
SAUSA300_2277	0,85	1,17	0,34203654	No	215	177	147	205	210	250	152	155	148	imidazolonepropionase	hutI
SAUSA300_0994	0,85	1,17	0,069100344	No	20732	25829	22888	22817	23263	27366	19736	21043	18448	pyruvate dehydrogenase E1 component, b	pdhB
SAUSA300_1151	0,85	1,17	0,104409474	No	3291	4317	4372	4418	4334	4748	3497	3264	3451	uridylate kinase	pyrH
SAUSA300_1706	0,85	1,17	0,16303964	No	3080	2942	2380	3463	3726	3783	2489	2400	2255	conserved hypothetical protein	SAUSA300_1706
SAUSA300_0906	0,85	1,17	0,223871744	No	510	604	524	278	241	183	446	459	487	conserved hypothetical protein	SAUSA300_0906
SAUSA300_2374	0,85	1,17	0,235813115	No	164	177	172	158	140	111	160	132	143	ABC transporter, ATP-binding/permease p	SAUSA300_2374
SAUSA300_2270	0,85	1,17	0,213745058	No	344	313	305	294	291	331	266	319	233	PTS system, arbutin-like IIBC component	glvC
SAUSA300_1301	0,85	1,17	0,041239952	No	2330	2489	2206	2665	2991	2799	1989	1915	2073	conserved hypothetical protein	SAUSA300_1301
SAUSA300_0043	0,85	1,18	0,764702501	No	14	7	4	9	23	13	7	6	6	conserved hypothetical protein	SAUSA300_0043
SAUSA300_1801	0,85	1,18	0,129049158	No	1087	1263	1031	1020	1095	1189	896	1095	880	fumarate hydratase, class II	fumC
SAUSA300_2233	0,85	1,18	0,137843862	No	222	241	246	183	167	201	211	199	193	BioY family protein	SAUSA300_2233
SAUSA300_2428	0,85	1,18	0,414431967	No	119	93	82	89	97	106	81	82	83	staphylococcal tandem lipoprotein	SAUSA300_2428
SAUSA300_1742	0,85	1,18	0,35137519	No	143	118	117	60	72	55	91	122	104	conserved hypothetical protein	SAUSA300_1742
SAUSA300_0338	0,85	1,18	0,208178351	No	148	156	156	127	115	138	135	137	120	glyoxalase family protein	SAUSA300_0338
SAUSA300_1799	0,85	1,18	0,028898168	No	818	840	718	662	712	766	685	660	664	putative sensor histidine kinase	SAUSA300_1799
SAUSA300_1219	0,85	1,18	0,296588514	No	186	149	165	127	128	96	144	132	145	putative sensor histidine kinase	SAUSA300_1219
SAUSA300_2642	0,85	1,18	0,696819811	No	17	8	23	19	14	6	9	19	12	conserved hypothetical protein	SAUSA300_2642
SAUSA300_1354	0,85	1,18	0,129049158	No	2410	2275	2228	1306	1458	1037	2010	1844	1984	conserved hypothetical protein	SAUSA300_1354
SAUSA300_0588	0,85	1,18	0,195580892	No	150	141	142	70	95	87	127	110	128	conserved hypothetical protein	SAUSA300_0588
SAUSA300_2570	0,85	1,18	0,643572941	No	21	30	20	34	41	31	21	17	21	arginine deiminase	arcA
SAUSA300_2478	0,84	1,18	0,236347095	No	1900	1744	1456	1824	1938	1955	1433	1742	1123	Holin-like protein cidB	cidB
SAUSA300_2349	0,84	1,18	0,057890531	No	839	903	988	632	577	637	776	785	747	formate/nitrite transporter family protein	SAUSA300_2349
SAUSA300_1394	0,84	1,19	0,827283739	No	0	1	0	4	2	0	1	1	0	conserved hypothetical phage protein	SAUSA300_1394
SAUSA300_0996	0,84	1,19	0,005947709	Yes	42936	47779	45256	42504	43115	47193	38002	40035	36561	dihydrolipoamide dehydrogenase	lpdA
SAUSA300_2456	0,84	1,19	0,172571127	No	785	819	1003	730	746	608	633	796	765	putative membrane protein	SAUSA300_2456
SAUSA300_0440	0,84	1,19	0,108407529	No	320	356	275	404	421	379	289	259	252	MutT/nudix family protein	SAUSA300_0440
SAUSA300_2536	0,84	1,19	0,215883477	No	746	1016	793	1002	949	933	743	794	612	alpha-acetolactate decarboxylase	budA
SAUSA300_0929	0,84	1,19	0,451635238	No	203	298	314	87	108	96	206	261	219	conserved hypothetical protein	SAUSA300_0929
SAUSA300_1311	0,84	1,19	0,071752984	No	1219	1396	1437	825	989	788	1130	1117	1162	undecaprenyldiphospho-muramoylpentap	murG
SAUSA300_1281	0,84	1,19	0,463657425	No	36	48	42	50	41	59	27	42	37	phosphate ABC transporter, permease pro	pstA
SAUSA300_1574	0,84	1,19	0,014666176	No	2112	2150	2207	1688	1521	1340	1905	1826	1708	conserved hypothetical protein	SAUSA300_1574
SAUSA300_0340	0,84	1,19	0,192215158	No	172	153	145	133	92	124	143	109	140	NADH-dependent FMN reductase	SAUSA300_0340
SAUSA300_0665	0,84	1,19	0,179592949	No	594	552	378	358	298	290	436	424	413	acetyltransferase, GNAT family	SAUSA300_0665
SAUSA300_1038	0,84	1,19	0,010264744	No	6261	6579	5748	4545	4330	4064	5186	5339	5075	phenylalanyl-tRNA synthetase, beta subur	pheT
SAUSA300_1046	0,84	1,19	0,34572331	No	160	185	261	84	61	58	149	198	163	succinate dehydrogenase, cytochrome b-5	sdhC
SAUSA300_0540	0,84	1,19	0,228442598	No	1357	1691	1026	1648	1497	1769	1262	1094	1055	HAD-superfamily hydrolase, subfamily IA,	SAUSA300_0540
SAUSA300_0577	0,84	1,19	0,450107997	No	236	301	148	255	187	211	242	150	178	putative transcriptional regulator	SAUSA300_0577
SAUSA300_1593	0,84	1,19	0,009532577	Yes	11853	10499	10147	7632	7873	6838	9009	8824	9419	protein-export membrane protein SecF	secF
SAUSA300_0404	0,84	1,19	0,445290421	No	52	47	56	40	36	37	37	46	45	exotoxin	SAUSA300_0404
SAUSA300_1722	0,84	1,19	0,735037065	No	10	5	13	4	9	4	10	5	9	conserved hypothetical protein	SAUSA300_1722
SAUSA300_1011	0,84	1,19	0,092249655	No	927	812	886	710	672	525	754	738	705	conserved hypothetical protein	SAUSA300_1011
SAUSA300_0583	0,84	1,19	0,668822956	No	69	36	74	14	9	13	44	38	64	conserved hypothetical protein	SAUSA300_0583
SAUSA300_2212	0,84	1,19	0,627101502	No	226	149	267	57	56	13	175	174	183	conserved hypothetical protein	SAUSA300_2212
SAUSA300_1898	0,84	1,19	0,327609532	No	241	389	298	271	246	333	241	281	254	conserved hypothetical protein	SAUSA300_1898
SAUSA300_0552	0,84	1,19	0,100442801	No	2890	3465	2401	4286	4086	4581	2687	2448	2190	conserved hypothetical protein	SAUSA300_0552
SAUSA300_0664	0,84	1,19	0,108550315	No	2659	1885	2055	1061	989	960	1948	1874	1693	conserved hypothetical protein	SAUSA300_0664
SAUSA300_1419	0,84	1,19	0,813982976	No	0	0	2	1	0	3	1	0	0	phiSLT ORF80-like protein	SAUSA300_1419
SAUSA300_1423	0,84	1,19	0,73371321	No	2	10	9	6	9	12	6	5	7	phage related DNA polymerase, family A	polA
SAUSA300_2101	0,84	1,20	0,672236544	No	41	36	110	36	56	47	49	40	66	SAP domain protein	SAUSA300_2101
SAUSA300_2320	0,84	1,20	0,081699231	No	277	246	264	285	257	327	216	217	224	conserved hypothetical protein	SAUSA300_2320
SAUSA300_1410	0,84	1,20	0,313740641	No	93	119	114	107	117	129	105	79	90	virulence-associated protein E	SAUSA300_1410
SAUSA300_1618	0,84	1,20	0,268312816	No	1032	866	1028	467	385	420	784	804	851	hemA concentration negative effector her	hemX
SAUSA300_2292	0,84	1,20	0,011847133	No	1821	2085	1898	1816	1972	2125	1554	1677	1620	isopentenyl-diphosphate delta-isomerase	fni
SAUSA300_0601	0,84	1,20	0,067060194	No	901	1085	956	846	843	711	784	830	844	hydrolase, alpha/beta hydrolase fold famil	SAUSA300_0601

SAUSA300_1110	0,84	1,20	0,019310568	No	2463	2527	2685	1597	1889	1513	2147	2130	2131	ribosomal RNA small subunit methyltransf	sun
SAUSA300_2328	0,84	1,20	0,63492123	No	375	185	434	107	142	59	243	234	341	conserved hypothetical protein	SAUSA300_2328
SAUSA300_1309	0,83	1,20	0,24168699	No	315	238	284	202	216	161	242	212	241	transposase, IS200 family	SAUSA300_1309
SAUSA300_0581	0,83	1,20	0,307911682	No	148	140	118	62	68	75	127	89	119	conserved hypothetical protein	SAUSA300_0581
SAUSA300_1937	0,83	1,20	0,676536871	No	41	21	5	125	126	130	11	17	24	phi77 ORF045-like protein	SAUSA300_1937
SAUSA300_1910	0,83	1,20	0,517591466	No	165	112	240	86	88	68	131	124	174	putative membrane protein	SAUSA300_1910
SAUSA300_0332	0,83	1,20	0,508581571	No	52	33	32	40	23	28	33	36	26	PTS system, IIA component	SAUSA300_0332
SAUSA300_1536	0,83	1,20	0,036382585	No	5346	6587	5468	5105	5657	5108	4880	4859	4736	conserved hypothetical protein	SAUSA300_1536
SAUSA300_1519	0,83	1,20	0,0102725	No	1645	1722	1664	1242	1298	1054	1397	1356	1431	conserved hypothetical protein	SAUSA300_1519
SAUSA300_1322	0,83	1,20	0,014191079	No	2105	2524	2045	2078	2038	2175	1936	1916	1698	conserved hypothetical protein	SAUSA300_1322
SAUSA300_0697	0,83	1,20	0,176495242	No	501	582	491	417	570	439	413	494	400	exsB protein	SAUSA300_0697
SAUSA300_2431	0,83	1,20	0,143633915	No	1054	1031	1159	779	751	698	860	807	1024	putative helicase	SAUSA300_2431
SAUSA300_0754	0,83	1,20	0,098248259	No	2472	2067	1985	2278	1981	2585	1865	1982	1564	conserved hypothetical protein	SAUSA300_0754
SAUSA300_1599	0,83	1,20	0,277870041	No	427	386	567	364	408	294	370	378	396	ACT domain protein PheB	SAUSA300_1599
SAUSA300_1999	0,83	1,20	0,047666618	No	4435	4309	4026	4975	4753	4830	3708	3687	3196	redox-sensing transcriptional repressor re:	rex
SAUSA300_1316	0,83	1,20	0,172571127	No	1674	1860	1490	1614	1526	1261	1461	1408	1294	methionine-R-sulfoxide reductase	msrB
SAUSA300_2605	0,83	1,20	0,394851222	No	55	74	82	99	86	126	59	64	53	histidine biosynthesis bifunctional protein	hisIE
SAUSA300_0122	0,83	1,21	0,334686867	No	112	153	128	458	642	705	119	105	102	siderophore biosynthesis protein, IucA/Iuc	SAUSA300_0122
SAUSA300_1741	0,83	1,21	0,499465296	No	47	26	30	6	14	13	28	32	23	putative lipoprotein	SAUSA300_1741
SAUSA300_1704	0,83	1,21	0,132994973	No	3500	5119	4190	4057	4089	4615	3531	3635	3427	leucyl-tRNA synthetase	leuS
SAUSA300_0541	0,83	1,21	0,027597594	No	1666	1372	1436	855	935	726	1190	1153	1352	deoxynucleoside kinase family protein	SAUSA300_0541
SAUSA300_1179	0,83	1,21	0,045340831	No	26528	25513	20965	28063	27495	29857	20802	20634	18952	conserved hypothetical protein	SAUSA300_1179
SAUSA300_1967	0,83	1,21	0,709993652	No	12	14	11	6	9	6	11	9	9	conserved hypothetical phage protein	SAUSA300_1967
SAUSA300_2412	0,83	1,21	0,277488016	No	84	89	74	79	72	64	61	69	72	conserved hypothetical protein	SAUSA300_2412
SAUSA300_2333	0,83	1,21	0,542489687	No	157	105	176	70	108	90	107	151	100	nitrite extrusion protein	narK
SAUSA300_0082	0,83	1,21	0,168415659	No	489	567	390	430	467	501	399	393	398	conserved hypothetical protein	SAUSA300_0082
SAUSA300_2258	0,83	1,21	0,08580832	No	9934	11175	10767	5962	5409	6702	8867	9162	8287	formate dehydrogenase, alpha subunit	SAUSA300_2258
SAUSA300_2367	0,83	1,21	0,188167855	No	115	85	89	112	126	111	68	85	82	gamma-hemolysin component B	hlgB
SAUSA300_1612	0,83	1,21	0,019494004	No	1121	1139	1097	752	760	736	1000	930	840	DNA-3-methyladenine glycosidase	tag
SAUSA300_1112	0,83	1,21	0,138127781	No	1421	1888	1945	1562	1722	1680	1350	1450	1530	protein phosphatase 2C domain protein	SAUSA300_1112
SAUSA300_2072	0,83	1,21	0,014908354	No	4204	3779	3590	2791	2844	2853	3201	3267	3067	peptide chain release factor 1	prfA
SAUSA300_1550	0,82	1,21	0,182698188	No	1202	949	1503	617	742	553	968	976	1063	conserved hypothetical protein	SAUSA300_1550
SAUSA300_0591	0,82	1,21	0,190397575	No	281	385	443	259	248	214	317	283	316	acetyltransferase, GNAT family	SAUSA300_0591
SAUSA300_0461	0,82	1,21	0,067384923	No	1323	1671	1561	896	816	722	1149	1226	1376	DNA polymerase III delta subunit	holB
SAUSA300_2095	0,82	1,21	0,127617106	No	560	506	491	311	302	274	418	486	376	conserved hypothetical protein	SAUSA300_2095
SAUSA300_2020	0,82	1,21	0,026155382	No	1132	1051	938	812	782	660	884	901	782	metallopeptidase, SprT family	SAUSA300_2020
SAUSA300_1723	0,82	1,21	0,660523594	No	167	68	259	53	88	50	115	95	189	conserved hypothetical protein	SAUSA300_1723
SAUSA300_0470	0,82	1,21	0,026916326	No	983	1220	1318	1362	1298	1272	1010	972	924	dimethyladenosine transferase	ksgA
SAUSA300_0529	0,82	1,21	0,362430594	No	3574	3266	4193	1328	1557	671	2986	2994	3061	conserved hypothetical protein	SAUSA300_0529
SAUSA300_1905	0,82	1,21	0,139320042	No	765	1032	904	1030	1082	1031	716	788	719	conserved hypothetical protein	SAUSA300_1905
SAUSA300_0469	0,82	1,22	0,088540988	No	591	906	723	852	906	951	660	611	556	primase-related protein	SAUSA300_0469
SAUSA300_2472	0,82	1,22	0,151262792	No	499	706	756	531	521	593	517	576	521	putative membrane protein	SAUSA300_2472
SAUSA300_1113	0,82	1,22	0,007929898	Yes	5057	5863	5880	4634	4812	4621	4499	4561	4747	protein kinase	pknB
SAUSA300_1195	0,82	1,22	0,003689505	Yes	1228	1088	1104	934	866	809	883	939	980	tRNA delta(2)-isopentenylpyrophosphate	miaA
SAUSA300_1368	0,82	1,22	0,026625238	No	1452	1524	1237	1359	1278	1387	1212	1107	1135	L-asparaginase	ansA
SAUSA300_2054	0,82	1,22	0,037357801	No	2491	2530	2462	2056	2132	2295	2099	2134	1905	(3R)-hydroxymyristoyl-[acyl carrier proteir	fabZ
SAUSA300_1510	0,82	1,22	0,128180255	No	205	271	268	216	147	149	186	199	227	5-formyltetrahydrofolate cyclo-ligase subf	SAUSA300_1510
SAUSA300_1948	0,82	1,22	0,74689672	No	7	0	7	23	14	30	4	5	3	phi77 ORF069-like protein	SAUSA300_1948
SAUSA300_2414	0,82	1,22	0,449464375	No	79	56	32	79	70	67	33	49	50	conserved hypothetical protein	SAUSA300_2414
SAUSA300_0463	0,82	1,22	0,022645215	No	687	798	776	506	543	469	671	627	560	conserved hypothetical protein	SAUSA300_0463
SAUSA300_2000	0,82	1,22	0,078216364	No	1912	2119	1687	1488	1377	1431	1594	1594	1489	ABC transporter, ATP-binding protein	vga
SAUSA300_2575	0,82	1,22	0,20601715	No	296	308	238	211	176	176	201	213	269	transcriptional antiterminator, BglG family	SAUSA300_2575
SAUSA300_0998	0,82	1,22	0,328824673	No	270	326	382	195	180	211	249	226	322	conserved hypothetical protein	SAUSA300_0998
SAUSA300_0771	0,82	1,22	0,362765435	No	233	327	212	306	270	383	236	205	187	acetyltransferase, GNAT family	SAUSA300_0771
SAUSA300_0084	0,82	1,22	0,288365472	No	53	71	72	46	77	49	57	49	56	conserved hypothetical protein	SAUSA300_0084
SAUSA300_2173	0,82	1,22	0,033678997	No	1834	1785	1572	1499	1571	1612	1463	1458	1317	tRNA pseudouridine synthase A	truA
SAUSA300_0560	0,82	1,22	0,213826737	No	312	361	286	255	223	355	243	264	274	acetyl-CoA c-acetyltransferase	vraB
SAUSA300_1744	0,82	1,22	0,290721528	No	122	111	93	66	70	35	71	108	85	conserved hypothetical protein	SAUSA300_1744
SAUSA300_2055	0,82	1,22	0,044216246	No	6641	6913	6908	6327	6832	7440	5691	6032	4979	UDP-N-acetylglucosamine 1-carboxyvinyltı	murA

SAUSA300_1934	0,82	1,22	0,460329598	No	41	29	28	242	228	288	22	31	26	phi77 ORF020-like protein, phage major ta	SAUSA300_1934
SAUSA300_1404	0,82	1,22	0,360206993	No	57	55	42	59	52	52	50	31	42	phiSLT ORF 563-like protein, terminase, lar	SAUSA300_1404
SAUSA300_2609	0,82	1,23	0,478966696	No	22	38	44	59	36	43	25	27	34	imidazole glycerol phosphate dehydratase	hisB
SAUSA300_0941	0,82	1,23	0,192215158	No	191	214	166	223	192	254	166	143	155	putative ferrichrome ABC transporter	SAUSA300_0941
SAUSA300_0486	0,82	1,23	0,015561363	No	6309	5790	5495	4978	4986	4476	5041	5226	4065	polyribonucleotide nucleotidyltransferase	SAUSA300_0486
SAUSA300_1907	0,82	1,23	0,05205678	No	660	601	658	468	482	384	493	487	581	conserved hypothetical protein	SAUSA300_1907
SAUSA300_1491	0,81	1,23	0,061548	No	3254	3652	2979	3944	3548	3726	2825	2541	2676	proline dipeptidase	SAUSA300_1491
SAUSA300_2543	0,81	1,23	0,298438572	No	282	223	217	107	115	92	196	207	181	conserved hypothetical protein	SAUSA300_2543
SAUSA300_1255	0,81	1,23	0,100129869	No	3379	3195	3626	1867	1830	1486	2797	2458	3034	oxacillin resistance-related FmtC protein	fmtC
SAUSA300_0106	0,81	1,23	0,152167433	No	453	602	485	560	548	648	479	388	386	putative drug transporter	SAUSA300_0106
SAUSA300_1962	0,81	1,23	0,630196817	No	62	16	19	146	133	129	14	40	22	phiPVL ORF39-like protein	SAUSA300_1962
SAUSA300_1315	0,81	1,23	0,056499022	No	2833	3013	2312	3686	3176	3540	2410	2212	2007	PTS system, glucose-specific IIA componer	crr
SAUSA300_2315	0,81	1,23	0,093965238	No	2809	2313	2090	1189	1030	938	1961	1839	2054	putative lipoprotein	SAUSA300_2315
SAUSA300_0197	0,81	1,23	0,341957338	No	482	408	539	193	160	105	368	365	423	conserved hypothetical protein	SAUSA300_0197
SAUSA300_1765	0,81	1,23	0,411080701	No	34	48	51	27	16	12	35	34	41	lantibiotic epidermin biosynthesis protein	epiC
SAUSA300_0630	0,81	1,23	0,088799069	No	5512	6091	6166	3731	3471	3576	4361	4124	5940	ABC transporter, ATP-binding protein	SAUSA300_0630
SAUSA300_1753	0,81	1,23	0,560662833	No	16	18	13	10	11	21	11	12	14	serine protease SplF	splF
SAUSA300_2424	0,81	1,23	0,096953424	No	146	145	167	122	142	140	127	117	129	putative staphylococcal tandem lipoprotei	SAUSA300_2424
SAUSA300_1271	0,81	1,23	0,078718925	No	906	861	821	454	485	316	663	711	723	hydrolase-related protein	SAUSA300_1271
SAUSA300_0083	0,81	1,23	0,078619992	No	224	246	187	236	255	271	183	179	169	putative membrane protein	SAUSA300_0083
SAUSA300_2626	0,81	1,23	0,308058509	No	317	220	233	112	142	149	248	211	163	conserved hypothetical protein	SAUSA300_2626
SAUSA300_2158	0,81	1,23	0,764702501	No	2	3	4	7	5	4	3	1	2	conserved hypothetical protein	SAUSA300_2158
SAUSA300_0481	0,81	1,23	0,080856646	No	2733	3564	3301	4610	4589	5417	2537	2600	2645	transcription-repair coupling factor	mfd
SAUSA300_2288	0,81	1,23	0,005225957	Yes	1056	1120	954	939	881	933	831	862	844	ABC transporter, ATP-binding protein	SAUSA300_2288
SAUSA300_2612	0,81	1,23	0,484592745	No	34	29	28	43	25	41	19	26	26	ATP phosphoribosyltransferase hisG	hisG
SAUSA300_1963	0,81	1,23	0,531321579	No	43	19	16	102	74	96	21	24	17	conserved hypothetical phage protein	SAUSA300_1963
SAUSA300_2282	0,81	1,23	0,010756791	No	7461	7100	5799	4717	5141	4861	5347	5283	5863	putative membrane protein	SAUSA300_2282
SAUSA300_1750	0,81	1,23	0,241069855	No	269	279	258	249	300	278	228	171	251	conserved hypothetical protein	SAUSA300_1750
SAUSA300_0915	0,81	1,23	0,118137415	No	288	375	291	261	241	336	283	246	243	conserved hypothetical protein	SAUSA300_0915
SAUSA300_0118	0,81	1,23	0,478442904	No	14	38	29	242	309	374	20	21	25	pyridoxal-phosphate dependent enzyme s	SAUSA300_0118
SAUSA300_2287	0,81	1,23	0,100095643	No	1450	1090	1161	848	769	702	958	950	1082	putative membrane protein	SAUSA300_2287
SAUSA300_0717	0,81	1,23	0,044487057	No	13624	11953	12266	8210	8977	7255	10019	10270	10341	ribonucleoside-diphosphate reductase, be	SAUSA300_0717
SAUSA300_1619	0,81	1,23	0,012986623	No	10391	9962	11129	8760	7832	7299	8109	8804	8583	glutamyl-tRNA reductase	hemA
SAUSA300_1108	0,81	1,23	0,003659855	Yes	816	802	816	531	536	568	688	639	646	polypeptide deformylase	def
SAUSA300_1764	0,81	1,23	0,687935257	No	3	5	13	1	0	1	6	4	8	lantibiotic epidermin biosynthesis protein	epiD
SAUSA300_0563	0,81	1,23	0,00717478	Yes	582	591	664	490	500	574	492	481	516	uracil-DNA glycosylase	ung
SAUSA300_0218	0,81	1,23	0,004730749	Yes	608	667	581	440	451	396	471	533	496	sensor histidine kinase family protein	SAUSA300_0218
SAUSA300_1485	0,81	1,23	0,555966777	No	16	23	20	16	36	46	17	19	12	conserved hypothetical protein	SAUSA300_1485
SAUSA300_1480	0,81	1,24	0,459260664	No	31	34	25	29	29	40	26	23	23	putative traG membrane protein	SAUSA300_1480
SAUSA300_1930	0,81	1,24	0,251927415	No	186	104	119	682	611	821	92	126	109	phi77 ORF001-like protein, phage tail tape	SAUSA300_1930
SAUSA300_1571	0,81	1,24	0,139866656	No	677	531	620	239	257	214	482	419	568	O-methyltransferase family protein	SAUSA300_1571
SAUSA300_1766	0,81	1,24	0,337925258	No	103	74	85	40	38	27	69	55	86	lantibiotic epidermin biosynthesis protein	epiB
SAUSA300_1556	0,81	1,24	0,002019839	Yes	1769	2026	1997	1494	1542	1470	1552	1515	1607	putative GTP-binding protein	SAUSA300_1556
SAUSA300_0698	0,81	1,24	0,144649193	No	258	220	156	153	178	139	184	168	155	para-aminobenzoate synthase, glutamine a	pabA
SAUSA300_2636	0,81	1,24	0,115127421	No	351	270	244	266	340	279	209	258	223	integrase/recombinase	SAUSA300_2636
SAUSA300_1928	0,81	1,24	0,150620659	No	334	233	204	657	575	694	209	211	195	phi77 ORF002-like protein, phage minor st	SAUSA300_1928
SAUSA300_1792	0,80	1,24	0,002269268	Yes	11280	10327	9754	5893	5754	5161	8334	8259	8612	conserved hypothetical protein	SAUSA300_1792
SAUSA300_0308	0,80	1,24	0,036688141	No	1318	1305	1281	710	588	642	1075	1147	910	ABC transporter, permease protein	SAUSA300_0308
SAUSA300_0993	0,80	1,24	0,000424308	Yes	32592	37809	33790	30571	29226	32487	27485	29130	27024	pyruvate dehydrogenase E1 component, a	pdhA
SAUSA300_2490	0,80	1,25	0,041024125	No	546	494	440	275	241	225	393	386	404	regulatory protein, TetR family	SAUSA300_2490
SAUSA300_0275	0,80	1,25	0,705237363	No	16	12	14	21	34	12	15	5	12	putative membrane protein	SAUSA300_0275
SAUSA300_1617	0,80	1,25	0,000285772	Yes	3279	3330	2856	2867	2500	2607	2485	2585	2508	porphobilinogen deaminase	hemC
SAUSA300_0680	0,80	1,25	0,078130216	No	1058	1477	1545	1256	1420	1433	1157	1106	1003	multi drug resistance protein	norA
SAUSA300_2096	0,80	1,25	0,002454248	Yes	2335	2130	2164	1456	1255	1406	1722	1823	1757	mannose-6-phosphate isomerase	manA
SAUSA300_1494	0,80	1,25	0,02924383	No	1464	1666	1308	1756	1612	1777	1173	1230	1143	conserved hypothetical protein	SAUSA300_1494
SAUSA300_1175	0,80	1,25	0,030534254	No	1819	1977	1792	1975	1983	1956	1524	1458	1484	conserved hypothetical protein	SAUSA300_1175
SAUSA300_1015	0,80	1,25	0,230481002	No	1736	1092	1513	1333	1192	1177	1094	1258	1101	cytochrome oxidase assembly protein	ctaA
SAUSA300_1616	0,80	1,25	0,044508302	No	1080	1043	970	556	561	469	833	736	894	uroporphyrinogen-III synthase	hemD
SAUSA300_1911	0,80	1,25	0,062061235	No	1355	1341	1488	769	735	731	1120	958	1255	ABC transporter, ATP-binding protein	SAUSA300_1911

SAUSA300_0392	0,80	1,25	0,492579862	No	31	21	34	14	16	1	24	17	26	conserved hypothetical protein	SAUSA300_0392
SAUSA300_2229	0,80	1,25	0,001064333	Yes	806	968	784	904	856	929	670	707	664	molybdenum ABC transporter, permease p	modB
SAUSA300_2293	0,80	1,25	0,139866656	No	358	352	537	143	185	130	310	287	397	magnesium and cobalt transport protein	corA
SAUSA300_1589	0,80	1,25	0,000553204	Yes	2441	2463	2263	1684	1618	1593	1930	1867	1915	D-tyrosyl-tRNA (Tyr) deacylase	dtd
SAUSA300_0047	0,80	1,25	0,190140035	No	255	215	210	116	149	93	191	151	197	conserved hypothetical protein	SAUSA300_0047
SAUSA300_0995	0,80	1,25	0,00356932	Yes	35406	42527	40160	38046	39606	44036	30745	32428	30894	dihydrolipoamide acetyltransferase	SAUSA300_0995
SAUSA300_2376	0,80	1,26	0,450107997	No	36	25	56	16	16	16	27	24	41	conserved hypothetical protein	SAUSA300_2376
SAUSA300_1849	0,80	1,26	0,005174561	Yes	797	914	803	838	778	902	670	661	668	A/G-specific adenine glycosylase	mutY
SAUSA300_0066	0,80	1,26	0,760600425	No	2	1	1	1	5	3	1	1	1	arginine repressor	argR
SAUSA300_0670	0,79	1,26	0,151051147	No	744	704	778	594	611	634	588	621	553	ABC transporter, ATP-binding protein, Msl	SAUSA300_0670
SAUSA300_2257	0,79	1,26	0,000340475	Yes	2074	1915	2038	908	780	818	1691	1561	1532	conserved hypothetical protein	SAUSA300_2257
SAUSA300_1707	0,79	1,26	0,040266799	No	1777	1645	1507	1432	1447	1164	1320	1276	1307	conserved hypothetical protein	SAUSA300_1707
SAUSA300_1310	0,79	1,26	0,029920216	No	925	1240	1131	756	809	674	893	866	855	PAP2 family protein	SAUSA300_1310
SAUSA300_0138	0,79	1,26	0,445290421	No	29	18	29	52	32	35	25	19	16	purine nucleoside phosphorylase	deoD
SAUSA300_1637	0,79	1,26	0,238333588	No	91	79	91	53	59	46	60	66	79	putative membrane protein	SAUSA300_1637
SAUSA300_2408	0,79	1,26	0,060420552	No	155	156	182	233	243	274	135	130	126	oligopeptide ABC transporter, ATP-binding	SAUSA300_2408
SAUSA300_1916	0,79	1,26	0,001147142	Yes	7503	7969	6836	5280	5049	4531	5819	6214	5630	aminotransferase	SAUSA300_1916
SAUSA300_1954	0,79	1,26	0,660756014	No	9	4	4	32	20	40	4	2	5	phiPVL ORF050-like protein	SAUSA300_1954
SAUSA300_1328	0,79	1,26	0,156215203	No	90	83	95	77	108	86	70	66	74	putative drug transporter	SAUSA300_1328
SAUSA300_0094	0,79	1,26	0,720962976	No	5	4	7	0	0	0	4	5	2	conserved hypothetical protein	SAUSA300_0094
SAUSA300_1111	0,79	1,26	0,004917578	Yes	2566	2901	2994	2075	2367	2146	2161	2216	2317	conserved hypothetical protein	SAUSA300_1111
SAUSA300_2265	0,79	1,26	0,057890531	No	1748	2194	2599	1784	1805	1887	1692	1888	1592	putative amino acid permease	SAUSA300_2265
SAUSA300_1757	0,79	1,26	0,663931612	No	14	7	6	3	2	12	6	8	6	serine protease SplB	splB
SAUSA300_2335	0,79	1,26	0,44723531	No	17	30	21	30	20	31	17	19	18	conserved hypothetical protein	SAUSA300_2335
SAUSA300_2477	0,79	1,27	0,101898584	No	8193	6837	5844	7710	7618	7528	5754	6250	4421	pyruvate oxidase	cidC
SAUSA300_1436	0,79	1,27	0,241327662	No	739	836	683	351	313	278	616	533	621	phiSLT ORF144-like protein, putative lipop	SAUSA300_1436
SAUSA300_2041	0,79	1,27	0,498666732	No	605	281	903	50	68	41	464	357	562	conserved hypothetical protein	SAUSA300_2041
SAUSA300_0196	0,79	1,27	0,032782054	No	4218	4757	3543	3278	3180	2974	3217	3437	3182	type I restriction-modification enzyme, R s	hsdR
SAUSA300_2071	0,79	1,27	0,011250491	No	3576	3166	3051	2276	2452	2196	2540	2606	2550	modification methylase, HemK family	SAUSA300_2071
SAUSA300_2175	0,79	1,27	0,002213687	Yes	1922	2077	1651	1753	1868	1788	1545	1454	1439	ABC transporter, ATP-binding protein	SAUSA300_2175
SAUSA300_0169	0,79	1,27	0,380244444	No	72	56	85	50	43	27	49	45	72	conserved hypothetical protein	SAUSA300_0169
SAUSA300_0676	0,79	1,27	0,001645476	Yes	548	517	487	441	397	405	410	444	365	anion transporter family protein	SAUSA300_0676
SAUSA300_1455	0,79	1,27	0,05855928	No	3154	2973	2510	1817	1670	1329	2299	2114	2354	transcriptional regulator, AraC family	SAUSA300_1455
SAUSA300_2069	0,79	1,27	0,00091411	Yes	753	865	742	597	582	540	625	650	580	conserved hypothetical protein	SAUSA300_2069
SAUSA300_1196	0,79	1,27	0,001929235	Yes	1061	861	821	785	764	797	744	741	662	RNA chaperone, host factor-1 protein	hfq
SAUSA300_0767	0,78	1,28	0,252467192	No	491	319	275	203	106	127	303	249	290	conserved hypothetical protein	SAUSA300_0767
SAUSA300_0310	0,78	1,28	0,164747716	No	2671	4258	3931	5345	5047	6422	2840	3259	2375	perfringolysin O regulator protein	pfoR
SAUSA300_1048	0,78	1,28	0,019652199	No	1712	1700	1554	888	827	954	1219	1558	1108	succinate dehydrogenase, iron-sulfur prot	sdhB
SAUSA300_1086	0,78	1,28	3,84E-07	Yes	5055	4814	4619	3968	4021	4139	3744	3728	3871	putative cell-division initiation protein	SAUSA300_1086
SAUSA300_1690	0,78	1,28	0,001355882	Yes	973	895	770	779	755	713	739	684	639	putative thioredoxin	SAUSA300_1690
SAUSA300_1256	0,78	1,28	0,148380865	No	865	1309	942	1077	989	1006	779	828	823	peptide methionine sulfoxide reductase M	msrA
SAUSA300_2021	0,78	1,28	3,79E-08	Yes	3117	3263	2897	2582	2723	2614	2409	2475	2377	S1 RNA binding domain protein	SAUSA300_2021
SAUSA300_0204	0,78	1,28	0,002489916	Yes	692	790	638	610	624	590	558	548	550	gamma-glutamyltranspeptidase	ggt
SAUSA300_0369	0,78	1,28	0,270693262	No	47	67	64	33	43	31	37	47	54	conserved hypothetical protein	SAUSA300_0369
SAUSA300_1062	0,78	1,28	0,32739128	No	38	33	37	30	14	34	29	30	25	ornithine carbamoyltransferase	argF
SAUSA300_1752	0,78	1,28	0,184116069	No	133	250	159	183	221	238	131	157	135	type I restriction-modification system, M s	hsdM
SAUSA300_1944	0,78	1,28	0,451635238	No	76	25	30	160	115	169	34	36	29	phi77 ORF026-like protein, putative phage	SAUSA300_1944
SAUSA300_2357	0,78	1,28	0,00010503	Yes	20727	20724	17144	14977	14795	15593	15332	15989	14379	ABC transporter, ATP-binding protein	SAUSA300_2357
SAUSA300_0716	0,78	1,28	0,030343401	No	12895	13734	13891	9284	10386	9301	9887	10952	10699	ribonucleoside-diphosphate reductase, alp	SAUSA300_0716
SAUSA300_1367	0,78	1,28	1,03E-05	Yes	2198	2219	2006	1829	1699	1826	1721	1682	1601	cytidylate kinase	cmk
SAUSA300_2449	0,78	1,28	0,128016836	No	536	757	648	478	503	555	468	649	391	putative transporter	SAUSA300_2449
SAUSA300_0374	0,78	1,28	0,234623416	No	12327	12681	14784	3817	4249	5727	11551	11111	8149	putative membrane protein	SAUSA300_0374
SAUSA300_2169	0,78	1,28	0,007746773	Yes	537	656	582	603	487	586	453	467	463	conserved hypothetical protein	SAUSA300_2169
SAUSA300_0187	0,78	1,28	0,129592376	No	350	501	293	450	451	495	298	305	283	ornithine--oxo-acid transaminase	rocD
SAUSA300_1154	0,78	1,28	0,005220508	Yes	1287	1240	1503	928	1057	967	1001	1076	1059	phosphatidate cytidylyltransferase	cdsA
SAUSA300_2354	0,78	1,28	0,028353902	No	307	338	384	115	151	111	220	279	301	putative lipoprotein	SAUSA300_2354
SAUSA300_0553	0,78	1,29	0,011862184	No	2106	1979	1622	2435	2315	2505	1653	1356	1421	conserved hypothetical protein	SAUSA300_0553
SAUSA300_1372	0,78	1,29	0,303251392	No	251	138	256	64	61	58	147	152	197	conserved hypothetical protein	SAUSA300_1372
SAUSA300_1761	0,78	1,29	0,271912824	No	36	30	58	21	18	37	31	32	35	lantibiotic epidermin immunity protein F	epiE

SAUSA300_0373	0,78	1,29	0,081978143	No	1030	788	859	463	500	413	720	595	754	conserved hypothetical protein	SAUSA300_0373
SAUSA300_0823	0,78	1,29	0,734668701	No	2	1	2	0	2	3	1	1	1	hypothetical protein	SAUSA300_0823
SAUSA300_1517	0,78	1,29	1,34E-07	Yes	2539	2432	2506	1661	1738	1709	1918	1950	1926	endonuclease IV	SAUSA300_1517
SAUSA300_0329	0,78	1,29	0,049737462	No	1190	1373	1190	1098	1080	1390	1011	996	895	putative oxidoreductase	SAUSA300_0329
SAUSA300_0513	0,77	1,29	2,46E-05	Yes	9516	9971	8433	6401	6151	6356	7599	7306	6681	glutamyl-tRNA synthetase	gltX
SAUSA300_0814	0,77	1,29	0,131483626	No	7742	4477	5144	3251	2869	2231	5122	4213	4022	conserved hypothetical protein	SAUSA300_0814
SAUSA300_0494	0,77	1,29	7,77E-05	Yes	1361	1478	1262	1240	1276	1281	1085	1096	985	2-amino-4-hydroxy-6-hydroxymethyldihyc	folK
SAUSA300_1858	0,77	1,30	0,000624696	Yes	1076	1142	979	778	841	843	824	882	760	conserved hypothetical protein	SAUSA300_1858
SAUSA300_0110	0,77	1,30	0,038363349	No	210	223	246	120	122	115	170	167	187	transcriptional regulator, GntR family/amii	SAUSA300_0110
SAUSA300_0740	0,77	1,30	0,143175634	No	493	480	576	255	286	166	371	395	423	conserved hypothetical protein	SAUSA300_0740
SAUSA300_2001	0,77	1,30	0,090722594	No	381	394	357	238	232	207	290	264	314	DNA mismatch repair protein-like protein	SAUSA300_2001
SAUSA300_1483	0,77	1,30	0,023577511	No	126	155	131	120	137	127	103	107	107	conserved hypothetical protein	SAUSA300_1483
SAUSA300_1036	0,77	1,30	0,001707703	Yes	673	697	827	569	563	631	511	613	573	RNA methyltransferase, TrmH family	SAUSA300_1036
SAUSA300_1203	0,77	1,30	NA	NA	0	1	11	3	5	1	1	0	6	conserved hypothetical protein	SAUSA300_1203
SAUSA300_2232	0,77	1,30	0,000146734	Yes	565	661	625	517	532	550	490	468	468	acetyltransferase, GNAT family	SAUSA300_2232
SAUSA300_1329	0,77	1,30	0,362752418	No	43	59	89	43	47	53	44	55	46	amino acid permease	SAUSA300_1329
SAUSA300_1932	0,77	1,30	0,320498809	No	60	34	32	155	178	217	22	38	34	conserved hypothetical phage protein	SAUSA300_1932
SAUSA300_0184	0,77	1,30	0,044216246	No	327	327	251	249	273	278	225	241	224	acetylglutamate kinase	argB
SAUSA300_1300	0,77	1,30	0,019414456	No	1976	1630	1799	1077	1269	855	1404	1301	1429	branched-chain amino acid transport syste	brnQ
SAUSA300_0822	0,77	1,30	1,62E-05	Yes	10417	10676	9071	13812	13738	12960	8041	7704	7362	FeS assembly protein SufB	sufB
SAUSA300_2343	0,77	1,30	0,60985342	No	59	37	209	47	54	61	30	145	49	respiratory nitrate reductase, alpha subun	SAUSA300_2343
SAUSA300_0952	0,77	1,31	0,001032032	Yes	2258	2031	2014	1806	2026	1656	1675	1564	1578	aminotransferase, class I	SAUSA300_0952
SAUSA300_0274	0,77	1,31	0,101224995	No	3567	2175	3338	939	856	668	2228	2333	2355	conserved hypothetical protein	SAUSA300_0274
SAUSA300_0098	0,77	1,31	0,011473718	No	298	301	357	126	122	123	257	225	250	conserved hypothetical protein	SAUSA300_0098
SAUSA300_0027	0,77	1,31	0,031746232	No	934	1010	821	646	674	609	711	703	695	conserved hypothetical protein	SAUSA300_0027
SAUSA300_0384	0,77	1,31	0,003428727	Yes	885	804	711	528	458	429	577	611	640	conserved hypothetical protein	SAUSA300_0384
SAUSA300_0403	0,76	1,31	0,39443185	No	28	22	25	20	38	16	17	18	19	exotoxin	SAUSA300_0403
SAUSA300_1588	0,76	1,31	0,002198493	Yes	5997	5905	4686	4263	3877	3991	4513	4177	3952	N-acetylmuramoyl-L-alanine amidase	lytH
SAUSA300_2507	0,76	1,31	0,130175679	No	91	78	71	74	86	86	64	57	60	regulatory protein-like protein	SAUSA300_2507
SAUSA300_1103	0,76	1,31	7,00E-06	Yes	2286	2304	2636	1203	1215	1192	1778	1846	1877	DNA-directed RNA polymerase, omega sul	rpoZ
SAUSA300_0198	0,76	1,31	0,080018298	No	422	486	413	205	180	117	309	338	354	conserved hypothetical protein	SAUSA300_0198
SAUSA300_2337	0,76	1,31	0,001852532	Yes	1028	1057	782	580	566	620	741	737	694	transcriptional regulator, DegU family	SAUSA300_2337
SAUSA300_2397	0,76	1,32	0,035527786	No	3080	2357	2415	1956	2083	1866	2206	1874	1854	putative transport protein	SAUSA300_2397
SAUSA300_1521	0,76	1,32	0,000106199	Yes	7997	6935	6389	5574	5876	5467	5571	5428	5146	RNA polymerase sigma factor RpoD	rpoD
SAUSA300_2552	0,76	1,32	0,088188323	No	193	194	137	185	151	210	130	134	129	citrate transporter, permease protein	SAUSA300_2552
SAUSA300_2094	0,76	1,32	0,001021136	Yes	3359	3528	2938	2026	1666	1706	2466	2340	2619	conserved hypothetical protein	SAUSA300_2094
SAUSA300_0313	0,76	1,32	0,28062096	No	36	36	39	37	79	41	28	28	26	putative nucleoside permease NupC	SAUSA300_0313
SAUSA300_0441	0,76	1,32	0,011662281	No	811	901	679	886	868	901	602	660	542	acetyltransferase, GNAT family	SAUSA300_0441
SAUSA300_1001	0,76	1,32	0,269962041	No	381	293	418	135	169	114	242	217	356	spermidine/putrescine ABC transporter, p	potC
SAUSA300_1187	0,76	1,32	0,000724376	Yes	1721	1670	1334	1391	1359	1301	1187	1211	1166	conserved hypothetical protein	SAUSA300_1187
SAUSA300_0820	0,76	1,32	0,024279745	No	8124	9260	6794	14053	13674	15703	6411	6316	5507	cysteine desulfurases, SufS subfamily subf	sufS
SAUSA300_1409	0,76	1,32	0,508581571	No	14	12	14	1	11	12	10	6	13	conserved hypothetical phage protein	SAUSA300_1409
SAUSA300_0012	0,76	1,32	0,048353536	No	589	753	497	670	604	682	524	448	413	putative homoserine O-acetyltransferase	SAUSA300_0012
SAUSA300_0151	0,76	1,32	0,417304266	No	293	329	391	339	430	410	212	341	197	alcohol dehydrogenase, iron-containing	adhE
SAUSA300_2411	0,76	1,32	0,007210074	Yes	424	601	550	740	739	797	355	430	405	oligopeptide permease, peptide-binding p	opp-1A
SAUSA300_1040	0,75	1,32	0,000812071	Yes	496	433	504	289	259	241	354	336	388	conserved hypothetical protein	SAUSA300_1040
SAUSA300_0473	0,75	1,32	2,26E-07	Yes	3047	2964	3296	1806	1803	1634	2233	2460	2330	pur operon repressor	purR
SAUSA300_1391	0,75	1,32	0,258435841	No	40	48	37	24	20	27	38	27	28	phiSLT ORF527-like protein	SAUSA300_1391
SAUSA300_0105	0,75	1,33	0,109467905	No	351	523	343	527	433	688	319	308	285	peptidase, M20/M25/M40 family	SAUSA300_0105
SAUSA300_0177	0,75	1,33	0,096969132	No	5341	6190	4803	3307	2939	3233	3819	3853	4546	conserved hypothetical protein	SAUSA300_0177
SAUSA300_1549	0,75	1,33	0,010575997	No	260	270	282	169	219	186	193	212	204	ComE operon protein 1	SAUSA300_1549
SAUSA300_2174	0,75	1,33	0,000117445	Yes	1002	1047	1016	789	893	827	790	745	767	cobalt transport family protein	SAUSA300_2174
SAUSA300_2554	0,75	1,33	0,049379624	No	1216	1830	1040	1657	1582	1678	1057	994	1004	sulfite reductase flavoprotein	SAUSA300_2554
SAUSA300_1037	0,75	1,33	0,00013559	Yes	1848	2025	1896	1309	1319	1260	1395	1506	1425	phenylalanyl-tRNA synthetase, alpha subu	pheS
SAUSA300_1897	0,75	1,33	0,05421011	No	546	650	601	454	385	484	450	435	458	sodium-dependent transporter	SAUSA300_1897
SAUSA300_0602	0,75	1,33	0,005220508	Yes	5670	4327	5418	4332	4186	4217	4018	4146	3368	conserved hypothetical protein	SAUSA300_0602
SAUSA300_1000	0,75	1,33	0,089214383	No	222	205	226	115	104	93	150	147	189	spermidine/putrescine ABC transporter, p	potB
SAUSA300_0821	0,75	1,34	0,005736864	Yes	3252	3621	2748	5430	5418	6357	2590	2340	2253	SUF system FeS assembly protein, NifU far	SAUSA300_0821
SAUSA300_0058	0,75	1,34	0,550297947	No	10	12	7	7	7	6	7	4	9	conserved hypothetical protein	SAUSA300_0058

SAUSA300_1321	0,75	1,34	0,000957256	Yes	1123	1240	930	1133	985	1241	874	798	782	conserved hypothetical protein	SAUSA300_1321
SAUSA300_1743	0,75	1,34	0,298334487	No	78	44	82	32	20	18	42	68	39	conserved hypothetical protein	SAUSA300_1743
SAUSA300_0265	0,75	1,34	0,039688376	No	587	600	463	394	417	528	410	457	358	putative ribose operon repressor	SAUSA300_0265
SAUSA300_0186	0,75	1,34	0,026625238	No	398	494	326	341	340	333	317	307	280	N-acetyl-gamma-glutamyl-phosphate redu	argC
SAUSA300_1694	0,75	1,34	0,000423563	Yes	3844	3236	3350	2079	2026	2119	2533	2516	2712	tRNA (guanine-N(7)-)-methyltransferase	trmB
SAUSA300_1371	0,75	1,34	5,29E-05	Yes	1455	1562	1300	1070	1073	896	1085	1074	1054	ATP-dependent DNA helicase RecQ	recQ
SAUSA300_1280	0,74	1,34	0,139866656	No	55	51	50	30	36	46	35	34	45	phosphate ABC transporter, ATP-binding p	pstB
SAUSA300_0176	0,74	1,34	0,041085627	No	2716	3308	2536	1800	1535	1782	2104	1981	2260	ABC transporter, permease protein	SAUSA300_0176
SAUSA300_1407	0,74	1,34	0,199935958	No	50	49	37	27	23	44	34	33	33	phi SLT ORF 145-like protein, phage transc	SAUSA300_1407
SAUSA300_2541	0,74	1,34	0,000263803	Yes	56601	59300	50638	48183	48856	52914	41657	44958	37147	malate:quinone-oxidoreductase	mqo
SAUSA300_1059	0,74	1,34	0,445213884	No	17	16	30	17	11	22	19	11	16	putative exotoxin 1	SAUSA300_1059
SAUSA300_2577	0,74	1,34	0,000449404	Yes	934	965	823	713	791	736	635	747	637	mannose-6-phosphate isomerase, class I	manA
SAUSA300_1629	0,74	1,34	0,010000021	No	10723	11424	8958	7159	6681	6978	7852	7953	7262	threonyl-tRNA synthetase	thrS
SAUSA300_1953	0,74	1,35	0,593297133	No	9	1	4	21	23	25	4	2	4	phiPVL ORF051-like protein	SAUSA300_1953
SAUSA300_1630	0,74	1,35	2,59E-05	Yes	2256	2697	2395	1935	1695	1791	1869	1834	1753	primosomal protein DnaI	dnaI
SAUSA300_2569	0,74	1,35	0,350857036	No	12	40	16	27	36	31	16	15	19	ornithine carbamoyltransferase	arcB
SAUSA300_0468	0,74	1,35	0,003795049	Yes	4097	5293	4101	5100	4814	5390	3595	3290	3094	hydrolase, TatD family	SAUSA300_0468
SAUSA300_1518	0,74	1,35	1,30E-09	Yes	4061	3914	3871	2543	2572	2505	3089	2816	2858	ATP-dependent RNA helicase, DEAD/DEAH	SAUSA300_1518
SAUSA300_0216	0,74	1,35	0,353441298	No	45	56	51	37	59	59	46	31	35	hexose phosphate transport protein	uhpT
SAUSA300_0937	0,74	1,35	0,323750573	No	375	329	613	34	122	59	377	281	301	conserved hypothetical protein	SAUSA300_0937
SAUSA300_1949	0,74	1,35	0,521734497	No	12	4	9	36	38	40	7	6	5	dUTP diphosphatase	dut
SAUSA300_2627	0,74	1,35	0,000243819	Yes	2943	2717	2239	1932	1954	2020	1968	2102	1763	2-oxoglutarate/malate translocator	SAUSA300_2627
SAUSA300_0582	0,74	1,35	0,069658681	No	177	164	160	126	110	104	142	102	124	conserved hypothetical protein	SAUSA300_0582
SAUSA300_1632	0,74	1,35	0,000239288	Yes	997	1127	1020	765	762	807	770	798	752	conserved hypothetical protein	SAUSA300_1632
SAUSA300_0595	0,74	1,35	0,011871959	No	637	954	860	513	390	544	605	590	613	conserved hypothetical protein	SAUSA300_0595
SAUSA300_1185	0,74	1,35	1,59E-05	Yes	4292	5162	4396	3460	3392	2935	3417	3333	3467	tRNA-i(6)A37 thiotransferase enzyme MiaB	miaB
SAUSA300_2373	0,74	1,35	0,470222144	No	12	11	18	10	14	15	9	9	10	dethiobiotin synthase	bioD
SAUSA300_1002	0,74	1,36	0,01112892	No	1497	1484	1273	1107	886	1176	1082	969	1074	spermidine/putrescine ABC transporter, sp	potD
SAUSA300_2355	0,74	1,36	0,243797067	No	41	48	93	34	36	56	32	49	53	putative lipoprotein	SAUSA300_2355
SAUSA300_1701	0,74	1,36	0,000590516	Yes	889	995	932	849	872	967	682	659	731	conserved hypothetical protein	SAUSA300_1701
SAUSA300_1695	0,74	1,36	3,48E-05	Yes	7458	6940	6277	5839	5569	6248	5075	5209	4928	conserved hypothetical protein	SAUSA300_1695
SAUSA300_0956	0,74	1,36	0,011014689	No	264	255	282	272	356	311	191	199	198	conserved hypothetical protein	SAUSA300_0956
SAUSA300_2525	0,74	1,36	0,085746773	No	377	480	359	398	295	509	331	298	262	conserved hypothetical protein	SAUSA300_2525
SAUSA300_0314	0,74	1,36	0,270514382	No	40	36	53	16	32	22	27	34	31	sodium:solute symporter family protein	SAUSA300_0314
SAUSA300_2281	0,74	1,36	0,065430562	No	832	1051	866	768	591	1037	726	683	601	formimidoylglutamase	hutG
SAUSA300_0111	0,73	1,36	0,019422248	No	305	242	246	125	124	84	196	177	204	conserved hypothetical protein	SAUSA300_0111
SAUSA300_0175	0,73	1,36	0,038582625	No	3653	4939	3364	2814	2576	3163	2660	2769	3310	putative lipoprotein	SAUSA300_0175
SAUSA300_0097	0,73	1,36	9,32E-06	Yes	839	1018	914	685	638	677	683	660	693	conserved hypothetical protein	SAUSA300_0097
SAUSA300_2070	0,73	1,36	7,17E-06	Yes	1111	1248	1153	1124	1147	1056	872	888	819	conserved hypothetical protein	SAUSA300_2070
SAUSA300_0576	0,73	1,36	0,14402983	No	1428	1474	790	1396	1046	1229	1092	773	820	putative Pyridine nucleotide-disulphide ox	SAUSA300_0576
SAUSA300_2285	0,73	1,36	0,000336505	Yes	505	663	622	461	455	427	436	431	449	aldose 1-epimerase	galM
SAUSA300_1268	0,73	1,36	0,06533892	No	119	127	90	95	122	105	75	79	89	tryptophan synthase, alpha subunit	trpA
SAUSA300_1513	0,73	1,37	0,016346287	No	21429	20890	14491	26403	25924	26306	16059	14355	11038	superoxide dismutase (Mn/Fe family)	SAUSA300_1513
SAUSA300_0390	0,73	1,37	0,157584094	No	53	60	43	36	45	33	36	42	36	conserved hypothetical protein	SAUSA300_0390
SAUSA300_1284	0,73	1,37	0,001842773	Yes	5954	4428	4667	3272	3079	3037	3782	3847	3353	conserved hypothetical protein	SAUSA300_1284
SAUSA300_2502	0,73	1,37	0,169822712	No	52	44	54	56	38	38	24	45	39	conserved hypothetical protein	SAUSA300_2502
SAUSA300_1063	0,73	1,37	0,085806529	No	57	51	71	40	50	30	43	44	44	carbamate kinase	arcC
SAUSA300_0168	0,73	1,37	0,083829665	No	126	178	174	113	115	114	98	119	130	conserved hypothetical protein	SAUSA300_0168
SAUSA300_0273	0,73	1,37	0,001415691	Yes	665	513	481	241	261	228	403	408	392	putative membrane protein	SAUSA300_0273
SAUSA300_0935	0,73	1,37	0,344796549	No	33	33	23	13	18	19	19	15	28	conserved hypothetical protein	SAUSA300_0935
SAUSA300_1793	0,73	1,37	0,002642351	Yes	3007	2814	2795	1340	1328	1013	1940	2176	2156	conserved hypothetical protein	SAUSA300_1793
SAUSA300_1899	0,73	1,37	0,000501728	Yes	4538	4547	4395	3123	3347	2910	3305	3430	3069	conserved hypothetical protein	SAUSA300_1899
SAUSA300_1312	0,73	1,37	0,022252267	No	558	779	586	584	568	665	486	475	434	acetyltransferase, GNAT family	SAUSA300_1312
SAUSA300_1740	0,73	1,37	0,002716594	Yes	2794	2690	2629	1585	1515	1482	1675	2415	1802	conserved hypothetical protein	SAUSA300_1740
SAUSA300_1355	0,73	1,37	4,50E-05	Yes	2868	3270	2625	2187	2445	2202	2161	2073	2130	3-phosphoshikimate 1-carboxyvinyltransfe	aroA
SAUSA300_0981	0,73	1,38	6,29E-06	Yes	751	825	812	438	500	416	578	583	576	conserved hypothetical protein	SAUSA300_0981
SAUSA300_2207	0,73	1,38	0,017531516	No	14910	18280	10278	6870	6306	7159	11569	10021	9857	xanthine/uracil permease family protein	SAUSA300_2207
SAUSA300_1377	0,73	1,38	0,487047295	No	12	11	11	3	7	3	8	6	9	conserved hypothetical protein	SAUSA300_1377
SAUSA300_2296	0,73	1,38	0,00010503	Yes	2840	3073	2447	2478	2378	2487	2058	2066	1932	esterase-like protein	SAUSA300_2296

SAUSA300_0819	0,73	1,38	0,000313865	Yes	6554	7546	5947	9926	10494	10295	5107	4898	4509	FeS assembly protein SufD	sufD
SAUSA300_1136	0,73	1,38	0,003461546	Yes	494	617	604	432	320	441	443	404	398	putative GTP-binding protein	SAUSA300_1136
SAUSA300_2036	0,73	1,38	0,022109411	No	179	207	153	127	92	109	155	108	126	DNA-binding response regulator, KdpE	kdpE
SAUSA300_1631	0,72	1,38	4,61E-06	Yes	2134	2476	2304	1555	1519	1631	1580	1773	1654	replication initiation and membrane attacl	SAUSA300_1631
SAUSA300_2168	0,72	1,38	0,01691214	No	348	316	358	155	207	148	224	214	298	conserved hypothetical protein	SAUSA300_2168
SAUSA300_2176	0,72	1,38	0,00011487	Yes	1082	1250	1087	1012	969	1119	900	784	789	ABC transporter, ATP-binding protein	SAUSA300_2176
SAUSA300_1805	0,72	1,38	5,01E-05	Yes	424	465	441	262	246	269	324	315	323	RNA methyltransferase	SAUSA300_1805
SAUSA300_0067	0,72	1,38	0,547419415	No	117	42	180	39	18	22	67	52	110	universal stress protein family	SAUSA300_0067
SAUSA300_1202	0,72	1,38	0,638966779	No	3	0	9	4	0	0	2	1	3	conserved hypothetical protein	SAUSA300_1202
SAUSA300_0701	0,72	1,38	0,033744552	No	515	534	672	198	225	124	403	367	467	conserved hypothetical protein TIGR0037(	SAUSA300_0701
SAUSA300_0370	0,72	1,38	0,109467905	No	195	156	91	97	65	81	117	95	101	putative staphylococcal enterotoxin	SAUSA300_0370
SAUSA300_0696	0,72	1,39	0,018861761	No	444	446	422	229	376	216	282	344	316	6-pyruvoyl tetrahydrobiopterin synthase-l	SAUSA300_0696
SAUSA300_0371	0,72	1,39	0,45968966	No	16	14	8	4	16	9	9	7	9	conserved hypothetical protein	SAUSA300_0371
SAUSA300_0695	0,72	1,39	0,002405056	Yes	973	953	898	822	1111	940	631	758	639	radical activating enzyme family protein	SAUSA300_0695
SAUSA300_0107	0,72	1,39	0,001999553	Yes	7540	9043	7329	4896	5297	5301	5632	6189	5346	Na/Pi cotransporter family protein	SAUSA300_0107
SAUSA300_1590	0,72	1,39	0,000351342	Yes	11149	13471	10426	8819	8319	8209	8456	8844	7868	GTP pyrophosphokinase	SAUSA300_1590
SAUSA300_2273	0,72	1,39	0,026691617	No	1280	2115	1755	1501	1352	1822	1252	1262	1174	Na+/H+ antiporter family protein	SAUSA300_2273
SAUSA300_0570	0,72	1,39	1,38E-05	Yes	9402	9832	7618	7538	7100	7934	6762	6726	5789	phosphate acetyltransferase	pta
SAUSA300_1325	0,72	1,39	0,090722594	No	67	52	47	53	56	58	40	48	30	conserved hypothetical protein	SAUSA300_1325
SAUSA300_2338	0,72	1,39	0,000397544	Yes	1178	1487	1112	812	834	837	821	928	956	sensor histidine kinase	SAUSA300_2338
SAUSA300_2564	0,72	1,39	0,001504198	Yes	431	580	426	318	307	303	351	335	343	tributyrin esterase	estA
SAUSA300_2359	0,72	1,39	3,46E-07	Yes	11493	12430	11258	6960	7084	6459	8334	8422	8465	amino acid ABC transporter, amino acid-bi	SAUSA300_2359
SAUSA300_1796	0,72	1,39	0,009471842	Yes	971	678	747	352	311	266	602	513	594	conserved hypothetical protein	SAUSA300_1796
SAUSA300_2358	0,72	1,40	8,53E-12	Yes	12093	12458	10881	9095	9072	9418	8387	8742	8247	ABC transporter, permease protein	SAUSA300_2358
SAUSA300_0255	0,72	1,40	7,68E-06	Yes	782	832	793	547	491	481	583	554	586	sensory transduction protein LytR	SAUSA300_0255
SAUSA300_1330	0,72	1,40	0,45968966	No	21	30	69	43	52	44	21	38	24	threonine dehydratase	ilvA
SAUSA300_1435	0,72	1,40	0,065060275	No	899	1059	725	609	485	458	714	586	606	phiSLT ORF153-like protein	SAUSA300_1435
SAUSA300_0200	0,72	1,40	0,030083179	No	653	939	543	594	647	633	458	538	522	peptide ABC transporter, ATP-binding prot	SAUSA300_0200
SAUSA300_1955	0,71	1,40	0,569846989	No	16	3	1	21	9	24	4	4	4	putative endodeoxyribonuclease RusA	SAUSA300_1955
SAUSA300_1481	0,71	1,40	0,01122058	No	295	256	193	222	201	198	195	184	147	putative membrane protein	SAUSA300_1481
SAUSA300_0038	0,71	1,40	0,015681347	No	153	130	106	150	147	169	96	93	86	cassette chromosome recombinase A	ccrA
SAUSA300_0272	0,71	1,40	0,030156979	No	739	427	470	256	241	177	375	384	398	conserved hypothetical protein	SAUSA300_0272
SAUSA300_0472	0,71	1,40	1,63E-05	Yes	3016	2898	3145	1948	1880	1840	2090	2164	2183	4-diphosphocytidyl-2C-methyl-D-erythrito	ispE
SAUSA300_2590	0,71	1,41	0,005413848	Yes	966	894	617	740	766	853	628	579	545	conserved hypothetical protein	SAUSA300_2590
SAUSA300_1252	0,71	1,41	0,000240208	Yes	4194	5003	4721	3921	4080	4398	3432	3327	3108	amino acid carrier protein	SAUSA300_1252
SAUSA300_2251	0,71	1,41	0,023875612	No	3204	3859	3073	1953	1941	1940	2519	2448	2189	dehydrogenase family protein	SAUSA300_2251
SAUSA300_0863	0,71	1,41	0,000441562	Yes	574	638	552	427	347	401	392	437	419	argininosuccinate lyase	argH
SAUSA300_2080	0,71	1,41	0,002347778	Yes	1133	819	1013	361	316	277	721	662	711	conserved hypothetical protein	SAUSA300_2080
SAUSA300_0980	0,71	1,41	6,07E-09	Yes	4948	4223	4280	3070	2982	2946	3080	3306	3142	putative membrane protein	SAUSA300_0980
SAUSA300_0195	0,71	1,41	0,023995733	No	79	78	77	53	43	47	48	65	53	RpiR family transcriptional regulator	SAUSA300_0195
SAUSA300_0261	0,71	1,41	0,028671776	No	186	138	123	180	110	203	103	105	104	conserved hypothetical protein	SAUSA300_0261
SAUSA300_1591	0,71	1,41	0,000176865	Yes	1810	1744	1633	1232	1314	1405	1246	1285	1134	adenine phosphoribosyltransferase	apt
SAUSA300_0666	0,71	1,41	0,016965649	No	2210	2155	1567	1764	1497	1937	1512	1444	1211	decarboxylase family protein	SAUSA300_0666
SAUSA300_0702	0,71	1,42	0,000813505	Yes	1886	2594	1784	1577	1571	1508	1521	1446	1442	urea amidolyase-related protein	SAUSA300_0702
SAUSA300_2252	0,70	1,42	0,037997473	No	661	359	422	145	142	102	370	303	333	conserved hypothetical protein	SAUSA300_2252
SAUSA300_2167	0,70	1,42	0,010711213	No	441	285	329	158	203	182	249	214	272	conserved hypothetical protein	SAUSA300_2167
SAUSA300_1213	0,70	1,42	0,621570366	No	3	4	1	7	0	6	1	4	0	conserved hypothetical protein	SAUSA300_1213
SAUSA300_0174	0,70	1,42	0,042024763	No	3142	4287	2917	2170	1889	2082	2127	2319	2779	conserved hypothetical protein	SAUSA300_0174
SAUSA300_0119	0,70	1,42	0,14402983	No	48	60	53	331	419	552	42	24	45	ornithine cyclodeaminase	SAUSA300_0119
SAUSA300_0356	0,70	1,42	9,79E-05	Yes	670	684	623	497	478	519	481	493	412	conserved hypothetical protein	SAUSA300_0356
SAUSA300_2593	0,70	1,42	0,010264744	No	565	652	475	365	370	444	390	398	394	conserved hypothetical protein	SAUSA300_2593
SAUSA300_0185	0,70	1,43	0,023623607	No	663	847	552	669	714	744	525	491	421	arginine biosynthesis bifunctional protein	argJ
SAUSA300_0545	0,70	1,43	0,001032032	Yes	1547	1482	1229	874	789	597	1042	932	999	Flavodoxin-like fold	SAUSA300_0545
SAUSA300_0120	0,70	1,43	0,035584029	No	98	138	105	467	509	646	84	64	90	siderophore biosynthesis protein, IucC fan	sbnC
SAUSA300_1102	0,70	1,43	3,04E-07	Yes	4475	4887	5145	3252	3113	3278	3511	3480	3156	guanylate kinase	gmk
SAUSA300_1787	0,70	1,43	0,071086365	No	503	486	748	162	137	118	363	390	452	HIT family protein	SAUSA300_1787
SAUSA300_1698	0,70	1,43	0,001341021	Yes	2213	1764	2135	1050	980	986	1473	1349	1442	conserved hypothetical protein	SAUSA300_1698
SAUSA300_1207	0,70	1,43	0,107468345	No	36	71	50	43	43	38	34	42	34	conserved hypothetical protein	SAUSA300_1207
SAUSA300_1081	0,70	1,43	1,03E-06	Yes	1059	1144	1077	652	636	565	709	764	816	conserved hypothetical protein	SAUSA300_1081

SAUSA300_0585	0,70	1,43	0,137362928	No	60	44	49	27	29	24	36	35	33	conserved hypothetical protein	SAUSA300_0585
SAUSA300_1432	0,70	1,43	0,274661139	No	834	397	830	53	110	31	452	432	515	phiSLT ORF78-like protein	SAUSA300_1432
SAUSA300_0940	0,70	1,44	0,013030839	No	2756	2493	2900	1140	1285	942	2074	1949	1624	conserved hypothetical protein	SAUSA300_0940
SAUSA300_2276	0,70	1,44	0,020065931	No	184	335	256	186	178	225	180	182	177	peptidase, M20/M25/M40 family	SAUSA300_2276
SAUSA300_0584	0,70	1,44	0,034336968	No	133	96	119	39	56	27	85	68	86	conserved hypothetical protein	SAUSA300_0584
SAUSA300_1673	0,70	1,44	0,000134485	Yes	952	1028	1002	566	563	642	603	665	799	1-acyl-sn-glycerol-3-phosphate acyltransfe	SAUSA300_1673
SAUSA300_1751	0,70	1,44	8,34E-05	Yes	608	795	638	510	426	525	498	474	446	type I restriction-modification enzyme, S s	hsdS
SAUSA300_0594	0,70	1,44	0,35583495	No	348	438	797	405	410	460	261	516	282	alcohol dehydrogenase	adh
SAUSA300_0194	0,69	1,44	0,019799384	No	65	86	93	59	74	80	54	59	59	sucrose-specific PTS tranporter protein	SAUSA300_0194
SAUSA300_0736	0,69	1,44	0,005413488	Yes	3223	3204	2532	1408	1032	1125	1888	2299	2007	ribosomal subunit interface protein	yfiA
SAUSA300_1653	0,69	1,44	0,00482437	Yes	1333	1688	1320	1002	791	945	1165	955	882	conserved hypothetical protein	SAUSA300_1653
SAUSA300_0761	0,69	1,44	0,337638161	No	133	73	268	42	41	35	84	91	143	conserved hypothetical protein	SAUSA300_0761
SAUSA300_1657	0,69	1,44	1,31E-08	Yes	12418	11731	10658	8053	7282	7627	8200	8449	7465	acetate kinase	ackA
SAUSA300_1293	0,69	1,44	2,72E-05	Yes	1426	1611	1502	1160	1244	1236	1058	974	1109	diaminopimelate decarboxylase	lysA
SAUSA300_1438	0,69	1,44	0,033078634	No	189	182	137	120	77	101	132	102	114	phiSLT ORF401-like protein, integrase	SAUSA300_1438
SAUSA300_1755	0,69	1,44	0,457713432	No	5	14	6	3	14	9	6	5	5	serine protease SplD	splD
SAUSA300_2633	0,69	1,44	0,152324315	No	34	21	28	40	34	30	16	23	18	ABC transporter, ATP-binding protein	SAUSA300_2633
SAUSA300_2537	0,69	1,44	0,003551485	Yes	3166	3701	2513	3915	3586	4109	2267	2307	1887	L-lactate dehydrogenase	SAUSA300_2537
SAUSA300_0566	0,69	1,45	0,028631349	No	7611	7749	8339	3401	3275	4176	5358	5918	4994	amino acid permease	SAUSA300_0566
SAUSA300_1929	0,69	1,45	0,152107602	No	38	26	27	137	110	145	19	25	18	phi77 ORF004-like protein, putative phage	SAUSA300_1929
SAUSA300_0567	0,69	1,45	0,00338657	Yes	739	775	595	405	469	418	481	514	451	conserved hypothetical protein	SAUSA300_0567
SAUSA300_2250	0,69	1,45	0,010901687	No	3593	4096	3015	2125	2078	2354	2747	2344	2245	Na+/H+ antiporter NhaC	nhaC
SAUSA300_1664	0,69	1,45	1,19E-06	Yes	11393	9720	10925	5704	5506	4977	7044	7612	7389	septation ring formation regulator EzrA	ezrA
SAUSA300_1956	0,69	1,45	0,502074428	No	12	4	4	34	14	31	2	6	3	conserved hypothetical phage protein	SAUSA300_1956
SAUSA300_0337	0,69	1,45	0,000307682	Yes	563	538	508	344	313	352	362	409	333	glycerol-3-phosphate transporter	glpT
SAUSA300_1965	0,69	1,45	0,571148031	No	16	7	97	4	5	0	11	11	49	conserved hypothetical phage protein	SAUSA300_1965
SAUSA300_1345	0,69	1,45	1,80E-05	Yes	10644	11439	8585	7935	7569	7587	7227	7254	6590	asparaginyl-tRNA synthetase	asnS
SAUSA300_1032	0,69	1,45	0,048761673	No	115	231	167	235	259	291	120	101	130	putative iron compound ABC transporter,	SAUSA300_1032
SAUSA300_2539	0,69	1,45	0,124067852	No	62	100	99	69	77	157	53	75	51	aminotransferase	SAUSA300_2539
SAUSA300_1265	0,69	1,46	0,380244444	No	9	16	20	11	16	16	4	12	13	indole-3-glycerol phosphate synthase	trpC
SAUSA300_0955	0,69	1,46	0,00297607	Yes	32811	34227	29419	23476	29141	28373	20274	23060	22607	autolysin	atl
SAUSA300_1260	0,69	1,46	2,49E-05	Yes	971	991	838	511	658	544	603	688	625	prephenate dehydrogenase	SAUSA300_1260
SAUSA300_1502	0,69	1,46	0,414519626	No	17	5	8	6	5	7	6	6	7	putative competence protein ComGC	SAUSA300_1502
SAUSA300_1896	0,69	1,46	6,73E-05	Yes	1009	913	1012	573	566	521	635	659	712	prephenate dehydratase	pheA
SAUSA300_1980	0,69	1,46	0,121409483	No	38	41	55	10	20	16	28	28	34	acetyltransferase, GNAT family	SAUSA300_1980
SAUSA300_1183	0,68	1,46	8,07E-05	Yes	2210	2345	1754	2705	2180	2638	1491	1296	1506	pyruvate ferredoxin oxidoreductase, beta	SAUSA300_1183
SAUSA300_2044	0,68	1,47	3,25E-09	Yes	2379	2568	2104	1643	1630	1594	1612	1594	1593	cardiolipin synthetase	cls
SAUSA300_1745	0,68	1,47	0,343748559	No	14	18	29	7	14	16	14	12	15	conserved hypothetical protein	SAUSA300_1745
SAUSA300_0571	0,68	1,47	6,95E-06	Yes	6831	6465	5554	4024	3532	3372	4366	4169	4260	lipoate-protein ligase A family protein	SAUSA300_0571
SAUSA300_2305	0,68	1,47	0,551400719	No	12	4	11	3	2	4	7	2	7	transposase, truncation	SAUSA300_2305
SAUSA300_2256	0,68	1,47	0,002708659	Yes	3162	3707	2807	2284	2385	2484	2276	2362	1911	putative N-acetylmuramoyl-L-alanine amic	SAUSA300_2256
SAUSA300_2106	0,68	1,47	0,005625983	Yes	921	957	825	380	354	476	650	620	557	putative transcriptional regulator	SAUSA300_2106
SAUSA300_0230	0,68	1,47	0,008464184	Yes	920	1445	1205	1021	1010	1153	779	806	830	putative membrane protein	SAUSA300_0230
SAUSA300_1970	0,68	1,47	0,00483831	Yes	630	691	517	206	165	206	428	353	460	putative exonuclease	SAUSA300_1970
SAUSA300_1672	0,68	1,47	1,77E-15	Yes	3021	3170	3016	1978	1884	1851	1963	2159	2127	phosphotransferase system, N-acetylgluco	nagE
SAUSA300_1052	0,68	1,47	0,093373572	No	5102	2004	2501	1320	1826	1150	2441	2216	1766	fibrinogen-binding protein	SAUSA300_1052
SAUSA300_2097	0,68	1,47	0,032996429	No	574	775	791	377	298	566	506	487	447	conserved hypothetical protein	SAUSA300_2097
SAUSA300_0880	0,68	1,48	0,533846746	No	5	3	21	0	11	1	5	5	8	conserved hypothetical protein	SAUSA300_0880
SAUSA300_0667	0,68	1,48	0,006659084	Yes	1474	1580	1055	971	832	846	1034	945	786	YaiI/YqxD family protein	SAUSA300_0667
SAUSA300_0818	0,68	1,48	0,000441562	Yes	4941	5694	4438	7148	6762	7009	3721	3460	2989	FeS assembly ATPase SufC	sufC
SAUSA300_1613	0,68	1,48	0,008689736	Yes	351	341	419	381	363	319	244	225	278	putative abrB protein	SAUSA300_1613
SAUSA300_1279	0,68	1,48	0,022709069	No	86	79	88	33	61	47	59	52	59	phosphate transport system regulatory pr	phoU
SAUSA300_2439	0,68	1,48	0,000176331	Yes	1502	1840	1364	1050	1089	1109	1060	1111	1001	UTP-glucose-1-phosphate uridylyltransfera	galU
SAUSA300_2576	0,68	1,48	0,00281629	Yes	1345	1470	1280	924	1041	1062	935	1025	795	phosphotransferase system, fructose-spec	SAUSA300_2576
SAUSA300_2034	0,68	1,48	0,255131236	No	10	22	22	23	32	28	16	9	11	K+-transporting ATPase, A subunit	kdpA
SAUSA300_0769	0,67	1,48	0,044464785	No	2334	1251	1265	484	379	291	1179	1029	1027	putative lipoprotein	SAUSA300_0769
SAUSA300_1739	0,67	1,48	0,000177439	Yes	1963	1838	1961	941	951	1003	1103	1517	1252	conserved hypothetical protein	SAUSA300_1739
SAUSA300_1375	0,67	1,48	0,512292756	No	7	4	15	4	0	0	4	4	7	conserved hypothetical protein	SAUSA300_1375
SAUSA300_2508	0,67	1,48	0,482511691	No	0	1	4	0	2	0	1	1	0	conserved hypothetical protein	SAUSA300_2508

SAUSA300_1064	0,67	1,49	0,009963661	Yes	315	363	298	241	210	281	239	228	186	transporter, TRAP family	SAUSA300_1064
SAUSA300_1500	0,67	1,49	0,514542716	No	2	7	5	6	2	3	1	4	3	putative competence protein ComYC	SAUSA300_1500
SAUSA300_2283	0,67	1,49	4,09E-07	Yes	768	953	855	579	541	637	575	611	548	ribose 5-phosphate isomerase A	rpiA
SAUSA300_1412	0,67	1,49	0,508581571	No	3	4	2	1	0	1	1	2	3	phiSLT ORF 50-like protein	SAUSA300_1412
SAUSA300_0148	0,67	1,49	0,530820397	No	2	0	0	0	2	3	0	0	0	conserved hypothetical protein	SAUSA300_0148
SAUSA300_2269	0,67	1,49	0,118806205	No	264	182	341	39	68	28	160	156	201	conserved hypothetical protein	SAUSA300_2269
SAUSA300_1206	0,67	1,49	0,567075889	No	0	1	1	1	0	1	1	1	0	conserved hypothetical protein	SAUSA300_1206
SAUSA300_0142	0,67	1,49	0,00753503	Yes	88	99	83	90	74	78	56	65	58	phosphonate ABC transporter, permease p	phnE
SAUSA300_0677	0,67	1,49	4,38E-10	Yes	858	920	830	589	543	552	547	617	578	putative deoxyribodipyrimidine photolyas	SAUSA300_0677
SAUSA300_1319	0,67	1,49	2,44E-05	Yes	2031	2070	1640	1153	1089	1066	1309	1300	1219	dihydrofolate reductase	folA
SAUSA300_1900	0,67	1,50	3,39E-10	Yes	11261	11249	10510	7070	7226	6048	7349	7640	7025	manganese-dependent inorganic pyropho	ppaC
SAUSA300_1285	0,67	1,50	1,95E-08	Yes	5136	5338	5013	2883	3074	2816	3492	3482	3343	ABC transporter, ATP-binding protein	SAUSA300_1285
SAUSA300_1592	0,67	1,50	1,15E-06	Yes	3555	3009	2813	2065	2200	1973	2147	2097	1994	single-stranded-DNA-specific exonuclease	recJ
SAUSA300_0467	0,67	1,50	1,16E-05	Yes	8684	11375	8932	7412	7368	7351	6493	6393	6396	methionyl-tRNA synthetase	metS
SAUSA300_1434	0,67	1,50	0,022684124	No	541	675	496	288	210	204	414	326	389	phiSLT ORF104a-like protein, repressor	SAUSA300_1434
SAUSA300_0668	0,67	1,50	0,002259811	Yes	4538	4248	3375	2046	1636	1862	2931	2767	2358	conserved hypothetical protein	SAUSA300_0668
SAUSA300_0345	0,67	1,50	0,11546709	No	53	129	96	102	81	154	60	52	69	Tat-translocated enzyme	SAUSA300_0345
SAUSA300_1499	0,66	1,50	0,002211716	Yes	308	268	382	74	79	52	197	210	227	shikimate kinase	aroK
SAUSA300_2571	0,66	1,50	0,174814133	No	28	33	23	27	14	16	23	12	19	arginine repressor	argR
SAUSA300_1137	0,66	1,50	2,06E-05	Yes	405	496	483	339	394	430	332	278	309	ribonuclease HII	rnhB
SAUSA300_1806	0,66	1,51	4,45E-05	Yes	935	1162	935	865	791	988	667	713	625	putative iron-sulfur cluster-binding protei	SAUSA300_1806
SAUSA300_0914	0,66	1,51	5,26E-05	Yes	448	553	455	282	300	312	311	364	288	sodium:alanine symporter family protein	SAUSA300_0914
SAUSA300_1969	0,66	1,51	0,018677199	No	1833	2515	1330	1161	915	1266	1342	1213	1166	phi77 ORF011-like protein, phage transcrip	SAUSA300_1969
SAUSA300_2045	0,66	1,51	0,0001716	Yes	963	921	823	567	543	621	633	566	583	HD domain protein	SAUSA300_2045
SAUSA300_1584	0,66	1,51	1,58E-08	Yes	3498	3158	2594	2340	2319	2478	2026	2145	1928	ATPase, AAA family	SAUSA300_1584
SAUSA300_1788	0,66	1,51	0,000915068	Yes	4695	3900	3507	1955	1859	2345	2781	2785	2394	conserved hypothetical protein	SAUSA300_1788
SAUSA300_0768	0,66	1,51	0,020065931	No	1381	835	712	593	527	469	779	594	541	conserved hypothetical protein	SAUSA300_0768
SAUSA300_2461	0,66	1,51	0,003378504	Yes	815	579	533	639	453	531	448	453	362	glyoxalase family protein	SAUSA300_2461
SAUSA300_0405	0,66	1,51	0,00013421	Yes	1702	2457	2136	1846	1717	2091	1387	1457	1302	type I restriction-modification system, M s	hsdM
SAUSA300_2356	0,66	1,52	0,000785374	Yes	291	279	214	252	176	200	177	174	161	fmhA protein	fmhA
SAUSA300_1924	0,66	1,52	0,481850404	No	7	1	2	17	11	13	1	2	3	holin	SAUSA300_1924
SAUSA300_1356	0,66	1,52	1,77E-07	Yes	863	1095	1048	649	760	689	635	659	684	3-dehydroquinate synthase	aroB
SAUSA300_1724	0,66	1,52	0,143466766	No	34	29	33	21	16	13	22	15	23	abortive infection protein family	SAUSA300_1724
SAUSA300_2384	0,66	1,52	0,000541338	Yes	4671	4009	4070	1991	2495	1911	2730	2770	2846	putative Na+/H+ antiporter	SAUSA300_2384
SAUSA300_0271	0,66	1,52	0,008946249	Yes	808	545	523	182	153	106	400	395	425	ABC transporter, ATP-binding protein	SAUSA300_0271
SAUSA300_2611	0,66	1,52	0,007735133	Yes	117	123	92	142	115	130	72	78	65	histidinol dehydrogenase hisD	hisD
SAUSA300_2161	0,65	1,53	0,000445376	Yes	308	259	246	165	178	114	160	179	187	Hyaluronate lyase precursor	hysA
SAUSA300_0596	0,65	1,53	1,91E-09	Yes	5276	5021	4913	2442	2450	1912	3342	3238	3346	arginyl-tRNA synthetase	argS
SAUSA300_1609	0,65	1,53	0,299012508	No	10	15	7	3	9	13	4	7	8	type III leader peptidase family protein	SAUSA300_1609
SAUSA300_2362	0,65	1,53	0,00886619	Yes	13278	10202	13210	4260	2612	3431	6408	7075	10268	2,3-bisphosphoglycerate-dependent phos	gpmA
SAUSA300_1729	0,65	1,53	0,001868308	Yes	6054	4840	5415	1747	1557	1184	3433	3312	3824	conserved hypothetical protein	SAUSA300_1729
SAUSA300_1763	0,65	1,53	0,002453723	Yes	172	212	160	109	88	98	120	109	122	lantibiotic epidermin leader peptide proce	epiP
SAUSA300_1891	0,65	1,54	0,002489916	Yes	400	405	404	498	602	683	287	267	225	conserved hypothetical protein	SAUSA300_1891
SAUSA300_1802	0,65	1,54	0,346488368	No	67	26	76	11	52	19	32	26	44	conserved hypothetical protein	SAUSA300_1802
SAUSA300_2297	0,65	1,54	2,80E-07	Yes	1574	1294	1184	727	683	631	923	809	880	conserved hypothetical protein	SAUSA300_2297
SAUSA300_0721	0,65	1,54	0,001543807	Yes	725	884	980	821	989	827	584	581	503	transferrin receptor	SAUSA300_0721
SAUSA300_0786	0,65	1,55	0,116063614	No	350	270	240	153	77	257	196	205	142	OsmC/Ohr family protein	SAUSA300_0786
SAUSA300_2035	0,65	1,55	0,000575385	Yes	379	498	415	334	246	269	295	240	296	sensor histidine kinase, KdpD	kdpD
SAUSA300_0391	0,65	1,55	0,109615301	No	45	53	30	16	7	6	29	25	26	conserved hypothetical protein	SAUSA300_0391
SAUSA300_1229	0,65	1,55	2,76E-11	Yes	3026	3103	3071	2294	2096	1878	1996	2000	1933	hydrolase, haloacid dehalogenase-like fam	SAUSA300_1229
SAUSA300_0651	0,64	1,55	0,000213549	Yes	13419	10692	8614	9257	10735	10808	7503	6837	6640	CHAP domain family	SAUSA300_0651
SAUSA300_0219	0,64	1,55	1,97E-05	Yes	398	423	348	319	293	308	233	280	236	putative iron compound A C transporter, i	SAUSA300_0219
SAUSA300_2025	0,64	1,56	5,18E-14	Yes	2218	2342	2241	1071	994	877	1469	1406	1486	sigma-B regulation protein	rsbU
SAUSA300_1033	0,64	1,56	0,002469778	Yes	210	237	168	309	273	311	133	129	129	iron/heme permease	SAUSA300_1033
SAUSA300_0147	0,64	1,56	0,000522108	Yes	272	387	322	374	358	481	203	221	204	5' nucleotidase family protein	SAUSA300_0147
SAUSA300_0254	0,64	1,56	1,97E-06	Yes	1516	1758	1537	1252	1028	1146	961	1073	1038	sensor histidine kinase	SAUSA300_0254
SAUSA300_2245	0,64	1,56	0,30463919	No	1245	471	1688	170	237	169	589	494	967	staphylococcal accessory regulator R	SAUSA300_2245
SAUSA300_2303	0,64	1,56	0,001960182	Yes	1021	627	810	289	374	250	497	478	584	transcriptional regulator TcaR	tcaR
SAUSA300_1331	0,64	1,57	0,288413128	No	26	12	71	44	36	30	14	32	20	alanine dehydrogenase	ald

SAUSA300_1976	0,64	1,57	0,00817608	Yes	873	1242	883	673	478	775	683	580	632	probable succinyl-diaminopimelate desuc	SAUSA300_1976
SAUSA300_1749	0,64	1,57	0,436695498	No	10	10	12	9	7	10	10	2	5	conserved hypothetical protein	SAUSA300_1749
SAUSA300_1424	0,64	1,57	0,342197732	No	7	10	6	7	5	7	5	4	4	conserved hypothetical phage protein	SAUSA300_1424
SAUSA300_1863	0,63	1,58	6,08E-05	Yes	3223	2742	2748	1101	960	988	2016	1923	1564	conserved hypothetical protein	SAUSA300_1863
SAUSA300_0586	0,63	1,58	0,021665453	No	100	62	77	30	29	25	54	45	49	conserved hypothetical protein	SAUSA300_0586
SAUSA300_0341	0,63	1,58	8,67E-11	Yes	966	1077	1054	570	561	615	612	670	677	putative membrane protein	SAUSA300_0341
SAUSA300_0026	0,63	1,58	0,004346711	Yes	439	623	366	428	363	531	294	305	296	conserved hypothetical protein OrfX	SAUSA300_0026
SAUSA300_1031	0,63	1,58	0,00416973	Yes	143	170	160	102	140	121	88	87	121	conserved hypothetical protein	SAUSA300_1031
SAUSA300_0544	0,63	1,59	1,34E-07	Yes	1877	2078	1513	1532	1339	1505	1175	1105	1158	hydrolase, haloacid dehalogenase-like fam	SAUSA300_0544
SAUSA300_2339	0,63	1,59	1,66E-07	Yes	806	909	672	524	550	501	493	517	489	conserved hypothetical protein	SAUSA300_2339
SAUSA300_0543	0,63	1,59	3,15E-07	Yes	296	290	259	248	248	291	190	171	170	putative deaminase	SAUSA300_0543
SAUSA300_1055	0,63	1,59	0,006232037	Yes	2864	1592	1566	1710	1963	1881	1229	1309	1213	fibrinogen-binding protein	efb
SAUSA300_0173	0,63	1,59	0,002268303	Yes	4669	5389	3723	2470	2044	2143	2731	2858	3017	conserved hypothetical protein	SAUSA300_0173
SAUSA300_0506	0,63	1,59	3,98E-06	Yes	4323	3462	4192	1416	1594	1272	2375	2689	2448	pyrimidine nucleoside transport protein	nupC
SAUSA300_2608	0,63	1,59	0,115127421	No	26	40	36	46	61	87	17	20	25	imidazole glycerol phosphate synthase sub	hisH
SAUSA300_2255	0,63	1,59	2,10E-11	Yes	1693	1870	1558	947	994	974	1130	1075	1009	monooxygenase family protein	SAUSA300_2255
SAUSA300_0897	0,63	1,59	3,48E-08	Yes	2833	3673	3343	1933	1893	1942	2103	2040	2036	tryptophanyl-tRNA synthetase	trpS
SAUSA300_2385	0,63	1,59	2,54E-06	Yes	3836	4124	3651	2626	2479	2545	2324	2379	2568	putative membrane protein	SAUSA300_2385
SAUSA300_2413	0,63	1,59	0,012168395	No	57	64	60	46	59	44	36	36	40	conserved hypothetical protein	SAUSA300_2413
SAUSA300_0315	0,63	1,59	0,168263174	No	24	25	16	17	14	15	11	16	12	N-acetylneuraminate lyase subunit	nanA
SAUSA300_1117	0,63	1,60	0,004917578	Yes	28741	30105	36894	9162	15442	11293	26864	17858	14709	50S ribosomal protein L28	rpmB
SAUSA300_1895	0,63	1,60	3,04E-05	Yes	1116	909	1030	566	660	568	613	642	650	nitric oxide synthase oxygenase	SAUSA300_1895
SAUSA300_1857	0,63	1,60	4,21E-05	Yes	150	151	149	62	61	62	101	94	86	conserved hypothetical protein	SAUSA300_1857
SAUSA300_2632	0,63	1,60	0,030298914	No	236	157	263	50	90	75	142	109	153	putative membrane protein	SAUSA300_2632
SAUSA300_1065	0,63	1,60	0,254309512	No	64	51	114	29	25	15	28	59	48	exfoliative toxin A	SAUSA300_1065
SAUSA300_1762	0,62	1,60	0,006841692	Yes	172	127	175	29	38	30	104	108	81	lantibiotic epidermin immunity protein F	epiF
SAUSA300_1181	0,62	1,60	4,40E-14	Yes	2160	2141	2028	1372	1377	1508	1320	1344	1272	conserved hypothetical protein	SAUSA300_1181
SAUSA300_2023	0,62	1,61	8,14E-05	Yes	3004	2895	2846	1285	1075	1301	1947	1834	1636	anti-sigma-B factor, serine-protein kinase	rsbW
SAUSA300_1182	0,62	1,61	8,53E-05	Yes	3830	4791	3152	4902	4007	4945	2595	2182	2475	pyruvate ferredoxin oxidoreductase, alpha	SAUSA300_1182
SAUSA300_1028	0,62	1,62	0,00021614	Yes	169	193	128	215	225	235	102	91	106	iron transport associated domain protein	SAUSA300_1028
SAUSA300_1807	0,62	1,62	2,55E-07	Yes	391	344	305	236	239	254	217	211	208	amino acid ABC transporter, ATP-binding p	SAUSA300_1807
SAUSA300_1054	0,62	1,62	0,315656234	No	7	18	13	13	18	16	9	6	6	conserved hypothetical protein	SAUSA300_1054
SAUSA300_2629	0,62	1,62	0,005619146	Yes	312	318	218	96	108	112	180	195	141	conserved hypothetical protein	SAUSA300_2629
SAUSA300_2399	0,62	1,62	0,001414679	Yes	377	434	411	255	230	257	261	260	227	ABC transporter, ATP-binding protein	SAUSA300_2399
SAUSA300_2306	0,62	1,62	0,010364463	No	67	81	84	56	59	59	36	55	50	ABC transporter, ATP-binding protein	SAUSA300_2306
SAUSA300_0960	0,62	1,62	3,56E-07	Yes	7477	7319	7137	2952	2364	1965	4186	4712	4553	quinol oxidase, subunit IV	qoxD
SAUSA300_1318	0,61	1,63	8,58E-10	Yes	1889	1937	1686	881	875	723	1145	1112	1119	DegV family protein	SAUSA300_1318
SAUSA300_0783	0,61	1,63	4,68E-08	Yes	961	886	743	662	584	710	521	508	552	phosphoglycerate mutase family protein	SAUSA300_0783
SAUSA300_1856	0,61	1,63	0,00307381	Yes	5226	5091	3562	2383	2017	2864	3023	2908	2501	conserved hypothetical protein	SAUSA300_1856
SAUSA300_1756	0,61	1,63	0,320985772	No	16	5	15	1	7	3	7	8	4	serine protease SplC	splC
SAUSA300_2268	0,61	1,63	3,03E-05	Yes	322	350	301	200	221	225	165	233	194	sodium/bile acid symporter family protein	SAUSA300_2268
SAUSA300_2610	0,61	1,64	0,005413848	Yes	79	99	92	74	99	98	46	65	52	histidinol-phosphate aminotransferase his	hisC
SAUSA300_0406	0,61	1,64	4,69E-10	Yes	971	1112	985	642	586	626	670	592	602	putative restriction/modification system s	SAUSA300_0406
SAUSA300_0222	0,61	1,64	8,58E-10	Yes	1616	1576	1526	726	724	562	997	940	922	putative membrane protein	SAUSA300_0222
SAUSA300_0346	0,61	1,65	0,000575571	Yes	148	196	134	165	137	182	115	84	90	putative membrane protein	SAUSA300_0346
SAUSA300_1357	0,61	1,65	1,50E-07	Yes	1078	1332	1143	892	913	1019	726	714	712	chorismate synthase	aroC
SAUSA300_1958	0,61	1,65	0,130606006	No	47	22	26	139	133	161	14	21	19	Single-strand binding protein	SAUSA300_1958
SAUSA300_1677	0,61	1,65	9,04E-08	Yes	27969	26602	23015	12736	13539	11304	15178	16314	15392	cell wall surface anchor family protein	SAUSA300_1677
SAUSA300_2403	0,61	1,65	0,019556561	No	1211	827	954	341	277	203	629	479	674	putative lipoprotein	SAUSA300_2403
SAUSA300_1320	0,61	1,65	2,19E-07	Yes	2000	2049	1708	1104	1195	1119	1235	1128	1112	thymidylate synthase	thyA
SAUSA300_1209	0,61	1,65	0,030589417	No	126	190	91	100	88	109	79	77	85	conserved hypothetical protein	SAUSA300_1209
SAUSA300_2410	0,60	1,65	0,001725451	Yes	103	122	152	125	144	161	71	71	85	oligopeptide ABC transporter, permease p	SAUSA300_2410
SAUSA300_1652	0,60	1,66	0,001286828	Yes	4454	4183	3925	2887	2078	2688	2910	2375	2222	conserved hypothetical protein	SAUSA300_1652
SAUSA300_2409	0,60	1,66	0,023623607	No	47	93	96	109	126	117	42	44	54	oligopeptide ABC transporter, permease p	SAUSA300_2409
SAUSA300_0892	0,60	1,67	0,116300402	No	16	22	27	14	23	9	14	12	11	oligopeptide ABC transporter, oligopeptid	oppA
SAUSA300_1808	0,60	1,67	1,69E-05	Yes	203	264	223	140	169	160	153	135	124	amino acid ABC transporter, permease/sul	SAUSA300_1808
SAUSA300_2489	0,60	1,67	7,32E-06	Yes	377	413	292	279	270	241	223	221	199	antibiotic transport-associated protein-like	SAUSA300_2489
SAUSA300_1968	0,60	1,67	0,169851251	No	19	12	11	29	43	25	6	9	8	putative phage transcriptional regulator	SAUSA300_1968
SAUSA300_2225	0,60	1,67	5,33E-05	Yes	1128	997	840	566	566	719	636	565	556	molybdenum cofactor biosynthesis protei	moaC

SAUSA300_2307	0,60	1,67	0,029723802	No	47	60	77	29	27	16	26	43	39	ABC transporter, permease protein	SAUSA300_2307
SAUSA300_2592	0,60	1,68	0,003551485	Yes	1104	743	736	266	214	263	566	563	390	conserved hypothetical protein	SAUSA300_2592
SAUSA300_2022	0,60	1,68	9,49E-05	Yes	11597	11010	9319	4825	4190	5671	6616	6478	5789	RNA polymerase sigma-37 factor	rpoF
SAUSA300_0542	0,59	1,68	0,105047004	No	91	66	113	29	47	30	54	39	61	deoxynucleoside kinase family protein	SAUSA300_0542
SAUSA300_1758	0,59	1,68	0,090056036	No	22	25	16	19	9	10	11	12	13	serine protease SplA	splA
SAUSA300_1809	0,59	1,69	2,68E-11	Yes	1323	1339	1141	755	726	714	737	790	715	putative membrane protein	SAUSA300_1809
SAUSA300_0794	0,59	1,69	0,000239035	Yes	241	201	221	84	101	77	155	115	119	Toprim domain protein	SAUSA300_0794
SAUSA300_1324	0,59	1,69	3,25E-05	Yes	369	394	408	170	133	127	220	210	257	putative membrane protein	SAUSA300_1324
SAUSA300_2517	0,59	1,70	6,49E-05	Yes	1025	1055	762	533	496	671	614	517	532	amidohydrolase family protein	SAUSA300_2517
SAUSA300_0247	0,59	1,70	1,52E-07	Yes	2447	2866	2216	1538	1535	1479	1517	1482	1419	putative teichoic acid biosynthesis protein	SAUSA300_0247
SAUSA300_1931	0,59	1,70	0,260759446	No	16	1	7	24	29	22	4	3	5	phi77 ORF100-like protein	SAUSA300_1931
SAUSA300_1429	0,59	1,70	0,428406257	No	7	0	1	9	0	13	2	1	0	phiSLT ORF53-like protein	SAUSA300_1429
SAUSA300_1030	0,59	1,70	0,002062016	Yes	109	186	152	162	178	158	75	85	99	iron transport associated domain protein	SAUSA300_1030
SAUSA300_1979	0,59	1,71	8,37E-05	Yes	389	316	341	206	273	152	205	182	219	cation transport family protein	SAUSA300_1979
SAUSA300_1671	0,58	1,71	2,19E-10	Yes	890	1122	1244	643	672	628	610	609	684	conserved hypothetical protein	SAUSA300_1671
SAUSA300_1366	0,58	1,71	4,74E-05	Yes	112	105	120	70	56	56	69	66	61	conserved hypothetical protein	SAUSA300_1366
SAUSA300_0070	0,58	1,72	0,00018926	Yes	1507	1244	1145	422	338	424	827	741	670	putative lysophospholipase	SAUSA300_0070
SAUSA300_1935	0,58	1,73	0,027702426	No	69	31	44	206	223	257	22	32	27	phi77 ORF029-like protein	SAUSA300_1935
SAUSA300_2416	0,58	1,73	0,010711213	No	310	350	377	200	144	353	205	196	189	glucose 1-dehydrogenase-like protein	SAUSA300_2416
SAUSA300_2340	0,58	1,73	7,58E-06	Yes	176	201	189	86	65	59	104	104	117	respiratory nitrate reductase, gamma subu	narI
SAUSA300_1966	0,58	1,73	0,094656001	No	363	103	119	871	724	884	80	141	99	phi77 ORF014-like protein, phage anti-rep	SAUSA300_1966
SAUSA300_1800	0,58	1,74	8,46E-10	Yes	3584	2569	2655	1615	1621	1488	1824	1655	1573	ribosomal large subunit pseudouridine syr	SAUSA300_1800
SAUSA300_1926	0,58	1,74	0,292534232	No	9	5	6	9	16	6	3	4	3	phi77 ORF044-like protein	SAUSA300_1926
SAUSA300_0203	0,57	1,75	1,78E-06	Yes	348	459	319	190	160	213	218	178	243	putative lipoprotein	SAUSA300_0203
SAUSA300_2538	0,57	1,75	0,002209854	Yes	2151	1748	2167	874	931	938	1057	1416	953	amino acid permease family protein	SAUSA300_2538
SAUSA300_0319	0,57	1,75	0,015857267	No	41	44	28	23	23	24	25	16	21	putative membrane protein	SAUSA300_0319
SAUSA300_0961	0,57	1,75	7,38E-19	Yes	17206	17521	17151	9369	8274	8398	9276	10510	9825	quinol oxidase, subunit III	qoxC
SAUSA300_2145	0,57	1,75	0,001178528	Yes	5133	4965	4819	1534	1355	1990	2989	2960	2440	glycine betaine transporter	SAUSA300_2145
SAUSA300_2613	0,57	1,76	0,030002273	No	79	73	58	100	70	74	24	42	49	conserved hypothetical protein	SAUSA300_2613
SAUSA300_2108	0,57	1,76	3,10E-09	Yes	1316	1161	1167	411	367	441	718	656	689	Mannitol-1-phosphate 5-dehydrogenase	mtlD
SAUSA300_0847	0,57	1,76	1,40E-08	Yes	1149	871	819	591	568	535	543	563	497	conserved hypothetical protein	SAUSA300_0847
SAUSA300_0962	0,57	1,76	2,04E-14	Yes	53346	54280	52496	26582	22686	23521	28472	32452	29859	quinol oxidase, subunit I	qoxB
SAUSA300_1971	0,57	1,76	0,008284804	Yes	176	203	120	105	83	102	128	78	72	phi77 ORF017-like protein	SAUSA300_1971
SAUSA300_0864	0,57	1,76	2,05E-06	Yes	465	478	382	295	311	288	243	275	228	argininosuccinate synthase	argG
SAUSA300_1988	0,57	1,76	0,341957338	No	11750	6178	18848	93	135	62	3024	1683	11749	delta-hemolysin precursor	SAUSA300_1988
SAUSA300_1947	0,57	1,76	0,184649626	No	14	5	8	33	25	33	6	3	5	phi77 ORF031-like protein	SAUSA300_1947
SAUSA300_1184	0,57	1,76	0,001884366	Yes	133	122	113	34	63	30	74	53	78	conserved hypothetical protein	SAUSA300_1184
SAUSA300_1058	0,57	1,77	0,000259871	Yes	987	1148	856	524	627	482	401	580	693	alpha-hemolysin precursor	SAUSA300_1058
SAUSA300_0949	0,57	1,77	0,010071554	No	110	63	95	20	34	15	41	44	63	cysteine protease	sspC
SAUSA300_2438	0,56	1,77	0,272068829	No	5	10	7	1	0	3	4	3	4	staphylococcal accessory regulator U	sarU
SAUSA300_0934	0,56	1,78	0,002825556	Yes	45	63	56	27	36	35	32	29	30	membrane protein	SAUSA300_0934
SAUSA300_0862	0,56	1,78	6,10E-05	Yes	155	119	106	47	59	53	65	77	68	glycerophosphoryl diester phosphodiester	glpQ
SAUSA300_1267	0,56	1,79	0,052672582	No	19	42	35	53	47	62	16	21	16	tryptophan synthase, beta subunit	trpB
SAUSA300_1708	0,56	1,79	0,000185715	Yes	3989	3069	3660	1743	1528	1168	2155	2190	1587	staphylococcal accessory regulator Rot	rot
SAUSA300_2107	0,56	1,79	0,000105177	Yes	580	517	445	295	259	395	303	286	261	PTS system, mannitol specific IIA compone	mtlA
SAUSA300_2026	0,56	1,80	2,60E-12	Yes	4599	5211	4343	2596	2558	2601	2884	2626	2344	PemK family protein	SAUSA300_2026
SAUSA300_1957	0,55	1,80	0,049369395	No	96	36	33	178	160	192	27	28	31	phiPVL ORF046-like protein	SAUSA300_1957
SAUSA300_1974	0,55	1,80	0,004219049	Yes	446	359	382	175	133	136	188	254	201	Leukocidin/Hemolysin toxin family proteir	SAUSA300_1974
SAUSA300_1204	0,55	1,81	0,085863689	No	59	56	72	21	16	4	29	25	44	conserved hypothetical protein	SAUSA300_1204
SAUSA300_1759	0,55	1,81	0,02258533	No	53	42	27	13	20	22	27	19	19	conserved hypothetical protein	SAUSA300_1759
SAUSA300_1056	0,55	1,81	0,000610712	Yes	1812	891	917	752	935	699	650	643	670	conserved hypothetical protein	SAUSA300_1056
SAUSA300_1685	0,55	1,82	7,88E-06	Yes	19635	13249	13798	5124	4296	4532	9779	8570	7137	conserved hypothetical protein	SAUSA300_1685
SAUSA300_2366	0,55	1,82	0,002982919	Yes	62	67	48	82	72	59	31	34	30	gamma-hemolysin component C	hlgC
SAUSA300_1453	0,55	1,82	6,34E-20	Yes	1326	1388	1192	879	816	847	711	727	698	ribonuclease Z	SAUSA300_1453
SAUSA300_0711	0,55	1,82	4,06E-05	Yes	1808	1162	1204	382	334	383	819	834	611	conserved hypothetical protein	SAUSA300_0711
SAUSA300_2572	0,55	1,83	0,000126863	Yes	472	423	266	292	228	272	251	196	180	zinc metalloproteinase aureolysin	aur
SAUSA300_1768	0,55	1,83	0,022978961	No	26	27	34	14	14	22	12	16	18	leukotoxin LukD	lukD
SAUSA300_1964	0,55	1,83	0,079920521	No	152	31	48	274	243	217	31	49	38	conserved hypothetical phage protein	SAUSA300_1964
SAUSA300_0631	0,55	1,83	1,44E-26	Yes	4562	4917	5024	2297	2227	2271	2645	2784	2482	putative nucleoside transporter	SAUSA300_0631

SAUSA300_2326	0,54	1,84	0,004227293	Yes	570	416	610	137	167	111	244	277	329	transcription regulatory protein	SAUSA300_2326
SAUSA300_0770	0,54	1,84	0,015046698	No	36	44	35	16	0	16	24	21	16	conserved hypothetical protein	SAUSA300_0770
SAUSA300_0344	0,54	1,85	0,012660163	No	34	82	69	29	29	43	31	29	38	putative lipoprotein	SAUSA300_0344
SAUSA300_0782	0,54	1,85	1,61E-05	Yes	785	565	574	269	268	250	381	368	278	conserved hypothetical protein	SAUSA300_0782
SAUSA300_0495	0,54	1,86	0,304950198	No	7	4	23	9	11	12	4	4	6	hypothetical protein	SAUSA300_0495
SAUSA300_0568	0,54	1,86	5,23E-12	Yes	939	1125	862	656	701	753	542	533	494	integral membrane protein	SAUSA300_0568
SAUSA300_0712	0,54	1,86	6,67E-07	Yes	14137	17684	15467	9031	9782	9476	8819	9584	6806	amino acid/peptide transporter (Peptide:H	SAUSA300_0712
SAUSA300_0389	0,54	1,86	1,50E-07	Yes	43813	50631	43823	24659	25604	27284	25903	26283	21454	GMP synthase	guaA
SAUSA300_1226	0,54	1,87	7,40E-08	Yes	1080	1543	1525	601	557	640	673	748	786	homoserine dehydrogenase	SAUSA300_1226
SAUSA300_0092	0,53	1,87	0,029228303	No	45	30	22	36	25	12	16	16	17	conserved hypothetical protein	SAUSA300_0092
SAUSA300_2518	0,53	1,88	2,98E-05	Yes	2949	3809	2531	1580	1287	2094	1782	1572	1537	hydrolase family protein	SAUSA300_2518
SAUSA300_1855	0,53	1,88	2,11E-13	Yes	1357	1101	1045	497	444	376	632	612	609	monofunctional glycosyltransferase	sgtB
SAUSA300_1582	0,53	1,88	0,002160691	Yes	2177	1589	1996	543	471	593	1224	1026	752	conserved hypothetical protein	SAUSA300_1582
SAUSA300_2396	0,53	1,88	6,00E-12	Yes	1269	1467	1117	600	557	593	690	701	650	para-nitrobenzyl esterase	pnbA
SAUSA300_2423	0,53	1,88	0,25333519	No	537	277	1178	165	99	81	145	533	185	conserved hypothetical protein	SAUSA300_2423
SAUSA300_2313	0,53	1,88	NA	NA	1903	1847	11133	617	539	577	633	4707	825	L-lactate permease	SAUSA300_2313
SAUSA300_2615	0,53	1,88	2,19E-05	Yes	119	130	106	53	47	34	62	58	65	conserved hypothetical protein	SAUSA300_2615
SAUSA300_1249	0,53	1,89	2,85E-08	Yes	2401	1717	2034	898	714	689	1150	1102	972	conserved hypothetical protein	SAUSA300_1249
SAUSA300_2024	0,53	1,90	1,18E-10	Yes	1591	1511	1694	451	408	361	950	819	750	anti-sigma-B factor, antagonist	rsbV
SAUSA300_0246	0,53	1,90	1,80E-06	Yes	1314	1842	1518	1133	969	1158	826	844	768	putative alcohol dehydrogenase	SAUSA300_0246
SAUSA300_1581	0,53	1,90	0,004034179	Yes	1440	843	1826	182	140	192	729	534	840	conserved hypothetical protein	SAUSA300_1581
SAUSA300_0963	0,52	1,91	2,01E-12	Yes	31741	29362	29377	13487	11858	12163	14237	16535	16463	quinol oxidase, subunit II	qoxA
SAUSA300_2544	0,52	1,91	0,013546018	No	62	57	93	13	16	3	50	26	32	conserved hypothetical protein	SAUSA300_2544
SAUSA300_2418	0,52	1,91	6,62E-05	Yes	1430	1129	1423	271	214	277	757	664	635	conserved hypothetical protein	SAUSA300_2418
SAUSA300_2528	0,52	1,92	2,37E-12	Yes	692	630	607	629	681	569	352	334	314	conserved hypothetical protein	SAUSA300_2528
SAUSA300_0146	0,52	1,93	1,33E-05	Yes	121	189	144	150	162	149	74	79	80	conserved hypothetical protein	SAUSA300_0146
SAUSA300_0245	0,52	1,93	8,72E-09	Yes	754	925	949	485	478	563	420	480	452	2-C-methyl-D-erythritol 4-phosphate cytid	SAUSA300_0245
SAUSA300_0145	0,52	1,94	4,89E-05	Yes	74	85	95	37	45	31	41	49	41	phosphonate ABC transporter, phosphona	SAUSA300_0145
SAUSA300_1804	0,52	1,94	8,03E-11	Yes	7100	5326	5859	1896	1634	1730	3246	3159	2959	conserved hypothetical protein	SAUSA300_1804
SAUSA300_2379	0,52	1,94	1,91E-10	Yes	2682	2254	2482	1219	1226	1167	1263	1324	1210	putative transporter protein	SAUSA300_2379
SAUSA300_1892	0,51	1,95	6,73E-05	Yes	529	532	686	561	609	642	301	264	317	conserved hypothetical protein	SAUSA300_1892
SAUSA300_2142	0,51	1,95	1,54E-09	Yes	44290	42359	44052	10460	8892	12108	23546	22741	20180	alkaline shock protein 23	asp23
SAUSA300_0979	0,51	1,95	1,57E-05	Yes	834	947	722	483	433	442	464	456	344	conserved hypothetical protein	SAUSA300_0979
SAUSA300_1883	0,51	1,96	2,93E-08	Yes	2646	3025	2639	1339	1267	1542	1411	1525	1279	high affinity proline permease	putP
SAUSA300_1490	0,51	1,96	1,47E-11	Yes	13865	10585	10818	5350	4679	4356	6390	6124	5349	translation elongation factor P	efp
SAUSA300_1057	0,51	1,96	4,21E-05	Yes	532	330	414	103	72	56	212	212	215	conserved hypothetical protein	SAUSA300_1057
SAUSA300_1035	0,51	1,96	4,58E-06	Yes	164	141	130	87	115	83	78	63	76	conserved hypothetical protein	SAUSA300_1035
SAUSA300_2436	0,51	1,97	4,60E-06	Yes	1216	2140	2344	1240	1093	1313	999	1000	868	putative cell wall surface anchor family pro	SAUSA300_2436
SAUSA300_1874	0,51	1,97	7,71E-05	Yes	4480	3200	2007	6981	6586	5127	1808	1473	1556	ferritins family protein	SAUSA300_1874
SAUSA300_1959	0,51	1,97	0,06480895	No	34	14	15	79	70	56	7	15	7	phiPVL ORF044-like protein	SAUSA300_1959
SAUSA300_2415	0,51	1,97	0,190397575	No	2	8	7	4	9	7	6	1	1	conserved hypothetical protein	SAUSA300_2415
SAUSA300_0816	0,51	1,97	0,000275696	Yes	22268	17045	19320	1979	1510	1622	10799	9697	8628	CsbD-like superfamily	SAUSA300_0816
SAUSA300_0878	0,51	1,98	0,022059687	No	26	31	35	10	7	9	12	14	19	transcriptional regulator, LysR family	SAUSA300_0878
SAUSA300_1975	0,51	1,98	0,00338657	Yes	348	231	345	126	101	72	121	157	175	Aerolysin/Leukocidin family protein	SAUSA300_1975
SAUSA300_2275	0,51	1,98	1,85E-05	Yes	1740	2172	2094	763	669	1128	1041	1012	941	oxidoreductase, short chain dehydrogenas	SAUSA300_2275
SAUSA300_2620	0,50	1,98	0,000189224	Yes	1169	835	723	404	241	263	435	387	526	conserved hypothetical protein	SAUSA300_2620
SAUSA300_2398	0,50	1,99	7,58E-06	Yes	971	757	727	302	277	358	447	433	339	putative membrane protein	SAUSA300_2398
SAUSA300_0388	0,50	1,99	1,02E-08	Yes	32737	42659	36761	19756	20294	22964	18814	19076	18092	inosine-5'-monophosphate dehydrogenas	guaB
SAUSA300_1684	0,50	2,01	1,47E-10	Yes	12601	11486	8889	4859	4100	5399	5840	5288	5170	conserved hypothetical protein	SAUSA300_1684
SAUSA300_1862	0,50	2,02	1,60E-09	Yes	5119	4145	4226	1461	1156	1541	2255	2235	2141	conserved hypothetical protein	SAUSA300_1862
SAUSA300_0720	0,49	2,02	1,97E-07	Yes	129	182	167	123	142	148	84	79	72	putative iron compound ABC transporter,	SAUSA300_0720
SAUSA300_1034	0,49	2,03	0,000171336	Yes	100	93	118	54	79	47	45	46	59	sortase B	srtB
SAUSA300_0785	0,49	2,03	0,002156435	Yes	1049	631	1099	113	110	58	426	372	531	acetyltransferase, GNAT family	SAUSA300_0785
SAUSA300_0618	0,49	2,03	1,28E-05	Yes	58744	53704	64154	10640	6667	8337	22062	24010	39633	ABC transporter, substrate-binding protein	SAUSA300_0618
SAUSA300_2617	0,49	2,03	7,26E-11	Yes	350	487	399	162	180	163	187	201	217	putative cobalt ABC transporter, ATP-bind	SAUSA300_2617
SAUSA300_1870	0,49	2,04	1,26E-14	Yes	486	496	460	243	234	170	236	253	216	conserved hypothetical protein	SAUSA300_1870
SAUSA300_1960	0,49	2,05	0,021192778	No	300	97	81	581	433	521	65	82	72	putative phage-related DNA recombinatio	SAUSA300_1960
SAUSA300_2115	0,48	2,07	0,202328661	No	3	7	4	3	2	4	1	4	0	IS1181, transposase	tnp
SAUSA300_0144	0,48	2,07	3,66E-05	Yes	115	96	65	62	61	61	44	49	38	phosphonate ABC transporter, ATP-bindin	phnC

SAUSA300_1769	0,48	2,08	0,005168375	Yes	29	47	32	20	32	18	16	18	16	leukotoxin LukE	lukE
SAUSA300_0913	0,48	2,08	8,21E-11	Yes	3917	3265	4456	801	856	608	1698	1830	2025	putative membrane protein	SAUSA300_0913
SAUSA300_1961	0,48	2,08	0,015561363	No	434	133	119	560	512	512	79	119	111	phiPVL ORF41-like protein	SAUSA300_1961
SAUSA300_0954	0,48	2,08	3,86E-08	Yes	1171	1061	924	450	311	290	473	525	500	transcriptional regulator, MarR family	SAUSA300_0954
SAUSA300_0977	0,48	2,09	1,33E-11	Yes	338	298	336	110	135	95	161	157	144	cobalt transport family protein	SAUSA300_0977
SAUSA300_0115	0,48	2,10	1,33E-05	Yes	2349	2007	1950	1044	1014	1217	1029	986	935	iron compound ABC transporter, permeas	sirC
SAUSA300_1227	0,47	2,11	4,54E-14	Yes	1533	1778	1670	886	771	797	719	828	802	threonine synthase	thrC
SAUSA300_0753	0,47	2,11	1,35E-09	Yes	1109	1157	923	460	392	492	502	556	436	conserved hypothetical protein	SAUSA300_0753
SAUSA300_0048	0,47	2,11	0,073164349	No	14	11	13	6	5	7	7	4	5	hypothetical protein	SAUSA300_0048
SAUSA300_0978	0,47	2,12	5,02E-09	Yes	2206	2699	1966	1204	1156	951	1109	1173	928	ABC transporter, ATP-binding protein	SAUSA300_0978
SAUSA300_2486	0,47	2,12	1,25E-08	Yes	5257	4086	3451	2049	1479	1998	2019	2020	1917	putative ATP-dependent Clp proteinase	SAUSA300_2486
SAUSA300_1683	0,47	2,13	1,88E-28	Yes	4297	5140	4891	2250	2087	2174	2177	2202	2341	chorismate mutase/phospho-2-dehydro-3	SAUSA300_1683
SAUSA300_2618	0,47	2,14	1,62E-05	Yes	71	97	100	30	27	31	32	43	48	conserved hypothetical protein	SAUSA300_2618
SAUSA300_1461	0,47	2,15	0,17359965	No	5	3	6	4	5	3	1	2	2	conserved hypothetical protein	SAUSA300_1461
SAUSA300_0202	0,47	2,15	1,54E-06	Yes	179	267	151	87	65	78	107	82	84	peptide ABC transporter, permease protei	SAUSA300_0202
SAUSA300_2600	0,46	2,15	0,000643451	Yes	36	57	39	46	45	30	20	19	21	intercellular adhesion protein A	icaA
SAUSA300_0774	0,46	2,16	4,90E-06	Yes	98	83	75	36	38	37	35	42	39	secretory extracellular matrix and plasma l	empbp
SAUSA300_0619	0,46	2,16	3,85E-06	Yes	30117	30046	36494	6801	4233	5655	11193	12470	20231	ABC transporter, permease protein	SAUSA300_0619
SAUSA300_1307	0,46	2,17	4,45E-12	Yes	2070	1859	1685	444	417	383	873	778	909	sensor histidine kinase protein	arlS
SAUSA300_0343	0,46	2,18	1,12E-05	Yes	443	791	529	291	289	389	269	251	274	acetyltransferase, GNAT family	SAUSA300_0343
SAUSA300_1068	0,46	2,18	0,13103438	No	1027	1680	1881	52	36	41	280	138	1201	antibacterial protein	SAUSA300_1068
SAUSA300_1228	0,46	2,18	2,42E-17	Yes	1602	1686	1533	693	719	652	703	720	776	homoserine kinase	thrB
SAUSA300_0982	0,46	2,18	1,46E-09	Yes	1362	1222	1233	338	394	414	672	616	446	conserved hypothetical protein	SAUSA300_0982
SAUSA300_0598	0,46	2,18	1,95E-17	Yes	1936	2074	1657	680	530	547	903	857	824	putative iron compound ABC transporter,	SAUSA300_0598
SAUSA300_0719	0,46	2,18	2,61E-05	Yes	67	118	118	42	52	49	46	42	49	iron compound ABC transporter, permeas	SAUSA300_0719
SAUSA300_2435	0,46	2,19	1,44E-09	Yes	1440	2230	2045	1224	1075	1522	858	920	807	cell wall surface anchor family protein	SAUSA300_2435
SAUSA300_0475	0,46	2,19	3,13E-11	Yes	8135	7129	6105	2717	2518	3388	3468	3413	2792	SpoVG protein	SAUSA300_0475
SAUSA300_1218	0,46	2,19	0,053402232	No	17	15	18	4	7	1	8	4	8	ABC transporter, permease protein	SAUSA300_1218
SAUSA300_1986	0,45	2,20	2,68E-05	Yes	665	441	467	160	196	132	247	203	248	nitroreductase family protein	SAUSA300_1986
SAUSA300_2327	0,45	2,20	1,80E-08	Yes	1659	1065	1189	405	376	393	656	571	526	conserved hypothetical protein	SAUSA300_2327
SAUSA300_1803	0,45	2,21	2,82E-06	Yes	1361	866	1297	169	189	152	554	479	534	conserved hypothetical protein	SAUSA300_1803
SAUSA300_1923	0,45	2,21	0,001298299	Yes	41	26	28	110	142	124	12	14	14	autolysin	SAUSA300_1923
SAUSA300_1437	0,45	2,23	7,83E-14	Yes	892	653	849	218	171	161	370	350	343	phiSLT ORF204-like protein	SAUSA300_1437
SAUSA300_0354	0,45	2,23	1,30E-06	Yes	909	642	984	168	212	138	336	367	412	low temperature requirement protein Ltr/	ltrA
SAUSA300_0387	0,45	2,23	3,36E-10	Yes	23362	27908	23714	10665	10796	11230	11193	11431	10578	xanthine permease	pbuX
SAUSA300_1670	0,45	2,24	3,11E-20	Yes	1545	1815	1269	748	811	662	723	673	662	D-3-phosphoglycerate dehydrogenase	serA
SAUSA300_2619	0,44	2,26	6,60E-05	Yes	65	131	109	39	47	52	42	36	53	conserved hypothetical protein	SAUSA300_2619
SAUSA300_0446	0,44	2,27	1,26E-07	Yes	405	678	623	430	446	618	256	250	234	glutamate synthase, small subunit	gltD
SAUSA300_1205	0,44	2,28	0,139097828	No	9	11	5	1	5	0	2	2	3	conserved hypothetical protein	SAUSA300_1205
SAUSA300_0112	0,44	2,28	2,45E-10	Yes	6390	8102	5764	3353	3153	3595	3213	2723	2845	L-lactate permease	lctP
SAUSA300_1308	0,43	2,30	3,59E-11	Yes	1424	1577	1285	404	356	359	625	621	594	DNA-binding response regulator	arlR
SAUSA300_0386	0,43	2,30	1,87E-10	Yes	9402	11668	9540	3716	3829	3697	4695	4468	3972	xanthine phosphoribosyltransferase	xpt
SAUSA300_2616	0,43	2,31	1,96E-08	Yes	171	192	193	40	52	40	79	72	85	cobalt transport family protein	SAUSA300_2616
SAUSA300_2264	0,43	2,31	7,62E-07	Yes	3861	2942	4056	557	485	389	1383	1335	1885	phosphosugar-binding transcriptional regu	SAUSA300_2264
SAUSA300_1990	0,43	2,32	0,027368877	No	4459	3544	5295	150	214	86	1369	1103	2684	accessory gene regulator protein D	agrD
SAUSA300_1864	0,43	2,33	6,17E-16	Yes	9755	7575	7007	1509	1352	1532	3551	3440	3352	putative membrane protein	SAUSA300_1864
SAUSA300_0784	0,43	2,33	1,07E-10	Yes	820	732	799	162	153	111	336	341	318	LysE/YggA family protein	SAUSA300_0784
SAUSA300_0620	0,43	2,34	5,58E-08	Yes	48549	46442	53199	9953	5966	8778	16440	17941	27928	ABC transporter ATP-binding protein	SAUSA300_0620
SAUSA300_1919	0,43	2,34	5,26E-05	Yes	4192	2035	2172	538	733	461	1221	996	1256	conserved hypothetical protein	SAUSA300_1919
SAUSA300_1212	0,43	2,34	0,008992323	Yes	26	29	23	6	5	9	11	7	13	conserved hypothetical protein	SAUSA300_1212
SAUSA300_0557	0,42	2,36	9,52E-13	Yes	546	619	477	391	435	404	248	231	211	HAD-superfamily hydrolase, subfamily IA, \	SAUSA300_0557
SAUSA300_0474	0,42	2,36	3,24E-20	Yes	6953	5722	5870	1983	1704	1987	2766	2678	2347	putative endoribonuclease L-PSP	SAUSA300_0474
SAUSA300_0116	0,42	2,38	1,43E-09	Yes	462	612	501	554	602	779	209	237	207	iron compound ABC transporter, permeas	sirB
SAUSA300_0889	0,42	2,38	4,96E-05	Yes	351	702	698	185	196	325	215	251	246	oligopeptide ABC transporter, ATP-binding	oppD
SAUSA300_0890	0,42	2,38	8,11E-07	Yes	630	1131	1084	281	300	322	368	413	389	oligopeptide ABC transporter, ATP-binding	oppF
SAUSA300_0143	0,42	2,39	1,48E-05	Yes	50	64	67	37	20	37	30	19	25	phosphonate ABC transporter, permease p	phnE
SAUSA300_1067	0,42	2,40	0,096185227	No	510	754	1176	7	14	13	113	65	551	antibacterial protein	SAUSA300_1067
SAUSA300_0951	0,41	2,43	1,47E-15	Yes	796	756	597	481	385	424	305	306	263	V8 protease	sspA
SAUSA300_0257	0,41	2,45	4,47E-08	Yes	460	645	707	178	234	185	218	320	190	Antiholin-like protein lrgB	SAUSA300_0257

SAUSA300_0718	0,41	2,45	4,79E-08	Yes	186	357	259	261	230	309	102	109	110	iron compound ABC transporter, permeas	SAUSA300_0718
SAUSA300_0888	0,41	2,46	1,37E-05	Yes	196	334	412	84	90	138	118	130	123	oligopeptide ABC transporter, permease p	oppC
SAUSA300_1669	0,40	2,47	1,38E-23	Yes	1016	1259	872	494	518	516	441	418	407	aminotransferase, class V	SAUSA300_1669
SAUSA300_1292	0,40	2,47	1,15E-30	Yes	536	565	576	170	192	172	216	219	239	alanine racemase	alr2
SAUSA300_1291	0,40	2,48	1,58E-27	Yes	589	600	588	235	219	217	230	238	245	hippurate hydrolase	SAUSA300_1291
SAUSA300_2331	0,40	2,48	1,49E-14	Yes	3551	2287	2375	534	600	427	1163	1035	1079	transcriptional regulator, MarR family	SarZ
SAUSA300_0360	0,40	2,49	0,000192247	Yes	121	208	86	34	41	41	50	61	48	Cys/Met metabolism PLP-dependent enzy	SAUSA300_0360
SAUSA300_0025	0,40	2,50	5,13E-16	Yes	1219	1741	1237	902	845	1023	585	569	511	5'-nucleotidase family protein	SAUSA300_0025
SAUSA300_0964	0,40	2,50	5,91E-18	Yes	5012	3744	3296	945	782	763	1706	1394	1677	chitinase-related protein	SAUSA300_0964
SAUSA300_0382	0,40	2,50	4,61E-13	Yes	3369	3607	2181	1860	1535	2035	1300	1132	1181	sodium:dicarboxylate symporter family pr	SAUSA300_0382
SAUSA300_1217	0,40	2,51	0,026477471	No	16	14	14	1	2	0	6	3	6	ABC transporter, ATP-binding protein	SAUSA300_1217
SAUSA300_2144	0,39	2,55	3,30E-16	Yes	18458	17978	17432	3176	2518	3574	7491	7015	6390	conserved hypothetical protein, asp23 op	SAUSA300_2144
SAUSA300_0201	0,39	2,57	4,09E-09	Yes	145	207	159	44	38	52	70	50	74	peptide ABC transporter, permease protei	SAUSA300_0201
SAUSA300_1225	0,38	2,62	6,22E-11	Yes	386	319	308	63	70	84	113	128	137	aspartate kinase	SAUSA300_1225
SAUSA300_1290	0,38	2,64	1,30E-20	Yes	482	508	537	185	151	217	184	210	181	tetrahydrodipicolinate acetyltransferase	dapD
SAUSA300_2143	0,38	2,64	8,11E-14	Yes	10122	10688	9358	2853	2256	3823	4092	3801	3378	conserved hypothetical protein (asp23 op	SAUSA300_2143
SAUSA300_0135	0,38	2,66	1,16E-13	Yes	1393	1441	1111	1075	1066	1245	477	514	468	Superoxide dismutase (Mn/Fe family)	SAUSA300_0135
SAUSA300_0437	0,37	2,67	3,42E-23	Yes	934	1354	1090	388	298	340	426	428	400	NLPA lipoprotein	SAUSA300_0437
SAUSA300_2013	0,37	2,67	7,51E-10	Yes	157	222	177	89	63	75	74	71	59	3-isopropylmalate dehydratase, small subu	leuD
SAUSA300_1989	0,37	2,68	0,005625983	Yes	12997	10873	16021	523	572	336	3525	2912	6919	accessory gene regulator protein B	agrB
SAUSA300_2012	0,37	2,71	2,74E-14	Yes	355	448	407	166	178	169	138	155	148	3-isopropylmalate dehydratase, large subu	leuC
SAUSA300_0887	0,37	2,71	1,64E-08	Yes	219	283	483	84	79	87	103	125	127	oligopeptide ABC transporter, permease p	oppB
SAUSA300_1286	0,37	2,71	2,84E-11	Yes	417	631	494	158	144	158	160	197	201	aspartate kinase	SAUSA300_1286
SAUSA300_1231	0,36	2,76	9,12E-27	Yes	10756	11538	8918	3297	3135	3370	3796	3752	3686	gamma-aminobutyrate permease	SAUSA300_1231
SAUSA300_0891	0,36	2,76	8,58E-10	Yes	2573	3434	3116	699	775	1032	1060	1126	1041	oligopeptide ABC transporter, substrate-b	oppA
SAUSA300_2014	0,36	2,77	7,74E-23	Yes	298	286	316	83	77	72	101	104	117	threonine dehydratase	ilvA
SAUSA300_1288	0,36	2,79	7,45E-16	Yes	575	788	622	155	187	161	216	234	250	dihydrodipicolinate synthase	dapA
SAUSA300_0206	0,36	2,81	1,52E-09	Yes	973	724	687	239	176	174	269	255	302	flavodoxin family protein	SAUSA300_0206
SAUSA300_0445	0,35	2,82	2,31E-06	Yes	579	1299	1040	743	665	847	378	313	301	glutamate synthase, large subunit	gltB
SAUSA300_0950	0,35	2,84	8,52E-24	Yes	792	617	613	282	230	214	211	244	247	cysteine protease precursor	sspB
SAUSA300_0898	0,35	2,85	3,01E-14	Yes	28457	15653	19490	3119	2750	2505	7542	7243	7159	Regulatory protein spx	spxA
SAUSA300_0256	0,35	2,87	5,10E-06	Yes	196	153	287	40	63	40	64	95	54	holin-like protein lrgA	SAUSA300_0256
SAUSA300_1991	0,35	2,90	1,54E-05	Yes	25251	22148	28164	1223	1404	849	6547	5852	12274	accessory gene regulator protein C	agrC
SAUSA300_0011	0,34	2,90	9,47E-11	Yes	357	501	352	193	153	158	140	144	124	conserved hypothetical protein	SAUSA300_0011
SAUSA300_1210	0,34	2,93	1,37E-09	Yes	200	162	216	13	23	24	68	48	75	conserved hypothetical protein	SAUSA300_1210
SAUSA300_2247	0,34	2,94	7,70E-11	Yes	215	190	177	44	72	30	68	61	64	staphylococcal accessory regulator sar?	SAUSA300_2247
SAUSA300_0357	0,34	2,94	3,26E-35	Yes	1200	1373	1006	342	356	313	431	402	377	5-methyltetrahydropteroyltriglutamate--h	metE
SAUSA300_1014	0,34	2,95	2,63E-30	Yes	10542	14029	9697	4308	3931	4667	3804	3995	3713	pyruvate carboxylase	pyc
SAUSA300_0117	0,34	2,96	1,05E-08	Yes	1683	2487	2043	1630	1456	1724	625	707	701	iron compound ABC transporter, iron com	sirA
SAUSA300_2011	0,34	2,97	1,89E-13	Yes	253	357	294	125	128	117	104	107	89	3-isopropylmalate dehydrogenase	leuB
SAUSA300_1992	0,33	3,00	1,78E-06	Yes	21033	19499	20788	1219	1237	732	5196	4756	9523	accessory gene regulator protein A	agrA
SAUSA300_0010	0,33	3,01	4,31E-14	Yes	615	840	617	268	257	313	229	240	208	putative membrane protein	SAUSA300_0010
SAUSA300_0436	0,33	3,01	1,55E-10	Yes	212	438	302	90	88	127	94	97	117	ABC transporter, permease protein	SAUSA300_0436
SAUSA300_0099	0,33	3,03	1,20E-10	Yes	169	209	228	80	74	117	70	59	65	1-phosphatidylinositol phosphodiesterase	plc
SAUSA300_1289	0,33	3,05	6,14E-16	Yes	665	842	723	228	237	253	209	255	254	dihydrodipicolinate reductase	dapB
SAUSA300_0435	0,32	3,12	1,78E-11	Yes	406	794	546	155	149	163	146	210	189	ABC transporter, ATP-binding protein	SAUSA300_0435
SAUSA300_2010	0,32	3,14	7,55E-20	Yes	422	572	464	156	165	155	168	160	132	2-isopropylmalate synthase	leuA
SAUSA300_0188	0,32	3,17	4,23E-39	Yes	5828	5386	5075	1159	1125	980	1651	1733	1707	branched-chain amino acid transport syste	brnQ
SAUSA300_1287	0,31	3,19	9,72E-16	Yes	765	997	868	262	243	257	219	295	292	aspartate semialdehyde dehydrogenase	asd
SAUSA300_0114	0,31	3,25	3,13E-09	Yes	1691	1174	2010	298	358	296	388	490	561	staphylococcal accessory regulator	sarS
SAUSA300_2417	0,31	3,26	2,20E-33	Yes	2580	2768	2766	750	728	784	794	873	795	putative transporter	SAUSA300_2417
SAUSA300_1029	0,30	3,35	3,82E-14	Yes	3872	4331	3120	2002	2008	2257	1079	1105	1103	iron transport associated domain protein	SAUSA300_1029
SAUSA300_0796	0,30	3,36	2,41E-15	Yes	1934	3267	2212	666	613	747	724	773	654	ABC transporter, ATP-binding protein	SAUSA300_0796
SAUSA300_0928	0,29	3,44	2,47E-14	Yes	146	136	147	50	43	37	42	35	44	competence transcription factor	comK
SAUSA300_2007	0,29	3,44	4,66E-20	Yes	477	645	484	205	165	197	147	159	151	acetolactate synthase, large subunit	ilvB
SAUSA300_2009	0,29	3,48	6,60E-22	Yes	291	376	319	83	72	102	102	90	88	ketol-acid reductoisomerase	ilvC
SAUSA300_2006	0,28	3,52	2,03E-09	Yes	389	642	450	146	133	176	148	135	120	dihydroxy-acid dehydratase	ilvD
SAUSA300_0815	0,28	3,55	4,65E-11	Yes	692	360	287	235	176	152	130	102	128	Ear protein	ear
SAUSA300_1922	0,28	3,56	7,47E-22	Yes	453	335	322	163	178	189	105	105	95	staphylokinase precursor	sak

SAUSA300_2008	0,28	3,62	1,54E-06	Yes	36	53	50	13	20	10	13	10	13	acetolactate synthase, small subunit	ilvN
SAUSA300_0358	0,28	3,63	5,95E-15	Yes	549	782	410	130	110	149	181	150	135	putative 5-methyltetrahydrofolate--homo	SAUSA300_0358
SAUSA300_0846	0,27	3,65	1,45E-14	Yes	1262	739	820	412	336	359	257	233	257	Na+/H+ antiporter family protein	SAUSA300_0846
SAUSA300_0797	0,27	3,67	8,97E-15	Yes	1059	1767	1134	296	273	321	373	383	292	ABC transporter permease protein	SAUSA300_0797
SAUSA300_0359	0,27	3,71	1,17E-07	Yes	134	298	100	27	29	30	53	44	38	trans-sulfuration enzyme family protein	SAUSA300_0359
SAUSA300_0372	0,26	3,85	6,90E-10	Yes	8665	10435	6068	1475	870	1612	2513	1861	1809	putative lipoprotein	SAUSA300_0372
SAUSA300_2364	0,26	3,88	3,64E-16	Yes	17391	12466	8400	6278	6926	6866	3212	3279	3063	IgG-binding protein SBI	sbi
SAUSA300_1211	0,25	3,99	1,13E-15	Yes	570	480	419	97	72	56	123	116	115	conserved hypothetical protein	SAUSA300_1211
SAUSA300_0798	0,24	4,20	7,62E-41	Yes	1845	2382	1784	278	270	322	511	456	443	ABC transporter, substrate-binding proteir	SAUSA300_0798
SAUSA300_2248	0,24	4,24	4,86E-19	Yes	468	424	511	69	97	59	108	95	117	transcriptional regulator, AraC family IKKE	SAUSA300_2248
SAUSA300_0672	0,22	4,48	6,68E-53	Yes	22874	14852	17997	2591	2328	2110	4110	4143	4026	transcriptional regulator, MarR family	mgrA
SAUSA300_0776	0,22	4,57	2,76E-20	Yes	4352	2888	2286	677	449	529	730	640	639	thermonuclease precursor	nuc
SAUSA300_0320	0,20	5,06	1,68E-76	Yes	12280	12686	9945	2904	2833	3417	2306	2005	2506	triacylglycerol lipase precursor	tig
SAUSA300_0136	0,18	5,71	3,42E-58	Yes	3471	2965	2856	384	345	349	549	566	481	cell wall surface anchor family protein	SAUSA300_0136
SAUSA300_1920	0,13	7,50	1,28E-09	Yes	160	71	83	14	27	9	9	9	16	chemotaxis-inhibiting protein CHIPS	chs
SAUSA300_1890	0,13	7,74	2,28E-47	Yes	1076	1061	837	139	149	143	128	128	114	staphopain A	SAUSA300_1890
SAUSA300_0307	0,13	7,76	8,82E-46	Yes	4907	4873	3772	567	401	503	606	538	537	5'-nucleotidase, lipoprotein e(P4) family	SAUSA300_0307
SAUSA300_0113	0,10	9,57	9,32E-09	Yes	304605	486402	340505	75164	82216	101920	14383	49815	21401	immunoglobulin G binding protein A precu	SAUSA300_0113
SAUSA300_2454	0,02	55,11	1,50E-97	Yes	594	664	769	24	18	35	11	11	11	membrane spanning protein B that binds	smpB
SAUSA300_2453	0,01	77,37	1,34E-91	Yes	1600	3107	1537	116	92	145	22	26	19	ABC transporter, ATP-binding protein	SAUSA300_2453

Supplementary Table 2. Transcription in JE2 clpP compared to JE2 expressing $ClpX_{I265E}$

ID	FoldChange (clpP/JE2 X265)	padj	Significant	JE2 WT E1	JE2 WT E2	JE2 WT E3	clpP E1	clpP E2	clpP E3	ΔclpXP E1	ΔclpXP E2	ΔclpXP E3	Product	Gene
SAUSA300_0877	**27,9**	2,75E-172	Yes	1121	1228	1055	35786	29274	39656	1215	1258	1128	Chaperone clpB	clpB
SAUSA300_0118	**12,8**	3,08E-37	Yes	14	38	29	242	309	374	20	21	25	StaphyloferrinB biosynthesis	sbnA
SAUSA300_0508	**12,5**	5,94E-168	Yes	804	1043	721	13951	13224	14696	1114	1119	1054	McsA regulation of CtsR actovotu	mcsA
SAUSA300_0509	**10,9**	4,74E-201	Yes	1454	1864	1408	22215	21284	22166	2024	1866	2028	MscB ATP guanido phosphotransferase	mcsB
SAUSA300_0119	**10,8**	6,30E-36	Yes	48	60	53	331	419	552	42	24	45	StaphyloferrinB biosynthesis	sbnB
SAUSA300_0510	**10,4**	0	Yes	6342	6759	5663	84486	81211	89621	8655	7840	7875	endopeptidase	clpC
SAUSA300_0507	**9,2**	0	Yes	613	649	597	7178	7120	6974	799	750	746	transcriptional regulator CtsR	ctsR
SAUSA300_1934	**9,1**	2,00E-31	Yes	41	29	28	242	228	288	22	31	26	ϕSA3 prophage, major phage tail protein	SAUSA300_1934
SAUSA300_1923	**8,5**	3,52E-24	Yes	41	26	28	110	142	124	12	14	14	ϕSA3 prophage	SAUSA300_1923
SAUSA300_1935	**7,9**	1,89E-22	Yes	69	31	44	206	223	257	22	32	27	ϕSA3 prophage	SAUSA300_1935
SAUSA300_1958	**7,0**	2,98E-12	Yes	47	22	26	139	133	161	14	21	19	ϕSA3 prophage	SAUSA300_1958
SAUSA300_0379	**7,0**	1,06E-87	Yes	27316	26164	19988	161751	165759	173600	23046	23661	24099	alkyl hydroperoxide reductase, subunit F	ahpF
SAUSA300_1966	**6,6**	1,38E-10	Yes	363	103	119	871	724	884	80	141	99	ϕSA3 prophage phage anti-repressor protein	SAUSA300_1966
SAUSA300_0120	**6,6**	1,72E-40	Yes	98	138	105	467	509	646	84	64	90	StaphyloferrinB biosynthesis	sbnC
SAUSA300_1930	**6,3**	8,01E-37	Yes	186	104	119	682	611	821	92	126	109	ϕSA3 prophage, phage tail tape measure protein	SAUSA300_1930
SAUSA300_1936	**6,3**	3,37E-11	Yes	14	5	12	62	52	72	6	11	10	ϕSA3 prophage	SAUSA300_1936
SAUSA300_0380	**6,3**	7,14E-94	Yes	14468	13311	10527	74606	75623	80851	11714	12085	12654	Alkyl hydroperoxide reductase subunit C	ahpC
SAUSA300_1937	**6,3**	2,68E-09	Yes	41	21	5	125	126	130	11	17	24	ϕSA3 prophage	SAUSA300_1937
SAUSA300_1954	**6,2**	1,79E-07	Yes	9	4	4	32	20	40	4	2	5	ϕSA3 prophage	SAUSA300_1954
SAUSA300_1960	**6,1**	3,67E-10	Yes	300	97	81	581	433	521	65	82	72	ϕSA3 prophage	SAUSA300_1960
SAUSA300_1929	**6,1**	4,22E-19	Yes	38	26	27	137	110	145	19	25	18	ϕSA3 prophage	SAUSA300_1929
SAUSA300_1959	**5,9**	3,41E-08	Yes	34	14	15	79	70	56	7	15	7	ϕSA3 prophage	SAUSA300_1959
SAUSA300_1953	**5,8**	8,41E-06	Yes	9	1	4	21	23	25	4	2	4	ϕSA3 prophage	SAUSA300_1953
SAUSA300_1939	**5,6**	3,15E-19	Yes	98	49	48	378	367	492	54	91	67	phϕSA3 prophage putative protease	SAUSA300_1939
SAUSA300_1932	**5,5**	4,00E-17	Yes	60	34	32	155	178	217	22	38	34	ϕSA3 prophage	SAUSA300_1932
SAUSA300_1957	**5,5**	1,89E-10	Yes	96	36	33	178	160	192	27	28	31	ϕSA3 prophage	SAUSA300_1957
SAUSA300_0121	**5,5**	4,77E-33	Yes	57	83	56	253	361	367	57	56	60	StaphyloferrinB biosynthesis	sbnD
SAUSA300_1964	**5,4**	6,91E-08	Yes	152	31	48	274	243	217	31	49	38	ϕSA3 prophage	SAUSA300_1964
SAUSA300_0122	**5,4**	2,87E-29	Yes	112	153	128	458	642	705	119	105	102	StaphyloferrinB biosynthesis	sbnE
SAUSA300_1947	**5,3**	2,19E-06	Yes	14	5	8	33	25	33	6	3	5	ϕSA3 prophage	SAUSA300_1947
SAUSA300_1949	**5,2**	1,61E-06	Yes	12	4	9	36	38	40	7	6	5	ϕSA3 prophage dUTP diphosphatase	dut
SAUSA300_1952	**5,1**	0,000542402	Yes	0	0	5	20	14	27	4	4	0	ϕSA3 prophage	SAUSA300_1952
SAUSA300_1924	**5,1**	0,000400399	Yes	7	1	2	17	11	13	1	2	3	ϕSA3 prophage holin	SAUSA300_1924
SAUSA300_1942	**4,9**	6,74E-10	Yes	53	19	14	256	194	226	29	60	38	ϕSA3 prophage	SAUSA300_1942
SAUSA300_1931	**4,9**	4,12E-05	Yes	16	1	7	24	29	22	4	3	5	ϕSA3 prophage	SAUSA300_1931
SAUSA300_1956	**4,8**	0,000289166	Yes	12	4	4	34	14	31	2	6	3	ϕSA3 prophage	SAUSA300_1956
SAUSA300_1938	**4,8**	6,58E-11	Yes	181	64	88	533	480	608	65	132	112	ϕSA3 prophage putative capsid protein	SAUSA300_1938
SAUSA300_2453	**4,8**	2,26E-11	Yes	1600	3107	1537	116	92	145	22	26	19	ABC transporter, ATP-binding protein	SAUSA300_2453
SAUSA300_1941	**4,8**	6,28E-12	Yes	157	64	75	547	406	478	69	122	91	ϕSA3 prophage, phage terminase, large subunit	SAUSA300_1941
SAUSA300_1962	**4,7**	1,31E-06	Yes	62	16	19	146	133	129	14	40	22	ϕSA3 prophage	SAUSA300_1962
SAUSA300_1943	**4,6**	3,18E-09	Yes	29	16	14	105	115	139	12	29	29	ϕSA3 prophage	SAUSA300_1943
SAUSA300_1961	**4,6**	1,12E-07	Yes	434	133	119	560	512	512	79	119	111	ϕSA3 prophage	SAUSA300_1961
SAUSA300_1948	**4,6**	0,000514385	Yes	7	0	7	23	14	30	4	5	3	ϕSA3 prophage	SAUSA300_1948
SAUSA300_0123	**4,3**	8,13E-22	Yes	146	203	165	497	717	799	160	157	139	StaphyloferrinB biosynthesis	sbnF
SAUSA300_0804	**4,2**	1,76E-12	Yes	55	19	32	1355	1562	1943	282	455	348	SaPI5 putative transcriptional regulator	SAUSA300_0804
SAUSA300_1933	**4,2**	0,000185655	Yes	12	3	4	27	25	34	8	5	4	ϕSA3 prophage	SAUSA300_1933
SAUSA300_1944	**4,2**	2,71E-08	Yes	76	25	30	160	115	169	34	36	29	ϕSA3 prophage, putative phage transcriptional activator	SAUSA300_1944
SAUSA300_1963	**4,2**	7,36E-09	Yes	43	19	16	102	74	96	21	24	17	ϕSA3 prophage	SAUSA300_1963
SAUSA300_0124	**4,0**	1,99E-15	Yes	78	83	99	298	361	495	90	96	92	StaphyloferrinB biosynthesis	sbnG
SAUSA300_0125	**3,9**	2,45E-50	Yes	133	159	140	518	593	665	151	150	154	StaphyloferrinB biosynthesis	sbnG
SAUSA300_1982	**3,8**	6,58E-30	Yes	4733	6643	4105	27780	24628	32361	7985	7376	6713	60 kDa chaperonin	groL
SAUSA300_0807	**3,8**	4,74E-08	Yes	40	16	18	690	751	1202	181	240	234	SaPI5 conserved hypothetical protein	SAUSA300_0807
SAUSA300_1874	**3,7**	1,99E-15	Yes	4480	3200	2007	6981	6586	5127	1808	1473	1556	ferritins family protein	SAUSA300_1874
SAUSA300_1968	**3,6**	5,90E-05	Yes	19	12	11	29	43	25	6	9	8	ϕSA3 prophage transcriptional regulator	SAUSA300_1968
SAUSA300_1983	**3,6**	8,71E-19	Yes	553	961	520	3648	3277	4353	1131	1041	910	10 kDa chaperonin	groES
SAUSA300_1429	**3,6**	0,028913189	No	7	0	1	9	0	13	2	1	0	phiSLT ORF53-like protein	SAUSA300_1429
SAUSA300_0126	**3,5**	7,18E-16	Yes	153	182	165	387	563	685	135	169	149	StaphyloferrinB biosynthesis	sbnI
SAUSA300_1540	**3,5**	9,22E-46	Yes	10046	12487	9723	48138	42777	54266	14277	13491	13205	chaperone protein DnaK	dnaK

SAUSA300_1946	**3,5**	0,000148435	Yes	5	5	4	26	23	27	8	6	5	ϕSA3 prophage	SAUSA300_1946	
SAUSA300_2139	**3,5**	3,83E-21	Yes	110	100	142	624	642	728	174	164	223	sfaA siderophore A putative transporter	SAUSA300_2139	
SAUSA300_1955	**3,4**	0,009625384	Yes	16	3	1	21	9	24	4	4	4	putative endodeoxyribonuclease RusA	SAUSA300_1955	
SAUSA300_1259	**3,4**	1,22E-18	Yes	310	222	190	980	935	875	266	278	259	ImpB/MucB/SamB family protein	SAUSA300_1259	
SAUSA300_0270	**3,3**	1,44E-15	Yes	1219	1332	1175	8050	11233	12126	3118	3497	2805	peptidoglycan hydrolase	lytM	
SAUSA300_0805	**3,2**	5,27E-06	Yes	86	29	44	1396	1526	1748	363	583	403	pathogenicity island protein	SAUSA300_0805	
SAUSA300_1951	**3,2**	0,014638088	No	5	0	1	7	11	12	3	2	2	ϕSA3 prophage	SAUSA300_1951	
SAUSA300_1928	**3,1**	9,19E-19	Yes	334	233	204	657	575	694	209	211	195	ϕSA3 prophage phage minor structural protein	SAUSA300_1928	
SAUSA300_2138	**3,0**	7,53E-35	Yes	1033	971	830	2373	2926	2949	879	893	935	sfaB siderophore A	SAUSA300_2138	
SAUSA300_1940	**3,0**	9,18E-10	Yes	162	83	85	337	302	302	80	109	117	ϕSA3 prophage	SAUSA300_1940	
SAUSA300_2608	**2,9**	3,94E-05	Yes	26	40	36	46	61	87	17	20	25	imidazole glycerol phosphate synthase subunit hisH	hisH	
SAUSA300_0116	**2,9**	7,37E-14	Yes	462	612	501	554	602	779	209	237	207	iron compound ABC transporter, permease protein SirB	sirB	
SAUSA300_0611	**2,9**	0,0664834	No	0	0	0	4	5	3	1	1	0	putative Na+/H+ antiporter, MnhB component	SAUSA300_0611	
SAUSA300_1267	**2,9**	0,000119194	Yes	19	42	35	53	47	62	16	21	16	tryptophan synthase, beta subunit	trpB	
SAUSA300_0812	**2,8**	2,13E-07	Yes	417	283	291	2499	2720	3036	809	1043	995	SaPI5 conserved hypothetical protein	SAUSA300_0812	
SAUSA300_0808	**2,8**	7,51E-08	Yes	19	4	4	282	284	322	84	116	108	SaPI5 conserved hypothetical protein	SAUSA300_0808	
SAUSA300_1384	**2,8**	0,076188985	No	0	0	0	3	5	1	0	0	0	phiSLT ORF100b-like protein, holin	SAUSA300_1384	
SAUSA300_2316	**2,8**	2,59E-06	Yes	86	168	205	374	417	418	121	130	168	acetyltransferase, GNAT family	SAUSA300_2316	
SAUSA300_1915	**2,7**	0,035000236	No	2	4	7	7	25	12	4	5	3	ϕSA3 prophage	SAUSA300_1915	
SAUSA300_1945	**2,7**	0,000511964	Yes	7	7	11	26	32	44	12	12	11	ϕSA3 prophage	SAUSA300_1945	
SAUSA300_0268	**2,7**	2,63E-17	Yes	122	131	125	500	609	597	171	240	223	putative drug transporter	SAUSA300_0268	
SAUSA300_1542	**2,6**	1,31E-19	Yes	1035	1196	978	4132	3264	3777	1557	1318	1350	heat-inducible transcription repressor HrcA	hrcA	
SAUSA300_1539	**2,6**	4,06E-11	Yes	474	657	852	3471	3322	4637	1359	1332	1605	chaperone protein DnaJ	dnaJ	
SAUSA300_0113	**2,6**	0,022280543	No	304605	486402	340505	75164	82216	101920	14383	49815	21401	immunoglobulin G binding protein A precursor	SAUSA300_0113	
SAUSA300_1925	**2,6**	0,000991734	Yes	22	22	28	53	41	87	24	24	19	ϕSA3 prophage	SAUSA300_1925	
SAUSA300_0381	**2,5**	5,28E-32	Yes	1455	1417	1204	5960	5340	6640	2555	2380	2214	putative NAD(P)H-flavin oxidoreductase	SAUSA300_0381	
SAUSA300_2253	**2,5**	3,23E-10	Yes	3613	4111	2769	15723	18523	21301	6615	6954	8386	secretory antigen precursor SsaA	ssaA	
SAUSA300_0718	**2,4**	8,68E-08	Yes	186	357	259	261	230	309	102	109	110	iron compound ABC transporter, permease	SAUSA300_0718	
SAUSA300_2607	**2,4**	0,000119156	Yes	29	33	60	64	95	129	39	38	38	phosphoribosylformimino-5-aminoimidazole carboxami	hisA	
SAUSA300_2409	**2,4**	3,08E-05	Yes	47	93	96	109	126	117	42	44	54	oligopeptide ABC transporter, permease protein	SAUSA300_2409	
SAUSA300_1926	**2,4**	0,066838624	No	9	5	6	9	16	6	3	4	3	ϕSA3 prophage	SAUSA300_1926	
SAUSA300_0821	**2,4**	9,30E-19	Yes	3252	3621	2748	5430	5418	6357	2590	2340	2253	SUF system FeS assembly protein, NifU family	SAUSA300_0821	
SAUSA300_0806	**2,4**	0,000882778	Yes	12	4	8	135	171	154	39	78	66	SaPI5 conserved hypothetical protein	SAUSA300_0806	
SAUSA300_1893	**2,4**	2,07E-08	Yes	482	699	782	1250	1368	1573	591	557	594	NH(3)-dependent NAD+ synthetase	nadE	
SAUSA300_0809	**2,4**	0,00018522	Yes	308	159	176	3194	3320	3784	1158	1640	1397	SaPI putative DNA primase	SAUSA300_0809	
SAUSA300_0820	**2,4**	9,19E-14	Yes	8124	9260	6794	14053	13674	15703	6411	6316	5507	cysteine desulfurases, SufS subfamily subfamily	sufS	
SAUSA300_1981	**2,4**	0,00145898	Yes	5	5	8	72	32	43	20	20	20	ϕSA3 prophage terminase family protein	SAUSA300_1981	
SAUSA300_2051	**2,3**	4,94E-09	Yes	150	168	125	636	726	765	276	334	284	conserved hypothetical protein	SAUSA300_2051	
SAUSA300_0224	**2,3**	1,25E-06	Yes	549	335	338	1459	2074	2047	701	976	673	staphylocoagulase precursor	coa	
SAUSA300_2606	**2,3**	5,25E-07	Yes	45	66	67	117	122	142	48	50	62	imidazole glycerol phosphate synthase subunit hisF	hisF	
SAUSA300_1538	**2,3**	8,63E-22	Yes	846	986	1185	4303	4573	4915	1915	1993	2019	ribosomal protein L11 methyltransferase	prmA	
SAUSA300_0859	**2,3**	1,04E-08	Yes	2373	2528	1498	5521	4091	6066	2593	1974	2120	NADH-dependent flavin oxidoreductase	SAUSA300_0859	
SAUSA300_0117	**2,3**	1,69E-05	Yes	1683	2487	2043	1630	1456	1724	625	707	701	iron compound ABC transporter, iron compound-bindin	sirA	
SAUSA300_1383	**2,3**	0,003650724	Yes	14	15	11	23	20	46	13	11	13	phiSLT ORF484-like protein, lysin	SAUSA300_1383	
SAUSA300_2137	**2,3**	2,10E-17	Yes	529	500	434	958	1208	939	467	420	452	sfaC siderophore A	SAUSA300_2137	
SAUSA300_0135	**2,3**	7,59E-10	Yes	1393	1441	1111	1075	1066	1245	477	514	468	Superoxide dismutase (Mn/Fe family)	SAUSA300_0135	
SAUSA300_2454	**2,3**	0,001161293	Yes	594	664	769	24	18	35	11	11	11	membrane spanning protein	SAUSA300_2454	
SAUSA300_0747	**2,3**	5,20E-11	Yes	2580	3502	3917	8485	7787	10478	3860	3736	3985	thioredoxin-disulfide reductase	trxB	
SAUSA300_1715	**2,3**	0,037558746	No	281	572	638	1171	1161	1668	511	474	588	riboflavin biosynthesis protein	ribD	
SAUSA300_1891	**2,3**	2,67E-09	Yes	400	405	404	498	602	683	287	267	225	conserved hypothetical protein	SAUSA300_1891	
SAUSA300_1033	**2,3**	5,04E-09	Yes	210	237	168	309	273	311	133	129	129	iron/heme permease	SAUSA300_1033	
SAUSA300_2135	**2,3**	1,13E-11	Yes	563	745	555	1371	1499	1658	632	710	644	htsB iron compound ABC transporter, permease protein	SAUSA300_2135	K02015
SAUSA300_0394	**2,3**	7,12E-11	Yes	67	64	69	378	356	488	178	174	187	FAD/NAD(P)-binding Rossmann fold Superfamily	SAUSA300_0394	
SAUSA300_0148	**2,2**	0,15212013	No	2	0	0	0	2	3	0	0	0	conserved hypothetical protein	SAUSA300_0148	
SAUSA300_1903	**2,2**	2,68E-05	Yes	76	75	75	285	358	356	115	172	147	conserved hypothetical protein	SAUSA300_1903	
SAUSA300_1580	**2,2**	7,20E-13	Yes	1321	984	827	2797	2274	2671	1239	1109	1078	bacterial luciferase family protein	SAUSA300_1580	
SAUSA300_2474	**2,2**	2,85E-11	Yes	224	309	205	964	832	963	383	432	406	conserved hypothetical protein	SAUSA300_2474	
SAUSA300_1028	**2,2**	1,45E-10	Yes	169	193	128	215	225	235	102	91	106	iron transport associated domain protein	SAUSA300_1028	
SAUSA300_0810	**2,2**	8,43E-05	Yes	81	71	68	703	658	801	259	374	323	conserved hypothetical protein	SAUSA300_0810	

SAUSA300_1448	**2,2**	2,75E-33	Yes	1280	1328	1364	2960	2946	3234	1486	1350	1299	transcriptional regulator, Fur family	SAUSA300_1448
SAUSA300_0324	**2,2**	9,59E-13	Yes	158	160	170	473	442	594	221	230	230	conserved hypothetical protein	SAUSA300_0324
SAUSA300_0445	**2,2**	0,000454503	Yes	579	1299	1040	743	665	847	378	313	301	glutamate synthase, large subunit	gltB
SAUSA300_2415	**2,2**	0,119540827	No	2	8	7	4	9	7	6	1	1	conserved hypothetical protein	SAUSA300_2415
SAUSA300_1032	**2,2**	8,88E-06	Yes	115	231	167	235	259	291	120	101	130	putative iron compound ABC transporter, iron compour	SAUSA300_1032
SAUSA300_2366	**2,2**	7,47E-05	Yes	62	67	48	82	72	59	31	34	30	gamma-hemolysin component C	hlgC
SAUSA300_2092	**2,2**	1,58E-08	Yes	2887	2242	1772	5250	4068	4170	1903	1953	2246	general stress protein 20U	dps
SAUSA300_2127	**2,2**	0,006620375	Yes	72	52	72	168	212	112	63	71	80	conserved hypothetical protein	SAUSA300_2127
SAUSA300_2422	**2,2**	4,79E-16	Yes	512	641	652	1792	1566	2030	852	793	850	oxidoreductase, short-chain dehydrogenase/reductase f	SAUSA300_2422
SAUSA300_0207	**2,1**	2,93E-08	Yes	102	110	98	470	527	510	235	250	214	conserved hypothetical protein	SAUSA300_0207
SAUSA300_2348	**2,1**	1,85E-10	Yes	115	179	146	561	588	602	287	271	261	conserved hypothetical protein	SAUSA300_2348
SAUSA300_2034	**2,1**	0,015119031	No	10	22	22	23	32	28	16	9	11	K+-transporting ATPase, A subunit	kdpA
SAUSA300_0819	**2,1**	2,42E-18	Yes	6554	7546	5947	9926	10494	10295	5107	4898	4509	FeS assembly protein SufD	sufD
SAUSA300_0328	**2,1**	6,45E-18	Yes	165	214	200	619	604	736	315	304	320	lipoate-protein ligase A family protein	SAUSA300_0328
SAUSA300_2475	**2,1**	1,79E-07	Yes	262	389	236	1150	1161	1201	577	565	539	conserved hypothetical protein	SAUSA300_2475
SAUSA300_0327	**2,1**	1,03E-12	Yes	210	244	225	670	717	865	341	359	384	conserved hypothetical protein	SAUSA300_0327
SAUSA300_2364	**2,1**	2,79E-05	Yes	17391	12466	8400	6278	6926	6866	3212	3279	3063	IgG-binding protein SBI	sbi
SAUSA300_2387	**2,0**	5,22E-18	Yes	627	728	641	2505	2635	2814	1313	1336	1220	NAD dependent epimerase/dehydratase family protein	SAUSA300_2387
SAUSA300_0037	**2,0**	2,30E-14	Yes	629	558	478	1578	1826	1801	889	859	781	cassette chromosome recombinase B	ccrB
SAUSA300_2613	**2,0**	0,005003476	Yes	79	73	58	100	70	74	24	42	49	conserved hypothetical protein	SAUSA300_2613
SAUSA300_2158	**2,0**	0,208731303	No	2	3	4	7	5	4	3	1	2	conserved hypothetical protein	SAUSA300_2158
SAUSA300_2485	**2,0**	4,66E-08	Yes	102	157	147	380	352	464	193	190	197	methylated DNA-protein cysteine methyltransferase	SAUSA300_2485
SAUSA300_0818	**2,0**	3,20E-11	Yes	4941	5694	4438	7148	6762	7009	3721	3460	2989	FeS assembly ATPase SufC	sufC
SAUSA300_0326	**2,0**	1,80E-13	Yes	127	170	175	481	480	495	231	237	246	conserved hypothetical protein	SAUSA300_0326
SAUSA300_0490	**2,0**	4,78E-06	Yes	844	1136	1367	3151	3248	3973	1634	1575	1832	33 kDa chaperonin (Heat shock protein 33-like protein)	SAUSA300_0490
SAUSA300_1394	**2,0**	0,264206988	No	0	1	0	4	2	0	1	1	0	conserved hypothetical phage protein	SAUSA300_1394
SAUSA300_1892	**2,0**	3,09E-05	Yes	529	532	686	561	609	642	301	264	317	conserved hypothetical protein	SAUSA300_1892
SAUSA300_1541	**2,0**	1,27E-12	Yes	2646	2998	2323	7633	6219	6687	3630	3150	3338	co-chaperone GrpE	grpE
SAUSA300_0269	**2,0**	6,39E-09	Yes	188	219	172	390	363	501	203	198	219	choloylglycine hydrolase family protein	SAUSA300_0269
SAUSA300_2400	**2,0**	2,37E-15	Yes	1061	1248	1100	2336	2207	2647	1259	1207	1103	glutamyl-aminopeptidase	SAUSA300_2400
SAUSA300_1397	**2,0**	0,002872498	Yes	31	25	18	60	41	40	27	22	20	phiSLT ORF213-like protein, major tail protein	SAUSA300_1397
SAUSA300_1400	**2,0**	0,048382544	No	12	3	14	16	20	18	9	9	7	phiSLT ORF92-like protein, uncharacterized phage prote	SAUSA300_1400
SAUSA300_0043	**2,0**	0,101292643	No	14	7	4	9	23	13	7	6	6	conserved hypothetical protein	SAUSA300_0043
SAUSA300_2048	**2,0**	9,11E-08	Yes	88	103	67	153	153	152	74	72	80	hydroxyethylthiazole kinase	thiM
SAUSA300_0446	**2,0**	1,43E-05	Yes	405	678	623	430	446	618	256	250	234	glutamate synthase, small subunit	gltD
SAUSA300_2498	**2,0**	2,38E-05	Yes	276	337	347	624	516	801	307	308	353	squalene synthase	crtN
SAUSA300_0742	**2,0**	4,32E-09	Yes	2950	3222	3122	9151	9394	9724	4553	4967	4654	excinuclease ABC, A subunit	uvrA
SAUSA300_0312	**2,0**	0,00564668	Yes	21	27	22	36	41	59	21	29	18	indigoidine synthase family protein	SAUSA300_0312
SAUSA300_0310	**2,0**	2,17E-05	Yes	2671	4258	3931	5345	5047	6422	2840	3259	2375	perfringolysin O regulator protein	pfoR
SAUSA300_0842	**2,0**	8,26E-12	Yes	735	793	658	1525	1321	1622	834	717	727	conserved hypothetical protein	SAUSA300_0842
SAUSA300_0146	**2,0**	1,32E-05	Yes	121	189	144	150	162	149	74	79	80	conserved hypothetical protein	SAUSA300_0146
SAUSA300_1485	**2,0**	0,022596803	No	16	23	20	16	36	46	17	19	12	conserved hypothetical protein	SAUSA300_1485
SAUSA300_2500	**2,0**	0,000156494	Yes	320	387	330	703	696	868	365	426	352	glycosyl transferase	SAUSA300_2500
SAUSA300_2047	**1,9**	1,58E-05	Yes	72	85	71	146	110	164	72	76	68	thiamine-phosphate pyrophosphorylase	thiE
SAUSA300_0138	**1,9**	0,008032275	Yes	29	18	29	52	32	35	25	19	16	purine nucleoside phosphorylase	deoD
SAUSA300_2501	**1,9**	3,56E-05	Yes	544	535	545	955	872	1105	488	577	423	phytoene dehydrogenase	SAUSA300_2501
SAUSA300_2600	**1,9**	0,005059075	Yes	36	57	39	46	45	30	20	19	21	intercellular adhesion protein A	icaA
SAUSA300_1054	**1,9**	0,144544363	No	7	18	13	13	18	16	9	6	6	conserved hypothetical protein	SAUSA300_1054
SAUSA300_0147	**1,9**	1,79E-07	Yes	272	387	322	374	358	481	203	221	204	5' nucleotidase family protein	SAUSA300_0147
SAUSA300_1608	**1,9**	0,034005652	No	14	18	11	24	14	33	8	14	14	DNA repair protein RadC	SAUSA300_1608
SAUSA300_2318	**1,9**	0,001187531	Yes	40	71	90	316	279	526	180	212	186	acetyltransferase, GNAT family	SAUSA300_2318
SAUSA300_2408	**1,9**	6,47E-09	Yes	155	156	182	233	243	274	135	130	126	oligopeptide ABC transporter, ATP-binding protein	SAUSA300_2408
SAUSA300_2411	**1,9**	5,57E-11	Yes	424	601	550	740	739	797	355	430	405	oligopeptide permease, peptide-binding protein	opp-1A
SAUSA300_1213	**1,9**	0,30662715	No	3	4	1	7	0	6	1	4	0	conserved hypothetical protein	SAUSA300_1213
SAUSA300_2235	**1,9**	2,98E-05	Yes	1728	2784	2773	5095	5119	7094	3199	3461	2341	iron compound ABC transporter, iron compound-binding	SAUSA300_2235
SAUSA300_1182	**1,9**	1,07E-07	Yes	3830	4791	3152	4902	4007	4945	2595	2182	2475	pyruvate ferredoxin oxidoreductase, alpha subunit	SAUSA300_1182
SAUSA300_0710	**1,9**	0,000126065	Yes	81	73	51	113	160	114	72	67	60	hypothetical protein	SAUSA300_0710
SAUSA300_1282	**1,9**	0,008171693	Yes	17	15	23	59	34	49	27	27	20	phosphate ABC transporter, permease protein PstC	pstC
SAUSA300_0556	**1,9**	4,13E-10	Yes	4494	4610	3716	8179	7835	9211	4670	4517	4125	SIS domain protein	SAUSA300_0556

SAUSA300_0237	**1,9**	3,88E-13	Yes	472	511	424	1173	1269	1471	684	674	710	inosine-uridine preferring nucleoside hydrolase	SAUSA300_0237
SAUSA300_2345	**1,9**	0,063399191	No	17	30	30	49	52	65	25	32	26	nitrite reductase [NAD(P)H	nirD
SAUSA300_1513	**1,9**	2,81E-07	Yes	21429	20890	14491	26403	25924	26306	16059	14355	11038	superoxide dismutase (Mn/Fe family)	SAUSA300_1513
SAUSA300_0741	**1,9**	4,41E-08	Yes	1371	1659	1482	4702	4823	5003	2470	2701	2503	excinuclease ABC, B subunit	uvrB
SAUSA300_1029	**1,9**	0,000167523	Yes	3872	4331	3120	2002	2008	2257	1079	1105	1103	iron transport associated domain protein	SAUSA300_1029
SAUSA300_1030	**1,9**	0,000240689	Yes	109	186	152	162	178	158	75	85	99	iron transport associated domain protein	SAUSA300_1030
SAUSA300_0066	**1,9**	0,324371051	No	2	1	1	1	5	3	1	1	1	arginine repressor	argR
SAUSA300_2319	**1,9**	0,000437074	Yes	69	136	168	643	532	819	338	342	368	pyridine nucleotide-disulfide oxidoreductase	SAUSA300_2319
SAUSA300_1728	**1,9**	1,59E-08	Yes	515	650	475	1340	1107	1338	737	624	649	oxidoreductase, aldo/keto reductase family	SAUSA300_1728
SAUSA300_0236	**1,9**	0,001220481	Yes	458	433	268	633	611	657	367	351	280	PTS system, IIBC components	SAUSA300_0236
SAUSA300_0036	**1,9**	0,186963485	No	3	7	1	9	16	10	4	7	4	conserved hypothetical protein	SAUSA300_0036
SAUSA300_0481	**1,9**	8,80E-09	Yes	2733	3564	3301	4610	4589	5417	2537	2600	2645	transcription-repair coupling factor	mfd
SAUSA300_2410	**1,9**	8,86E-05	Yes	103	122	152	125	144	161	71	71	85	oligopeptide ABC transporter, permease protein	SAUSA300_2410
SAUSA300_2528	**1,9**	5,07E-11	Yes	692	630	607	629	681	569	352	334	314	conserved hypothetical protein	SAUSA300_2528
SAUSA300_1461	**1,9**	0,284034777	No	5	3	6	4	5	3	1	2	2	conserved hypothetical protein	SAUSA300_1461
SAUSA300_0055	**1,9**	1,62E-06	Yes	93	145	114	235	237	241	121	136	123	alcohol dehydrogenase, zinc-containing	SAUSA300_0055
SAUSA300_1178	**1,9**	1,94E-11	Yes	10913	9742	8495	23354	21759	21721	11432	12736	11601	recombinase A protein	recA
SAUSA300_1733	**1,9**	1,52E-08	Yes	214	267	233	528	523	642	304	311	295	conserved hypothetical protein	SAUSA300_1733
SAUSA300_2407	**1,9**	4,40E-07	Yes	153	205	153	241	325	274	146	146	154	oligopeptide ABC transporter, ATP-binding protein	SAUSA300_2407
SAUSA300_2049	**1,8**	7,00E-05	Yes	60	85	40	153	113	136	70	68	78	phosphomethylpyrimidine kinase	thiD
SAUSA300_2147	**1,8**	3,12E-12	Yes	2065	2192	1667	5200	4756	5755	3029	2864	2595	alcohol dehydrogenase, zinc-containing	SAUSA300_2147
SAUSA300_1894	**1,8**	0,000381857	Yes	357	491	686	901	949	1239	499	525	627	nicotinate phosphoribosyltransferase	SAUSA300_1894
SAUSA300_2382	**1,8**	1,27E-09	Yes	202	185	168	418	478	479	266	236	242	conserved hypothetical protein	SAUSA300_2382
SAUSA300_0534	**1,8**	4,24E-14	Yes	1803	1719	1358	2843	2851	3252	1600	1646	1619	amidohydrolase	SAUSA300_0534
SAUSA300_0227	**1,8**	0,200344122	No	2	3	6	9	7	10	5	4	4	acyl-CoA dehydrogenase FadD	fadD
SAUSA300_0275	**1,8**	0,217147612	No	16	12	14	21	34	12	15	5	12	putative membrane protein	SAUSA300_0275
SAUSA300_0396	**1,8**	0,077378948	No	12	10	8	23	9	30	15	5	13	exotoxin 7	set7
SAUSA300_2622	**1,8**	1,06E-05	Yes	1135	1040	608	2089	2098	2137	1255	1084	1103	conserved hypothetical protein	SAUSA300_2622
SAUSA300_1393	**1,8**	0,000289539	Yes	71	115	105	156	142	197	87	80	102	phiSLT ORF2067-like protein, phage tail tape measure p	SAUSA300_1393
SAUSA300_1460	**1,8**	3,33E-09	Yes	1342	1823	1437	3269	2869	3647	1838	1750	1798	peptidase, M20/M25/M40 family	SAUSA300_1460
SAUSA300_0345	**1,8**	0,016007985	No	53	129	96	102	81	154	60	52	69	Tat-translocated enzyme	SAUSA300_0345
SAUSA300_2633	**1,8**	0,014203951	No	34	21	28	40	34	30	16	23	18	ABC transporter, ATP-binding protein	SAUSA300_2633
SAUSA300_2514	**1,8**	0,002444944	Yes	29	42	26	66	65	58	27	33	42	conserved hypothetical protein	SAUSA300_2514
SAUSA300_1714	**1,8**	0,206158563	No	191	267	270	455	419	529	229	216	244	riboflavin synthase, alpha subunit	ribE
SAUSA300_0313	**1,8**	0,010553599	No	36	36	39	37	79	41	28	28	26	putative nucleoside permease NupC	SAUSA300_0313
SAUSA300_2569	**1,8**	0,040519936	No	12	40	16	27	36	31	16	15	19	ornithine carbamoyltransferase	arcB
SAUSA300_0105	**1,8**	0,000375921	Yes	351	523	343	527	433	688	319	308	285	peptidase, M20/M25/M40 family	SAUSA300_0105
SAUSA300_2537	**1,8**	2,24E-06	Yes	3166	3701	2513	3915	3586	4109	2267	2307	1887	L-lactate dehydrogenase	SAUSA300_2537
SAUSA300_2611	**1,8**	0,000169855	Yes	117	123	92	142	115	130	72	78	65	histidinol dehydrogenase hisD	hisD
SAUSA300_1387	**1,8**	0,220364365	No	3	1	7	9	9	13	5	5	6	phiSLT ORF129-like protein	SAUSA300_1387
SAUSA300_1713	**1,8**	0,236740591	No	370	542	505	884	951	1109	489	428	535	riboflavin biosynthesis protein	ribBA
SAUSA300_0879	**1,8**	0,198025477	No	0	1	6	14	7	13	7	6	5	isopropylmalate synthase-related protein	SAUSA300_0879
SAUSA300_1692	**1,8**	0,223625142	No	21	19	20	37	68	53	28	25	26	conserved hypothetical protein	SAUSA300_1692
SAUSA300_0557	**1,8**	6,14E-06	Yes	546	619	477	391	435	404	248	231	211	HAD-superfamily hydrolase, subfamily IA, variant 1	SAUSA300_0557
SAUSA300_1735	**1,8**	7,66E-07	Yes	305	465	464	730	699	874	414	432	452	O-succinylbenzoic acid synthetase	menC
SAUSA300_2033	**1,8**	7,43E-05	Yes	93	129	133	168	232	185	113	102	110	K+-transporting ATPase, B subunit	kdpB
SAUSA300_0552	**1,8**	6,28E-09	Yes	2890	3465	2401	4286	4086	4581	2687	2448	2190	conserved hypothetical protein	SAUSA300_0552
SAUSA300_2473	**1,8**	3,19E-07	Yes	1628	2261	1721	5999	5267	6569	3566	3263	3285	conserved hypothetical protein	SAUSA300_2473
SAUSA300_2605	**1,8**	0,002456657	Yes	55	74	82	99	86	126	59	64	53	histidine biosynthesis bifunctional protein hisIE	hisIE
SAUSA300_0822	**1,8**	4,89E-21	Yes	10417	10676	9071	13812	13738	12960	8041	7704	7362	FeS assembly protein SufB	sufB
SAUSA300_0325	**1,7**	9,91E-06	Yes	93	77	109	269	216	291	145	152	147	glycine cleavage H-protein	SAUSA300_0325
SAUSA300_0799	**1,7**	4,98E-07	Yes	326	286	259	811	897	849	493	497	463	integrase	int
SAUSA300_0140	**1,7**	1,00E-06	Yes	799	1120	1154	2964	2982	3885	1832	2010	1753	deoxyribose-phosphate aldolase	deoC
SAUSA300_1183	**1,7**	4,72E-09	Yes	2210	2345	1754	2705	2180	2638	1491	1296	1506	pyruvate ferredoxin oxidoreductase, beta subunit	SAUSA300_1183
SAUSA300_0056	**1,7**	0,344057674	No	7	3	0	4	5	9	2	4	3	conserved hypothetical protein	SAUSA300_0056
SAUSA300_0141	**1,7**	8,95E-09	Yes	2074	2790	2779	6855	7010	8046	4075	4538	3915	phosphopentomutase	deoB
SAUSA300_0720	**1,7**	0,000126479	Yes	129	182	167	123	142	148	84	79	72	putative iron compound ABC transporter, ATP-binding p	SAUSA300_0720
SAUSA300_1232	**1,7**	2,41E-08	Yes	12084	12038	9119	18975	14443	17565	9660	9554	10062	catalase	SAUSA300_1232
SAUSA300_2404	**1,7**	2,13E-07	Yes	388	576	515	1551	1506	1698	943	932	860	conserved hypothetical protein	SAUSA300_2404

SAUSA300_2206	**1,7**	0,187973562	No	7	16	14	39	68	59	36	33	19	conserved hypothetical protein	SAUSA300_2206	
SAUSA300_0495	**1,7**	0,357691052	No	7	4	23	9	11	12	4	4	6	hypothetical protein	SAUSA300_0495	
SAUSA300_1922	**1,7**	0,000216558	Yes	453	335	322	163	178	189	105	105	95	staphylokinase precursor	sak	
SAUSA300_1402	**1,7**	0,051132353	No	21	25	13	27	34	30	16	19	15	phiSLT ORF257-like protein, putative prophage protease	SAUSA300_1402	
SAUSA300_2136	**1,7**	0,001114325	Yes	1147	1585	1342	2735	2387	2850	1486	1532	1560	htsA iron compound ABC transporter, iron compound-b	SAUSA300_2136	K02016
SAUSA300_2236	**1,7**	8,32E-08	Yes	482	608	520	1310	1168	1633	734	828	823	conserved hypothetical protein	SAUSA300_2236	
SAUSA300_0491	**1,7**	7,00E-05	Yes	9335	13683	9114	20782	18534	24498	13705	11958	11226	cysteine synthase A	cysK	
SAUSA300_2076	**1,7**	8,70E-05	Yes	3827	4420	3522	5926	5635	7426	3907	3775	3301	aldehyde dehydrogenase family protein	SAUSA300_2076	
SAUSA300_2570	**1,7**	0,06543476	No	21	30	20	34	41	31	21	17	21	arginine deiminase	arcA	
SAUSA300_2534	**1,7**	0,002104597	Yes	1159	2371	1588	2446	2416	3070	1527	1547	1503	3-methyl-2-oxobutanoate hydroxymethyltransferase	panB	
SAUSA300_0613	**1,7**	0,045888879	No	16	14	25	26	56	56	25	30	23	putative Na+/H+ antiporter, MnhD component	SAUSA300_0613	
SAUSA300_0129	**1,7**	5,74E-07	Yes	555	583	530	1402	1334	1775	838	885	913	Acetoin(diacetyl) reductase	SAUSA300_0129	
SAUSA300_1389	**1,7**	0,003187266	Yes	64	111	55	116	142	191	91	81	90	phiSLT ORF636-like protein	SAUSA300_1389	
SAUSA300_0038	**1,7**	9,37E-05	Yes	153	130	106	150	147	169	96	93	86	cassette chromosome recombinase A	ccrA	
SAUSA300_0942	**1,7**	0,325839682	No	3	5	2	4	14	3	4	3	3	conserved hypothetical protein	SAUSA300_0942	
SAUSA300_2221	**1,7**	0,000816788	Yes	67	101	134	195	246	285	146	139	141	molybdopterin converting factor, subunit 1	moaD	
SAUSA300_0346	**1,7**	0,000426316	Yes	148	196	134	165	137	182	115	84	90	putative membrane protein	SAUSA300_0346	
SAUSA300_1192	**1,7**	8,70E-05	Yes	205	303	308	1000	976	1210	669	684	534	glycerol kinase	glpK	
SAUSA300_2539	**1,7**	0,026773339	No	62	100	99	69	77	157	53	75	51	aminotransferase	SAUSA300_2539	
SAUSA300_0438	**1,7**	2,72E-05	Yes	6774	9174	7514	10156	11195	13689	6844	7071	6921	CHAP domain family	SAUSA300_0438	
SAUSA300_1385	**1,7**	0,332092897	No	2	7	1	6	5	10	4	1	5	phiSLT ORF 99-like protein	SAUSA300_1385	
SAUSA300_0025	**1,7**	2,05E-05	Yes	1219	1741	1237	902	845	1023	585	569	511	5'-nucleotidase family protein	SAUSA300_0025	
SAUSA300_0900	**1,7**	0,076414066	No	24	16	28	52	47	28	29	17	28	putative competence protein	SAUSA300_0900	
SAUSA300_1782	**1,6**	4,29E-09	Yes	851	1136	973	1776	1697	1635	994	1030	1066	ferrochelatase	hemH	
SAUSA300_0825	**1,6**	6,89E-05	Yes	1039	1455	1173	2420	2261	2756	1478	1494	1519	oxidoreductase, 2-nitropropane dioxygenase family	SAUSA300_0825	
SAUSA300_0610	**1,6**	0,134406262	No	0	0	0	10	2	16	0	0	0	putative Na+/H+ antiporter, MnhA component	SAUSA300_0610	
SAUSA300_2226	**1,6**	5,38E-06	Yes	339	434	351	666	539	707	404	365	392	molybdenum cofactor biosynthesis protein B	moaB	
SAUSA300_0609	**1,6**	0,151203284	No	0	0	0	3	5	7	0	0	0	phage integrase family protein	SAUSA300_0609	
SAUSA300_0453	**1,6**	0,001916683	Yes	1037	1632	1968	3205	3223	4776	2334	2319	2113	conserved hypothetical protein	SAUSA300_0453	
SAUSA300_0350	**1,6**	0,026600475	No	31	52	47	82	56	65	48	34	41	transcriptional regulator, Cro/CI family-related protein	SAUSA300_0350	
SAUSA300_2347	**1,6**	0,320707046	No	2	7	25	17	27	21	11	16	8	nitrite reductase transcriptional regulator NirR	nirR	
SAUSA300_0553	**1,6**	2,44E-07	Yes	2106	1979	1622	2435	2315	2505	1653	1356	1421	conserved hypothetical protein	SAUSA300_0553	
SAUSA300_0948	**1,6**	1,29E-08	Yes	4941	5515	5254	10471	9906	12559	6658	7331	6140	naphthoate synthase	menB	
SAUSA300_2610	**1,6**	0,007361371	Yes	79	99	92	74	99	98	46	65	52	histidinol-phosphate aminotransferase hisC	hisC	
SAUSA300_2050	**1,6**	0,022607422	No	41	62	39	77	86	62	40	38	56	TENA/THI-4 family protein	SAUSA300_2050	
SAUSA300_1237	**1,6**	7,00E-05	Yes	3576	2542	2798	7816	7192	7197	4570	4817	4210	LexA repressor	lexA	
SAUSA300_2540	**1,6**	3,11E-06	Yes	13066	9464	9558	29448	27263	27256	16978	18160	16393	fructose-bisphosphate aldolase class-I	SAUSA300_2540	
SAUSA300_0063	**1,6**	0,456862412	No	0	1	1	4	7	0	2	2	1	cyclic nucleotide-binding domain protein	SAUSA300_0063	
SAUSA300_1396	**1,6**	0,0102641	No	45	68	53	70	88	109	60	49	53	phiSLT ORF151-like protein, major tail protein	SAUSA300_1396	
SAUSA300_0789	**1,6**	6,47E-05	Yes	2100	1782	1351	3353	3471	3633	2453	2202	1787	putative thioredoxin	SAUSA300_0789	
SAUSA300_1049	**1,6**	1,13E-11	Yes	2597	2642	2302	5117	5288	5219	3551	3138	2969	glutamate racemase	murI	
SAUSA300_1294	**1,6**	0,42843697	No	14	1	0	4	5	13	7	1	3	conserved hypothetical protein	SAUSA300_1294	
SAUSA300_0817	**1,6**	4,54E-10	Yes	558	605	649	1529	1841	1638	1049	1063	984	putative membrane protein	SAUSA300_0817	
SAUSA300_1206	**1,6**	0,465004215	No	0	1	1	1	0	1	1	1	0	conserved hypothetical protein	SAUSA300_1206	
SAUSA300_2503	**1,6**	0,054652663	No	1052	501	490	2072	2148	2066	1686	1075	1090	secretory antigen precursor SsaA	SAUSA300_2503	
SAUSA300_2554	**1,6**	0,000754937	Yes	1216	1830	1040	1657	1582	1678	1057	994	1004	sulfite reductase flavoprotein	SAUSA300_2554	
SAUSA300_2594	**1,6**	1,34E-05	Yes	86	105	86	206	207	207	132	121	133	methionine-S-sulfoxide reductase	msrA	
SAUSA300_1781	**1,6**	4,80E-11	Yes	1479	1774	1667	3016	2772	2949	1858	1745	1862	protoporphyrinogen oxidase	hemG	
SAUSA300_2224	**1,6**	0,000257806	Yes	517	854	744	1325	1195	1622	923	821	840	molybdopterin biosynthesis protein A	moeA	
SAUSA300_0261	**1,6**	0,002534146	Yes	186	138	123	180	110	203	103	105	104	conserved hypothetical protein	SAUSA300_0261	
SAUSA300_1927	**1,6**	0,204255178	No	14	7	12	17	18	15	8	12	10	phi77 ORF109-like protein	SAUSA300_1927	
SAUSA300_1641	**1,6**	3,76E-05	Yes	212	318	274	1307	1197	1588	861	883	820	citrate synthase II	gltA	
SAUSA300_2414	**1,6**	0,04092367	No	79	56	32	79	70	67	33	49	50	conserved hypothetical protein	SAUSA300_2414	
SAUSA300_2115	**1,6**	0,438995843	No	3	7	4	3	2	4	1	4	0	IS1181, transposase	tnp	
SAUSA300_2027	**1,6**	3,61E-09	Yes	2244	2360	2125	3176	3311	3468	2244	2060	1954	alanine racemase	alr	
SAUSA300_1416	**1,6**	0,470189473	No	0	1	1	0	2	10	2	2	1	phiSLT ORF 81b-like protein	SAUSA300_1416	
SAUSA300_0843	**1,6**	1,39E-10	Yes	1168	1073	991	1548	1465	1501	1011	953	885	conserved hypothetical protein	SAUSA300_0843	
SAUSA300_1398	**1,6**	0,134092099	No	14	11	9	20	23	33	18	13	15	phiSLT ORF123-like protein	SAUSA300_1398	
SAUSA300_2365	**1,6**	0,062903433	No	40	29	19	43	59	49	31	35	26	gamma-hemolysin component A	hlgA	

SAUSA300_2553	**1,6**	0,001000604	Yes	844	921	573	1077	911	1226	703	645	672	putative siroheme synthase	SAUSA300_2553	
SAUSA300_1297	**1,6**	3,59E-05	Yes	264	385	356	842	935	951	590	611	516	conserved hypothetical protein	SAUSA300_1297	
SAUSA300_2609	**1,6**	0,067588276	No	22	38	44	59	36	43	25	27	34	imidazole glycerol phosphate dehydratase hisB	hisB	
SAUSA300_2317	**1,6**	1,05E-06	Yes	1593	1567	1528	5178	5083	5473	3425	3616	2896	putative zinc-binding dehydrogenase	SAUSA300_2317	
SAUSA300_0956	**1,6**	9,61E-05	Yes	264	255	282	272	356	311	191	199	198	conserved hypothetical protein	SAUSA300_0956	
SAUSA300_1261	**1,6**	1,45E-16	Yes	744	731	693	1514	1503	1445	967	970	895	putative glutamyl aminopeptidase	SAUSA300_1261	
SAUSA300_1283	**1,6**	0,211555059	No	9	10	14	26	38	13	13	10	22	phosphate ABC transporter, phosphate-binding protein	pstS	
SAUSA300_0413	**1,6**	0,079378667	No	7	7	8	27	36	41	18	19	26	staphylococcal tandem lipoprotein	SAUSA300_0413	
SAUSA300_1330	**1,6**	0,283855081	No	21	30	69	43	52	44	21	38	24	threonine dehydratase	ilvA	
SAUSA300_2134	**1,6**	1,39E-11	Yes	520	620	524	878	886	871	546	565	562	htsC iron compound ABC transporter, permease protein	SAUSA300_2134	K02015
SAUSA300_0721	**1,6**	0,001083139	Yes	725	884	980	821	989	827	584	581	503	transferrin receptor	SAUSA300_0721	
SAUSA300_0072	**1,6**	9,24E-07	Yes	363	405	293	723	708	825	508	481	449	hypothetical protein	SAUSA300_0072	
SAUSA300_2406	**1,6**	0,00034205	Yes	491	546	442	692	694	711	441	434	454	putative transporter	SAUSA300_2406	
SAUSA300_0988	**1,6**	0,000806453	Yes	575	723	887	1875	1823	1749	1073	1284	1100	potassium uptake protein	trkA	
SAUSA300_2580	**1,6**	5,25E-07	Yes	1070	983	805	2469	2265	2481	1588	1599	1412	isochorismatase family protein	SAUSA300_2580	
SAUSA300_0041	**1,6**	0,069022458	No	40	25	53	63	72	55	40	28	50	conserved hypothetical protein	SAUSA300_0041	
SAUSA300_0484	**1,6**	3,46E-08	Yes	673	801	769	1273	1217	1341	834	804	808	conserved hypothetical protein	SAUSA300_0484	
SAUSA300_1315	**1,6**	9,24E-06	Yes	2833	3013	2312	3686	3176	3540	2410	2212	2007	PTS system, glucose-specific IIA component	crr	
SAUSA300_2643	**1,6**	8,38E-06	Yes	1946	2474	1779	4926	5013	5479	3375	3418	3029	putative chromosome partioning protein, ParB family	SAUSA300_2643	
SAUSA300_0187	**1,6**	0,003695872	Yes	350	501	293	450	451	495	298	305	283	ornithine--oxo-acid transaminase	rocD	
SAUSA300_2628	**1,6**	1,09E-08	Yes	661	698	643	1441	1382	1349	900	932	836	RarD protein	rarD	
SAUSA300_1973	**1,6**	0,000478303	Yes	131	174	112	243	203	237	150	148	140	truncated beta-hemolysin	SAUSA300_1973	
SAUSA300_1559	**1,6**	0,152860699	No	10	11	14	23	23	19	8	12	19	putative enterotoxin type A	SAUSA300_1559	
SAUSA300_1950	**1,6**	0,438143185	No	2	1	1	4	0	7	2	2	3	conserved hypothetical phage protein	SAUSA300_1950	
SAUSA300_1331	**1,6**	0,287065226	No	26	12	71	44	36	30	14	32	20	alanine dehydrogenase	ald	
SAUSA300_1783	**1,6**	4,78E-06	Yes	1047	1269	1110	2127	1864	2044	1289	1276	1300	uroporphyrinogen decarboxylase	hemE	
SAUSA300_2290	**1,6**	0,009928235	Yes	126	116	100	145	198	213	121	124	109	putative 3-methyladenine DNA glycosylase	SAUSA300_2290	
SAUSA300_2462	**1,5**	0,000650131	Yes	1653	1465	1084	4249	3205	4401	2752	2443	2422	NAD(P)H-flavin oxidoreductase	frp	
SAUSA300_2125	**1,5**	1,66E-05	Yes	2435	3113	2423	6503	6169	7639	4615	4705	3792	ATP-binding protein, Mrp/Nbp35 family	SAUSA300_2125	
SAUSA300_0813	**1,5**	0,028846888	No	88	82	107	371	372	334	209	250	229	conserved hypothetical protein	SAUSA300_0813	
SAUSA300_2516	**1,5**	0,00015495	Yes	133	142	127	278	248	265	181	163	168	oxidoreductase, short chain dehydrogenase/reductase f	SAUSA300_2516	
SAUSA300_0686	**1,5**	1,74E-10	Yes	859	1062	904	2324	2297	2543	1549	1574	1523	N-acetylglucosamine-6-phosphate deacetylase	nagA	
SAUSA300_0815	**1,5**	0,04345634	No	692	360	287	235	176	152	130	102	128	Ear protein	ear	
SAUSA300_2511	**1,5**	0,000600701	Yes	184	216	147	284	275	287	207	165	175	conserved hypothetical protein	SAUSA300_2511	
SAUSA300_0225	**1,5**	0,075932661	No	33	70	35	56	72	106	57	49	45	putative acyl-CoA acetyltransferase FadA	SAUSA300_0225	
SAUSA300_0454	**1,5**	6,55E-05	Yes	2358	2887	3263	4713	4675	5934	3337	3466	3147	recombination protein RecR	recR	
SAUSA300_1419	**1,5**	0,507444528	No	0	0	2	1	0	3	1	0	0	phiSLT ORF80-like protein	SAUSA300_1419	
SAUSA300_2146	**1,5**	4,43E-08	Yes	796	872	652	1476	1404	1533	1009	969	900	alcohol dehydrogenase, zinc-containing	SAUSA300_2146	
SAUSA300_1548	**1,5**	5,56E-06	Yes	813	850	723	1260	1041	1149	747	760	744	ComE operon protein 2	SAUSA300_1548	
SAUSA300_1706	**1,5**	4,22E-05	Yes	3080	2942	2380	3463	3726	3783	2489	2400	2255	conserved hypothetical protein	SAUSA300_1706	
SAUSA300_2425	**1,5**	0,01733901	No	72	131	170	221	176	197	147	111	127	conserved hypothetical protein	SAUSA300_2425	
SAUSA300_0468	**1,5**	2,66E-05	Yes	4097	5293	4101	5100	4814	5390	3595	3290	3094	hydrolase, TatD family	SAUSA300_0468	
SAUSA300_0151	**1,5**	0,184467614	No	293	329	391	339	430	410	212	341	197	alcohol dehydrogenase, iron-containing	adhE	
SAUSA300_0213	**1,5**	0,003954331	Yes	90	142	103	140	144	158	105	94	91	oxidoreductase, Gfo/Idh/MocA family	SAUSA300_0213	
SAUSA300_2148	**1,5**	0,000458623	Yes	238	318	417	838	791	914	562	572	539	conserved hypothetical protein	SAUSA300_2148	
SAUSA300_0771	**1,5**	0,036032758	No	233	327	212	306	270	383	236	205	187	acetyltransferase, GNAT family	SAUSA300_0771	
SAUSA300_1665	**1,5**	1,86E-05	Yes	734	749	616	901	992	889	646	594	590	conserved hypothetical protein	SAUSA300_1665	
SAUSA300_2278	**1,5**	0,061412365	No	74	64	37	112	65	92	54	59	64	urocanate hydratase	hutU	
SAUSA300_0492	**1,5**	2,21E-09	Yes	599	697	603	988	947	1004	667	674	606	dihydropteroate synthase	folP	
SAUSA300_0743	**1,5**	4,94E-09	Yes	1459	1614	1766	2973	2993	3306	1952	2043	2140	HPr(Ser) kinase/phosphatase	hprK	
SAUSA300_1463	**1,5**	5,70E-06	Yes	999	869	832	1507	1375	1752	1110	995	966	conserved hypothetical protein	SAUSA300_1463	
SAUSA300_2335	**1,5**	0,140880569	No	17	30	21	30	20	31	17	19	18	conserved hypothetical protein	SAUSA300_2335	
SAUSA300_0539	**1,5**	4,72E-09	Yes	4585	4970	3900	6399	6313	6542	4412	4320	4033	branched-chain amino acid aminotransferase	ilvE	
SAUSA300_0773	**1,5**	0,063746005	No	48	40	33	44	52	65	34	36	35	putative staphylocoagulase	SAUSA300_0773	
SAUSA300_1401	**1,5**	0,055799808	No	50	36	42	59	77	46	31	49	37	phiSLT ORF387-like protein, putative phage capsid prote	SAUSA300_1401	
SAUSA300_0614	**1,5**	0,507879786	No	2	1	4	4	7	3	6	1	1	putative Na+/H+ antiporter, MnhE component	SAUSA300_0614	
SAUSA300_1752	**1,5**	0,01830641	No	133	250	159	183	221	238	131	157	135	type I restriction-modification system, M subunit	hsdM	
SAUSA300_2612	**1,5**	0,125880151	No	34	29	28	43	25	41	19	26	26	ATP phosphoribosyltransferase hisG	hisG	
SAUSA300_1533	**1,5**	3,37E-07	Yes	9645	10511	9240	13739	14468	15422	9519	10248	9212	conserved hypothetical protein	SAUSA300_1533	

SAUSA300_1734	**1,5**	0,009780804	Yes	62	120	113	206	201	234	138	133	152	conserved hypothetical protein	SAUSA300_1734
SAUSA300_2210	**1,5**	7,27E-06	Yes	2416	3011	3067	6066	6018	5973	4123	4398	3477	probable glucose uptake protein	glcU
SAUSA300_0629	**1,5**	2,28E-05	Yes	2075	2116	2331	3169	3394	3366	2236	2160	2197	penicillin-binding protein 4	pbp4
SAUSA300_0214	**1,5**	0,002501592	Yes	148	198	167	218	248	243	161	165	143	conserved hypothetical protein	SAUSA300_0214
SAUSA300_0440	**1,5**	3,46E-05	Yes	320	356	275	404	421	379	289	259	252	MutT/nudix family protein	SAUSA300_0440
SAUSA300_0382	**1,5**	0,002792439	Yes	3369	3607	2181	1860	1535	2035	1300	1132	1181	sodium:dicarboxylate symporter family protein	SAUSA300_0382
SAUSA300_0615	**1,5**	0,185237832	No	7	19	16	26	14	30	16	16	14	putative Na+/H+ antiporter, MnhF component	SAUSA300_0615
SAUSA300_1263	**1,5**	0,058932644	No	22	29	29	54	63	62	37	38	42	anthranilate synthase, glutamine amidotransferase, con	trpG
SAUSA300_0728	**1,5**	8,88E-06	Yes	372	423	326	579	631	519	390	391	370	conserved hypothetical protein	SAUSA300_0728
SAUSA300_2494	**1,5**	8,73E-06	Yes	2702	2870	2024	8847	8247	8844	6094	5504	5727	copper-translocating P-type ATPase	SAUSA300_2494
SAUSA300_2140	**1,5**	2,16E-08	Yes	822	972	854	1257	1388	1380	842	877	970	sfaD siderophore A , modsat orientering	SAUSA300_2140
SAUSA300_0040	**1,5**	0,183433177	No	24	18	15	24	41	22	21	17	18	conserved hypothetical protein	SAUSA300_0040
SAUSA300_1725	**1,5**	0,001846431	Yes	2844	2567	1964	4086	3798	3959	3014	2413	2484	transaldolase	SAUSA300_1725
SAUSA300_2089	**1,5**	1,32E-06	Yes	513	580	641	1585	1557	1671	1038	1171	1019	pyrimidine nucleoside phosphorylase	pdp
SAUSA300_1447	**1,5**	6,96E-05	Yes	1378	1437	1208	2195	1902	1973	1381	1253	1434	tyrosine recombinase XerD	xerD
SAUSA300_0543	**1,5**	2,71E-05	Yes	296	290	259	248	248	291	190	171	170	putative deaminase	SAUSA300_0543
SAUSA300_2631	**1,5**	0,042001947	No	172	162	138	485	469	526	378	304	304	putative N-acetyltransferase	SAUSA300_2631
SAUSA300_0342	**1,5**	0,059395573	No	38	41	41	57	56	55	49	35	28	conserved hypothetical protein	SAUSA300_0342
SAUSA300_2090	**1,5**	0,000377702	Yes	127	179	169	418	453	534	311	335	298	deoxyribose-phosphate aldolase	deoC
SAUSA300_2249	**1,5**	0,002284804	Yes	9313	10643	7102	30532	38977	35875	22647	23727	24519	secretory antigen precursor SsaA	ssaA
SAUSA300_0951	**1,5**	0,00122761	Yes	796	756	597	481	385	424	305	306	263	V8 protease	sspA
SAUSA300_1105	**1,5**	1,74E-09	Yes	2509	3048	2568	4544	4517	4691	3157	3196	2942	primosomal protein N`	priA
SAUSA300_0469	**1,5**	0,000265756	Yes	591	906	723	852	906	951	660	611	556	primase-related protein	SAUSA300_0469
SAUSA300_0811	**1,5**	0,19340077	No	33	16	14	109	124	117	62	73	95	conserved hypothetical protein	SAUSA300_0811
SAUSA300_2435	**1,5**	0,004911481	Yes	1440	2230	2045	1224	1075	1522	858	920	807	cell wall surface anchor family protein	SAUSA300_2435
SAUSA300_1737	**1,5**	0,000327591	Yes	512	734	643	980	829	994	629	647	627	O-succinylbenzoate-CoA ligase	menE
SAUSA300_0026	**1,5**	0,018513765	No	439	623	366	428	363	531	294	305	296	conserved hypothetical protein OrfX	SAUSA300_0026
SAUSA300_0185	**1,5**	0,013423826	No	663	847	552	669	714	744	525	491	421	arginine biosynthesis bifunctional protein ArgJ	argJ
SAUSA300_0846	**1,5**	0,039748461	No	1262	739	820	412	336	359	257	233	257	Na+/H+ antiporter family protein	SAUSA300_0846
SAUSA300_0441	**1,5**	0,000381857	Yes	811	901	679	886	868	901	602	660	542	acetyltransferase, GNAT family	SAUSA300_0441
SAUSA300_1055	**1,5**	0,025131841	No	2864	1592	1566	1710	1963	1881	1229	1309	1213	fibrinogen-binding protein	efb
SAUSA300_0745	**1,5**	4,09E-09	Yes	558	615	578	1038	994	1134	718	747	699	putative acetyltransferase	SAUSA300_0745
SAUSA300_0170	**1,5**	0,008508283	Yes	119	126	130	440	347	396	258	293	254	aldehyde dehydrogenase	SAUSA300_0170
SAUSA300_2367	**1,5**	0,004340654	Yes	115	85	89	112	126	111	68	85	82	gamma-hemolysin component B	hlgB
SAUSA300_2574	**1,5**	0,000865789	Yes	691	741	560	997	1086	1130	699	789	700	conserved hypothetical protein	SAUSA300_2574
SAUSA300_1061	**1,5**	0,266192272	No	5	10	7	17	16	19	8	9	17	putative exotoxin 3	SAUSA300_1061
SAUSA300_0651	**1,5**	0,001641104	Yes	13419	10692	8614	9257	10735	10808	7503	6837	6640	CHAP domain family	SAUSA300_0651
SAUSA300_2067	**1,5**	6,75E-05	Yes	5437	7486	7146	10102	10372	12151	7472	7919	6943	serine hydroxymethyltransferase	glyA
SAUSA300_1716	**1,5**	0,002118406	Yes	122	175	183	233	214	268	163	160	169	conserved hypothetical protein	SAUSA300_1716
SAUSA300_2346	**1,5**	0,347960127	No	60	57	162	125	149	201	84	142	87	nitrite reductase [NAD(P)H	nirB
SAUSA300_2342	**1,5**	0,123380158	No	29	40	40	63	36	61	39	45	27	respiratory nitrate reductase, beta subunit	narH
SAUSA300_2277	**1,5**	0,011585435	No	215	177	147	205	210	250	152	155	148	imidazolonepropionase	hutI
SAUSA300_0803	**1,5**	0,000600701	Yes	847	903	686	1527	1560	1238	1020	994	954	transcriptional regulator, Cro/CI family	SAUSA300_0803
SAUSA300_1847	**1,5**	3,27E-07	Yes	1121	991	1084	2158	2382	2174	1590	1568	1460	conserved hypothetical protein	SAUSA300_1847
SAUSA300_2487	**1,4**	0,001316399	Yes	96	94	107	424	325	418	256	274	276	ferrous iron transport protein B	feoB
SAUSA300_2506	**1,4**	0,001619392	Yes	48989	43113	35762	81423	96654	105774	68819	61169	65053	immunodominant staphylococcal antigen A precursor	isaA
SAUSA300_1248	**1,4**	0,000595952	Yes	574	563	676	1011	924	1020	719	684	632	conserved hypothetical protein	SAUSA300_1248
SAUSA300_2002	**1,4**	0,002828778	Yes	1052	1131	1224	2114	2267	2470	1619	1505	1582	glycoprotein endopeptidase	SAUSA300_2002
SAUSA300_2087	**1,4**	0,078635781	No	468	999	1155	1001	1098	1485	774	869	801	putative peptidase	SAUSA300_2087
SAUSA300_1494	**1,4**	0,000160275	Yes	1464	1666	1308	1756	1612	1777	1173	1230	1143	conserved hypothetical protein	SAUSA300_1494
SAUSA300_1845	**1,4**	7,45E-10	Yes	1383	1508	1394	2410	2416	2625	1668	1819	1656	glutamate-1-semialdehyde-2,1-aminomutase	hemL
SAUSA300_1921	**1,4**	0,000161767	Yes	1740	1493	1433	2427	2421	2366	1655	1804	1514	truncated amidase	SAUSA300_1921
SAUSA300_0092	**1,4**	0,245987658	No	45	30	22	36	25	12	16	16	17	conserved hypothetical protein	SAUSA300_0092
SAUSA300_0941	**1,4**	0,011115126	No	191	214	166	223	192	254	166	143	155	putative ferrichrome ABC transporter	SAUSA300_0941
SAUSA300_2377	**1,4**	0,000331631	Yes	634	817	718	1273	1208	1482	948	980	815	glycerate kinase	SAUSA300_2377
SAUSA300_0715	**1,4**	0,002865227	Yes	2313	3020	2923	3171	3631	3630	2453	2558	2195	nrdI protein	nrdI
SAUSA300_0802	**1,4**	5,02E-05	Yes	1242	1332	946	2154	2067	1822	1423	1421	1341	conserved hypothetical protein	SAUSA300_0802
SAUSA300_0061	**1,4**	0,00037591	Yes	372	393	308	584	591	683	472	407	413	carbamate kinase	arcC
SAUSA300_0760	**1,4**	3,62E-05	Yes	43212	45552	38251	64809	68476	68200	48112	50678	41182	phosphopyruvate hydratase	eno

SAUSA300_0540	**1,4**	0,006259956	Yes	1357	1691	1026	1648	1497	1769	1262	1094	1055	HAD-superfamily hydrolase, subfamily IA, variant 1	SAUSA300_0540
SAUSA300_0489	**1,4**	6,81E-09	Yes	23358	24630	25398	54484	58079	61444	39770	41366	39992	putative cell division protein FtsH	SAUSA300_0489
SAUSA300_0896	**1,4**	0,06190373	No	34	56	32	67	74	77	49	61	42	oligopeptide ABC transporter, permease protein	oppC
SAUSA300_1702	**1,4**	9,64E-05	Yes	663	730	575	871	976	920	590	694	643	cell wall surface anchor family protein	SAUSA300_1702
SAUSA300_0788	**1,4**	0,003826336	Yes	1095	1181	953	1552	1251	1698	992	974	1173	nitroreductase family protein	SAUSA300_0788
SAUSA300_0083	**1,4**	0,001434889	Yes	224	246	187	236	255	271	183	179	169	putative membrane protein	SAUSA300_0083
SAUSA300_2222	**1,4**	0,005464658	Yes	269	457	417	869	875	858	680	578	557	molybdopterin converting factor, subunit 2	moaE
SAUSA300_0226	**1,4**	0,088895388	No	29	53	43	59	90	71	52	54	45	3-hydroxyacyl-CoA dehydrogenase	SAUSA300_0226
SAUSA300_0477	**1,4**	0,002968715	Yes	2470	3864	3147	5234	4932	6468	3831	4153	3640	UDP-N-acetylglucosamine pyrophosphorylase	glmU
SAUSA300_1865	**1,4**	1,55E-07	Yes	2079	2060	1779	3271	3205	3141	2347	2272	2123	DNA-binding response regulator	vraR
SAUSA300_2068	**1,4**	0,000533696	Yes	1748	2532	2444	3635	3888	4303	2762	2917	2616	conserved hypothetical protein	SAUSA300_2068
SAUSA300_2641	**1,4**	0,122657327	No	31	25	48	42	56	52	32	35	36	conserved hypothetical protein	SAUSA300_2641
SAUSA300_0589	**1,4**	2,10E-06	Yes	808	923	856	1222	1210	1230	875	860	840	aldo/keto reductase family protein	SAUSA300_0589
SAUSA300_0244	**1,4**	0,016437287	No	157	177	128	166	180	240	150	137	125	oxidoreductase, zinc-binding dehydrogenase family	SAUSA300_0244
SAUSA300_2533	**1,4**	0,014206931	No	1731	2931	2157	3245	3176	3861	2459	2353	2405	pantoate--beta-alanine ligase	panC
SAUSA300_1423	**1,4**	0,441159673	No	2	10	9	6	9	12	6	5	7	phage related DNA polymerase, family A	polA
SAUSA300_1901	**1,4**	1,86E-05	Yes	3005	2950	2395	5504	4932	4976	3755	3587	3518	aldehyde dehydrogenase	aldA2
SAUSA300_1325	**1,4**	0,077378948	No	67	52	47	53	56	58	40	48	30	conserved hypothetical protein	SAUSA300_1325
SAUSA300_0514	**1,4**	0,001265399	Yes	420	595	602	839	791	849	569	642	537	serine acetyltransferase	cysE
SAUSA300_1080	**1,4**	1,15E-07	Yes	27533	30446	29512	40086	41147	45229	29108	30766	29309	cell division protein ftsZ	ftsZ
SAUSA300_1281	**1,4**	0,099125745	No	36	48	42	50	41	59	27	42	37	phosphate ABC transporter, permease protein PstA	pstA
SAUSA300_1424	**1,4**	0,47848042	No	7	10	6	7	5	7	5	4	4	conserved hypothetical phage protein	SAUSA300_1424
SAUSA300_2102	**1,4**	2,38E-05	Yes	746	791	702	1800	1589	1730	1203	1280	1137	haloacid dehalogenase-like hydrolase	SAUSA300_2102
SAUSA300_2028	**1,4**	0,000375714	Yes	770	753	652	855	922	915	660	647	593	holo-(acyl-carrier-protein) synthase	acpS
SAUSA300_1613	**1,4**	0,022322702	No	351	341	419	381	363	319	244	225	278	putative abrB protein	SAUSA300_1613
SAUSA300_1179	**1,4**	0,000117005	Yes	26528	25513	20965	28063	27495	29857	20802	20634	18952	conserved hypothetical protein	SAUSA300_1179
SAUSA300_0780	**1,4**	7,64E-05	Yes	248	211	198	422	464	452	319	312	313	conserved hypothetical protein	SAUSA300_0780
SAUSA300_1301	**1,4**	3,30E-06	Yes	2330	2489	2206	2665	2991	2799	1989	1915	2073	conserved hypothetical protein	SAUSA300_1301
SAUSA300_1433	**1,4**	0,590049386	No	0	1	0	3	0	0	1	1	0	putative phage regulatory protein	SAUSA300_1433
SAUSA300_2286	**1,4**	0,070045602	No	171	311	352	369	327	379	239	290	228	conserved hypothetical protein	SAUSA300_2286
SAUSA300_0212	**1,4**	0,04935168	No	67	112	123	119	131	174	97	98	104	oxidoreductase, Gfo/Idh/MocA family	SAUSA300_0212
SAUSA300_0695	**1,4**	0,001560948	Yes	973	953	898	822	1111	940	631	758	639	radical activating enzyme family protein	SAUSA300_0695
SAUSA300_0012	**1,4**	0,013703191	No	589	753	497	670	604	682	524	448	413	putative homoserine O-acetyltransferase	SAUSA300_0012
SAUSA300_1905	**1,4**	0,005349096	Yes	765	1032	904	1030	1082	1031	716	788	719	conserved hypothetical protein	SAUSA300_1905
SAUSA300_1755	**1,4**	0,481629132	No	5	14	6	3	14	9	6	5	5	serine protease SplD	splD
SAUSA300_1786	**1,4**	7,66E-07	Yes	1796	1693	1632	3895	3877	3664	2850	2782	2497	ABC transporter, ATP-binding protein EcsA	SAUSA300_1786
SAUSA300_2519	**1,4**	2,33E-05	Yes	183	179	177	378	381	430	292	275	280	putative cobalamin synthesis protein	SAUSA300_2519
SAUSA300_0248	**1,4**	0,00021428	Yes	1932	1517	1499	4018	4781	4053	3280	3000	2852	putative teichoic acid biosynthesis protein F	SAUSA300_0248
SAUSA300_1486	**1,4**	0,412910339	No	7	5	7	11	16	6	6	6	9	conserved hypothetical protein	SAUSA300_1486
SAUSA300_0064	**1,4**	0,004011022	Yes	107	111	112	228	230	240	178	150	167	arginine/oirnithine antiporter	arcD
SAUSA300_0144	**1,4**	0,096041817	No	115	96	65	62	61	61	44	49	38	phosphonate ABC transporter, ATP-binding protein	phnC
SAUSA300_1712	**1,4**	0,515445769	No	205	222	222	301	282	319	211	189	194	riboflavin synthase, beta subunit	ribH
SAUSA300_0106	**1,4**	0,013423826	No	453	602	485	560	548	648	479	388	386	putative drug transporter	SAUSA300_0106
SAUSA300_1193	**1,4**	0,017821109	No	243	389	350	438	347	452	337	272	277	glycerol-3-phosphate dehydrogenase	glpD
SAUSA300_1418	**1,4**	0,594805145	No	0	0	1	1	2	0	0	1	0	phiSLT ORF 82-like protein	SAUSA300_1418
SAUSA300_1679	**1,4**	0,035473241	No	136	119	124	183	171	149	108	139	113	acetyl-coenzyme A synthetase	acsA
SAUSA300_1876	**1,4**	0,000130353	Yes	265	250	215	557	467	621	416	397	375	DNA polymerase IV	SAUSA300_1876
SAUSA300_1491	**1,4**	0,001547569	Yes	3254	3652	2979	3944	3548	3726	2825	2541	2676	proline dipeptidase	SAUSA300_1491
SAUSA300_2128	**1,4**	6,96E-07	Yes	501	467	452	623	665	584	453	445	441	putative drug transporter	SAUSA300_2128
SAUSA300_0099	**1,4**	0,103469221	No	169	209	228	80	74	117	70	59	65	1-phosphatidylinositol phosphodiesterase	plc
SAUSA300_1597	**1,4**	0,00254378	Yes	1288	1774	1750	2203	2416	2684	1635	1843	1759	holliday junction DNA helicase RuvB	ruvB
SAUSA300_2552	**1,4**	0,039166793	No	193	194	137	185	151	210	130	134	129	citrate transporter, permease protein	SAUSA300_2552
SAUSA300_0551	**1,4**	0,000293973	Yes	4041	4020	3166	4696	4341	4717	3600	3198	3110	conserved hypothetical protein	SAUSA300_0551
SAUSA300_1674	**1,4**	0,001489918	Yes	3874	4777	3874	8424	7846	8367	5974	5850	5916	putative serine protease HtrA	SAUSA300_1674
SAUSA300_2477	**1,4**	0,018294402	No	8193	6837	5844	7710	7618	7528	5754	6250	4421	pyruvate oxidase	cidC
SAUSA300_0517	**1,4**	0,000892628	Yes	713	847	901	1276	1406	1529	993	1070	970	RNA methyltransferase, TrmH family, group 3	SAUSA300_0517
SAUSA300_1904	**1,4**	0,247855183	No	60	60	85	62	126	70	60	48	71	conserved hypothetical protein	SAUSA300_1904
SAUSA300_0943	**1,4**	0,000776623	Yes	381	413	428	738	753	699	493	573	515	acetyltransferase, GNAT family family	SAUSA300_0943
SAUSA300_2523	**1,4**	0,008508283	Yes	141	168	159	967	760	1091	650	693	696	conserved hypothetical protein	SAUSA300_2523

SAUSA300_0260	**1,4**	0,03269595	No	214	352	273	342	327	442	259	274	270	6-phospho-beta-glucosidase	bglA
SAUSA300_1239	**1,4**	4,97E-06	Yes	21416	21600	17633	31088	31685	32070	23894	24057	20739	transketolase	tkt
SAUSA300_0465	**1,4**	0,002974046	Yes	131	141	112	255	192	243	173	161	170	conserved hypothetical protein	SAUSA300_0465
SAUSA300_0634	**1,4**	0,005837763	Yes	481	580	620	722	775	969	575	595	614	ferrichrome transport permease protein fhuB	fhuB
SAUSA300_1265	**1,4**	0,451385035	No	9	16	20	11	16	16	4	12	13	indole-3-glycerol phosphate synthase	trpC
SAUSA300_1693	**1,4**	0,000515197	Yes	512	537	512	1161	992	1050	797	860	670	conserved hypothetical protein	SAUSA300_1693
SAUSA300_0483	**1,4**	2,94E-06	Yes	2615	3276	2763	4270	4210	4367	3180	3149	2994	tetrapyrrole methylase family protein	SAUSA300_0483
SAUSA300_1128	**1,4**	0,05881107	No	455	823	999	1382	1519	1770	1128	1078	1166	signal recognition particle-docking protein FtsY	ftsY
SAUSA300_1156	**1,4**	3,57E-06	Yes	5338	5907	5822	7656	8114	7644	5592	5978	5425	prolyl-tRNA synthetase	proS
SAUSA300_1020	**1,4**	4,05E-05	Yes	1531	1708	1329	3211	3365	3534	2469	2595	2275	Glycerophosphoryl diester phosphodiesterase family pr	SAUSA300_1020
SAUSA300_1999	**1,4**	0,000410221	Yes	4435	4309	4026	4975	4753	4830	3708	3687	3196	redox-sensing transcriptional repressor rex	rex
SAUSA300_2223	**1,4**	0,012230547	No	179	267	305	434	403	503	320	294	361	molybdopterin-guanine dinucleotide biosynthesis prote	mobB
SAUSA300_1071	**1,4**	0,000846386	Yes	1102	1287	1231	2412	2236	2299	1754	1577	1724	conserved hypothetical protein	SAUSA300_1071
SAUSA300_1321	**1,4**	0,000376713	Yes	1123	1240	930	1133	985	1241	874	798	782	conserved hypothetical protein	SAUSA300_1321
SAUSA300_2491	**1,4**	0,001705915	Yes	589	678	565	1700	1397	1655	1203	1215	1049	1-pyrroline-5-carboxylate dehydrogenase	SAUSA300_2491
SAUSA300_0349	**1,4**	0,010100085	No	1786	1340	1304	2788	3038	2396	2325	1959	1709	conserved hypothetical protein	SAUSA300_0349
SAUSA300_0739	**1,4**	0,002872498	Yes	227	245	194	258	291	284	202	188	214	LysM domain protein	SAUSA300_0739
SAUSA300_1487	**1,4**	0,161254458	No	40	37	30	59	74	49	42	45	42	replication initiation factor family protein	SAUSA300_1487
SAUSA300_2341	**1,4**	0,514907786	No	3	8	7	14	5	7	8	6	5	respiratory nitrate reductase, delta subunit	narJ
SAUSA300_2492	**1,4**	0,005645098	Yes	267	348	275	889	775	940	664	605	636	acetyltransferase family protein	SAUSA300_2492
SAUSA300_1507	**1,4**	0,01742861	No	1166	1799	1766	1802	1963	2304	1392	1447	1593	glucokinase	glk
SAUSA300_0216	**1,4**	0,327451425	No	45	56	51	37	59	59	46	31	35	hexose phosphate transport protein	uhpT
SAUSA300_2066	**1,4**	0,001843471	Yes	2304	2896	2892	3383	3516	3885	2749	2605	2544	uracil phosphoribosyltransferase	upp
SAUSA300_0405	**1,4**	0,005837763	Yes	1702	2457	2136	1846	1717	2091	1387	1457	1302	type I restriction-modification system, M subunit	hsdM
SAUSA300_2267	**1,4**	1,99E-05	Yes	797	980	886	1207	1181	1176	802	907	905	hydrolase, haloacid dehalogenase-like family	SAUSA300_2267
SAUSA300_2623	**1,4**	0,016229457	No	372	434	371	527	575	540	379	453	368	pyrrolidone-carboxylate peptidase	pcp
SAUSA300_0682	**1,4**	0,018982629	No	107	130	139	183	149	186	122	136	125	ybaK/ebsC protein	ybaK
SAUSA300_0142	**1,4**	0,055315322	No	88	99	83	90	74	78	56	65	58	phosphonate ABC transporter, permease protein	phnE
SAUSA300_1480	**1,4**	0,249761383	No	31	34	25	29	29	40	26	23	23	putative traG membrane protein	SAUSA300_1480
SAUSA300_1532	**1,4**	0,0121551	No	10511	11304	10359	13844	14853	15576	10759	10982	10731	conserved hypothetical protein	SAUSA300_1532
SAUSA300_1304	**1,4**	0,000257806	Yes	2091	2259	1929	2831	2558	2777	2074	2033	1902	conserved hypothetical protein	SAUSA300_1304
SAUSA300_1920	**1,4**	0,476597835	No	160	71	83	14	27	9	9	9	16	chemotaxis-inhibiting protein CHIPS	chs
SAUSA300_1243	**1,4**	1,92E-05	Yes	782	850	877	1492	1440	1424	1089	981	1138	exonuclease SbcC	sbcC
SAUSA300_0576	**1,4**	0,14548382	No	1428	1474	790	1396	1046	1229	1092	773	820	putative Pyridine nucleotide-disulphide oxidoreductase	SAUSA300_0576
SAUSA300_2088	**1,4**	0,005683434	Yes	2384	2181	1871	3255	2955	3447	2564	2450	2097	S-ribosylhomocysteinase	luxS
SAUSA300_0016	**1,4**	0,001943216	Yes	8619	9223	7457	14566	15453	16547	12010	12211	10102	replicative DNA helicase	dnaB
SAUSA300_0024	**1,4**	0,001410224	Yes	1359	1087	1117	2396	2364	2506	1868	1828	1663	metallo-beta-lactamase family protein	SAUSA300_0024
SAUSA300_0616	**1,4**	0,41914007	No	16	19	20	14	29	24	18	15	15	putative Na+/H+ antiporter, MnhG component	SAUSA300_0616
SAUSA300_0470	**1,4**	0,000378269	Yes	983	1220	1318	1362	1298	1272	1010	972	924	dimethyladenosine transferase	ksgA
SAUSA300_2126	**1,4**	0,000185979	Yes	940	873	776	1123	1100	1032	860	818	729	drug resistance transporter, EmrB/QacA subfamily	SAUSA300_2126
SAUSA300_0612	**1,3**	0,615441509	No	0	1	1	6	5	1	2	4	2	putative Na+/H+ antiporter, MnhC component	SAUSA300_0612
SAUSA300_0875	**1,3**	0,000437517	Yes	1717	1692	1615	3194	3541	3503	2640	2641	2296	conserved hypothetical protein	SAUSA300_0875
SAUSA300_0264	**1,3**	0,021711189	No	198	189	165	364	293	370	221	319	223	ribose transporter RbsU	SAUSA300_0264
SAUSA300_2525	**1,3**	0,09321747	No	377	480	359	398	295	509	331	298	262	conserved hypothetical protein	SAUSA300_2525
SAUSA300_2497	**1,3**	0,035297972	No	615	968	669	861	798	972	598	758	590	aminotransferase, class I	SAUSA300_2497
SAUSA300_2561	**1,3**	0,186770945	No	28	49	35	67	92	87	63	72	45	alkaline phosphatase	phoB
SAUSA300_1640	**1,3**	1,21E-06	Yes	813	854	826	3069	3110	3230	2352	2436	2211	isocitrate dehydrogenase, NADP-dependent	icd
SAUSA300_2590	**1,3**	0,017652838	No	966	894	617	740	766	853	628	579	545	conserved hypothetical protein	SAUSA300_2590
SAUSA300_1341	**1,3**	1,02E-07	Yes	22454	22390	21402	33253	36567	36425	25679	27412	25940	penicillin binding protein 2	pbp2
SAUSA300_2507	**1,3**	0,098575109	No	91	78	71	74	86	86	64	57	60	regulatory protein-like protein	SAUSA300_2507
SAUSA300_0452	**1,3**	0,005640731	Yes	1762	2141	2267	3027	2673	3556	2261	2359	2261	DNA polymerase III, gamma and tau subunits	dnaX
SAUSA300_0568	**1,3**	0,00231033	Yes	939	1125	862	656	701	753	542	533	494	integral membrane protein	SAUSA300_0568
SAUSA300_0320	**1,3**	0,002508805	Yes	12280	12686	9945	2904	2833	3417	2306	2005	2506	triacylglycerol lipase precursor	SAUSA300_0320
SAUSA300_2482	**1,3**	0,107044601	No	1280	1181	644	1588	2092	1980	1372	1241	1580	conserved hypothetical protein	SAUSA300_2482
SAUSA300_2535	**1,3**	7,73E-05	Yes	1576	1747	1447	3265	3261	3480	2504	2616	2346	2-dehydropantoate 2-reductase	panE
SAUSA300_1351	**1,3**	0,001999445	Yes	19769	18778	16775	33396	34379	36849	28065	28102	21832	conserved hypothetical protein	SAUSA300_1351
SAUSA300_2536	**1,3**	0,023661149	No	746	1016	793	1002	949	933	743	794	612	alpha-acetolactate decarboxylase	budA
SAUSA300_0655	**1,3**	8,31E-07	Yes	2449	2606	2608	3394	3415	3750	2613	2767	2512	conserved hypothetical protein	SAUSA300_0655
SAUSA300_1050	**1,3**	2,31E-05	Yes	2726	2397	2189	4150	3904	3478	2980	2769	2870	non-canonical purine NTP pyrophosphatase, rdgB/HAM	SAUSA300_1050

SAUSA300_2513	**1,3**	0,028406522	No	62	133	103	200	180	210	153	146	144	conserved hypothetical protein	SAUSA300_2513
SAUSA300_1348	**1,3**	2,69E-05	Yes	734	892	777	1588	1704	1581	1161	1221	1260	polyA polymerase	SAUSA300_1348
SAUSA300_1749	**1,3**	0,620863491	No	10	10	12	9	7	10	10	2	5	conserved hypothetical protein	SAUSA300_1749
SAUSA300_0246	**1,3**	0,046441397	No	1314	1842	1518	1133	969	1158	826	844	768	putative alcohol dehydrogenase	SAUSA300_0246
SAUSA300_1458	**1,3**	0,021241176	No	2472	1764	1966	2350	2603	2489	1994	1750	1820	glyoxalase family protein	SAUSA300_1458
SAUSA300_0073	**1,3**	0,003905614	Yes	646	843	628	1299	1388	1335	1039	1012	962	peptide ABC transporter, peptide-binding protein	SAUSA300_0073
SAUSA300_2510	**1,3**	0,038752876	No	141	127	127	198	167	191	140	134	143	conserved hypothetical protein	SAUSA300_2510
SAUSA300_2320	**1,3**	0,003811595	Yes	277	246	264	285	257	327	216	217	224	conserved hypothetical protein	SAUSA300_2320
SAUSA300_2645	**1,3**	0,000256273	Yes	1412	1729	1580	3098	3424	3314	2453	2510	2439	glucose-inhibited division protein A	gidA
SAUSA300_0075	**1,3**	0,001443183	Yes	417	387	361	649	769	728	560	551	500	oligopeptide permease, channel-forming protein	opp-3C
SAUSA300_2478	**1,3**	0,032102695	No	1900	1744	1456	1824	1938	1955	1433	1742	1123	Holin-like protein cidB	cidB
SAUSA300_1451	**1,3**	6,71E-05	Yes	432	534	460	696	683	683	503	498	551	oxidoreductase, short-chain dehydrogenase/reductase f	SAUSA300_1451
SAUSA300_2239	**1,3**	0,024887228	No	107	107	120	3560	3142	3641	2512	2716	2550	urease, beta subunit	ureB
SAUSA300_2483	**1,3**	0,000359017	Yes	1526	1489	1383	2419	2168	2637	1764	1996	1691	hydroxymethylglutaryl-CoA reductase	SAUSA300_2483
SAUSA300_2272	**1,3**	0,018694901	No	1218	1578	1306	1560	1436	1825	1332	1176	1126	conserved hypothetical protein	SAUSA300_2272
SAUSA300_1705	**1,3**	0,005526733	Yes	622	530	557	760	721	685	571	557	505	putative drug transporter	SAUSA300_1705
SAUSA300_1039	**1,3**	0,000631724	Yes	427	478	434	729	739	754	549	576	552	ribonuclease HIII	rnhC
SAUSA300_0430	**1,3**	1,37E-05	Yes	1431	1510	1323	1989	1887	1936	1496	1547	1349	conserved hypothetical protein	SAUSA300_0430
SAUSA300_1769	**1,3**	0,369471181	No	29	47	32	20	32	18	16	18	16	leukotoxin LukE	lukE
SAUSA300_0726	**1,3**	1,98E-05	Yes	1264	1488	1265	2172	2216	2350	1684	1652	1753	glycerate kinase family protein	SAUSA300_0726
SAUSA300_0504	**1,3**	0,06683575	No	1686	2743	2477	2502	2443	3012	2355	2006	1631	pyridoxine biosynthesis protein	SAUSA300_0504
SAUSA300_2312	**1,3**	0,011689459	No	644	998	909	1862	1893	2308	1464	1719	1395	malate:quinone-oxidoreductase	mqo
SAUSA300_1175	**1,3**	0,005740365	Yes	1819	1977	1792	1975	1983	1956	1524	1458	1484	conserved hypothetical protein	SAUSA300_1175
SAUSA300_1104	**1,3**	0,00021428	Yes	916	1094	971	1385	1330	1430	1097	1047	993	phosphopantothenoylcysteine decarboxylase/phosphop	coaBC
SAUSA300_0007	**1,3**	0,095929803	No	605	560	520	845	965	868	701	733	581	conserved hypothetical protein	SAUSA300_0007
SAUSA300_0688	**1,3**	0,015403835	No	1414	1376	1069	1926	1772	1715	1427	1312	1351	oxidoreductase, aldo/keto reductase family	SAUSA300_0688
SAUSA300_0339	**1,3**	0,015800076	No	236	287	236	308	239	333	231	217	223	conserved hypothetical protein	SAUSA300_0339
SAUSA300_1151	**1,3**	0,002595544	Yes	3291	4317	4372	4418	4334	4748	3497	3264	3451	uridylate kinase	pyrH
SAUSA300_2229	**1,3**	5,66E-05	Yes	806	968	784	904	856	929	670	707	664	molybdenum ABC transporter, permease protein ModB	modB
SAUSA300_1806	**1,3**	0,008508283	Yes	935	1162	935	865	791	988	667	713	625	putative iron-sulfur cluster-binding protein	SAUSA300_1806
SAUSA300_2496	**1,3**	0,013060446	No	734	977	700	891	843	983	683	753	623	D-isomer specific 2-hydroxyacid dehydrogenase family p	SAUSA300_2496
SAUSA300_2109	**1,3**	0,012817094	No	1323	1421	876	1442	1456	1587	1171	1104	1120	truncated FmtB protein	fmtB
SAUSA300_2464	**1,3**	0,056914964	No	107	118	85	189	176	169	133	145	125	hydrolase, haloacid dehalogenase-like family	SAUSA300_2464
SAUSA300_0131	**1,3**	0,152278939	No	55	73	71	105	95	93	48	87	85	putative Bacterial sugar transferase	SAUSA300_0131
SAUSA300_1426	**1,3**	0,676947768	No	0	1	0	0	2	1	1	1	0	conserved hypothetical phage protein	SAUSA300_1426
SAUSA300_2441	**1,3**	0,183044972	No	1416	1274	1409	5065	6557	6617	3916	6074	3767	fibronectin binding protein A	fnbA
SAUSA300_0228	**1,3**	0,341000699	No	34	29	18	39	59	37	35	39	26	acyl-CoA synthetase FadE	fadE
SAUSA300_1615	**1,3**	0,00314569	Yes	2229	2265	1665	2447	2141	2354	1811	1731	1734	delta-aminolevulinic acid dehydratase	hemB
SAUSA300_1343	**1,3**	0,018982629	No	1064	1055	754	1445	1282	1346	1129	949	1015	endonuclease III	nth
SAUSA300_0562	**1,3**	0,014094745	No	2160	2446	2370	3865	3816	4410	3130	3291	2765	phosphomethylpyrimidine kinase	thiD
SAUSA300_0230	**1,3**	0,071839181	No	920	1445	1205	1021	1010	1153	779	806	830	putative membrane protein	SAUSA300_0230
SAUSA300_0555	**1,3**	0,007574961	Yes	4420	4432	3765	6227	6126	5930	4923	4869	4101	putative hexulose-6-phosphate synthase	SAUSA300_0555
SAUSA300_1382	**1,3**	0,142413004	No	179	85	119	170	151	163	118	111	138	Panton-Valentine leukocidin, LukS-PV	lukS-PV
SAUSA300_1390	**1,3**	0,404682951	No	12	12	21	21	18	16	17	13	12	phiSLT ORF96-like protein	SAUSA300_1390
SAUSA300_2078	**1,3**	5,77E-05	Yes	2298	2680	2514	5125	5247	5832	4226	3951	4163	UDP-N-acetylglucosamine 1-carboxyvinyltransferase	murA
SAUSA300_0318	**1,3**	0,15212013	No	281	275	252	295	307	358	229	255	243	N-acetylmannosamine-6-phosphate 2-epimerase	SAUSA300_0318
SAUSA300_1357	**1,3**	0,007361371	Yes	1078	1332	1143	892	913	1019	726	714	712	chorismate synthase	aroC
SAUSA300_2234	**1,3**	0,062084889	No	165	259	224	259	257	313	211	215	206	Inosine-uridine preferring nucleoside hydrolase	SAUSA300_2234
SAUSA300_0744	**1,3**	0,002638771	Yes	582	798	795	1315	1199	1431	968	1053	991	prolipoprotein diacylglyceryl transferase	lgt
SAUSA300_0485	**1,3**	0,006866407	Yes	1016	1244	1000	1627	1690	1591	1312	1295	1132	cell-division initiation protein	SAUSA300_0485
SAUSA300_0348	**1,3**	0,04149272	No	706	523	552	904	956	772	706	671	623	twin-arginine translocation protein, TatA/E family	SAUSA300_0348
SAUSA300_1628	**1,3**	0,135996321	No	532	920	1114	922	884	1136	729	758	748	lysine-specific permease	lysP
SAUSA300_0062	**1,3**	0,058645342	No	231	305	205	437	392	389	331	324	273	ornithine carbamoyltransferase	arcB
SAUSA300_0021	**1,3**	0,000102273	Yes	5126	5422	5017	10900	11488	11880	8756	9076	8347	sensory box histidine kinase	SAUSA300_0021
SAUSA300_1404	**1,3**	0,20604115	No	57	55	42	59	52	52	50	31	42	phiSLT ORF 563-like protein, terminase, large subunit	SAUSA300_1404
SAUSA300_0143	**1,3**	0,294511469	No	50	64	67	37	20	37	30	19	25	phosphonate ABC transporter, permease protein	phnE
SAUSA300_2646	**1,3**	0,002974418	Yes	785	757	726	1570	1724	1764	1313	1265	1297	tRNA modification GTPase	trmE
SAUSA300_0732	**1,3**	0,000762489	Yes	689	645	637	902	962	895	772	677	665	conserved hypothetical protein	SAUSA300_0732
SAUSA300_2391	**1,3**	0,064063837	No	1421	1432	1112	4795	3656	4870	3910	2698	3589	glycine betaine/carnitine/choline ABC transporter	opuCc

SAUSA300_2032	**1,3**	0,114016979	No	65	86	64	90	108	135	74	91	90 K+-transporting ATPase, C subunit	kdpC
SAUSA300_1268	**1,3**	0,133527622	No	119	127	90	95	122	105	75	79	89 tryptophan synthase, alpha subunit	trpA
SAUSA300_0076	**1,3**	0,008032275	Yes	281	289	254	481	516	432	342	374	377 ABC transporter, ATP-binding protein	SAUSA300_0076
SAUSA300_1500	**1,3**	0,666645325	No	2	7	5	6	2	3	1	4	3 putative competence protein ComYC	SAUSA300_1500
SAUSA300_1312	**1,3**	0,064926411	No	558	779	586	584	568	665	486	475	434 acetyltransferase, GNAT family	SAUSA300_1312
SAUSA300_2521	**1,3**	0,026678277	No	83	71	71	285	248	279	200	192	231 conserved hypothetical protein	SAUSA300_2521
SAUSA300_0617	**1,3**	0,015551031	No	2863	2501	2016	3375	3104	2709	2637	2192	2237 Na+/H+ antiporter	SAUSA300_0617
SAUSA300_0039	**1,3**	0,016379764	No	146	152	128	182	165	180	147	132	129 conserved hypothetical protein	SAUSA300_0039
SAUSA300_1678	**1,3**	0,045381784	No	2084	3293	2150	2836	2786	3417	2384	2525	2045 formate-tetrahydrofolate ligase	fhs
SAUSA300_1701	**1,3**	0,0044746	Yes	889	995	932	849	872	967	682	659	731 conserved hypothetical protein	SAUSA300_1701
SAUSA300_0873	**1,3**	2,95E-06	Yes	6461	6119	5692	7063	7499	7105	5422	5848	5451 coenzyme A disulfide reductase	cdr
SAUSA300_0347	**1,3**	0,037973242	No	818	724	676	1160	1296	1007	929	931	805 Sec-independent protein translocase TatC	tatC
SAUSA300_2484	**1,3**	1,83E-06	Yes	5868	5403	4876	11619	11517	12000	9192	9522	8436 hydroxymethylglutaryl-CoA synthase	SAUSA300_2484
SAUSA300_2413	**1,3**	0,219263842	No	57	64	60	46	59	44	36	36	40 conserved hypothetical protein	SAUSA300_2413
SAUSA300_0995	**1,3**	0,000884523	Yes	35406	42527	40160	38046	39606	44036	30745	32428	30894 dihydrolipoamide acetyltransferase	SAUSA300_0995
SAUSA300_2522	**1,3**	0,227324823	No	45	30	50	160	176	211	127	134	159 conserved hypothetical protein	SAUSA300_2522
SAUSA300_0086	**1,3**	0,121778721	No	152	145	133	212	185	201	160	171	132 conserved hypothetical protein	SAUSA300_0086
SAUSA300_2070	**1,3**	0,000365516	Yes	1111	1248	1153	1124	1147	1056	872	888	819 conserved hypothetical protein	SAUSA300_2070
SAUSA300_0242	**1,3**	0,171501337	No	34	59	56	79	59	80	62	60	49 sorbitol dehydrogenase	gutB
SAUSA300_1859	**1,3**	0,001565528	Yes	2304	2490	2150	3184	3246	3568	2521	2634	2604 conserved hypothetical protein	SAUSA300_1859
SAUSA300_2644	**1,3**	0,001111272	Yes	687	768	778	1471	1575	1652	1190	1249	1207 glucose-inhibited division protein B	gidB
SAUSA300_1035	**1,3**	0,144425272	No	164	141	130	87	115	83	78	63	76 conserved hypothetical protein	SAUSA300_1035
SAUSA300_2557	**1,3**	0,061354169	No	141	118	173	186	167	216	133	152	159 ABC transporter protein	SAUSA300_2557
SAUSA300_1410	**1,3**	0,135742555	No	93	119	114	107	117	129	105	79	90 virulence-associated protein E	SAUSA300_1410
SAUSA300_2461	**1,3**	0,093367679	No	815	579	533	639	453	531	448	453	362 glyoxalase family protein	SAUSA300_2461
SAUSA300_2213	**1,3**	0,001954253	Yes	4110	4321	4618	8149	8506	8997	6734	6720	6493 AcrB/AcrD/AcrF family protein	SAUSA300_2213
SAUSA300_0393	**1,3**	0,291945072	No	9	12	18	44	50	41	38	28	38 conserved hypothetical protein	SAUSA300_0393
SAUSA300_0991	**1,3**	0,005453519	Yes	2771	2382	2223	4215	4528	4021	3519	3320	3099 peptide deformylase	def
SAUSA300_2227	**1,3**	0,004937882	Yes	716	861	746	1002	947	1053	799	737	807 molybdopterin biosynthesis protein B	moeB
SAUSA300_2304	**1,3**	0,00574973	Yes	598	572	460	988	1003	1019	816	768	762 putative membrane protein	SAUSA300_2304
SAUSA300_1482	**1,3**	0,481100878	No	12	5	19	23	25	12	18	15	12 FtsK/SpoIIIE family protein	SAUSA300_1482
SAUSA300_0181	**1,3**	0,014032489	No	5763	5753	5320	6784	6931	6758	5529	4994	5447 non-ribosomal peptide synthetase	SAUSA300_0181
SAUSA300_2542	**1,3**	0,016173705	No	6049	7370	5802	11296	10551	12186	8882	9313	8361 putative AMP-binding enzyme	SAUSA300_2542
SAUSA300_2371	**1,3**	0,238834588	No	47	41	39	72	52	78	54	46	58 biotin synthase	bioB
SAUSA300_0536	**1,3**	0,009102959	Yes	28179	24016	21804	27712	27317	25954	22035	21775	19453 DJ-1/PfpI family protein	SAUSA300_0536
SAUSA300_2551	**1,3**	0,168551951	No	524	478	496	1043	1062	1037	796	781	870 anaerobic ribonucleotide reductase, large subunit	nrdD
SAUSA300_1668	**1,3**	0,012331277	No	667	590	505	1070	985	935	816	764	760 OsmC/Ohr family protein	SAUSA300_1668
SAUSA300_1408	**1,3**	0,223751272	No	59	77	79	103	50	93	70	65	62 phage helicase	SAUSA300_1408
SAUSA300_1139	**1,3**	0,044056992	No	1464	2113	1979	3527	3417	4753	3001	3168	2976 succinyl-CoA synthetase, alpha subunit	sucD
SAUSA300_0894	**1,3**	0,507879786	No	19	16	8	23	25	13	14	14	18 oligopeptide ABC transporter, ATP-binding protein	oppD
SAUSA300_0860	**1,3**	0,015585959	No	596	840	760	1034	956	1041	790	819	767 Ornithine aminotransferase	rocD
SAUSA300_0193	**1,3**	0,418710761	No	12	15	22	20	23	19	12	13	22 conserved hypothetical protein	SAUSA300_0193
SAUSA300_2469	**1,3**	0,039411847	No	224	245	273	609	703	812	519	590	554 L-serine dehydratase, iron-sulfur-dependent, alpha subu	sdaAA
SAUSA300_0544	**1,3**	0,009251422	Yes	1877	2078	1513	1532	1339	1505	1175	1105	1158 hydrolase, haloacid dehalogenase-like family	SAUSA300_0544
SAUSA300_2636	**1,3**	0,075852199	No	351	270	244	266	340	279	209	258	223 integrase/recombinase	SAUSA300_2636
SAUSA300_1621	**1,3**	4,19E-05	Yes	13040	12534	11464	20807	20479	19845	16508	16618	14959 ATP-dependent Clp protease, ATP-binding subunit ClpX	clpX
SAUSA300_1165	**1,3**	0,000555505	Yes	1860	1951	1618	2685	2725	2784	2113	2169	2162 riboflavin biosynthesis protein ribF	ribF
SAUSA300_0316	**1,3**	0,429072436	No	31	67	49	70	52	56	39	49	51 ROK family protein	SAUSA300_0316
SAUSA300_0845	**1,3**	8,33E-05	Yes	2623	2237	2193	3184	3135	2972	2498	2424	2388 cytosol aminopeptidase	ampA
SAUSA300_1120	**1,3**	0,000600701	Yes	1507	1522	1349	2121	2132	1850	1626	1624	1550 ATP-dependent DNA helicase RecG	recG
SAUSA300_1489	**1,3**	0,42466111	No	22	22	18	30	11	24	21	18	15 conserved hypothetical protein	SAUSA300_1489
SAUSA300_0403	**1,3**	0,446265466	No	28	22	25	20	38	16	17	18	19 exotoxin	SAUSA300_0403
SAUSA300_0633	**1,3**	0,095158237	No	1247	1461	1367	1930	1521	2447	1519	1671	1448 ferrichrome transport ATP-binding protein fhuA	fhuA
SAUSA300_2388	**1,3**	0,032540891	No	1433	1710	1568	1724	1911	1968	1360	1537	1506 2-dehydropantoate 2-reductase	panE
SAUSA300_0361	**1,3**	0,212342021	No	227	407	350	392	347	467	304	330	315 ParB-like partition protein	SAUSA300_0361
SAUSA300_0722	**1,3**	0,00014641	Yes	1266	1376	1261	1475	1555	1653	1196	1275	1222 UDP-N-acetylenolpyruvoylglucosamine reductase	murB
SAUSA300_2436	**1,3**	0,145513179	No	1216	2140	2344	1240	1093	1313	999	1000	868 putative cell wall surface anchor family protein	SAUSA300_2436
SAUSA300_0466	**1,3**	0,000714294	Yes	365	381	347	510	572	578	443	441	424 conserved hypothetical protein	SAUSA300_0466
SAUSA300_0521	**1,3**	0,002037714	Yes	3569	3929	3713	4956	5123	5199	4073	4161	3825 transcription termination/antitermination factor NusG	nusG

SAUSA300_2572	**1,3**	0,178422531	No	472	423	266	292	228	272	251	196	180	zinc metalloproteinase aureolysin	aur
SAUSA300_2585	**1,3**	0,105116015	No	191	244	336	955	888	1022	749	650	859	accessory secretory protein Asp3	SAUSA300_2585
SAUSA300_0754	**1,3**	0,029959739	No	2472	2067	1985	2278	1981	2585	1865	1982	1564	conserved hypothetical protein	SAUSA300_0754
SAUSA300_2273	**1,3**	0,129326494	No	1280	2115	1755	1501	1352	1822	1252	1262	1174	Na+/H+ antiporter family protein	SAUSA300_2273
SAUSA300_2373	**1,3**	0,580771348	No	12	11	18	10	14	15	9	9	10	dethiobiotin synthase	bioD
SAUSA300_1137	**1,3**	0,023786929	No	405	496	483	339	394	430	332	278	309	ribonuclease HII	rnhB
SAUSA300_0383	**1,3**	0,010752508	No	5909	6457	4995	6655	6599	7160	5408	5688	5061	conserved hypothetical protein	SAUSA300_0383
SAUSA300_0397	**1,3**	0,408547189	No	16	16	7	24	34	38	17	35	23	exotoxin	SAUSA300_0397
SAUSA300_1750	**1,3**	0,179305712	No	269	279	258	249	300	278	228	171	251	conserved hypothetical protein	SAUSA300_1750
SAUSA300_2231	**1,3**	0,042210371	No	1786	1422	1176	2911	2662	2616	2482	1970	2028	formate dehydrogenase family accessory protein FdhD	fdhD
SAUSA300_1634	**1,3**	0,008597205	Yes	1130	1229	1153	1912	1880	2010	1661	1492	1445	dephospho-CoA kinase	coaE
SAUSA300_1849	**1,3**	0,005003476	Yes	797	914	803	838	778	902	670	661	668	A/G-specific adenine glycosylase	mutY
SAUSA300_0727	**1,3**	3,03E-05	Yes	2894	3003	2574	3605	3563	3794	2975	2999	2727	peptidase T	pepT
SAUSA300_1466	**1,3**	0,00027304	Yes	3612	4007	3232	5844	5926	6220	4979	4828	4463	2-oxoisovalerate dehydrogenase, E1 component, alpha	SAUSA300_1466
SAUSA300_1007	**1,3**	0,002284804	Yes	2537	2242	2015	2674	2578	2641	2138	2206	1920	inositol monophosphatase family protein	SAUSA300_1007
SAUSA300_2295	**1,3**	0,003339396	Yes	389	363	349	716	773	800	576	589	648	conserved hypothetical protein	SAUSA300_2295
SAUSA300_1256	**1,3**	0,169121028	No	865	1309	942	1077	989	1006	779	828	823	peptide methionine sulfoxide reductase MsrA	msrA
SAUSA300_0089	**1,3**	0,018242985	No	1343	1347	1175	1975	2080	2396	1688	1903	1528	Probable tRNA-dihydrouridine synthase	SAUSA300_0089
SAUSA300_2344	**1,3**	0,357343381	No	86	134	139	137	126	167	105	126	110	uroporphyrin-III C-methyl transferase	SAUSA300_2344
SAUSA300_0331	**1,3**	0,411461575	No	19	38	29	40	25	33	26	28	23	conserved hypothetical protein	SAUSA300_0331
SAUSA300_1328	**1,3**	0,169121028	No	90	83	95	77	108	86	70	66	74	putative drug transporter	SAUSA300_1328
SAUSA300_2284	**1,3**	0,003177217	Yes	684	634	599	779	746	762	648	613	558	conserved hypothetical protein	SAUSA300_2284
SAUSA300_0511	**1,3**	0,00777196	Yes	2179	2936	2713	3456	3527	3891	2897	2871	2876	DNA repair protein RadA	radA
SAUSA300_1369	**1,3**	0,024515275	No	651	687	670	1159	1183	1170	966	871	953	pyridine nucleotide-disulfide oxidoreductase	SAUSA300_1369
SAUSA300_1993	**1,3**	0,000129934	Yes	973	1135	1131	1681	1517	1680	1310	1342	1240	kinase, pfkB family	SAUSA300_1993
SAUSA300_1780	**1,3**	0,00294997	Yes	589	627	714	833	895	893	709	659	715	conserved hypothetical protein	SAUSA300_1780
SAUSA300_1252	**1,3**	0,018740875	No	4194	5003	4721	3921	4080	4398	3432	3327	3108	amino acid carrier protein	SAUSA300_1252
SAUSA300_0680	**1,3**	0,068322987	No	1058	1477	1545	1256	1420	1433	1157	1106	1003	multi drug resistance protein	norA
SAUSA300_2176	**1,3**	0,009403136	Yes	1082	1250	1087	1012	969	1119	900	784	789	ABC transporter, ATP-binding protein	SAUSA300_2176
SAUSA300_1313	**1,3**	0,000780927	Yes	3004	2914	2760	3330	3629	3253	2834	2531	2766	carboxyl-terminal protease	ctpA
SAUSA300_1985	**1,3**	0,211394462	No	1624	2443	1488	2327	2518	2789	2109	2239	1715	serine-aspartate repeat family protein, SdrH	sdrH
SAUSA300_2579	**1,3**	0,021482971	No	8989	9397	7458	11467	12331	9742	8619	8926	9234	N-acetylmuramoyl-L-alanine amidase domain protein	SAUSA300_2579
SAUSA300_0459	**1,2**	0,066972752	No	255	311	338	390	518	463	365	375	349	thymidylate kinase	tmk
SAUSA300_2393	**1,2**	0,134900085	No	1099	1393	1022	4325	3074	4738	3504	2719	3465	glycine betaine/carnitine/choline ABC transporter ATP-b	opuCa
SAUSA300_1317	**1,2**	0,044666672	No	3577	3782	2900	3711	3793	3892	3270	3028	2822	methionine-S-sulfoxide reductase	msrA
SAUSA300_2350	**1,2**	0,083829663	No	419	338	357	547	498	489	457	424	349	conserved hypothetical protein	SAUSA300_2350
SAUSA300_0729	**1,2**	0,012389672	No	365	442	330	500	471	489	374	389	408	integral membrane protein	SAUSA300_0729
SAUSA300_1296	**1,2**	0,095786401	No	298	238	242	583	557	574	496	422	454	conserved hypothetical protein	SAUSA300_1296
SAUSA300_0666	**1,2**	0,151497459	No	2210	2155	1567	1764	1497	1937	1512	1444	1211	decarboxylase family protein	SAUSA300_0666
SAUSA300_0355	**1,2**	0,000150025	Yes	1767	1844	1761	3686	3904	4002	3185	3213	2917	acetyl-CoA acetyltransferase	SAUSA300_0355
SAUSA300_2621	**1,2**	0,075824537	No	198	229	175	241	232	237	211	202	159	conserved hypothetical protein	SAUSA300_2621
SAUSA300_0232	**1,2**	0,357063039	No	98	52	70	122	92	108	83	95	81	conserved hypothetical protein	SAUSA300_0232
SAUSA300_1609	**1,2**	0,626267097	No	10	15	7	3	9	13	4	7	8	type III leader peptidase family protein	SAUSA300_1609
SAUSA300_1425	**1,2**	0,646713647	No	0	7	6	9	14	4	6	8	5	conserved hypothetical phage protein	SAUSA300_1425
SAUSA300_2007	**1,2**	0,188167165	No	477	645	484	205	165	197	147	159	151	acetolactate synthase, large subunit	ilvB
SAUSA300_2065	**1,2**	0,009757202	Yes	5811	6503	6392	7686	7826	8475	6493	6561	6279	UDP-N-acetylglucosamine 2-epimerase	SAUSA300_2065
SAUSA300_0783	**1,2**	0,02753517	No	961	886	743	662	584	710	521	508	552	phosphoglycerate mutase family protein	SAUSA300_0783
SAUSA300_0994	**1,2**	0,010967681	No	20732	25829	22888	22817	23263	27366	19736	21043	18448	pyruvate dehydrogenase E1 component, beta subunit	pdhB
SAUSA300_0899	**1,2**	0,092901699	No	6364	4287	4925	8098	7753	6876	6812	5584	5919	putative negative regulator of genetic competence	SAUSA300_0899
SAUSA300_1138	**1,2**	0,201153817	No	722	1367	1443	2284	2294	2966	1842	2106	2128	succinyl-CoA synthetase, beta subunit	sucC
SAUSA300_0010	**1,2**	0,217147612	No	615	840	617	268	257	313	229	240	208	putative membrane protein	SAUSA300_0010
SAUSA300_2502	**1,2**	0,379082354	No	52	44	54	56	38	38	24	45	39	conserved hypothetical protein	SAUSA300_2502
SAUSA300_0194	**1,2**	0,223997355	No	65	86	93	59	74	80	54	59	59	sucrose-specific PTS tranporter protein	SAUSA300_0194
SAUSA300_1146	**1,2**	0,164167309	No	591	650	917	1448	1499	1844	1119	1358	1389	ATP-dependent protease hslV	hslV
SAUSA300_0011	**1,2**	0,287165453	No	357	501	352	193	153	158	140	144	124	conserved hypothetical protein	SAUSA300_0011
SAUSA300_1169	**1,2**	1,33E-06	Yes	5716	5694	5300	6470	6491	6496	5281	5195	5286	DNA translocase FtsK	ftsK
SAUSA300_1569	**1,2**	0,003567487	Yes	4142	4438	3967	4444	4828	4377	3722	3659	3664	peptidase, U32 family	SAUSA300_1569
SAUSA300_2356	**1,2**	0,1258579	No	291	279	214	252	176	200	177	174	161	fmhA protein	fmhA
SAUSA300_2440	**1,2**	0,298930356	No	1671	1148	1895	3470	5333	4244	3158	4483	2875	fibronectin binding protein B	fnbB

SAUSA300_0078	**1,2**	0,013676219	No	3892	3871	3137	13147	12881	12645	11078	9741	10504	ATPase copper transport	copA
SAUSA300_0912	**1,2**	1,12E-07	Yes	5076	5136	5298	7553	7826	7660	6248	6482	5962	trans-2-enoyl-ACP reductase	SAUSA300_0912
SAUSA300_0708	**1,2**	0,058932644	No	582	571	459	766	721	627	625	574	515	histidinol-phosphate aminotransferase	hisC
SAUSA300_2055	**1,2**	0,036407454	No	6641	6913	6908	6327	6832	7440	5691	6032	4979	UDP-N-acetylglucosamine 1-carboxyvinyltransferase 1	murA
SAUSA300_1242	**1,2**	0,03269595	No	303	386	340	672	552	578	484	464	517	exonuclease SbcD	sbcD
SAUSA300_1909	**1,2**	0,309075334	No	3906	5126	2658	4724	3593	4401	3818	3298	3164	conserved hypothetical protein	SAUSA300_1909
SAUSA300_0823	**1,2**	0,763281074	No	2	1	2	0	2	3	1	1	1	hypothetical protein	SAUSA300_0823
SAUSA300_1370	**1,2**	0,025131841	No	17373	19213	14794	23077	21879	25037	19430	18719	18672	cell surface elastin binding protein	ebpS
SAUSA300_2011	**1,2**	0,256549987	No	253	357	294	125	128	117	104	107	89	3-isopropylmalate dehydrogenase	leuB
SAUSA300_0219	**1,2**	0,069022458	No	398	423	348	319	293	308	233	280	236	putative iron compound A C transporter, iron compound	SAUSA300_0219
SAUSA300_1579	**1,2**	0,025198761	No	3743	4070	3157	4611	4621	4705	3986	3639	3694	aminotransferase, class V	SAUSA300_1579
SAUSA300_0200	**1,2**	0,204326126	No	653	939	543	594	647	633	458	538	522	peptide ABC transporter, ATP-binding protein	SAUSA300_0200
SAUSA300_1209	**1,2**	0,425085817	No	126	190	91	100	88	109	79	77	85	conserved hypothetical protein	SAUSA300_1209
SAUSA300_0329	**1,2**	0,122486393	No	1190	1373	1190	1098	1080	1390	1011	996	895	putative oxidoreductase	SAUSA300_0329
SAUSA300_2489	**1,2**	0,112853727	No	377	413	292	279	270	241	223	221	199	antibiotic transport-associated protein-like protein	SAUSA300_2489
SAUSA300_1428	**1,2**	0,766408123	No	0	1	0	1	2	1	1	1	1	conserved hypothetical phage protein	SAUSA300_1428
SAUSA300_1595	**1,2**	0,058752385	No	1910	2447	2862	4951	5563	6149	4474	4792	4292	queuine tRNA-ribosyltransferase	tgt
SAUSA300_0955	**1,2**	0,130720485	No	32811	34227	29419	23476	29141	28373	20274	23060	22607	autolysin	atl
SAUSA300_1645	**1,2**	0,133617553	No	5933	9431	8034	9993	9548	12109	8308	9224	8244	6-phosphofructokinase	pfkA
SAUSA300_0250	**1,2**	0,005325733	Yes	3806	3849	3555	7160	7808	7057	5934	6045	5983	alcohol dehydrogenase, zinc-containing	SAUSA300_0250
SAUSA300_1647	**1,2**	0,000365217	Yes	3161	3204	2921	3646	3915	3697	3173	3045	2968	acetyl-CoA carboxylase, carboxyl transferase, beta subu	accD
SAUSA300_0749	**1,2**	0,067916868	No	2019	2324	2113	2359	2207	2569	1923	2100	1798	conserved hypothetical protein	SAUSA300_0749
SAUSA300_1167	**1,2**	0,006497174	Yes	9626	10002	11159	11762	12759	12442	9821	10780	9567	polyribopolyribonucleotide nucleotidyltransferase	pnpA
SAUSA300_2531	**1,2**	0,145481148	No	389	426	289	422	482	463	392	393	330	hydrolase, CocE/NonD family	SAUSA300_2531
SAUSA300_0322	**1,2**	0,135417159	No	518	664	512	709	780	893	607	707	630	NADH-dependent flavin oxidoreductase, Oye family	SAUSA300_0322
SAUSA300_2254	**1,2**	0,03269595	No	2432	2430	2003	2344	2189	2668	1981	2090	1825	glycerate dehydrogenase-like protein	SAUSA300_2254
SAUSA300_1691	**1,2**	0,000642375	Yes	1331	1185	1084	2164	2087	2044	1775	1742	1640	glutamyl-aminopeptidase	SAUSA300_1691
SAUSA300_1730	**1,2**	0,080761995	No	7647	7934	6989	8973	8299	9009	7278	7373	6870	S-adenosylmethionine synthetase	metK
SAUSA300_2568	**1,2**	0,32613024	No	31	74	61	77	70	102	64	76	65	arginine/ornithine antiporter	arcD
SAUSA300_1987	**1,2**	0,021889137	No	1039	1061	900	1157	980	1019	872	809	912	hydrolase, carbon-nitrogen family	SAUSA300_1987
SAUSA300_2292	**1,2**	0,005465994	Yes	1821	2085	1898	1816	1972	2125	1554	1677	1620	isopentenyl-diphosphate delta-isomerase	fni
SAUSA300_2219	**1,2**	0,025131841	No	1652	1745	1551	2539	2405	2336	2119	1999	1855	molybdenum cofactor biosynthesis protein A	moaA
SAUSA300_2175	**1,2**	0,014096716	No	1922	2077	1651	1753	1868	1788	1545	1454	1439	ABC transporter, ATP-binding protein	SAUSA300_2175
SAUSA300_0522	**1,2**	0,127627894	No	23875	23715	22330	24184	26166	27528	21577	23410	18857	ribosomal protein L11	rplK
SAUSA300_2169	**1,2**	0,043613583	No	537	656	582	603	487	586	453	467	463	conserved hypothetical protein	SAUSA300_2169
SAUSA300_2306	**1,2**	0,373828717	No	67	81	84	56	59	59	36	55	50	ABC transporter, ATP-binding protein	SAUSA300_2306
SAUSA300_0343	**1,2**	0,342819014	No	443	791	529	291	289	389	269	251	274	acetyltransferase, GNAT family	SAUSA300_0343
SAUSA300_2296	**1,2**	0,026623419	No	2840	3073	2447	2478	2378	2487	2058	2066	1932	esterase-like protein	SAUSA300_2296
SAUSA300_1130	**1,2**	0,012135357	No	3031	3529	3674	5939	6164	6709	5077	5137	5313	signal recognition particle protein	ffh
SAUSA300_2541	**1,2**	0,022808256	No	56601	59300	50638	48183	48856	52914	41657	44958	37147	malate:quinone-oxidoreductase	mqo
SAUSA300_2586	**1,2**	0,061657002	No	591	754	774	2406	2065	2231	1897	1754	1887	accessory secretory protein Asp2	SAUSA300_2586
SAUSA300_2445	**1,2**	0,002696618	Yes	1070	988	1036	2303	2346	2305	1835	1882	2028	transcriptional regulator, MerR family	SAUSA300_2445
SAUSA300_0020	**1,2**	0,006259956	Yes	2055	2071	2035	4521	4657	4923	3950	4063	3646	DNA-binding response regulator	SAUSA300_0020
SAUSA300_1635	**1,2**	0,00875666	Yes	930	1136	1064	1645	1479	1405	1307	1224	1219	formamidopyrimidine-DNA glycosylase	mutM
SAUSA300_1056	**1,2**	0,332992819	No	1812	891	917	752	935	699	650	643	670	conserved hypothetical protein	SAUSA300_1056
SAUSA300_2392	**1,2**	0,143672316	No	615	678	576	2248	1603	2274	1929	1394	1748	glycine betaine/carnitine/choline ABC transporter	opuCb
SAUSA300_1669	**1,2**	0,07462998	No	1016	1259	872	494	518	516	441	418	407	aminotransferase, class V	SAUSA300_1669
SAUSA300_1152	**1,2**	0,012774573	No	3901	4690	4425	4497	4503	4924	3806	3818	3911	ribosome recycling factor	rrf
SAUSA300_1250	**1,2**	0,020145459	No	3398	4230	3709	5294	5509	5566	4491	4566	4511	DNA topoisomerase IV, subunit B	parE
SAUSA300_1031	**1,2**	0,30662715	No	143	170	160	102	140	121	88	87	121	conserved hypothetical protein	SAUSA300_1031
SAUSA300_1483	**1,2**	0,135417159	No	126	155	131	120	137	127	103	107	107	conserved hypothetical protein	SAUSA300_1483
SAUSA300_1526	**1,2**	0,019079849	No	763	873	891	1136	1159	1158	926	955	982	DNA repair protein RecO	recO
SAUSA300_1173	**1,2**	0,040247646	No	1364	1385	1440	1355	1501	1609	1249	1157	1297	putative acetoacetyl-CoA reductase	SAUSA300_1173
SAUSA300_2238	**1,2**	0,20965523	No	47	41	50	1684	1400	1973	1402	1452	1340	urease, gamma subunit	ureA
SAUSA300_0593	**1,2**	0,007673921	Yes	1509	1455	1385	1720	1508	1767	1435	1287	1437	conserved hypothetical protein	SAUSA300_0593
SAUSA300_0827	**1,2**	0,022891117	No	463	468	464	878	847	747	709	691	653	putative membrane protein	SAUSA300_0827
SAUSA300_2567	**1,2**	0,078739784	No	357	360	306	577	615	694	522	540	505	carbamate kinase	arcC
SAUSA300_1704	**1,2**	0,137503328	No	3500	5119	4190	4057	4089	4615	3531	3635	3427	leucyl-tRNA synthetase	leuS
SAUSA300_0015	**1,2**	0,023022483	No	3911	4245	3487	5736	5646	5575	4795	4899	4414	50S ribosomal protein L9	rplI

SAUSA300_0841	**1,2**	0,054329211	No	2833	3236	2805	3151	3273	3462	2708	3012	2506	conserved hypothetical protein	SAUSA300_0841
SAUSA300_2635	**1,2**	0,733158169	No	3	4	7	3	16	6	4	9	5	conserved hypothetical protein	SAUSA300_2635
SAUSA300_1373	**1,2**	0,171215439	No	915	753	711	856	753	918	772	649	684	ferredoxin	SAUSA300_1373
SAUSA300_0829	**1,2**	0,032118725	No	4976	6097	5108	6666	6421	7159	5986	5830	5043	lipoic acid synthetase	lipA
SAUSA300_1043	**1,2**	0,002823033	Yes	1800	1966	1733	2655	2766	2532	2297	2170	2154	DNA mismatch repair MutS2 protein	mutS2
SAUSA300_2240	**1,2**	0,210392366	No	415	448	491	10623	9356	11218	8338	9013	8595	urease, alpha subunit	ureC
SAUSA300_0494	**1,2**	0,008041643	Yes	1361	1478	1262	1240	1276	1281	1085	1096	985	2-amino-4-hydroxy-6-hydroxymethyldihydropteridine p	folK
SAUSA300_0493	**1,2**	0,016319881	No	422	509	433	686	667	655	567	604	507	dihydroneopterin aldolase	folB
SAUSA300_0085	**1,2**	0,309075334	No	29	30	40	105	86	83	75	81	71	conserved hypothetical protein	SAUSA300_0085
SAUSA300_0364	**1,2**	0,15561274	No	965	1387	1631	1951	2013	2353	1818	1781	1672	GTP-binding protein YchF	ychF
SAUSA300_0433	**1,2**	0,128292624	No	568	641	581	2114	2076	2532	1668	1882	2062	cysteine synthase/cystathionine beta-synthase	cysM
SAUSA300_1614	**1,2**	0,076394193	No	6352	6254	4609	6824	6117	6242	5752	5303	4992	glutamate-1-semialdehyde-2,1-aminomutase	hemL
SAUSA300_1650	**1,2**	0,004710537	Yes	1311	1362	1168	1448	1521	1498	1193	1345	1199	conserved hypothetical protein	SAUSA300_1650
SAUSA300_0864	**1,2**	0,194791974	No	465	478	382	295	311	288	243	275	228	argininosuccinate synthase	argG
SAUSA300_1194	**1,2**	0,206158563	No	226	298	256	239	293	278	207	206	261	hydrolase, alpha/beta hydrolase fold family	SAUSA300_1194
SAUSA300_0907	**1,2**	0,142751287	No	1521	1989	1488	1919	1736	1983	1504	1642	1574	GTP pyrophosphokinase	SAUSA300_0907
SAUSA300_0737	**1,2**	0,000379133	Yes	11466	10981	11605	22634	24500	23148	19318	19871	19705	preprotein translocase, SecA subunit	secA
SAUSA300_1453	**1,2**	0,017813759	No	1326	1388	1192	879	816	847	711	727	698	ribonuclease Z	SAUSA300_1453
SAUSA300_2457	**1,2**	0,390581029	No	31	48	49	72	52	84	67	54	57	phospholipase/carboxylesterase family protein	SAUSA300_2457
SAUSA300_1462	**1,2**	0,025131841	No	1300	1155	995	1542	1332	1411	1242	1189	1170	conserved hypothetical protein	SAUSA300_1462
SAUSA300_2281	**1,2**	0,323517026	No	832	1051	866	768	591	1037	726	683	601	formimidoylglutamase	hutG
SAUSA300_1848	**1,2**	0,058728526	No	815	793	713	1064	1147	1258	1027	948	939	conserved hypothetical protein	SAUSA300_1848
SAUSA300_0262	**1,2**	0,041028138	No	374	365	355	553	491	584	434	503	439	ribokinase	rbsK
SAUSA300_0930	**1,2**	0,182253498	No	1090	1101	945	2185	1805	1800	1753	1575	1535	lipoate-protein ligase A family protein	SAUSA300_0930
SAUSA300_2504	**1,2**	0,001899709	Yes	7005	6583	6659	11225	11770	11246	9192	9890	9696	acyltransferase	SAUSA300_2504
SAUSA300_0030	**1,2**	0,042517426	No	791	918	832	1635	1530	1483	1380	1282	1250	putative glycerophosphoryl diester phosphodiesterase	SAUSA300_0030
SAUSA300_1327	**1,2**	0,185433875	No	1666	2190	1958	2044	2040	1961	1405	1917	1758	cell surface protein	SAUSA300_1327
SAUSA300_1126	**1,2**	0,175480275	No	224	334	408	597	600	670	495	514	563	ribonuclease III	rnc
SAUSA300_0259	**1,2**	0,434129905	No	48	140	98	112	104	98	83	98	82	PTS system, IIA component	SAUSA300_0259
SAUSA300_2416	**1,2**	0,481375083	No	310	350	377	200	144	353	205	196	189	glucose 1-dehydrogenase-like protein	SAUSA300_2416
SAUSA300_0480	**1,2**	0,12314628	No	623	652	721	849	805	908	709	773	679	peptidyl-tRNA hydrolase	pth
SAUSA300_1006	**1,2**	0,097279055	No	2950	2264	2079	2897	2524	2569	2451	2056	2231	conserved hypothetical protein	SAUSA300_1006
SAUSA300_0572	**1,2**	0,216024078	No	773	1038	872	1290	1291	1594	1282	1169	1068	mevalonate kinase	mvk
SAUSA300_0622	**1,2**	0,237734574	No	186	137	131	149	171	186	157	128	141	putative membrane protein	SAUSA300_0622
SAUSA300_2428	**1,2**	0,390581029	No	119	93	82	89	97	106	81	82	83	staphylococcal tandem lipoprotein	SAUSA300_2428
SAUSA300_1314	**1,2**	0,217981202	No	1528	1659	1241	1956	1675	1718	1652	1484	1378	conserved hypothetical protein	SAUSA300_1314
SAUSA300_0527	**1,2**	0,041274154	No	54156	59298	49264	79899	80773	83059	71693	68256	65830	DNA-directed RNA polymerase, beta subunit	rpoB
SAUSA300_0594	**1,2**	0,677317943	No	348	438	797	405	410	460	261	516	282	alcohol dehydrogenase	adh
SAUSA300_0424	**1,2**	0,386246124	No	67	56	55	130	104	102	108	84	92	putative cobalamin synthesis protein	SAUSA300_0424
SAUSA300_2157	**1,2**	0,135500507	No	518	549	450	581	548	596	514	504	444	NAD-dependent deacetylase	SAUSA300_2157
SAUSA300_1654	**1,2**	0,135742555	No	1924	2029	1616	2699	2436	2335	2218	2174	1924	proline dipeptidase	SAUSA300_1654
SAUSA300_1481	**1,2**	0,254391276	No	295	256	193	222	201	198	195	184	147	putative membrane protein	SAUSA300_1481
SAUSA300_2581	**1,2**	0,052197452	No	561	728	666	1392	1163	1344	1086	1045	1173	putative surface anchored protein	SAUSA300_2581
SAUSA300_1648	**1,2**	0,044126342	No	5086	5594	4658	5356	5637	5927	4816	4929	4576	putative NADP-dependent malic enzyme	SAUSA300_1648
SAUSA300_0758	**1,2**	0,370187706	No	7472	10867	10254	10423	11037	12664	8879	10978	8957	triosephosphate isomerase	tpiA
SAUSA300_0496	**1,2**	0,009948595	Yes	13095	12852	12638	14179	15419	13508	11824	12867	11809	lysyl-tRNA synthetase	lysS
SAUSA300_0775	**1,2**	0,563892021	No	17	10	16	34	23	33	30	28	19	conserved hypothetical protein	SAUSA300_0775
SAUSA300_2215	**1,2**	0,42127783	No	102	246	238	349	268	342	241	260	311	conserved hypothetical protein	SAUSA300_2215
SAUSA300_2103	**1,2**	0,152443129	No	117	144	140	272	304	337	257	261	255	ABC transporter, ATP-binding protein	SAUSA300_2103
SAUSA300_1449	**1,2**	0,132255546	No	327	305	317	440	428	426	375	339	382	MutT/nudix family protein	SAUSA300_1449
SAUSA300_0946	**1,2**	0,076488961	No	1824	1730	1805	2496	2495	2650	2190	2110	2178	2-succinyl-6-hydroxy-2,4-cyclohexadiene-1-carboxylic a	menD
SAUSA300_1349	**1,2**	0,017915494	No	846	820	895	1209	1321	1226	1105	1009	1065	glycosyl transferase, group 1 family protein	SAUSA300_1349
SAUSA300_2110	**1,2**	0,308678383	No	744	1075	553	841	820	969	702	706	818	truncated FmtB protein	fmtB
SAUSA300_2595	**1,2**	0,323387307	No	71	55	47	119	95	118	89	100	95	acetyltransferase, GNAT family	SAUSA300_2595
SAUSA300_0840	**1,2**	0,224861448	No	76	90	114	199	194	226	176	189	162	conserved hypothetical protein	SAUSA300_0840
SAUSA300_1886	**1,2**	0,023398387	No	2907	3662	3059	4613	4575	4877	4117	3968	3868	ATP-dependent DNA helicase, PcrA	pcrA
SAUSA300_1869	**1,2**	0,01200918	No	3138	2940	2911	4580	4055	4555	3835	3888	3505	methionine aminopeptidase	map
SAUSA300_0005	**1,2**	0,088372944	No	4685	6373	5656	9410	8901	10307	8371	8217	7764	DNA gyrase, B subunit	gyrB
SAUSA300_1719	**1,2**	0,159014409	No	164	162	155	170	214	210	171	163	168	arsenate reductase	arsC

SAUSA300_1090	**1,2**	0,037432977	No	1442	1332	1437	1726	1990	1785	1519	1585	1571	conserved hypothetical protein	SAUSA300_1090
SAUSA300_0315	**1,2**	0,680098307	No	24	25	16	17	14	15	11	16	12	N-acetylneuraminate lyase subunit	nanA
SAUSA300_0872	**1,2**	0,211555059	No	336	335	380	327	473	426	352	318	367	conserved hypothetical protein	SAUSA300_0872
SAUSA300_2058	**1,2**	0,042976063	No	23334	26870	23178	32109	32211	34656	28308	30176	25949	ATP synthase F1, beta subunit	atpD
SAUSA300_0636	**1,2**	0,348883078	No	52	75	60	122	90	146	91	117	102	dihydroxyacetone kinase, DhaK subunit	SAUSA300_0636
SAUSA300_0082	**1,2**	0,259768142	No	489	567	390	430	467	501	399	393	398	conserved hypothetical protein	SAUSA300_0082
SAUSA300_1464	**1,2**	0,005680398	Yes	4716	4939	4147	7299	7156	6965	6373	6145	5776	2-oxoisovalerate dehydrogenase, E2 component, dihydr	SAUSA300_1464
SAUSA300_0570	**1,2**	0,051655307	No	9402	9832	7618	7538	7100	7934	6762	6726	5789	phosphate acetyltransferase	pta
SAUSA300_1100	**1,2**	0,091975293	No	1018	793	797	1230	1118	1051	1009	939	956	conserved hypothetical protein	SAUSA300_1100
SAUSA300_1584	**1,2**	0,048796834	No	3498	3158	2594	2340	2319	2478	2026	2145	1928	ATPase, AAA family	SAUSA300_1584
SAUSA300_1245	**1,2**	0,145429701	No	10041	11968	12421	12565	12678	13396	11042	10742	11204	glycine betaine transporter opuD	opuD
SAUSA300_1406	**1,2**	0,797252848	No	0	0	1	3	0	0	1	1	0	phiSLT ORF 104b-like protein	SAUSA300_1406
SAUSA300_0079	**1,2**	0,092238539	No	1168	1085	973	3891	3805	4058	3531	3078	3434	putative lipoprotein	SAUSA300_0079
SAUSA300_1019	**1,2**	0,076414066	No	1071	880	805	1149	1091	1180	1032	937	958	conserved hypothetical protein	SAUSA300_1019
SAUSA300_1994	**1,2**	0,182422202	No	1619	2342	2317	3098	2894	3506	2545	2842	2735	sucrose-6-phosphate hydrolase	scrB
SAUSA300_1512	**1,2**	0,044634086	No	6044	6090	4779	7279	7215	6802	6106	6223	5893	penicillin-binding protein 3	pbp3
SAUSA300_1034	**1,2**	0,509120911	No	100	93	118	54	79	47	45	46	59	sortase B	srtB
SAUSA300_2442	**1,2**	0,253716835	No	289	285	301	455	403	492	358	472	331	gluconate permease	gntP
SAUSA300_1368	**1,2**	0,094631334	No	1452	1524	1237	1359	1278	1387	1212	1107	1135	L-asparaginase	ansA
SAUSA300_0367	**1,2**	0,395407085	No	5977	8748	10889	13188	13149	17023	12394	13540	11191	single-strand binding protein	ssb
SAUSA300_2578	**1,2**	0,042204188	No	7079	6706	5841	8300	8423	8689	7070	7409	7338	putative phage infection protein	SAUSA300_2578
SAUSA300_1720	**1,2**	0,147960933	No	8808	7044	6448	9521	9527	9625	8582	7553	8473	conserved hypothetical protein	SAUSA300_1720
SAUSA300_0824	**1,2**	0,22625843	No	737	1002	789	1130	1066	992	934	895	908	conserved hypothetical protein	SAUSA300_0824
SAUSA300_0637	**1,2**	0,290998203	No	52	67	63	133	106	123	104	115	95	dihydroxyacetone kinase, DhaL subunit	SAUSA300_0637
SAUSA300_1563	**1,2**	0,228403665	No	117	172	160	306	257	352	261	277	254	acetyl-CoA carboxylase, biotin carboxylase	accC
SAUSA300_1885	**1,2**	0,014246648	No	3102	3474	3305	5168	5238	5343	4820	4454	4280	DNA ligase	ligA
SAUSA300_0892	**1,2**	0,696953605	No	16	22	27	14	23	9	14	12	11	oligopeptide ABC transporter, oligopeptide-binding prot	oppA
SAUSA300_1890	**1,2**	0,421929405	No	1076	1061	837	139	149	143	128	128	114	staphopain A	SAUSA300_1890
SAUSA300_1214	**1,2**	0,459137776	No	93	179	134	249	178	297	200	210	216	conserved hypothetical protein	SAUSA300_1214
SAUSA300_2214	**1,2**	0,129285582	No	1896	2480	2420	3778	3509	4166	3245	3340	3279	FmhB protein	SAUSA300_2214
SAUSA300_1695	**1,2**	0,058932644	No	7458	6940	6277	5839	5569	6248	5075	5209	4928	conserved hypothetical protein	SAUSA300_1695
SAUSA300_0870	**1,2**	0,017180012	No	4079	4509	3833	5032	5261	5162	4514	4405	4395	exonuclease RexA	rexA
SAUSA300_0953	**1,2**	0,632418049	No	90	53	89	196	316	223	212	153	260	putative membrane protein	SAUSA300_0953
SAUSA300_1326	**1,2**	0,382677636	No	224	201	184	354	419	393	377	313	312	putative cell wall enzyme EbsB	SAUSA300_1326
SAUSA300_0996	**1,2**	0,018835448	No	42936	47779	45256	42504	43115	47193	38002	40035	36561	dihydrolipoamide dehydrogenase	lpdA
SAUSA300_0006	**1,2**	0,034781095	No	9664	10851	9356	14632	14540	16504	13900	13361	12156	DNA gyrase, A subunit	gyrA
SAUSA300_1697	**1,2**	0,127048656	No	4170	5508	4817	4856	4794	5377	4293	4495	4185	Peptidase family M20/M25/M40	SAUSA300_1697
SAUSA300_1293	**1,2**	0,129482102	No	1426	1611	1502	1160	1244	1236	1058	974	1109	diaminopimelate decarboxylase	lysA
SAUSA300_2012	**1,2**	0,372089475	No	355	448	407	166	178	169	138	155	148	3-isopropylmalate dehydratase, large subunit	leuC
SAUSA300_0635	**1,2**	0,126448722	No	1228	1440	1467	1608	1535	1912	1428	1517	1426	ferrichrome transport permease protein fhuG	fhuG
SAUSA300_0902	**1,2**	0,171947975	No	6876	7080	5817	10635	10070	9201	9145	8207	8472	oligoendopeptidase F	pepF
SAUSA300_2008	**1,2**	0,699055766	No	36	53	50	13	20	10	13	10	13	acetolactate synthase, small subunit	ilvN
SAUSA300_0487	**1,2**	0,028315629	No	1154	1205	1159	1538	1510	1607	1439	1328	1265	MesJ/Ycf62 family protein	SAUSA300_0487
SAUSA300_0074	**1,2**	0,146870705	No	389	437	359	614	739	677	628	595	530	oligopeptide permease, channel-forming protein	opp-3B
SAUSA300_0184	**1,2**	0,313819674	No	327	327	251	249	273	278	225	241	224	acetylglutamate kinase	argB
SAUSA300_0057	**1,2**	0,714450031	No	16	11	8	24	9	16	17	13	14	conserved hypothetical protein	SAUSA300_0057
SAUSA300_1596	**1,2**	0,229336715	No	925	1306	1359	1645	1690	1751	1403	1469	1531	S-adenosylmethionine:tRNA ribosyltransferase-isomera	queA
SAUSA300_0564	**1,2**	0,20335134	No	661	720	756	656	699	800	605	621	640	conserved hypothetical protein	SAUSA300_0564
SAUSA300_1753	**1,2**	0,690692342	No	16	18	13	10	11	21	11	12	14	serine protease SplF	splF
SAUSA300_1116	**1,2**	0,047845003	No	828	966	876	1202	1260	1254	1038	1151	1034	thiamine pyrophosphokinase	SAUSA300_1116
SAUSA300_0600	**1,2**	0,151625997	No	467	598	553	553	570	526	443	471	514	hydrolase, haloacid dehalogenase-like family	SAUSA300_0600
SAUSA300_1177	**1,2**	0,103878127	No	551	698	615	1021	1059	973	923	826	901	competence/damage-inducible protein cinA	cinA
SAUSA300_1801	**1,2**	0,194828054	No	1087	1263	1031	1020	1095	1189	896	1095	880	fumarate hydratase, class II	fumC
SAUSA300_1379	**1,1**	0,673401448	No	12	5	8	17	29	25	24	15	21	putative lipoprotein	SAUSA300_1379
SAUSA300_1622	**1,1**	0,110029334	No	21460	22103	19251	28391	26474	26767	24869	24708	21413	trigger factor	tig
SAUSA300_0112	**1,1**	0,364285867	No	6390	8102	5764	3353	3153	3595	3213	2723	2845	L-lactate permease	lctP
SAUSA300_0516	**1,1**	0,310950218	No	350	445	476	564	505	521	443	479	462	conserved hypothetical protein	SAUSA300_0516
SAUSA300_0476	**1,1**	0,792342634	No	0	1	0	0	2	0	1	0	0	hypothetical protein	SAUSA300_0476
SAUSA300_2420	**1,1**	0,390184972	No	119	99	114	122	110	126	115	88	110	conserved hypothetical protein	SAUSA300_2420

SAUSA300_0759	**1,1**	0,444187685	No	16786	22898	20543	20777	22350	26025	19737	22133	18268	2,3-bisphosphoglycerate-independent phosphoglycerat	gpmI
SAUSA300_0467	**1,1**	0,179010782	No	8684	11375	8932	7412	7368	7351	6493	6393	6396	methionyl-tRNA synthetase	metS
SAUSA300_2159	**1,1**	0,230922148	No	815	522	593	1879	1968	1704	1594	1654	1585	aldo/keto reductase family protein	SAUSA300_2159
SAUSA300_1656	**1,1**	0,235518989	No	2508	2576	2217	2679	2766	3375	2590	2769	2329	universal stress protein family	SAUSA300_1656
SAUSA300_1807	**1,1**	0,220663255	No	391	344	305	236	239	254	217	211	208	amino acid ABC transporter, ATP-binding protein	SAUSA300_1807
SAUSA300_0432	**1,1**	0,381847896	No	481	519	298	463	575	460	469	372	458	sodium dependent transporter	SAUSA300_0432
SAUSA300_1646	**1,1**	0,054408347	No	3024	3209	2991	4109	4224	3997	3729	3620	3408	acetyl-CoA carboxylase, carboxyl transferase, alpha sub	accA
SAUSA300_1013	**1,1**	0,193313768	No	2454	2016	2037	3818	3958	3769	3463	3400	3210	cell division protein, FtsW/RodA/SpoVE family	SAUSA300_1013
SAUSA300_1112	**1,1**	0,312272795	No	1421	1888	1945	1562	1722	1680	1350	1450	1530	protein phosphatase 2C domain protein	SAUSA300_1112
SAUSA300_0052	**1,1**	0,707496044	No	0	0	0	1	0	0	0	0	0	hypothetical protein	SAUSA300_0052
SAUSA300_0065	**1,1**	0,306981131	No	158	224	159	378	331	356	332	282	319	arginine deiminase	arcA
SAUSA300_1199	**1,1**	0,065477679	No	1102	1112	1079	1292	1208	1187	1118	1036	1071	putative aluminium resistance protein	SAUSA300_1199
SAUSA300_0577	**1,1**	0,556608772	No	236	301	148	255	187	211	242	150	178	putative transcriptional regulator	SAUSA300_0577
SAUSA300_1150	**1,1**	0,075754001	No	14053	16226	14736	18112	17655	19657	16145	16959	15361	translation elongation factor Ts	tsf
SAUSA300_0464	**1,1**	0,332675828	No	200	167	201	262	241	327	257	252	221	Methyltransferase	SAUSA300_0464
SAUSA300_0866	**1,1**	0,408547189	No	648	587	597	660	663	497	575	497	516	conserved hypothetical protein	SAUSA300_0866
SAUSA300_2029	**1,1**	0,137625527	No	463	527	461	557	559	500	483	451	478	conserved hypothetical protein	SAUSA300_2029
SAUSA300_0910	**1,1**	0,112052994	No	1619	1960	1639	2214	2328	2146	2059	1957	1839	magnesium transporter	mgtE
SAUSA300_1578	**1,1**	0,158390146	No	4535	4764	3960	4899	5125	4779	4468	4239	4256	tRNA (5-methylaminomethyl-2-thiouridylate)-methyltra	trmU
SAUSA300_1537	**1,1**	0,122360782	No	780	887	875	851	967	933	812	832	762	conserved hypothetical protein	SAUSA300_1537
SAUSA300_0936	**1,1**	0,615441509	No	41	38	44	42	50	35	39	39	31	ABC transporter, ATP-binding protein	SAUSA300_0936
SAUSA300_2430	**1,1**	0,362739009	No	245	357	281	352	340	413	352	274	343	staphylococcal tandem lipoprotein	SAUSA300_2430
SAUSA300_1363	**1,1**	0,034332041	No	3839	4024	4015	3748	4420	4115	3520	3758	3485	glycerol-3-phosphate dehydrogenase, NAD-dependent	gpsA
SAUSA300_0678	**1,1**	0,710535355	No	134	90	155	137	214	140	134	112	179	putative membrane protein	SAUSA300_0678
SAUSA300_1003	**1,1**	0,098720421	No	14755	12764	12199	14289	13742	13616	12120	12307	12117	conserved hypothetical protein	SAUSA300_1003
SAUSA300_1479	**1,1**	0,723946882	No	19	16	12	19	9	18	12	15	14	conserved hypothetical protein	SAUSA300_1479
SAUSA300_0022	**1,1**	0,084073241	No	5417	5537	4774	9394	9730	8747	8324	8254	7909	YycH protein	SAUSA300_0022
SAUSA300_0952	**1,1**	0,144879587	No	2258	2031	2014	1806	2026	1656	1675	1564	1578	aminotransferase, class I	SAUSA300_0952
SAUSA300_2360	**1,1**	0,349990038	No	892	879	1253	902	1066	1254	911	1013	909	multidrug resistance protein	SAUSA300_2360
SAUSA300_1187	**1,1**	0,155429705	No	1721	1670	1334	1391	1359	1301	1187	1211	1166	conserved hypothetical protein	SAUSA300_1187
SAUSA300_0223	**1,1**	0,411850788	No	129	100	85	165	185	152	156	147	136	conserved hypothetical protein	SAUSA300_0223
SAUSA300_1659	**1,1**	0,155814171	No	5606	5187	4212	8300	7936	8463	7885	7061	6795	thiol peroxidase	tpx
SAUSA300_0528	**1,1**	0,201030633	No	56002	61345	53942	78987	82356	79999	72424	74168	65775	DNA-directed RNA polymerase, beta' subunit	rpoC
SAUSA300_0826	**1,1**	0,135336407	No	866	869	795	1561	1422	1247	1293	1196	1236	conserved hypothetical protein	SAUSA300_0826
SAUSA300_1860	**1,1**	0,124766627	No	3300	3658	3415	4080	4447	4062	3665	3936	3486	aminopeptidase PepS	pepS
SAUSA300_1322	**1,1**	0,109329199	No	2105	2524	2045	2078	2038	2175	1936	1916	1698	conserved hypothetical protein	SAUSA300_1322
SAUSA300_1365	**1,1**	0,226415041	No	19618	21756	17565	28662	26670	26258	25098	23986	22871	30S ribosomal protein S1	rpsA
SAUSA300_1246	**1,1**	0,223751272	No	5108	5319	4572	8234	8360	7831	8019	7347	6181	aconitate hydratase	acnA
SAUSA300_0876	**1,1**	0,443499821	No	160	134	160	175	171	152	162	124	152	putative membrane protein	SAUSA300_0876
SAUSA300_2030	**1,1**	0,225552723	No	996	1206	1220	1150	1161	1165	1008	990	1068	putative membrane protein	SAUSA300_2030
SAUSA300_2156	**1,1**	0,22602711	No	680	827	704	740	739	676	628	670	607	lactose phosphotransferase system repressor	SAUSA300_2156
SAUSA300_2006	**1,1**	0,643106919	No	389	642	450	146	133	176	148	135	120	dihydroxy-acid dehydratase	ilvD
SAUSA300_1808	**1,1**	0,398094511	No	203	264	223	140	169	160	153	135	124	amino acid ABC transporter, permease/substrate-bindir	SAUSA300_1808
SAUSA300_1087	**1,1**	0,079234728	No	10677	11420	10120	10862	11159	11085	9406	10387	9493	isoleucyl-tRNA synthetase	ileS
SAUSA300_2587	**1,1**	0,103469221	No	1087	1176	1124	2942	2784	2709	2420	2410	2633	accessory secretory protein Asp1	SAUSA300_2587
SAUSA300_2520	**1,1**	0,332202968	No	177	133	120	299	239	254	219	235	249	transporter gate domain protein	SAUSA300_2520
SAUSA300_2524	**1,1**	0,428578222	No	72	57	68	285	248	254	223	225	248	conserved hypothetical protein	SAUSA300_2524
SAUSA300_0048	**1,1**	0,818401552	No	14	11	13	6	5	7	7	4	5	hypothetical protein	SAUSA300_0048
SAUSA300_1174	**1,1**	0,066383168	No	2639	2861	2704	2774	2675	2551	2406	2346	2329	conserved hypothetical protein	SAUSA300_1174
SAUSA300_0793	**1,1**	0,426345722	No	351	419	335	408	385	361	365	338	318	conserved hypothetical protein	SAUSA300_0793
SAUSA300_0756	**1,1**	0,638053349	No	38060	43886	51962	44688	46667	52473	36563	54075	36106	glyceraldehyde-3-phosphate dehydrogenase, type I	gap
SAUSA300_0245	**1,1**	0,372089475	No	754	925	949	485	478	563	420	480	452	2-C-methyl-D-erythritol 4-phosphate cytidylyltransferas	SAUSA300_0245
SAUSA300_2584	**1,1**	0,395808536	No	722	972	1238	2021	1995	2264	1842	1691	2022	preprotein translocase, secA protein	SAUSA300_2584
SAUSA300_1998	**1,1**	0,417739722	No	656	888	740	905	751	1096	777	866	795	putative membrane protein	SAUSA300_1998
SAUSA300_2075	**1,1**	0,057167487	No	8782	8153	8602	11712	11916	11488	10221	11419	9466	transcription termination factor Rho	rho
SAUSA300_0288	**1,1**	0,383889662	No	923	976	1103	1717	1564	2412	1719	1572	1752	conserved hypothetical protein	SAUSA300_0288
SAUSA300_0399	**1,1**	0,76820128	No	3	4	13	13	11	18	9	16	11	exotoxin	SAUSA300_0399
SAUSA300_2372	**1,1**	0,686018368	No	22	31	46	32	50	33	34	41	23	adenosylmethionine-8-amino-7-oxononanoate transam	bioA
SAUSA300_0407	**1,1**	0,709198471	No	88	22	48	93	110	81	92	92	66	exotoxin	SAUSA300_0407

SAUSA300_0179	**1,1**	0,558137283	No	186	193	142	238	151	195	163	161	197	putative D-isomer specific 2-hydroxyacid dehydrogenas	SAUSA300_0179
SAUSA300_1207	**1,1**	0,646713647	No	36	71	50	43	43	38	34	42	34	conserved hypothetical protein	SAUSA300_1207
SAUSA300_1064	**1,1**	0,50466953	No	315	363	298	241	210	281	239	228	186	transporter, TRAP family	SAUSA300_1064
SAUSA300_2013	**1,1**	0,578715558	No	157	222	177	89	63	75	74	71	59	3-isopropylmalate dehydratase, small subunit	leuD
SAUSA300_0573	**1,1**	0,357820289	No	1479	1441	1266	1766	1690	2118	1728	1661	1567	diphosphomevalonate decarboxylase	mvaD
SAUSA300_0388	**1,1**	0,404328161	No	32737	42659	36761	19756	20294	22964	18814	19076	18092	inosine-5'-monophosphate dehydrogenase	guaB
SAUSA300_2458	**1,1**	0,699416916	No	7	30	23	27	23	40	28	27	26	glyoxylase family protein	SAUSA300_2458
SAUSA300_0523	**1,1**	0,236228819	No	22266	20411	21527	22283	24392	24379	20759	22799	19690	ribosomal protein L1	rplA
SAUSA300_2381	**1,1**	0,369471181	No	1123	1012	765	1707	1648	1624	1574	1542	1315	conserved hypothetical protein	SAUSA300_2381
SAUSA300_0458	**1,1**	0,223751272	No	276	327	289	398	419	373	363	356	338	Orn/Lys/Arg decarboxylase	SAUSA300_0458
SAUSA300_0647	**1,1**	0,241189278	No	756	679	648	733	676	702	723	554	608	ABC transporter, ATP-binding protein	SAUSA300_0647
SAUSA300_1144	**1,1**	0,333438279	No	6092	6863	6189	7783	8835	8457	7291	7661	7377	glucose inhibited division protein	gid
SAUSA300_2270	**1,1**	0,385277783	No	344	313	305	294	291	331	266	319	233	PTS system, arbutin-like IIBC component	glvC
SAUSA300_2107	**1,1**	0,525042197	No	580	517	445	295	259	395	303	286	261	PTS system, mannitol specific IIA component	mtlA
SAUSA300_2232	**1,1**	0,142663963	No	565	661	625	517	532	550	490	468	468	acetyltransferase, GNAT family	SAUSA300_2232
SAUSA300_2086	**1,1**	0,400003094	No	1383	1855	1765	1484	1562	1831	1478	1517	1351	conserved hypothetical protein	SAUSA300_2086
SAUSA300_1014	**1,1**	0,314421918	No	10542	14029	9697	4308	3931	4667	3804	3995	3713	pyruvate carboxylase	pyc
SAUSA300_0712	**1,1**	0,433008526	No	14137	17684	15467	9031	9782	9476	8819	9584	6806	amino acid/peptide transporter (Peptide:H+ symporter)	SAUSA300_0712
SAUSA300_0311	**1,1**	0,795438168	No	9	8	12	10	23	10	9	12	15	PfkB family carbohydrate kinase	SAUSA300_0311
SAUSA300_0186	**1,1**	0,442965718	No	398	494	326	341	340	333	317	307	280	N-acetyl-gamma-glutamyl-phosphate reductase	argC
SAUSA300_1417	**1,1**	0,872885859	No	0	0	1	3	0	4	2	2	2	phiSLT ORF 175-like protein	SAUSA300_1417
SAUSA300_0706	**1,1**	0,127981804	No	2067	1848	1695	3166	3043	2974	2853	2670	2698	putative osmoprotectant ABC transporter, ATP-binding	SAUSA300_0706
SAUSA300_0254	**1,1**	0,309471145	No	1516	1758	1537	1252	1028	1146	961	1073	1038	sensor histidine kinase	SAUSA300_0254
SAUSA300_0547	**1,1**	0,648677292	No	5887	9912	11563	9251	8732	9307	6503	9323	8554	sdrD protein	sdrD
SAUSA300_2499	**1,1**	0,603741588	No	179	164	162	169	169	229	159	155	196	squalene desaturase	crtM
SAUSA300_1415	**1,1**	0,885315633	No	0	1	1	3	2	0	2	1	0	phiSLT ORF 77-like protein	SAUSA300_1415
SAUSA300_1624	**1,1**	0,344192426	No	629	902	895	1015	1089	1116	991	969	933	MutT/nudix family protein	SAUSA300_1624
SAUSA300_2470	**1,1**	0,587557662	No	69	70	88	168	207	234	175	171	199	L-serine dehydratase, iron-sulfur-dependent, beta subu	sdaAB
SAUSA300_1600	**1,1**	0,325839682	No	1989	2438	2617	2529	2698	2779	2401	2489	2318	GTP-binding protein Obg/CgtA	SAUSA300_1600
SAUSA300_1531	**1,1**	0,125053428	No	1474	1484	1362	1605	1589	1557	1414	1496	1374	phosphate starvation-induced protein, PhoH family	phoH
SAUSA300_0014	**1,1**	0,193390747	No	10692	10982	9670	15620	16738	16892	14910	15533	13961	DHH subfamily 1 protein	SAUSA300_0014
SAUSA300_0115	**1,1**	0,615441509	No	2349	2007	1950	1044	1014	1217	1029	986	935	iron compound ABC transporter, permease protein SirC	sirC
SAUSA300_1203	**1,1**	NA	NA	0	1	11	3	5	1	1	0	6	conserved hypothetical protein	SAUSA300_1203
SAUSA300_2577	**1,1**	0,291080451	No	934	965	823	713	791	736	635	747	637	mannose-6-phosphate isomerase, class I	manA
SAUSA300_2429	**1,1**	0,467440301	No	145	192	174	198	178	197	187	155	176	staphylococcal tandem lipoprotein	SAUSA300_2429
SAUSA300_0319	**1,1**	0,733143252	No	41	44	28	23	23	24	25	16	21	putative membrane protein	SAUSA300_0319
SAUSA300_2077	**1,1**	0,357820289	No	2950	2319	1968	3262	2966	3104	2846	2658	2941	conserved hypothetical protein	SAUSA300_2077
SAUSA300_2173	**1,1**	0,332285343	No	1834	1785	1572	1499	1571	1612	1463	1458	1317	tRNA pseudouridine synthase A	truA
SAUSA300_2004	**1,1**	0,396840928	No	961	957	853	1209	1080	1168	1107	976	1048	conserved hypothetical protein	SAUSA300_2004
SAUSA300_0689	**1,1**	0,328606979	No	1671	1597	1486	1800	1609	1525	1525	1424	1519	glycosyl transferase, group 2 family protein	SAUSA300_0689
SAUSA300_0602	**1,1**	0,387355279	No	5670	4327	5418	4332	4186	4217	4018	4146	3368	conserved hypothetical protein	SAUSA300_0602
SAUSA300_0533	**1,1**	0,349278464	No	153518	161072	133150	197108	199577	227855	185546	209493	170522	translation elongation factor Tu	tuf
SAUSA300_0993	**1,1**	0,143672316	No	32592	37809	33790	30571	29226	32487	27485	29130	27024	pyruvate dehydrogenase E1 component, alpha subunit	pdhA
SAUSA300_0848	**1,1**	0,697970865	No	69	56	46	64	56	64	44	78	46	conserved hypothetical protein	SAUSA300_0848
SAUSA300_0750	**1,1**	0,166291182	No	2585	2497	2328	2824	2687	2610	2589	2525	2264	conserved hypothetical protein	SAUSA300_0750
SAUSA300_2308	**1,1**	0,476334621	No	203	287	194	301	264	312	259	291	249	response regulator protein	SAUSA300_2308
SAUSA300_0204	**1,1**	0,297713853	No	692	790	638	610	624	590	558	548	550	gamma-glutamyltranspeptidase	ggt
SAUSA300_0009	**1,1**	0,295015605	No	4795	4658	4177	4418	4323	4160	3900	4358	3473	seryl-tRNA synthetase	serS
SAUSA300_1747	**1,1**	0,64489101	No	17	40	62	84	83	120	86	82	95	conserved hypothetical protein	SAUSA300_1747
SAUSA300_2468	**1,1**	0,537441507	No	145	172	134	232	221	291	262	215	203	acetyltransferase, GNAT family	SAUSA300_2468
SAUSA300_2576	**1,1**	0,53893419	No	1345	1470	1280	924	1041	1062	935	1025	795	phosphotransferase system, fructose-specific IIABC com	SAUSA300_2576
SAUSA300_1667	**1,1**	0,563410587	No	153	240	218	341	329	300	295	288	300	putative glycerophosphoryl diester phosphodiesterase	SAUSA300_1667
SAUSA300_0883	**1,1**	0,585473824	No	295	327	235	504	500	543	519	416	475	putative surface protein	SAUSA300_0883
SAUSA300_0643	**1,1**	0,258416227	No	1738	1495	1408	2538	2389	2397	2279	2323	2078	acetyltransferase, GNAT family	SAUSA300_0643
SAUSA300_0856	**1,1**	0,497959603	No	229	249	215	243	248	265	244	244	203	conserved hypothetical protein	SAUSA300_0856
SAUSA300_1898	**1,1**	0,622497413	No	241	389	298	271	246	333	241	281	254	conserved hypothetical protein	SAUSA300_1898
SAUSA300_0575	**1,1**	0,548101651	No	665	637	510	726	755	837	877	636	601	conserved hypothetical protein	SAUSA300_0575
SAUSA300_1536	**1,1**	0,332092897	No	5346	6587	5468	5105	5657	5108	4880	4859	4736	conserved hypothetical protein	SAUSA300_1536
SAUSA300_1345	**1,1**	0,359344868	No	10644	11439	8585	7935	7569	7587	7227	7254	6590	asparaginyl-tRNA synthetase	asnS

SAUSA300_2276	**1,1**	0,615908948	No	184	335	256	186	178	225	180	182	177	peptidase, M20/M25/M40 family	SAUSA300_2276
SAUSA300_0253	**1,1**	0,52681489	No	241	189	204	206	216	209	180	191	203	ScdA protein	scdA
SAUSA300_2162	**1,1**	0,53204866	No	298	420	425	755	669	726	652	649	663	M23/M37 peptidase domain protein	SAUSA300_2162
SAUSA300_0919	**1,1**	0,443499821	No	1869	2334	2394	2522	2585	2810	2357	2454	2417	UDP-N-acetylmuramoylalanyl-D-glutamate--2, 6-diamin	murE
SAUSA300_1476	**1,1**	0,27951412	No	2840	3110	2736	3116	3022	3410	2994	2948	2785	acetyl-CoA carboxylase, biotin carboxyl carrier protein	accB
SAUSA300_1196	**1,1**	0,306067428	No	1061	861	821	785	764	797	744	741	662	RNA chaperone, host factor-1 protein	hfq
SAUSA300_1543	**1,1**	0,372089475	No	338	381	359	448	469	426	423	385	415	oxygen-independent coproporphyrinogen III oxidase	SAUSA300_1543
SAUSA300_1768	**1,1**	0,796081377	No	26	27	34	14	14	22	12	16	18	leukotoxin LukD	lukD
SAUSA300_1059	**1,1**	0,831875222	No	17	16	30	17	11	22	19	11	16	putative exotoxin 1	SAUSA300_1059
SAUSA300_0265	**1,1**	0,578336532	No	587	600	463	394	417	528	410	457	358	putative ribose operon repressor	SAUSA300_0265
SAUSA300_0362	**1,1**	0,563764917	No	198	339	317	358	462	470	398	398	382	conserved hypothetical protein	SAUSA300_0362
SAUSA300_2260	**1,1**	0,535988689	No	146	160	237	296	313	331	261	310	290	inositol monophosphatase family protein	SAUSA300_2260
SAUSA300_2259	**1,1**	0,493617058	No	587	735	671	947	976	1158	963	903	955	putative transcriptional regulator	SAUSA300_2259
SAUSA300_2256	**1,1**	0,556608772	No	3162	3707	2807	2284	2385	2484	2276	2362	1911	putative N-acetylmuramoyl-L-alanine amidase	SAUSA300_2256
SAUSA300_1690	**1,1**	0,313819674	No	973	895	770	779	755	713	739	684	639	putative thioredoxin	SAUSA300_1690
SAUSA300_0285	**1,1**	0,656701502	No	171	136	169	239	230	313	269	207	243	conserved hypothetical protein	SAUSA300_0285
SAUSA300_0828	**1,1**	0,204326126	No	1283	1359	1244	2061	2189	1868	1895	1893	1812	5'-nucleotidase family protein	SAUSA300_0828
SAUSA300_0915	**1,1**	0,557349723	No	288	375	291	261	241	336	283	246	243	conserved hypothetical protein	SAUSA300_0915
SAUSA300_1157	**1,1**	0,293013974	No	8660	9002	7809	9508	9683	9837	8988	8937	8697	DNA polymerase III, alpha subunit	polC
SAUSA300_0410	**1,1**	0,743096682	No	12	12	12	29	23	43	26	36	27	staphylococcal tandem lipoprotein	SAUSA300_0410
SAUSA300_2021	**1,1**	0,082236465	No	3117	3263	2897	2582	2723	2614	2409	2475	2377	S1 RNA binding domain protein	SAUSA300_2021
SAUSA300_2508	**1,1**	0,888620952	No	0	1	4	0	2	0	1	1	0	conserved hypothetical protein	SAUSA300_2508
SAUSA300_1475	**1,1**	0,242212216	No	6149	7278	6306	7597	7873	8179	7315	7520	6870	acetyl-CoA carboxylase, biotin carboxylase	accC
SAUSA300_1758	**1,1**	0,827255327	No	22	25	16	19	9	10	11	12	13	serine protease SplA	splA
SAUSA300_1568	**1,1**	0,213163375	No	3675	3374	3448	3645	3762	3417	3234	3407	3294	uridine kinase	udk
SAUSA300_0478	**1,1**	0,280385026	No	6349	6572	5502	7455	7551	8333	7359	7371	6720	ribose-phosphate pyrophosphokinase	prs
SAUSA300_0077	**1,1**	0,531984888	No	229	196	182	299	327	278	276	284	268	ABC transporter, ATP-binding protein	SAUSA300_0077
SAUSA300_1360	**1,1**	0,404447893	No	1299	1508	1644	1509	1470	1667	1417	1408	1447	menaquinone biosynthesis methyltransferase ubiE	ubiE
SAUSA300_2268	**1,1**	0,563892021	No	322	350	301	200	221	225	165	233	194	sodium/bile acid symporter family protein	SAUSA300_2268
SAUSA300_2358	**1,1**	0,128292624	No	12093	12458	10881	9095	9072	9418	8387	8742	8247	ABC transporter, permease protein	SAUSA300_2358
SAUSA300_2288	**1,1**	0,317835678	No	1056	1120	954	939	881	933	831	862	844	ABC transporter, ATP-binding protein	SAUSA300_2288
SAUSA300_2174	**1,1**	0,333311571	No	1002	1047	1016	789	893	827	790	745	767	cobalt transport family protein	SAUSA300_2174
SAUSA300_2433	**1,1**	0,404328161	No	1306	1406	1147	1939	1661	1652	1627	1592	1621	phosphoglucomutase/phosphomannomutase family pro	SAUSA300_2433
SAUSA300_0697	**1,1**	0,578715558	No	501	582	491	417	570	439	413	494	400	exsB protein	SAUSA300_0697
SAUSA300_2038	**1,1**	0,336699918	No	4203	4922	4297	4690	4983	5019	4522	4880	4139	UDP-N-acetylmuramoyl-tripeptide--D-alanyl-D- alanine	murF
SAUSA300_0908	**1,1**	0,3775698	No	2788	3243	2388	3165	2926	3196	2893	2937	2738	NAD(+)/NADH kinase	SAUSA300_0908
SAUSA300_2130	**1,1**	0,442965718	No	1326	1366	1817	2204	2360	2165	1992	2106	2103	UTP-glucose-1-phosphate uridylyltransferase family pro	SAUSA300_2130
SAUSA300_0003	**1,1**	0,339579416	No	927	875	916	985	962	976	929	973	799	conserved hypothetical protein	SAUSA300_0003
SAUSA300_0450	**1,1**	0,529886027	No	239	292	335	490	451	513	405	518	420	trehalose operon repressor	treR
SAUSA300_1527	**1,1**	0,359679345	No	1268	1560	1582	1608	1733	1686	1483	1514	1642	GTP-binding protein Era	era
SAUSA300_1181	**1,1**	0,291447167	No	2160	2141	2028	1372	1377	1508	1320	1344	1272	conserved hypothetical protein	SAUSA300_1181
SAUSA300_2163	**1,1**	0,631239242	No	140	198	272	355	336	359	302	326	343	conserved hypothetical protein	SAUSA300_2163
SAUSA300_2471	**1,1**	0,648677292	No	324	285	277	510	651	543	527	489	552	perfringolysin O regulator protein	SAUSA300_2471
SAUSA300_0857	**1,1**	0,370098114	No	2677	3154	2783	3848	3597	4055	3367	3763	3502	conserved hypothetical protein	SAUSA300_0857
SAUSA300_0928	**1,1**	0,741699256	No	146	136	147	50	43	37	42	35	44	competence transcription factor	comK
SAUSA300_2625	**1,1**	0,781398012	No	17	70	60	105	65	114	92	84	89	transcriptional regulator, PadR family	SAUSA300_2625
SAUSA300_0356	**1,1**	0,475742187	No	670	684	623	497	478	519	481	493	412	conserved hypothetical protein	SAUSA300_0356
SAUSA300_1122	**1,1**	0,297248459	No	3491	3829	4298	6155	6460	6282	5865	6087	5541	fatty acid/phospholipid synthesis protein PlsX	plsX
SAUSA300_1143	**1,1**	0,549433772	No	8360	10372	8861	11639	13465	13196	12039	11825	11620	DNA topoisomerase I	topA
SAUSA300_1867	**1,1**	0,306736948	No	708	691	630	826	908	788	805	770	756	conserved hypothetical protein	SAUSA300_1867
SAUSA300_2571	**1,1**	0,838609884	No	28	33	23	27	14	16	23	12	19	arginine repressor	argR
SAUSA300_1323	**1,1**	0,624031316	No	475	701	534	647	660	512	607	577	500	conserved hypothetical protein	SAUSA300_1323
SAUSA300_2589	**1,1**	0,385277783	No	27981	24308	23675	58582	56215	56054	47860	54899	55728	LPXTG-motif cell wall surface anchor family protein	SAUSA300_2589
SAUSA300_1639	**1,1**	0,543594874	No	1025	1280	981	1128	960	1063	958	1038	933	alkaline phosphatase synthesis transcriptional regulator	phoP
SAUSA300_1591	**1,1**	0,486404421	No	1810	1744	1633	1232	1314	1405	1246	1285	1134	adenine phosphoribosyltransferase	apt
SAUSA300_2183	**1,1**	0,572714921	No	9358	11379	10727	14849	15703	17707	15360	15872	13533	adenylate kinase	adk
SAUSA300_0240	**1,1**	0,887031032	No	2	5	5	6	9	7	5	6	8	PTS system, galactitol-specific enzyme II, B component	SAUSA300_0240
SAUSA300_2424	**1,1**	0,602096634	No	146	145	167	122	142	140	127	117	129	putative staphylococcal tandem lipoprotein	SAUSA300_2424
SAUSA300_1670	**1,1**	0,499730772	No	1545	1815	1269	748	811	662	723	673	662	D-3-phosphoglycerate dehydrogenase	serA

SAUSA300_0330	**1,1**	0,654537268	No	96	162	111	152	122	135	138	128	117	putative transport protein SgaT	SAUSA300_0330
SAUSA300_0418	**1,1**	0,713643585	No	47	88	75	139	119	127	131	108	121	staphylococcal tandem lipoprotein	SAUSA300_0418
SAUSA300_0451	**1,1**	0,630739431	No	112	155	167	126	128	161	126	125	136	acetyltransferase, GNAT family	SAUSA300_0451
SAUSA300_0167	**1,1**	0,465306395	No	649	652	543	740	778	818	762	727	685	capsular polysaccharide biosynthesis protein Cap5P	cap5P
SAUSA300_0241	**1,1**	0,79228994	No	19	38	35	42	41	50	41	45	38	PTS system, sorbitol-specific IIC component	SAUSA300_0241
SAUSA300_2512	**1,1**	0,632279898	No	90	88	98	160	144	154	143	133	151	glyoxalase family protein	SAUSA300_2512
SAUSA300_1147	**1,1**	0,476597835	No	3293	4027	4417	6944	7005	7431	5935	7068	6913	heat shock protein HslVU, ATPase subunit HslU	hslU
SAUSA300_0979	**1,1**	0,710535355	No	834	947	722	483	433	442	464	456	344	conserved hypothetical protein	SAUSA300_0979
SAUSA300_1355	**1,1**	0,438995843	No	2868	3270	2625	2187	2445	2202	2161	2073	2130	3-phosphoshikimate 1-carboxyvinyltransferase	aroA
SAUSA300_1367	**1,1**	0,285363999	No	2198	2219	2006	1829	1699	1826	1721	1682	1601	cytidylate kinase	cmk
SAUSA300_1015	**1,1**	0,728826368	No	1736	1092	1513	1333	1192	1177	1094	1258	1101	cytochrome oxidase assembly protein	ctaA
SAUSA300_0560	**1,1**	0,695783991	No	312	361	286	255	223	355	243	264	274	acetyl-CoA c-acetyltransferase	vraB
SAUSA300_2583	**1,1**	0,551575516	No	1061	1076	1371	1968	2110	2348	1956	1978	2065	putative glycosyl transferase	SAUSA300_2583
SAUSA300_1880	**1,1**	0,309471145	No	17201	18050	16108	18259	19219	18007	17556	17824	16440	Aspartyl/glutamyl-tRNA amidotransferase subunit B	gatB
SAUSA300_0479	**1,1**	0,586415797	No	38088	35872	30407	38424	39040	43148	39750	40767	32161	ribosomal protein L25, Ctc-form	SAUSA300_0479
SAUSA300_1086	**1,1**	0,219992057	No	5055	4814	4619	3968	4021	4139	3744	3728	3871	putative cell-division initiation protein	SAUSA300_1086
SAUSA300_0569	**1,1**	0,567592983	No	3989	3488	3220	4687	4057	3882	4143	3899	3772	conserved hypothetical protein	SAUSA300_0569
SAUSA300_0371	**1,1**	0,898042315	No	16	14	8	4	16	9	9	7	9	conserved hypothetical protein	SAUSA300_0371
SAUSA300_0443	**1,1**	0,483793242	No	1354	1278	1307	2327	2542	2291	2224	2277	2192	YibE/F-like protein	SAUSA300_0443
SAUSA300_0934	**1,1**	0,801966558	No	45	63	56	27	36	35	32	29	30	membrane protein	SAUSA300_0934
SAUSA300_1887	**1,1**	0,382285542	No	701	883	802	1004	1055	945	961	954	895	geranylgeranylglyceryl phosphate synthase family prote	pcrB
SAUSA300_2197	**1,1**	0,743096682	No	6343	8510	9169	10712	10961	14125	11688	12237	9576	50S ribosomal protein L16	rplP
SAUSA300_1459	**1,1**	0,135830864	No	12268	12844	12784	12917	12345	12800	11983	11943	11757	6-phosphogluconate dehydrogenase, decarboxylating	gnd
SAUSA300_1799	**1,1**	0,457382066	No	818	840	718	662	712	766	685	660	664	putative sensor histidine kinase	SAUSA300_1799
SAUSA300_1534	**1,1**	0,420333985	No	5131	5140	5202	5024	5337	5031	4658	4923	4863	conserved hypothetical protein	SAUSA300_1534
SAUSA300_0861	**1,1**	0,575402645	No	1436	1619	1479	2046	1706	2261	1784	2098	1775	NAD-specific glutamate dehydrogenase	gudB
SAUSA300_0537	**1,1**	0,685918749	No	386	434	378	375	437	356	368	361	366	L-ribulokinase	SAUSA300_0537
SAUSA300_0408	**1,1**	0,775084369	No	406	208	188	485	532	393	449	478	396	putative surface protein	SAUSA300_0408
SAUSA300_0628	**1,1**	0,575788321	No	289	412	414	858	886	855	788	887	771	teichoic acid biosynthesis protein D	SAUSA300_0628
SAUSA300_0886	**1,1**	0,579253127	No	7156	9293	9561	12946	13438	15048	13165	13874	11998	3-oxoacyl-(acyl-carrier-protein) synthase II	fabF
SAUSA300_1198	**1,1**	0,633909117	No	530	531	567	455	595	445	495	424	480	putative GTP-binding protein	SAUSA300_1198
SAUSA300_2480	**1,1**	0,617386237	No	834	564	572	1153	1039	994	1031	979	995	transcriptional regulator, LysR family	SAUSA300_2480
SAUSA300_0669	**1,1**	0,511788908	No	4886	5503	4392	5787	5734	5520	5415	5748	4922	undecaprenol kinase	SAUSA300_0669
SAUSA300_1009	**1,1**	0,660290411	No	9531	6987	8244	11518	13411	11101	11528	11959	10524	GTP-binding protein	typA
SAUSA300_2265	**1,1**	0,682945208	No	1748	2194	2599	1784	1805	1887	1692	1888	1592	putative amino acid permease	SAUSA300_2265
SAUSA300_2301	**1,1**	0,712592321	No	291	379	511	455	440	418	428	397	416	teicoplanin resistance associated membrane protein Tc	tcaB
SAUSA300_1336	**1,1**	0,463715464	No	1233	1184	1174	1697	1733	1736	1673	1622	1589	conserved hypothetical protein	SAUSA300_1336
SAUSA300_1133	**1,1**	0,666645325	No	937	1007	852	1005	1023	1193	1037	1041	969	tRNA (guanine-N1)-methyltransferase	trmD
SAUSA300_2084	**1,1**	0,597392065	No	360	411	482	980	978	1106	934	1048	918	pantothenate kinase	coaA
SAUSA300_1229	**1,1**	0,483793242	No	3026	3103	3071	2294	2096	1878	1996	2000	1933	hydrolase, haloacid dehalogenase-like family	SAUSA300_1229
SAUSA300_0847	**1,1**	0,656932661	No	1149	871	819	591	568	535	543	563	497	conserved hypothetical protein	SAUSA300_0847
SAUSA300_0592	**1,1**	0,448412839	No	2453	2829	2703	2661	2657	2579	2582	2442	2447	conserved hypothetical protein	SAUSA300_0592
SAUSA300_1316	**1,1**	0,708547079	No	1674	1860	1490	1614	1526	1261	1461	1408	1294	methionine-R-sulfoxide reductase	msrB
SAUSA300_0239	**1,1**	0,918387146	No	5	10	5	7	14	3	7	8	5	PTS system, fructose-specific enzyme II, BC component	SAUSA300_0239
SAUSA300_2054	**1,1**	0,608318058	No	2491	2530	2462	2056	2132	2295	2099	2134	1905	(3R)-hydroxymyristoyl-[acyl carrier protein	fabZ
SAUSA300_1356	**1,1**	0,581204014	No	863	1095	1048	649	760	689	635	659	684	3-dehydroquinate synthase	aroB
SAUSA300_2412	**1,1**	0,780988761	No	84	89	74	79	72	64	61	69	72	conserved hypothetical protein	SAUSA300_2412
SAUSA300_2529	**1,1**	0,742897812	No	2127	1261	1612	4281	4064	3737	4249	4049	3139	conserved hypothetical protein	SAUSA300_2529
SAUSA300_0702	**1,1**	0,664952702	No	1886	2594	1784	1577	1571	1508	1521	1446	1442	urea amidolyase-related protein	SAUSA300_0702
SAUSA300_1431	**1,1**	0,943849397	No	0	0	1	1	2	1	2	1	1	phiSLT ORF71-like protein	SAUSA300_1431
SAUSA300_0911	**1,1**	0,420333985	No	1674	1666	1754	2029	1977	1971	1986	1837	1850	transporter, monovalent cation:proton antiporter-2 (CP	SAUSA300_0911
SAUSA300_2225	**1,1**	0,737351631	No	1128	997	840	566	566	719	636	565	556	molybdenum cofactor biosynthesis protein C	moaC
SAUSA300_1376	**1,1**	0,708362663	No	96	97	105	113	95	121	108	106	102	putative lipoprotein	SAUSA300_1376
SAUSA300_1617	**1,1**	0,460227669	No	3279	3330	2856	2867	2500	2607	2485	2585	2508	porphobilinogen deaminase	hemC
SAUSA300_0389	**1,1**	0,723946882	No	43813	50631	43823	24659	25604	27284	25903	26283	21454	GMP synthase	guaA
SAUSA300_2149	**1,1**	0,794475733	No	171	171	95	175	180	201	193	149	185	6-phospho-beta-galactosidase	lacG
SAUSA300_1971	**1,1**	0,853993665	No	176	203	120	105	83	102	128	78	72	phi77 ORF017-like protein	SAUSA300_1971
SAUSA300_2385	**1,1**	0,674531058	No	3836	4124	3651	2626	2479	2545	2324	2379	2568	putative membrane protein	SAUSA300_2385
SAUSA300_0060	**1,1**	0,855217829	No	50	23	35	40	43	37	42	31	39	putative transposase	SAUSA300_0060

SAUSA300_1583	**1,1**	0,70797661	No	1512	1372	1423	2194	2001	2147	2172	2146	1713	conserved hypothetical protein	SAUSA300_1583
SAUSA300_2179	**1,1**	0,68521304	No	6605	7705	6717	9267	9309	10189	9382	9741	8222	30S ribosomal protein S11	rpsK
SAUSA300_2114	**1,1**	0,723946882	No	103	155	126	223	207	188	197	217	177	arginase	rocF
SAUSA300_0563	**1,1**	0,58936833	No	582	591	664	490	500	574	492	481	516	uracil-DNA glycosylase	ung
SAUSA300_1026	**1,0**	0,608318058	No	14202	11234	11873	14183	14560	13887	14017	13833	12759	conserved hypothetical protein	SAUSA300_1026
SAUSA300_1045	**1,0**	0,687467686	No	696	834	644	884	764	902	852	772	812	excinuclease ABC, C subunit	uvrC
SAUSA300_0590	**1,0**	0,616611811	No	1349	1331	1159	1660	1479	1625	1521	1626	1400	conserved hypothetical protein	SAUSA300_0590
SAUSA300_1454	**1,0**	0,582536901	No	8970	8837	7426	9109	8756	8229	8452	7746	8665	glucose-6-phosphate 1-dehydrogenase	zwf
SAUSA300_2339	**1,0**	0,666222284	No	806	909	672	524	550	501	493	517	489	conserved hypothetical protein	SAUSA300_2339
SAUSA300_1552	**1,0**	0,65282889	No	1650	1914	1801	1643	1684	1622	1548	1615	1552	conserved hypothetical protein	SAUSA300_1552
SAUSA300_1521	**1,0**	0,583868107	No	7997	6935	6389	5574	5876	5467	5571	5428	5146	RNA polymerase sigma factor RpoD	rpoD
SAUSA300_0692	**1,0**	0,818401552	No	5320	3055	2422	3901	3868	3385	3590	3308	3748	conserved hypothetical protein	SAUSA300_0692
SAUSA300_0869	**1,0**	0,564545787	No	1767	2012	1918	2102	2236	2047	2014	1956	2123	exonuclease RexB	rexB
SAUSA300_2057	**1,0**	0,580771348	No	7892	9023	7710	10582	10893	11349	10793	11007	9589	ATP synthase F1, epsilon subunit	atpC
SAUSA300_0958	**1,0**	0,630378134	No	7127	5972	6199	8822	10602	8770	8982	8943	9023	transcriptional regulator	SAUSA300_0958
SAUSA300_1227	**1,0**	0,723946882	No	1533	1778	1670	886	771	797	719	828	802	threonine synthase	thrC
SAUSA300_0505	**1,0**	0,781398012	No	1728	2541	2081	2001	2026	2081	2414	1868	1564	conserved hypothetical protein	SAUSA300_0505
SAUSA300_0834	**1,0**	0,475742187	No	2013	2179	2067	2565	2353	2447	2307	2458	2296	D-isomer specific 2-hydroxyacid dehydrogenase	SAUSA300_0834
SAUSA300_0947	**1,0**	0,680658691	No	1219	1058	1316	1678	1661	1532	1499	1595	1566	hydrolase, alpha/beta hydrolase fold family	SAUSA300_0947
SAUSA300_1302	**1,0**	0,712239242	No	1107	1295	1043	1217	1244	1072	1151	1090	1137	ATPase family protein	SAUSA300_1302
SAUSA300_0670	**1,0**	0,816445408	No	744	704	778	594	611	634	588	621	553	ABC transporter, ATP-binding protein, MsbA family	SAUSA300_0670
SAUSA300_2467	**1,0**	0,610279391	No	2180	1826	1985	3023	2901	2614	2825	2708	2646	sortase	srtA
SAUSA300_2039	**1,0**	0,545354598	No	3980	4294	4075	4351	4287	4265	4071	4363	3934	D-alanine--D-alanine ligase	ddl
SAUSA300_1142	**1,0**	0,840671424	No	60	57	64	54	52	58	55	51	52	DNA protecting protein DprA	dprA
SAUSA300_0252	**1,0**	0,464215764	No	9509	9260	9173	16014	17342	16383	15819	15976	15892	glycosyl transferase, group 2 family protein	SAUSA300_0252
SAUSA300_1850	**1,0**	0,697970865	No	1080	908	1005	1525	1560	1514	1401	1469	1538	conserved hypothetical protein	SAUSA300_1850
SAUSA300_2187	**1,0**	0,810926632	No	8872	11309	10511	13928	14213	17290	14849	15787	12936	30S ribosomal protein S5	rpsE
SAUSA300_1866	**1,0**	0,659927245	No	2323	2294	2100	2525	2626	2215	2375	2285	2400	two-component sensor histidine kinase	vraS
SAUSA300_1651	**1,0**	0,564787653	No	1300	1285	1272	1243	1215	1193	1189	1273	1047	CBS domain protein	SAUSA300_1651
SAUSA300_0306	**1,0**	0,803388684	No	3686	2762	3269	3330	3751	3101	3172	3401	3200	branched-chain amino acid transport system II carrier p	brnQ
SAUSA300_1036	**1,0**	0,691203152	No	673	697	827	569	563	631	511	613	573	RNA methyltransferase, TrmH family	SAUSA300_1036
SAUSA300_0035	**1,0**	0,692108502	No	5577	4972	4604	5435	5955	6064	5482	6380	4913	truncated hypothetical protein	SAUSA300_0035
SAUSA300_2063	**1,0**	0,695783991	No	3443	4038	3883	4457	4758	5659	4604	4857	4836	ATP synthase F0, C subunit	atpE
SAUSA300_0950	**1,0**	0,796081377	No	792	617	613	282	230	214	211	244	247	cysteine protease precursor	sspB
SAUSA300_1573	**1,0**	0,615441509	No	3374	3110	3050	3234	3002	2952	3079	2944	2835	conserved hypothetical protein TIGR00250	SAUSA300_1573
SAUSA300_0294	**1,0**	0,930181584	No	9	14	12	13	7	25	16	14	16	conserved hypothetical protein	SAUSA300_0294
SAUSA300_1278	**1,0**	0,682657565	No	5749	5315	4656	5677	5186	4773	5157	4785	5134	oligoendopeptidase F	pepF
SAUSA300_2010	**1,0**	0,843529105	No	422	572	464	156	165	155	168	160	132	2-isopropylmalate synthase	leuA
SAUSA300_1751	**1,0**	0,763281074	No	608	795	638	510	426	525	498	474	446	type I restriction-modification enzyme, S subunit	hsdS
SAUSA300_2619	**1,0**	0,905839417	No	65	131	109	39	47	52	42	36	53	conserved hypothetical protein	SAUSA300_2619
SAUSA300_2228	**1,0**	0,622754584	No	632	682	681	594	568	611	564	598	556	molybdenum ABC transporter, ATP-binding protein Moc	modC
SAUSA300_2083	**1,0**	0,710535355	No	343	327	385	735	627	660	658	635	666	acetyltransferase, GNAT family	SAUSA300_2083
SAUSA300_0674	**1,0**	0,681090984	No	1411	1278	1379	1668	1720	1693	1638	1729	1541	oxidoreductase, aldo/keto reductase family	SAUSA300_0674
SAUSA300_0764	**1,0**	0,620856194	No	5172	5573	5722	5959	6237	5949	5718	6275	5536	ribonuclease R	rnr
SAUSA300_0895	**1,0**	0,936953745	No	9	10	11	14	9	15	11	14	12	oligopeptide ABC transporter, permease protein	oppB
SAUSA300_2563	**1,0**	0,734583764	No	744	672	621	729	793	775	793	681	744	putative transcriptional regulator	SAUSA300_2563
SAUSA300_1707	**1,0**	0,800817015	No	1777	1645	1507	1432	1447	1164	1320	1276	1307	conserved hypothetical protein	SAUSA300_1707
SAUSA300_0023	**1,0**	0,697970865	No	2990	2583	2644	4475	4941	4386	4463	4467	4432	YycI protein	SAUSA300_0023
SAUSA300_0641	**1,0**	0,81148312	No	815	772	722	941	1007	821	853	796	1029	putative lipase/esterase	SAUSA300_0641
SAUSA300_0978	**1,0**	0,862329588	No	2206	2699	1966	1204	1156	951	1109	1173	928	ABC transporter, ATP-binding protein	SAUSA300_0978
SAUSA300_1575	**1,0**	0,743260807	No	4073	4968	4765	4478	4571	4957	4364	4861	4370	alanyl-tRNA synthetase	alaS
SAUSA300_0247	**1,0**	0,819468019	No	2447	2866	2216	1538	1535	1479	1517	1482	1419	putative teichoic acid biosynthesis protein B	SAUSA300_0247
SAUSA300_0865	**1,0**	0,776177978	No	14295	14099	11719	14534	13046	12741	14132	12954	12061	glucose-6-phosphate isomerase	pgi
SAUSA300_1688	**1,0**	0,781103623	No	810	794	764	848	998	974	934	900	901	phenylalanyl-tRNA synthetase (beta subunit)	SAUSA300_1688
SAUSA300_1979	**1,0**	0,878805964	No	389	316	341	206	273	152	205	182	219	cation transport family protein	SAUSA300_1979
SAUSA300_1551	**1,0**	0,680098307	No	1164	1235	1310	1283	1355	1355	1299	1318	1264	conserved hypothetical protein	SAUSA300_1551
SAUSA300_0234	**1,0**	0,915898425	No	3212	1756	2003	3772	2716	2641	2759	3327	2794	putative flavohemoprotein	SAUSA300_0234
SAUSA300_0554	**1,0**	0,856403635	No	415	474	400	441	397	379	428	395	363	glucosamine-6-phosphate isomerase	SAUSA300_0554
SAUSA300_2035	**1,0**	0,875362405	No	379	498	415	334	246	269	295	240	296	sensor histidine kinase, KdpD	kdpD

SAUSA300_0676	**1,0**	0,792145154	No	548	517	487	441	397	405	410	444	365	anion transporter family protein	SAUSA300_0676
SAUSA300_0084	**1,0**	0,914510292	No	53	71	72	46	77	49	57	49	56	conserved hypothetical protein	SAUSA300_0084
SAUSA300_1083	**1,0**	0,753139419	No	7468	6491	6621	6870	7188	7058	6988	7014	6591	conserved hypothetical protein	SAUSA300_1083
SAUSA300_1488	**1,0**	0,951471551	No	22	8	18	13	25	12	16	18	13	conserved hypothetical protein	SAUSA300_1488
SAUSA300_0415	**1,0**	0,944668726	No	10	15	16	23	20	25	21	21	25	staphylococcal tandem lipoprotein	lpl3
SAUSA300_2517	**1,0**	0,888910441	No	1025	1055	762	533	496	671	614	517	532	amidohydrolase family protein	SAUSA300_2517
SAUSA300_1164	**1,0**	0,849645757	No	632	725	658	730	622	627	614	666	654	tRNA pseudouridine synthase B	truB
SAUSA300_0344	**1,0**	0,942655185	No	34	82	69	29	29	43	31	29	38	putative lipoprotein	SAUSA300_0344
SAUSA300_0945	**1,0**	0,818401552	No	661	857	824	1077	1174	1125	1198	1002	1090	isochorismate synthase family protein	SAUSA300_0945
SAUSA300_0293	**1,0**	0,94957329	No	28	12	22	14	29	38	26	29	24	conserved hypothetical protein	SAUSA300_0293
SAUSA300_2439	**1,0**	0,865486187	No	1502	1840	1364	1050	1089	1109	1060	1111	1001	UTP-glucose-1-phosphate uridylyltransferase	galU
SAUSA300_1524	**1,0**	0,880744543	No	415	532	551	497	446	408	404	475	443	CBS domain pair protein	SAUSA300_1524
SAUSA300_0449	**1,0**	0,90549721	No	327	416	503	596	595	676	548	717	560	alpha,alpha-phosphotrehalase	treC
SAUSA300_1506	**1,0**	0,86513618	No	424	515	592	443	559	504	456	481	529	conserved hypothetical protein	SAUSA300_1506
SAUSA300_1528	**1,0**	0,803295639	No	605	697	713	778	751	725	726	731	748	cytidine deaminase	cdd
SAUSA300_0719	**1,0**	0,940051842	No	67	118	118	42	52	49	46	42	49	iron compound ABC transporter, permease protein	SAUSA300_0719
SAUSA300_2111	**1,0**	0,79278617	No	6521	7249	6233	8190	7650	8163	8436	7769	7309	phosphoglucosamine mutase	glmM
SAUSA300_1636	**1,0**	0,841381333	No	2980	3343	3023	3815	3460	3508	3691	3445	3429	DNA polymerase I superfamily	polA
SAUSA300_0791	**1,0**	0,865486187	No	4003	4230	4173	5050	4855	4904	5137	5081	4293	glycine cleavage system H protein	gcvH
SAUSA300_2285	**1,0**	0,860109542	No	505	663	622	461	455	427	436	431	449	aldose 1-epimerase	galM
SAUSA300_0650	**1,0**	0,816218807	No	5408	5315	4620	5398	5529	5922	5438	5814	5265	phosphate transporter family protein	SAUSA300_0650
SAUSA300_1652	**1,0**	0,923734497	No	4454	4183	3925	2887	2078	2688	2910	2375	2222	conserved hypothetical protein	SAUSA300_1652
SAUSA300_2472	**1,0**	0,905839417	No	499	706	756	531	521	593	517	576	521	putative membrane protein	SAUSA300_2472
SAUSA300_1976	**1,0**	0,930181667	No	873	1242	883	673	478	775	683	580	632	probable succinyl-diaminopimelate desuccinylase	SAUSA300_1976
SAUSA300_0901	**1,0**	0,967838701	No	17	7	15	13	16	12	14	8	16	putative competence protein	SAUSA300_0901
SAUSA300_1671	**1,0**	0,872885859	No	890	1122	1244	643	672	628	610	609	684	conserved hypothetical protein	SAUSA300_1671
SAUSA300_0850	**1,0**	0,890797784	No	587	569	554	530	579	540	560	557	498	Na(+)/H(+) antiporter subunit F	mnhF
SAUSA300_1452	**1,0**	0,906347142	No	1328	1693	1345	1243	1258	1381	1231	1281	1300	pyrroline-5-carboxylate reductase	proC
SAUSA300_1113	**1,0**	0,842401966	No	5057	5863	5880	4634	4812	4621	4499	4561	4747	protein kinase	pknB
SAUSA300_2449	**1,0**	0,930181667	No	536	757	648	478	503	555	468	649	391	putative transporter	SAUSA300_2449
SAUSA300_2283	**1,0**	0,872284732	No	768	953	855	579	541	637	575	611	548	ribose 5-phosphate isomerase A	rpiA
SAUSA300_2062	**1,0**	0,853050799	No	5908	6885	6441	7547	7614	8210	7640	7875	7459	ATP synthase F0, B subunit	atpF
SAUSA300_1159	**1,0**	0,76820128	No	3613	3721	3414	4003	4282	4161	4150	4105	3972	transcription termination factor NusA	nusA
SAUSA300_0042	**1,0**	0,871775119	No	1188	1140	1226	1434	1415	1406	1440	1339	1406	conserved hypothetical protein	SAUSA300_0042
SAUSA300_2060	**1,0**	0,84269818	No	18164	21075	19358	23061	23776	24117	23179	24537	22102	ATP synthase F1, alpha subunit	atpA
SAUSA300_1002	**1,0**	0,918387146	No	1497	1484	1273	1107	886	1176	1082	969	1074	spermidine/putrescine ABC transporter, spermidine/pu	potD
SAUSA300_0694	**1,0**	0,966217197	No	36	34	33	60	97	81	100	73	59	putative membrane protein	SAUSA300_0694
SAUSA300_2105	**1,0**	0,913736371	No	425	301	315	375	347	334	368	361	312	PTS system, mannitol specific IIBC component	mtlF
SAUSA300_1644	**1,0**	0,824528491	No	33792	31459	29585	28397	27928	27722	28172	28788	25805	pyruvate kinase	pyk
SAUSA300_2518	**1,0**	0,941666837	No	2949	3809	2531	1580	1287	2094	1782	1572	1537	hydrolase family protein	SAUSA300_2518
SAUSA300_0765	**1,0**	0,899557448	No	1462	1422	1419	1647	1697	1760	1661	1798	1568	SsrA-binding protein	smpB
SAUSA300_2375	**1,0**	0,962754282	No	43	86	69	57	43	83	57	65	60	ABC transporter, ATP-binding/permease protein	SAUSA300_2375
SAUSA300_2196	**1,0**	0,940435604	No	2999	3705	4196	4374	4429	5118	4618	4866	4235	50S ribosomal protein L29	rpmC
SAUSA300_2044	**1,0**	0,873729731	No	2379	2568	2104	1643	1630	1594	1612	1594	1593	cardiolipin synthetase	cls
SAUSA300_2627	**1,0**	0,906347142	No	2943	2717	2239	1932	1954	2020	1968	2102	1763	2-oxoglutarate/malate translocator	SAUSA300_2627
SAUSA300_0656	**1,0**	0,865486187	No	1297	1347	1353	1475	1596	1449	1471	1620	1366	conserved hypothetical protein	SAUSA300_0656
SAUSA300_2383	**1,0**	0,961298706	No	57	45	47	69	65	106	85	80	75	amino acid permease	SAUSA300_2383
SAUSA300_1247	**1,0**	0,916421132	No	944	758	775	891	875	762	870	821	804	conserved hypothetical protein	SAUSA300_1247
SAUSA300_1197	**1,0**	0,943326875	No	1452	1573	1231	1618	1476	1661	1685	1537	1483	glutathione peroxidase	SAUSA300_1197
SAUSA300_0317	**1,0**	0,944668726	No	532	654	499	699	629	735	671	678	694	conserved hypothetical protein	SAUSA300_0317
SAUSA300_0546	**1,0**	0,95735763	No	920	938	1086	1120	974	1236	912	1213	1172	sdrC protein	sdrC
SAUSA300_0922	**1,0**	0,957673639	No	2888	2560	1894	2665	2319	2703	2796	2619	2203	membrane protein, TerC family	SAUSA300_0922
SAUSA300_1576	**1,0**	0,921485995	No	5482	4931	4379	4586	4853	4143	4626	4340	4480	helicase, RecD/TraA family	SAUSA300_1576
SAUSA300_2515	**1,0**	0,970602196	No	55	41	61	100	79	74	103	66	83	transcriptional regulator, TetR family	SAUSA300_2515
SAUSA300_0839	**1,0**	0,959886421	No	2571	2480	2248	3115	3212	3416	3375	3331	2960	conserved hypothetical protein	SAUSA300_0839
SAUSA300_1359	**1,0**	0,905839417	No	3030	3356	3176	3151	3192	3107	3047	3224	3103	polyprenyl synthetase	SAUSA300_1359
SAUSA300_0486	**1,0**	0,944668726	No	6309	5790	5495	4978	4986	4476	5041	5226	4065	polyribonucleotide nucleotidyltransferase	SAUSA300_0486
SAUSA300_1590	**1,0**	0,954519591	No	11149	13471	10426	8819	8319	8209	8456	8844	7868	GTP pyrophosphokinase	SAUSA300_1590
SAUSA300_1632	**1,0**	0,950942674	No	997	1127	1020	765	762	807	770	798	752	conserved hypothetical protein	SAUSA300_1632

SAUSA300_1495	**1,0**	0,964249008	No	2801	2753	2618	3365	3827	3758	3943	3849	3089	conserved hypothetical protein	SAUSA300_1495
SAUSA300_0333	**1,0**	0,98140707	No	105	90	70	89	68	70	72	76	78	transcriptional antiterminator, BglG family	SAUSA300_0333
SAUSA300_0180	**1,0**	0,970602196	No	231	282	280	316	316	336	288	314	361	integral membrane protein LmrP	SAUSA300_0180
SAUSA300_0460	**1,0**	0,98558909	No	1822	2443	2819	2263	2328	2617	2305	2524	2358	conserved hypothetical protein	SAUSA300_0460
SAUSA300_0624	**1,0**	0,981603924	No	2942	2562	2787	4136	3741	4442	4080	3980	4238	teichoic acid translocation ATP-binding protein	tagH
SAUSA300_1364	**1,0**	0,978716776	No	5997	5833	6009	5495	5966	5508	5599	5794	5531	GTP-binding protein EngA	engA
SAUSA300_1660	**1,0**	0,994505078	No	150	174	224	223	309	238	224	264	273	putative membrane protein	SAUSA300_1660
SAUSA300_2526	**1,0**	0,98558909	No	2496	2096	2293	3752	4183	4033	4015	3888	4036	dihydroorotate dehydrogenase	pyrD
SAUSA300_1378	**1,0**	0,996675325	No	38	22	30	69	70	78	90	62	65	conserved hypothetical protein	SAUSA300_1378
SAUSA300_2597	**1,0**	0,996013839	No	117	131	109	205	187	166	173	196	186	capsular polysaccharide biosynthesis protein Cap1B	cap1B
SAUSA300_2211	**1,0**	0,997436359	No	398	371	345	427	440	398	436	414	412	putative membrane protein	SAUSA300_2211
SAUSA300_2166	**1,0**	1	No	844	582	666	1292	1179	1088	1022	1533	1002	alpha-acetolactate synthase	alsS
SAUSA300_0578	**1,0**	1	No	0	0	0	0	0	0	0	0	0	conserved hypothetical protein	SAUSA300_0578
SAUSA300_1422	**1,0**	1	No	0	0	0	0	0	0	0	0	0	phiSLT ORF65-like protein	SAUSA300_1422
SAUSA300_1663	**1,0**	1	No	0	0	0	0	0	0	0	0	0	conserved hypothetical protein	SAUSA300_1663
SAUSA300_0587	**1,0**	1	No	5	8	11	4	11	6	11	6	4	conserved hypothetical protein	SAUSA300_0587
SAUSA300_0158	**1,0**	1	No	41	66	40	96	86	142	107	106	114	capsular polysaccharide biosynthesis protein Cap5G	cap5G
SAUSA300_2593	**1,0**	0,999588948	No	565	652	475	365	370	444	390	398	394	conserved hypothetical protein	SAUSA300_2593
SAUSA300_1687	**1,0**	0,996675325	No	13105	13209	11282	14753	14923	14088	14798	14546	14440	FtsK/SpoIIIE family protein	SAUSA300_1687
SAUSA300_1570	**1,0**	0,996675325	No	2151	2250	2053	2114	2074	1970	2135	1986	2038	peptidase, U32 family	SAUSA300_1570
SAUSA300_1592	**1,0**	0,996675325	No	3555	3009	2813	2065	2200	1973	2147	2097	1994	single-stranded-DNA-specific exonuclease RecJ	recJ
SAUSA300_0426	**1,0**	0,999588948	No	1993	2687	2855	3295	3171	4293	2964	4669	3137	conserved hypothetical protein	SAUSA300_0426
SAUSA300_1155	**1,0**	0,995488965	No	2389	2625	2592	2778	3034	2566	2814	2872	2693	putative membrane-associated zinc metalloprotease	SAUSA300_1155
SAUSA300_2527	**1,0**	0,996013839	No	1125	986	1021	1564	1733	1593	1669	1525	1696	conserved hypothetical protein	SAUSA300_2527
SAUSA300_1289	**1,0**	0,996675325	No	665	842	723	228	237	253	209	255	254	dihydrodipicolinate reductase	dapB
SAUSA300_1858	**1,0**	0,98558909	No	1076	1142	979	778	841	843	824	882	760	conserved hypothetical protein	SAUSA300_1858
SAUSA300_2079	**1,0**	0,98558909	No	53555	45629	42197	45767	42675	44766	47140	50454	35980	fructose bisphosphate aldolase	fba
SAUSA300_0436	**1,0**	0,992157381	No	212	438	302	90	88	127	94	97	117	ABC transporter, permease protein	SAUSA300_0436
SAUSA300_2399	**1,0**	0,988730486	No	377	434	411	255	230	257	261	260	227	ABC transporter, ATP-binding protein	SAUSA300_2399
SAUSA300_0406	**1,0**	0,975396038	No	971	1112	985	642	586	626	670	592	602	putative restriction/modification system specificity prot	SAUSA300_0406
SAUSA300_0852	**1,0**	0,96940659	No	5384	5338	5044	5120	5540	4958	5173	5458	5043	Na(+)/H(+) antiporter subunit D	mnhD
SAUSA300_0833	**1,0**	0,962754282	No	1573	1796	1710	2016	1796	1986	1920	2022	1891	conserved hypothetical protein	SAUSA300_0833
SAUSA300_2434	**1,0**	0,981603924	No	96	116	83	152	178	124	154	143	154	transporter protein	SAUSA300_2434
SAUSA300_0673	**1,0**	0,970476271	No	298	309	294	381	383	359	380	374	373	cobalamin synthesis protein/P47K family protein	SAUSA300_0673
SAUSA300_0071	**1,0**	0,966720646	No	470	476	460	654	640	578	623	614	643	ISSep1-like transposase	SAUSA300_0071
SAUSA300_1630	**1,0**	0,958189394	No	2256	2697	2395	1935	1695	1791	1869	1834	1753	primosomal protein DnaI	dnaI
SAUSA300_2180	**1,0**	0,971107409	No	6316	7867	6821	9362	9268	10073	9937	10458	8460	30S ribosomal protein S13	rpsM
SAUSA300_1544	**1,0**	0,940051842	No	4991	4999	4754	4156	4526	4225	4448	4265	4251	GTP-binding protein LepA	lepA
SAUSA300_2397	**1,0**	0,975191604	No	3080	2357	2415	1956	2083	1866	2206	1874	1854	putative transport protein	SAUSA300_2397
SAUSA300_0889	**1,0**	0,98558909	No	351	702	698	185	196	325	215	251	246	oligopeptide ABC transporter, ATP-binding protein	oppD
SAUSA300_0787	**1,0**	0,966992179	No	239	315	254	262	257	247	266	233	271	3-dehydroquinate dehydratase, type I	aroD
SAUSA300_1567	**1,0**	0,942655185	No	4893	4397	4378	4081	4136	3394	3941	4038	3707	transcription elongation factor GreA	greA
SAUSA300_1470	**1,0**	0,918159276	No	3686	4145	3694	4258	4222	4284	4392	4238	4228	geranyltranstransferase	SAUSA300_1470
SAUSA300_2357	**1,0**	0,933352597	No	20727	20724	17144	14977	14795	15593	15332	15989	14379	ABC transporter, ATP-binding protein	SAUSA300_2357
SAUSA300_0515	**1,0**	0,951279104	No	980	1303	1338	1426	1384	1461	1324	1519	1463	cysteinyl-tRNA synthetase	cysS
SAUSA300_2395	**1,0**	0,962597199	No	3798	3088	3219	3902	4321	4072	4019	4304	4077	amino acid permease	SAUSA300_2395
SAUSA300_1873	**1,0**	0,918387146	No	1879	1797	1784	2243	2326	2317	2344	2342	2260	Mur ligase family protein	SAUSA300_1873
SAUSA300_0390	**1,0**	0,975396038	No	53	60	43	36	45	33	36	42	36	conserved hypothetical protein	SAUSA300_0390
SAUSA300_1168	**1,0**	0,89099162	No	6552	6401	6932	7218	6778	6832	6635	7512	6894	RNA-metabolising metallo-beta-lactamase	SAUSA300_1168
SAUSA300_1124	**1,0**	0,871775119	No	3765	4027	4252	7016	7032	7009	7140	7441	6690	3-oxoacyl-(acyl-carrier-protein) reductase	fabG
SAUSA300_0334	**1,0**	0,957673639	No	133	126	110	126	126	170	139	141	148	transcriptional regulator, MarR family	SAUSA300_0334
SAUSA300_2488	**1,0**	0,976398535	No	7	5	11	33	25	15	22	21	29	ferrous iron transport protein A	feoA
SAUSA300_1897	**1,0**	0,94957329	No	546	650	601	454	385	484	450	435	458	sodium-dependent transporter	SAUSA300_1897
SAUSA300_2026	**1,0**	0,917355974	No	4599	5211	4343	2596	2558	2601	2884	2626	2344	PemK family protein	SAUSA300_2026
SAUSA300_2390	**1,0**	0,926183079	No	782	639	652	2363	1814	2322	2488	1824	2282	glycine betaine/carnitine/choline transport system pern	opuCd
SAUSA300_2220	**1,0**	0,928179512	No	310	298	420	398	464	423	457	428	414	molybdopterin-guanine dinucleotide biosynthesis prote	mobA
SAUSA300_0772	**1,0**	0,939236038	No	15244	12177	11888	19484	20272	20772	19410	21706	20261	clumping factor A	clfA
SAUSA300_1586	**1,0**	0,835445877	No	5508	5634	5071	5627	5795	5473	5452	6043	5632	aspartyl-tRNA synthetase	aspS
SAUSA300_0387	**1,0**	0,931082735	No	23362	27908	23714	10665	10796	11230	11193	11431	10578	xanthine permease	pbuX

SAUSA300_1266	**1,0**	0,973638216	No	10	16	9	9	11	10	11	9	11	N-(5'phosphoribosyl)anthranilate isomerase	trpF
SAUSA300_1883	**1,0**	0,926183079	No	2646	3025	2639	1339	1267	1542	1411	1525	1279	high affinity proline permease	putP
SAUSA300_0097	**1,0**	0,868042168	No	839	1018	914	685	638	677	683	660	693	conserved hypothetical protein	SAUSA300_0097
SAUSA300_0332	**1,0**	0,96089238	No	52	33	32	40	23	28	33	36	26	PTS system, IIA component	SAUSA300_0332
SAUSA300_1329	**1,0**	0,962754282	No	43	59	89	43	47	53	44	55	46	amino acid permease	SAUSA300_1329
SAUSA300_1681	**1,0**	0,955999261	No	60	79	62	49	72	50	47	66	58	acetoin utilization protein AcuC	acuC
SAUSA300_2165	**1,0**	0,96126977	No	458	305	298	503	568	429	478	603	444	alpha-acetolactate decarboxylase	budA
SAUSA300_2505	**1,0**	0,966315778	No	129	82	172	93	230	98	122	112	189	acetyltransferase, GNAT family	SAUSA300_2505
SAUSA300_1320	**1,0**	0,899557448	No	2000	2049	1708	1104	1195	1119	1235	1128	1112	thymidylate synthase	thyA
SAUSA300_0168	**1,0**	0,94265134	No	126	178	174	113	115	114	98	119	130	conserved hypothetical protein	SAUSA300_0168
SAUSA300_1111	**1,0**	0,87219259	No	2566	2901	2994	2075	2367	2146	2161	2216	2317	conserved hypothetical protein	SAUSA300_1111
SAUSA300_0704	**1,0**	0,894339377	No	2728	2691	3054	3645	3579	3487	3929	3585	3389	ABC transporter, ATP-binding protein	SAUSA300_0704
SAUSA300_0338	**1,0**	0,913859024	No	148	156	156	127	115	138	135	137	120	glyoxalase family protein	SAUSA300_0338
SAUSA300_0166	**1,0**	0,899557448	No	369	376	309	431	424	454	494	412	429	capsular polysaccharide biosynthesis protein Cap5O	cap5O
SAUSA300_2432	**1,0**	0,910919334	No	177	242	232	169	187	194	169	200	190	hydrolase, MutT/nudix family	SAUSA300_2432
SAUSA300_1547	**1,0**	0,936953745	No	107	77	119	123	115	89	98	109	125	DNA internalization-related competence protein ComEC	SAUSA300_1547
SAUSA300_1170	**1,0**	0,821930289	No	1678	1566	1517	1366	1605	1374	1473	1392	1548	transcriptional regulator, GntR family	SAUSA300_1170
SAUSA300_0044	**1,0**	0,944668726	No	22	42	42	30	41	28	37	34	28	metallo-beta-lactamase family protein	SAUSA300_0044
SAUSA300_1280	**1,0**	0,940405877	No	55	51	50	30	36	46	35	34	45	phosphate ABC transporter, ATP-binding protein	pstB
SAUSA300_1809	**1,0**	0,848857547	No	1323	1339	1141	755	726	714	737	790	715	putative membrane protein	SAUSA300_1809
SAUSA300_2040	**1,0**	0,872323707	No	2813	2343	2526	3224	3577	2926	3504	3475	2951	putative membrane protein	SAUSA300_2040
SAUSA300_0175	**1,0**	0,911537695	No	3653	4939	3364	2814	2576	3163	2660	2769	3310	putative lipoprotein	SAUSA300_0175
SAUSA300_1332	**1,0**	0,766579593	No	1883	1829	1719	1813	1889	1751	1860	1949	1758	putative 5'-3' exonuclease	SAUSA300_1332
SAUSA300_2369	**1,0**	0,934355022	No	50	79	68	92	68	56	65	76	80	6-carboxyhexanoate--CoA ligase	SAUSA300_2369
SAUSA300_1565	**1,0**	0,895640171	No	79	110	111	153	124	145	134	161	141	putative urea amidolyase	SAUSA300_1565
SAUSA300_1042	**1,0**	0,846345334	No	1073	964	1034	1247	1240	1041	1253	1135	1216	DNA-dependent DNA polymerase family X	SAUSA300_1042
SAUSA300_0997	**1,0**	0,875884811	No	320	290	370	285	277	265	278	303	265	conserved hypothetical protein	SAUSA300_0997
SAUSA300_2532	**1,0**	0,818401552	No	1068	1340	1115	1338	1370	1396	1469	1339	1398	aspartate 1-decarboxylase	panD
SAUSA300_2550	**1,0**	0,906347142	No	119	127	145	278	255	229	244	248	288	anaerobic ribonucleotide reductase, small subunit	nrdG
SAUSA300_1785	**1,0**	0,898042315	No	1144	1083	1443	1804	2112	1877	1969	1850	2118	putative ABC transporter protein EcsB	SAUSA300_1785
SAUSA300_0601	**1,0**	0,833242299	No	901	1085	956	846	843	711	784	830	844	hydrolase, alpha/beta hydrolase fold family	SAUSA300_0601
SAUSA300_0368	**1,0**	0,905603865	No	1996	2976	3815	4152	4161	5324	4600	5002	4390	ribosomal protein S18	rpsR
SAUSA300_0986	**1,0**	0,938874062	No	1113	691	1192	3023	2536	2163	2334	3111	2484	cytochrome D ubiquinol oxidase, subunit I	SAUSA300_0986
SAUSA300_1290	**1,0**	0,871775119	No	482	508	537	185	151	217	184	210	181	tetrahydrodipicolinate acetyltransferase	dapD
SAUSA300_1058	**1,0**	0,903209042	No	987	1148	856	524	627	482	401	580	693	alpha-hemolysin precursor	SAUSA300_1058
SAUSA300_2201	**1,0**	0,878677229	No	16175	18466	19526	23482	25192	28806	26698	28943	23884	50S ribosomal protein L2	rplB
SAUSA300_1062	**1,0**	0,934218311	No	38	33	37	30	14	34	29	30	25	ornithine carbamoyltransferase	argF
SAUSA300_1465	**1,0**	0,725869715	No	3285	3406	2847	4067	4246	3803	4302	4127	4007	2-oxoisovalerate dehydrogenase, E1 component, beta s	SAUSA300_1465
SAUSA300_0462	**1,0**	0,712239242	No	1586	1808	1651	1376	1463	1545	1572	1499	1434	conserved hypothetical protein	SAUSA300_0462
SAUSA300_2045	**1,0**	0,849849901	No	963	921	823	567	543	621	633	566	583	HD domain protein	SAUSA300_2045
SAUSA300_1682	**1,0**	0,838609884	No	2828	3837	2753	4200	3798	3919	4480	4106	3677	catabolite control protein A	ccpA
SAUSA300_0366	**1,0**	0,881174683	No	4816	6621	7870	9353	9169	11423	10129	11403	9289	ribosomal protein S6	rpsF
SAUSA300_1362	**1,0**	0,838609884	No	86334	65394	67123	78261	95419	86145	97351	89033	81088	DNA-binding protein HU	hup
SAUSA300_2321	**1,0**	0,847214182	No	214	196	240	213	147	209	196	199	197	putative membrane protein	SAUSA300_2321
SAUSA300_1078	**1,0**	0,699650717	No	4311	5139	4392	5551	5603	5680	5746	5810	5781	cell division protein	divIB
SAUSA300_1661	**1,0**	0,776177978	No	541	546	639	607	703	719	668	703	715	thiamine biosynthesis protein ThiI	thiI
SAUSA300_0004	**1,0**	0,698492004	No	3326	3655	3462	3953	3629	3667	3835	3987	3777	DNA replication and repair protein recF	recF
SAUSA300_1350	**1,0**	0,748899737	No	239	231	254	381	363	348	381	373	373	conserved hypothetical protein	SAUSA300_1350
SAUSA300_0925	**1,0**	0,74985731	No	932	1087	993	1053	1003	983	1063	1066	1007	5' nucleotidase family protein	SAUSA300_0925
SAUSA300_1407	**1,0**	0,918159276	No	50	49	37	27	23	44	34	33	33	phi SLT ORF 145-like protein, phage transcriptional regu	SAUSA300_1407
SAUSA300_1264	**1,0**	0,924071072	No	31	27	35	49	45	31	45	35	47	anthranilate phosphoribosyltransferase	trpD
SAUSA300_1683	**1,0**	0,723946882	No	4297	5140	4891	2250	2087	2174	2177	2202	2341	chorismate mutase/phospho-2-dehydro-3-deoxyheptor	SAUSA300_1683
SAUSA300_1484	**1,0**	0,951741232	No	5	5	8	6	18	7	12	8	10	conserved hypothetical protein	SAUSA300_1484
SAUSA300_0677	**1,0**	0,707496044	No	858	920	830	589	543	552	547	617	578	putative deoxyribodipyrimidine photolyase	SAUSA300_0677
SAUSA300_2614	**1,0**	0,795438168	No	684	701	658	1227	1023	1139	1025	1298	1182	putative lipoprotein	SAUSA300_2614
SAUSA300_0221	**1,0**	0,944246385	No	84	105	140	112	128	136	87	228	76	pyruvate formate-lyase activating enzyme	pflA
SAUSA300_1791	**1,0**	0,743096682	No	7062	6566	5519	5736	5687	5554	6229	5943	5377	cmp-binding-factor 1	cbf1
SAUSA300_1136	**1,0**	0,805213448	No	494	617	604	432	320	441	443	404	398	putative GTP-binding protein	SAUSA300_1136
SAUSA300_0774	**1,0**	0,894237283	No	98	83	75	36	38	37	35	42	39	secretory extracellular matrix and plasma binding prote	empbp

SAUSA300_2446	**1,0**	0,785638627	No	537	497	632	1113	1030	856	986	1082	1039	conserved hypothetical protein	SAUSA300_2446
SAUSA300_1119	**1,0**	0,422722241	No	8911	9022	8483	10976	11220	10555	11610	11454	10878	conserved hypothetical protein	SAUSA300_1119
SAUSA300_1556	**1,0**	0,648677292	No	1769	2026	1997	1494	1542	1470	1552	1515	1607	putative GTP-binding protein	SAUSA300_1556
SAUSA300_1004	**1,0**	0,766408123	No	1159	947	1031	1083	881	801	1077	894	905	conserved hypothetical protein	SAUSA300_1004
SAUSA300_1566	**1,0**	0,818401552	No	76	82	86	90	97	123	98	112	114	conserved hypothetical protein	SAUSA300_1566
SAUSA300_2198	**1,0**	0,819468019	No	8860	11097	11807	13032	13963	15159	14536	15763	13508	30S ribosomal protein S3	rpsC
SAUSA300_0429	**1,0**	0,563892021	No	1245	1250	1160	1289	1310	1150	1339	1232	1319	PAP2 family protein	SAUSA300_0429
SAUSA300_1696	**1,0**	0,541669058	No	2778	3025	2954	2442	2581	2496	2570	2654	2590	D-alanine aminotransferase	dat
SAUSA300_0893	**1,0**	0,922801501	No	21	21	14	11	14	19	19	14	14	oligopeptide ABC transporter, ATP-binding protein	oppF
SAUSA300_0017	**1,0**	0,777715626	No	417	645	445	1524	1569	1894	1889	1627	1680	adenylosuccinate synthetase	purA
SAUSA300_1525	**1,0**	0,615133984	No	14483	13716	12493	13284	12741	12009	12824	14314	12444	glycyl-tRNA synthetase	glyS
SAUSA300_1878	**1,0**	0,673213631	No	730	717	623	780	773	865	911	806	805	RNA methyltransferase, TrmA family	rumA
SAUSA300_1588	**1,0**	0,69618924	No	5997	5905	4686	4263	3877	3991	4513	4177	3952	N-acetylmuramoyl-L-alanine amidase	lytH
SAUSA300_0667	**1,0**	0,815562766	No	1474	1580	1055	971	832	846	1034	945	786	YaiI/YqxD family protein	SAUSA300_0667
SAUSA300_0156	**1,0**	0,872885859	No	28	19	22	64	47	74	70	52	74	capsular polysaccharide biosynthesis protein Cap5E	cap5E
SAUSA300_0196	**1,0**	0,74697663	No	4218	4757	3543	3278	3180	2974	3217	3437	3182	type I restriction-modification enzyme, R subunit	hsdR
SAUSA300_1075	**1,0**	0,474054728	No	10167	9925	9504	9833	9946	9344	9922	10827	9617	penicillin-binding protein 1	pbpA
SAUSA300_1978	**1,0**	0,894737304	No	38	62	56	37	56	43	41	38	61	ferric hydroxamate receptor	SAUSA300_1978
SAUSA300_0090	**1,0**	0,766090482	No	86	79	99	159	131	135	145	158	143	conserved hypothetical protein	SAUSA300_0090
SAUSA300_1114	**1,0**	0,645727189	No	763	731	844	862	1055	884	993	943	975	ribosome small subunit-dependent GTPase A	rsgA
SAUSA300_1185	**1,0**	0,608318058	No	4292	5162	4396	3460	3392	2935	3417	3333	3467	tRNA-i(6)A37 thiotransferase enzyme MiaB	miaB
SAUSA300_0249	**1,0**	0,67363013	No	2069	2003	1943	2950	3167	2527	3060	2940	3023	2-C-methyl-D-erythritol 4-phosphate cytidylyltransferas	SAUSA300_0249
SAUSA300_1899	**1,0**	0,683439446	No	4538	4547	4395	3123	3347	2910	3305	3430	3069	conserved hypothetical protein	SAUSA300_1899
SAUSA300_1446	**1,0**	0,677317943	No	148	136	126	228	212	229	232	220	249	conserved hypothetical protein	SAUSA300_1446
SAUSA300_2171	**1,0**	0,742897812	No	18329	17566	16247	18087	19598	21922	21033	22514	18893	30S ribosomal protein S9	rpsI
SAUSA300_2186	**1,0**	0,759739169	No	3260	4316	4042	4743	4877	5090	5249	5445	4720	50S ribosomal protein L30	rpmD
SAUSA300_1016	**1,0**	0,824111147	No	1932	2064	3374	2148	2047	2226	1959	2342	2435	protoheme IX farnesyltransferase	cyoE
SAUSA300_1502	**1,0**	0,933352597	No	17	5	8	6	5	7	6	6	7	putative competence protein ComGC	SAUSA300_1502
SAUSA300_0733	**1,0**	0,678222453	No	1151	1355	1715	1249	1206	1275	1310	1335	1268	degV family protein	SAUSA300_0733
SAUSA300_0748	**1,0**	0,597797269	No	773	778	881	939	780	760	832	870	904	conserved hypothetical protein	SAUSA300_0748
SAUSA300_0526	**1,0**	0,52714916	No	1612	1578	1622	1906	2085	1987	2090	2064	2110	Methyltransferase small domain	SAUSA300_0526
SAUSA300_1657	**1,0**	0,531984888	No	12418	11731	10658	8053	7282	7627	8200	8449	7465	acetate kinase	ackA
SAUSA300_2379	**1,0**	0,70026182	No	2682	2254	2482	1219	1226	1167	1263	1324	1210	putative transporter protein	SAUSA300_2379
SAUSA300_1102	**1,0**	0,549433772	No	4475	4887	5145	3252	3113	3278	3511	3480	3156	guanylate kinase	gmk
SAUSA300_1109	**1,0**	0,653692056	No	1998	2357	2034	1691	1830	1931	1968	1894	1874	methionyl-tRNA formyltransferase	fmt
SAUSA300_2355	**1,0**	0,872885859	No	41	48	93	34	36	56	32	49	53	putative lipoprotein	SAUSA300_2355
SAUSA300_0623	**0,9**	0,573838146	No	2137	1878	1716	1700	1612	1837	1807	1872	1748	teichoic acid biosynthesis protein	tagA
SAUSA300_2129	**0,9**	0,763065776	No	436	423	706	699	818	685	718	779	821	putative hemolysin III	SAUSA300_2129
SAUSA300_0104	**0,9**	0,522199819	No	2267	2133	1834	2490	2362	2183	2466	2562	2389	transcriptional regulator, AraC family	SAUSA300_0104
SAUSA300_2456	**0,9**	0,70026182	No	785	819	1003	730	746	608	633	796	765	putative membrane protein	SAUSA300_2456
SAUSA300_1287	**0,9**	0,78264143	No	765	997	868	262	243	257	219	295	292	aspartate semialdehyde dehydrogenase	asd
SAUSA300_1879	**0,9**	0,381345336	No	1421	1589	1418	1318	1242	1343	1362	1391	1372	conserved hypothetical protein	SAUSA300_1879
SAUSA300_0626	**0,9**	0,589271995	No	815	856	942	1296	1443	1264	1318	1485	1419	teichoic acid biosynthesis protein B	tagB
SAUSA300_1757	**0,9**	0,922848544	No	14	7	6	3	2	12	6	8	6	serine protease SplB	splB
SAUSA300_0165	**0,9**	0,67766011	No	195	172	155	215	207	186	230	193	220	capsular polysaccharide biosynthesis protein Cap5N	cap5N
SAUSA300_0863	**0,9**	0,640926412	No	574	638	552	427	347	401	392	437	419	argininosuccinate lyase	argH
SAUSA300_0574	**0,9**	0,622099876	No	1905	1752	1538	1714	1760	1911	2040	1844	1816	phosphomevalonate kinase	SAUSA300_0574
SAUSA300_1371	**0,9**	0,514223379	No	1455	1562	1300	1070	1073	896	1085	1074	1054	ATP-dependent DNA helicase RecQ	recQ
SAUSA300_0980	**0,9**	0,416968324	No	4948	4223	4280	3070	2982	2946	3080	3306	3142	putative membrane protein	SAUSA300_0980
SAUSA300_1520	**0,9**	0,627924481	No	704	757	749	604	631	603	648	640	657	conserved hypothetical protein	SAUSA300_1520
SAUSA300_1505	**0,9**	0,426345722	No	1605	1656	1687	1432	1594	1510	1660	1510	1626	conserved hypothetical protein	SAUSA300_1505
SAUSA300_0844	**0,9**	0,368413035	No	22616	22164	20527	23396	23896	23484	25405	27136	22436	conserved hypothetical protein	SAUSA300_0844
SAUSA300_2208	**0,9**	0,53893419	No	2682	2202	2262	2187	2391	2055	2297	2511	2214	DNA topoisomerase III	topB
SAUSA300_1710	**0,9**	0,573948277	No	1209	1257	1355	1909	1855	1891	2031	2087	1878	putative lysophospholipase	SAUSA300_1710
SAUSA300_2642	**0,9**	0,89855178	No	17	8	23	19	14	6	9	19	12	conserved hypothetical protein	SAUSA300_2642
SAUSA300_0796	**0,9**	0,76820128	No	1934	3267	2212	666	613	747	724	773	654	ABC transporter, ATP-binding protein	SAUSA300_0796
SAUSA300_1420	**0,9**	0,918433099	No	0	0	0	1	0	0	1	1	0	conserved hypothetical phage protein	SAUSA300_1420
SAUSA300_2243	**0,9**	0,699836727	No	305	313	355	4196	3760	3558	3873	4194	4146	urease accessory protein UreG	ureG
SAUSA300_1754	**0,9**	0,90549721	No	3	11	13	13	5	10	9	10	12	serine protease SplE	splE

SAUSA300_1291	**0,9**	0,613795114	No	589	600	588	235	219	217	230	238	245	hippurate hydrolase	SAUSA300_1291
SAUSA300_0835	**0,9**	0,515132382	No	11107	11665	9370	10385	10075	10036	11807	10348	10214	D-alanine-activating enzyme/D-alanine-D-alanyl, dltA pr	dltA
SAUSA300_1877	**0,9**	0,716599695	No	179	222	235	160	162	197	180	166	207	conserved hypothetical protein	SAUSA300_1877
SAUSA300_2421	**0,9**	0,675947481	No	220	205	250	315	279	251	325	244	330	conserved hypothetical protein	SAUSA300_2421
SAUSA300_1366	**0,9**	0,743096682	No	112	105	120	70	56	56	69	66	61	conserved hypothetical protein	SAUSA300_1366
SAUSA300_0984	**0,9**	0,428975253	No	45888	44884	37544	42486	38452	38072	43130	44971	38326	phosphoenolpyruvate-protein phosphotransferase	ptsI
SAUSA300_1642	**0,9**	0,557349723	No	14902	14113	13507	14587	14479	13100	14923	16217	13669	D-serine/D-alanine/glycine transporter	SAUSA300_1642
SAUSA300_0190	**0,9**	0,588165804	No	2079	1838	1470	2972	2545	2758	2983	2799	3021	indole-3-pyruvate decarboxylase	ipdC
SAUSA300_1631	**0,9**	0,462478572	No	2134	2476	2304	1555	1519	1631	1580	1773	1654	replication initiation and membrane attachment protein	SAUSA300_1631
SAUSA300_1895	**0,9**	0,654537268	No	1116	909	1030	566	660	568	613	642	650	nitric oxide synthase oxygenase	SAUSA300_1895
SAUSA300_1154	**0,9**	0,553665295	No	1287	1240	1503	928	1057	967	1001	1076	1059	phosphatidate cytidylyltransferase	cdsA
SAUSA300_0921	**0,9**	0,429298394	No	3937	4222	4292	3918	3852	3967	4049	4271	4171	peptide chain release factor 3	prfC
SAUSA300_0058	**0,9**	0,911999763	No	10	12	7	7	7	6	7	4	9	conserved hypothetical protein	SAUSA300_0058
SAUSA300_0251	**0,9**	0,390581029	No	6660	6398	6168	10269	11425	9358	10968	11236	10837	putative teichoic acid biosynthesis protein	SAUSA300_0251
SAUSA300_2093	**0,9**	0,667919467	No	374	346	342	377	329	311	339	403	342	conserved hypothetical protein	SAUSA300_2093
SAUSA300_2493	**0,9**	0,776565832	No	57	40	43	77	131	80	102	117	83	conserved hypothetical protein	SAUSA300_2493
SAUSA300_0419	**0,9**	0,723946882	No	157	283	249	546	449	584	539	548	600	staphylococcal tandem lipoprotein	SAUSA300_0419
SAUSA300_0434	**0,9**	0,648677292	No	2024	1610	1476	5264	4479	4824	4984	5168	5376	cystathionine gamma-synthase	metB
SAUSA300_1388	**0,9**	0,860458018	No	26	15	15	17	20	19	25	16	19	phiSLT ORF488-like protein	SAUSA300_1388
SAUSA300_1474	**0,9**	0,431379491	No	1142	1278	1050	1130	1219	1091	1266	1216	1177	conserved hypothetical protein	SAUSA300_1474
SAUSA300_1228	**0,9**	0,580347474	No	1602	1686	1533	693	719	652	703	720	776	homoserine kinase	thrB
SAUSA300_1619	**0,9**	0,494769558	No	10391	9962	11129	8760	7832	7299	8109	8804	8583	glutamyl-tRNA reductase	hemA
SAUSA300_2573	**0,9**	0,673213631	No	2308	2004	1721	3542	2973	3201	3910	3371	3097	immunodominant antigen B	isaB
SAUSA300_2374	**0,9**	0,663944102	No	164	177	172	158	140	111	160	132	143	ABC transporter, ATP-binding/permease protein	SAUSA300_2374
SAUSA300_1649	**0,9**	0,310950218	No	3436	3221	3266	3102	3433	2906	3438	3238	3391	DNA polymerase III, alpha subunit	dnaE
SAUSA300_1257	**0,9**	0,360671325	No	2263	2092	2077	2213	2272	2112	2301	2297	2445	peptide methionine sulfoxide reductase regulator MsrR	msrR
SAUSA300_1800	**0,9**	0,541669058	No	3584	2569	2655	1615	1621	1488	1824	1655	1573	ribosomal large subunit pseudouridine synthase, RluD su	SAUSA300_1800
SAUSA300_2009	**0,9**	0,716479797	No	291	376	319	83	72	102	102	90	88	ketol-acid reductoisomerase	ilvC
SAUSA300_0524	**0,9**	0,675947481	No	29201	27270	27286	24272	23506	23979	24775	28412	23633	ribosomal protein L10	rplJ
SAUSA300_0897	**0,9**	0,500120091	No	2833	3673	3343	1933	1893	1942	2103	2040	2036	tryptophanyl-tRNA synthetase	trpS
SAUSA300_2202	**0,9**	0,594805145	No	4583	5423	5643	6212	6588	6752	7120	7431	6393	50S ribosomal protein L23	rplW
SAUSA300_1745	**0,9**	0,885494333	No	14	18	29	7	14	16	14	12	15	conserved hypothetical protein	SAUSA300_1745
SAUSA300_0229	**0,9**	0,723946882	No	55	66	50	87	83	90	93	110	80	putative acyl-CoA transferase FadX	SAUSA300_0229
SAUSA300_1549	**0,9**	0,602523903	No	260	270	282	169	219	186	193	212	204	ComE operon protein 1	SAUSA300_1549
SAUSA300_2389	**0,9**	0,538530688	No	6004	6141	6369	4984	5700	4723	5355	5724	5465	putative drug transporter	SAUSA300_2389
SAUSA300_1023	**0,9**	0,436389698	No	508	498	510	637	604	708	687	697	716	conserved hypothetical protein	SAUSA300_1023
SAUSA300_1658	**0,9**	0,597392065	No	1393	1393	1260	2425	2074	2069	2551	2157	2354	conserved hypothetical protein	SAUSA300_1658
SAUSA300_0243	**0,9**	0,90549721	No	2	8	7	7	5	1	2	6	6	conserved hypothetical protein	SAUSA300_0243
SAUSA300_1195	**0,9**	0,349278464	No	1228	1088	1104	934	866	809	883	939	980	tRNA delta(2)-isopentenylpyrophosphate transferase	miaA
SAUSA300_2486	**0,9**	0,656932661	No	5257	4086	3451	2049	1479	1998	2019	2020	1917	putative ATP-dependent Clp proteinase	SAUSA300_2486
SAUSA300_2003	**0,9**	0,567592983	No	536	513	546	643	676	630	697	629	768	ribosomal-protein-alanine acetyltransferase	rimI
SAUSA300_0914	**0,9**	0,564202383	No	448	553	455	282	300	312	311	364	288	sodium:alanine symporter family protein	SAUSA300_0914
SAUSA300_1599	**0,9**	0,686823452	No	427	386	567	364	408	294	370	378	396	ACT domain protein PheB	SAUSA300_1599
SAUSA300_1202	**0,9**	0,918159276	No	3	0	9	4	0	0	2	1	3	conserved hypothetical protein	SAUSA300_1202
SAUSA300_0299	**0,9**	0,768888386	No	10	42	47	62	65	62	65	68	70	conserved hypothetical protein	SAUSA300_0299
SAUSA300_1299	**0,9**	0,423482877	No	1967	1953	2099	2499	2615	2470	2498	2709	2962	putative tellurite resistance protein	SAUSA300_1299
SAUSA300_2189	**0,9**	0,623095578	No	6231	7330	7767	7613	7995	8093	8669	9183	7708	50S ribosomal protein L6	rplF
SAUSA300_1626	**0,9**	0,463192589	No	8131	6674	6727	6599	6095	6385	7098	7333	6143	50S ribosomal protein L35	rpmI
SAUSA300_0411	**0,9**	0,778565657	No	16	7	19	32	34	44	42	35	41	staphylococcal tandem lipoprotein	SAUSA300_0411
SAUSA300_1188	**0,9**	0,341625968	No	1932	1855	1812	2728	2867	2743	3024	2827	3136	DNA mismatch repair protein mutS	mutS
SAUSA300_0582	**0,9**	0,698492004	No	177	164	160	126	110	104	142	102	124	conserved hypothetical protein	SAUSA300_0582
SAUSA300_2069	**0,9**	0,372091872	No	753	865	742	597	582	540	625	650	580	conserved hypothetical protein	SAUSA300_2069
SAUSA300_0163	**0,9**	0,540099909	No	181	190	153	239	219	228	265	225	253	capsular polysaccharide biosynthesis protein Cap5L	cap5L
SAUSA300_0659	**0,9**	0,270903878	No	1087	1021	1012	1266	1377	1146	1383	1364	1340	sugar efflux transporter	SAUSA300_0659
SAUSA300_1900	**0,9**	0,291945072	No	11261	11249	10510	7070	7226	6048	7349	7640	7025	manganese-dependent inorganic pyrophosphatase	ppaC
SAUSA300_1700	**0,9**	0,32613024	No	2186	2149	2303	2174	2459	1948	2355	2415	2339	polysaccharide biosynthesis protein	SAUSA300_1700
SAUSA300_2205	**0,9**	0,582401489	No	9444	10562	11219	14046	14819	17080	16926	17985	14880	30S ribosomal protein S10	rpsJ
SAUSA300_0238	**0,9**	0,747427317	No	29	34	28	42	34	34	44	44	33	transcriptional antiterminator, BglG family	SAUSA300_0238
SAUSA300_1562	**0,9**	0,605101044	No	76	90	105	160	126	195	176	190	164	LamB/YcsF family protein	SAUSA300_1562

SAUSA300_0698	**0,9**	0,627153492	No	258	220	156	153	178	139	184	168	155	para-aminobenzoate synthase, glutamine amidotransfe	pabA
SAUSA300_2059	**0,9**	0,291945072	No	11807	13765	12640	13851	13388	13047	14323	14736	14643	ATP synthase F1, gamma subunit	atpG
SAUSA300_2233	**0,9**	0,510328443	No	222	241	246	183	167	201	211	199	193	BioY family protein	SAUSA300_2233
SAUSA300_2443	**0,9**	0,720573766	No	127	167	146	176	203	234	180	296	192	gluconate kinase	gntK
SAUSA300_2459	**0,9**	0,602004609	No	189	218	170	179	174	235	219	233	191	transcriptional regulator, MarR family	SAUSA300_2459
SAUSA300_0482	**0,9**	0,455597981	No	1431	1577	1476	1376	1512	1281	1569	1459	1497	polysaccharide biosynthesis protein	SAUSA300_0482
SAUSA300_2417	**0,9**	0,503862741	No	2580	2768	2766	750	728	784	794	873	795	putative transporter	SAUSA300_2417
SAUSA300_2000	**0,9**	0,495654437	No	1912	2119	1687	1488	1377	1431	1594	1594	1489	ABC transporter, ATP-binding protein	vga
SAUSA300_0716	**0,9**	0,507879786	No	12895	13734	13891	9284	10386	9301	9887	10952	10699	ribonucleoside-diphosphate reductase, alpha subunit	SAUSA300_0716
SAUSA300_1611	**0,9**	0,370187706	No	5629	6925	7415	5929	6626	6549	6521	7219	7071	valyl-tRNA synthetase	valS
SAUSA300_2338	**0,9**	0,429700173	No	1178	1487	1112	812	834	837	821	928	956	sensor histidine kinase	SAUSA300_2338
SAUSA300_1870	**0,9**	0,480447816	No	486	496	460	243	234	170	236	253	216	conserved hypothetical protein	SAUSA300_1870
SAUSA300_1604	**0,9**	0,727697933	No	155	142	211	160	176	172	183	136	235	rod shape-determining protein MreD	mreD
SAUSA300_0337	**0,9**	0,482714962	No	563	538	508	344	313	352	362	409	333	glycerol-3-phosphate transporter	glpT
SAUSA300_0882	**0,9**	0,538925018	No	150	203	155	146	131	142	155	147	157	conserved hypothetical protein	SAUSA300_0882
SAUSA300_2160	**0,9**	0,606511333	No	67	51	51	82	126	108	124	103	114	transcriptional regulator, MerR family	SAUSA300_2160
SAUSA300_1653	**0,9**	0,556608772	No	1333	1688	1320	1002	791	945	1165	955	882	conserved hypothetical protein	SAUSA300_1653
SAUSA300_0532	**0,9**	0,429677624	No	133142	144012	127175	124954	130005	137370	138594	158620	131668	translation elongation factor G	fusA
SAUSA300_0027	**0,9**	0,52714916	No	934	1010	821	646	674	609	711	703	695	conserved hypothetical protein	SAUSA300_0027
SAUSA300_1403	**0,9**	0,654822763	No	74	85	64	66	63	56	75	61	67	phiSLT ORF412-like protein, portal protein	SAUSA300_1403
SAUSA300_0867	**0,9**	0,369471181	No	1340	1006	1243	1415	1551	1511	1683	1593	1616	signal peptidase IA	spsA
SAUSA300_0786	**0,9**	0,777715626	No	350	270	240	153	77	257	196	205	142	OsmC/Ohr family protein	SAUSA300_0786
SAUSA300_1672	**0,9**	0,11332896	No	3021	3170	3016	1978	1884	1851	1963	2159	2127	phosphotransferase system, N-acetylglucosamine-speci	nagE
SAUSA300_0535	**0,9**	0,331393175	No	940	1035	1237	1935	1774	2045	2026	2390	1890	putative pyridoxal phosphate-dependent acyltransferas	SAUSA300_0535
SAUSA300_0323	**0,9**	0,875884811	No	2	0	1	0	0	0	1	0	1	conserved hypothetical protein	SAUSA300_0323
SAUSA300_1443	**0,9**	0,148972253	No	1009	1040	1048	951	1046	1000	1107	1030	1133	ribosomal large subunit pseudouridine synthase B, RluB	rluB
SAUSA300_0108	**0,9**	0,675947481	No	346	237	193	226	162	210	237	223	201	antigen, 67 kDa	SAUSA300_0108
SAUSA300_0675	**0,9**	0,727697933	No	95	51	93	74	50	75	80	85	57	conserved hypothetical protein	SAUSA300_0675
SAUSA300_0923	**0,9**	0,328715843	No	2089	2323	2105	1842	1920	1661	1883	2007	2048	serine protease	htrA
SAUSA300_2291	**0,9**	0,484754046	No	413	428	534	451	566	575	565	598	579	sodium/glutamate symporter	gltS
SAUSA300_1603	**0,9**	0,36597292	No	12050	12670	13242	14619	13807	14585	16190	16321	14714	50S ribosomal protein L21	rplU
SAUSA300_1123	**0,9**	0,166947898	No	3999	4223	4920	6267	6313	5826	6455	7088	6669	malonyl CoA-acyl carrier protein transacylase	fabD
SAUSA300_0137	**0,9**	0,404447893	No	596	686	529	518	392	484	484	538	521	transcriptional regulator, GntR family	SAUSA300_0137
SAUSA300_2043	**0,9**	0,623914119	No	150	111	116	97	90	109	128	98	102	conserved hypothetical protein	SAUSA300_2043
SAUSA300_1162	**0,9**	0,055141287	No	9261	9980	9181	10231	10789	10458	11398	11942	11263	translation initiation factor IF-2	infB
SAUSA300_0340	**0,9**	0,517687177	No	172	153	145	133	92	124	143	109	140	NADH-dependent FMN reductase	SAUSA300_0340
SAUSA300_1251	**0,9**	0,291945072	No	4154	4345	4556	4517	4799	3895	4583	4859	5099	DNA topoisomerase IV, subunit A	parC
SAUSA300_0444	**0,9**	0,557349723	No	152	162	135	199	189	167	175	224	212	LysR family regulatory protein	gltC
SAUSA300_0164	**0,9**	0,556608772	No	105	107	82	123	92	124	140	112	128	capsular polysaccharide biosynthesis protein Cap5M	cap5M
SAUSA300_2255	**0,9**	0,231809526	No	1693	1870	1558	947	994	974	1130	1075	1009	monooxygenase family protein	SAUSA300_2255
SAUSA300_2101	**0,9**	0,811492711	No	41	36	110	36	56	47	49	40	66	SAP domain protein	SAUSA300_2101
SAUSA300_0918	**0,9**	0,383889662	No	4860	3773	3422	4786	4722	4124	5111	5140	4799	conserved hypothetical protein	SAUSA300_0918
SAUSA300_1605	**0,9**	0,362129639	No	949	1118	1080	1091	1107	1066	1231	1182	1194	rod shape-determining protein MreC	mreC
SAUSA300_1221	**0,9**	0,883157951	No	0	1	1	0	9	3	5	4	4	conserved hypothetical protein	SAUSA300_1221
SAUSA300_0034	**0,9**	0,812913397	No	12	12	13	14	29	6	20	14	18	IS1272, transposase	SAUSA300_0034
SAUSA300_1115	**0,9**	0,264206988	No	818	869	855	935	1021	750	950	1058	973	ribulose-phosphate 3-epimerase	rpe
SAUSA300_0753	**0,9**	0,508098015	No	1109	1157	923	460	392	492	502	556	436	conserved hypothetical protein	SAUSA300_0753
SAUSA300_1467	**0,9**	0,093995001	No	3953	4390	3839	4955	5170	5006	5454	5783	5494	2-oxoisovalerate dehydrogenase, E3 component, lipoam	lpdA
SAUSA300_1889	**0,9**	0,291945072	No	5038	5646	4039	6095	5955	6390	7312	6451	6648	adenylosuccinate lyase	purB
SAUSA300_1088	**0,9**	0,306736948	No	353	370	355	627	489	489	606	588	591	glyoxalase family protein	SAUSA300_1088
SAUSA300_2630	**0,9**	0,422622128	No	1135	945	817	2220	2024	1794	2416	2166	2104	high-affinity nickel-transporter	nixA
SAUSA300_0671	**0,9**	0,572150875	No	982	875	1026	780	868	671	857	909	802	ABC transporter, ATP-binding protein, MsbA family	SAUSA300_0671
SAUSA300_1629	**0,9**	0,424556048	No	10723	11424	8958	7159	6681	6978	7852	7953	7262	threonyl-tRNA synthetase	thrS
SAUSA300_0107	**0,9**	0,391774598	No	7540	9043	7329	4896	5297	5301	5632	6189	5346	Na/Pi cotransporter family protein	SAUSA300_0107
SAUSA300_1145	**0,9**	0,417739722	No	694	750	1105	1256	1240	1289	1278	1456	1461	tyrosine recombinase xerC	xerC
SAUSA300_0416	**0,9**	0,715756307	No	5	18	22	26	27	43	34	32	41	staphylococcal tandem lipoprotein	SAUSA300_0416
SAUSA300_2548	**0,9**	0,377337031	No	295	249	252	243	214	234	257	243	269	conserved hypothetical protein	SAUSA300_2548
SAUSA300_2564	**0,9**	0,403839032	No	431	580	426	318	307	303	351	335	343	tributyrin esterase	estA
SAUSA300_0579	**0,9**	0,52681489	No	522	676	631	511	489	540	552	535	623	conserved hypothetical protein	SAUSA300_0579

SAUSA300_1969	**0,9**	0,605101044	No	1833	2515	1330	1161	915	1266	1342	1213	1166	phi77 ORF011-like protein, phage transcriptional repres	SAUSA300_1969
SAUSA300_0189	**0,9**	0,339743233	No	539	430	369	646	597	660	764	674	680	isochorismatase	entB
SAUSA300_0904	**0,9**	0,255357711	No	1374	1318	1188	1716	1724	1421	1860	1784	1750	protozoan/cyanobacterial globin family protein	SAUSA300_0904
SAUSA300_2091	**0,9**	0,376298786	No	6097	5333	5207	7420	7679	5860	8162	8610	6523	purine nucleoside phosphorylase	deoD
SAUSA300_0277	**0,9**	0,505458768	No	1411	1102	807	1320	1084	1319	1435	1386	1327	putative staphyloxanthin biosynthesis protein	SAUSA300_0277
SAUSA300_2555	**0,9**	0,527033	No	105	104	128	103	110	109	113	126	120	glutathione peroxidase	SAUSA300_2555
SAUSA300_1063	**0,9**	0,635213367	No	57	51	71	40	50	30	43	44	44	carbamate kinase	arcC
SAUSA300_2071	**0,9**	0,306589629	No	3576	3166	3051	2276	2452	2196	2540	2606	2550	modification methylase, HemK family	SAUSA300_2071
SAUSA300_1572	**0,9**	0,312272795	No	2050	1885	2137	1770	2056	1823	2213	2067	1999	conserved hypothetical protein	SAUSA300_1572
SAUSA300_0685	**0,9**	0,412857858	No	3297	3852	4462	6672	5858	7236	6849	8719	6441	fructose specific permease	fruA
SAUSA300_1037	**0,9**	0,204140798	No	1848	2025	1896	1309	1319	1260	1395	1506	1425	phenylalanyl-tRNA synthetase, alpha subunit	pheS
SAUSA300_1843	**0,9**	0,27951412	No	1078	1092	984	1339	1271	1111	1359	1394	1392	D-isomer specific 2-hydroxyacid dehydrogenase family p	SAUSA300_1843
SAUSA300_0905	**0,9**	0,549433772	No	313	361	350	325	316	216	300	333	322	putative adenylate cyclase	SAUSA300_0905
SAUSA300_0871	**0,9**	0,22823187	No	8713	8461	8447	9976	10620	8729	10832	11643	10224	conserved hypothetical protein	SAUSA300_0871
SAUSA300_2266	**0,9**	0,489676245	No	723	491	646	663	665	566	767	718	628	conserved hypothetical protein	SAUSA300_2266
SAUSA300_2200	**0,9**	0,497888673	No	4485	5618	5984	7549	8700	9312	9480	10280	8803	30S ribosomal protein S19	rpsS
SAUSA300_1069	**0,9**	0,266192272	No	257	289	310	337	345	278	348	354	364	conserved hypothetical protein	SAUSA300_1069
SAUSA300_0885	**0,9**	0,366984755	No	2775	3584	4038	3930	4282	4384	4785	4839	4450	3-oxoacyl-(acyl-carrier-protein) synthase III	fabH
SAUSA300_2098	**0,9**	0,542100843	No	1104	1242	680	898	805	688	910	809	956	transcriptional repressor, ArsR family	arsR
SAUSA300_2250	**0,9**	0,502856053	No	3593	4096	3015	2125	2078	2354	2747	2344	2245	Na+/H+ antiporter NhaC	nhaC
SAUSA300_1558	**0,9**	0,295442958	No	625	795	769	629	568	522	638	613	670	5'-methylthioadenosine/S-adenosylhomocysteine nucle	mtnN
SAUSA300_2194	**0,9**	0,485166559	No	5167	6588	6754	7518	8173	8964	9357	9986	8246	50S ribosomal protein L14	rplN
SAUSA300_1736	**0,9**	0,299347293	No	727	568	555	513	618	538	659	640	561	conserved hypothetical protein	SAUSA300_1736
SAUSA300_0351	**0,9**	0,598271464	No	136	303	293	209	185	216	242	208	235	putative membrane protein	SAUSA300_0351
SAUSA300_1374	**0,9**	0,422718387	No	1419	1324	1201	1690	1479	1384	1723	1617	1761	conserved hypothetical protein	SAUSA300_1374
SAUSA300_1457	**0,9**	0,673213631	No	122	55	81	142	122	120	122	190	120	maltose operon transcriptional repressor	malR
SAUSA300_0475	**0,9**	0,41985509	No	8135	7129	6105	2717	2518	3388	3468	3413	2792	SpoVG protein	SAUSA300_0475
SAUSA300_0401	**0,9**	0,572663993	No	59	48	57	74	106	53	88	77	91	exotoxin	SAUSA300_0401
SAUSA300_0341	**0,9**	0,175350742	No	966	1077	1054	570	561	615	612	670	677	putative membrane protein	SAUSA300_0341
SAUSA300_0567	**0,9**	0,433008526	No	739	775	595	405	469	418	481	514	451	conserved hypothetical protein	SAUSA300_0567
SAUSA300_2601	**0,9**	0,705919769	No	24	40	37	26	23	31	26	34	31	intercellular adhesion protein B	icaB
SAUSA300_1675	**0,9**	0,134381503	No	2081	1999	2155	1968	2044	1906	2013	2481	2134	tyrosyl-tRNA synthetase	tyrS
SAUSA300_2282	**0,9**	0,192535012	No	7461	7100	5799	4717	5141	4861	5347	5283	5863	putative membrane protein	SAUSA300_2282
SAUSA300_2380	**0,9**	0,541048034	No	148	205	167	137	119	155	178	149	140	conserved hypothetical protein	SAUSA300_2380
SAUSA300_0709	**0,9**	0,290998203	No	999	793	882	918	978	762	1019	1022	938	5'(3')-deoxyribonucleotidase	SAUSA300_0709
SAUSA300_2072	**0,9**	0,16544291	No	4204	3779	3590	2791	2844	2853	3201	3267	3067	peptide chain release factor 1	prfA
SAUSA300_1427	**0,9**	0,873817392	No	0	1	2	1	0	1	1	2	2	phiSLT ORF86-like protein	SAUSA300_1427
SAUSA300_1260	**0,9**	0,262014545	No	971	991	838	511	658	544	603	688	625	prephenate dehydrogenase	SAUSA300_1260
SAUSA300_1872	**0,9**	0,04149272	No	1211	1232	1278	1574	1612	1384	1692	1751	1680	conserved hypothetical protein	SAUSA300_1872
SAUSA300_0855	**0,9**	0,099169594	No	6493	6457	6878	6148	6428	5974	6614	7144	7085	Na(+)/H(+) antiporter subunit A	mnhA
SAUSA300_1914	**0,9**	0,460227669	No	512	665	565	481	433	430	497	474	544	GntR family regulatory protein	SAUSA300_1914
SAUSA300_0989	**0,9**	0,132291284	No	17568	15788	15096	14889	14707	13078	15726	17075	15192	conserved hypothetical protein	SAUSA300_0989
SAUSA300_2351	**0,9**	0,484252176	No	1044	1168	863	818	721	847	1008	963	720	Zn-binding lipoprotein adcA-like protein	SAUSA300_2351
SAUSA300_2193	**0,9**	0,438143185	No	5348	6911	6872	7510	7992	8748	9193	9614	8506	50S ribosomal protein L24	rplX
SAUSA300_2378	**0,9**	0,247855183	No	754	860	890	852	784	893	907	958	987	conserved hypothetical protein	SAUSA300_2378
SAUSA300_0414	**0,9**	0,697993895	No	7	12	19	39	16	25	30	28	35	staphylococcal tandem lipoprotein	SAUSA300_0414
SAUSA300_0203	**0,9**	0,413050123	No	348	459	319	190	160	213	218	178	243	putative lipoprotein	SAUSA300_0203
SAUSA300_0999	**0,9**	0,521484759	No	379	508	452	345	327	364	357	344	468	spermidine/putrescine ABC transporter, ATP-binding pr	potA
SAUSA300_1411	**0,9**	0,849849901	No	0	0	2	0	0	0	0	1	0	phiSLT ORF66-like protein	SAUSA300_1411
SAUSA300_0404	**0,9**	0,627153492	No	52	47	56	40	36	37	37	46	45	exotoxin	SAUSA300_0404
SAUSA300_1731	**0,9**	0,425085817	No	303	263	198	347	340	348	319	477	372	phosphoenolpyruvate carboxykinase (ATP)	pckA
SAUSA300_0565	**0,9**	0,251176068	No	341	409	428	311	302	297	358	331	338	conserved hypothetical protein	SAUSA300_0565
SAUSA300_0646	**0,9**	0,165986741	No	1044	920	844	985	965	929	1137	1092	1015	sensor histidine kinase	SAUSA300_0646
SAUSA300_0531	**0,9**	0,274246623	No	25076	26594	23828	23380	24126	24388	26379	29894	24818	30S ribosomal protein S7	SAUSA300_0531
SAUSA300_1610	**0,9**	0,078784569	No	3908	4204	4371	3331	3636	3187	3573	3949	3918	folylpolyglutamate synthase	folC
SAUSA300_1997	**0,9**	0,372929739	No	532	569	461	564	471	645	593	669	641	conserved hypothetical protein	SAUSA300_1997
SAUSA300_1450	**0,9**	0,261803427	No	2084	1936	1828	1965	1751	1752	2105	2076	1998	oxidoreductase, aldo/keto reductase family	SAUSA300_1450
SAUSA300_0599	**0,9**	0,3616372	No	439	494	515	364	449	396	412	506	442	iron compound ABC transporter, permease protein	SAUSA300_0599
SAUSA300_1523	**0,9**	0,142751287	No	996	1154	1003	1057	935	954	1087	1121	1127	conserved hypothetical protein	SAUSA300_1523

SAUSA300_2444	**0,9**	0,675947481	No	45	81	70	80	81	67	69	108	83	gluconate operon transcriptional repressor	gntR
SAUSA300_0966	**0,9**	0,667178177	No	203	719	299	441	291	535	503	447	496	phosphoribosylaminoimidazole carboxylase, catalytic su	purE
SAUSA300_0195	**0,9**	0,522199819	No	79	78	77	53	43	47	48	65	53	RpiR family transcriptional regulator	SAUSA300_0195
SAUSA300_1273	**0,9**	0,31992662	No	245	249	231	284	252	241	303	265	313	oligopeptide permease, ATP-binding protein	opp-2F
SAUSA300_1585	**0,9**	0,217535401	No	1662	1369	1279	1249	1165	1090	1321	1291	1358	conserved hypothetical protein	SAUSA300_1585
SAUSA300_0255	**0,9**	0,148972253	No	782	832	793	547	491	481	583	554	586	sensory transduction protein LytR	SAUSA300_0255
SAUSA300_1234	**0,9**	0,331393175	No	184	152	133	163	131	142	173	161	166	30S ribosomal protein S14-2	rpmN
SAUSA300_2081	**0,9**	0,033790149	No	3942	3935	3868	6033	6545	6305	7154	7278	6962	CTP synthase	pyrG
SAUSA300_1684	**0,9**	0,325677296	No	12601	11486	8889	4859	4100	5399	5840	5288	5170	conserved hypothetical protein	SAUSA300_1684
SAUSA300_1517	**0,9**	0,016305927	No	2539	2432	2506	1661	1738	1709	1918	1950	1926	endonuclease IV	SAUSA300_1517
SAUSA300_1852	**0,9**	0,172501307	No	1340	1452	1360	1266	1251	1053	1317	1372	1363	putative ABC transporter, ATP-binding protein	SAUSA300_1852
SAUSA300_0472	**0,9**	0,146870705	No	3016	2898	3145	1948	1880	1840	2090	2164	2183	4-diphosphocytidyl-2C-methyl-D-erythritol kinase	ispE
SAUSA300_0307	**0,9**	0,483793242	No	4907	4873	3772	567	401	503	606	538	537	5'-nucleotidase, lipoprotein e(P4) family	SAUSA300_0307
SAUSA300_1381	**0,9**	0,359344868	No	184	205	209	202	174	152	197	185	219	Panton-Valentine leukocidin, LukF-PV	lukF-PV
SAUSA300_1135	**0,9**	0,3775698	No	450	385	501	427	426	402	433	453	541	putative membrane protein	SAUSA300_1135
SAUSA300_0961	**0,9**	0,069374477	No	17206	17521	17151	9369	8274	8398	9276	10510	9825	quinol oxidase, subunit III	qoxC
SAUSA300_0763	**0,9**	0,066181616	No	1006	1076	1125	1282	1280	1207	1513	1441	1332	carboxylesterase	est
SAUSA300_1216	**0,9**	0,249041556	No	1154	1033	1294	1240	1359	1164	1431	1451	1399	cardiolipin synthetase	SAUSA300_1216
SAUSA300_2064	**0,9**	0,228942909	No	7478	8830	8057	8173	8107	7490	9283	9482	8308	ATP synthase F0, A subunit	atpB
SAUSA300_0849	**0,9**	0,266016913	No	1252	1118	1122	1033	1129	940	1171	1184	1175	Na(+)/H(+) antiporter subunit G	mnhG
SAUSA300_2020	**0,9**	0,160945944	No	1132	1051	938	812	782	660	884	901	782	metallopeptidase, SprT family	SAUSA300_2020
SAUSA300_2394	**0,9**	0,166975502	No	575	501	604	985	974	884	1084	1140	1016	conserved hypothetical protein	SAUSA300_2394
SAUSA300_1342	**0,9**	0,572663993	No	264	190	217	235	223	167	256	182	275	conserved hypothetical protein	SAUSA300_1342
SAUSA300_1222	**0,9**	0,332092897	No	181	167	191	129	158	161	179	155	174	thermonuclease	nuc
SAUSA300_0013	**0,9**	0,049893266	No	2628	2356	2294	2863	3128	2906	3408	3404	3327	putative membrane protein	SAUSA300_0013
SAUSA300_0513	**0,9**	0,041618013	No	9516	9971	8433	6401	6151	6356	7599	7306	6681	glutamyl-tRNA synthetase	gltX
SAUSA300_2419	**0,9**	0,320131363	No	289	204	186	213	255	198	262	225	268	conserved hypothetical protein	SAUSA300_2419
SAUSA300_1881	**0,9**	0,018359687	No	11381	11449	10973	9488	10028	8954	11039	10983	10489	Aspartyl/glutamyl-tRNA amidotransferase subunit A	gatA
SAUSA300_2172	**0,9**	0,249041556	No	15911	15735	14301	14577	15315	15154	17453	18277	15796	50S ribosomal protein L13	rplM
SAUSA300_1405	**0,9**	0,853993665	No	3	4	0	3	0	1	1	2	2	phiSLT ORF 101-like protein, terminase, small subunit	SAUSA300_1405
SAUSA300_1160	**0,9**	0,091708478	No	959	1014	925	961	962	843	1010	1065	1087	conserved hypothetical protein	SAUSA300_1160
SAUSA300_1231	**0,9**	0,223751272	No	10756	11538	8918	3297	3135	3370	3796	3752	3686	gamma-aminobutyrate permease	SAUSA300_1231
SAUSA300_0002	**0,9**	0,043007172	No	6371	6175	5579	5148	5362	4732	6008	5734	5728	DNA polymerase III, beta subunit	dnaN
SAUSA300_1073	**0,9**	0,165986741	No	14506	13912	12496	13235	12552	13352	14578	16942	13495	S-adenosyl-methyltransferase MraW	mraW
SAUSA300_1438	**0,9**	0,483793242	No	189	182	137	120	77	101	132	102	114	phiSLT ORF401-like protein, integrase	SAUSA300_1438
SAUSA300_1518	**0,9**	0,009373568	Yes	4061	3914	3871	2543	2572	2505	3089	2816	2858	ATP-dependent RNA helicase, DEAD/DEAH box family	SAUSA300_1518
SAUSA300_2097	**0,9**	0,490443653	No	574	775	791	377	298	566	506	487	447	conserved hypothetical protein	SAUSA300_2097
SAUSA300_2455	**0,9**	0,297675118	No	270	238	208	444	367	421	421	596	410	putative fructose-1,6-bisphosphatase	SAUSA300_2455
SAUSA300_1759	**0,9**	0,67593481	No	53	42	27	13	20	22	27	19	19	conserved hypothetical protein	SAUSA300_1759
SAUSA300_0145	**0,9**	0,52714916	No	74	85	95	37	45	31	41	49	41	phosphonate ABC transporter, phosphonate-binding pro	SAUSA300_0145
SAUSA300_0008	**0,9**	0,434129905	No	88	79	84	113	144	152	116	180	177	histidine ammonia-lyase	hutH
SAUSA300_2634	**0,9**	0,538697978	No	67	38	69	49	65	28	55	50	55	ABC transporter, permease protein	SAUSA300_2634
SAUSA300_1011	**0,9**	0,192535012	No	927	812	886	710	672	525	754	738	705	conserved hypothetical protein	SAUSA300_1011
SAUSA300_0924	**0,9**	0,044866135	No	2358	2190	2204	1833	1947	1674	2087	2064	2124	sodium transport family protein	SAUSA300_0924
SAUSA300_2204	**0,9**	0,255357711	No	12950	14395	15253	17646	18804	20537	22882	23888	19039	50S ribosomal protein L3	rplC
SAUSA300_0431	**0,9**	0,743260807	No	2	4	0	9	9	16	11	18	11	conserved hypothetical protein	SAUSA300_0431
SAUSA300_2104	**0,9**	0,370187706	No	11381	9897	9062	19292	17206	19208	21580	22394	20475	glucosamine--fructose-6-phosphate aminotransferase (i	glmS
SAUSA300_0217	**0,9**	0,198163656	No	417	424	354	301	309	275	329	381	311	DNA-binding response regulator, AraC family	SAUSA300_0217
SAUSA300_2151	**0,9**	0,75543405	No	5	4	7	7	14	15	12	9	19	PTS system, lactose-specific IIA component	lacF
SAUSA300_1319	**0,9**	0,175102138	No	2031	2070	1640	1153	1089	1066	1309	1300	1219	dihydrofolate reductase	folA
SAUSA300_1856	**0,9**	0,431856173	No	5226	5091	3562	2383	2017	2864	3023	2908	2501	conserved hypothetical protein	SAUSA300_1856
SAUSA300_0757	**0,9**	0,516868174	No	7663	10736	12430	8477	8684	9143	9196	12217	9144	phosphoglycerate kinase	pgk
SAUSA300_0713	**0,9**	0,195284455	No	675	623	562	447	554	492	594	570	559	GTP cyclohydrolase I	folE
SAUSA300_1189	**0,9**	0,08451288	No	1779	1785	1794	2175	2511	2112	2615	2485	2764	DNA mismatch repair protein mutL	mutL
SAUSA300_2217	**0,9**	0,192535012	No	315	335	264	274	248	209	297	276	273	putative drug transporter	SAUSA300_2217
SAUSA300_0400	**0,9**	0,598271464	No	14	15	20	21	29	25	31	28	28	exotoxin	SAUSA300_0400
SAUSA300_1338	**0,9**	0,336343	No	3171	3092	3142	3066	2815	2600	3251	3094	3505	conserved hypothetical protein	SAUSA300_1338
SAUSA300_2036	**0,9**	0,364280165	No	179	207	153	127	92	109	155	108	126	DNA-binding response regulator, KdpE	kdpE
SAUSA300_1553	**0,9**	0,066721081	No	1738	2053	1954	1481	1537	1461	1691	1769	1735	nicotinate (nicotinamide) nucleotide adenylyltransferas	nadD

SAUSA300_1254	**0,9**	0,185261614	No	4755	4350	4037	4266	3868	3558	4518	4332	4733	putative membrane protein	SAUSA300_1254
SAUSA300_1620	**0,9**	0,098667758	No	467	474	415	591	480	473	580	627	594	probable GTP-binding protein engB	SAUSA300_1620
SAUSA300_0696	**0,9**	0,334789066	No	444	446	422	229	376	216	282	344	316	6-pyruvoyl tetrahydrobiopterin synthase-like protein	SAUSA300_0696
SAUSA300_0202	**0,9**	0,461974335	No	179	267	151	87	65	78	107	82	84	peptide ABC transporter, permease protein	SAUSA300_0202
SAUSA300_2396	**0,9**	0,162858369	No	1269	1467	1117	600	557	593	690	701	650	para-nitrobenzyl esterase	pnbA
SAUSA300_0435	**0,9**	0,476597835	No	406	794	546	155	149	163	146	210	189	ABC transporter, ATP-binding protein	SAUSA300_0435
SAUSA300_2275	**0,9**	0,414916899	No	1740	2172	2094	763	669	1128	1041	1012	941	oxidoreductase, short chain dehydrogenase/reductase f	SAUSA300_2275
SAUSA300_0631	**0,9**	0,017652838	No	4562	4917	5024	2297	2227	2271	2645	2784	2482	putative nucleoside transporter	SAUSA300_0631
SAUSA300_2161	**0,9**	0,295188961	No	308	259	246	165	178	114	160	179	187	Hyaluronate lyase precursor	hysA
SAUSA300_1442	**0,9**	0,440473193	No	1178	1155	1945	1171	1163	1253	1261	1649	1283	staphylococcal respiratory response protein, SrrA	srrA
SAUSA300_2037	**0,9**	0,314045822	No	32489	19399	25932	21275	24820	21235	28111	26712	23752	ATP-dependent RNA helicase	SAUSA300_2037
SAUSA300_1673	**0,9**	0,151273066	No	952	1028	1002	566	563	642	603	665	799	1-acyl-sn-glycerol-3-phosphate acyltransferases	SAUSA300_1673
SAUSA300_1589	**0,9**	0,026182246	No	2441	2463	2263	1684	1618	1593	1930	1867	1915	D-tyrosyl-tRNA (Tyr) deacylase	dtd
SAUSA300_0386	**0,9**	0,314164199	No	9402	11668	9540	3716	3829	3697	4695	4468	3972	xanthine phosphoribosyltransferase	xpt
SAUSA300_1519	**0,9**	0,038446909	No	1645	1722	1664	1242	1298	1054	1397	1356	1431	conserved hypothetical protein	SAUSA300_1519
SAUSA300_0632	**0,9**	0,16101659	No	699	897	712	776	649	731	935	824	767	conserved hypothetical protein	SAUSA300_0632
SAUSA300_1284	**0,9**	0,148890876	No	5954	4428	4667	3272	3079	3037	3782	3847	3353	conserved hypothetical protein	SAUSA300_1284
SAUSA300_1310	**0,9**	0,166947898	No	925	1240	1131	756	809	674	893	866	855	PAP2 family protein	SAUSA300_1310
SAUSA300_0139	**0,9**	0,335816974	No	110	136	133	115	140	121	148	137	152	putative tetracycline resistance protein	SAUSA300_0139
SAUSA300_0218	**0,9**	0,056797552	No	608	667	581	440	451	396	471	533	496	sensor histidine kinase family protein	SAUSA300_0218
SAUSA300_1421	**0,9**	0,818401552	No	0	0	0	0	0	0	1	0	0	phiSLT ORF122-like protein, DNA polymerase	SAUSA300_1421
SAUSA300_0571	**0,9**	0,092901699	No	6831	6465	5554	4024	3532	3372	4366	4169	4260	lipoate-protein ligase A family protein	SAUSA300_0571
SAUSA300_1158	**0,9**	0,010281462	No	1786	1844	1574	1764	1677	1604	2032	1986	1893	conserved hypothetical protein	SAUSA300_1158
SAUSA300_2177	**0,9**	0,130081582	No	5737	6275	5506	5396	5587	5288	6593	6721	5752	50S ribosomal protein L17	rplQ
SAUSA300_0797	**0,9**	0,442288763	No	1059	1767	1134	296	273	321	373	383	292	ABC transporter permease protein	SAUSA300_0797
SAUSA300_0321	**0,9**	0,259612306	No	847	820	755	692	766	612	804	849	771	conserved hypothetical protein	SAUSA300_0321
SAUSA300_1907	**0,9**	0,154297573	No	660	601	658	468	482	384	493	487	581	conserved hypothetical protein	SAUSA300_1907
SAUSA300_0174	**0,9**	0,393087666	No	3142	4287	2917	2170	1889	2082	2127	2319	2779	conserved hypothetical protein	SAUSA300_0174
SAUSA300_1201	**0,9**	0,330082595	No	13860	16753	11483	13124	12973	13801	17325	14642	14946	glutamine synthetase, type I	glnA
SAUSA300_1079	**0,9**	0,013049172	No	14140	16497	16122	14437	14441	12949	16693	16498	15922	cell division protein ftsA	ftsA
SAUSA300_0888	**0,9**	0,52714916	No	196	334	412	84	90	138	118	130	123	oligopeptide ABC transporter, permease protein	oppC
SAUSA300_1703	**0,9**	0,316077359	No	257	301	370	284	257	359	375	322	367	rhodanese-like domain protein	SAUSA300_1703
SAUSA300_1285	**0,9**	0,03856272	No	5136	5338	5013	2883	3074	2816	3492	3482	3343	ABC transporter, ATP-binding protein	SAUSA300_1285
SAUSA300_1127	**0,8**	0,214529991	No	1690	2322	2470	2505	2303	2492	2816	2675	3126	chromosome segregation protein SMC	smc
SAUSA300_1530	**0,8**	0,042777049	No	797	875	793	748	748	683	829	880	858	conserved hypothetical protein	SAUSA300_1530
SAUSA300_1077	**0,8**	0,00025227	Yes	3278	3488	3416	2860	2874	2978	3268	3423	3574	UDP-N-acetylmuramoylalanine--D-glutamate ligase	murD
SAUSA300_1763	**0,8**	0,332092897	No	172	212	160	109	88	98	120	109	122	lantibiotic epidermin leader peptide processing serine p	epiP
SAUSA300_1875	**0,8**	0,322058981	No	605	461	464	630	694	473	701	644	775	exonuclease	SAUSA300_1875
SAUSA300_1724	**0,8**	0,630378134	No	34	29	33	21	16	13	22	15	23	abortive infection protein family	SAUSA300_1724
SAUSA300_0707	**0,8**	0,056879214	No	2954	2280	2402	3496	3388	2866	3935	3666	3894	osmoprotectant ABC transporter, permease	SAUSA300_0707
SAUSA300_0358	**0,8**	0,424556048	No	549	782	410	130	110	149	181	150	135	putative 5-methyltetrahydrofolate--homocysteine meth	SAUSA300_0358
SAUSA300_1510	**0,8**	0,227324823	No	205	271	268	216	147	149	186	199	227	5-formyltetrahydrofolate cyclo-ligase subfamily	SAUSA300_1510
SAUSA300_2495	**0,8**	0,200580432	No	873	602	629	1701	1936	1755	2243	2051	2084	copper chaperone copZ	SAUSA300_2495
SAUSA300_0874	**0,8**	0,089130074	No	1469	1168	1414	1452	1553	1289	1646	1669	1757	conserved hypothetical protein	SAUSA300_0874
SAUSA300_1655	**0,8**	0,112620258	No	288	282	266	332	325	364	373	448	391	alanine dehydrogenase	ald
SAUSA300_1409	**0,8**	0,708547079	No	14	12	14	1	11	12	10	6	13	conserved hypothetical phage protein	SAUSA300_1409
SAUSA300_1972	**0,8**	0,298504663	No	122	159	92	137	135	135	175	147	163	integrase	int
SAUSA300_1025	**0,8**	0,050605935	No	505	391	391	488	485	421	571	548	532	conserved hypothetical protein	SAUSA300_1025
SAUSA300_2053	**0,8**	0,151497459	No	231	207	308	371	460	373	493	501	429	conserved hypothetical protein	SAUSA300_2053
SAUSA300_1882	**0,8**	0,012331277	No	3422	3621	3233	3321	3498	3281	4246	4114	3624	aspartyl/glutamyl-tRNA amidotransferase subunit C	gatC
SAUSA300_0755	**0,8**	0,67766011	No	19786	21863	33063	18136	17549	22091	17753	34544	17651	glycolytic operon regulator	SAUSA300_0755
SAUSA300_1262	**0,8**	0,475869016	No	69	79	53	60	56	49	47	70	78	anthranilate synthase component I	trpE
SAUSA300_1361	**0,8**	0,141686019	No	484	498	623	427	482	402	557	496	502	conserved hypothetical protein	SAUSA300_1361
SAUSA300_1916	**0,8**	0,019208241	No	7503	7969	6836	5280	5049	4531	5819	6214	5630	aminotransferase	SAUSA300_1916
SAUSA300_0417	**0,8**	0,420897718	No	21	37	36	50	45	55	65	58	58	staphylococcal tandem lipoprotein	SAUSA300_0417
SAUSA300_2547	**0,8**	0,332092897	No	954	1007	736	980	802	985	1200	1037	1068	conserved hypothetical protein	SAUSA300_2547
SAUSA300_2476	**0,8**	0,235948404	No	1094	1358	1336	2011	1916	2314	2316	3074	2056	phosphotransferase system, glucose-specific IIABC com	ptsG
SAUSA300_0287	**0,8**	0,372929739	No	78	90	82	107	97	142	147	129	140	conserved hypothetical protein	SAUSA300_0287
SAUSA300_0627	**0,8**	0,032687832	No	761	917	968	1283	1233	1162	1392	1538	1453	teichoic acid biosynthesis protein X	tagX

SAUSA300_1412	**0,8**	0,783036377	No	3	4	2	1	0	1	1	2	3	phiSLT ORF 50-like protein	SAUSA300_1412
SAUSA300_1884	**0,8**	0,013196217	No	3303	3263	3242	3390	3618	2814	3999	3873	3842	CamS sex pheromone cAM373 precursor	SAUSA300_1884
SAUSA300_0606	**0,8**	0,165986741	No	195	242	184	160	183	145	206	186	187	putative membrane protein	SAUSA300_0606
SAUSA300_1574	**0,8**	0,014096716	No	2112	2150	2207	1688	1521	1340	1905	1826	1708	conserved hypothetical protein	SAUSA300_1574
SAUSA300_0357	**0,8**	0,095786401	No	1200	1373	1006	342	356	313	431	402	377	5-methyltetrahydropteroyltriglutamate--homocysteine	metE
SAUSA300_1219	**0,8**	0,270943174	No	186	149	165	127	128	96	144	132	145	putative sensor histidine kinase	SAUSA300_1219
SAUSA300_0538	**0,8**	0,105211364	No	1400	1399	1262	1141	1050	1177	1208	1691	1148	NAD dependent epimerase/dehydratase family	SAUSA300_0538
SAUSA300_2085	**0,8**	0,022607422	No	2823	3254	2592	2281	2195	2094	2732	2692	2457	conserved hypothetical protein	SAUSA300_2085
SAUSA300_2185	**0,8**	0,145513179	No	5653	6835	6656	7387	7627	8917	9834	10271	8634	50S ribosomal protein L15	rplO
SAUSA300_0768	**0,8**	0,353188262	No	1381	835	712	593	527	469	779	594	541	conserved hypothetical protein	SAUSA300_0768
SAUSA300_1309	**0,8**	0,241189278	No	315	238	284	202	216	161	242	212	241	transposase, IS200 family	SAUSA300_1309
SAUSA300_0096	**0,8**	0,265577541	No	59	60	72	83	119	101	118	118	124	conserved hypothetical protein	SAUSA300_0096
SAUSA300_2617	**0,8**	0,167549189	No	350	487	399	162	180	163	187	201	217	putative cobalt ABC transporter, ATP-binding protein	SAUSA300_2617
SAUSA300_1101	**0,8**	0,078946807	No	2937	2331	2064	2230	2171	1856	2653	2414	2467	putative fibronectin/fibrinogen binding protein	SAUSA300_1101
SAUSA300_1286	**0,8**	0,317835678	No	417	631	494	158	144	158	160	197	201	aspartate kinase	SAUSA300_1286
SAUSA300_1108	**0,8**	0,016173705	No	816	802	816	531	536	568	688	639	646	polypeptide deformylase	def
SAUSA300_0776	**0,8**	0,345963183	No	4352	2888	2286	677	449	529	730	640	639	thermonuclease precursor	nuc
SAUSA300_1038	**0,8**	0,006108656	Yes	6261	6579	5748	4545	4330	4064	5186	5339	5075	phenylalanyl-tRNA synthetase, beta subunit	pheT
SAUSA300_0427	**0,8**	0,023113124	No	846	834	1066	856	922	790	992	1056	1041	conserved hypothetical protein	SAUSA300_0427
SAUSA300_0132	**0,8**	0,155452612	No	176	186	190	149	144	139	168	170	183	glycosyl transferase, group 1 family protein	SAUSA300_0132
SAUSA300_2294	**0,8**	0,083532392	No	339	318	287	463	471	376	494	534	549	conserved hypothetical protein	SAUSA300_2294
SAUSA300_2431	**0,8**	0,137503328	No	1054	1031	1159	779	751	698	860	807	1024	putative helicase	SAUSA300_2431
SAUSA300_1896	**0,8**	0,069374477	No	1009	913	1012	573	566	521	635	659	712	prephenate dehydratase	pheA
SAUSA300_1172	**0,8**	0,001165555	Yes	2311	2313	2323	1767	1857	1644	2161	1979	2220	peptidase, M16 family	SAUSA300_1172
SAUSA300_1496	**0,8**	0,007997014	Yes	2609	2757	2482	2648	2784	2816	3228	3560	3197	glycine dehydrogenase, subunit 2	SAUSA300_1496
SAUSA300_2575	**0,8**	0,236196283	No	296	308	238	211	176	176	201	213	269	transcriptional antiterminator, BglG family	SAUSA300_2575
SAUSA300_0437	**0,8**	0,104739908	No	934	1354	1090	388	298	340	426	428	400	NLPA lipoprotein	SAUSA300_0437
SAUSA300_2199	**0,8**	0,206972357	No	4447	5158	5946	6066	6252	7345	7664	8360	7910	50S ribosomal protein L22	rplV
SAUSA300_1761	**0,8**	0,444858845	No	36	30	58	21	18	37	31	32	35	lantibiotic epidermin immunity protein F	epiE
SAUSA300_1340	**0,8**	0,000138488	Yes	2854	2762	2738	3185	3403	3058	3864	3971	3876	recombination protein U	recU
SAUSA300_1430	**0,8**	0,753057108	No	2	3	0	3	2	4	2	5	5	phiSLT ORF 87-like protein, putative DNA-binding protei	SAUSA300_1430
SAUSA300_0257	**0,8**	0,315807226	No	460	645	707	178	234	185	218	320	190	Antiholin-like protein lrgB	SAUSA300_0257
SAUSA300_0352	**0,8**	0,126161185	No	394	558	489	369	365	364	458	421	461	ABC transporter, ATP-binding protein	SAUSA300_0352
SAUSA300_1118	**0,8**	0,000187044	Yes	1459	1485	1497	1552	1537	1541	1993	1803	1851	conserved hypothetical protein	SAUSA300_1118
SAUSA300_0103	**0,8**	0,272894439	No	114	105	85	153	137	132	182	155	180	staphylococcal tandem lipoprotein	SAUSA300_0103
SAUSA300_1593	**0,8**	0,003110326	Yes	11853	10499	10147	7632	7873	6838	9009	8824	9419	protein-export membrane protein SecF	secF
SAUSA300_1435	**0,8**	0,299103897	No	899	1059	725	609	485	458	714	586	606	phiSLT ORF153-like protein	SAUSA300_1435
SAUSA300_2251	**0,8**	0,211180597	No	3204	3859	3073	1953	1941	1940	2519	2448	2189	dehydrogenase family protein	SAUSA300_2251
SAUSA300_1380	**0,8**	0,631239242	No	12	3	4	13	18	9	15	16	16	conserved hypothetical protein	SAUSA300_1380
SAUSA300_1441	**0,8**	0,332675828	No	1381	1893	2451	1445	1323	1275	1489	2016	1466	staphylococcal respiratory response protein, SrrB	srrB
SAUSA300_1226	**0,8**	0,125888554	No	1080	1543	1525	601	557	640	673	748	786	homoserine dehydrogenase	SAUSA300_1226
SAUSA300_1279	**0,8**	0,331393175	No	86	79	88	33	61	47	59	52	59	phosphate transport system regulatory protein PhoU	phoU
SAUSA300_0654	**0,8**	0,699836727	No	14	18	41	21	29	34	35	26	45	staphylococcal accessory protein X	sarX
SAUSA300_2082	**0,8**	0,015238201	No	3303	3339	3559	2913	2883	2572	3660	3383	3209	DNA-directed RNA polymerase, delta subunit	rpoE
SAUSA300_2178	**0,8**	0,06214081	No	18539	21458	17789	19572	19541	19159	24193	25650	21653	DNA-directed RNA polymerase alpha subunit	rpoA
SAUSA300_2509	**0,8**	0,078482557	No	257	226	247	223	174	206	221	243	280	transcriptional regulator, TetR family	SAUSA300_2509
SAUSA300_0463	**0,8**	0,024359376	No	687	798	776	506	543	469	671	627	560	conserved hypothetical protein	SAUSA300_0463
SAUSA300_1347	**0,8**	0,052771418	No	851	865	861	988	983	735	1073	1033	1210	BirA bifunctional protein	birA
SAUSA300_1561	**0,8**	0,073514104	No	162	145	134	212	167	163	223	210	236	putative membrane protein	SAUSA300_1561
SAUSA300_1191	**0,8**	0,301487141	No	295	257	303	236	250	248	308	353	246	glycerol uptake facilitator	glpF
SAUSA300_0091	**0,8**	0,498029514	No	40	27	36	17	45	15	29	31	30	putative permease	SAUSA300_0091
SAUSA300_2337	**0,8**	0,028115266	No	1028	1057	782	580	566	620	741	737	694	transcriptional regulator, DegU family	SAUSA300_2337
SAUSA300_0699	**0,8**	0,071096707	No	446	515	396	337	322	312	390	411	394	chorismate binding enzyme domain protein	SAUSA300_0699
SAUSA300_2188	**0,8**	0,117473546	No	5713	7373	7409	7696	7762	8058	9705	10118	9133	50S ribosomal protein L18	rplR
SAUSA300_0983	**0,8**	0,011163572	No	7652	7396	6222	5809	5698	5139	7349	7284	5844	phosphocarrier protein HPr	ptsH
SAUSA300_2359	**0,8**	0,002366614	Yes	11493	12430	11258	6960	7084	6459	8334	8422	8465	amino acid ABC transporter, amino acid-binding protein	SAUSA300_2359
SAUSA300_1612	**0,8**	0,013528495	No	1121	1139	1097	752	760	736	1000	930	840	DNA-3-methyladenine glycosidase	tag
SAUSA300_1498	**0,8**	0,116940504	No	592	935	855	808	978	868	1011	1203	1056	aminomethyltransferase (glycine cleavage system T pro	gcvT
SAUSA300_2230	**0,8**	0,014471516	No	837	812	964	649	624	552	722	737	788	molybdenum ABC transporter, molybdenum-binding pr	modA

SAUSA300_1395	**0,8**	0,749361566	No	0	0	1	1	0	1	2	3	1	phiSLT ORF116b-like protein	SAUSA300_1395
SAUSA300_1805	**0,8**	0,021799414	No	424	465	441	262	246	269	324	315	323	RNA methyltransferase	SAUSA300_1805
SAUSA300_0300	**0,8**	0,465306395	No	5	29	40	44	56	64	72	62	70	conserved hypothetical protein	SAUSA300_0300
SAUSA300_1802	**0,8**	0,66470104	No	67	26	76	11	52	19	32	26	44	conserved hypothetical protein	SAUSA300_1802
SAUSA300_0176	**0,8**	0,156967656	No	2716	3308	2536	1800	1535	1782	2104	1981	2260	ABC transporter, permease protein	SAUSA300_0176
SAUSA300_1902	**0,8**	0,005829414	Yes	1798	1639	1635	1919	1857	1717	2297	2297	2200	conserved hypothetical protein	SAUSA300_1902
SAUSA300_1081	**0,8**	0,008208494	Yes	1059	1144	1077	652	636	565	709	764	816	conserved hypothetical protein	SAUSA300_1081
SAUSA300_0935	**0,8**	0,557349723	No	33	33	23	13	18	19	19	15	28	conserved hypothetical protein	SAUSA300_0935
SAUSA300_1601	**0,8**	0,044727443	No	8818	9006	9992	10295	10478	11876	13882	14234	12405	50S ribosomal protein L27	rpmA
SAUSA300_1490	**0,8**	0,049650985	No	13865	10585	10818	5350	4679	4356	6390	6124	5349	translation elongation factor P	efp
SAUSA300_2191	**0,8**	0,113602266	No	3262	4079	3962	4348	4224	4336	5451	5654	4939	30S ribosomal protein S14	rpsN
SAUSA300_1274	**0,8**	0,192070286	No	105	148	137	165	151	172	218	159	231	peptide ABC transporter, ATP-binding protein	SAUSA300_1274
SAUSA300_0595	**0,8**	0,0915334	No	637	954	860	513	390	544	605	590	613	conserved hypothetical protein	SAUSA300_0595
SAUSA300_1024	**0,8**	0,024506706	No	622	617	551	599	651	489	729	694	729	phosphopantetheine adenylyltransferase	coaD
SAUSA300_1161	**0,8**	0,015323358	No	885	908	917	799	834	725	1011	920	994	ribosomal protein L7Ae	SAUSA300_1161
SAUSA300_0684	**0,8**	0,194127278	No	603	850	1043	1289	1028	1417	1347	1871	1443	fructose 1-phosphate kinase	fruB
SAUSA300_0309	**0,8**	0,064781725	No	1388	1242	1166	882	771	1020	1087	1208	1041	ABC transporter ATP-binding protein	SAUSA300_0309
SAUSA300_0159	**0,8**	0,328715843	No	36	19	26	34	41	44	52	42	54	capsular polysaccharide biosynthesis protein Cap5H	cap5H
SAUSA300_2538	**0,8**	0,281344213	No	2151	1748	2167	874	931	938	1057	1416	953	amino acid permease family protein	SAUSA300_2538
SAUSA300_0644	**0,8**	0,027227894	No	1018	1054	950	822	879	688	902	943	1122	conserved hypothetical protein	SAUSA300_0644
SAUSA300_1694	**0,8**	0,01123473	No	3844	3236	3350	2079	2026	2119	2533	2516	2712	tRNA (guanine-N(7)-)-methyltransferase	trmB
SAUSA300_1677	**0,8**	0,02751805	No	27969	26602	23015	12736	13539	11304	15178	16314	15392	cell wall surface anchor family protein	SAUSA300_1677
SAUSA300_0962	**0,8**	0,005345972	Yes	53346	54280	52496	26582	22686	23521	28472	32452	29859	quinol oxidase, subunit I	qoxB
SAUSA300_2349	**0,8**	0,014032489	No	839	903	988	632	577	637	776	785	747	formate/nitrite transporter family protein	SAUSA300_2349
SAUSA300_2242	**0,8**	0,179214598	No	239	212	239	3055	2493	2356	3187	3147	3554	urease accessory protein UreF	ureF
SAUSA300_1662	**0,8**	0,035277539	No	410	435	460	382	430	379	474	514	494	aminotransferase, class V	SAUSA300_1662
SAUSA300_0211	**0,8**	0,380015503	No	34	45	42	49	32	19	38	44	44	maltose ABC transporter, permease protein	SAUSA300_0211
SAUSA300_0028	**0,8**	0,095929803	No	403	315	305	266	279	257	330	345	329	putative transposase	SAUSA300_0028
SAUSA300_0717	**0,8**	0,029915129	No	13624	11953	12266	8210	8977	7255	10019	10270	10341	ribonucleoside-diphosphate reductase, beta subunit	SAUSA300_0717
SAUSA300_0580	**0,8**	0,467440301	No	17	36	35	20	18	22	28	24	25	conserved hypothetical protein	SAUSA300_0580
SAUSA300_2246	**0,8**	0,098722097	No	636	517	508	470	428	447	641	536	515	conserved hypothetical protein	SAUSA300_2246
SAUSA300_1509	**0,8**	0,153285918	No	505	638	883	467	485	479	505	556	736	peptidase, rhomboid family	SAUSA300_1509
SAUSA300_2558	**0,8**	0,005464658	Yes	716	637	662	1061	1046	889	1219	1290	1243	sensor histidine kinase	SAUSA300_2558
SAUSA300_1305	**0,8**	0,003177217	Yes	1169	1418	1183	1233	1255	1267	1537	1668	1511	2-oxoglutarate dehydrogenase, E2 component, dihydrol	sucB
SAUSA300_1270	**0,8**	0,009497764	Yes	7508	6321	6718	6230	6728	5522	7529	7636	8038	methicillin resistance protein FemB	femB
SAUSA300_0903	**0,8**	0,048676746	No	1750	1395	1591	1855	1961	1550	2132	2230	2385	conserved hypothetical protein	SAUSA300_0903
SAUSA300_2143	**0,8**	0,119679627	No	10122	10688	9358	2853	2256	3823	4092	3801	3378	conserved hypothetical protein	SAUSA300_2143
SAUSA300_2604	**0,8**	0,591454984	No	26	12	12	7	14	12	16	15	11	conserved hypothetical protein	SAUSA300_2604
SAUSA300_0963	**0,8**	0,020488401	No	31741	29362	29377	13487	11858	12163	14237	16535	16463	quinol oxidase, subunit II	qoxA
SAUSA300_2279	**0,8**	0,298311727	No	100	116	77	87	81	58	86	102	97	LysR family regulatory protein	SAUSA300_2279
SAUSA300_0370	**0,8**	0,296367732	No	195	156	91	97	65	81	117	95	101	putative staphylococcal enterotoxin	SAUSA300_0370
SAUSA300_0302	**0,8**	0,152860699	No	86	57	84	72	79	114	118	105	114	conserved hypothetical protein	SAUSA300_0302
SAUSA300_1082	**0,8**	1,18E-05	Yes	4440	4038	4283	3274	3338	2994	4121	4088	3948	conserved hypothetical protein	SAUSA300_1082
SAUSA300_0591	**0,8**	0,108436856	No	281	385	443	259	248	214	317	283	316	acetyltransferase, GNAT family	SAUSA300_0591
SAUSA300_2334	**0,8**	0,378486966	No	21	36	32	16	27	31	34	29	31	conserved hypothetical protein	SAUSA300_2334
SAUSA300_1205	**0,8**	0,712239242	No	9	11	5	1	5	0	2	2	3	conserved hypothetical protein	SAUSA300_1205
SAUSA300_1602	**0,8**	0,018242985	No	4817	4634	5774	4961	4970	5523	6608	7105	5905	conserved hypothetical protein	SAUSA300_1602
SAUSA300_1861	**0,8**	0,023316088	No	172	215	225	274	243	256	320	330	334	conserved hypothetical protein	SAUSA300_1861
SAUSA300_1391	**0,8**	0,388371075	No	40	48	37	24	20	27	38	27	28	phiSLT ORF527-like protein	SAUSA300_1391
SAUSA300_1472	**0,8**	0,001796566	Yes	3390	3437	3535	2814	2835	2784	3559	3333	3820	exodeoxyribonuclease VII, large subunit	xseA
SAUSA300_1346	**0,8**	0,001687685	Yes	2606	2367	2605	2452	2547	2157	2966	2989	3128	putative DnaQ family exonuclease/DinG family helicase	SAUSA300_1346
SAUSA300_2298	**0,8**	0,176148916	No	2961	1983	2274	2555	2191	2123	3155	3183	2428	multidrug resistance protein B, drug resistance transpor	SAUSA300_2298
SAUSA300_0530	**0,8**	0,014862528	No	26731	27467	25289	22081	23977	23583	28974	31834	27802	ribosomal protein S12	rpsL
SAUSA300_1587	**0,8**	0,00220418	Yes	1738	2000	1792	1594	1661	1428	1913	2062	1975	histidyl-tRNA synthetase	hisS
SAUSA300_1292	**0,8**	0,01830641	No	536	565	576	170	192	172	216	219	239	alanine racemase	alr2
SAUSA300_0638	**0,8**	0,147044079	No	48	67	36	69	90	74	103	111	82	dihydroxyacetone kinase, phosphotransfer subunit	SAUSA300_0638
SAUSA300_1722	**0,8**	0,629477395	No	10	5	13	4	9	4	10	5	9	conserved hypothetical protein	SAUSA300_1722
SAUSA300_0868	**0,8**	0,002576382	Yes	1405	1105	1236	1488	1499	1255	1878	1733	1792	signal peptidase IB	spsB
SAUSA300_0691	**0,8**	0,167549189	No	16719	10309	8018	10272	10136	7760	12147	11378	12537	DNA-binding response regulator SaeR	saeR

SAUSA300_1478	**0,8**	0,313708527	No	91	88	131	109	110	104	119	133	163	putative lipoprotein	SAUSA300_1478
SAUSA300_2073	**0,8**	0,014094745	No	2530	2192	2041	1545	1503	1579	2100	2014	1801	thymidine kinase	tdk
SAUSA300_1121	**0,8**	0,008508283	Yes	1488	1363	1835	1840	2031	1701	2415	2337	2357	conserved hypothetical protein	SAUSA300_1121
SAUSA300_1557	**0,8**	0,066864117	No	622	939	863	563	600	553	704	690	799	hydrolase, HAD-superfamily, subfamily IIIA	SAUSA300_1557
SAUSA300_1163	**0,8**	0,062630702	No	1076	868	1260	1012	967	843	1201	1131	1280	ribosome-binding factor A	rbfA
SAUSA300_2297	**0,8**	0,00777196	Yes	1574	1294	1184	727	683	631	923	809	880	conserved hypothetical protein	SAUSA300_2297
SAUSA300_0304	**0,8**	0,229536825	No	944	463	648	640	489	565	751	610	821	conserved hypothetical protein	SAUSA300_0304
SAUSA300_2001	**0,8**	0,121902365	No	381	394	357	238	232	207	290	264	314	DNA mismatch repair protein-like protein	SAUSA300_2001
SAUSA300_0891	**0,8**	0,192070286	No	2573	3434	3116	699	775	1032	1060	1126	1041	oligopeptide ABC transporter, substrate-binding protein	oppA
SAUSA300_1027	**0,8**	0,008380151	Yes	6250	5344	5579	5796	5898	5250	7416	7231	7059	50S ribosomal protein L32	rpmF
SAUSA300_2113	**0,8**	0,009362768	Yes	2416	2721	2639	2124	1880	1693	2441	2388	2473	conserved hypothetical protein	SAUSA300_2113
SAUSA300_0046	**0,8**	0,046011065	No	2053	1428	1611	2281	2270	1990	3072	2873	2452	conserved hypothetical protein	SAUSA300_0046
SAUSA300_2022	**0,8**	0,081343094	No	11597	11010	9319	4825	4190	5671	6616	6478	5789	RNA polymerase sigma-37 factor	rpoF
SAUSA300_0032	**0,8**	0,001807012	Yes	130433	114846	118479	99940	107248	85166	125427	131781	117747	penicillin-binding protein 2'	mecA
SAUSA300_0209	**0,8**	0,30438339	No	45	77	71	66	45	59	64	90	69	putative maltose ABC transporter, maltose-binding prot	SAUSA300_0209
SAUSA300_2046	**0,8**	0,016319881	No	11932	8861	10436	12833	14290	11647	17470	17000	15329	membrane protein oxaA precursor	oxaA
SAUSA300_0177	**0,8**	0,148972253	No	5341	6190	4803	3307	2939	3233	3819	3853	4546	conserved hypothetical protein	SAUSA300_0177
SAUSA300_1740	**0,8**	0,022357377	No	2794	2690	2629	1585	1515	1482	1675	2415	1802	conserved hypothetical protein	SAUSA300_1740
SAUSA300_1106	**0,8**	0,30662715	No	1598	661	816	1094	929	762	1271	1182	1155	putative lipoprotein	SAUSA300_1106
SAUSA300_2096	**0,8**	0,000801876	Yes	2335	2130	2164	1456	1255	1406	1722	1823	1757	mannose-6-phosphate isomerase	manA
SAUSA300_2133	**0,8**	0,004847587	Yes	1192	1161	1336	1025	1289	1063	1430	1528	1367	transporter gate domain protein	SAUSA300_2133
SAUSA300_1148	**0,8**	0,001351045	Yes	2441	2385	2692	3155	3315	3027	3795	4169	4238	GTP-sensing transcriptional pleiotropic repressor CodY	codY
SAUSA300_1110	**0,8**	0,001026775	Yes	2463	2527	2685	1597	1889	1513	2147	2130	2131	ribosomal RNA small subunit methyltransferase B	sun
SAUSA300_0442	**0,8**	0,058932644	No	1180	1014	1162	1538	1686	1285	1893	2023	1887	YibE/F-like protein	SAUSA300_0442
SAUSA300_1140	**0,8**	0,291945072	No	74	79	92	66	79	70	65	75	135	cell wall hydrolase	lytN
SAUSA300_0890	**0,8**	0,212644639	No	630	1131	1084	281	300	322	368	413	389	oligopeptide ABC transporter, ATP-binding protein	oppF
SAUSA300_0210	**0,8**	0,285362229	No	78	99	85	70	47	87	85	109	76	maltose ABC transporter, permease protein	SAUSA300_0210
SAUSA300_2287	**0,8**	0,045166027	No	1450	1090	1161	848	769	702	958	950	1082	putative membrane protein	SAUSA300_2287
SAUSA300_0679	**0,8**	0,012223846	No	761	630	611	559	460	410	661	603	581	conserved hypothetical protein	SAUSA300_0679
SAUSA300_0687	**0,8**	0,000395151	Yes	4066	3612	4231	3918	4068	3364	4791	4991	4824	putative hemolysin	SAUSA300_0687
SAUSA300_0173	**0,8**	0,118257795	No	4669	5389	3723	2470	2044	2143	2731	2858	3017	conserved hypothetical protein	SAUSA300_0173
SAUSA300_1788	**0,8**	0,054185874	No	4695	3900	3507	1955	1859	2345	2781	2785	2394	conserved hypothetical protein	SAUSA300_1788
SAUSA300_0384	**0,8**	0,008508283	Yes	885	804	711	528	458	429	577	611	640	conserved hypothetical protein	SAUSA300_0384
SAUSA300_0933	**0,8**	0,589943945	No	45	81	228	83	110	130	100	117	220	conserved hypothetical protein	SAUSA300_0933
SAUSA300_1555	**0,8**	0,006286574	Yes	878	1075	1155	670	766	670	902	861	946	shikimate 5-dehydrogenase	aroE
SAUSA300_2398	**0,8**	0,142413004	No	971	757	727	302	277	358	447	433	339	putative membrane protein	SAUSA300_2398
SAUSA300_0420	**0,8**	0,069559026	No	911	909	909	1577	1237	1319	1867	1590	1894	conserved hypothetical protein	SAUSA300_0420
SAUSA300_0981	**0,8**	0,00107935	Yes	751	825	812	438	500	416	578	583	576	conserved hypothetical protein	SAUSA300_0981
SAUSA300_0920	**0,8**	0,016096903	No	363	461	427	327	361	345	430	454	445	conserved hypothetical protein	SAUSA300_0920
SAUSA300_1300	**0,8**	0,025114714	No	1976	1630	1799	1077	1269	855	1404	1301	1429	branched-chain amino acid transport system II carrier p	brnQ
SAUSA300_0518	**0,8**	0,01887128	No	756	879	840	799	764	695	915	1018	994	conserved hypothetical protein	SAUSA300_0518
SAUSA300_1497	**0,8**	0,002915861	Yes	1380	1745	1618	1703	1799	1726	2093	2428	2248	glycine dehydrogenase, subunit 1 (glycine cleavage syst	SAUSA300_1497
SAUSA300_0102	**0,8**	0,067968093	No	84	78	86	123	142	117	156	157	178	staphylococcal tandem lipoprotein	SAUSA300_0102
SAUSA300_2203	**0,8**	0,053339925	No	9156	9581	10775	10016	10512	12770	13904	15306	14020	50S ribosomal protein L4	rplD
SAUSA300_2244	**0,8**	0,063967368	No	293	256	323	2788	2804	2470	3190	3393	3911	urease accessory protein UreD	ureD
SAUSA300_0585	**0,8**	0,342334482	No	60	44	49	27	29	24	36	35	33	conserved hypothetical protein	SAUSA300_0585
SAUSA300_0782	**0,8**	0,103878127	No	785	565	574	269	268	250	381	368	278	conserved hypothetical protein	SAUSA300_0782
SAUSA300_0001	**0,8**	3,66E-07	Yes	7623	6914	6674	5028	4902	4702	6229	6540	6246	chromosomal replication initiator protein DnaA	dnaA
SAUSA300_1306	**0,8**	0,003807337	Yes	2167	2671	2136	2314	2013	2048	2597	3113	2593	2-oxoglutarate dehydrogenase, E1 component	sucA
SAUSA300_1711	**0,8**	0,299103897	No	47	37	62	53	45	35	49	66	61	proline dehydrogenase	putA
SAUSA300_1190	**0,8**	0,000924528	Yes	398	375	419	501	530	413	635	593	640	glycerol uptake operon antiterminator regulatory protei	glpP
SAUSA300_2363	**0,8**	0,023916799	No	716	567	670	463	449	393	610	498	591	cation efflux family protein	SAUSA300_2363
SAUSA300_1699	**0,8**	0,000502419	Yes	2275	2337	2330	1887	2263	1838	2563	2575	2639	pseudouridine synthase, family 1	SAUSA300_1699
SAUSA300_2184	**0,8**	0,004849209	Yes	18842	22204	22200	23531	23556	22481	30717	32200	27725	preprotein translocase, SecY subunit	SAUSA300_2184
SAUSA300_1853	**0,8**	0,112620258	No	264	379	341	243	295	250	368	337	326	conserved hypothetical protein	SAUSA300_1853
SAUSA300_2384	**0,8**	0,037013337	No	4671	4009	4070	1991	2495	1911	2730	2770	2846	putative Na+/H+ antiporter	SAUSA300_2384
SAUSA300_0880	**0,8**	0,673544034	No	5	3	21	0	11	1	5	5	8	conserved hypothetical protein	SAUSA300_0880
SAUSA300_0267	**0,8**	0,140597093	No	103	99	99	76	86	47	82	100	87	transposase	SAUSA300_0267
SAUSA300_1149	**0,8**	0,002175043	Yes	23932	21036	19897	20725	20815	19873	27490	28090	24694	30S ribosomal protein S2	rpsB

SAUSA300_2150	**0,8**	0,156677432	No	65	79	67	96	56	83	118	85	111	PTS system, lactose-specific IIBC component	lacE
SAUSA300_0917	**0,8**	0,033483611	No	2919	2100	2284	2667	2626	1998	3192	3161	3187	putative membrane protein	SAUSA300_0917
SAUSA300_0369	**0,8**	0,262943326	No	47	67	64	33	43	31	37	47	54	conserved hypothetical protein	SAUSA300_0369
SAUSA300_1637	**0,8**	0,193313768	No	91	79	91	53	59	46	60	66	79	putative membrane protein	SAUSA300_1637
SAUSA300_1912	**0,8**	0,447611912	No	26	31	39	13	36	24	37	25	33	putative membrane protein	SAUSA300_1912
SAUSA300_0862	**0,8**	0,139554276	No	155	119	106	47	59	53	65	77	68	glycerophosphoryl diester phosphodiesterase	glpQ
SAUSA300_2343	**0,8**	0,582536901	No	59	37	209	47	54	61	30	145	49	respiratory nitrate reductase, alpha subunit	SAUSA300_2343
SAUSA300_0398	**0,8**	0,483793242	No	9	7	8	6	14	7	12	12	11	exotoxin	SAUSA300_0398
SAUSA300_2352	**0,8**	0,088021523	No	861	768	646	682	591	620	887	836	772	addiction module toxin, Txe/YoeB family	SAUSA300_2352
SAUSA300_1468	**0,8**	0,002340573	Yes	3088	3363	3294	3131	3210	2956	3790	4035	4382	DNA repair protein RecN	recN
SAUSA300_2056	**0,8**	NA	NA	9	12	34	11	11	9	11	6	30	conserved hypothetical protein	SAUSA300_2056
SAUSA300_1564	**0,8**	0,122657327	No	26	40	40	44	41	44	54	61	59	acetyl-CoA carboxylase, biotin carboxyl carrier protein	accB
SAUSA300_2195	**0,8**	0,087425692	No	2838	3203	4115	2933	3097	2932	3918	3876	4015	30S ribosomal protein S17	rpsQ
SAUSA300_0545	**0,8**	0,016066397	No	1547	1482	1229	874	789	597	1042	932	999	Flavodoxin-like fold	SAUSA300_0545
SAUSA300_1311	**0,8**	0,003622036	Yes	1219	1396	1437	825	989	788	1130	1117	1162	undecaprenyldiphospho-muramoylpentapeptide beta-N	murG
SAUSA300_1072	**0,8**	0,000270099	Yes	7933	6583	7130	5436	5150	4884	7034	7141	6177	protein mraZ	mraZ
SAUSA300_2241	**0,8**	0,045620933	No	191	218	219	2906	2466	2205	3201	3357	3433	urease accessory protein UreE	ureE
SAUSA300_0916	**0,8**	0,001485414	Yes	1893	1725	1506	1309	1228	1254	1640	1771	1588	conserved hypothetical protein	SAUSA300_0916
SAUSA300_2560	**0,8**	0,020845926	No	288	271	266	508	458	345	574	639	517	conserved hypothetical protein	SAUSA300_2560
SAUSA300_0705	**0,8**	0,002249389	Yes	2739	2742	3001	2664	3016	2397	3420	3530	3685	ATP-dependent DNA helicase RecQ	recQ
SAUSA300_0220	**0,8**	0,605101044	No	167	178	334	182	169	191	116	540	114	formate acetyltransferase	pflB
SAUSA300_0965	**0,8**	0,012331277	No	2096	2944	2057	3878	3250	3552	4908	4959	4273	methylenetetrahydrofolate dehydrogenase/methenylte	folD
SAUSA300_1176	**0,8**	0,00367264	Yes	1397	1270	1399	995	1149	844	1311	1305	1323	CDP-diacylglycerol--glycerol-3-phosphate 3-phosphatid	pgsA
SAUSA300_0395	**0,8**	0,296046469	No	67	48	68	92	38	75	96	104	82	exotoxin	SAUSA300_0395
SAUSA300_2333	**0,8**	0,338262994	No	157	105	176	70	108	90	107	151	100	nitrite extrusion protein	narK
SAUSA300_0191	**0,8**	0,024515275	No	2625	3530	3328	3486	2840	3933	4392	4819	4432	PTS system, glucose-specific IIBC component domain pr	ptsG
SAUSA300_2370	**0,8**	0,308813249	No	40	25	49	24	32	37	31	45	48	putative 8-amino-7-oxononanoate synthase	SAUSA300_2370
SAUSA300_2602	**0,8**	0,516175971	No	7	14	14	11	18	12	14	16	24	intercellular adhesion protein C	icaC
SAUSA300_1708	**0,8**	0,08851933	No	3989	3069	3660	1743	1528	1168	2155	2190	1587	staphylococcal accessory regulator Rot	rot
SAUSA300_2099	**0,8**	0,02973704	No	2456	2275	1631	1580	1244	1241	1931	1511	1983	cation efflux family protein	SAUSA300_2099
SAUSA300_2638	**0,8**	0,36414865	No	26	22	28	26	20	10	24	30	22	conserved hypothetical protein	SAUSA300_2638
SAUSA300_1097	**0,8**	0,118301722	No	67	127	125	337	316	427	498	421	534	orotidine 5'-phosphate decarboxylase	pyrF
SAUSA300_1739	**0,8**	0,009074796	Yes	1963	1838	1961	941	951	1003	1103	1517	1252	conserved hypothetical protein	SAUSA300_1739
SAUSA300_1166	**0,7**	0,00147196	Yes	16962	13416	14096	12156	14053	12490	17486	17998	16235	30S ribosomal protein S15	rpsO
SAUSA300_0630	**0,7**	0,013423826	No	5512	6091	6166	3731	3471	3576	4361	4124	5940	ABC transporter, ATP-binding protein	SAUSA300_0630
SAUSA300_0665	**0,7**	0,020032322	No	594	552	378	358	298	290	436	424	413	acetyltransferase, GNAT family	SAUSA300_0665
SAUSA300_0731	**0,7**	0,011319765	No	1231	887	1142	1083	1159	957	1406	1411	1469	glycosyl transferase, group 4 family protein	SAUSA300_0731
SAUSA300_0473	**0,7**	2,13E-07	Yes	3047	2964	3296	1806	1803	1634	2233	2460	2330	pur operon repressor	purR
SAUSA300_0314	**0,7**	0,331357963	No	40	36	53	16	32	22	27	34	31	sodium:solute symporter family protein	SAUSA300_0314
SAUSA300_2192	**0,7**	0,022977321	No	6913	8360	9151	8522	8770	8626	11512	12525	10861	50S ribosomal protein L5	rplE
SAUSA300_1186	**0,7**	0,007801154	Yes	1827	1957	1575	1220	1186	1053	1537	1476	1641	conserved hypothetical protein	SAUSA300_1186
SAUSA300_1074	**0,7**	0,000792614	Yes	5725	5564	5317	4863	4605	4719	6028	7094	5990	cell division protein	ftsL
SAUSA300_0998	**0,7**	0,130041149	No	270	326	382	195	180	211	249	226	322	conserved hypothetical protein	SAUSA300_0998
SAUSA300_1456	**0,7**	0,136275225	No	186	205	163	328	302	319	317	656	317	alpha glucosidase	SAUSA300_1456
SAUSA300_0360	**0,7**	0,317096841	No	121	208	86	34	41	41	50	61	48	Cys/Met metabolism PLP-dependent enzyme	SAUSA300_0360
SAUSA300_1414	**0,7**	0,653881845	No	0	0	0	0	0	3	5	1	0	phiSLT ORF 78B-like protein	SAUSA300_1414
SAUSA300_1790	**0,7**	0,019947375	No	3264	2977	3356	2520	2455	2047	3188	3100	3225	foldase protein PrsA precursor	prsA
SAUSA300_0640	**0,7**	0,01541032	No	3932	3237	3974	4133	4406	3962	5490	5815	5648	putative membrane protein	SAUSA300_0640
SAUSA300_0700	**0,7**	0,008211121	Yes	310	371	295	231	232	179	294	272	300	conserved hypothetical protein	SAUSA300_0700
SAUSA300_0050	**0,7**	0,598486731	No	10	5	32	4	27	6	14	13	26	conserved hypothetical protein	SAUSA300_0050
SAUSA300_1353	**0,7**	1,40E-05	Yes	699	693	716	444	523	435	627	602	655	conserved hypothetical protein	SAUSA300_1353
SAUSA300_0660	**0,7**	0,165986741	No	227	151	316	231	329	207	335	300	409	conserved hypothetical protein	SAUSA300_0660
SAUSA300_1718	**0,7**	0,001899709	Yes	276	300	243	208	228	185	297	264	276	aresenical pump membrane protein	arsB
SAUSA300_2582	**0,7**	0,000581234	Yes	1438	1155	1493	1376	1535	1313	1852	1812	2064	conserved hypothetical protein	SAUSA300_2582
SAUSA300_2167	**0,7**	0,038304275	No	441	285	329	158	203	182	249	214	272	conserved hypothetical protein	SAUSA300_2167
SAUSA300_2305	**0,7**	0,63251591	No	12	4	11	3	2	4	7	2	7	transposase, truncation	SAUSA300_2305
SAUSA300_0734	**0,7**	0,057340152	No	72	75	60	59	47	72	89	77	83	putative comf operon protein 1	SAUSA300_0734
SAUSA300_1666	**0,7**	0,000437074	Yes	24616	21400	21302	19587	20094	18356	26871	27915	24164	30S ribosomal protein S4	rpsD
SAUSA300_0157	**0,7**	0,092595887	No	45	25	33	64	52	83	97	84	96	capsular polysaccharide biosynthesis protein Cap5F	cap5F

SAUSA300_2247	**0,7**	0,152669424	No	215	190	177	44	72	30	68	61	64	staphylococcal accessory regulator	SAUSA300_2247
SAUSA300_1664	**0,7**	8,85E-05	Yes	11393	9720	10925	5704	5506	4977	7044	7612	7389	septation ring formation regulator EzrA	ezrA
SAUSA300_1040	**0,7**	0,000783613	Yes	496	433	504	289	259	241	354	336	388	conserved hypothetical protein	SAUSA300_1040
SAUSA300_1318	**0,7**	0,000309854	Yes	1889	1937	1686	881	875	723	1145	1112	1119	DegV family protein	SAUSA300_1318
SAUSA300_1967	**0,7**	0,525042197	No	12	14	11	6	9	6	11	9	9	conserved hypothetical phage protein	SAUSA300_1967
SAUSA300_0133	**0,7**	0,160299613	No	62	77	112	62	79	50	84	79	95	putative membrane protein	SAUSA300_0133
SAUSA300_0832	**0,7**	0,014246648	No	536	526	632	483	433	350	558	562	616	conserved hypothetical protein	SAUSA300_0832
SAUSA300_0284	**0,7**	0,069415081	No	122	82	83	89	101	99	143	105	148	conserved hypothetical protein	SAUSA300_0284
SAUSA300_0474	**0,7**	0,001954253	Yes	6953	5722	5870	1983	1704	1987	2766	2678	2347	putative endoribonuclease L-PSP	SAUSA300_0474
SAUSA300_0178	**0,7**	0,030244101	No	2954	2940	2484	2482	1999	2242	3293	2754	3204	conserved hypothetical protein	SAUSA300_0178
SAUSA300_1689	**0,7**	0,00955251	Yes	1085	842	911	809	746	605	1029	928	1011	conserved hypothetical protein	SAUSA300_1689
SAUSA300_2618	**0,7**	0,174984215	No	71	97	100	30	27	31	32	43	48	conserved hypothetical protein	SAUSA300_2618
SAUSA300_2465	**0,7**	0,587901226	No	2	4	6	9	11	1	12	4	15	ABC transporter, ATP-binding protein	SAUSA300_2465
SAUSA300_0128	**0,7**	0,001295512	Yes	307	227	243	296	237	282	389	394	349	conserved hypothetical protein	SAUSA300_0128
SAUSA300_2094	**0,7**	0,000214192	Yes	3359	3528	2938	2026	1666	1706	2466	2340	2619	conserved hypothetical protein	SAUSA300_2094
SAUSA300_0169	**0,7**	0,250178149	No	72	56	85	50	43	27	49	45	72	conserved hypothetical protein	SAUSA300_0169
SAUSA300_2615	**0,7**	0,095929803	No	119	130	106	53	47	34	62	58	65	conserved hypothetical protein	SAUSA300_2615
SAUSA300_0977	**0,7**	0,016920478	No	338	298	336	110	135	95	161	157	144	cobalt transport family protein	SAUSA300_0977
SAUSA300_1241	**0,7**	0,075607205	No	420	292	405	371	295	269	491	378	431	conserved hypothetical protein	SAUSA300_1241
SAUSA300_0208	**0,7**	0,234420851	No	34	68	49	37	25	40	47	55	44	putative maltose ABC transporter, ATP-binding protein	SAUSA300_0208
SAUSA300_0838	**0,7**	6,07E-06	Yes	13073	11229	11710	8558	8865	7113	11361	11181	11314	D-alanine-activating enzyme/D-alanine-D-alanyl, dltD pr	dltD
SAUSA300_1738	**0,7**	0,14975189	No	62	51	56	43	52	27	57	58	52	putative lipoprotein	SAUSA300_1738
SAUSA300_2014	**0,7**	0,021370279	No	298	286	316	83	77	72	101	104	117	threonine dehydratase	ilvA
SAUSA300_0503	**0,7**	0,099060503	No	109	146	93	103	65	78	129	91	128	transcriptional regulator, gntR family protein	SAUSA300_0503
SAUSA300_2426	**0,7**	0,028883922	No	95	110	128	82	88	74	113	105	117	conserved hypothetical protein	SAUSA300_2426
SAUSA300_0206	**0,7**	0,098575109	No	973	724	687	239	176	174	269	255	302	flavodoxin family protein	SAUSA300_0206
SAUSA300_1598	**0,7**	0,001523015	Yes	729	876	992	682	764	568	865	954	961	holliday junction DNA helicase RuvA	ruvA
SAUSA300_0488	**0,7**	0,003325617	Yes	913	935	1139	869	987	726	1224	1117	1229	hypoxanthine phosphoribosyltransferase	hpt
SAUSA300_0171	**0,7**	0,020614042	No	207	151	218	140	167	120	185	200	202	cation efflux family protein	SAUSA300_0171
SAUSA300_1625	**0,7**	0,00072467	Yes	12380	10299	10592	9198	9225	8976	13122	13482	11384	50S ribosomal protein L20	rplT
SAUSA300_0525	**0,7**	0,001761588	Yes	11226	10247	11896	7341	7118	7709	10249	11135	9451	ribosomal protein L7/L12	rplL
SAUSA300_2052	**0,7**	0,012910338	No	251	224	187	155	178	154	223	248	206	single-stranded DNA- binding protein family	SAUSA300_2052
SAUSA300_1235	**0,7**	0,078343232	No	355	631	292	311	273	350	488	371	453	guanosine monophosphate reductase	guaC
SAUSA300_2451	**0,7**	0,014264486	No	109	125	120	87	95	74	113	117	124	drug transporter	SAUSA300_2451
SAUSA300_1288	**0,7**	0,026720711	No	575	788	622	155	187	161	216	234	250	dihydrodipicolinate synthase	dapA
SAUSA300_0657	**0,7**	0,00047182	Yes	701	654	812	669	717	608	937	896	943	conserved hypothetical protein	SAUSA300_0657
SAUSA300_0887	**0,7**	0,116940504	No	219	283	483	84	79	87	103	125	127	oligopeptide ABC transporter, permease protein	oppB
SAUSA300_1249	**0,7**	0,007431221	Yes	2401	1717	2034	898	714	689	1150	1102	972	conserved hypothetical protein	SAUSA300_1249
SAUSA300_1996	**0,7**	0,325266796	No	17	14	12	19	7	12	17	16	23	ammonium transporter	amt
SAUSA300_1504	**0,7**	0,195563217	No	24	36	35	34	38	21	41	38	50	putative competence protein ComGA	SAUSA300_1504
SAUSA300_1269	**0,7**	0,000582929	Yes	5451	4813	5120	4422	4988	3545	6274	5788	6069	methicillin resistance protein FemA	femA
SAUSA300_1455	**0,7**	0,006569074	Yes	3154	2973	2510	1817	1670	1329	2299	2114	2354	transcriptional regulator, AraC family	SAUSA300_1455
SAUSA300_2299	**0,7**	0,042253687	No	1188	790	975	925	942	770	1352	1275	1089	multidrug resistance protein A, drug resistance transpor	SAUSA300_2299
SAUSA300_1277	**0,7**	0,037973242	No	217	160	201	163	187	192	256	238	269	conserved hypothetical protein	SAUSA300_1277
SAUSA300_1333	**0,7**	0,000261862	Yes	4178	3945	3935	2536	2790	2100	3317	3452	3655	conserved hypothetical protein	SAUSA300_1333
SAUSA300_0201	**0,7**	0,101044522	No	145	207	159	44	38	52	70	50	74	peptide ABC transporter, permease protein	SAUSA300_0201
SAUSA300_1855	**0,7**	0,000395491	Yes	1357	1101	1045	497	444	376	632	612	609	monofunctional glycosyltransferase	sgtB
SAUSA300_0192	**0,7**	0,235423521	No	22	38	29	20	25	25	27	29	45	conserved hypothetical protein	SAUSA300_0192
SAUSA300_1851	**0,7**	0,027795897	No	320	281	295	241	268	250	368	329	374	putative membrane protein	SAUSA300_1851
SAUSA300_1908	**0,7**	4,92E-06	Yes	582	560	656	463	543	410	664	636	677	conserved hypothetical protein	SAUSA300_1908
SAUSA300_1698	**0,7**	0,002580967	Yes	2213	1764	2135	1050	980	986	1473	1349	1442	conserved hypothetical protein	SAUSA300_1698
SAUSA300_1529	**0,7**	0,065090777	No	138	133	177	117	131	84	138	149	181	diacylglycerol kinase	dgkA
SAUSA300_0448	**0,7**	0,177578023	No	742	698	870	760	818	872	1006	1611	900	PTS system, trehalose-specific IIBC component	treP
SAUSA300_0954	**0,7**	0,018295756	No	1171	1061	924	450	311	290	473	525	500	transcriptional regulator, MarR family	SAUSA300_0954
SAUSA300_1275	**0,7**	0,026812375	No	81	64	76	102	83	111	142	120	163	peptide ABC transporter, permease protein	SAUSA300_1275
SAUSA300_2302	**0,7**	7,19E-05	Yes	2513	2031	1883	1373	1260	1344	2011	1763	1872	teicoplanin resistance associated membrane protein Tc	tcaA
SAUSA300_1047	**0,7**	0,018760282	No	1510	1904	2003	1159	1073	1180	1426	2089	1350	succinate dehydrogenase, flavoprotein subunit	sdhA
SAUSA300_1986	**0,7**	0,095334787	No	665	441	467	160	196	132	247	203	248	nitroreductase family protein	SAUSA300_1986
SAUSA300_0693	**0,7**	0,071839181	No	5246	3710	2332	3026	2757	2237	3735	3426	4345	putative lipoprotein	SAUSA300_0693

SAUSA300_1076	**0,7**	2,22E-07	Yes	1257	1278	1444	902	929	800	1177	1304	1253	phospho-N-acetylmuramoyl-pentapeptide-transferase	mraY
SAUSA300_0222	**0,7**	5,46E-05	Yes	1616	1576	1526	726	724	562	997	940	922	putative membrane protein	SAUSA300_0222
SAUSA300_2153	**0,7**	0,179214598	No	12	15	12	20	25	28	33	35	37	tagatose-6-phosphate kinase	lacC
SAUSA300_1522	**0,7**	0,00019015	Yes	3817	3358	3576	3607	3410	3327	5170	4664	4933	DNA primase	dnaG
SAUSA300_1084	**0,7**	0,108436856	No	520	407	510	311	304	291	430	425	452	conserved hypothetical protein	SAUSA300_1084
SAUSA300_1974	**0,7**	0,114225362	No	446	359	382	175	133	136	188	254	201	Leukocidin/Hemolysin toxin family protein	SAUSA300_1974
SAUSA300_1686	**0,7**	2,08E-08	Yes	3961	3648	3911	3715	3509	3037	4763	4892	4989	UDP-N-acetylmuramate--alanine ligase	murC
SAUSA300_2530	**0,7**	0,000642375	Yes	165	170	168	222	196	231	315	308	313	transcriptional regulator, TetR family	SAUSA300_2530
SAUSA300_1010	**0,7**	0,020792902	No	394	308	335	265	273	238	367	421	329	conserved hypothetical protein	SAUSA300_1010
SAUSA300_2314	**0,7**	6,30E-07	Yes	1528	1545	1331	1113	1120	948	1447	1544	1562	conserved hypothetical protein	SAUSA300_2314
SAUSA300_2248	**0,7**	0,073669217	No	468	424	511	69	97	59	108	95	117	transcriptional regulator, AraC family	SAUSA300_2248
SAUSA300_1627	**0,7**	3,06E-06	Yes	11891	10236	9882	8593	8648	8416	13132	12920	10815	translation initiation factor IF-3	infC
SAUSA300_2095	**0,7**	0,003180052	Yes	560	506	491	311	302	274	418	486	376	conserved hypothetical protein	SAUSA300_2095
SAUSA300_0298	**0,7**	0,051194471	No	43	40	56	70	61	86	91	92	133	conserved hypothetical protein	SAUSA300_0298
SAUSA300_0653	**0,7**	0,049286006	No	512	386	532	485	568	430	699	633	817	transcriptional regulator, AraC family	SAUSA300_0653
SAUSA300_1444	**0,7**	0,000176542	Yes	512	419	525	344	334	315	459	478	494	segregation and condensation protein B	scpB
SAUSA300_0101	**0,7**	0,016738694	No	81	74	75	105	142	117	165	165	190	staphylococcal tandem lipoprotein	SAUSA300_0101
SAUSA300_0258	**0,7**	0,000865647	Yes	1609	1248	1324	970	1046	846	1373	1417	1340	transcriptional regulator, GntR family	SAUSA300_0258
SAUSA300_0542	**0,7**	0,295015605	No	91	66	113	29	47	30	54	39	61	deoxynucleoside kinase family protein	SAUSA300_0542
SAUSA300_0668	**0,7**	0,006447322	Yes	4538	4248	3375	2046	1636	1862	2931	2767	2358	conserved hypothetical protein	SAUSA300_0668
SAUSA300_0992	**0,7**	0,003619369	Yes	2713	2081	2546	2831	2635	2085	4134	3211	3606	putative lipoprotein	SAUSA300_0992
SAUSA300_1399	**0,7**	0,568799635	No	0	1	5	1	2	0	4	1	2	phiSLT ORF110-like protein	SAUSA300_1399
SAUSA300_1048	**0,7**	0,000335406	Yes	1712	1700	1554	888	827	954	1219	1558	1108	succinate dehydrogenase, iron-sulfur protein	sdhB
SAUSA300_0182	**0,7**	0,000521314	Yes	971	754	753	580	548	507	786	739	852	4'-phosphopantetheinyl transferase superfamily protein	SAUSA300_0182
SAUSA300_0031	**0,7**	0,008755503	Yes	262	216	224	331	347	247	464	433	447	conserved hypothetical protein	SAUSA300_0031
SAUSA300_2258	**0,7**	0,000339039	Yes	9934	11175	10767	5962	5409	6702	8867	9162	8287	formate dehydrogenase, alpha subunit	SAUSA300_2258
SAUSA300_2168	**0,7**	0,008740026	Yes	348	316	358	155	207	148	224	214	298	conserved hypothetical protein	SAUSA300_2168
SAUSA300_1358	**0,7**	1,21E-06	Yes	1042	1040	1040	766	782	716	1084	1093	1112	nucleoside diphosphate kinase superfamily	ndk
SAUSA300_0663	**0,7**	0,009838899	Yes	1411	899	1101	1050	1055	849	1534	1437	1340	putative lipoprotein	SAUSA300_0663
SAUSA300_0053	**0,7**	0,337942298	No	107	62	152	54	77	58	103	71	110	Spermidine N(1)-acetyltransferase	speG
SAUSA300_0658	**0,7**	1,28E-10	Yes	1693	1688	1821	1471	1467	1258	1967	2046	2085	transcriptional regulator, LysR family	SAUSA300_0658
SAUSA300_1795	**0,7**	0,007454846	Yes	15673	11004	11014	9900	9324	7756	14552	12578	12345	conserved hypothetical protein	SAUSA300_1795
SAUSA300_0162	**0,7**	0,305346755	No	22	22	21	16	16	9	24	15	21	capsular polysaccharide biosynthesis protein Cap5K	cap5K
SAUSA300_1238	**0,7**	0,001025718	Yes	813	762	711	468	512	367	688	611	662	conserved hypothetical protein	SAUSA300_1238
SAUSA300_0520	**0,7**	6,90E-06	Yes	1190	1235	1265	1090	1001	938	1508	1434	1483	preprotein translocase, SecE subunit	SAUSA300_0520
SAUSA300_1212	**0,7**	0,376345151	No	26	29	23	6	5	9	11	7	13	conserved hypothetical protein	SAUSA300_1212
SAUSA300_0596	**0,7**	2,37E-07	Yes	5276	5021	4913	2442	2450	1912	3342	3238	3346	arginyl-tRNA synthetase	argS
SAUSA300_2368	**0,7**	0,3775698	No	5	12	13	6	9	7	11	12	10	putative membrane protein	SAUSA300_2368
SAUSA300_0588	**0,7**	0,005108933	Yes	150	141	142	70	95	87	127	110	128	conserved hypothetical protein	SAUSA300_0588
SAUSA300_0080	**0,7**	0,457826368	No	0	0	0	0	0	0	3	1	0	conserved hypothetical protein	SAUSA300_0080
SAUSA300_0029	**0,7**	0,106871328	No	126	89	110	89	115	65	130	125	140	conserved hypothetical protein	SAUSA300_0029
SAUSA300_1134	**0,7**	0,005932563	Yes	13109	11515	12060	10106	10875	11315	16382	16884	14156	50S ribosomal protein L19	rplS
SAUSA300_1218	**0,7**	0,44837792	No	17	15	18	4	7	1	8	4	8	ABC transporter, permease protein	SAUSA300_1218
SAUSA300_0290	**0,7**	0,36238238	No	7	10	16	16	20	7	15	20	30	putative lipoprotein	SAUSA300_0290
SAUSA300_2620	**0,7**	0,055310651	No	1169	835	723	404	241	263	435	387	526	conserved hypothetical protein	SAUSA300_2620
SAUSA300_0970	**0,7**	0,048154473	No	482	957	701	637	496	951	1140	782	1164	phosphoribosylformylglycinamidine synthase I	purQ
SAUSA300_0598	**0,7**	0,000161757	Yes	1936	2074	1657	680	530	547	903	857	824	putative iron compound ABC transporter, iron compour	SAUSA300_0598
SAUSA300_0110	**0,7**	0,004337827	Yes	210	223	246	120	122	115	170	167	187	transcriptional regulator, GntR family/aminotransferase	SAUSA300_0110
SAUSA300_2209	**0,7**	0,000606582	Yes	1242	1252	1064	885	863	621	1158	1142	1177	conserved hypothetical protein	SAUSA300_2209
SAUSA300_2216	**0,7**	0,007812885	Yes	114	120	106	86	77	74	123	106	121	transcriptional regulator, MarR family	SAUSA300_2216
SAUSA300_1096	**0,7**	0,033790149	No	377	642	490	1511	1377	1964	2604	2011	2571	carbamoyl-phosphate synthase, large subunit	carB
SAUSA300_0256	**0,7**	0,160945944	No	196	153	287	40	63	40	64	95	54	holin-like protein lrgA	SAUSA300_0256
SAUSA300_0136	**0,7**	0,002076025	Yes	3471	2965	2856	384	345	349	549	566	481	cell wall surface anchor family protein	SAUSA300_0136
SAUSA300_1131	**0,7**	0,004849209	Yes	4289	3727	3541	2804	3302	2819	4666	4347	4173	30S ribosomal protein S16	rpsP
SAUSA300_2023	**0,7**	0,001904561	Yes	3004	2895	2846	1285	1075	1301	1947	1834	1636	anti-sigma-B factor, serine-protein kinase	rsbW
SAUSA300_0794	**0,7**	0,018603905	No	241	201	221	84	101	77	155	115	119	Toprim domain protein	SAUSA300_0794
SAUSA300_0541	**0,7**	3,99E-06	Yes	1666	1372	1436	855	935	726	1190	1153	1352	deoxynucleoside kinase family protein	SAUSA300_0541
SAUSA300_1060	**0,7**	0,327423834	No	9	10	13	6	11	9	14	11	14	putative exotoxin 4	SAUSA300_1060
SAUSA300_0854	**0,7**	0,001437642	Yes	773	771	982	624	651	470	827	866	884	Na(+)/H(+) antiporter subunit B	mnhB

SAUSA300_1052	**0,7**	0,090336461	No	5102	2004	2501	1320	1826	1150	2441	2216	1766	fibrinogen-binding protein	SAUSA300_1052
SAUSA300_0881	**0,7**	0,448313466	No	9	5	7	10	5	4	14	5	12	putative membrane protein	SAUSA300_0881
SAUSA300_2327	**0,7**	0,010366121	No	1659	1065	1189	405	376	393	656	571	526	conserved hypothetical protein	SAUSA300_2327
SAUSA300_2596	**0,7**	0,002400407	Yes	121	122	113	102	131	136	184	174	185	capsular polysaccharide biosynthesis protein Cap1C	cap1C
SAUSA300_1846	**0,7**	3,12E-05	Yes	1281	1236	1236	894	992	736	1176	1274	1427	conserved hypothetical protein	SAUSA300_1846
SAUSA300_1975	**0,7**	0,124934575	No	348	231	345	126	101	72	121	157	175	Aerolysin/Leukocidin family protein	SAUSA300_1975
SAUSA300_2025	**0,7**	5,55E-10	Yes	2218	2342	2241	1071	994	877	1469	1406	1486	sigma-B regulation protein	rsbU
SAUSA300_1272	**0,7**	0,001630236	Yes	522	441	439	388	343	303	529	487	525	conserved hypothetical protein	SAUSA300_1272
SAUSA300_2280	**0,7**	0,171501337	No	28	25	25	14	23	12	22	24	25	metallothiol transferase fosB	fosB
SAUSA300_2566	**0,7**	0,002076025	Yes	226	181	228	160	212	161	253	248	284	transcriptional regulator, Crp/Fnr family	arcR
SAUSA300_2307	**0,7**	0,156038302	No	47	60	77	29	27	16	26	43	39	ABC transporter, permease protein	SAUSA300_2307
SAUSA300_1911	**0,7**	0,000694447	Yes	1355	1341	1488	769	735	731	1120	958	1255	ABC transporter, ATP-binding protein	SAUSA300_1911
SAUSA300_0183	**0,7**	0,363494289	No	551	546	1260	822	733	979	826	2333	857	conserved hypothetical protein	SAUSA300_0183
SAUSA300_0289	**0,7**	0,013403867	No	358	327	568	524	548	566	801	747	904	conserved hypothetical protein	SAUSA300_0289
SAUSA300_2556	**0,7**	0,000240659	Yes	513	480	446	337	293	266	452	413	472	ABC transporter protein	SAUSA300_2556
SAUSA300_0566	**0,7**	0,017652838	No	7611	7749	8339	3401	3275	4176	5358	5918	4994	amino acid permease	SAUSA300_0566
SAUSA300_2155	**0,7**	0,36927105	No	5	7	5	6	16	21	17	24	23	galactose-6-phosphate isomerase	lacA
SAUSA300_0645	**0,7**	0,000423597	Yes	570	609	510	430	381	321	597	526	571	DNA-binding response regulator	SAUSA300_0645
SAUSA300_1098	**0,7**	0,015626781	No	134	163	121	272	239	315	432	375	440	orotate phosphoribosyltransferase	pyrE
SAUSA300_0114	**0,7**	0,070045602	No	1691	1174	2010	298	358	296	388	490	561	staphylococcal accessory regulator	SAUSA300_0114
SAUSA300_2106	**0,7**	0,004816778	Yes	921	957	825	380	354	476	650	620	557	putative transcriptional regulator	SAUSA300_2106
SAUSA300_1792	**0,7**	5,04E-09	Yes	11280	10327	9754	5893	5754	5161	8334	8259	8612	conserved hypothetical protein	SAUSA300_1792
SAUSA300_0425	**0,7**	0,32613024	No	808	987	1619	1024	1032	1213	1311	2638	1215	NADH dehydrogenase I, F subunit	SAUSA300_0425
SAUSA300_0373	**0,7**	0,004117799	Yes	1030	788	859	463	500	413	720	595	754	conserved hypothetical protein	SAUSA300_0373
SAUSA300_2386	**0,7**	9,04E-05	Yes	1058	850	977	929	951	720	1251	1255	1397	beta-lactamase	SAUSA300_2386
SAUSA300_1857	**0,7**	0,0048241	Yes	150	151	149	62	61	62	101	94	86	conserved hypothetical protein	SAUSA300_1857
SAUSA300_0982	**0,7**	0,003780639	Yes	1362	1222	1233	338	394	414	672	616	446	conserved hypothetical protein	SAUSA300_0982
SAUSA300_0836	**0,7**	0,00025808	Yes	3419	2836	3539	2148	2189	1989	3198	2861	3476	dltB protein	dltB
SAUSA300_1413	**0,7**	0,420478032	No	9	8	6	4	9	0	6	6	9	conserved hypothetical phage protein	SAUSA300_1413
SAUSA300_1633	**0,7**	0,001491486	Yes	219	263	172	208	187	192	281	297	312	glyceraldehyde-3-phosphate dehydrogenase, type I	gap
SAUSA300_1298	**0,7**	0,033086066	No	406	412	646	526	487	429	678	654	863	putative XpaC protein	SAUSA300_1298
SAUSA300_1676	**0,7**	0,001198243	Yes	351	300	323	238	250	158	315	334	322	probable transglycosylase	sgtA
SAUSA300_0730	**0,7**	0,003094897	Yes	484	430	548	497	512	464	706	687	836	GGDEF domain protein	SAUSA300_0730
SAUSA300_0359	**0,7**	0,180769925	No	134	298	100	27	29	30	53	44	38	trans-sulfuration enzyme family protein	SAUSA300_0359
SAUSA300_0662	**0,7**	0,053361289	No	41	56	46	39	23	30	55	42	47	acetyltransferase, GNAT family	SAUSA300_0662
SAUSA300_0047	**0,7**	0,015626781	No	255	215	210	116	149	93	191	151	197	conserved hypothetical protein	SAUSA300_0047
SAUSA300_1798	**0,7**	6,46E-06	Yes	529	568	496	318	397	274	478	497	501	DNA-binding response regulator	SAUSA300_1798
SAUSA300_0853	**0,7**	1,65E-05	Yes	1254	1187	1238	992	1010	722	1353	1389	1375	Na(+)/H(+) antiporter subunit C	mnhC
SAUSA300_1501	**0,7**	0,515415586	No	0	4	1	1	2	3	4	3	5	putative competence protein ComG	SAUSA300_1501
SAUSA300_1984	**0,7**	0,011090727	No	496	428	491	318	385	235	479	465	478	putative membrane protein	SAUSA300_1984
SAUSA300_0971	**0,7**	0,00759567	Yes	2015	3612	3120	2069	1936	2956	3748	2945	3927	phosphoribosylformylglycinamidine synthase II	purL
SAUSA300_1044	**0,7**	0,001904561	Yes	7174	6239	6593	5270	7641	5306	10577	8949	8184	thioredoxin	trx
SAUSA300_2340	**0,7**	0,005345972	Yes	176	201	189	86	65	59	104	104	117	respiratory nitrate reductase, gamma subunit	narI
SAUSA300_1018	**0,7**	0,00018447	Yes	1765	1266	1499	1124	1100	849	1471	1481	1723	conserved hypothetical protein	SAUSA300_1018
SAUSA300_2447	**0,7**	0,01515568	No	3343	2612	3166	2336	1841	2817	4026	3562	3147	conserved hypothetical protein	SAUSA300_2447
SAUSA300_0295	**0,7**	0,290998203	No	7	10	11	6	16	10	22	8	19	conserved hypothetical protein	SAUSA300_0295
SAUSA300_0801	**0,7**	0,002624275	Yes	625	528	524	444	550	393	656	687	772	staphylococcal enterotoxin Q	seq
SAUSA300_1103	**0,7**	1,53E-11	Yes	2286	2304	2636	1203	1215	1192	1778	1846	1877	DNA-directed RNA polymerase, omega subunit	rpoZ
SAUSA300_2626	**0,7**	0,027011834	No	317	220	233	112	142	149	248	211	163	conserved hypothetical protein	SAUSA300_2626
SAUSA300_0968	**0,7**	0,072439638	No	143	396	247	233	205	365	483	288	485	phosphoribosylaminoimidazole-succinocarboxamide sy	purC
SAUSA300_0283	**0,7**	0,013676219	No	3224	1993	1551	2130	1756	1964	3479	2475	3067	essC protein	SAUSA300_0283
SAUSA300_0987	**0,7**	0,160299613	No	350	182	464	703	721	402	761	1045	1058	cytochrome D ubiquinol oxidase, subunit II	SAUSA300_0987
SAUSA300_0944	**0,7**	2,94E-11	Yes	1948	1926	1962	1233	1285	1171	1735	1831	2075	1,4-dihydroxy-2-naphthoate octaprenyltransferase	menA
SAUSA300_2479	**0,7**	0,032519108	No	301	271	222	255	210	186	317	433	262	Holin-like protein cidA	cidA
SAUSA300_0372	**0,7**	0,075966179	No	8665	10435	6068	1475	870	1612	2513	1861	1809	putative lipoprotein	SAUSA300_0372
SAUSA300_1220	**0,7**	0,000692413	Yes	332	313	288	241	183	180	322	290	325	DNA-binding response regulator, LuxR family	SAUSA300_1220
SAUSA300_1354	**0,7**	2,67E-05	Yes	2410	2275	2228	1306	1458	1037	2010	1844	1984	conserved hypothetical protein	SAUSA300_1354
SAUSA300_1211	**0,7**	0,038304275	No	570	480	419	97	72	56	123	116	115	conserved hypothetical protein	SAUSA300_1211
SAUSA300_1810	**0,7**	0,001916683	Yes	234	281	253	183	201	143	243	281	286	IS1181, transposase	SAUSA300_1810

SAUSA300_0461	**0,7**	2,06E-05	Yes	1323	1671	1561	896	816	722	1149	1226	1376	DNA polymerase III delta subunit	holB
SAUSA300_0153	**0,7**	0,057340152	No	38	18	14	52	41	52	82	67	78	capsular polysaccharide biosynthesis protein Cap5B	cap5B
SAUSA300_2207	**0,7**	0,001071006	Yes	14910	18280	10278	6870	6306	7159	11569	10021	9857	xanthine/uracil permease family protein	SAUSA300_2207
SAUSA300_1744	**0,6**	0,022167768	No	122	111	93	66	70	35	71	108	85	conserved hypothetical protein	SAUSA300_1744
SAUSA300_1854	**0,6**	0,00875666	Yes	257	268	323	242	255	222	360	328	430	regulatory protein RecX	SAUSA300_1854
SAUSA300_0561	**0,6**	0,05799513	No	48	55	90	40	41	35	57	60	64	hypothetical protein	SAUSA300_0561
SAUSA300_0081	**0,6**	0,001485414	Yes	685	550	630	667	561	497	931	793	961	conserved hypothetical protein	SAUSA300_0081
SAUSA300_1000	**0,6**	0,011799634	No	222	205	226	115	104	93	150	147	189	spermidine/putrescine ABC transporter, permease prot	potB
SAUSA300_0690	**0,6**	0,006283034	Yes	16162	9137	8088	8621	9624	7101	13140	12699	13749	sensor histidine kinase SaeS	saeS
SAUSA300_1616	**0,6**	6,27E-05	Yes	1080	1043	970	556	561	469	833	736	894	uroporphyrinogen-III synthase	hemD
SAUSA300_1680	**0,6**	0,065046185	No	38	57	55	43	50	34	48	87	64	acetoin utilization protein AcuA	acuA
SAUSA300_0422	**0,6**	0,000129521	Yes	897	769	641	1204	931	1047	1784	1500	1692	conserved hypothetical protein	SAUSA300_0422
SAUSA300_0188	**0,6**	4,13E-06	Yes	5828	5386	5075	1159	1125	980	1651	1733	1707	branched-chain amino acid transport system II carrier p	brnQ
SAUSA300_2640	**0,6**	0,003437187	Yes	78	97	91	74	83	70	102	135	118	putative transcriptional regulator	SAUSA300_2640
SAUSA300_0161	**0,6**	0,039724985	No	43	41	36	43	43	34	76	42	70	capsular polysaccharide biosynthesis protein Cap5J	cap5J
SAUSA300_1888	**0,6**	4,19E-05	Yes	885	761	748	614	629	586	1046	881	937	conserved hypothetical protein	SAUSA300_1888
SAUSA300_0439	**0,6**	0,005420826	Yes	413	389	420	405	435	374	769	639	502	conserved hypothetical protein	SAUSA300_0439
SAUSA300_0967	**0,6**	0,057490339	No	577	1762	800	997	742	1282	1519	1400	1893	phosphoribosylaminoimidazole carboxylase, ATPase sul	purK
SAUSA300_2323	**0,6**	0,009156311	Yes	954	673	999	872	1017	821	1367	1238	1671	transporter, CorA family	cobI
SAUSA300_0878	**0,6**	0,22956124	No	26	31	35	10	7	9	12	14	19	transcriptional regulator, LysR family	SAUSA300_0878
SAUSA300_1091	**0,6**	0,018982629	No	1373	1533	1037	1362	1064	1239	2079	1629	2109	PyrR bifunctional protein	pyrR
SAUSA300_1550	**0,6**	0,000775519	Yes	1202	949	1503	617	742	553	968	976	1063	conserved hypothetical protein	SAUSA300_1550
SAUSA300_2131	**0,6**	0,005209307	Yes	422	274	442	338	270	234	430	465	442	conserved hypothetical protein	SAUSA300_2131
SAUSA300_2565	**0,6**	0,005648477	Yes	28277	21907	31159	15816	19769	16152	24249	31848	26050	clumping factor B	clfB
SAUSA300_0199	**0,6**	0,001159275	Yes	210	242	303	125	142	160	193	234	251	conserved hypothetical protein	SAUSA300_0199
SAUSA300_1756	**0,6**	0,387554756	No	16	5	15	1	7	3	7	8	4	serine protease SplC	splC
SAUSA300_2218	**0,6**	0,000170385	Yes	704	523	540	388	370	288	603	536	519	staphylococcal accessory regulator	SAUSA300_2218
SAUSA300_2190	**0,6**	2,82E-05	Yes	4258	5151	5722	4508	4481	4390	7196	7379	6662	30S ribosomal protein S8	rpsH
SAUSA300_1493	**0,6**	0,114016979	No	55	38	48	37	50	43	60	76	74	conserved hypothetical protein	SAUSA300_1493
SAUSA300_2074	**0,6**	1,10E-05	Yes	11822	10387	11880	7835	10111	7623	14604	14230	11729	50S ribosomal protein L31 type B	rpmE
SAUSA300_1324	**0,6**	0,001135076	Yes	369	394	408	170	133	127	220	210	257	putative membrane protein	SAUSA300_1324
SAUSA300_0762	**0,6**	0,000912278	Yes	2153	1862	2161	1600	1968	1816	3137	2668	2761	preprotein translocase, SecG subunit	secG
SAUSA300_2152	**0,6**	0,137855574	No	16	15	12	30	18	30	47	41	40	tagatose 1,6-diphosphate aldolase	lacD
SAUSA300_1770	**0,6**	0,151497459	No	12	27	28	16	7	16	19	25	23	conserved hypothetical protein	SAUSA300_1770
SAUSA300_1862	**0,6**	0,000161716	Yes	5119	4145	4226	1461	1156	1541	2255	2235	2141	conserved hypothetical protein	SAUSA300_1862
SAUSA300_1434	**0,6**	0,011230724	No	541	675	496	288	210	204	414	326	389	phiSLT ORF104a-like protein, repressor	SAUSA300_1434
SAUSA300_0814	**0,6**	0,003887637	Yes	7742	4477	5144	3251	2869	2231	5122	4213	4022	conserved hypothetical protein	SAUSA300_0814
SAUSA300_2490	**0,6**	2,20E-05	Yes	546	494	440	275	241	225	393	386	404	regulatory protein, TetR family	SAUSA300_2490
SAUSA300_0770	**0,6**	0,142963758	No	36	44	35	16	0	16	24	21	16	conserved hypothetical protein	SAUSA300_0770
SAUSA300_1255	**0,6**	6,44E-05	Yes	3379	3195	3626	1867	1830	1486	2797	2458	3034	oxacillin resistance-related FmtC protein	fmtC
SAUSA300_0932	**0,6**	0,074065376	No	837	491	1161	806	956	661	1256	1037	1656	putative membrane protein	SAUSA300_0932
SAUSA300_2112	**0,6**	4,41E-05	Yes	2120	2181	2219	1313	1280	998	1937	1810	2007	conserved hypothetical protein	SAUSA300_2112
SAUSA300_2324	**0,6**	0,011904825	No	320	454	380	226	187	232	281	461	306	PTS system, sucrose-specific IIBC component	SAUSA300_2324
SAUSA300_1760	**0,6**	0,162858369	No	16	14	33	9	20	12	22	21	24	lantibiotic epidermin immunity protein F	epiG
SAUSA300_1352	**0,6**	0,022130364	No	338	246	350	152	223	127	256	254	296	putative membrane protein	SAUSA300_1352
SAUSA300_0621	**0,6**	0,000266204	Yes	1977	1251	1568	1179	1021	977	1653	1704	1760	iron-dependent repressor	SAUSA300_0621
SAUSA300_0278	**0,6**	0,026826056	No	23574	8865	7963	8053	7891	8143	13395	12595	13241	conserved hypothetical protein	SAUSA300_0278
SAUSA300_0308	**0,6**	3,50E-06	Yes	1318	1305	1281	710	588	642	1075	1147	910	ABC transporter, permease protein	SAUSA300_0308
SAUSA300_1577	**0,6**	9,13E-06	Yes	1145	999	1017	624	633	455	996	834	919	TPR domain protein	SAUSA300_1577
SAUSA300_2629	**0,6**	0,011712152	No	312	318	218	96	108	112	180	195	141	conserved hypothetical protein	SAUSA300_2629
SAUSA300_0512	**0,6**	0,000174762	Yes	797	765	953	654	739	583	1037	930	1217	PIN domain protein	SAUSA300_0512
SAUSA300_2559	**0,6**	0,006557991	Yes	670	502	699	599	620	479	906	860	988	DNA-binding response regulator	SAUSA300_2559
SAUSA300_2300	**0,6**	0,010467288	No	396	337	492	314	545	244	592	584	605	transcriptional regulator, TetR family	SAUSA300_2300
SAUSA300_0798	**0,6**	0,000113469	Yes	1845	2382	1784	278	270	322	511	456	443	ABC transporter, substrate-binding protein	SAUSA300_0798
SAUSA300_2438	**0,6**	0,408969727	No	5	10	7	1	0	3	4	3	4	staphylococcal accessory regulator U	sarU
SAUSA300_1440	**0,6**	0,001048661	Yes	62	68	62	113	108	129	211	169	191	conserved hypothetical protein	SAUSA300_1440
SAUSA300_0100	**0,6**	0,011703447	No	29	27	30	49	50	47	76	84	78	staphylococcal tandem lipoprotein	SAUSA300_0100
SAUSA300_0581	**0,6**	0,005511052	Yes	148	140	118	62	68	75	127	89	119	conserved hypothetical protein	SAUSA300_0581
SAUSA300_1784	**0,6**	6,22E-07	Yes	3446	3141	3222	2012	1848	1434	3014	2816	2773	signal transduction protein TRAP	SAUSA300_1784

SAUSA300_2309	**0,6**	6,36E-11	Yes	782	680	684	527	566	433	831	776	855	sensor histidine kinase	SAUSA300_2309
SAUSA300_0130	**0,6**	0,024291594	No	45	51	71	36	41	22	47	51	61	NAD-dependent epimerase/dehydratase family protein	SAUSA300_0130
SAUSA300_1053	**0,6**	0,064328566	No	52	27	32	29	34	24	52	36	54	conserved hypothetical protein	SAUSA300_1053
SAUSA300_1308	**0,6**	0,000343636	Yes	1424	1577	1285	404	356	359	625	621	594	DNA-binding response regulator	arlR
SAUSA300_1184	**0,6**	0,021894929	No	133	122	113	34	63	30	74	53	78	conserved hypothetical protein	SAUSA300_1184
SAUSA300_1171	**0,6**	0,000472143	Yes	1888	1576	1863	1060	1071	716	1558	1344	1764	conserved hypothetical protein	SAUSA300_1171
SAUSA300_2353	**0,6**	0,000117235	Yes	627	673	525	603	545	526	940	929	883	conserved hypothetical protein	SAUSA300_2353
SAUSA300_1771	**0,6**	0,132944162	No	127	73	177	62	106	58	109	108	163	conserved hypothetical protein	SAUSA300_1771
SAUSA300_0263	**0,6**	0,001838028	Yes	124	112	102	110	90	84	114	198	162	ribose permease	rbsD
SAUSA300_1995	**0,6**	0,000256273	Yes	183	186	180	206	144	136	241	305	263	sucrose operon repressor	scrR
SAUSA300_1117	**0,6**	0,002819865	Yes	28741	30105	36894	9162	15442	11293	26864	17858	14709	50S ribosomal protein L28	rpmB
SAUSA300_0909	**0,6**	5,22E-08	Yes	1288	1306	1080	871	818	717	1376	1242	1336	pseudouridine synthases, RluA subfamily	SAUSA300_0909
SAUSA300_0377	**0,6**	5,38E-07	Yes	1529	1158	1379	1086	1086	796	1486	1710	1688	putative lipoprotein	SAUSA300_0377
SAUSA300_2322	**0,6**	3,60E-05	Yes	863	750	704	709	712	531	1130	1025	1065	transcriptional regulator, TetR family	SAUSA300_2322
SAUSA300_0724	**0,6**	0,000551485	Yes	591	480	597	407	451	299	648	567	694	putative lipoprotein	SAUSA300_0724
SAUSA300_0746	**0,6**	0,006201793	Yes	625	639	910	541	613	460	862	803	1023	TPR domain protein	SAUSA300_0746
SAUSA300_0273	**0,6**	1,64E-06	Yes	665	513	481	241	261	228	403	408	392	putative membrane protein	SAUSA300_0273
SAUSA300_1236	**0,6**	0,000192414	Yes	753	864	782	597	721	609	1091	896	1214	conserved hypothetical protein	SAUSA300_1236
SAUSA300_0059	**0,6**	0,342788288	No	7	10	5	3	7	0	4	6	6	conserved hypothetical protein	SAUSA300_0059
SAUSA300_0231	**0,6**	0,004100125	Yes	486	316	454	821	787	566	1120	1155	1380	ABC transporter, substrate-binding protein	SAUSA300_0231
SAUSA300_2329	**0,6**	4,49E-12	Yes	6461	5113	6460	3897	3762	3369	6124	6154	6087	proton/sodium-glutamate symport protein	gltT
SAUSA300_0940	**0,6**	0,000358666	Yes	2756	2493	2900	1140	1285	942	2074	1949	1624	conserved hypothetical protein	SAUSA300_0940
SAUSA300_2042	**0,6**	5,97E-05	Yes	189	196	160	146	113	136	249	220	202	conserved hypothetical protein	SAUSA300_2042
SAUSA300_1871	**0,6**	0,374056825	No	7	4	36	9	11	6	13	6	32	conserved hypothetical protein	SAUSA300_1871
SAUSA300_1271	**0,6**	5,82E-06	Yes	906	861	821	454	485	316	663	711	723	hydrolase-related protein	SAUSA300_1271
SAUSA300_0740	**0,6**	0,002426391	Yes	493	480	576	255	286	166	371	395	423	conserved hypothetical protein	SAUSA300_0740
SAUSA300_1377	**0,6**	0,284654895	No	12	11	11	3	7	3	8	6	9	conserved hypothetical protein	SAUSA300_1377
SAUSA300_0972	**0,6**	5,44E-06	Yes	3121	4777	4038	2393	2495	3182	4603	4154	4846	amidophosphoribosyltransferase	purF
SAUSA300_2108	**0,6**	4,40E-07	Yes	1316	1161	1167	411	367	441	718	656	689	Mannitol-1-phosphate 5-dehydrogenase	mtlD
SAUSA300_1008	**0,6**	0,190359503	No	29	49	90	23	27	37	54	38	65	conserved hypothetical protein	SAUSA300_1008
SAUSA300_0703	**0,6**	5,07E-07	Yes	20985	16234	18341	17751	18299	14057	27334	27472	29664	sulfatase family protein	SAUSA300_0703
SAUSA300_1344	**0,6**	8,27E-06	Yes	625	598	610	470	399	382	797	636	683	putative DNA replication protein DnaD	SAUSA300_1344
SAUSA300_2460	**0,6**	0,004952209	Yes	279	204	267	126	153	98	238	213	188	acetyltransferase family protein	SAUSA300_2460
SAUSA300_1012	**0,6**	0,003873705	Yes	226	285	453	202	196	167	263	302	398	conserved hypothetical protein	SAUSA300_1012
SAUSA300_1094	**0,6**	0,018288092	No	79	156	113	441	329	583	852	567	911	dihydroorotase	pyrC
SAUSA300_2437	**0,6**	0,367788622	No	5	5	21	11	2	13	11	12	35	staphylococcal accessory regulator T	sarT
SAUSA300_0335	**0,6**	1,21E-06	Yes	215	194	218	178	189	135	266	282	291	MATE efflux family protein	SAUSA300_0335
SAUSA300_1200	**0,6**	0,005755145	Yes	2074	2129	1681	1426	1521	1267	2763	2079	2362	glutamine synthetase repressor	glnR
SAUSA300_0976	**0,6**	7,99E-13	Yes	8627	11283	8925	5778	5883	6677	10342	10485	10145	phosphoribosylamine--glycine ligase	purD
SAUSA300_0365	**0,6**	0,045166027	No	110	48	53	82	115	43	115	183	113	conserved hypothetical protein	SAUSA300_0365
SAUSA300_1742	**0,6**	0,002462686	Yes	143	118	117	60	72	55	91	122	104	conserved hypothetical protein	SAUSA300_1742
SAUSA300_2145	**0,6**	0,002548035	Yes	5133	4965	4819	1534	1355	1990	2989	2960	2440	glycine betaine transporter	SAUSA300_2145
SAUSA300_1793	**0,6**	2,77E-07	Yes	3007	2814	2795	1340	1328	1013	1940	2176	2156	conserved hypothetical protein	SAUSA300_1793
SAUSA300_0272	**0,6**	0,000694447	Yes	739	427	470	256	241	177	375	384	398	conserved hypothetical protein	SAUSA300_0272
SAUSA300_2303	**0,6**	0,000322636	Yes	1021	627	810	289	374	250	497	478	584	transcriptional regulator TcaR	tcaR
SAUSA300_1516	**0,6**	0,00653964	Yes	277	186	316	142	185	111	257	236	257	ABC transporter, ATP-binding protein	SAUSA300_1516
SAUSA300_1515	**0,6**	0,00086889	Yes	358	320	352	236	252	132	327	360	373	ABC transporter, permease protein	SAUSA300_1515
SAUSA300_0421	**0,6**	0,000308428	Yes	532	550	461	715	584	481	1129	915	1021	conserved hypothetical protein	SAUSA300_0421
SAUSA300_0586	**0,6**	0,020442434	No	100	62	77	30	29	25	54	45	49	conserved hypothetical protein	SAUSA300_0586
SAUSA300_0548	**0,6**	0,000456424	Yes	4521	4169	5683	4216	4693	4031	6138	8220	8064	sdrE protein	sdrE
SAUSA300_2237	**0,6**	0,003131472	Yes	102	74	96	791	760	562	1133	1154	1381	putative urea transporter	SAUSA300_2237
SAUSA300_1225	**0,6**	0,002057995	Yes	386	319	308	63	70	84	113	128	137	aspartate kinase	SAUSA300_1225
SAUSA300_0736	**0,6**	2,43E-05	Yes	3223	3204	2532	1408	1032	1125	1888	2299	2007	ribosomal subunit interface protein	yfiA
SAUSA300_0652	**0,6**	0,001619392	Yes	648	520	617	464	458	356	734	677	813	putative membrane protein	SAUSA300_0652
SAUSA300_0045	**0,6**	0,115190973	No	41	19	28	13	18	12	26	21	27	HNH endonuclease family protein	SAUSA300_0045
SAUSA300_1910	**0,6**	0,028533505	No	165	112	240	86	88	68	131	124	174	putative membrane protein	SAUSA300_1910
SAUSA300_0111	**0,6**	9,51E-05	Yes	305	242	246	125	124	84	196	177	204	conserved hypothetical protein	SAUSA300_0111
SAUSA300_1233	**0,6**	1,64E-06	Yes	2780	1934	2358	1912	1618	1603	3356	2644	2953	50S ribosomal protein L33	rpmG
SAUSA300_2311	**0,6**	0,000912278	Yes	107	100	118	57	65	40	82	94	104	conserved hypothetical protein	SAUSA300_2311

SAUSA300_0672	**0,6**	1,19E-07	Yes	22874	14852	17997	2591	2328	2110	4110	4143	4026	transcriptional regulator, MarR family	SAUSA300_0672
SAUSA300_2588	**0,6**	1,22E-05	Yes	257	233	268	401	361	260	537	581	673	preprotein translocase, SecY protein	SAUSA300_2588
SAUSA300_0559	**0,6**	1,18E-05	Yes	238	188	212	103	169	96	211	209	208	putative substrate--CoA ligase	SAUSA300_0559
SAUSA300_0858	**0,6**	0,00178306	Yes	3391	2182	2756	2257	2222	2016	4048	3862	3599	conserved hypothetical protein	SAUSA300_0858
SAUSA300_2549	**0,6**	0,002555201	Yes	272	312	307	564	509	676	870	856	1382	choline/carnitine/betaine transporter, BCCT family	bccT
SAUSA300_1623	**0,6**	2,03E-06	Yes	1066	1096	1282	743	825	589	1185	1229	1372	conserved hypothetical protein	SAUSA300_1623
SAUSA300_0506	**0,6**	3,48E-08	Yes	4323	3462	4192	1416	1594	1272	2375	2689	2448	pyrimidine nucleoside transport protein	nupC
SAUSA300_1913	**0,6**	1,44E-06	Yes	1111	1070	1156	600	618	500	984	912	1123	ABC transporter, ATP-binding protein	SAUSA300_1913
SAUSA300_1051	**0,6**	7,87E-05	Yes	2389	1881	2048	1797	1828	1063	2720	2581	2978	conserved hypothetical protein	SAUSA300_1051
SAUSA300_1153	**0,6**	2,95E-06	Yes	1033	936	1416	607	627	492	946	969	1123	undecaprenyl diphosphate synthase	uppS
SAUSA300_1125	**0,6**	8,02E-05	Yes	7728	8728	9863	5085	7188	5122	12966	9804	7989	acyl carrier protein	acpP
SAUSA300_2591	**0,6**	0,012128333	No	65	60	47	32	36	25	54	59	52	conserved hypothetical protein	SAUSA300_2591
SAUSA300_0109	**0,6**	0,300115674	No	5	1	7	1	2	1	4	4	5	integral membrane domain protein	SAUSA300_0109
SAUSA300_1005	**0,6**	4,07E-06	Yes	1288	1307	1343	1568	1377	1476	2253	2308	3297	Mn2+/Fe2+ transporter, NRAMP family	SAUSA300_1005
SAUSA300_1804	**0,6**	5,49E-08	Yes	7100	5326	5859	1896	1634	1730	3246	3159	2959	conserved hypothetical protein	SAUSA300_1804
SAUSA300_1132	**0,6**	5,41E-07	Yes	391	364	315	186	185	209	377	329	333	16S rRNA processing protein RimM	rimM
SAUSA300_0088	**0,6**	0,019248298	No	40	42	36	29	23	21	53	36	42	hypothetical protein	SAUSA300_0088
SAUSA300_0851	**0,6**	0,000472143	Yes	1040	887	1356	713	841	540	1186	1203	1382	Na(+)/H(+) antiporter subunit E	mnhE
SAUSA300_2450	**0,6**	0,000220028	Yes	794	532	760	729	816	525	1224	1314	1188	DedA family protein	SAUSA300_2450
SAUSA300_2616	**0,6**	0,002104781	Yes	171	192	193	40	52	40	79	72	85	cobalt transport family protein	SAUSA300_2616
SAUSA300_1471	**0,6**	3,39E-06	Yes	1102	1033	1072	560	611	469	955	934	1054	exodeoxyribonuclease VII, small subunit	xseB
SAUSA300_1863	**0,6**	3,42E-07	Yes	3223	2742	2748	1101	960	988	2016	1923	1564	conserved hypothetical protein	SAUSA300_1863
SAUSA300_1070	**0,6**	0,000629915	Yes	1001	691	796	733	672	560	1320	1130	1131	acetyltransferase, GNAT family	SAUSA300_1070
SAUSA300_1095	**0,6**	0,001950218	Yes	121	148	121	378	331	416	797	570	695	carbamoyl-phosphate synthase, small subunit	carA
SAUSA300_0336	**0,6**	5,99E-05	Yes	165	130	151	89	131	74	161	194	165	conserved hypothetical protein	SAUSA300_0336
SAUSA300_1065	**0,6**	0,150362496	No	64	51	114	29	25	15	28	59	48	exfoliative toxin A	SAUSA300_1065
SAUSA300_0800	**0,6**	0,000563032	Yes	632	519	617	354	412	263	589	548	739	staphylococcal enterotoxin K	sek
SAUSA300_0683	**0,6**	0,00106339	Yes	484	467	860	511	460	417	705	981	859	transcriptional regulator, DeoR family	SAUSA300_0683
SAUSA300_0969	**0,6**	0,000319468	Yes	174	318	254	206	153	251	420	282	427	phosphoribosylformylglycinamidine synthase	purS
SAUSA300_0353	**0,6**	2,69E-07	Yes	854	955	964	636	638	560	1245	1058	1029	conserved hypothetical protein	SAUSA300_0353
SAUSA300_1685	**0,6**	1,04E-05	Yes	19635	13249	13798	5124	4296	4532	9779	8570	7137	conserved hypothetical protein	SAUSA300_1685
SAUSA300_0830	**0,6**	1,44E-06	Yes	420	411	419	258	250	254	514	380	500	conserved hypothetical protein	SAUSA300_0830
SAUSA300_1560	**0,5**	6,96E-05	Yes	117	137	149	67	106	95	148	157	180	conserved hypothetical protein	SAUSA300_1560
SAUSA300_0664	**0,5**	3,61E-09	Yes	2659	1885	2055	1061	989	960	1948	1874	1693	conserved hypothetical protein	SAUSA300_0664
SAUSA300_1796	**0,5**	2,93E-06	Yes	971	678	747	352	311	266	602	513	594	conserved hypothetical protein	SAUSA300_1796
SAUSA300_0049	**0,5**	0,037973242	No	50	23	46	23	32	12	38	37	48	hypothetical protein	SAUSA300_0049
SAUSA300_1582	**0,5**	0,003900039	Yes	2177	1589	1996	543	471	593	1224	1026	752	conserved hypothetical protein	SAUSA300_1582
SAUSA300_2376	**0,5**	0,044726334	No	36	25	56	16	16	16	27	24	41	conserved hypothetical protein	SAUSA300_2376
SAUSA300_0301	**0,5**	0,013628624	No	21	42	58	33	32	34	65	48	71	conserved hypothetical protein	SAUSA300_0301
SAUSA300_1906	**0,5**	4,13E-06	Yes	1326	1102	1497	779	744	544	1174	1193	1438	conserved hypothetical protein	SAUSA300_1906
SAUSA300_2543	**0,5**	0,000971681	Yes	282	223	217	107	115	92	196	207	181	conserved hypothetical protein	SAUSA300_2543
SAUSA300_1638	**0,5**	4,65E-06	Yes	1571	1495	1504	796	825	627	1338	1338	1478	sensory box histidine kinase PhoR	phoR
SAUSA300_2632	**0,5**	0,0071583	Yes	236	157	263	50	90	75	142	109	153	putative membrane protein	SAUSA300_2632
SAUSA300_0385	**0,5**	4,49E-08	Yes	639	517	585	494	417	435	842	840	816	conserved hypothetical protein	SAUSA300_0385
SAUSA300_0960	**0,5**	2,19E-10	Yes	7477	7319	7137	2952	2364	1965	4186	4712	4553	quinol oxidase, subunit IV	qoxD
SAUSA300_2315	**0,5**	7,55E-08	Yes	2809	2313	2090	1189	1030	938	1961	1839	2054	putative lipoprotein	SAUSA300_2315
SAUSA300_2463	**0,5**	0,233365953	No	2790	2757	9543	2541	2459	2301	2290	11336	2565	D-lactate dehydrogenase	ddh
SAUSA300_1436	**0,5**	0,000896613	Yes	739	836	683	351	313	278	616	533	621	phiSLT ORF144-like protein, putative lipoprotein	SAUSA300_1436
SAUSA300_1765	**0,5**	0,016738694	No	34	48	51	27	16	12	35	34	41	lantibiotic epidermin biosynthesis protein EpiC	epiC
SAUSA300_1445	**0,5**	1,38E-06	Yes	801	699	777	397	449	313	764	667	718	segregation and condensation protein A	scpA
SAUSA300_0266	**0,5**	0,242718767	No	28	5	23	9	7	18	25	17	32	conserved hypothetical protein	SAUSA300_0266
SAUSA300_1386	**0,5**	0,306981131	No	0	4	2	3	0	0	4	3	3	phiETA ORF59-like protein	SAUSA300_1386
SAUSA300_0714	**0,5**	1,24E-08	Yes	701	574	660	359	379	272	635	601	638	Integral membrane protein	SAUSA300_0714
SAUSA300_0070	**0,5**	3,46E-05	Yes	1507	1244	1145	422	338	424	827	741	670	putative lysophospholipase	SAUSA300_0070
SAUSA300_1503	**0,5**	0,141975069	No	14	14	16	7	14	6	21	14	17	putative competence protein ComGB	SAUSA300_1503
SAUSA300_2031	**0,5**	9,23E-05	Yes	103	151	155	84	61	65	118	128	155	conserved hypothetical protein	SAUSA300_2031
SAUSA300_1842	**0,5**	0,00025227	Yes	3917	2328	2765	1458	1510	1196	2710	2231	2925	transcriptional regulator, Fur family	SAUSA300_1842
SAUSA300_2274	**0,5**	0,040043238	No	43	40	99	37	36	13	50	47	71	putative membrane protein	SAUSA300_2274
SAUSA300_0767	**0,5**	0,001392882	Yes	491	319	275	203	106	127	303	249	290	conserved hypothetical protein	SAUSA300_0767

SAUSA300_2271	**0,5**	6,34E-06	Yes	391	320	352	168	178	136	296	270	343	phosphosugar-binding transcriptional regulator	SAUSA300_2271
SAUSA300_2624	**0,5**	4,78E-06	Yes	134	156	183	119	97	78	184	178	197	putative membrane protein	SAUSA300_2624
SAUSA300_1980	**0,5**	0,022832548	No	38	41	55	10	20	16	28	28	34	acetyltransferase, GNAT family	SAUSA300_1980
SAUSA300_0423	**0,5**	0,204820208	No	3	4	5	1	0	7	5	8	11	conserved hypothetical protein	SAUSA300_0423
SAUSA300_1721	**0,5**	4,38E-06	Yes	436	411	412	242	239	152	386	359	459	conserved hypothetical protein	SAUSA300_1721
SAUSA300_0792	**0,5**	0,230316742	No	7	5	6	4	0	0	7	5	4	conserved hypothetical protein	SAUSA300_0792
SAUSA300_1276	**0,5**	4,17E-05	Yes	98	83	89	115	122	83	190	161	255	oligopeptide permease, channel-forming protein	opp-2B
SAUSA300_1618	**0,5**	9,49E-06	Yes	1032	866	1028	467	385	420	784	804	851	hemA concentration negative effector hemX	hemX
SAUSA300_2257	**0,5**	7,04E-23	Yes	2074	1915	2038	908	780	818	1691	1561	1532	conserved hypothetical protein	SAUSA300_2257
SAUSA300_1375	**0,5**	0,273008429	No	7	4	15	4	0	0	4	4	7	conserved hypothetical protein	SAUSA300_1375
SAUSA300_0276	**0,5**	0,005680398	Yes	169	83	149	69	81	64	122	133	158	putative membrane protein	SAUSA300_0276
SAUSA300_1767	**0,5**	0,090336461	No	21	8	28	10	7	7	24	14	19	lantibiotic epidermin biosynthesis protein EpiA	epiA
SAUSA300_0964	**0,5**	8,10E-09	Yes	5012	3744	3296	945	782	763	1706	1394	1677	chitinase-related protein	SAUSA300_0964
SAUSA300_2423	**0,5**	0,230549578	No	537	277	1178	165	99	81	145	533	185	conserved hypothetical protein	SAUSA300_2423
SAUSA300_1437	**0,5**	1,12E-07	Yes	892	653	849	218	171	161	370	350	343	phiSLT ORF204-like protein	SAUSA300_1437
SAUSA300_0296	**0,5**	0,006722471	Yes	29	52	63	33	25	47	61	64	87	conserved hypothetical protein	SAUSA300_0296
SAUSA300_1001	**0,5**	0,005522725	Yes	381	293	418	135	169	114	242	217	356	spermidine/putrescine ABC transporter, permease prot	potC
SAUSA300_1514	**0,5**	0,011284007	No	112	93	189	66	95	59	135	121	173	ferric uptake regulation protein	fur
SAUSA300_0603	**0,5**	1,38E-05	Yes	350	278	354	206	261	191	410	420	434	conserved hypothetical protein	SAUSA300_0603
SAUSA300_2466	**0,5**	0,000240909	Yes	78	37	46	73	63	61	118	115	151	putative membrane protein	SAUSA300_2466
SAUSA300_0093	**0,5**	0,001222284	Yes	129	94	126	74	88	68	156	114	179	transcriptional regulator, LysR family domain protein	SAUSA300_0093
SAUSA300_0378	**0,5**	0,009308603	Yes	167	100	190	90	122	56	161	160	208	conserved hypothetical protein	SAUSA300_0378
SAUSA300_1766	**0,5**	0,003096277	Yes	103	74	85	40	38	27	69	55	86	lantibiotic epidermin biosynthesis protein EpiB	epiB
SAUSA300_2448	**0,5**	0,000296411	Yes	1328	708	875	1372	1170	948	2488	2049	2301	putative membrane protein	SAUSA300_2448
SAUSA300_2061	**0,5**	1,16E-11	Yes	4325	4498	5108	2952	3135	2367	5194	5402	5763	ATP synthase F1, delta subunit	atpH
SAUSA300_0286	**0,5**	1,06E-05	Yes	148	144	142	102	110	135	247	202	229	conserved hypothetical protein	SAUSA300_0286
SAUSA300_2336	**0,5**	0,003826819	Yes	45	62	53	30	29	15	39	54	52	transcriptional regulator, MerR family	SAUSA300_2336
SAUSA300_0661	**0,5**	4,27E-07	Yes	341	234	285	139	158	111	241	255	291	conserved hypothetical protein	SAUSA300_0661
SAUSA300_2647	**0,5**	0,000268381	Yes	86	73	83	83	74	67	164	128	150	ribonuclease P protein component	rnpA
SAUSA300_1337	**0,5**	1,62E-06	Yes	4213	3220	4439	2247	2191	1735	3844	3828	4418	conserved hypothetical protein	SAUSA300_1337
SAUSA300_2405	**0,5**	0,01015337	No	229	168	352	109	178	92	236	222	297	putative membrane protein	SAUSA300_2405
SAUSA300_0068	**0,5**	NA	NA	3	1	4	3	2	1	8	4	9	cadmium-exporting ATPase, truncation	SAUSA300_0068
SAUSA300_0642	**0,5**	0,000700427	Yes	646	423	715	231	293	257	486	421	646	conserved hypothetical protein	SAUSA300_0642
SAUSA300_0597	**0,5**	0,011235814	No	28	42	30	19	16	19	36	41	35	putative endonuclease III	SAUSA300_0597
SAUSA300_2637	**0,5**	0,175958661	No	9	7	20	7	5	7	14	14	17	conserved hypothetical protein	SAUSA300_2637
SAUSA300_1919	**0,5**	0,002019609	Yes	4192	2035	2172	538	733	461	1221	996	1256	conserved hypothetical protein	SAUSA300_1919
SAUSA300_1794	**0,5**	0,114016979	No	10	11	7	6	9	1	11	11	11	conserved hypothetical protein	SAUSA300_1794
SAUSA300_0098	**0,5**	6,39E-09	Yes	298	301	357	126	122	123	257	225	250	conserved hypothetical protein	SAUSA300_0098
SAUSA300_0549	**0,5**	9,13E-13	Yes	739	753	791	339	352	312	642	677	652	glycosyl transferase, group 1 family protein	SAUSA300_0549
SAUSA300_0973	**0,5**	1,16E-11	Yes	2895	4413	3716	2025	2123	2498	4380	4195	4527	phosphoribosylformylglycinamidine cyclo-ligase	purM
SAUSA300_0409	**0,5**	0,000401314	Yes	2398	1199	1043	1635	1875	1667	3638	2864	3846	conserved hypothetical protein	SAUSA300_0409
SAUSA300_1723	**0,5**	0,065256253	No	167	68	259	53	88	50	115	95	189	conserved hypothetical protein	SAUSA300_1723
SAUSA300_0906	**0,5**	3,18E-08	Yes	510	604	524	278	241	183	446	459	487	conserved hypothetical protein	SAUSA300_0906
SAUSA300_0198	**0,5**	8,77E-06	Yes	422	486	413	205	180	117	309	338	354	conserved hypothetical protein	SAUSA300_0198
SAUSA300_2545	**0,5**	0,075648894	No	293	348	223	1272	940	1548	2231	1717	4109	choline dehydrogenase	betA
SAUSA300_1303	**0,5**	4,78E-06	Yes	496	464	602	319	286	217	523	526	596	conserved hypothetical protein	SAUSA300_1303
SAUSA300_1469	**0,5**	3,64E-06	Yes	422	381	545	308	343	257	559	600	651	arginine repressor	argR
SAUSA300_0152	**0,5**	0,014916449	No	17	11	8	23	18	19	37	40	49	capsular polysaccharide biosynthesis protein Cap5A	cap5A
SAUSA300_1508	**0,5**	0,002582422	Yes	59	60	98	49	36	38	71	72	110	conserved hypothetical protein	SAUSA300_1508
SAUSA300_0584	**0,5**	0,000431374	Yes	133	96	119	39	56	27	85	68	86	conserved hypothetical protein	SAUSA300_0584
SAUSA300_2005	**0,5**	2,75E-09	Yes	432	427	410	294	322	232	606	483	597	conserved hypothetical protein	SAUSA300_2005
SAUSA300_1092	**0,5**	0,003826819	Yes	78	86	85	266	237	336	662	403	670	uracil permease	pyrP
SAUSA300_2592	**0,5**	0,000116619	Yes	1104	743	736	266	214	263	566	563	390	conserved hypothetical protein	SAUSA300_2592
SAUSA300_0975	**0,5**	1,20E-17	Yes	6330	8841	7105	4091	4206	4623	8627	8358	8947	bifunctional purine biosynthesis protein	purH
SAUSA300_2326	**0,5**	0,001528219	Yes	570	416	610	137	167	111	244	277	329	transcription regulatory protein	SAUSA300_2326
SAUSA300_1789	**0,5**	0,099060503	No	43	12	82	23	29	22	58	31	76	conserved hypothetical protein	SAUSA300_1789
SAUSA300_1743	**0,5**	0,013423826	No	78	44	82	32	20	18	42	68	39	conserved hypothetical protein	SAUSA300_1743
SAUSA300_1223	**0,5**	2,18E-06	Yes	1262	994	1338	788	703	581	1410	1254	1545	conserved hypothetical protein	SAUSA300_1223
SAUSA300_0392	**0,5**	0,035352329	No	31	21	34	14	16	1	24	17	26	conserved hypothetical protein	SAUSA300_0392

SAUSA300_0681	**0,5**	0,07666924	No	327	189	567	163	295	229	411	328	786	conserved hypothetical protein	SAUSA300_0681
SAUSA300_0711	**0,5**	2,77E-06	Yes	1808	1162	1204	382	334	383	819	834	611	conserved hypothetical protein	SAUSA300_0711
SAUSA300_0375	**0,5**	1,93E-08	Yes	468	381	406	332	298	197	565	552	571	putative phosphoglycerate mutase family protein	SAUSA300_0375
SAUSA300_1041	**0,5**	0,065046185	No	150	64	232	40	88	55	121	84	203	conserved hypothetical protein	SAUSA300_1041
SAUSA300_1307	**0,5**	2,09E-09	Yes	2070	1859	1685	444	417	383	873	778	909	sensor histidine kinase protein	arlS
SAUSA300_2603	**0,5**	6,10E-05	Yes	257	281	207	235	187	195	411	343	537	triacylglycerol lipase precursor	lip
SAUSA300_2024	**0,5**	1,26E-11	Yes	1591	1511	1694	451	408	361	950	819	750	anti-sigma-B factor, antagonist	rsbV
SAUSA300_0778	**0,5**	1,09E-07	Yes	301	214	250	130	122	112	224	266	267	conserved hypothetical protein	SAUSA300_0778
SAUSA300_1571	**0,5**	1,36E-07	Yes	677	531	620	239	257	214	482	419	568	O-methyltransferase family protein	SAUSA300_1571
SAUSA300_2182	**0,5**	1,49E-17	Yes	2701	2974	3260	2390	2342	1775	4433	4669	4344	translation initiation factor IF-1	infA
SAUSA300_2310	**0,5**	0,144432956	No	22	12	51	6	16	12	17	17	49	conserved hypothetical protein	SAUSA300_2310
SAUSA300_1085	**0,5**	2,86E-09	Yes	1860	1677	1708	859	978	587	1584	1614	1847	conserved hypothetical protein	SAUSA300_1085
SAUSA300_0795	**0,5**	0,023135013	No	43	36	64	17	25	15	37	33	56	putative thioredoxin	SAUSA300_0795
SAUSA300_1797	**0,5**	5,09E-17	Yes	3567	2755	2937	1550	1571	1227	2984	3035	3044	conserved hypothetical protein	SAUSA300_1797
SAUSA300_1844	**0,5**	2,62E-05	Yes	525	490	527	355	397	211	635	633	766	bacterioferritin comigratory protein	SAUSA300_1844
SAUSA300_0938	**0,5**	0,006908982	Yes	71	36	93	21	25	41	54	56	83	conserved hypothetical protein	SAUSA300_0938
SAUSA300_1977	**0,5**	0,019169505	No	31	31	57	17	29	18	47	39	52	conserved hypothetical protein	SAUSA300_1977
SAUSA300_2181	**0,5**	4,80E-11	Yes	2968	3232	3187	2233	2308	1745	4233	4146	4821	50S ribosomal protein L36	rpmJ
SAUSA300_2331	**0,5**	3,61E-09	Yes	3551	2287	2375	534	600	427	1163	1035	1079	transcriptional regulator, MarR family	SAUSA300_2331
SAUSA300_0949	**0,5**	0,004258692	Yes	110	63	95	20	34	15	41	44	63	cysteine protease	sspC
SAUSA300_2142	**0,5**	3,34E-11	Yes	44290	42359	44052	10460	8892	12108	23546	22741	20180	alkaline shock protein 23	asp23
SAUSA300_0790	**0,5**	1,04E-05	Yes	379	368	664	271	352	226	573	597	636	putative arsenate reductase	SAUSA300_0790
SAUSA300_1335	**0,5**	7,00E-08	Yes	212	168	221	123	142	123	288	251	281	conserved hypothetical protein	SAUSA300_1335
SAUSA300_2403	**0,5**	0,000427061	Yes	1211	827	954	341	277	203	629	479	674	putative lipoprotein	SAUSA300_2403
SAUSA300_1970	**0,5**	9,93E-08	Yes	630	691	517	206	165	206	428	353	460	putative exonuclease	SAUSA300_1970
SAUSA300_1204	**0,5**	0,04180078	No	59	56	72	21	16	4	29	25	44	conserved hypothetical protein	SAUSA300_1204
SAUSA300_0282	**0,5**	1,21E-06	Yes	741	542	419	358	307	340	869	561	744	conserved hypothetical protein	SAUSA300_0282
SAUSA300_1594	**0,5**	5,20E-11	Yes	2153	1545	2191	1139	1242	938	2448	2234	2427	preprotein translocase, YajC subunit	yajC
SAUSA300_0354	**0,5**	1,76E-05	Yes	909	642	984	168	212	138	336	367	412	low temperature requirement protein LtrA	ltrA
SAUSA300_2325	**0,5**	7,46E-05	Yes	34	15	33	37	34	34	69	87	76	conserved hypothetical protein	SAUSA300_2325
SAUSA300_0558	**0,5**	4,99E-08	Yes	5098	2884	3287	4176	3744	3731	8044	8803	8367	putative proline/betaine transporter	SAUSA300_0558
SAUSA300_2354	**0,5**	4,13E-10	Yes	307	338	384	115	151	111	220	279	301	putative lipoprotein	SAUSA300_2354
SAUSA300_0725	**0,5**	3,96E-07	Yes	1678	1280	1593	735	708	592	1625	1406	1404	conserved hypothetical protein	SAUSA300_0725
SAUSA300_1546	**0,5**	5,19E-05	Yes	276	164	210	106	122	92	257	203	241	DNA polymerase III, delta subunit	holA
SAUSA300_2293	**0,5**	1,65E-07	Yes	358	352	537	143	185	130	310	287	397	magnesium and cobalt transport protein	corA
SAUSA300_0837	**0,5**	7,46E-08	Yes	1061	891	1180	530	518	417	1100	996	1122	D-alanine-activating enzyme/D-alanine-D-alanyl, dltC pr	dltC
SAUSA300_0550	**0,5**	1,40E-07	Yes	1371	986	1282	617	591	408	1103	1238	1212	glycosyl transferase, group 1 family protein	SAUSA300_0550
SAUSA300_2080	**0,5**	1,04E-11	Yes	1133	819	1013	361	316	277	721	662	711	conserved hypothetical protein	SAUSA300_2080
SAUSA300_1726	**0,5**	7,23E-05	Yes	198	108	174	70	79	52	140	170	134	crcB family protein	SAUSA300_1726
SAUSA300_0374	**0,5**	2,38E-05	Yes	12327	12681	14784	3817	4249	5727	11551	11111	8149	putative membrane protein	SAUSA300_0374
SAUSA300_2546	**0,5**	0,037187768	No	344	331	236	1411	1044	1386	2562	2041	4656	glycine betaine aldehyde dehydrogenase	betB
SAUSA300_1217	**0,5**	0,130359283	No	16	14	14	1	2	0	6	3	6	ABC transporter, ATP-binding protein	SAUSA300_1217
SAUSA300_1868	**0,5**	3,16E-07	Yes	265	203	263	142	189	177	332	328	471	conserved hypothetical protein	SAUSA300_1868
SAUSA300_0095	**0,5**	0,001111677	Yes	43	40	60	16	27	25	55	45	55	transcriptional regulator, LysR family domain protein	SAUSA300_0095
SAUSA300_2562	**0,5**	0,136598918	No	40	37	125	29	43	35	93	58	167	conserved hypothetical protein	SAUSA300_2562
SAUSA300_0154	**0,5**	6,66E-06	Yes	36	21	22	49	52	35	102	87	116	capsular polysaccharide biosynthesis protein Cap5C	cap5C
SAUSA300_1334	**0,4**	0,001024818	Yes	574	292	419	222	176	183	394	510	445	putative membrane protein	SAUSA300_1334
SAUSA300_0974	**0,4**	1,54E-20	Yes	1982	2775	2547	1226	1379	1328	2963	2788	3061	phosphoribosylglycinamide formyltransferase	purN
SAUSA300_2144	**0,4**	8,26E-12	Yes	18458	17978	17432	3176	2518	3574	7491	7015	6390	conserved hypothetical protein	SAUSA300_2144
SAUSA300_0779	**0,4**	8,55E-08	Yes	229	162	177	84	88	71	184	175	192	conserved hypothetical protein	SAUSA300_0779
SAUSA300_0428	**0,4**	0,162738081	No	2	4	2	0	0	1	3	5	2	conserved hypothetical protein	SAUSA300_0428
SAUSA300_1215	**0,4**	0,174984215	No	0	7	6	0	0	1	5	5	1	conserved hypothetical protein	SAUSA300_1215
SAUSA300_1607	**0,4**	3,81E-06	Yes	470	326	399	271	440	214	733	701	662	conserved hypothetical protein	SAUSA300_1607
SAUSA300_0701	**0,4**	1,01E-07	Yes	515	534	672	198	225	124	403	367	467	conserved hypothetical protein TIGR00370	SAUSA300_0701
SAUSA300_2639	**0,4**	0,001266203	Yes	52585	20831	31930	15030	20373	13062	46996	37194	29649	cold shock protein	SAUSA300_2639
SAUSA300_1129	**0,4**	0,000343636	Yes	96	114	230	99	135	80	221	209	296	conserved hypothetical protein	SAUSA300_1129
SAUSA300_0160	**0,4**	2,01E-05	Yes	60	36	46	36	29	34	85	67	80	capsular polysaccharide biosynthesis protein Cap5I	cap5I
SAUSA300_2481	**0,4**	3,01E-05	Yes	5382	4957	5409	2276	4918	2135	8492	7316	5770	conserved hypothetical protein	SAUSA300_2481
SAUSA300_0735	**0,4**	0,007731	Yes	36	25	29	9	11	15	30	30	27	competence protein F	SAUSA300_0735

SAUSA300_2313	**0,4**	NA	NA	1903	1847	11133	617	539	577	633	4707	825	L-lactate permease	SAUSA300_2313
SAUSA300_0939	**0,4**	1,99E-08	Yes	1347	1274	1728	607	676	500	1267	1342	1479	glycosyl transferase, group 1 family protein	SAUSA300_0939
SAUSA300_0291	**0,4**	0,021894929	No	36	22	48	19	25	27	47	46	81	putative membrane protein	SAUSA300_0291
SAUSA300_2362	**0,4**	1,59E-07	Yes	13278	10202	13210	4260	2612	3431	6408	7075	10268	2,3-bisphosphoglycerate-dependent phosphoglycerate	gpmA
SAUSA300_0067	**0,4**	0,08261263	No	117	42	180	39	18	22	67	52	110	universal stress protein family	SAUSA300_0067
SAUSA300_1339	**0,4**	8,16E-05	Yes	727	549	867	460	552	290	950	874	1219	conserved hypothetical protein	SAUSA300_1339
SAUSA300_2263	**0,4**	0,001850517	Yes	24	25	21	17	11	13	39	33	32	putative transposase	SAUSA300_2263
SAUSA300_0929	**0,4**	4,06E-05	Yes	203	298	314	87	108	96	206	261	219	conserved hypothetical protein	SAUSA300_0929
SAUSA300_0363	**0,4**	6,30E-07	Yes	117	141	226	107	92	89	222	209	243	conserved hypothetical protein	SAUSA300_0363
SAUSA300_2164	**0,4**	0,002637747	Yes	119	96	82	24	27	47	100	65	90	conserved hypothetical protein	SAUSA300_2164
SAUSA300_0784	**0,4**	1,04E-08	Yes	820	732	799	162	153	111	336	341	318	LysE/YggA family protein	SAUSA300_0784
SAUSA300_0279	**0,4**	4,73E-07	Yes	3498	1834	1660	1531	1246	1153	3323	2617	3299	putative membrane protein	SAUSA300_0279
SAUSA300_0134	**0,4**	1,37E-06	Yes	248	145	249	90	115	95	208	232	265	polysaccharide extrusion protein	SAUSA300_0134
SAUSA300_0648	**0,4**	4,75E-11	Yes	1157	892	1077	520	575	455	1188	1089	1356	ABC transporter, permease protein	SAUSA300_0648
SAUSA300_1258	**0,4**	2,18E-05	Yes	610	736	814	402	471	500	1414	932	939	4-oxalocrotonate tautomerase	SAUSA300_1258
SAUSA300_0990	**0,4**	2,05E-15	Yes	1402	1103	1274	528	552	464	1151	1158	1306	conserved hypothetical protein	SAUSA300_0990
SAUSA300_1729	**0,4**	2,35E-10	Yes	6054	4840	5415	1747	1557	1184	3433	3312	3824	conserved hypothetical protein	SAUSA300_1729
SAUSA300_1473	**0,4**	5,61E-09	Yes	1502	1235	1710	541	789	512	1367	1311	1653	transcription antitermination factor NusB	nusB
SAUSA300_1864	**0,4**	2,99E-15	Yes	9755	7575	7007	1509	1352	1532	3551	3440	3352	putative membrane protein	SAUSA300_1864
SAUSA300_1253	**0,4**	2,10E-05	Yes	253	249	344	110	131	80	220	225	320	transcription antiterminator	glcT
SAUSA300_1554	**0,4**	3,49E-06	Yes	375	345	551	140	210	114	342	314	441	conserved hypothetical protein	SAUSA300_1554
SAUSA300_0604	**0,4**	9,20E-11	Yes	920	760	917	316	264	348	743	758	717	hydrolase, alpha/beta hydrolase fold family	SAUSA300_0604
SAUSA300_0402	**0,4**	0,041859648	No	9	11	13	7	0	3	14	9	11	exotoxin	SAUSA300_0402
SAUSA300_1240	**0,4**	3,13E-09	Yes	2740	2397	2948	1222	1427	972	2986	2787	2892	conserved hypothetical protein	SAUSA300_1240
SAUSA300_1741	**0,4**	0,00314569	Yes	47	26	30	6	14	13	28	32	23	putative lipoprotein	SAUSA300_1741
SAUSA300_2289	**0,4**	8,27E-11	Yes	419	235	282	200	180	191	441	456	485	conserved hypothetical protein	SAUSA300_2289
SAUSA300_0625	**0,4**	4,23E-07	Yes	2670	1703	2541	1133	1201	741	2350	2272	2855	teichoic acid ABC transporter protein	tagG
SAUSA300_0957	**0,4**	7,36E-07	Yes	2578	1890	2594	1242	1769	875	2900	2955	3596	conserved hypothetical protein	SAUSA300_0957
SAUSA300_0391	**0,4**	0,005819984	Yes	45	53	30	16	7	6	29	25	26	conserved hypothetical protein	SAUSA300_0391
SAUSA300_0172	**0,4**	0,002104597	Yes	115	68	69	24	34	35	95	89	60	conserved hypothetical protein	SAUSA300_0172
SAUSA300_1046	**0,4**	4,56E-07	Yes	160	185	261	84	61	58	149	198	163	succinate dehydrogenase, cytochrome b-558 subunit	sdhC
SAUSA300_1093	**0,4**	0,000223104	Yes	57	88	64	199	147	204	491	296	612	aspartate carbamoyltransferase	pyrB
SAUSA300_0761	**0,4**	0,011389805	No	133	73	268	42	41	35	84	91	143	conserved hypothetical protein	SAUSA300_0761
SAUSA300_0913	**0,4**	1,52E-14	Yes	3917	3265	4456	801	856	608	1698	1830	2025	putative membrane protein	SAUSA300_0913
SAUSA300_1141	**0,4**	0,005732038	Yes	36	21	60	17	23	13	37	44	59	endopeptidase resistance gene	SAUSA300_1141
SAUSA300_2261	**0,4**	1,63E-14	Yes	1130	854	998	566	541	410	1163	1219	1348	conserved hypothetical protein	SAUSA300_2261
SAUSA300_0197	**0,4**	3,01E-06	Yes	482	408	539	193	160	105	368	365	423	conserved hypothetical protein	SAUSA300_0197
SAUSA300_2328	**0,4**	0,00333266	Yes	375	185	434	107	142	59	243	234	341	conserved hypothetical protein	SAUSA300_2328
SAUSA300_0620	**0,4**	8,00E-09	Yes	48549	46442	53199	9953	5966	8778	16440	17941	27928	ABC transporter ATP-binding protein	SAUSA300_0620
SAUSA300_1764	**0,4**	0,067446795	No	3	5	13	1	0	1	6	4	8	lantibiotic epidermin biosynthesis protein EpiD	epiD
SAUSA300_0529	**0,4**	5,06E-07	Yes	3574	3266	4193	1328	1557	671	2986	2994	3061	conserved hypothetical protein	SAUSA300_0529
SAUSA300_0639	**0,4**	7,31E-08	Yes	5963	3552	4994	2596	2488	1786	5932	5775	5859	conserved hypothetical protein	SAUSA300_0639
SAUSA300_0205	**0,4**	1,27E-11	Yes	239	229	201	99	106	67	238	218	231	staphylococcal tandem lipoprotein	SAUSA300_0205
SAUSA300_2252	**0,4**	3,18E-08	Yes	661	359	422	145	142	102	370	303	333	conserved hypothetical protein	SAUSA300_2252
SAUSA300_0297	**0,4**	0,009373568	Yes	16	16	27	4	9	7	16	23	22	putative lipoprotein	SAUSA300_0297
SAUSA300_0831	**0,4**	1,57E-14	Yes	465	413	441	248	275	189	628	564	639	conserved hypothetical protein	SAUSA300_0831
SAUSA300_0619	**0,4**	1,34E-08	Yes	30117	30046	36494	6801	4233	5655	11193	12470	20231	ABC transporter, permease protein	SAUSA300_0619
SAUSA300_1372	**0,4**	2,77E-05	Yes	251	138	256	64	61	58	147	152	197	conserved hypothetical protein	SAUSA300_1372
SAUSA300_1208	**0,4**	0,091708478	No	9	4	34	0	5	9	12	14	36	conserved hypothetical protein	SAUSA300_1208
SAUSA300_0898	**0,4**	1,59E-11	Yes	28457	15653	19490	3119	2750	2505	7542	7243	7159	Regulatory protein spx	spxA
SAUSA300_2648	**0,4**	2,04E-12	Yes	9745	5753	7647	4505	5630	4405	13826	12368	11880	50S ribosomal protein L34	rpmH
SAUSA300_2262	**0,4**	0,000109506	Yes	667	424	987	463	638	383	1187	1182	1653	putative membrane protein	SAUSA300_2262
SAUSA300_0649	**0,4**	5,06E-07	Yes	878	704	1132	378	458	293	913	888	1203	conserved hypothetical protein	SAUSA300_0649
SAUSA300_2154	**0,4**	0,013708347	No	7	3	11	9	5	12	29	23	24	galactose-6-phosphate isomerase	lacB
SAUSA300_0931	**0,4**	1,58E-09	Yes	718	524	758	496	530	327	1315	1220	1068	conserved hypothetical protein	SAUSA300_0931
SAUSA300_2418	**0,4**	5,68E-09	Yes	1430	1129	1423	271	214	277	757	664	635	conserved hypothetical protein	SAUSA300_2418
SAUSA300_0959	**0,4**	6,51E-09	Yes	1137	817	1222	485	491	342	1110	1069	1383	fmt protein	fmt
SAUSA300_2132	**0,4**	7,56E-12	Yes	134	126	107	93	83	62	196	232	216	conserved hypothetical protein	SAUSA300_2132
SAUSA300_1057	**0,4**	6,66E-08	Yes	532	330	414	103	72	56	212	212	215	conserved hypothetical protein	SAUSA300_1057

SAUSA300_1089	**0,4**	2,68E-06	Yes	208	126	229	89	65	62	183	165	253	lipoprotein signal peptidase	lspA
SAUSA300_0271	**0,4**	8,68E-10	Yes	808	545	523	182	153	106	400	395	425	ABC transporter, ATP-binding protein	SAUSA300_0271
SAUSA300_0233	**0,4**	0,003873705	Yes	127	45	92	43	52	25	104	115	145	conserved hypothetical protein	SAUSA300_0233
SAUSA300_1392	**0,4**	0,026448261	No	16	10	6	1	7	1	11	11	11	phiSLT ORF191-like protein	SAUSA300_1392
SAUSA300_0235	**0,4**	NA	NA	730	387	26175	261	266	281	258	27193	874	L-lactate dehydrogenase	SAUSA300_0235
SAUSA300_0769	**0,4**	7,10E-08	Yes	2334	1251	1265	484	379	291	1179	1029	1027	putative lipoprotein	SAUSA300_0769
SAUSA300_0777	**0,4**	0,001117462	Yes	10995	5641	13242	4309	6730	4547	15275	17750	13094	cold shock protein	SAUSA300_0777
SAUSA300_1535	**0,4**	1,73E-07	Yes	5152	5300	6517	1952	3092	1921	7049	6998	5652	30S ribosomal protein S21	rpsU
SAUSA300_0519	**0,4**	0,00118442	Yes	171	122	390	70	137	87	231	261	393	conserved hypothetical protein	SAUSA300_0519
SAUSA300_0274	**0,4**	9,50E-12	Yes	3567	2175	3338	939	856	668	2228	2333	2355	conserved hypothetical protein	SAUSA300_0274
SAUSA300_1492	**0,4**	1,51E-05	Yes	346	283	481	185	248	127	532	451	638	putative lipoprotein	SAUSA300_1492
SAUSA300_1787	**0,4**	7,51E-08	Yes	503	486	748	162	137	118	363	390	452	HIT family protein	SAUSA300_1787
SAUSA300_2599	**0,4**	1,64E-06	Yes	496	333	672	338	277	237	850	822	806	intercellular adhesion operon transcription regulator, Te	tetR
SAUSA300_1717	**0,4**	0,000102785	Yes	76	57	78	36	38	18	78	87	106	arsenical resistance operon repressor	arsR
SAUSA300_2100	**0,4**	1,64E-05	Yes	3484	2016	3559	2025	2466	1461	5030	4925	7538	lytic regulatory protein	SAUSA300_2100
SAUSA300_1224	**0,4**	1,50E-05	Yes	231	136	226	64	108	59	203	211	262	conserved hypothetical protein	SAUSA300_1224
SAUSA300_2332	**0,4**	0,000132404	Yes	386	200	348	143	189	86	392	394	485	heat shock protein	SAUSA300_2332
SAUSA300_1295	**0,4**	1,20E-10	Yes	22868	17617	25509	5886	7954	5625	21141	19423	16261	cold shock protein, CSD family	SAUSA300_1295
SAUSA300_0305	**0,3**	NA	NA	62	68	1663	46	43	58	89	1444	143	formate/nitrite transporter family protein	SAUSA300_0305
SAUSA300_1762	**0,3**	2,20E-07	Yes	172	127	175	29	38	30	104	108	81	lantibiotic epidermin immunity protein F	epiF
SAUSA300_0605	**0,3**	0,002524811	Yes	1831	809	2046	782	1095	736	2962	2274	3378	staphylococcal accessory regulator A	sarA
SAUSA300_2330	**0,3**	1,22E-08	Yes	351	238	421	113	126	81	331	298	328	conserved hypothetical protein	SAUSA300_2330
SAUSA300_0376	**0,3**	1,23E-09	Yes	326	193	310	116	97	53	241	265	291	conserved hypothetical protein	SAUSA300_0376
SAUSA300_0033	**0,3**	7,38E-07	Yes	4692	3136	5965	1448	1878	1056	3806	4185	5323	methicillin-resistance MecR1 regulatory protein	SAUSA300_0033
SAUSA300_0280	**0,3**	1,97E-08	Yes	417	229	226	156	124	80	393	312	381	conserved hypothetical protein	SAUSA300_0280
SAUSA300_1918	**0,3**	0,001083139	Yes	269	86	61	56	54	33	231	109	136	truncated beta-hemolysin	SAUSA300_1918
SAUSA300_1803	**0,3**	5,57E-10	Yes	1361	866	1297	169	189	152	554	479	534	conserved hypothetical protein	SAUSA300_1803
SAUSA300_2245	**0,3**	0,004504399	Yes	1245	471	1688	170	237	169	589	494	967	staphylococcal accessory regulator R	SAUSA300_2245
SAUSA300_2452	**0,3**	0,0135156	No	9	4	6	6	2	0	11	9	13	transcriptional regulator, MarR family	SAUSA300_2452
SAUSA300_1606	**0,3**	9,09E-10	Yes	196	120	204	73	83	61	222	199	251	conserved hypothetical protein	SAUSA300_1606
SAUSA300_1107	**0,3**	1,48E-10	Yes	1314	936	1335	480	559	441	1556	1454	1581	conserved hypothetical protein	SAUSA300_1107
SAUSA300_1022	**0,3**	8,75E-06	Yes	81	68	104	37	70	28	123	134	165	conserved hypothetical protein	SAUSA300_1022
SAUSA300_1210	**0,3**	3,10E-06	Yes	200	162	216	13	23	24	68	48	75	conserved hypothetical protein	SAUSA300_1210
SAUSA300_1099	**0,3**	1,67E-10	Yes	90	119	121	67	68	67	219	171	245	conserved hypothetical protein	SAUSA300_1099
SAUSA300_1499	**0,3**	2,42E-13	Yes	308	268	382	74	79	52	197	210	227	shikimate kinase	aroK
SAUSA300_1017	**0,3**	9,84E-08	Yes	1080	799	1644	329	372	259	846	1009	1215	conserved hypothetical protein	SAUSA300_1017
SAUSA300_2402	**0,3**	2,25E-15	Yes	646	597	555	308	248	198	859	703	818	conserved hypothetical protein	SAUSA300_2402
SAUSA300_2544	**0,3**	0,00033557	Yes	62	57	93	13	16	3	50	26	32	conserved hypothetical protein	SAUSA300_2544
SAUSA300_2264	**0,3**	3,05E-11	Yes	3861	2942	4056	557	485	389	1383	1335	1885	phosphosugar-binding transcriptional regulator, RpiR fa	SAUSA300_2264
SAUSA300_0127	**0,3**	8,95E-05	Yes	34	19	40	20	27	13	70	56	77	conserved hypothetical protein	SAUSA300_0127
SAUSA300_0094	**0,3**	0,036422011	No	5	4	7	0	0	0	4	5	2	conserved hypothetical protein	SAUSA300_0094
SAUSA300_0471	**0,3**	5,58E-12	Yes	6026	3888	6294	1600	1659	1238	5256	4723	5002	veg protein	SAUSA300_0471
SAUSA300_0618	**0,3**	1,15E-13	Yes	58744	53704	64154	10640	6667	8337	22062	24010	39633	ABC transporter, substrate-binding protein	SAUSA300_0618
SAUSA300_1965	**0,3**	0,036512455	No	16	7	97	4	5	0	11	11	49	conserved hypothetical phage protein	SAUSA300_1965
SAUSA300_1746	**0,3**	0,030416078	No	2	3	8	0	0	0	2	4	7	conserved hypothetical protein	SAUSA300_1746
SAUSA300_0583	**0,3**	0,000724548	Yes	69	36	74	14	9	13	44	38	64	conserved hypothetical protein	SAUSA300_0583
SAUSA300_1545	**0,3**	3,98E-15	Yes	5720	4564	5843	1915	2340	1446	7072	6320	6607	30S ribosomal protein S20	rpsT
SAUSA300_1511	**0,3**	9,19E-14	Yes	4020	3783	4231	1783	1909	1668	7393	6020	5670	50S ribosomal protein L33	rpmG
SAUSA300_0985	**0,3**	3,60E-05	Yes	133	83	139	32	68	13	135	129	162	conserved hypothetical protein	SAUSA300_0985
SAUSA300_2269	**0,3**	3,20E-07	Yes	264	182	341	39	68	28	160	156	201	conserved hypothetical protein	SAUSA300_2269
SAUSA300_2598	**0,3**	0,00777196	Yes	14	8	7	4	7	1	19	16	27	capsular polysaccharide biosynthesis protein Cap1A	cap1A
SAUSA300_0215	**0,3**	4,15E-06	Yes	167	105	218	46	56	28	160	129	237	conserved hypothetical protein	SAUSA300_0215
SAUSA300_2212	**0,3**	9,15E-06	Yes	226	149	267	57	56	13	175	174	183	conserved hypothetical protein	SAUSA300_2212
SAUSA300_1230	**0,3**	7,93E-11	Yes	503	387	508	99	156	77	442	378	483	conserved hypothetical protein	SAUSA300_1230
SAUSA300_0303	**0,3**	5,67E-07	Yes	455	162	355	96	86	53	319	231	421	putative lipoprotein	SAUSA300_0303
SAUSA300_1581	**0,3**	1,17E-09	Yes	1440	843	1826	182	140	192	729	534	840	conserved hypothetical protein	SAUSA300_1581
SAUSA300_2361	**0,3**	1,77E-05	Yes	62	37	53	14	27	9	88	69	71	conserved hypothetical protein	SAUSA300_2361
SAUSA300_0723	**0,2**	0,004093602	Yes	16	8	28	4	5	1	21	12	28	conserved hypothetical protein	SAUSA300_0723
SAUSA300_0937	**0,2**	1,34E-07	Yes	375	329	613	34	122	59	377	281	301	conserved hypothetical protein	SAUSA300_0937

SAUSA300_2401	**0,2**	2,07E-21	Yes	484	444	471	179	140	108	718	578	590	addiction module toxin, Txe/YoeB family	SAUSA300_2401
SAUSA300_0752	**0,2**	4,44E-112	Yes	8071	6606	6264	2062	2103	2045	9312	9047	8675	ATP-dependent Clp protease, proteolytic subunit ClpP	clpP
SAUSA300_0785	**0,2**	1,82E-10	Yes	1049	631	1099	113	110	58	426	372	531	acetyltransferase, GNAT family	SAUSA300_0785
SAUSA300_1021	**0,2**	1,82E-07	Yes	105	57	127	23	32	19	118	89	157	hypothetical protein	SAUSA300_1021
SAUSA300_1244	**0,2**	3,97E-21	Yes	7131	5386	8487	1454	1805	1170	6733	6409	7194	large conductance mechanosensitive channel protein	mscL
SAUSA300_0781	**0,2**	0,000521012	Yes	81	22	90	6	5	12	55	40	66	conserved hypothetical protein	SAUSA300_0781
SAUSA300_1068	**0,2**	0,001178896	Yes	1027	1680	1881	52	36	41	280	138	1201	phenol soluble modulin beta 1/beta 2	SAUSA300_1068
SAUSA300_0292	**0,2**	4,00E-08	Yes	110	52	113	23	18	13	121	86	94	conserved hypothetical protein	SAUSA300_0292
SAUSA300_1067	**0,2**	0,001278425	Yes	510	754	1176	7	14	13	113	65	551	phenol-soluble modulin beta1	SAUSA300_1067
SAUSA300_0884	**0,2**	2,86E-16	Yes	1082	955	1215	211	282	180	1384	1032	1237	conserved hypothetical protein	SAUSA300_0884
SAUSA300_0281	**0,2**	7,67E-10	Yes	91	37	53	17	11	13	85	64	108	esXB	SAUSA300_0281
SAUSA300_0816	**0,2**	5,21E-21	Yes	22268	17045	19320	1979	1510	1622	10799	9697	8628	CsbD-like superfamily	SAUSA300_0816
SAUSA300_1992	**0,2**	2,85E-14	Yes	21033	19499	20788	1219	1237	732	5196	4756	9523	accessory gene regulator protein A	agrA
SAUSA300_1432	**0,2**	4,16E-10	Yes	834	397	830	53	110	31	452	432	515	phiSLT ORF78-like protein	SAUSA300_1432
SAUSA300_1991	**0,2**	1,52E-14	Yes	25251	22148	28164	1223	1404	849	6547	5852	12274	accessory gene regulator protein C	agrC
SAUSA300_1988	**0,2**	0,000309854	Yes	11750	6178	18848	93	135	62	3024	1683	11749	rnaIII	SAUSA300_1988
SAUSA300_1989	**0,1**	8,10E-09	Yes	12997	10873	16021	523	572	336	3525	2912	6919	accessory gene regulator protein B	agrB
SAUSA300_2041	**0,1**	2,01E-11	Yes	605	281	903	50	68	41	464	357	562	conserved hypothetical protein	SAUSA300_2041
SAUSA300_1990	**0,1**	5,61E-09	Yes	4459	3544	5295	150	214	86	1369	1103	2684	accessory gene regulator protein D	agrD
SAUSA300_1180	**0,1**	7,42E-11	Yes	491	293	751	66	128	37	977	714	1174	conserved hypothetical protein	SAUSA300_1180
